# Broad-spectrum kinetic resolution of alcohols enabled by Cu–H-catalysed dehydrogenative coupling with hydrosilanes

**DOI:** 10.1038/ncomms15547

**Published:** 2017-06-01

**Authors:** Xichang Dong, Andreas Weickgenannt, Martin Oestreich

**Affiliations:** 1 Institut für Chemie, Technische Universität Berlin, Strasse des 17. Juni 115, 10623 Berlin, Germany

## Abstract

The enantioselective silylation of racemic alcohols, where one enantiomer reacts faster than the other, is an alternative approach to established enzymatic and non-enzymatic acylation techniques. The existing art is either limited to structurally biased alcohols or requires elaborate catalysts. Simple substrates, such as benzylic and allylic alcohols, with no coordinating functionality in the proximity of the hydroxy group have been challenging in these kinetic resolutions. We report here the identification of a broadly applicable chiral catalyst for the enantioselective dehydrogenative coupling of alcohols and hydrosilanes with both the chiral ligand and the hydrosilane being commercially available. The efficiency of kinetic resolutions is characterized by the selectivity factor, that is, the ratio of the reaction rates of the fast-reacting over the slow-reacting enantiomer. The selectivity factors achieved with the new method are good for acyclic benzylic alcohols (≤170) and high for synthetically usefully cyclic benzylic (≤40.1) and allylic alcohols (≤159).

## Abstract (web summary)

Kinetic resolution of alcohols by silylation is an attractive method to produce enantiopure compounds, but known systems often display limited substrate scope. Here the authors report a copper catalysed enantioselective dehydrogenative silylation of alcohols that have high selectivity across a broad range of substrates.

A common method to kinetically resolve alcohols is by acylation, either enzymatic or non-enzymatic^1^. Conversely, non-enzymatic kinetic resolution through catalytic alcohol silylation had been unknown until a decade ago (Fig. 1a)^2^,^3^,^4^. Part of the attractiveness of this transformation lies in rendering an often-used alcohol protection enantioselective. The ‘obvious' strategy of achieving this goal is by the design of chiral imidazole-based catalysts for chlorosilane activation, that is, an asymmetric version of the original Corey–Venkateswarlu protocol^5^. It was Hoveyda and Snapper^6^,^7^,^8^,^9^,^10^ and, later, Tan^11^,^12^,^13^,^14^,^15^ to introduce such catalysts that enable the kinetic resolution^7^,^9^,^12^,^15^ and, likewise, desymmetrization^6^,^8^,^10^,^11^,^13^,^14^ of 1,2-diol motifs but not monools (Strategy 1). Inspired by an early contribution by Ishikawa^16^, Wiskur used isourea-based catalysts to kinetically resolve cyclic monools with decent success (Strategy 1 and Fig. 1b)^17^,^18^,^19^,^20^,^21^. Just recently, Song^22^ presented a broadly applicable solution to the long-standing challenge of resolving simple alcohols, typically 1-phenylethan-1-ol derivatives, by Brønsted-acid catalysis (Strategy 2 and Fig. 1b). Desymmetrization of selected 1,2-diols was also demonstrated^22^,^23^.

Our laboratory had approached the problem from a different angle. We had used Cu–H catalysis to couple alcohols and hydrosilanes with release of dihydrogen (Strategy 3 and Fig. 2a)^24^,^25^. The resulting silyl ether is usually considered waste in the catalytic generation of copper(I) hydride-reducing agents. At that time, we had chosen the dehydrogenative Si–O coupling because it allowed us to employ silicon-stereogenic hydrosilanes as resolving reagents (Fig. 2b). Unlike chlorosilanes, these react without racemization with hydroxy groups. By this, we accomplished the reagent-controlled kinetic resolution of alcohols with an achiral monodentate phosphine ligand at the copper(I) atom^26^,^27^,^28^,^29^,^30^. We were later able to turn this kinetic resolution into a catalyst-controlled process with a chiral monodentate ligand and achiral hydrosilanes^31^. However, both transformations required alcohols with pending donors (TS1 and TS2, Fig. 2c), making two-point binding of the substrate the salient feature of these coupling reactions. All subsequent attempts to extend this methodology to monools had failed for years^32^,^33^ until we discovered that a chiral bidentate ligand together with trialkylsilanes having long aliphatic chains lead to high selectivity factors (TS3, Fig. 2c). We disclose here the Cu–H-catalysed enantioselective silylation of structurally non-biased alcohols, including synthetically useful allylic alcohols, with both a commercially available catalyst and hydrosilane.

## Results

### Catalyst identification and optimization

An extensive screening of chiral ligands finally culminated in the identification of commercial (*R*,*R*)-Ph-BPE [**L1**, 1,2-bis((2*R*,5*R*)-2,5-diphenylphospholano)ethane] as a superior ligand in the catalytic asymmetric Si–O coupling of 1-phenylethan-1-ol and structurally related congeners (see Supplementary Table 1 for the complete ligand survey)^32^,^34^,^35^,^36^,^37^. Systematic variation of the copper(I) source, base and solvent did not reveal any evidence of a trend, and these data are collected in Supplementary Tables 2–4. The CuCl–NaO^*t*^Bu system in toluene was subsequently used in the further optimization of the model reaction **1a**→**3a**/**1a** (Table 1). The selectivity factor was low with Ph_3_SiH (**2a**, *s*=2.96, entry 1) but at least moderate with MePh_2_SiH and Me_2_PhSiH (**2b**, *s*=5.52, entry 2 and **2c**, *s*=6.33, entry 3). However, any steric and electronic modification of the aryl groups in these diarylmethyl- and aryldimethylsilanes had little effect (*s*≈5 and *s*≈6, respectively), if at all resulting in less reactive hydrosilanes (see Supplementary Table 5 for the collection of tested hydrosilanes). We then found that linear alkyl substituents instead of the methyl group(s) dramatically improved the selectivity factor, reaching promising *s* values of 10.0 with **2d** (entry 4) and 10.6 with **2e** (entry 5). This prompted us to (re-)investigate trialkylsilanes as coupling partners. We had initially excluded these from the present study because of their lack of reactivity in our earlier catalyst-controlled kinetic resolution of donor-functionalized alcohols^31^. To our delight, trialkylsilanes **2f**–**2i** consistently yielded selectivity factors above 10 (entries 6–9), and the best value was obtained for **2h** with *n*-butyl groups (*s*=14.3, entry 8). Branching close to the silicon atom as in **2j**–**2l** was detrimental (entries 10–12), and *tert*-butyl substituents were generally not accepted (not shown). Bn_3_SiH performed also well (**2m**, *s*=11.6, entry 13) but was inferior to ^*n*^Bu_3_SiH (**2h**, cf. entry 8). Reactions were routinely run for 18 h to achieve synthetically useful conversions. To boost the reactivity^28^, we tested cyclic and, hence, more Lewis-acidic hydrosilane **2n** but the enormous reactivity gain was at the expense of selectivity (*s*=3.51, entry 14). Also, alkoxy-substituted hydrosilanes, such as (EtO)_3_SiH (**2o**), reacted rapidly yet without any asymmetric induction (entry 15). Cognate ligands (*S*,*S*)-Me-BPE (**L2**) and (*R*,*R*)-^*i*^Pr-BPE (**L3**) with different R groups either led to a catalytically inactive copper(I) complex (entry 16) or a clearly lower *s* value (entry 17) with ^*n*^Bu_3_SiH (**2h**) (cf. entry 8).

### Scope and limitations

**Figure 1:**
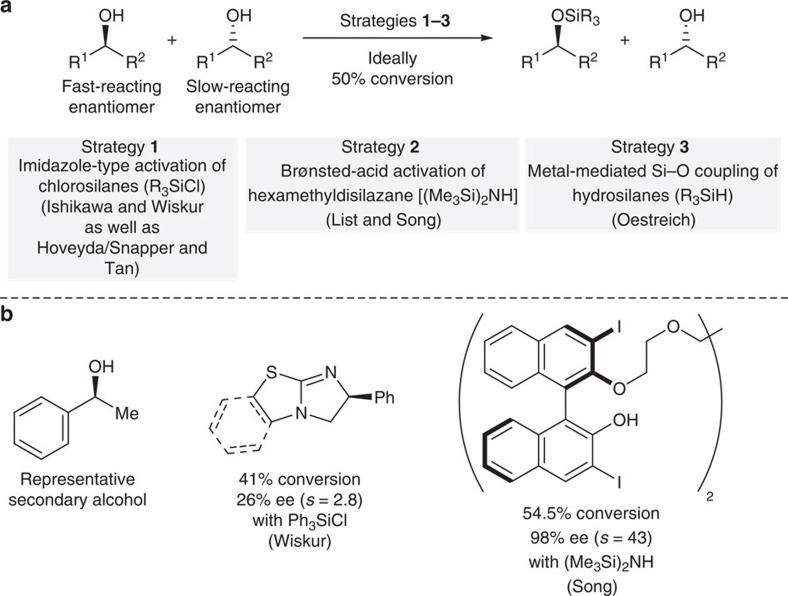
Kinetic resolution of alcohols through silylation. (**a**) General equation and known strategies. (**b**) Representative substrate and reported catalysts. *s*, selectivity factor.

**Figure 3:**
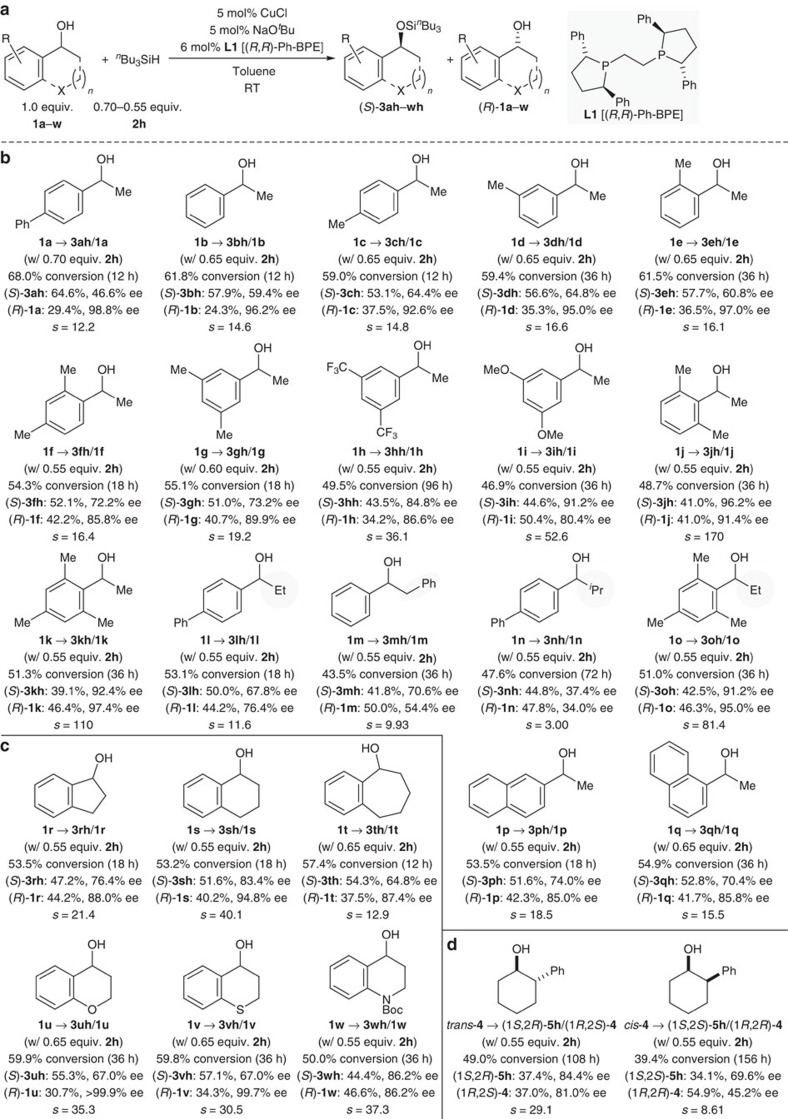
Kinetic resolution of benzylic alcohols and an example of a pair of diastereomeric cyclohexanols. (**a**) General equation. (**b**) Acyclic benzylic alcohols. (**c**) Cyclic benzylic alcohols. (**d**) Diastereomeric 2-phenylcyclohexan-1-ols.

**Figure 4:**
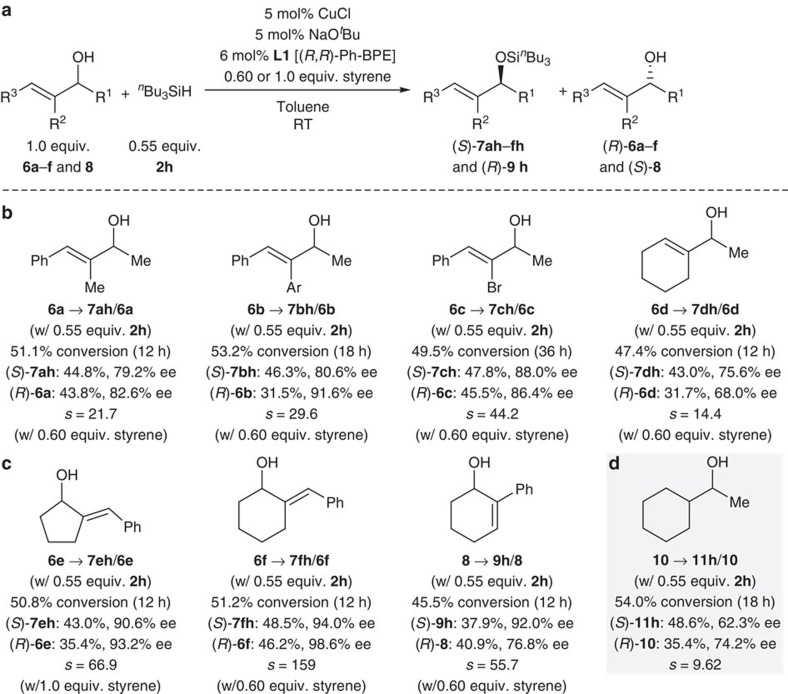
Kinetic resolution of representative allylic alcohols and an example of an aliphatic secondary alcohol. (**a**) General equation. (**b**) Selected allylic alcohols (Ar=4-anisyl). (**c**) Cyclic systems with exo- and endocyclic double bonds. (**d**) 1-Cyclohexylethan-1-ol.

We continued by exploring the substrate scope with this readily accessible catalytic set-up (CuCl/**L1**–NaO^*t*^Bu and ^*n*^Bu_3_SiH, Figs 3 and 4). An analysis of the steric effects in 1-phenylethan-1-ol derivatives showed that monosubstitution of the aryl group in any of the three available positions with methyl groups is not significantly influencing the reaction outcome; selectivity factors for **1b**–**e** were in the same range, just slightly better in the case of the *ortho*-substituted substrate (Fig. 3b). Additional methyl substitution as in **1f** and **1g** generally had little effect but selectivity factors increased substantially with CF_3_ (as in **1h**) or OMe (as in **1i**) instead of the methyl groups. The substituent effect was dramatic for substrates with both *ortho* positions occupied; selectivity factors for **1j** and **1k** were exceedingly high (Fig. 3b). When increasing the size of the alkyl group at the carbinol carbon atom, Me (**1a**/**1b**)<Et (**1l**)<Bn (**1m**)<^*i*^Pr (**1n**), the selectivity factor collapses (12.2/14.6>11.6>9.93>>3.00) while maintaining sufficient reactivity; this effect is also seen for mesityl-substituted **1o** but 81.4 is still an excellent *s* value (highlighted by grey ovals, Fig. 3b). Both β- and α-naphthyl-substituted derivatives **1p** and **1q** fit into the observed selectivity pattern (Fig. 3b). The selectivity factors for the acyclic benzylic alcohols **1a**–**q** were uniformly lower than those obtained with Song's Brønsted-acid catalyst (Strategy 2, Fig. 1)^22^. Conversely, cyclic substrates **1r**–**w** not reported by Song^22^ afforded synthetically valuable selectivity factors independent of the ring size, **1r**–**t**, and with excellent functional-group tolerance, **1u**–**w** (Fig. 3c). Our results compare favourably with those described by Wiskur for the same class of compounds using chlorosilanes (Strategy 1, Fig. 1)^17^.

We also investigated the resolution of isomerically pure 2-phenylcyclohexan-1-ols *trans*-**4** and *cis*-**4** with reasonable success (Fig. 3d). Various types of these compounds had been subject of a dedicated study by Wiskur (Strategy 1, Fig. 1)^21^, and Wiskur found that, from an isomeric mixture of **4**, the isourea-based catalyst preferentially selects the *trans* over the *cis* relative configuration in the kinetic resolution of **4**. Our catalytic system showed the same preference in the individual experiments. Both kinetic resolutions were slow, reaching useful conversion after several days only for *trans*-**4**. The selectivity factor was markedly higher than that achieved by Wiskur^21^ (*s*=29.1 versus *s*=10).

While both acyclic^22^ and cyclic^17^ benzylic alcohols had been resolved by other catalytic methods before, just one example of an acyclic allylic alcohol, (*E*)-1,3-diphenylprop-2-en-1-ol, was described^22^. Allylic alcohols are however ubiquitous synthetic building blocks and, as such, particularly attractive substrates. Without making any changes to our catalytic set-up, allylic alcohols were equally amenable to this enantioselective alcohol silylation but underwent competing partial alkene reduction^38^. This issue was overcome by the substoichiometric addition of a sacrificial, more reactive alkene, styrene^39^,^40^ (Fig. 4a). With this measure, representative allylic alcohols **6a**–**d** were resolved with excellent selectivity factors (Fig. 4b). Cyclic systems with exo- and endocyclic double bonds, **6e** and **6f** as well as **8**, participated in this kinetic resolution with superb efficiency (*s*>55, Fig. 4c). The functional-group tolerance was expanded further by a vinylic bromide (as in **6c**). For comparison, we subjected purely aliphatic alcohol **10** to the standard protocol, and the selectivity factor (*s*=9.62, Fig. 4d) was lower than those obtained for allylic alcohol **6d** (*s*=14.4, Fig. 4b) and benzylic alcohol **1b** (*s*=14.6, Fig. 3b). This indicates that the π system attached to the carbinol carbon atom is important for enantiomer discrimination by the catalyst.

## Discussion

We have disclosed here that the commercially available CuCl/(*R*,*R*)-Ph-BPE–NaO^*t*^Bu catalyst system allows for the kinetic resolution of alcohols by enantioselective Si–O coupling. The choice of the hydrosilane coupling partner is crucial as high selectivity factors have only been achieved with trialkylsilanes, and commercial ^*n*^Bu_3_SiH was used throughout this study. This easy-to-apply catalyst–hydrosilane combination kinetically resolves a broad range of structurally unbiased benzylic and allylic alcohols, reaching synthetically useful selectivity factors for cyclic benzylic (*s*≤40.1, Fig. 3c) and various allylic alcohols (*s*≤159, Fig. 4b,c). It must be emphasized here that the inaccuracy of the analytical tools to measure conversion and enantiomeric purity leads to imprecise selectivity factors, particularly for *s*>50 (refs 27, 41). Hence, the reported values are not exact but rather an approximation of the order of magnitude. While the present protocol is limited to secondary alcohols, we will now tackle the most difficult class of alcohols in this chemistry, tertiary alcohols^28^.

## Methods

### General

Supplementary Figs 1–122 for the HPLC traces, Supplementary Figs 123–256 for the NMR spectra and Supplementary Methods with full experimental details, and the characterization of compounds are given in the Supplementary Information.

### Data availability

The authors declare that the data supporting the findings of this study are available within the article and its Supplementary Information file.

## Additional information

**How to cite this article:** Dong, X. *et al*. Broad-spectrum kinetic resolution of alcohols enabled by Cu–H-catalysed dehydrogenative coupling with hydrosilanes. *Nat. Commun.* **8,** 15547 doi: 10.1038/ncomms15547 (2017).

**Publisher's note**: Springer Nature remains neutral with regard to jurisdictional claims in published maps and institutional affiliations.

## Supplementary Material

Supplementary Information Supplementary figures, supplementary tables, supplementary methods and supplementary references.

## Figures and Tables

**Figure 2:**
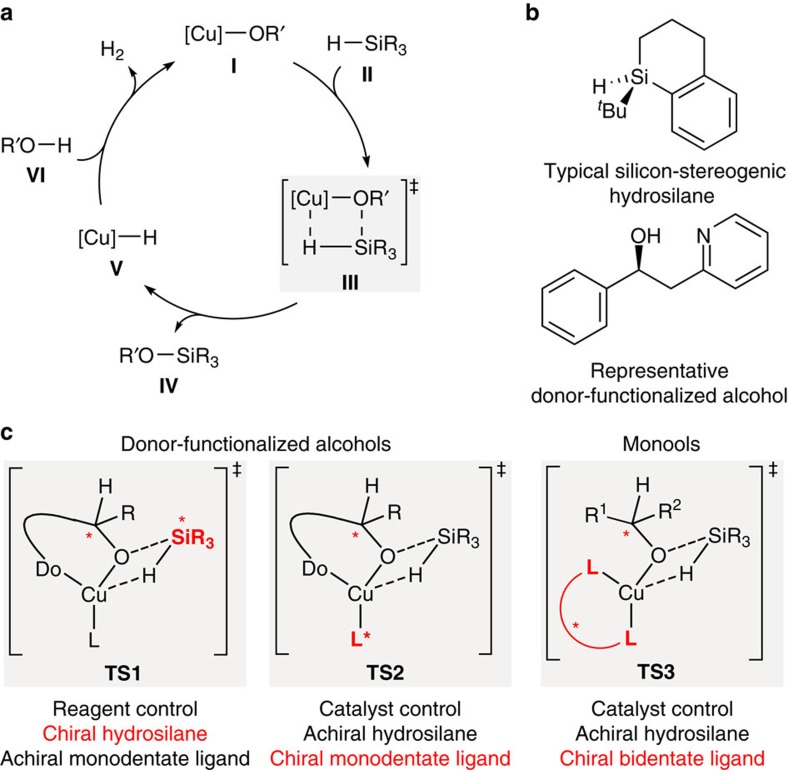
Catalytic asymmetric Si–O coupling for kinetic resolution of alcohols. (**a**) Catalytic cycle of Cu–H catalysis. (**b**) Silicon-stereogenic hydrosilane and typical donor-functionalized substrate. (**c**) Enantioselectivity-determining transition states in the various approaches.

**Table 1:**
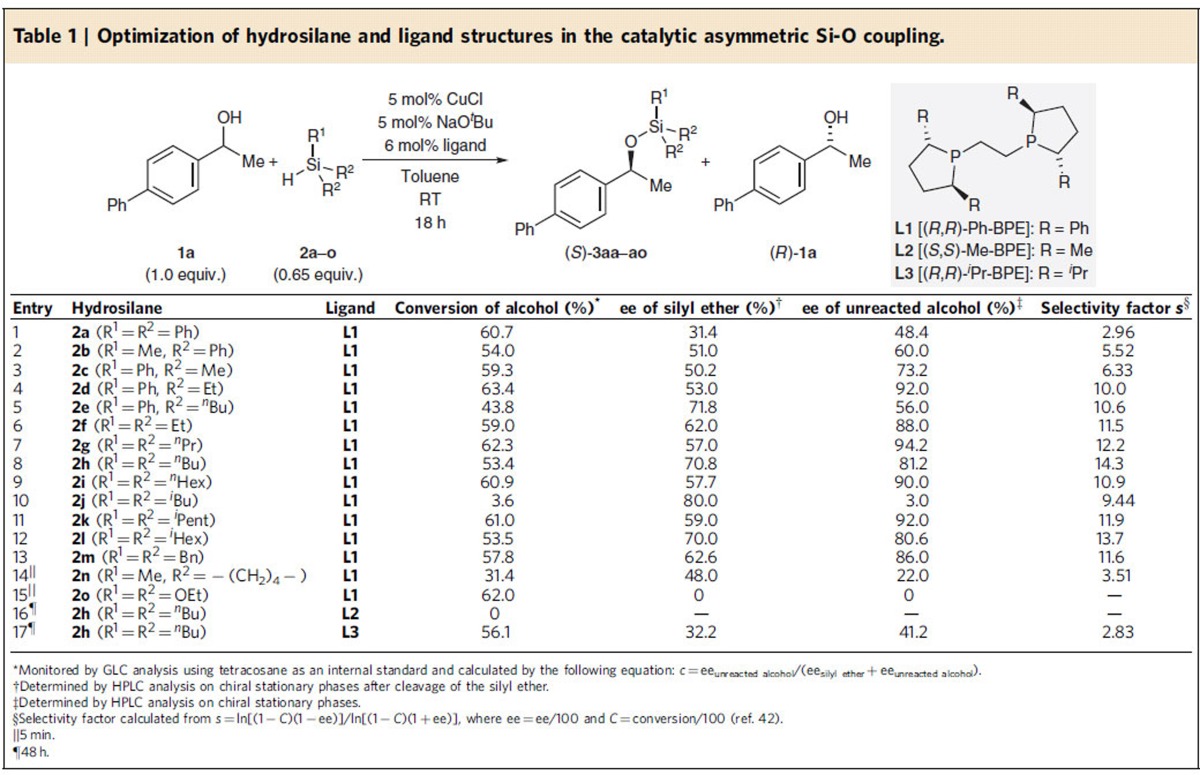
Optimization of hydrosilane and ligand structures in the catalytic asymmetric Si-O coupling.
