# Supplementary material for: Broad-spectrum kinetic resolution of alcohols enabled by Cu–H-catalysed dehydrogenative coupling with hydrosilanes
Source: Nat Commun. 2017 Jun 1;8:15547. doi: 10.1038/ncomms15547 (PMC5461486; doi:10.1038/ncomms15547)
Supplement: Supplementary Information — Supplementary figures, supplementary tables, supplementary methods and supplementary references. [file ncomms15547-s1.pdf]

Supplementary Methods

1 General Information

All reactions were performed in flame-dried glassware using an *MBraun* glove box (O_2 < 0.5 ppm, H_2O < 0.5 ppm) or conventional Schlenk techniques under a static pressure of argon (glove box) or nitrogen. Liquids and solutions were transferred with syringes. Toluene was distilled over sodium, degassed, and stored in glove box over 4 Å molecular sieves. C_6D_6 (purchased from *Aldrich*) and $CDCl_3$ (purchased from *Eurisotop*) were dried over 4 Å molecular sieves; Technical grade solvents for extraction and chromatography (cyclohexane, *n*-pentane, ethyl acetate, *tert*-butyl methyl ether and diethyl ether) were distilled prior to use. (*R*,*R*)-Ph-BPE, (*S*,*S*)-Me-BPE, (*R*,*R*)-^{i}Pr-BPE (**L1–3**, purchased from *abcr* or *Strem*) and ^{n}Bu$_3$SiH (**2h**, purchased from TCI) were used as received. Styrene (purchased from *Aldrich*) was dried over molecular sieves before use. ^{1}H and ^{13}C NMR spectra were recorded in C_6D_6 or $CDCl_3$ on *Bruker* AV 400 and *Bruker* AV 500 instruments. Chemical shifts are reported in parts per million (ppm) downfield from tetramethylsilane and are referenced to the residual solvent resonance as the internal standard (C_6H_6: δ/ppm = 7.16 for ^{1}H NMR and C_6D_6: δ/ppm = 128.06 for ^{13}C NMR, $CHCl_3$: δ/ppm = 7.26 for ^{1}H NMR and $CDCl_3$: δ/ppm = 77.16 for ^{13}C NMR). ^{19}F NMR spectrum was calibrated according to the IUPAC recommendation using a unified chemical shift scale based on the proton resonance of trimethylsilane as primary reference. Data are reported as follows: chemical shift, multiplicity (br s = broad singlet, s = singlet, d = doublet, t = triplet, q = quartet, m = multiplet), coupling constant (Hz), and integration. Gas liquid chromatography (GLC) was performed on an *Agilent Technologies* 7820A gas chromatograph equipped with a SE-54 capillary column (30 m × 0.32 mm, 0.25 µm film thickness) by *CS-Chromatography Service* using the following programs: N_2 carrier gas, column flow 1.7 mL/min, injection temperature 280 °C, detector temperature 300 °C; temperature program: start tempe-rature 40 °C, heating rate 10 °C/min, final temperature 280 °C for 10 min. Melting points (m.p.) were determined with a Stuart Scientific SMP20 melting point apparatus and are not corrected. High resolution mass spectrometry [HRMS, Atmospheric-Pressure Chemical Ionization (APCI) and Electronic Impact (EI)] were performed by the analytical facility at the Institut für Chemie, Technische Universität Berlin. Analytical thin-layer chromatography (TLC) was performed on silica gel 60 F254 glass plates from Merck. Flash-column chromatography was performed on silica gel 60 (40–63 µm, 230–400 mesh, ASTM) by *Merck* using the indicated solvents. Infrared (IR) spectra were recorded on an *Agilent Technologies* Cary 630 FT-IR spectrophotometer equipped with an ATR unit and are reported in wavenumbers (cm^{-1}). Optical rotations were measured on a *Schmidt & Haensch* Polatronic H532 polarimeter with $[\alpha]_\lambda$ values reported in 10^{-1} (cm^2 g^{-1}); concentration *c* is in g/100 mL and λ as indicated. Enantiomeric excesses were determined by analytical high performance liquid chromatography (HPLC) analysis on an *Agilent Technologies* 1290 Infinity instrument with a chiral stationary phase using a *Daicel* Chiralcel OD-H column, or a *Daicel* Chiralcel AD-H column (*n*-heptane/isopropanol mixtures as solvent).

2 General Procedures (GPs)

2.1 GP1: General Procedure for the Kinetic Resolution of Alcohols (1a–w, *trans*-4 and *cis*-4)

In an argon-filled glove box, a 1.5-mL GC vial containing a magnetic stir bar was charged with CuCl (2.00 mg, 20.0 µmol, 5.00 mol%), NaO^{t}Bu (2.00 mg, 20.0 µmol, 5.00 mol%), and (*R*,*R*)-Ph-BPE (**L1**, 12.5 mg, 24.0 µmol, 6.00 mol%), 0.200 mL toluene was added via syringe and then stirred at room temperature for 10 min to give a pale yellow solution. ^{n}Bu$_3$SiH (**2h**, 254.0 mg, 0.260 mmol, 0.650 equiv..) was introduced, resulting a yellowish solution after stirring at room temperature for another 10 min. A solution of the indicated alcohol (0.400 mmol) in toluene (1.00 mL) was added. The reaction was maintained at ambient temperature and monitored by GLC until a satisfying conversion was reached. Direct purification by column chromatography on silica gel using *n*-pentane/diethyl ether or cyclohexane/ethyl acetate as eluent afforded the analytically pure silyl ethers and the unreacted enantioenriched alcohols.

2.2 GP2: General Procedure for the Kinetic Resolution of Allylic Alcohols (6a–f and 8)

In an argon-filled glove box, a 1.5-mL GC vial containing a magnetic stir bar was charged with CuCl (2.00 mg, 20.0 µmol, 5.00 mol%), NaO^{t}Bu (2.00 mg, 20.0 µmol, 5.00 mol%), and (*R*,*R*)-Ph-BPE (12.5 mg, 24.0 µmol, 6.00 mol%), 0.200 mL toluene was added via syringe and then stirred at room temperature for 10 min to give a pale yellow solution. ^{n}Bu$_3$SiH (**2h**, 46.0 mg, 0.220 mmol, 0.550 equiv.) was introduced, resulting a yellowish solution after stirring at room temperature for another 10 min. A solution of the indicated alcohol (0.400 mmol) and the styrene (25.0 mg, 0.240 mmol, 0.600 equiv.) in toluene (1.00 mL) was added. The reaction was maintained at ambient temperature and monitored by GLC until a satisfying conversion was reached. Purification by column chromatography on silica gel using pentane/diethyl ether or cyclohexane/ethyl acetate as eluent afforded the analytically pure silyl ethers and the unreacted enantioenriched allylic alcohols.

2.3 GP3: General Procedure for the Preparation of the Alcohols (1a, 1d–e, 1h, 1m, 1p, 1r, 1t–w and *cis*-4)[1]

To a 25-mL flask with a stir bar was added the indicated ketone (5.0 mmol, 1.0 equiv.) and methanol to a concentration of 0.50 M. The resulting mixture was cooled to 0 °C and NaBH$_4$ (0.50–1.0 equiv.) was added in portions. The ice bath was allowed to warm to room temperature, and the reaction was stirred overnight. After full conversion of the ketone as monitored by TLC or GLC analysis, the reaction mixture was quenched by the addition of brine (10 mL) and extracted with diethyl ether (3 × 20 mL). The organic layers were combined and dried over anhydrous MgSO$_4$. The solvent was removed under reduced pressure.

Purification of the residue by flash chromatography on silica gel afforded the racemic alcohols.

2.4 GP4: General Procedure for the Preparation of the Alcohols (1g, 1i–l, 1n, 1o)[2]

Magnesium turnings (292mg, 13.0 mmol, 1.30 equiv.), some iodine crystals and dry tetrahydrofuran (THF, 20.0 mL) was added to a 100-mL two necked flask equipped with a condenser. The resulting mixture was stirred at room temperature for 10min. A solution of indicated bromoarene (10.0 mmol, 1.00 equiv.) in 5.00 mL THF was added dropwise. As soon as the reaction has started (initiate with a heat gun if necessary), the remainder of the tetrahydrofuran solution is added at such a rate that a gentle reflux is maintained. After complete addition, reflux is continued for 1 hour by heating with an oil bath, and then cooled to 0 °C.

The indicated aldehyde (15.0 mmol, 1.50 equiv.) was added to the indicated Grignard reagent dropwise over 5 min at 0 °C. The mixture was allowed to warm to room temperature and stirred at reflux for 1-3 hours. After cooled to room temperature, the reaction was quenched with 20.0 mL 1 N hydrochloric acid. The mixture was extracted with dichloromethane (3 × 30 mL). The combined organic layer was washed with brine (40.0 mL) and dried over anhydrous $MgSO_4$. The volatiles removed under vacuum. Purification of the residue by flash chromatography on silica gel afforded the racemic alcohols.

2.5 GP5: General Procedure for the Preparation of the Allylic Alcohols (6a–f and 8)[3]

The corresponding *α*,*β*-unsaturated ketone (5.00 mmol, 1.00 equiv.) and $CeCl_3 \cdot 7H_2O$ (1.86 g, 5.00 mmol, 1.00 equiv.) were dissolved in 12.5 mL methanol to a concentration of 0.400 M. $NaBH_4$ (189 mg, 5.00 mmol, 1.00 equiv.) was added one portion with stirring. The reaction was stirred for another 5 min and quenched by the addition of brine (10.0 mL). The mixture was extracted with diethyl ether (3 × 20 mL). The organic layers were combined, dried over $MgSO_4$, and the volatiles removed under vacuum. The residue was then purified by silica gel column chromatography with *n*-pentane/diethyl ether or cyclohexane/ethyl acetate as eluent.

2.6 GP6: General Procedure for Deprotection of Silylated Alcohols[1]

The indicated silyl ether (5.0-10 mg) was dissolved in 0.50 mL THF in a 2-dram vial, and tetra-*n*-butylammonium fluoride (TBAF, 0.5 mL, 1M in THF) was added subsequently. The resulting mixture was stirred at room temperature. The reaction was monitored by TLC or GLC analysis. Upon completion, the reaction was then quenched with brine (5.0 mL), and extracted with diethyl ether (3 × 10 mL). The organic layers were combined, concentrated under vacuum, and the crude material was purified by preparative TLC. The isolated, deprotected alcohol was then analyzed by HPLC.

3 Hydrosilane and Alcohol Reagents

Triphenylsilane (**2a**, Aldrich), methyldiphenylsilane (**2b**, TCI), dimethyl(phenyl)silane (**2c**, ABCR), Triethylsilane (**2f**, Aldrich), tripropylsilane (**2g**, Acros), tributylsilane (**2h**, TCI), trihexylsilane (**2i**, ABCR), triethoxysilane (**2o**, Alfa Aesar), diethoxy(methyl)silane (ABCR), 1,1,1,3,5,5,5-heptamethyltrisiloxane (ABCR), triisopropylsilane (ABCR) are commercial available. All the dimethylarenesilane and methyldiarenesilane are synthesized according to a method reported by our group.[4] The synthesis of other silanes will be reported in due course.

rac-**1a**: *rac*-1-([1,1'-Biphenyl]-4-yl)ethan-1-ol was prepared from 1-([1,1'-biphenyl]-4-yl)ethan-1-one (purchased from Sigma-Aldrich) according to GP3.

rac-**1b**: *rac*-1-Phenylethan-1-ol was purchased from Sigma-Aldrich.

rac-**1c**: *rac*-1-(*p*-Tolyl)ethan-1-ol was purchased from TCI.

rac-**1d**: *rac*-1-(*m*-Tolyl)ethan-1-ol was prepared from 1-(*m*-tolyl)ethan-1-one (purchased from Acros) according to GP3.

rac-**1e**: *rac*-1-(*o*-Tolyl)ethan-1-ol was prepared from 1-(*o*-tolyl)ethan-1-one (purchased from ABCR) according to GP3.

rac-**1f**: *rac*-1-(2,4-Dimethylphenyl)ethan-1-ol was prepared from 1-(2,4-dimethyl -phenyl)ethan-1-one (purchased from Sigma-Aldrich) according to GP3.

rac-**1g**: *rac*-1-(3,5-Dimethylphenyl)ethan-1-ol was prepared from 1-bromo-3,5-dimethylbenzene (purchased from ABCR) and acetaldehyde (purchased from Fluka) according to GP4.

rac-**1h**: *rac*-1-(3,5-Bis(trifluoromethyl)phenyl)ethan-1-ol was prepared from 1-(3,5-bis(trifluoromethyl) phenyl)ethan-1-one (purchased from Sigma-Aldrich) according to GP3.

rac-**1i**: *rac*-1-(3,5-Dimethoxyphenyl)ethan-1-ol was prepared from 1-bromo-3,5-dimethoxybenzene (purchased from TCI) and acetaldehyde (purchased from Fluka) according to GP4.

rac-**1j**: *rac*-1-(2,6-Dimethylphenyl)ethan-1-ol was prepared from 2-bromo-1,3-dimethylbenzene (purchased from ABCR) and acetaldehyde (purchased from Fluka) according to GP4.

rac-**1k**: *rac*-1-Mesitylethan-1-ol was prepared from 2-bromo-1,3,5-trimethylbenzene (purchased from TCI) and acetaldehyde (purchased from Fluka) according to GP4.

rac-**1l**: *rac*-1-([1,1'-Biphenyl]-4-yl)propan-1-ol was prepared from [1,1'-biphenyl]-

4-carbaldehyde (purchased from Acros) and ethylmagnesium bromide (3.0 mol/L in Et_2O, purchased from Acros) according to modified GP4.

rac-**1m**: *rac*-1,2-Diphenylethan-1-ol was prepared from 1,2-diphenylethan-1-one (purchased from Sigma-Aldrich) according to GP3.

rac-**1n**: *rac*-1-([1,1'-Biphenyl]-4-yl)-2-methylpropan-1-ol was prepared from [1,1'-biphenyl]-4-carbaldehyde (purchased from Acros) and isopropylmagnesium bromide (1.0 mol/L in THF, purchased from Sigma-Aldrich) according to modified GP4.

rac-**1o**: *rac*-1-Mesitylpropan-1-ol was prepared from 2-bromo-1,3,5-trimethylbenzene (purchased from TCI) and propionaldehyde (purchased from Aldrich) according to GP4.

rac-**1p**: *rac*-1-(Naphthalen-2-yl)ethan-1-ol was prepared from 1-(naphthalen-2-yl)ethan-1-one purchased from Sigma-Aldrich) according to GP3.

rac-**1q**: *rac*-1-(Naphthalen-1-yl)ethan-1-ol was purchased from Aldrich.

rac-**1r**: *rac*-2,3-Dihydro-1*H*-inden-1-ol was prepared from 2,3-dihydro-1*H*-inden-1-one (purchased from Fluka) according to GP3.

rac-**1s**: *rac*-1,2,3,4-tetrahydronaphthalen-1-ol was purchased from Aldrich.

rac-**1t**: *rac*-6,7,8,9-tetrahydro-5*H*-benzo[7]annulen-5-ol was prepared from 6,7,8,9-tetrahydro-5*H*-benzo[7]annulen-5-one (purchased from TCI) according to GP3.

rac-**1u**: *rac*-Chroman-4-ol was prepared from chroman-4-one (purchased from ABCR) according to GP3.

rac-**1v**: *rac*-Thiochroman-4-ol was prepared from thiochroman-4-one (purchased from Acros) according to GP3.

rac-**1w**: *rac*-*tert*-Butyl 4-hydroxy-3,4-dihydroquinoline-1(2*H*)-carboxylate was prepared from *tert*-butyl 4-oxo-3,4-dihydroquinoline-1(2*H*)-carboxylate (prepared from known procedure[5]) according to GP3.

rac-*trans*-**4**: *rac*-*trans*-2-Phenylcyclohexan-1-ol was purchased from TCI.

rac-*cis*-**4**: *rac*-*cis*-2-Phenylcyclohexan-1-ol was prepared from *rac*-2-phenylcyclohexan-1-one (purchased from TCI) according to GP3.

rac-**6a**: *rac*-(*E*)-3-Methyl-4-phenylbut-3-en-2-ol prepared from (*E*)-3-methyl-4-phenylbut-3-en-2-one (prepared from known procedure[6]) according to GP5.

rac-**6b**: *rac*-(*E*)-3-(4-Methoxyphenyl)-4-phenylbut-3-en-2-ol prepared from (*E*)-3-(4-

methoxyphenyl)-4-phenylbut-3-en-2-one (prepared from known procedure[6]) according to GP5.

rac-**6c**: *rac*-(*Z*)-3-Bromo-4-phenylbut-3-en-2-ol prepared from (*Z*)-3-bromo-4-phenylbut-3-en-2-one (prepared from known procedure[7]) according to GP5.

rac-**6d**: *rac*-1-(Cyclohex-1-en-1-yl)ethan-1-one prepared from 1-(cyclohex-1-en-1-yl)ethan-1-ol (purchased from TCI) according to GP5.

rac-**6e**: *rac*-(*E*)-2-Benzylidenecyclopentan-1-ol prepared from (*E*)-2-benzylidenecyclopentan-1-one (prepared from known procedure[6]) according to GP5.

rac-**6f**: *rac*-(*E*)-2-Benzylidenecyclohexan-1-ol prepared from (*E*)-2-benzylidenecyclohexan-1-one (prepared from known procedure[6]) according to GP5.

rac-**8**: *rac*-2,3,4,5-Tetrahydro-[1,1'-biphenyl]-2-ol prepared from 4,5-dihydro-[1,1'-biphenyl]-2(3*H*)-one (prepared from known procedure[8]) according to GP5.

rac-**10**: *rac*-1-cyclohexylethan-1-ol was purchased from Merck.

4 Optimization Tables

Otherwise noted, conversion was monitored by GLC analysis using tetracosane as an internal standard and calculated by the following equation: $c = ee_{\text{unreacted alcohol}}/(ee_{\text{silyl ether}} + ee_{\text{unreacted alcohol}})$. The ee of the recovered alcohol was determined by HPLC analysis on chiral stationary phase. The ee of silyl ether product was determined by HPLC analysis on chiral stationary phase after the cleavage of the silyl ether. In all the cases, selectivity factor was calculated form $s = \ln[(1–C)(1–ee_{\text{unreacted alcohol}})]/\ln[(1–C)(1+ee_{\text{unreacted alcohol}})]$ where ee = ee/100 and C = conversion/100.

Supplementary Table 1. Screening of ligand

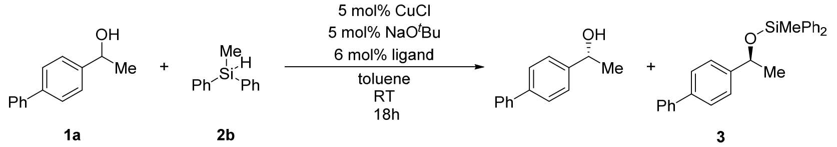

1a (1.0 equiv.) **2b** (0.65 equiv.) **3**

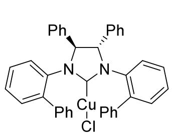

C1
28% conversion[a]
1a: 3% ee
s = 1.20 (72h)

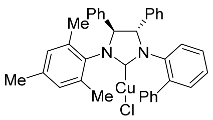

C2
0% conversion[a]
72h

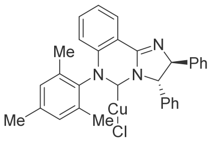

C3
30% conversion[a]
1a: 6% ee
s = 1.40

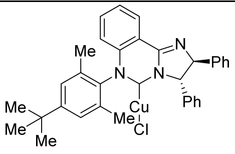

C4
25% conversion[a]
1a: 6% ee
s = 1.52

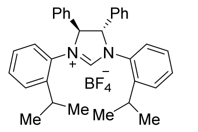

L4
19% conversion[a]
1a: 4% ee
s = 1.46

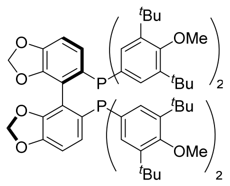

L5
46% conversion[b]
(*S*)-**1b:** 22% ee
s = 2.22 @ 55°C

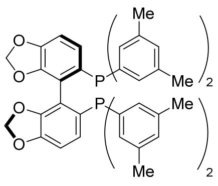

L6
55% conversion[a,b]
(*S*)-**1b:** 17% ee
s = 1.53

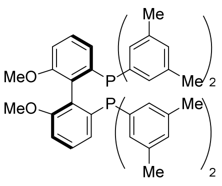

L7
55% conversion[a,b]
1b: 3% ee
s = 1.03

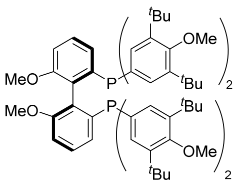

L8
57% conversion[a,b]
1b: 0% ee
@ 55°C

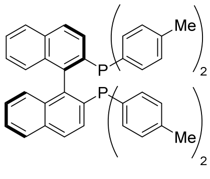

L9
0% conversion[a,b]

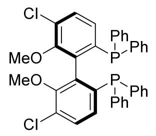

L10[b]
0% conversion[a,b]

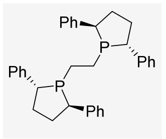

L1
54% conversion
(*S*)-**3ab,** 51% ee
(*R*)-**1a**: 60% ee
s = 5.52

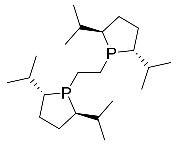

L2
56% conversion[d]
(*S*)-**3ab,** 32% ee
(*R*)-**1a**: 41% ee
s = 2.83

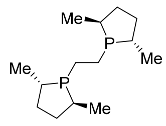

L3
0% conversion[a,d]

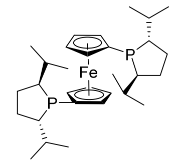

L11
63% conversion[a,c]
1a: 0% ee

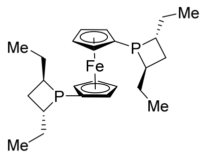

L12
62% conversion[a,c]
(*R*)-**3ab,** 5% ee
s = 1.10

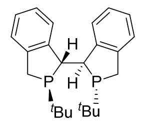

L13
13% conversion
(*R*)-**3bb:** 54% ee
(*S*)-**1b**: 7% ee
s = 2.98

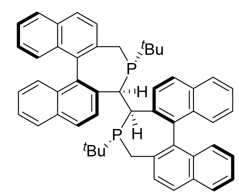

L14
60% conversion
(*R*)-**3bb:** 20% ee
(*S*)-**1b**: 30% ee
s = 1.94

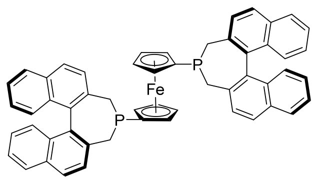

L15
55% conversion[a,c]
(*S*)-**1a,** 13% ee
s = 1.38

[a] Conversion was determined by GLC analysis using tetracosane as an internal standard. [b] *rac*-1-phenylethan-1-ol (**1b**) was used as model substrate. [c] dimethylphenylsilane (**2c**) was used as resolving reagent. [d] ^{n}Bu$_3$SiH (**2h**) was used as resolving reagent.

Supplementary Table 2. Screening of metal source

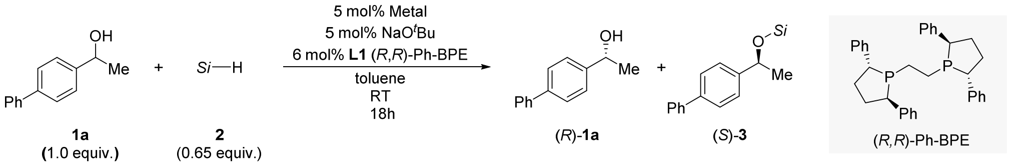

Entry	Metal Source	Conversion of *rac*-**1a** [%]	ee of unreacted alchol [%]	ee of silyl ether [%]	Selectivity factors s	Hydrosilane
1	CuCl	59	73	50	6.33	$PhMe_2SiH$
2	$(CF_3SO_3Cu)_2{\cdot}C_6H_6$	59	76	52	6.92	$PhMe_2SiH$
3	$(MeCN)_4CuBF_4$	62	77	48	6.14	$PhMe_2SiH$
4	CuCN	64	77	44	5.64	$PhMe_2SiH$
1°	CuCl	59	88	62	11.5	Et_3SiH
5	$Cu(OAc)_2$	0	--	--	--	Et_3SiH
6[a]	Mesitylcopper(I)	61	92	58	11.9	Et_3SiH
7[b]	$[Rh(COD)Cl]_2$	26				Et_3SiH
8[b]	$[Ir(COD)Cl]_2$	9				Et_3SiH

[a] No base was used. [b] Measured by GLC using tetracosane as an internal standard.

Supplementary Table 3. Screening of base

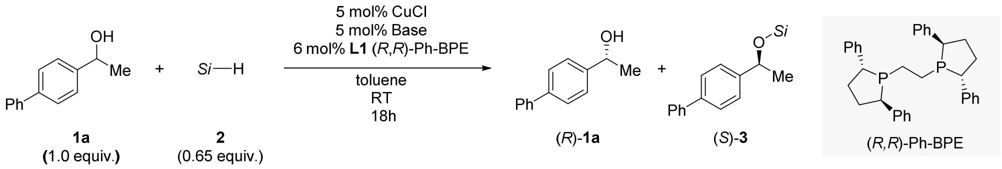

Entry	Base	Conversion of *rac*-**1a** [%]	ee of unreacted alchol [%]	ee of silyl ether [%]	Selectivity factors s	Hydrosilane
1	NaO^tBu	59	73	50	6.33	$PhMe_2SiH$
9[a]	NaOMe	61	75	51	6.09	$PhMe_2SiH$
10	Cs_2CO_3	60	71	47	5.67	$PhMe_2SiH$
1°	NaO^tBu	59	88	62	11.5	Et_3SiH
11	KO^tBu	57	82	62	10.4	Et_3SiH
12	LiO^tBu	37	44	76	10.6	Et_3SiH
13	CsF	60	83	56	8.61	Et_3SiH

[a] 4 days.

Supplementary Table 4. Screening of solvent

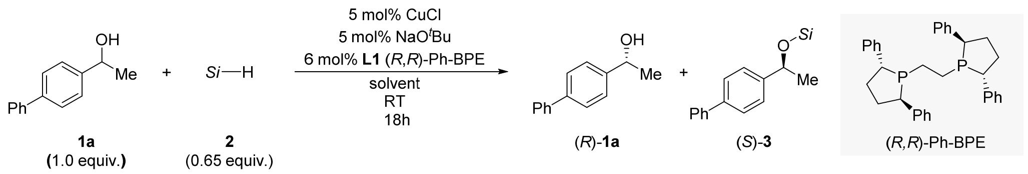

Entry	Solvent	Conversion of **1a** [%]	ee of unreacted alchol [%]	ee of silyl ether [%]	Selectivity factors s	Hydro silane
1	Toluene	59	73	50	6.33	$PhMe_2SiH$
14	DCM	65	42	23	2.28	$PhMe_2SiH$
15	Benzene	64	81	46	6.26	$PhMe_2SiH$
16	Chlorobenzene	51	43	41	3.56	$PhMe_2SiH$
17	1,2-Dichloro benzene	50	59	59	6.90	$PhMe_2SiH$
1[o]	Toluene	59	88	62	11.5	Et_3SiH
18	THF	29	32	80	11.2	Et_3SiH
19[a]	Fluorobenzene	60	89	57	10.3	Et_3SiH
20[a]	1,2-Difluoro benzene	60	71	48	5.67	Et_3SiH
21[a]	Et_2O	42	55	76	12.6	Et_3SiH
22[a]	1,4-Dioxane	24	26	81	13.2	Et_3SiH
23[a]	MeCN		29			Et_3SiH
24[a]	DMF	39	48	76	11.2	Et_3SiH
25[a]	DMSO	38	42	70	8.02	Et_3SiH

[e] Mesitylcopper(I) was used instead of CuCl/NaO^{t}Bu

Supplementary Table 5. Collections of tested hydrosilanes

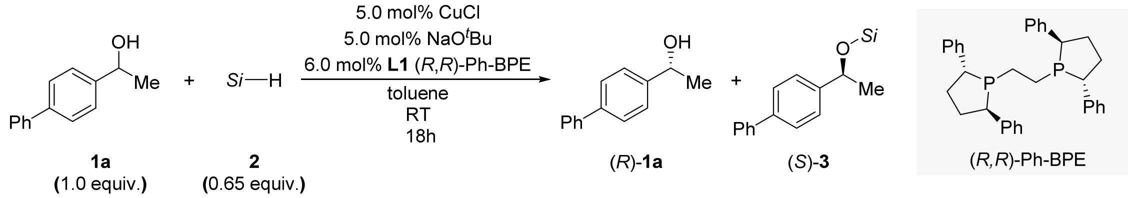

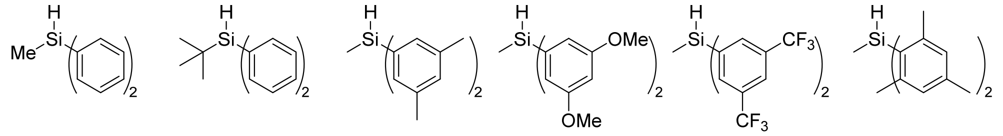

**S1** (**2b**)	**S2**	**S3**	**S4**	**S5**	**S6**
54.0% conversion,	38.0% conversion[a],	62.7% conversion,	68.4% conversion,	undefined mixture	0% conversion[a]
(*R*)-**1a**: 60.0% ee	(*R*)-**1a**: 13.0% ee	(*R*)-**1a**: 71.0% ee	(*R*)-**1a**: 67.0% ee		@ 110°C, 24h
(*S*)-**3ab**: 51% ee	***s* = 1.73**	(*S*)-SiO: 42.2% ee	(*S*)-SiO: 31.0% ee		
***s* = 5.52**		***s* = 4.95**	***s* = 3.53**		

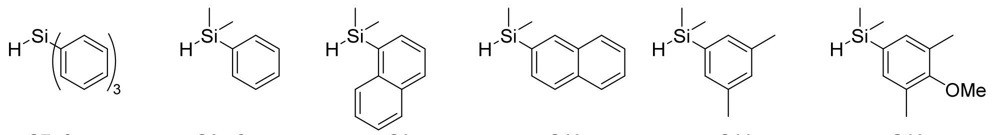

**S7** (**2a**)	**S8** (**2c**)	**S9**	**S10**	**S11**	**S12**
60.7% conversion,	59.3% conversion,	62.7% conversion,	60.4% conversion,	60.5% conversion,	58.3% conversion,
(*R*)-**1a**: 48.4% ee	(*R*)-**1a**: 73.2% ee	(*R*)-**1a**: 75.0% ee	(*R*)-**1a**: 71.0% ee	(*R*)-**1a**: 75.0% ee	(*R*)-**1a**: 70.0% ee
(*S*)-**3aa**: 31.4% ee	(*S*)-**3ac** 50.2% ee	(*S*)-SiO: 44.6% ee	(*S*)-SiO: 46.6% ee	(*S*)-SiO: 49.0% ee	(*S*)-SiO: 50.0% ee
***s* = 2.96**	***s* = 6.33**	***s* = 5.56**	***s* = 5.55**	***s* = 6.26**	***s* = 6.04**

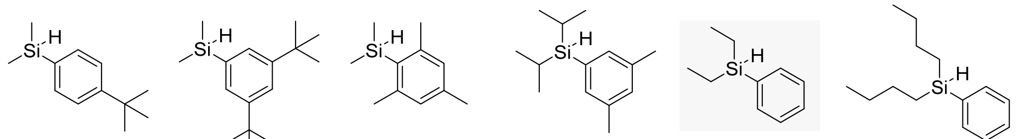

**S13**	**S14**	**S15**	**S16**	**S17** (**2d**)	**S18** (**2e**)
64.7% conversion,	59.7% conversion,	65.0% conversion[a]	51.2% conversion,	63.4% conversion,	43.8% conversion,
(*R*)-**1a**: 81.0% ee	(*R*)-**1a**: 63.0% ee	(*R*)-**1a**: 71.0% ee	(*R*)-**1a**: 51.0% ee	(*R*)-**1a**: 92.0% ee	(*R*)-**1a**: 56.0% ee
(*S*)-SiO: 44.2% ee	(*S*)-SiO: 42.6% ee	***s* = 4.45**	(*S*)-SiO: 48.6% ee	(*S*)-**3ad**: 53.0% ee	(*S*)-**3ae**: 71.8% ee
***s* = 6.06**	***s* = 4.52**		***s* = 4.68** @ 84h	***s* = 10.0** @ 24h	***s* = 10.6**

Supplementary Table 5 (continued).

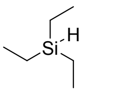

S19 (**2f**)
58.7% conversion,
(*R*)-**1a**: 88.0% ee
(*S*)-**3af**: 62.0% ee
***s* = 11.8**

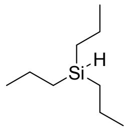

S20 (**2g**)
62.3 % conversion,
(*R*)-**1a**: 94.2% ee
(*S*)-**3ag**: 57.0% ee
***s* = 12.2**

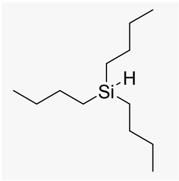

S21 (**2h**)
53.4% conversion,
(*R*)-**1a**: 81.2% ee
(*S*)-**3ah**: 70.8% ee
***s* = 14.3**

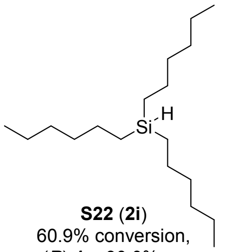

S22 (**2i**)
60.9% conversion,
(*R*)-**1a**: 90.0% ee
(*S*)-**3ai**: 57.7% ee
***s* = 10.9**

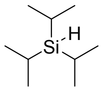

S23
0% conversion[a]
72h @ rt

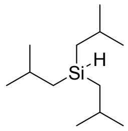

S24 (**2j**)
3.6% conversion,
(*R*)-**1a**: 3.0% ee
(*S*)-**3aj**: 80.0% ee
***s* = 9.44**

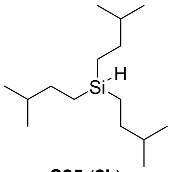

S25 (**2k**)
60.9% conversion,
(*R*)-**1a**: 92.0% ee
(*S*)-**3ak**: 59.0% ee
***s* = 12.0**

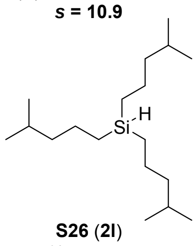

S26 (**2l**)
53.5% conversion,
(*R*)-**1a**: 80.6% ee
(*S*)-**3al**: 70.0% ee
***s* = 13.7**

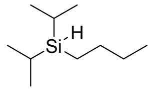

S27
57.9% conversion,
(*R*)-**1a**: 82.6% ee
(*S*)-SiO: 60.0% ee
***s* = 9.93 @** 5d

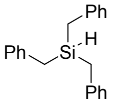

S28 (**2m**)
57.8% conversion,
(*R*)-**1a**: 86% ee
(*S*)-**3am**: 62.6% ee
***s* = 11.6**

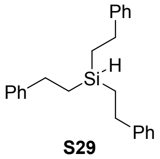

S29
56.0% conversion,
(*R*)-**1a**: 84.0% ee
(*S*)-SiO: 64.0% ee
***s* = 12.5**

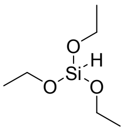

S30 (**2o**)
62.0% conversion[a],
1a: 0% ee
3ao: 0% ee
5min

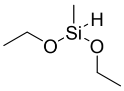

S31
63.0% conversion[a]
1a: 0% ee
SiO: 0% ee

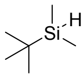

S32
54.8% conversion,
(*R*)-**1a**: 71.0% ee
(*S*)-SiO: 58.5% ee
***s* = 7.88**

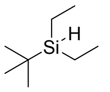

S33
10.6 % conversion,
(*R*)-**1a**: 10.2 % ee
(*S*)-SiO: 86.0% ee
***s* = 14.7 @** 3d

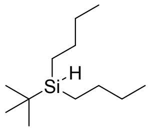

S34
<3% conversion[a]
3d

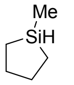

S35 (**2n**)
31.4% conversion,
(*R*)-**1a**: 22.0% ee
(*S*)-**3an**: 48.0% ee
***s* = 3.51**
5min

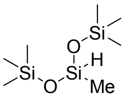

S36
63.0% conversion[a],
(*R*)-**1a**: 56% ee
***s* = 3.30**

[a]Measured by GLC using tetracosane as an internal standard. [b] SiO = the corresponding silyl ether product.

5 Procedures and Characterization Data for Alcohols (1a–w, *trans*-4/*cis*-4, 6a–f, 8 and 10) and Silyl Ethers (3ah–wh, *trans*-5h/*cis*-5h, 7ah–fh, 9h and 11h)

(*R*)-1-([1,1'-Biphenyl]-4-yl)ethan-1-ol [(*R*)-1a]

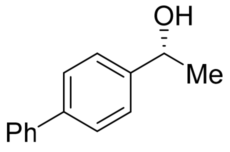

(*R*)-**1a**

$C_{14}H_{14}O$

M = 198.27 g/mol

1st run: Prepared according to GP1 from *rac*-1-([1,1'-biphenyl]-4-yl)ethan-1-ol (*rac*-**1a**, 40.0 mg, 200 µmol, 1.00 equiv.), $^{n}Bu_3SiH$ (**2h**, 26.0 mg, 130 µmol, 0.650 equiv.). Reaction was stopped after 12 h, and the crude product was purified by flash-column chromatography using cyclohexane/ethyl acetate (5:1) as eluent. The title compound (*R*)-**1a** was isolated as a white solid (16.1 mg, 40.3% yield, 81.2% ee).

2nd run: Prepared according to GP1 from *rac*-1-([1,1'-biphenyl]-4-yl)ethan-1-ol (*rac*-**1a**, 80.0 mg, 400 µmol, 1.00 equiv.), $^{n}Bu_3SiH$ (56.0 mg, 280 µmol, 0.700 equiv.). Reaction was stopped after 12 h, and the crude product was purified by flash-column chromatography using *n*-pentane/ethyl acetate (5:1) as eluent. The title compound (*R*)-**1a** was isolated as a white solid (23.5 mg, 29.4% yield, 98.8% ee).

M.p.: 98–99°C. $\mathbf{R_f}$ = 0.23 (*n*-pentane:ethyl acetate 5:1). ^{1}**H NMR** (400 MHz, C_6D_6): δ/ppm = 7.51–7.45 (m, 4H), 7.28–7.21 (m, 4H), 7.15–7.12 (m, 1H), 4.58 (q, *J* = 6.5 Hz, 1H), 1.42 (s_{br}, 1H), 1.33 (d, *J* = 6.5 Hz, 3H). ^{13}**C NMR** (126 MHz, $CDCl_3$): δ/ppm = 145.0, 141.0, 140.6, 128.9, 127.4, 127.2, 126.0, 70.3, 25.3. **HRMS** (APCI) for $C_{14}H_{13}O^+$ $[M–H]^+$ calcd *m/z* 197.0961 found 197.0959. **IR** (ATR): $\tilde{\nu}$/cm^{-1} = 3296, 2970, 2655, 2099, 1563, 1400, 1340, 1067, 1003, 833, 758, 725, 685. **Optical rotation**: $[\alpha]_D^{20}$ = +50.2 (*c* 0.89, $CHCl_3$, 98.8% ee); $[\alpha]_D^{25}$ = –30 (*c* 0.94, $CHCl_3$, 70% ee) reported for (*S*)-enantiomer.[9] The enantiomeric excess of (*R*)-**1a** was determined by HPLC analysis on a chiral stationary phase (Daicel Chiralcel AD-H column, column temperature 20°C, solvent *n*-heptane:isopropanol = 95:5, flow rate 0.8 mL/min, λ = 254 nm): t_R = 16.3 min for (*S*)-**1a**, t_R = 17.8 min for (*R*)-**1a**.

The analytical data are in accordance with those reported.[9]

(*S*)-(1-([1,1'-Biphenyl]-4-yl)ethoxy)tributylsilane [(*S*)-3ah]

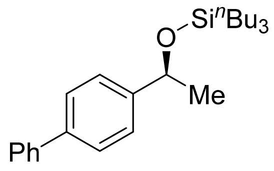

(*S*)-**3ah**

$C_{26}H_{40}OSi$

M = 396.69 g/mol

1st run: Prepared according to GP1 from *rac*-1-([1,1'-biphenyl]-4-yl)ethan-1-ol (*rac*-**1a**, 40.0 mg, 200 µmol, 1.00 equiv.), $^{n}Bu_3SiH$ (26.0 mg, 130 µmol, 0.650 equiv.). Reaction was stopped after 12 h, and the crude product was purified by flash-column chromatography using cyclohexane as eluent. The title compound (*R*)-**3ah** was isolated as a colorless liquid (37.5 mg, 46.7% yield, 70.8% ee).

2nd run: Prepared according to GP1 from *rac*-1-([1,1'-biphenyl]-4-yl)ethan-1-ol (*rac*-**1a**, 80.0 mg, 400 µmol, 1.00 equiv.), $^{n}Bu_3SiH$ (56.0 mg, 280 µmol, 0.700 equiv.). Reaction was stopped after 12 h, and the crude product was purified by flash-column chromatography using pentane as eluent. The title compound (*R*)-**3ah** was isolated as a colorless liquid (102.5 mg, 64.4% yield, 46.6% ee).

$\mathbf{R_f}$ = 0.53 (*n*-pentane:ethyl acetate 50:1). **^{1}H NMR** (400 MHz, C_6D_6): δ/ppm = 7.51–7.48 (m, 4H), 7.43–7.41 (m, 2H), 7.23–7.19 (m, 2H), 7.15–7.11 (m, 1H), 4.89 (q, *J* = 6.3 Hz, 1H), 1.49 (d, *J* = 6.3 Hz, 3H), 1.47–1.32 (m, 12H), 0.92 (t, *J* = 7.1 Hz, 9H), 0.72–0.69 (m, 6H). **^{13}C NMR** (126 MHz, C_6D_6): δ/ppm = 146.4, 141.6, 140.5, 129.0, 127.4, 127.4, 127.3, 126.2, 71.0, 27.7, 27.1, 26.0, 14.3, 14.0. **HRMS** (EI) for $C_{22}H_{31}OSi^+$ $[M–C_4H_9]^+$ calcd *m/z* 339.2139 found 339.2139. **IR** (ATR): $\tilde{\nu}$/cm^{-1} = 3027, 2954, 2919, 2869, 2854, 1600, 1459, 1193, 1092, 1078, 955, 883, 837, 730. **Optical rotation**: $[\alpha]_D^{20}$ = –24.2 (*c* 1.0, $CHCl_3$, 46.6% ee). The enantiomeric excess of **3ah** was determined after deprotection using the same HPLC setup as for **1a**: (Daicel Chiralcel AD-H column, column temperature 20°C, solvent *n*-heptane:isopropanol = 95:5, flow rate 0.8 mL/min, λ = 254 nm): t_R = 21.7 min for (*S*)-**1a**, t_R = 23.8 min for (*R*)-**1a**.

Kinetic resolution data for **1a**

Run	Conversion of *rac*-**1a** [%]	ee of silyl ether (*S*)-**3ah** [%]	ee of unreacted (*R*)-**1a** [%]	Selectivity factor s
1	53.4	70.8	81.2	14.3
2	68.0	46.6	98.8	12.2

(*R*)-1-Phenylethan-1-ol [(*R*)-1b]

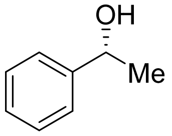

(*R*)-**1b**

$C_8H_{10}O$

M = 122.17 g/mol

1st run: Prepared according to GP1 from *rac*-1-phenylethan-1-ol (*rac*-**1b**, 49.0 mg, 400 µmol, 1.00 equiv.), $^{n}Bu_3SiH$ (**2h**, 52.0 mg, 260 µmol, 0.650 equiv.). Reaction was stopped after 12 h, and the crude product was purified by flash-column chromatography using *n*-pentane/diethyl ether (4:1) as eluent. The title compound (*R*)-**1b** was isolated as colorless oil (11.9 mg, 24.3% yield, 96.2% ee).

2nd run: Prepared according to GP1 from *rac*-1-phenylethan-1-ol (*rac*-**1b**, 122.0 mg, 1.00 mmol, 1.00 equiv.), $^{n}Bu_3SiH$ (130.0 mg, 0.650 mmol, 0.650 equiv.). Reaction was stopped after 12 h, and the crude product was purified by flash-column chromatography using *n*-pentane/diethyl ether (4:1) as eluent. The title compound (*R*)-**1b** was isolated as colorless oil (30.0 mg, 24.6% yield, 95.8% ee).

$\mathbf{R}_f$ = 0.22 (*n*-pentane/diethyl ether 4:1). ^{1}**H NMR** (500 MHz, $CDCl_3$): δ/ppm = 7.28–7.22 (m, 4H), 7.18–7.14 (m, 1H), 4.78 (q, *J* = 6.5 Hz, 1H), 1.82 (s_{br}, 1H), 1.50 (d, *J* = 6.5 Hz, 3H). ^{13}**C NMR** (126 MHz, $CDCl_3$): δ/ppm = 145.9, 128.6, 127.6, 125.5, 70.5, 25.3. **HRMS** (APCI) for $C_8H_9O^+$ $[M–H]^+$ calcd *m/z* 121.0648 found 121.0644. **IR** (ATR): $\tilde{\nu}/cm^{-1}$ = 3333, 2971, 3027, 1600, 1492, 1449, 1202, 1096, 1074, 1009, 896, 758, 696. **Optical rotation**: $[\alpha]_D^{20}$ = +52.6 (*c* 1.09, $CHCl_3$, 95.8% ee); $[\alpha]_D^{20}$ = –48 (*c* 0.17, $CHCl_3$, 93% ee) reported for (*S*)-enantiomer.[9] The enantiomeric excess of (*R*)-**1b** was determined by HPLC analysis on a chiral stationary phase (Daicel Chiralcel OD-H column, column temperature 20°C, solvent *n*-heptane:isopropanol = 95:5, flow rate 0.8 mL/min, λ = 210 nm): t_R = 11.2 min for (*R*)-**1b**, t_R = 13.0 min for (*S*)-**1b**.

The analytical data are in accordance with those reported.[9]

(*S*)-Tributyl(1-phenylethoxy)silane [(*S*)-3bh]

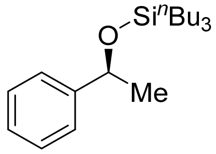

(*S*)-**3bh**

$C_{20}H_{36}OSi$

M = 320.59 g/mol

1st run: Prepared according to GP1 from *rac*-1-phenylethan-1-ol (*rac*-**1b**, 49.0 mg, 400 µmol, 1.00 equiv.), $^{n}Bu_3SiH$ (**2h**, 52.0 mg, 260 µmol, 0.650 equiv.). Reaction was stopped after 12 h, and the crude product was purified by flash-column chromatography using *n*-pentane as eluent. The title compound (*S*)-**3bh** was isolated as colorless oil (74.2 mg, 57.9% yield, 59.4% ee).

2nd run: Prepared according to GP1 from *rac*-1-phenylethan-1-ol (*rac*-**1b**, 122.0 mg, 1.00 mmol, 1.00 equiv.), $^{n}Bu_3SiH$ (130.0 mg, 0.650 mmol, 0.650 equiv.). Reaction was stopped after 12 h, and the crude product was purified by flash-column chromatography using *n*-pentane/diethyl ether (4:1) as eluent. The title compound (*S*)-**3bh** was isolated as a colorless oil (179.6 mg, 56.0% yield, 56.4% ee).

R_f = 0.72 (*n*-pentane/diethyl ether 20:1). **^{1}H NMR** (400 MHz, C_6D_6): δ/ppm = 7.37–7.35 (m, 2H), 7.21–7.17 (m, 2H), 7.10–7.06 (m, 1H), 4.84 (q, *J* = 6.3 Hz, 1H), 1.43 (d, *J* = 6.3 Hz, 3H), 1.42–1.29 (m, 12H), 0.90 (t, *J* = 7.1 Hz, 9H), 0.63–0.68 (m, 6H). **^{13}C NMR** (126 MHz, C_6D_6): δ/ppm = 147.4, 128.5, 127.2, 125.7, 71.3, 27.7, 27.0, 25.9, 14.2, 14.0. **HRMS** (EI) for $C_{16}H_{27}OSi^+$ $[M–C_4H_9]^+$ calcd *m/z* 263.1826 found 263.1831. **IR** (ATR): $\tilde{\nu}$/cm^{-1} = 2955, 2919, 2870, 1491, 1453, 1369, 1297, 1194, 1093, 1078, 955, 792, 756, 697. **Optical rotation**: $[\alpha]_D^{20}$ = –28.0 (*c* 1.26, $CHCl_3$, 56.4% ee). The enantiomeric excess of **3bh** was determined after deprotection using the same HPLC setup as for **1b** (Daicel Chiralcel OD-H column, column temperature 20°C, solvent *n*-heptane:isopropanol = 95:5, flow rate 0.8 mL/min, λ = 210 nm): t_R = 11.2 min for (*R*)-**1b**, t_R = 13.0 min for (*S*)-**1b**.

Kinetic resolution data for **1b**

Run	Conversion of *rac*-**1b** [%]	ee of silyl ether (*S*)-**3bh** [%]	ee of unreacted (*R*)-**1b** [%]	Selectivity factor s
1	61.8	59.4	96.2	14.6
2	62.9	56.4	95.8	12.8

(*R*)-1-(*p*-Tolyl)ethan-1-ol [(*R*)-1c]

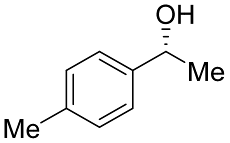

(*R*)-**1c**

$C_9H_{12}O$

M = 136.19 g/mol

Prepared according to GP1 from *rac*-1-(*p*-tolyl)ethan-1-ol (*rac*-**1c**, 54.5 mg, 400 µmol, 1.00 equiv.), $^{n}Bu_3SiH$ (**2h**, 52.0 mg, 260 µmol, 0.650 equiv.). Reaction was stopped after 12 h, and the crude product was purified by flash-column chromatography using *n*-pentane/diethyl ether (4:1) as eluent. The title compound (*R*)-**1c** was isolated as colorless oil (20.6 mg, 37.5% yield, 92.6% ee).

R_f = 0.40 (*n*-pentane/ethyl acetate 5:1). **^{1}H NMR** (500 MHz, $CDCl_3$): δ/ppm = 7.27 (d, *J* = 7.8 Hz, 2H), 7.16 (d, *J* = 7.8 Hz, 2H), 4.87 (q, *J* = 6.5 Hz, 1H), 2.35 (s, 3H), 1.78 (s_{br}, 1H), 1.49 (d, *J* = 6.5 Hz, 3H). **^{13}C NMR** (126 MHz, $CDCl_3$): δ/ppm = 143.0, 137.3, 129.3, 125.5, 70.4, 25.2, 21.2. **HRMS** (APCI) for $C_9H_{11}O^+$ $[M–H]^+$ calcd *m/z* 135.0804 found 135.0803. **IR** (ATR): $\tilde{\nu}/cm^{-1}$ = 3339, 2970, 2922, 1613, 1512, 1447, 1367, 1301, 1200, 1069, 1007, 896, 815, 725. **Optical rotation**: $[\alpha]_D^{20}$ = +53.4 (*c* 0.20, $CHCl_3$, 92.6% ee); $[\alpha]_D^{20}$ = –49 (*c* 0.33, $CHCl_3$, 85% ee) reported for (*S*)-enantiomer.[9] The enantiomeric excess of (*R*)-**1c** was determined by HPLC analysis on a chiral stationary phase (Daicel Chiralcel AD-H column, column temperature 20°C, solvent *n*-heptane:isopropanol = 98:2, flow rate 0.8 mL/min, λ = 210 nm): t_R = 27.3 min for (*R*)-**1c**, t_R = 29.0 min for (*S*)-**1c**.

The analytical data are in accordance with those reported.[9]

(*S*)-Tributyl(1-(*p*-tolyl)ethoxy)silane [(*S*)-3ch]

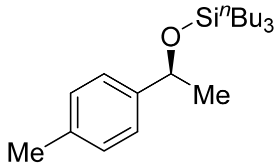

(*S*)-**3ch**

$C_{21}H_{38}OSi$

M = 334.62 g/mol

Prepared according to GP1 from *rac*-1-(*p*-tolyl)ethan-1-ol (*rac*-**1c**, 54.5 mg, 400 μmol, 1.00 equiv.), $^{n}Bu_3SiH$ (**2h**, 52.0 mg, 260 μmol, 0.650 equiv.). Reaction was stopped after 12 h, and the crude product was purified by flash-column chromatography using *n*-pentane/diethyl ether (100:1) as eluent. The title compound (*S*)-**3ch** was isolated as colorless oil (71.1 mg, 53.1% yield, 64.4% ee).

R_f = 0.40 (*n*-pentane). **^{1}H NMR** (500 MHz, C_6D_6): δ/ppm = 7.30 (d, *J* = 8.0 Hz, 2H), 7.03 (d, *J* = 7.9 Hz, 2H), 4.85 (q, *J* = 6.3 Hz, 1H), 2:13 (s, 3H), 1.47 (d, *J* = 6.3 Hz, 3H), 1.44–1.31 (m, 12H), 0.91 (t, *J* = 7.1 Hz, 9H), 0.69–0.65 (m, 6H). **^{13}C NMR** (126 MHz, C_6D_6): δ/ppm = 144.5, 136.5, 129.2, 125.7, 71.2, 27.1, 26.0, 21.1, 14.3, 14.0. **HRMS** (EI) for $C_{17}H_{29}OSi^+$ $[M–C_4H_9]^+$ calcd *m/z* 277.1982 found 277.1985. **IR** (ATR): $\tilde{\nu}$/cm^{-1} = 2955, 2919, 2869, 1512, 1459, 1409, 1369, 1297, 1195, 1092, 1078, 1028, 955, 833, 815, 785, 761, 729. **Optical rotation**: $[\alpha]_D^{20}$ = –30.1 (*c* 1.1, $CHCl_3$, 64.4% ee). The enantiomeric excess of **3ch** was determined after deprotection using the same HPLC setup as for **1c** (Daicel Chiralcel AD-H column, column temperature 20°C, solvent *n*-heptane:isopropanol = 98:2, flow rate 0.8 mL/min, λ = 210 nm): t_R = 27.3 min for (*R*)-**1c**, t_R = 29.0 min for (*S*)-**1c**.

Kinetic resolution data for **1c**

Run	Conversion of *rac*-**1c** [%]	ee of silyl ether (*S*)-**3ch** [%]	ee of unreacted (*R*)-**1c** [%]	Selectivity factor s
1	59.0	64.4	92.6	14.8

(*R*)-1-(*m*-Tolyl)ethan-1-ol [(*R*)-1d]

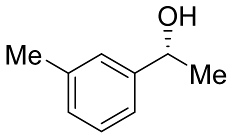

(*R*)-**1d**

$C_9H_{12}O$

M = 136.19 g/mol

Prepared according to GP1 from *rac*-1-(*m*-tolyl)ethan-1-ol (*rac*-**1d**, 54.5 mg, 400 µmol, 1.00 equiv.), ^{n}Bu$_3$SiH (**2h**, 52.0 mg, 260 µmol, 0.650 equiv.). Reaction was stopped after 36 h, and the crude product was purified by flash-column chromatography using *n*-pentane/diethyl ether (5:1) as eluent. The title compound (*R*)-**1d** was isolated as colorless oil (19.4 mg, 35.3% yield, 95.0% ee).

R_f = 0.40 (*n*-pentane/ethyl acetate 5:1). **^{1}H NMR** (500 MHz, C_6D_6): δ/ppm = 7.23–7.14 (m, 3H), 7.01–6.98 (m, 1H), 4.64–4.63 (m, 1H), 2.23 (s, 3H), 1.46 (s_{br}, 1H), 1.40 (d, *J* = 6.4 Hz, 3H). **^{13}C NMR** (126 MHz, C_6D_6): δ/ppm = 146.9, 137.9, 128.5, 128.1, 126.4, 122.8, 70.3, 25.7, 21.5. **HRMS** (APCI) for $C_9H_{11}O^+$ $[M–H]^+$ calcd *m/z* 135.0804 found 135.0802. **IR** (ATR): $\tilde{\nu}$/cm^{-1} = 3339, 2970, 2921, 2866, 1607, 1487, 1447, 1368, 1282, 1159, 1072, 1010, 920, 856, 783, 701. **Optical rotation**: $[\alpha]_D^{20}$ = +42.4 (*c* 0.6, $CHCl_3$, 95.0% ee); $[\alpha]_D^{20}$ = +44.0 (*c* 0.95, $CHCl_3$, 96% ee) reported for (*R*)-enantiomer.[10] The enantiomeric excess of (*R*)-**1d** was determined by HPLC analysis on a chiral stationary phase (Daicel Chiralcel OD-H column, column temperature 20°C, solvent *n*-heptane:isopropanol = 95:5, flow rate 0.8 mL/min, λ = 210 nm): t_R = 13.1 min for (*R*)-**1d**, t_R = 15.3 min for (*S*)-**1d**.

The analytical data are in accordance with those reported.[10]

(*S*)-Tributyl(1-(*m*-tolyl)ethoxy)silane [(*S*)-3dh]

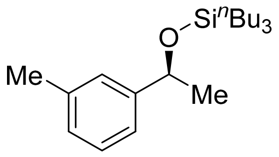

(*S*)-**3dh**

$C_{21}H_{38}OSi$

M = 334.62 g/mol

Prepared according to GP1 from *rac*-1-(*m*-tolyl)ethan-1-ol (*rac*-**1d**, 54.5 mg, 400 µmol, 1.00 equiv.), $^{n}Bu_3SiH$ (**2h**, 52.0 mg, 260 µmol, 0.650 equiv.). Reaction was stopped after 36 h, and the crude product was purified by flash-column chromatography using *n*-pentane/diethyl ether (100:1) as eluent. The title compound (*R*)-**3dh** was isolated as colorless oil (75.7 mg, 56.6% yield, 64.8% ee).

R_f = 0.37 (*n*-pentane). **^{1}H NMR** (400 MHz, C_6D_6): δ/ppm = 7.22–7.21 (m, 2H), 7.25–7.13 (m, 1H), 6.94–6.92 (m, 1H), 4.85 (q, *J* = 6.3 Hz, 1H), 2.18 (s, 3H), 1.47 (d, *J* = 6.3 Hz, 3H), 1.45–1.29 (m, 12H), 0.91 (t, *J* = 7.1 Hz, 9H), 0.70–0.65 (m, 6H). **^{13}C NMR** (126 MHz, C_6D_6): δ/ppm = 147.4, 137.8, 128.5, 128.3, 126.5, 122.9, 71.3, 27.7, 27.1, 26.0, 21.5, 14.3, 14.0. **HRMS** (EI) for $C_{17}H_{29}OSi^+$ $[M–C_4H_9]^+$ calcd *m/z* 277.1982 found 277.1988. **IR** (ATR): $\tilde{\nu}$/cm^{-1} = 2955, 2919, 2869, 1459, 1408, 1369, 1193, 1162, 1111, 1092, 1077, 1032, 961, 885, 784, 762, 701. **Optical rotation**: $[\alpha]_D^{20}$ = –27.6 (*c* 1.4, $CHCl_3$, 64.8% ee). The enantiomeric excess of **3dh** was determined after deprotection using the same HPLC setup as for **1d** (Daicel Chiralcel OD-H column, column temperature 20°C, solvent *n*-heptane:isopropanol = 95:5, flow rate 0.8 mL/min, λ = 210 nm): t_R = 13.1 min for (*R*)-**1d**, t_R = 15.3 min for (*S*)-**1d**.

Kinetic resolution data for **1d**

Run	Conversion of *rac*-**1d** [%]	ee of silyl ether (*S*)-**3dh** [%]	ee of unreacted (*R*)-**1d** [%]	Selectivity factor s
1	59.4	64.8	95.0	16.6

(*R*)-1-(*o*-Tolyl)ethan-1-ol [(*R*)-1e]

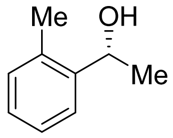

(*R*)-**1e**

$C_9H_{12}O$

M = 136.19 g/mol

Prepared according to GP1 from *rac*-1-(*o*-tolyl)ethan-1-ol (*rac*-**1e**, 54.5 mg, 400 µmol, 1.00 equiv.), ^{n}Bu$_3$SiH (**2h**, 52.0 mg, 260 µmol, 0.650 equiv.). Reaction was stopped after 36 h, and the crude product was purified by flash-column chromatography using *n*-pentane/diethyl ether (4:1) as eluent. The title compound (*R*)-**1e** was isolated as colorless oil (20.1 mg, 36.5% yield, 97.0% ee).

R*f* = 0.40 (*n*-pentane/ethyl acetate 5:1). **^{1}H NMR** (500 MHz, $CDCl_3$): δ/ppm = 7.52–7.50 (m, 1H), 7.25–7.22 (m, 1H), 7.18–7.16 (m, 1H), 7.14–7.12 (m, 1H), 5.14 (q, *J* = 6.5 Hz, 1H), 2.35 (s, 3H), 1.70 (s_{br}, 1H), 1.47 (d, *J* = 6.5 Hz, 3H). **^{13}C NMR** (126 MHz, $CDCl_3$): δ/ppm = 144.0, 134.4, 130.5, 127.3, 126.5, 124.6, 67.0, 24.1, 19.0. **HRMS** (APCI) for $C_9H_{11}O^+$ $[M–H]^+$ calcd *m/z* 135.0804 found 135.0801. **IR** (ATR): $\tilde{\nu}/cm^{-1}$ = 3332, 2970, 2924, 1486, 1457, 1367, 1280, 1214, 1126, 1073, 1003, 942, 894, 809, 756. **Optical rotation**: $[\alpha]_D^{20}$ = +63.0 (*c* 0.5, $CHCl_3$, 97.0% ee); $[\alpha]_D^{20}$ = +73.2 (*c* 0.99, $CHCl_3$, 99.7% ee) reported for (*R*)-enantiomer.[10] The enantiomeric excess of (*R*)-**1e** was determined by HPLC analysis on a chiral stationary phase (Daicel Chiralcel AD-H column, column temperature 20°C, solvent *n*-heptane:isopropanol = 95:5, flow rate 0.8 mL/min, λ = 210 nm): t_R = 13.2 min for (*R*)-**1e**, t_R = 14.6 min for (*S*)-**1e**.

The analytical data are in accordance with those reported.[10]

(*S*)-Tributyl(1-(*o*-tolyl)ethoxy)silane [(*S*)-3eh]

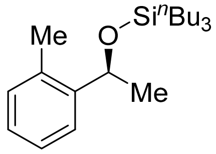

(*S*)-**3eh**

$C_{21}H_{38}OSi$

M = 334.62 g/mol

Prepared according to GP1 from *rac*-1-(*o*-tolyl)ethan-1-ol (*rac*-**1e**, 54.5 mg, 400 µmol, 1.00 equiv.), $^{n}Bu_3SiH$ (**2h**, 52.0 mg, 260 µmol, 0.650 equiv.). Reaction was stopped after 36 h, and the crude product was purified by flash-column chromatography using *n*-pentane/diethyl ether (100:1) as eluent. The title compound (*S*)-**3eh** was isolated as colorless oil (72.2 mg, 57.7% yield, 60.8% ee).

$\mathbf{R_f}$ = 0.37 (*n*-pentane). **^{1}H NMR** (500 MHz, C_6D_6): δ/ppm = 7.74–7.73 (m, 1H), 7.19–7.17 (m, 1H), 7.06–7.03 (m, 1H), 6.98–6.96 (m, 1H), 5.11 (q, *J* = 6.3 Hz, 1H), 2.18 (s, 3H), 1.42 (d, *J* = 6.3 Hz, 3H), 1.40–1.29 (m, 12H), 0.89 (t, *J* = 7.1 Hz, 9H), 0.68–0.64 (m, 6H). **^{13}C NMR** (126 MHz, C_6D_6): δ/ppm = 145.4, 133.0, 130.4, 127.0, 126.6, 125.9, 68.1, 27.1, 26.3, 25.9, 19.0, 14.2, 14.0. **HRMS** (EI) for $C_{17}H_{29}OSi^+$ $[M–C_4H_9]^+$ calcd *m/z* 277.1982 found 277.1994 .**IR** (ATR): $\tilde{\nu}$/cm^{-1} = 2955, 2920, 2869, 1459, 1374, 1193, 1129, 1092, 1075, 1025, 953, 884, 783, 756, 724. **Optical rotation**: $[\alpha]_D^{20}$ = –27.0 (*c* 1.8, $CHCl_3$, 60.8% ee). The enantiomeric excess of **3eh** was determined after deprotection using the same HPLC setup as for **1e** (Daicel Chiralcel AD-H column, column temperature 20°C, solvent *n*-heptane:isopropanol = 95:5, flow rate 0.8 mL/min, λ = 210 nm): t_R = 13.2 min for (*R*)-**1e**, t_R = 14.6 min for (*S*)-**1e**.

Kinetic resolution data for **1e**

Run	Conversion of *rac*-**1e** [%]	ee of silyl ether (*S*)-**3eh** [%]	ee of unreacted (*R*)-**1e** [%]	Selectivity factor s
1	61.5	60.8	97.0	16.1

(*R*)-1-(2,4-Dimethylphenyl)ethan-1-ol [(*R*)-1f]

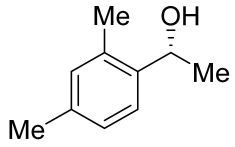

(*R*)-**1f**

$C_{10}H_{14}O$

M = 150.22 g/mol

Prepared according to GP1 from *rac*-1-(2,4-dimethylphenyl)ethan-1-ol (*rac*-**1f**, 60.0 mg, 400 µmol, 1.00 equiv.), $^{n}Bu_3SiH$ (**2h**, 44.0 mg, 220 µmol, 0.550 equiv.). Reaction was stopped after 18 h, and the crude product was purified by flash-column chromatography using *n*-pentane/diethyl ether (4:1) as eluent. The title compound (*R*)-**1f** was isolated as colorless oil (25.3 mg, 42.2% yield, 85.8% ee).

$\mathbf{R_f}$ = 0.48 (*n*-pentane/diethyl ether 4:1). **^{1}H NMR** (400 MHz, C_6D_6): δ/ppm = 7.45 (d, 7.8 Hz, 1H), 6.96 (d, *J* = 7.8 Hz, 1H), 6.79 (s, 1H), 4.81 (q, *J* = 6.5 Hz, 1H), 2.14 (s, 3H), 2.09 (s, 3H), 1.85 (s_{br}, 1H), 1.32 (d, *J* = 6.5 Hz, 3H). **^{13}C NMR** (126 MHz, C_6D_6): δ/ppm = 141.8, 136.3, 134.1, 131.3, 127.2, 125.1, 66.7, 24.3, 21.0, 18.8. **HRMS** (APCI) for $C_{10}H_{13}O^+$ $[M–H]^+$ calcd *m/z* 149.0961 found 149.0958. **IR** (ATR): $\tilde{\nu}$/cm^{-1} = 3339, 2969, 2921, 1614, 1499, 1445, 1366, 1285, 1237, 1127, 1070, 1006, 930, 888, 819, 747, 709. $[\alpha]_D^{20}$ = +50.7 (*c* 1.1, $CHCl_3$, 85.8% ee); $[\alpha]_D^{25}$ = +47.9 (*c* 1.7, $CHCl_3$, 74% ee) reported for (*R*)-enantiomer.[11] The enantiomeric excess of (*R*)-**1f** was determined by HPLC analysis on a chiral stationary phase (Daicel Chiralcel OD-H column, column temperature 20°C, solvent *n*-heptane:isopropanol = 95:5, flow rate 0.8 mL/min, λ = 210 nm): t_R = 12.1 min for (*R*)-**1f**, t_R = 13.9 min for (*S*)-**1f**.

The analytical data are in accordance with those reported.[11,12]

(*S*)-Tributyl(1-(2,4-dimethylphenyl)ethoxy)silane [(*S*)-3fh]

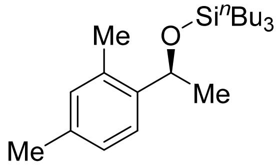

(*S*)-**3fh**

$C_{22}H_{40}OSi$

M = 348.65 g/mol

Prepared according to GP1 from *rac*-1-(2,4-dimethylphenyl)ethan-1-ol (*rac*-**1f**, 60.0 mg, 400 μmol, 1.00 equiv.), $^{n}Bu_3SiH$ (**2h**, 44.0 mg, 220 μmol, 0.550 equiv.). Reaction was stopped after 18 h, and the crude product was purified by flash-column chromatography using *n*-pentane/diethyl ether (100:1) as eluent. The title compound (*R*)-**3fh** was isolated as colorless oil (73.0 mg, 52.1% yield, 72.2% ee).

$\mathbf{R_f}$ = 0.73 (*n*-pentane/diethyl ether 20:1). **^{1}H NMR** (500 MHz, C_6D_6): δ/ppm = 7.65 (d, *J* = 7.9 Hz, 1H), 7.00 (d, *J* = 7.9 Hz, 1H), 6.81 (s, 1H), 5.11 (q, *J* = 6.3 Hz, 1H), 2.20 (s, 3H), 2.14 (s, 3H), 1.45 (d, *J* = 6.3 Hz, 3H), 1.42–1.30 (m, 12H), 0.90 (t, *J* = 7.1 Hz, 9H), 0.70–0.65 (m, 6H). **^{13}C NMR** (126 MHz, C_6D_6): δ/ppm = 142.5, 136.1, 132.9, 131.2, 127.3, 126.0, 68.1, 27.1, 26.4, 26.0, 21.0, 19.0, 14.3, 14.0. **HRMS** (EI) for $C_{18}H_{31}OSi^+$ $[M–C_4H_9]^+$ calcd *m/z* 291.2139 found 291.2146. **IR** (ATR): $\tilde{\nu}$/cm^{-1} = 2955, 2919, 2870, 1458, 1408, 1374, 1193, 1132, 1092, 1076, 1027, 957, 883, 820, 789, 760, 727. **Optical rotation**: $[\alpha]_D^{20}$ = –32.3 (*c* 1.1, $CHCl_3$, 72.2% ee). The enantiomeric excess of **3fh** was determined after deprotection using the same HPLC setup as for **1f** (Daicel Chiralcel OD-H column, column temperature 20°C, solvent *n*-heptane:isopropanol = 95:5, flow rate 0.8 mL/min, λ = 210 nm): t_R = 12.1 min for (*R*)-**1f**, t_R = 13.9 min for (*S*)-**1f**.

Kinetic resolution data for **1f**

Run	Conversion of *rac*-**1f** [%]	ee of silyl ether (*S*)-**3fh** [%]	ee of unreacted (*R*)-**1f** [%]	Selectivity factor s
1	54.3	72.2	85.8	16.4

(*R*)-1-(3,5-Dimethylphenyl)ethan-1-ol [(*R*)-1g]

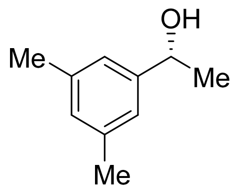

(*R*)-**1g**

$C_{10}H_{14}O$

M = 150.22 g/mol

Prepared according to GP1 from *rac*-1-(3,5-dimethylphenyl)ethan-1-ol (*rac*-**1g**, 60.0 mg, 400 µmol, 1.00 equiv.), $^{n}Bu_3SiH$ (**2h**, 48.0 mg, 240 µmol, 0.600 equiv.). Reaction was stopped after 18 h, and the crude product was purified by flash-column chromatography using *n*-pentane/diethyl ether (4:1) as eluent. The title compound (*R*)-**1g** was isolated as colorless oil (24.2 mg, 40.7% yield, 89.9% ee).

$\mathbf{R_f}$ = 0.43 (*n*-pentane/diethyl ether 5:1). **^{1}H NMR** (400 MHz, C_6D_6): δ/ppm = (500 MHz, C_6D_6): δ/ppm = 6.94 (s, 2H), 6.75 (s, 1H), 4.61 (q, J = 6.4 Hz, 1H), 2.19 (s_{br}, 1H), 2.16 (m, 6H), 1.37 (d, J = 6.5 Hz, 3H). **^{13}C NMR** (126 MHz, C_6D_6): δ/ppm = 146.9, 137.8, 129.0, 123.7, 70.3, 25.7, 21.4. **HRMS** (APCI) for $C_{10}H_{13}O^+$ $[M–H]^+$ calcd *m/z* 149.0961 found 149.0959. **IR** (ATR): $\tilde{\nu}$/cm^{-1} = 3338, 2970, 2917, 0865, 1606, 1449, 1368, 1292, 1161, 1072, 1032, 929, 847, 704. **Optical rotation**: $[\alpha]_D^{20}$ = +39.2 (*c* 0.3, $CHCl_3$, 89.9% ee); $[\alpha]_D^{20}$ = +38.9 (*c* 1.0, $CHCl_3$, 97% ee) reported for (*R*)-enantiomer.[13,14] The enantiomeric excess of (*R*)-**1g** was determined by HPLC analysis on a chiral stationary phase (Daicel Chiralcel OD-H column, column temperature 20°C, solvent *n*-heptane:isopropanol = 99:1, flow rate 0.8 mL/min, λ = 210 nm): t_R = 30.5 min for (*R*)-**1g**, t_R = 33.5 min for (*S*)-**1g**.

The analytical data are in accordance with those reported.[13,14]

(*S*)-Tributyl(1-(3,5-dimethylphenyl)ethoxy)silane [(*S*)-3gh]

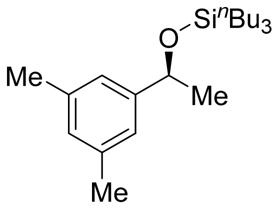

(*S*)-**3gh**

$C_{22}H_{40}OSi$

M = 348.65 g/mol

Prepared according to GP1 from *rac*-1-(3,5-dimethylphenyl)ethan-1-ol (*rac*-**1g**, 60.0 mg, 400 µmol, 1.00 equiv.), $^{n}Bu_3SiH$ (**2h**, 48.0 mg, 240 µmol, 0.600 equiv.). Reaction was stopped after 18 h, and the crude product was purified by flash-column chromatography using *n*-pentane/diethyl ether (100:1) as eluent. The title compound (*S*)-**3gh** was isolated as colorless oil (71.2 mg, 51.0% yield, 73.2% ee).

$\mathbf{R_f}$ = 0.74 (*n*-pentane/diethyl ether 20:1). **^{1}H NMR** (500 MHz, C_6D_6): δ/ppm = 7.07 (s, 2H), 6.76 (s, 1H), 4.85 (q, *J* = 6.3 Hz, 1H), 2.20 (m, 6H), 1.51 (d, *J* = 6.3 Hz, 3H), 1.45–1.32 (m, 12H), 0.91 (t, *J* = 7.2 Hz, 9H), 0.71–0.67 (m, 6H). **^{13}C NMR** (126 MHz, C_6D_6): δ/ppm = 147.3, 137.7, 128.9, 123.7, 71.4, 30.2, 27.8, 27.1, 26.0, 21.4, 14.3, 14.0. **HRMS** (EI) for $C_{18}H_{31}OSi^+$ $[M–C_4H_9]^+$ calcd *m/z* 291.2139 found 291.2147. **IR** (ATR): $\tilde{\nu}/cm^{-1}$ = 2955, 2919, 2869, 2856, 1606, 1460, 1408, 1374, 1163, 1110, 1091, 1080, 1046, 964, 891, 847, 785, 761, 730, 704. **Optical rotation**: $[\alpha]_D^{20}$ = –30.7 (*c* 1.4, $CHCl_3$, 73.2% ee). The enantiomeric excess of **3gh** was determined after deprotection using the same HPLC setup as for **1g** (Daicel Chiralcel OD-H column, column temperature 20°C, solvent *n*-heptane:isopropanol = 99:1, flow rate 0.8 mL/min, λ = 210 nm): t_R = 30.5 min for (*R*)-**1g**, t_R = 33.5 min for (*S*)-**1g**.

Kinetic resolution data for **1g**

Run	Conversion of *rac*-**1g** [%]	ee of silyl ether (*S*)-**3gh** [%]	ee of unreacted (*R*)-**1g** [%]	Selectivity factor s
1	55.1	73.2	89.9	19.2

(*R*)-1-(3,5-Bis(trifluoromethyl)phenyl)ethan-1-ol [(*R*)-1h]

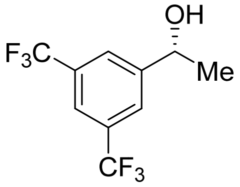

(*R*)-**1h**

$C_{10}H_8F_6O$

M = 258.16 g/mol

1st run: Prepared according to GP1 from *rac*-1-(3,5-bis(trifluoromethyl)phenyl)ethan-1-ol (*rac*-**1h**, 103mg, 400 μmol, 1.00 equiv.), ^{n}Bu$_3$SiH (**2h**, 44.0 mg, 220 μmol, 0.550 equiv.). Reaction was stopped after 96 h, and the crude product was purified by flash-column chromatography using *n*-pentane/diethyl ether (5:1) as eluent. The title compound (*R*)-**1h** was isolated as white solid (35.3 mg, 34.2% yield, 86.6% ee).

2nd run: Prepared according to GP1 from *rac*-1-(3,5-bis(trifluoromethyl)phenyl)ethan-1-ol (*rac*-**1h**, 103mg, 400 μmol, 1.00 equiv.), ^{n}Bu$_3$SiH (**2h**, 44.0 mg, 220 μmol, 0.550 equiv.). Reaction was stopped after 96 h, and the crude product was purified by flash-column chromatography using *n*-pentane/diethyl ether (5:1) as eluent. The title compound (*R*)-**1h** was isolated as white solid (37.0 mg, 35.8% yield, 79.6% ee).

M.p.: 74–75°C. **R_f** = 0.24 (*n*-pentane/diethyl ether 5:1). **^{1}H NMR** (400 MHz, C_6D_6): δ/ppm = (400 MHz, C_6D_6): δ/ppm = 7.66 (s, 1H), 7.50 (s, 2H), 4.10 (q, *J* = 6.5 Hz, 1H), 0.96 (s_{br}, 1H), 0.90 (d, *J* = 6.5 Hz, 3H). **^{13}C NMR** (101 MHz, C_6D_6): δ/ppm = 149.4, 131.7 (q, *J* = 32.9 Hz), 125.8 (d, *J* = 3.0 Hz), 124.1 (q, *J* = 272.8 Hz), 121.1–121.0 (m), 68.6, 25.2. **^{19}F NMR** (188 MHz, C_6D_6): δ/ppm = –62.6. HRMS (APCI) for $C_{10}H_7F_6^+$ [M–OH]$^+$ calcd *m/z* 241.0446 found 241.0443. **IR** (ATR): $\tilde{\nu}$/cm^{-1} = 3246, 3150, 2978, 1457, 1376, 1338, 1273, 1158, 1111, 1023, 895, 840, 680. **Optical rotation**: $[\alpha]_D^{20}$ = +20.4 (*c* 0.95, $CHCl_3$, 79.6% ee); $[\alpha]_D^{20}$ = +22.1 (*c* 1.01, $CHCl_3$, 95.6% ee) reported for (*R*)-enantiomer.[10] The enantiomeric excess of (*R*)-**1h** was determined by HPLC analysis on a chiral stationary phase (Daicel Chiralcel OD-H column, column temperature 20°C, solvent *n*-heptane:isopropanol = 98:2, flow rate 0.8 mL/min, λ = 210 nm): t_R = 10.4 min for (*S*)-**1h**, t_R = 12.0 min for (*R*)-**1h**.

The analytical data are in accordance with those reported.[10]

(*S*)-(1-(3,5-Bis(trifluoromethyl)phenyl)ethoxy)tributylsilane [(*S*)-3hh]

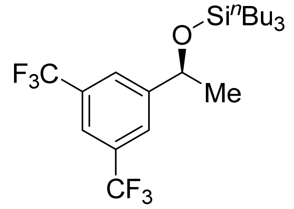

(*S*)-**3hh**

$C_{22}H_{34}F_6OSi$

M = 456.59 g/mol

1st run: Prepared according to GP1 from *rac*-1-(3,5-bis(trifluoromethyl)phenyl)ethan-1-ol (*rac*-**1h**, 103mg, 400 µmol, 1.00 equiv.), ^{n}Bu$_3$SiH (**2h**, 44.0 mg, 220 µmol, 0.550 equiv.). Reaction was stopped after 96 h, and the crude product was purified by flash-column chromatography using *n*-pentane as eluent. The title compound (*S*)-**3hh** was isolated as colorless oil (79.6 mg, 43.5% yield, 84.8% ee).

2nd run: Prepared according to GP1 from *rac*-1-(3,5-bis(trifluoromethyl)phenyl) ethan-1-ol (*rac*-**1h**, 103mg, 400 µmol, 1.00 equiv.), ^{n}Bu$_3$SiH (**2h**, 44.0 mg, 220 µmol, 0.550 equiv.). Reaction was stopped after 96 h, and the crude product was purified by flash-column chromatography using *n*-pentane as eluent. The title compound (*S*)-**3hh** was isolated as colorless oil (80.7 mg, 44.2% yield, 85.8% ee).

R$_f$ = 0.61 (*n*-pentane). **^{1}H NMR** (500 MHz, C_6D_6): δ/ppm = 7.74 (s, 2H), 7.70 (s, 1H), 4.60 (q, *J* = 6.4 Hz, 1H), 1.38–1.23 (m, 12H), 1.14 (d, *J* = 6.4 Hz, 3H), 0.89 (t, *J* = 7.1 Hz, 9H), 0.66–0.44 (m, 6H). **^{13}C NMR** (126 MHz, C_6D_6): δ/ppm = 150.2, 131.9 (q, *J* = 33.0 Hz), 125.9 (d, *J* = 2.9 Hz), 124.1 (q, *J* = 272.8 Hz), 121.14–120.93 (m), 69.8, 30.2, 26.9, 25.8, 14.0, 13.9. **^{19}F NMR** (188 MHz, C_6D_6): δ/ppm = –62.6. **HRMS** (EI) for $C_{18}H_{25}F_6OSi^+$ $[M–C_4H_9]^+$ calcd *m/z* 399.1573 found 399.1581. **IR** (ATR): $\tilde{\nu}$/cm^{-1} = 2958, 2923, 2872, 1462, 1380, 1351, 1276, 1172, 1131, 1100, 1038, 965, 895, 868, 781, 727, 707, 681. **Optical rotation**: $[\alpha]_D^{20}$ = –26.3 (*c* 1.2, $CHCl_3$, 85.8% ee). The enantiomeric excess of **3hh** was determined after deprotection using the same HPLC setup as for **1h** (Daicel Chiralcel OD-H column, column temperature 20°C, solvent *n*-heptane:isopropanol = 98:2, flow rate 0.8 mL/min, λ = 210 nm): t_R = 10.4 min for (*S*)-**1h**, t_R = 12.0 min for (*R*)-**1h**.

Kinetic resolution data for **1h**

Run	Conversion of *rac*-**1h** [%]	ee of silyl ether (*S*)-**3hh** [%]	ee of unreacted (*R*)-**1h** [%]	Selectivity factor s
1	46.9	84.8	86.6	36.1
2	48.1	85.8	79.6	31.9

(*R*)-1-(3,5-Dimethoxyphenyl)ethan-1-ol [(*R*)-1i]

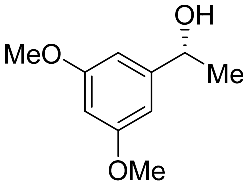

(*R*)-**1i**

$C_{10}H_{14}O_3$

M = 182.22 g/mol

Prepared according to GP1 from *rac*-1-(3,5-dimethoxyphenyl)ethan-1-ol (*rac*-**1i**, 75.0 mg, 400 µmol, 1.00 equiv.), $^{n}Bu_3SiH$ (**2h**, 44.0 mg, 220 µmol, 0.550 equiv.). Reaction was stopped after 36 h, and the crude product was purified by flash-column chromatography using *n*-pentane/diethyl ether (4:1) as eluent. The title compound (*R*)-**1i** was isolated as colorless oil (37.8 mg, 50.4% yield, 80.4% ee).

$\mathbf{R}_f$ = 0.13 (cyclohexane/ethyl acetate 5:1). ^{1}**H NMR** (500 MHz, $CDCl_3$): δ/ppm = 6.52 (d, *J* = 2.3 Hz, 2H), 6.35 (t, *J* = 2.3 Hz, 1H), 4.81 (q, *J* = 6.4 Hz, 1H), 3.78 (s, 6H), 2.08 (s_{br}, 1H), 1.46 (d, *J* = 6.5 Hz, 3H). ^{13}**C NMR** (101 MHz, $CDCl_3$): δ/ppm = 161.0, 148.7, 103.4, 99.4, 70.5, 55.4, 25.2. **HRMS** (APCI) for $C_{10}H_{13}O_3^+$ $[M–H]^+$ calcd *m/z* 181.0859 found 181.0857.**IR** (ATR): $\tilde{\nu}$/cm^{-1} = 3390, 2967, 2934, 2836, 1593, 1456, 1526, 1343, 1291, 1201, 1147, 1101, 1049, 922, 834, 697. **Optical rotation**: $[\alpha]_D^{20}$ = +35.4 (*c* 1.5, $CHCl_3$, 80.4% ee); $[\alpha]_D^{25}$ = –32.7 (*c* 2.0, $CHCl_3$, 97% ee) reported for (*S*)-enantiomer.[15] The enantiomeric excess of (*R*)-**1i** was determined by HPLC analysis on a chiral stationary phase (Daicel Chiralcel OD-H column, column temperature 20°C, solvent *n*-heptane:isopropanol = 90:10, flow rate 0.8 mL/min, λ = 210 nm): t_R = 15.5 min for (*R*)-**1i**, t_R = 23.1 min for (*S*)-**1i.**

The analytical data are in accordance with those reported.[15]

(*S*)-Tributyl(1-(3,5-dimethoxyphenyl)ethoxy)silane [(*S*)-3ih]

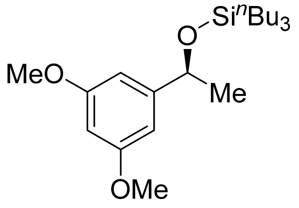

(*S*)-**3ih**

$C_{22}H_{40}O_3Si$

M = 380.64 g/mol

Prepared according to GP1 from *rac*-1-(3,5-dimethoxyphenyl)ethan-1-ol (*rac*-**1i**, 75.0 mg, 400 µmol, 1.00 equiv.), ^{n}Bu$_3$SiH (**2h**, 44.0 mg, 220 µmol, 0.550 equiv.). Reaction was stopped after 36 h, and the crude product was purified by flash-column chromatography using *n*-pentane/diethyl ether (50:1) as eluent. The title compound (*S*)-**3ih** was isolated as colorless oil (70.0 mg, 44.6% yield, 91.2% ee).

$\mathbf{R_f}$ = 0.13 (cyclohexane/ethyl acetate 5:1). **^{1}H NMR** (500 MHz, C_6D_6): δ/ppm = 6.73 (d, *J* = 2.3 Hz, 2H), 6.47 (t, *J* = 2.3 Hz, 1H), 4.83 (q, *J* = 6.3 Hz, 1H), 3.40 (s, 6H), 1.48 (d, *J* = 6.3 Hz, 3H), 1.45–1.31 (m, 12H), 0.90 (t, *J* = 7.2 Hz, 9H), 0.73–0.63 (m, 6H). **^{13}C NMR** (126 MHz, C_6D_6): δ/ppm = 161.6, 150.0, 103.8, 99.4, 71.4, 54.9, 27.7, 27.1, 26.0, 14.3, 14.0. **HRMS** (EI) for $C_{18}H_{31}O_3Si^+$ $[M–C_4H_9]^+$ calcd *m/z* 323.2037 found 323.2042. **IR** (ATR): $\tilde{\nu}$ /cm^{-1} = 2954, 2920, 2860, 1596, 1459, 1426, 1350, 1294, 1203, 1152, 1092, 1055, 974, 895, 839, 786, 730, 696. **Optical rotation**: $[\alpha]_D^{20}$ = –40.7 (*c* 0.6, $CHCl_3$, 91.2% ee). The enantiomeric excess of **3ih** was determined after deprotection using the same HPLC setup as for **1i** (Daicel Chiralcel OD-H column, column temperature 20°C, solvent *n*-heptane:isopropanol = 90:10, flow rate 0.8 mL/min, λ = 210 nm): t_R = 15.5 min for (*R*)-**1i**, t_R = 23.1 min for (*S*)-**1i.**

Kinetic resolution data for **1i**

Run	Conversion of *rac*-**1i** [%]	ee of silyl ether (*S*)-**3ih** [%]	ee of unreacted (*R*)-**1i** [%]	Selectivity factor s
1	46.9	91.2	80.4	52.6

(*R*)-1-(2,6-Dimethylphenyl)ethan-1-ol [(*R*)-1j]

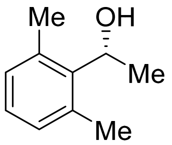

(*R*)-**1j**

$C_{10}H_{14}O$

M = 150.22 g/mol

1st run: Prepared according to GP1 from *rac*-1-(2,6-dimethylphenyl)ethan-1-ol (*rac*-**1j**, 60.0 mg, 400 µmol, 1.00 equiv.), $^{n}Bu_3SiH$ (**2h**, 44.0 mg, 220 µmol, 0.550 equiv.). Reaction was stopped after 36 h, and the crude product was purified by flash-column chromatography using *n*-pentane/diethyl ether (8:1) as eluent. The title compound (*R*)-**1j** was isolated as white solid (24.5 mg, 41.0% yield, 91.4% ee).

2nd run: Prepared according to GP1 from *rac*-1-(2,6-dimethylphenyl)ethan-1-ol (*rac*-**1j**, 60.0 mg, 400 µmol, 1.00 equiv.), $^{n}Bu_3SiH$ (**2h**, 44.0 mg, 220 µmol, 0.550 equiv.). Reaction was stopped after 36 h, and the crude product was purified by flash-column chromatography using *n*-pentane/diethyl ether (8:1) as eluent. The title compound (*R*)-**1j** was isolated as white solid (23.9 mg, 39.8% yield, >99.5% ee).

M.p.: 69–70°C. $\mathbf{R_f}$ = 0.48 (*n*-pentane/diethyl ether 4:1). **^{1}H NMR** (500 MHz, C_6D_6): δ/ppm = 6.99–6.96 (m, 1H), 6.90 (d, J = 7.5 Hz, 2H), 4.99 (q, J = 6.5 Hz, 1H), 2.30 (s, 6H), 1.33 (d, J = 6.5 Hz, 3H), 1.00 (s_{br}, 1H). **^{13}C NMR** (126 MHz, C_6D_6): δ/ppm = 141.4, 135.7, 129.7, 127.0, 67.5, 21.7, 20.7. **HRMS** (APCI) for $C_{10}H_{13}O^+$ $[M–H]^+$ calcd *m/z* 149.0961 found 149.0959. **IR** (ATR): $\tilde{\nu}/cm^{-1}$ = 3294, 2977, 2927, 1581, 1467, 1410, 1365, 1297, 1191, 1070, 1024, 999, 911, 891, 711, 698, 666. **Optical rotation**: $[\alpha]_D^{20}$ = +64.6 (*c* 0.81, $CHCl_3$, >99.5% ee). Absolute configuration was assigned based on analogy. The enantiomeric excess of (*R*)-**1j** was determined by HPLC analysis on a chiral stationary phase (Daicel Chiralcel OD-H column, column temperature 20°C, solvent *n*-heptane:isopropanol = 95:5, flow rate 0.8 mL/min, λ = 210 nm): t_R = 8.8 min for (*S*)-**1j**, t_R = 10.9 min for (*R*)-**1j**.

The analytical data are in accordance with those reported.[16,17]

(*S*)-Tributyl(1-(2,6-dimethylphenyl)ethoxy)silane [(*S*)-3jh]

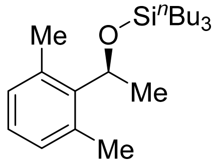

(*S*)-**3jh**

$C_{22}H_{40}OSi$

M = 348.65 g/mol

1st run: Prepared according to GP1 from *rac*-1-(2,6-dimethylphenyl)ethan-1-ol (*rac*-**1j**, 60.0 mg, 400 µmol, 1.00 equiv.), $^{n}Bu_3SiH$ (**2h**, 44.0 mg, 220 µmol, 0.550 equiv.). Reaction was stopped after 36 h, and the crude product was purified by flash-column chromatography using *n*-pentane as eluent. The title compound (*S*)-**3jh** was isolated as colorless oil (57.6 mg, 41.0% yield, 96.2% ee).

2nd run: Prepared according to GP1 from *rac*-1-(2,6-dimethylphenyl)ethan-1-ol (*rac*-**1j**, 60.0 mg, 400 µmol, 1.00 equiv.), $^{n}Bu_3SiH$ (**2h**, 44.0 mg, 220 µmol, 0.550 equiv.). Reaction was stopped after 36 h, and the crude product was purified by flash-column chromatography using *n*-pentane as eluent. The title compound (*S*)-**3jh** was isolated as colorless oil (68.0 mg, 48.8% yield, 89.2% ee).

$\mathbf{R_f}$ = 0.25 (*n*-pentane). **^{1}H NMR** (500 MHz, C_6D_6): δ/ppm = 7.00–6.96 (m, 1H), 6.92 (d, *J* = 7.4 Hz, 2H), 5.34 (q, *J* = 6.5 Hz, 1H), 2.45 (s, 6H), 1.48 (d, *J* = 6.5 Hz, 3H), 1.39–1.28 (m, 12H), 0.89 (t, *J* = 7.1 Hz, 9H), 0.66–0.62 (m, 6H). **^{13}C NMR** (126 MHz, C_6D_6): δ/ppm = 141.8, 126.9, 68.2, 27.1, 25.9, 23.3, 20.8, 14.1, 14.0. **HRMS** (EI) for $C_{18}H_{31}OSi^+$ $[M–C_4H_9]^+$ calcd *m/z* 291.2139 found 291.2141. **IR** (ATR): $\tilde{\nu}$/cm^{-1} = 2955, 2920, 2869, 2854, 1460, 1375, 1194, 1077, 1038, 1013, 954, 884, 806, 765, 730. **Optical rotation**: $[\alpha]_D^{20}$ = –30.3 (*c* 1.68, $CHCl_3$, 89.2% ee). The enantiomeric excess of **3jh** was determined after deprotection using the same HPLC setup as for **1j** (Daicel Chiralcel OD-H column, column temperature 20°C, solvent *n*-heptane:isopropanol = 95:5, flow rate 0.8 mL/min, λ = 210 nm): t_R = 8.8 min for (*S*)-**1j**, t_R = 10.9 min for (*R*)-**1j.**

Kinetic resolution data for **1j**

Run	Conversion of *rac*-**1j** [%]	ee of silyl ether (*S*)-**3jh** [%]	ee of unreacted (*R*)-**1j** [%]	Selectivity factor s
1	48.7	96.2	91.4	170
2	52.7	89.2	>99.5	>100

(*R*)-1-Mesitylethan-1-ol [(*R*)-1k]

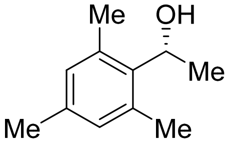

(*R*)-**1k**

$C_{11}H_{16}O$

M = 164.25 g/mol

1st run: Prepared according to GP1 from *rac*-1-mesitylethan-1-ol (*rac*-**1k**, 33.0 mg, 200 µmol, 1.00 equiv.), ^{n}Bu$_3$SiH (**2h**, 22.0 mg, 110 µmol, 0.550 equiv.). Reaction was stopped after 36 h, and the crude product was purified by flash-column chromatography using *n*-pentane/diethyl ether (5:1) as eluent. The title compound (*R*)-**1k** was isolated as white solid (15.3 mg, 46.4% yield, 97.4% ee).

2nd run: Prepared according to GP1 from *rac*-1-mesitylethan-1-ol (*rac*-**1k**, 66.0 mg, 400 µmol, 1.00 equiv.), ^{n}Bu$_3$SiH (**2h**, 48.0 mg, 240 µmol, 0.600 equiv.). Reaction was stopped after 36 h, and the crude product was purified by flash-column chromatography using *n*-pentane/diethyl ether (8:1) as eluent. The title compound (*R*)-**1k** was isolated as white solid (27.8 mg, 42.1% yield, >99.5% ee).

M.p.: 72–73°C. **R_f** = 0.52 (*n*-pentane/ethyl acetate 5:1). **^{1}H NMR** (500 MHz, $CDCl_3$): δ/ppm = 6.82 (s, 2H), 5.36 (q, *J* = 6.8 Hz, 1H), 2.42 (s, 6H), 2.25 (s, 3H), 1.70 (s_{br}, 1H), 1.53 (d, *J* = 6.8 Hz, 3H). **^{13}C NMR** (126 MHz, $CDCl_3$): δ/ppm = 137.8, 136.6, 135.8, 130.3, 67.6, 21.7, 20.8, 20.6. **HRMS** (APCI) for $C_{11}H_{15}O^+$ $[M–H]^+$ calcd *m/z* 163.1117 found 163.1115. **IR** (ATR): $\tilde{\nu}$/cm^{-1} = 3191, 2966, 2917, 1609, 1443, 1363, 1312, 1209, 1149, 1094, 1073, 1030, 999, 892, 848, 743, 719, 660. **Optical rotation**: $[\alpha]_D^{20}$ = +64.1 (*c* 1.0, $CHCl_3$, 97.4% ee); $[\alpha]_D^{20}$ = +66.2 (*c* 5, $CHCl_3$, 99.7% ee) reported for (*R*)-enantiomer.[18] The enantiomeric excess of (*R*)-**1k** was determined by HPLC analysis on a chiral stationary phase (Daicel Chiralcel OD-H column, column temperature 20°C, solvent *n*-heptane:isopropanol = 99:1, flow rate 0.8 mL/min, λ = 210 nm): t_R = 29.2 min for (*R*)-**1k**, t_R = 34.3 min for (*S*)-**1k**.

The analytical data are in accordance with those reported.[18]

(*S*)-Tributyl(1-mesitylethoxy)silane [(*S*)-3kh]

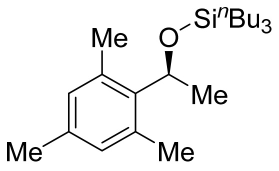

(*S*)-**3kh**

$C_{23}H_{42}OSi$

M = 362.67 g/mol

1st run: Prepared according to GP1 from *rac*-1-mesitylethan-1-ol (*rac*-**1k**, 33.0 mg, 200 µmol, 1.00 equiv.), ^{n}Bu$_3$SiH (**2h**, 22.0 mg, 110 µmol, 0.550 equiv.). Reaction was stopped after 36 h, and the crude product was purified by flash-column chromatography using *n*-pentane/diethyl ether (50:1) as eluent. The title compound (*S*)-**3kh** was isolated as colorless oil (28.4 mg, 39.1% yield, 92.4% ee).

2nd run: Prepared according to GP1 from *rac*-1-mesitylethan-1-ol (*rac*-**1k**, 66.0 mg, 400 µmol, 1.00 equiv.), ^{n}Bu$_3$SiH (**2h**, 48.0 mg, 240 µmol, 0.600 equiv.). Reaction was stopped after 36 h, and the crude product was purified by flash-column chromatography using *n*-pentane/diethyl ether (50:1) as eluent. The title compound (*S*)-**3kh** was isolated as colorless oil (70.8 mg, 48.4% yield, 81.8% ee).

R$_f$ = 0.53 (*n*-pentane). **^{1}H NMR** (500 MHz, C_6D_6): δ/ppm = 6.75 (s, 2H), 5.35 (q, *J* = 6.6 Hz, 1H), 2.46 (s, 6H), 2.13 (s, 3H), 1.52 (d, *J* = 6.6 Hz, 3H), 1.39–1.30 (m, 12H), 0.89 (t, *J* = 7.1 Hz, 9H), 0.69–0.62 (m, 6H). **^{13}C NMR** (126 MHz, C_6D_6): δ/ppm = 138.9, 135.9, 68.1, 27.1, 25.9, 23.5, 20.8, 20.8, 14.2, 14.0. **HRMS** (EI) for $C_{22}H_{39}OSi^+$ $[M–CH_3]^+$ calcd *m/z* 347.2765 found 347.2775. **IR** (ATR): $\tilde{\nu}$/cm^{-1} = 2955, 2919, 2869, 1611, 1458, 1408, 1375, 1193, 1153, 1077, 1039, 1013, 964, 948, 883, 849, 790, 759, 724. **Optical rotation**: $[\alpha]_D^{20}$ = –31.3 (*c* 1.6, $CHCl_3$, 81.8% ee). The enantiomeric excess of **3kh** was determined after deprotection using the same HPLC setup as for **1k** (Daicel Chiralcel OD-H column, column temperature 20°C, solvent *n*-heptane:isopropanol = 99:1, flow rate 0.8 mL/min, λ = 210 nm): t_R = 29.2 min for (*R*)-**1k**, t_R = 34.3 min for (*S*)-**1k**.

Kinetic resolution data for **1k**

Run	Conversion of *rac*-**1k** [%]	ee of silyl ether (*S*)-**3kh** [%]	ee of unreacted (*R*)-**1k** [%]	Selectivity factor s
1	51.3	92.4	97.4	110
2	54.9	81.8	>99.5	>50

(*R*)-1-([1,1'-Biphenyl]-4-yl)propan-1-ol [(*R*)-1l]

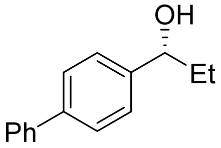

(*R*)-**1l**

$C_{15}H_{16}O$

M = 212.29 g/mol

Prepared according to GP1 from *rac*-1-([1,1'-biphenyl]-4-yl)propan-1-ol (*rac*-**1l**, 43.0 mg, 200 µmol, 1.00 equiv.), $^{n}Bu_3SiH$ (**2h**, 22.0 mg, 110 µmol, 0.550 equiv.). Reaction was stopped after 18 h, and the crude product was purified by flash-column chromatography using *n*-pentane/ethyl acetate (5:1) as eluent. The title compound (*R*)-**1l** was isolated as white solid (19.0 mg, 44.2% yield, 76.4% ee).

M.p.: 61–62°C. $\mathbf{R_f}$ = 0.14 (*n*-pentane/ethyl acetate 4:1). ^{1}**H NMR** (500 MHz, $CDCl_3$): δ/ppm = 7.61–7.55 (m, 4H), 7.46–7.42 (m, 4H), 7.37–7.33 (m, 1H), 4.66 (t, *J* = 6.5 Hz, 1H), 1.92–1.76 (m, 3H), 0.96 (t, *J* = 7.4 Hz, 3H). ^{13}**C NMR** (126 MHz, $CDCl_3$): δ/ppm = 143.8, 141.0, 140.6, 128.9, 127.4, 127.3, 127.2, 126.6, 75.9, 32.0, 10.3. **HRMS** (APCI) for $C_{15}H_{15}O^+$ $[M–H]^+$ calcd *m/z* 211.1117 found 211.1113. **IR** (ATR): $\tilde{\nu}$/cm^{-1} =.3304, 3031, 2956, 2929, 2871, 1596, 1484, 1452, 1406, 1332, 1278, 1192, 1124, 1078, 1002, 900, 831, 759, 726, 688. **Optical rotation**: $[\alpha]_D^{20}$ = +26.4 (*c* 0.9, $CHCl_3$, 76.4% ee). $[\alpha]_D^{20}$ = +26.9 (*c* 0.5, $CHCl_3$, 75% ee) reported for (*R*)-enantiomer.[19] The enantiomeric excess of (*R*)-**1l** was determined by HPLC analysis on a chiral stationary phase (Daicel Chiralcel AD-H column, column temperature 20°C, solvent *n*-heptane:isopropanol = 95:5, flow rate 0.8 mL/min, λ = 250 nm): t_R = 22.9 min for (*S*)-**1l**, t_R = 25.4 min for (*R*)-**1l**.

The analytical data are in accordance with those reported.[19,20]

(*S*)-(1-([1,1'-Biphenyl]-4-yl)propoxy)tributylsilane [(*S*)-3lh]

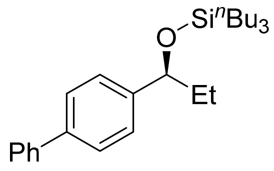

(*S*)-**3lh**

$C_{27}H_{42}OSi$

M = 410.72 g/mol

Prepared according to GP1 from *rac*-1-([1,1'-biphenyl]-4-yl)propan-1-ol (*rac*-**1l**, 43.0 mg, 200 μmol, 1.00 equiv.), $^{n}Bu_3SiH$ (**2h**, 22.0 mg, 110 μmol, 0.550 equiv.). Reaction was stopped after 18 h, and the crude product was purified by flash-column chromatography using *n*-pentane/ethyl acetate (50:1) as eluent. The title compound (*S*)-**3lh** was isolated as colorless oil (42.0 mg, 50.0% yield, 67.8% ee).

R_f = 0.32 (*n*-pentane/ethyl acetate 20:1). **^{1}H NMR** (500 MHz, C_6D_6): δ/ppm = 7.50–7.48 (m, 4H), 7.37 (d, *J* = 8.0 Hz, 2H), 7.23–7.20 (m, 2H), 7.14–7.11 (m, 1H), 4.66 (t, *J* = 6.2 Hz, 1H), 1.92–1.72 (m, 2H), 1.44–1.31 (m, 12H), 0.96 (t, *J* = 7.4 Hz, 3H), 0.91 (t, *J* = 7.1 Hz, 9H), 0.73–0.63 (m, 6H). **^{13}C NMR** (126 MHz, C_6D_6): δ/ppm = 145.0, 141.6, 140.6, 129.0, 127.4, 127.4, 127.3, 126.9, 76.5, 34.1, 27.1, 26.0, 14.3, 14.0, 10.3. **HRMS** (EI) for $C_{25}H_{37}OSi^+$ $[M–C_2H_5]^+$ calcd *m/z* 381.2608 found 381.2610. **IR** (ATR): $\tilde{\nu}/cm^{-1}$ = 2955, 2919, 2869, 1485, 1460, 1375, 1193, 1098, 1078, 1057, 1006, 962, 886, 845, 759, 729, 695. **Optical rotation**: $[\alpha]_D^{20}$ = –38.2 (*c* 1.5, $CHCl_3$, 67.8% ee). The enantiomeric excess of **3lh** was determined after deprotection using the same HPLC setup as for **1l** (Daicel Chiralcel AD-H column, column temperature 20°C, solvent *n*-heptane:isopropanol = 95:5, flow rate 0.8 mL/min, λ = 250 nm): t_R = 22.9 min for (*S*)-**1l**, t_R = 25.4 min for (*R*)-**1l.**

Kinetic resolution data for **1l**

Run	Conversion of *rac*-**1l** [%]	ee of silyl ether (*S*)-**3lh** [%]	ee of unreacted (*R*)-**1l** [%]	Selectivity factor s
1	53.1	67.8	76.4	11.6

(*R*)-1,2-Diphenylethan-1-ol [(*R*)-1m]

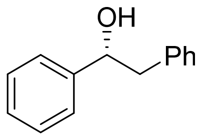

(*R*)-**1m**

$C_{14}H_{14}O$

M = 198.27 g/mol

Prepared according to GP1 from *rac*-1,2-diphenylethan-1-ol (*rac*-**1m**, 80.0 mg, 400 µmol, 1.00 equiv.), ^{n}Bu$_3$SiH (**2h**, 44.0 mg, 220 µmol, 0.550 equiv.). Reaction was stopped after 36 h, and the crude product was purified by flash-column chromatography using *n*-pentane/diethyl ether (4:1) as eluent. The title compound (*R*)-**1m** was isolated as white solid (40.0 mg, 50.0% yield, 54.4% ee).

M.p.: 68–69°C. **R*f*** = 0.38 (cyclohexane/ethyl acetate 4:1). **^{1}H NMR** (400 MHz, $CDCl_3$): δ/ppm = 7.27–7.26 (m, 4H), 7.24–7.18 (m, 3H), 7.17–7.13 (m, 1H), 7.12–7.08 (m, 2H), 4.79 (dd, *J* = 8.3, 5.0 Hz, 1H), 2.97–2.86 (m, 2H), 1.92 (s_{br}, 1H). **13C NMR** (126 MHz, CDCl3): δ/ppm = 144.0, 138.2, 129.6, 128.6, 128.5, 127.7, 126.7, 126.0, 75.5, 46.2. **HRMS** (APCI) for $C_{14}H_{13}O^+$ $[M–H]^+$ calcd *m/z* 197.0961 found 197.0956. **IR** (ATR): $\tilde{\nu}$/cm^{-1} = 3295, 3025, 2921, 2860, 1493, 1450, 1406, 1316, 1271, 1071, 1025, 951, 916, 777, 760, 694. **Optical rotation**: $[\alpha]_D^{20}$ = +10.9 (*c* 1.0, $CHCl_3$, 54.4% ee); $[\alpha]_D^{20}$ = –9.0 (*c* 0.77, $CHCl_3$, 74% ee) reported for (*S*)-enantiomer.[9] The enantiomeric excess of (*R*)-**1m** was determined by HPLC analysis on a chiral stationary phase (Daicel Chiralcel OD-H column, column temperature 20°C, solvent *n*-heptane:isopropanol = 95:5, flow rate 0.8 mL/min, λ = 210 nm): t_R = 20.8 min for (*R*)-**1m**, t_R = 24.7 min for (*S*)-**1m.**

The analytical data are in accordance with those reported. [9]

(*S*)-Tributyl(1,2-diphenylethoxy)silane [(*S*)-3mh]

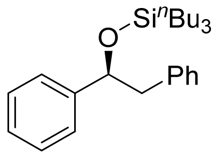

(*S*)-**3mh**

$C_{26}H_{40}OSi$

M = 396.69 g/mol

Prepared according to GP1 from *rac*-1,2-diphenylethan-1-ol (*rac*-**1m**, 80.0 mg, 400 µmol, 1.00 equiv.), ^{n}Bu$_3$SiH (**2h**, 44.0 mg, 220 µmol, 0.550 equiv.). Reaction was stopped after 36 h, and the crude product was purified by flash-column chromatography using *n*-pentane/diethyl ether (50:1) as eluent. The title compound (*S*)-**3mh** was isolated as colorless oil (66.2 mg, 41.8% yield, 70.6% ee).

R*f* = 0.20 (cyclohexane). **^{1}H NMR** (500 MHz, C_6D_6): δ/ppm = 7.29–7.27 (m, 2H), 7.16–7.11 (m, 6H), 7.09–7.07 (m, 2H), 4.83 (dd, *J* = 8.1, 4.6 Hz, 1H), 3.02–2.87 (m, 2H), 1.28–1.20 (m, 12H), 0.87 (t, *J* = 6.9 Hz, 9H), 0.50–0.47 (m, 6H). **^{13}C NMR** (126 MHz, C_6D_6): δ/ppm = 145.7, 139.3, 130.3, 128.4, 128.3, 127.4, 126.5, 126.4, 77.2, 48.4, 27.1, 25.7, 14.1, 14.0. **HRMS** (EI) for $C_{22}H_{31}OSi^+$ $[M–C_4H_9]^+$ calcd *m/z* 339.2139 found 339.2143. **IR** (ATR): $\tilde{\nu}$ /cm^{-1} = 2953, 2918, 2869, 1493, 1453, 1375, 1295, 1194, 1067, 1027, 938, 884, 756, 696. **Optical rotation**: $[\alpha]_D^{20}$ = –18.3 (*c* 0.9, $CHCl_3$, 70.6% ee). The enantiomeric excess of **3mh** was determined after deprotection using the same HPLC setup as for **1m** (Daicel Chiralcel OD-H column, column temperature 20°C, solvent *n*-heptane:isopropanol = 95:5, flow rate 0.8 mL/min, λ = 210 nm): t_R = 20.8 min for (*R*)-**1m**, t_R = 24.7 min for (*S*)-**1m.**

Kinetic resolution data for **1m**

Run	Conversion of *rac*-**1m** [%]	ee of silyl ether (*S*)-**3mh** [%]	ee of unreacted (*R*)-**1m** [%]	Selectivity factor s
1	43.5	70.6	54.4	9.93

(*R*)-1-([1,1'-Biphenyl]-4-yl)-2-methylpropan-1-ol [(*R*)-1n]

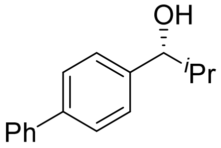

(*R*)-**1n**

$C_{16}H_{18}O$

M = 226.32 g/mol

Prepared according to GP1 from *rac*-1-([1,1'-biphenyl]-4-yl)-2-methylpropan-1-ol (*rac*-**1n**, 46.0 mg, 200 µmol, 1.00 equiv.), $^{n}Bu_3SiH$ (**2h**, 22.0 mg, 110 µmol, 0.550 equiv.). Reaction was stopped after 72 h, and the crude product was purified by flash-column chromatography using *n*-pentane/ethyl acetate (8:1) as eluent. The title compound (*R*)-**1n** was isolated as white solid (22.0 mg, 47.8% yield, 34.0% ee).

M.p.: 62–63°C. $\mathbf{R_f}$ = 0.58 (*n*-pentane/ethyl acetate 5:1). **^{1}H NMR** (500 MHz, $CDCl_3$): δ/ppm = 7.62–7.57 (m, 4H), 7.44 (t, *J* = 7.6 Hz, 2H), 7.40–7.38 (m, 2H), 7.36–7.33 (m, 1H), 4.42 (d, *J* = 6.8 Hz, 1H), 2.01 (dq, *J* = 13.5, 6.7 Hz, 1H), 1.84 (s_{br}, 1H), 1.04 (d, *J* = 6.7 Hz, 3H), 0.85 (d, *J* = 6.8 Hz, 3H). **^{13}C NMR** (126 MHz, $CDCl_3$): δ/ppm = 142.8, 141.0, 140.5, 128.9, 127.4, 127.2, 127.1, 127.1, 79.9, 35.4, 19.2, 18.4. **HRMS** (APCI) for $C_{16}H_{17}O^+$ $[M–H]^+$ calcd *m/z* 225.1274 found 225.1275. **IR** (ATR): $\tilde{\nu}/cm^{-1}$ = 3309, 3026, 2956, 2868, 1656, 1463, 1401, 1317, 1243, 1172, 1106, 1032, 1004, 833, 758, 732, 692. **Optical rotation**: $[\alpha]_D^{20}$ = +12.0 (*c* 1.1, $CHCl_3$, 34.0% ee). Absolute configuration was assigned based on analogy.[21] The enantiomeric excess of (*R*)-**1n** was determined by HPLC analysis on a chiral stationary phase (Daicel Chiralcel AD-H column, column temperature 20°C, solvent *n*-heptane:isopropanol = 95:5, flow rate 0.8 mL/min, λ = 250 nm): t_R = 20.2 min for (*S*)-**1n**, t_R = 24.1 min for (*R*)-**1n**.

(*S*)-(1-([1,1'-Biphenyl]-4-yl)-2-methylpropoxy)tributylsilane [(*S*)-3nh]

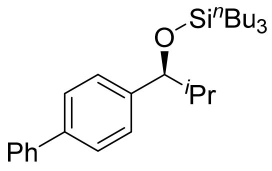

(*S*)-**3nh**

$C_{28}H_{44}OSi$

M = 424.74 g/mol

Prepared according to GP1 from *rac*-1-([1,1'-biphenyl]-4-yl)-2-methylpropan-1-ol (*rac*-**1n**, 46.0 mg, 200 µmol, 1.00 equiv.), $^{n}Bu_3SiH$ (**2h**, 22.0 mg, 110 µmol, 0.550 equiv.). Reaction was stopped after 72 h, and the crude product was purified by flash-column chromatography using *n*-pentane/ethyl acetate (100:1) as eluent. The title compound (*S*)-**3nh** was isolated as colorless oil (38.1 mg, 44.8% yield, 37.4% ee).

R_f = 0.33 (*n*-pentane). **^{1}H NMR** (500 MHz, C_6D_6): δ/ppm = 7.51–7.48 (m, 4H), 7.35 (d, *J* = 8.1 Hz, 2H), 7.22 (t, *J* = 7.6 Hz, 2H), 7.14–7.11 (m, 1H), 4.43 (d, *J* = 6.3 Hz, 1H), 1.98 (dq, *J* = 13.3, 6.7 Hz, 1H), 1.42–1.30 (m, 12H), 1.09 (d, *J* = 6.7 Hz, 3H), 0.92–0.88 (m, 12H), 0.68–0.65 (m, 6H). **^{13}C NMR** (126 MHz, C_6D_6): δ/ppm = 143.9, 141.6, 140.6, 129.0, 127.7, 127.4, 127.4, 127.0, 80.7, 37.1, 27.1, 26.0, 19.2, 18.7, 14.3, 14.0. **HRMS** (EI) for $C_{25}H_{37}OSi^+$ $[M–C_3H_7]^+$ calcd *m/z* 381.2608 found 381.2617. **IR** (ATR): $\tilde{\nu}/cm^{-1}$ = 2954, 2919, 2869, 1599, 1460, 1406, 1378, 1296, 1270, 1193, 1061, 1006, 960, 885, 847, 786, 759, 730, 694. **Optical rotation**: $[\alpha]_D^{20}$ = –18.9 (*c* 1.2, $CHCl_3$, 44.8% ee). The enantiomeric excess of **3nh** was determined after deprotection using the same HPLC setup as for **1n** (Daicel Chiralcel AD-H column, column temperature 20°C, solvent *n*-heptane:isopropanol = 95:5, flow rate 0.8 mL/min, λ = 250 nm): t_R = 20.2 min for (*S*)-**1n**, t_R = 24.1 min for (*R*)-**1n.**

Kinetic resolution data for **1n**

Run	Conversion of *rac*-**1n** [%]	ee of silyl ether (*S*)-**3nh** [%]	ee of unreacted (*R*)-**1n** [%]	Selectivity factor s
1	47.6	37.4	34.0	3.00

(*R*)-1-Mesitylpropan-1-ol [(*R*)-1o]

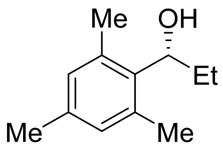

(*R*)-**1o**

$C_{12}H_{18}O$

M = 178.28 g/mol

1st run: Prepared according to GP1 from *rac*-1-mesitylpropan-1-ol (*rac*-**1o**, 36.0 mg, 200 µmol, 1.00 equiv.), $^{n}Bu_3SiH$ (**2h**, 22.0 mg, 110 µmol, 0.550 equiv.). Reaction was stopped after 36 h, and the crude product was purified by flash-column chromatography using *n*-pentane/diethyl ether (9:1) as eluent. The title compound (*R*)-**1o** was isolated as colorless oil and solidified upon standing (16.7 mg, 46.3% yield, 95.0% ee).

2nd run: Prepared according to GP1 from *rac*-1-mesitylpropan-1-ol (*rac*-**1o**, 72.0 mg, 400 µmol, 1.00 equiv.), $^{n}Bu_3SiH$ (**2h**, 44.0 mg, 220 µmol, 0.550 equiv.). Reaction was stopped after 36 h, and the crude product was purified by flash-column chromatography using *n*-pentane/diethyl ether (9:1) as eluent. The title compound (*R*)-**1o** was isolated as colorless oil (26.6 mg, 36.9% yield, >99.5% ee).

M.p.: 44–45°C. $\mathbf{R_f}$ = 0.24 (*n*-pentane/diethyl ether 9:1). ^{1}**H NMR** (400 MHz, C_6D_6): δ/ppm = 6.74 (s, 2H), 4.79–4.75 (m, 1H), 2.33 (s, 6H), 2.14 (s, 3H), 1.97–1.86 (m, 1H), 1.72–1.62 (m, 1H), 1.05 (s_{br}, 1H), 0.89 (t, J = 7.4 Hz, 3H).ppm. ^{13}**C NMR** (126 MHz, C_6D_6): δ/ppm = 137.7, 136.1, 136.0, 130.4, 72.9, 29.3, 20.9, 20.8, 11.3. **HRMS** (APCI) for $C_{12}H_{17}O^{+}$ $[M–H]^{+}$ calcd *m/z* 177.1274 found 177.1270. **IR** (ATR): $\tilde{\nu}$/cm^{-1} = 3220, 2965, 2920, 1609, 1444, 1377, 1338, 1206, 1149, 1078, 1047, 1004, 969, 849, 819, 748. **Optical rotation**: $[\alpha]_D^{20}$ = +43.8 (*c* 0.73, $CHCl_3$, >99.5% ee). Absolute configuration was assigned based on analogy. The enantiomeric excess of (*R*)-**1o** was determined by HPLC analysis on a chiral stationary phase (Daicel Chiralcel AD-H column, column temperature 20°C, solvent *n*-heptane:isopropanol = 95:5, flow rate 0.8 mL/min, λ = 230 nm): t_R = 7.1 min for (*R*)-**1o**, t_R = 8.2 min for (*S*)-**1o**.

(*S*)-Tributyl(1-mesitylpropoxy)silane [(*S*)-3oh]

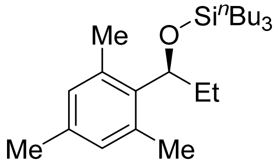

(*S*)-**3oh**

$C_{24}H_{44}OSi$

M = 376.70 g/mol

1st run: Prepared according to GP1 from *rac*-1-mesitylpropan-1-ol (*rac*-**1o**, 36.0 mg, 200 μmol, 1.00 equiv.), ^{n}Bu$_3$SiH (**2h**, 22.0 mg, 110 μmol, 0.550 equiv.). Reaction was stopped after 36 h, and the crude product was purified by flash-column chromatography using *n*-pentane/diethyl ether (50:1) as eluent. The title compound (*S*)-**3oh** was isolated as colorless oil (32.0 mg, 42.5% yield, 91.2% ee).

2nd run: Prepared according to GP1 from *rac*-1-mesitylpropan-1-ol (*rac*-**1o**, 72.0 mg, 400 μmol, 1.00 equiv.), ^{n}Bu$_3$SiH (**2h**, 44.0 mg, 220 μmol, 0.550 equiv.). Reaction was stopped after 36 h, and the crude product was purified by flash-column chromatography using *n*-pentane/diethyl ether (50:1) as eluent. The title compound (*S*)-**3oh** was isolated as colorless oil (75.1 mg, 49.8% yield, 84.2% ee).

R_f = 0.22 (*n*-pentane). **^{1}H NMR** (500 MHz, C_6D_6): δ/ppm = 6.74–6.72 (m, 2H), 5.06 (dd, *J* = 8.5, 5.8 Hz, 1H), 2.65 (s, 3H), 2.26 (s, 3H), 2.13 (s, 3H), 2.09–2.00 (m, 1H), 1.80–1.72 (m, 1H), 1.38–1.27 (m, 12H), 0.98 (t, *J* = 7.4 Hz, 3H), 0.89 (t, *J* = 7.1 Hz, 9H), 0.67–0.63 (m, 6H). **^{13}C NMR** (126 MHz, C_6D_6): δ/ppm = 138.1, 136.0, 73.6, 30.6, 27.2, 25.9, 21.0, 20.8, 14.3, 14.0, 11.5. **HRMS** (EI) for $C_{22}H_{39}OSi^+$ $[M–C_2H_5]^+$ calcd *m/z* 347.2765 found 347.2775. **IR** (ATR): $\tilde{\nu}$/cm^{-1} = 2955, 2919, 2870, 1611, 1459, 1408, 1375, 1295, 1193, 1154, 1058, 1001, 962, 885, 850, 788, 757, 727. **Optical rotation**: $[\alpha]_D^{20}$ = –31.6 (*c* 0.79, $CHCl_3$, 84.6% ee). The enantiomeric excess of **3oh** was determined after deprotection using the same HPLC setup as for **1o** (Daicel Chiralcel AD-H column, column temperature 20°C, solvent *n*-heptane:isopropanol = 95:5, flow rate 0.8 mL/min, λ = 230 nm): t_R = 7.1 min for (*R*)-**1o**, t_R = 8.2 min for (*S*)-**1o**.

Kinetic resolution data for **1o**

Run	Conversion of *rac*-**1o** [%]	ee of silyl ether (*S*)-**3oh** [%]	ee of unreacted (*R*)-**1o**[%]	Selectivity factor s
1	51.0	91.2	95.0	81.4
2	54.2	84.2	>99.5	>50

(*R*)-1-(Naphthalen-2-yl)ethan-1-ol [(*R*)-1p]

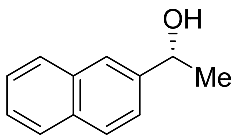

(*R*)-**1p**

$C_{12}H_{12}O$

M = 172.23 g/mol

Prepared according to GP1 from *rac*-1-(naphthalen-2-yl)ethan-1-ol (*rac*-**1p**, 35.0 mg, 200 μmol, 1.00 equiv.), $^{n}Bu_3SiH$ (**2h**, 22.0 mg, 110 μmol, 0.550 equiv.). Reaction was stopped after 18 h, and the crude product was purified by flash-column chromatography using *n*-pentane/diethyl ether (5:1) as eluent. The title compound (*R*)-**1p** was isolated as white solid (14.8 mg, 42.3% yield, 85.0% ee).

M.p.: 73–74°C. $\mathbf{R_f}$ = 0.36 (*n*-pentane/ethyl acetate 5:1). **^{1}H NMR** (500 MHz, $CDCl_3$): δ/ppm = 7.85–7.81 (m, 4H), 7.52–7.45 (m, 3H), 5.07 (d, *J* = 6.3 Hz, 1H), 1.98 (s_{br}, 1H), 1.59 (d, *J* = 6.4 Hz, 3H). **^{13}C NMR** (126 MHz, $CDCl_3$): δ/ppm = 143.4, 133.5, 133.1, 128.4, 128.1, 127.8, 126.3, 125.9, 124.0, 124.0, 70.7, 25.3. **HRMS** (APCI) for $C_{12}H_{11}O^+$ $[M–H]^+$ calcd *m/z* 171.0804 found 171.0802. **IR** (ATR): $\tilde{\nu}/cm^{-1}$ = 3295, 3051, 2970, 2919, 2877, 1598, 1505, 1361, 1321, 1274, 1166, 1122, 1070, 1022, 899, 860, 822, 722, 739, 702. **Optical rotation**: $[\alpha]_D^{20}$ = +42.2 (*c* 0.70, $CHCl_3$, 85.0% ee); $[\alpha]_D^{20}$ = –47 (*c* 0.28, $CHCl_3$, 80% ee) reported for (*S*)-enantiomer.[9] The enantiomeric excess of (*R*)-**1p** was determined by HPLC analysis on a chiral stationary phase (Daicel Chiralcel AD-H column, column temperature 20°C, solvent *n*-heptane:isopropanol = 90:10, flow rate 0.8 mL/min, λ = 210 nm): t_R = 14.5 min for (*S*)-**1p**, t_R = 17.8 min for (*R*)-**1p.**

The analytical data are in accordance with those reported.[9]

(*S*)-Tributyl(1-(naphthalen-2-yl)ethoxy)silane [(*S*)-3ph]

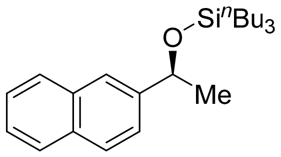

(*S*)-**3ph**

$C_{24}H_{38}OSi$

M = 370.65 g/mol

Prepared according to GP1 from *rac*-1-(naphthalen-2-yl)ethan-1-ol (*rac*-**1p**, 35.0 mg, 200 μmol, 1.00 equiv.), ^{n}Bu$_3$SiH (**2h**, 22.0 mg, 110 μmol, 0.550 equiv.). Reaction was stopped after 18 h, and the crude product was purified by flash-column chromatography using *n*-pentane/diethyl ether (50:1) as eluent. The title compound (*S*)-**3ph** was isolated as colorless oil (39.2 mg, 51.6% yield, 74.0% ee).

$\mathbf{R_f}$ = 0.33 (*n*-pentane). **^{1}H NMR** (500 MHz, C_6D_6): δ/ppm = 7.79 (s, 1H), 7.71 (d, *J* = 8.0 Hz, 1H), 7.67 (d, *J* = 8.5 Hz, 1H), 7.63 (d, *J* = 7.9 Hz, 1H), 7.54–7.51 (m, 1H), 7.29–7.22 (m, 2H), 5.00 (q, *J* = 6.3 Hz, 1H), 1.53 (d, *J* = 6.3 Hz, 3H), 1.44–1.28 (m, 12H), 0.87 (t, *J* = 7.2 Hz, 9H), 0.70–0.66 (m, 6H). **^{13}C NMR** (126 MHz, C_6D_6): δ/ppm = 144.8, 134.0, 133.5, 128.4, 126.3, 125.8, 124.4, 124.2, 71.4, 27.6, 27.0, 26.0, 14.3, 14.0. **HRMS** (EI) for $C_{20}H_{29}OSi^+$ $[M–C_4H_9]^+$ calcd *m/z* 313.1982 found 313.1985. **IR** (ATR): $\tilde{\nu}$/cm^{-1} = 2954, 2919, 2869, 1459, 1374, 1193, 1172, 1078, 1024, 963, 942, 884, 815, 786, 742. **Optical rotation**: $[\alpha]_D^{20}$ = –32.2 (*c* 0.76, $CHCl_3$, 74.0% ee). The enantiomeric excess of **3ph** was determined after deprotection using the same HPLC setup as for **1p** (Daicel Chiralcel AD-H column, column temperature 20°C, solvent *n*-heptane:isopropanol = 90:10, flow rate 0.8 mL/min, λ = 210 nm): t_R = 14.5 min for (*S*)-**1p**, t_R = 17.8 min for (*R*)-**1p.**

Kinetic resolution data for **1p**

Run	Conversion of *rac*-**1p** [%]	ee of silyl ether (*S*)-**3ph** [%]	ee of unreacted (*R*)-**1p** [%]	Selectivity factor s
1	53.5	74.0	85.0	18.5

(*R*)-1-(Naphthalen-1-yl)ethan-1-ol [(*R*)-1q]

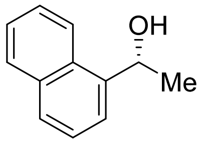

(*R*)-**1q**

$C_{12}H_{12}O$

M = 172.23 g/mol

Prepared according to GP1 from *rac*-1-(naphthalen-1-yl)ethan-1-ol (*rac*-**1q**, 70.0 mg, 400 µmol, 1.00 equiv.), $^{n}Bu_3SiH$ (**2h**, 44.0 mg, 220 µmol, 0.550 equiv.). Reaction was stopped after 36 h, and the crude product was purified by flash-column chromatography using *n*-pentane/diethyl ether (5:1) as eluent. The title compound (*R*)-**1q** was isolated as white solid (29.2 mg, 41.7% yield, 85.8% ee).

M.p.: 63–64°C. $\mathbf{R_f}$ = 0.28 (*n*-pentane/diethyl ether 4:1). **^{1}H NMR** (500 MHz, $CDCl_3$): δ/ppm = 8.13–8.11 (m, 1H), 7.89–7.87 (m, 1H), 7.79 (d, *J* = 8.2 Hz, 1H), 7.69–7.67 (m, 1H), 7.54–7.47 (m, 3H), 5.67 (q, *J* = 6.5 Hz, 1H), 2.00 (s_{br}, 1H), 1.67 (d, *J* = 6.5 Hz, 3H). **^{13}C NMR** (126 MHz, $CDCl_3$): δ/ppm = 141.5, 134.0, 130.4, 129.0, 128.1, 126.2, 125.7, 125.7, 123.3, 122.1, 67.3, 24.5. **HRMS** (APCI) for $C_{12}H_{11}O^{+}$ $[M–H]^{+}$ calcd *m/z* 171.0804 found 171.0800. **IR** (ATR): $\tilde{\nu}/cm^{-1}$ = 3209, 3043, 2971, 1593, 1507, 1367, 1320, 1287, 1226, 1163, 1106, 1066, 1010, 897, 864, 798, 799, 777, 688. **Optical rotation**: $[\alpha]_D^{20}$ = +58.6 (*c* 1.2, $CHCl_3$, 85.8% ee); $[\alpha]_D^{27}$ = +53.3 (*c* 1.16, $CHCl_3$, 98% ee) reported for (*R*)-enantiomer.[22] The enantiomeric excess of (*R*)-**1q** was determined by HPLC analysis on a chiral stationary phase (Daicel Chiralcel OD-H column, column temperature 20°C, solvent *n*-heptane:isopropanol = 90:10, flow rate 0.8 mL/min, λ = 280 nm): t_R = 16.4 min for (*S*)-**1q**, t_R = 25.9 min for (*R*)-**1q**.

The analytical data are in accordance with those reported.[22]

(*S*)-Tributyl(1-(naphthalen-1-yl)ethoxy)silane [(*S*)-3qh]

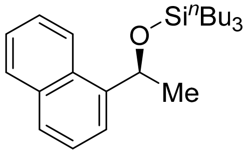

(*S*)-**3qh**

$C_{24}H_{38}OSi$

M = 370.65 g/mol

Prepared according to GP1 from *rac*-1-(naphthalen-1-yl)ethan-1-ol (*rac*-**1q**, 70.0 mg, 400 μmol, 1.00 equiv.), $^{n}Bu_3SiH$ (**2h**, 44.0 mg, 220 μmol, 0.550 equiv.). Reaction was stopped after 36 h, and the crude product was purified by flash-column chromatography using *n*-pentane/diethyl ether (50:1) as eluent. The title compound (*S*)-**3qh** was isolated as colorless oil (79.7 mg, 52.8% yield, 70.4% ee).

R_f = 0.38 (*n*-pentane). **^{1}H NMR** (500 MHz, C_6D_6): δ/ppm = 8.14 (d, *J* = 8.5 Hz, 1H), 7.83 (d, *J* = 7.1 Hz, 1H), 7.67 (d, *J* = 8.1 Hz, 1H), 7.57 (d, *J* = 8.2 Hz, 1H), 7.36–7.31 (m, 2H), 7.28–7.25 (m, 1H), 5.64 (q, *J* = 6.3 Hz, 1H), 1.63 (d, *J* = 6.4 Hz, 3H), 1.40–1.27 (m, 12H), 0.85 (t, *J* = 7.2 Hz, 9H), 0.73–0.62 (m, 6H). **^{13}C NMR** (126 MHz, C_6D_6): δ/ppm = 143.0, 134.5, 130.6, 129.3, 128.3, 126.0, 125.8, 125.5, 123.7, 123.3, 67.0, 27.0, 25.9, 14.2, 14.0. **HRMS** (EI) for $C_{20}H_{29}OSi^+$ $[M–C_4H_9]^+$ calcd *m/z* 313.1982 found 313.1971. **IR** (ATR): $\tilde{\nu}$ /cm^{-1} = 2954, 2919, 2856, 1459, 1374, 1193, 1170, 1115, 1092, 1077, 1022, 947, 883, 796, 773, 730. **Optical rotation**: $[\alpha]_D^{20}$ = –25.5 (*c* 1.08, $CHCl_3$, 70.0% ee). The enantiomeric excess of **3qh** was determined after deprotection using the same HPLC setup as for **1q** (Daicel Chiralcel OD-H column, column temperature 20°C, solvent *n*-heptane:isopropanol = 90:10, flow rate 0.8 mL/min, λ = 280 nm): t_R = 16.4 min for (*S*)-**1q**, t_R = 25.9 min for (*R*)-**1q**.

Kinetic resolution data for **1q**

Run	Conversion of *rac*-**1q** [%]	ee of silyl ether (*S*)-**3qh** [%]	ee of unreacted (*R*)-**1q** [%]	Selectivity factor s
1	52.8	70.4	85.8	15.5

(*R*)-2,3-Dihydro-1*H*-inden-1-ol [(*R*)-1r]

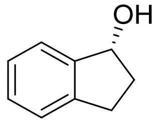

(*R*)-**1r**

$C_9H_{10}O$

M = 134.18 g/mol

1st run: Prepared according to GP1 from *rac*-2,3-dihydro-1*H*-inden-1-ol (*rac*-**1r**, 27.0 mg, 200 µmol, 1.00 equiv.), $^{n}Bu_3SiH$ (**2h**, 22.0 mg, 110 µmol, 0.550 equiv.). Reaction was stopped after 18 h, and the crude product was purified by flash-column chromatography using *n*-pentane/diethyl ethyl (4:1) as eluent. The title compound (*R*)-**1r** was isolated as white solid (11.9 mg, 44.2% yield, 88.0% ee).

2nd run: Prepared according to GP1 from *rac*-2,3-dihydro-1*H*-inden-1-ol (*rac*-**1r**, 54.0 mg, 400 µmol, 1.00 equiv.), $^{n}Bu_3SiH$ (**2h**, 48.0 mg, 240 µmol, 0.600 equiv.). Reaction was stopped after 18 h, and the crude product was purified by flash-column chromatography using *n*-pentane/diethyl ethyl (4:1) as eluent. The title compound (*R*)-**1r** was isolated as white solid (20.6 mg, 38.1% yield, 97.7% ee).

M.p.: 51–52°C. **R_f** = 0.12 (*n*-pentane/diethyl ethyl 4:1). **^{1}H NMR** (500 MHz, $CDCl_3$): δ/ppm = 7.43–7.40 (m, 1H), 7.27–7.23 (m, 3H), 5.25 (t, *J* = 6.0 Hz, 1H), 3.06 (ddd, *J* = 15.9, 8.5, 4.8 Hz, 1H), 2.85–2.79 (m, 1H), 2.53–2.46 (m, 1H), 1.98–1.92 (m, 1H), 1.76 (s_{br}, 1H). **^{13}C NMR** (126 MHz, $CDCl_3$): δ/ppm = 145.1, 143.5, 128.5, 126.9, 125.0, 124.3, 76.6, 36.1, 29.9. **HRMS** (APCI) for $C_9H_9O^+$ $[M–H]^+$ calcd *m/z* 133.0648 found 133.0647. **IR** (ATR): $\tilde{\nu}$ /cm^{-1} = 3206, 3068, 2973, 2933, 2862, 1474, 1452, 1418, 1323, 1213, 1161, 1092, 1052, 973, 759, 737. **Optical rotation**: $[\alpha]_D^{20}$ = –35.8 (*c* 1.1, $CHCl_3$, 97.7% ee); $[\alpha]_D^{27}$ = –29.1 (*c* 1.1, $CHCl_3$, 98% ee) reported for (*R*)-enantiomer.[10] The enantiomeric excess of (*R*)-**1r** was determined by HPLC analysis on a chiral stationary phase (Daicel Chiralcel OD-H column, column temperature 20°C, solvent *n*-heptane:isopropanol = 96:4, flow rate 0.8 mL/min, λ = 210 nm): t_R = 28.6 min for (*S*)-**1r**, t_R = 31.8 min for (*R*)-**1r**.

The analytical data are in accordance with those reported.[10]

(*S*)-Tributyl((2,3-dihydro-1*H*-inden-1-yl)oxy)silane [(*S*)-3rh]

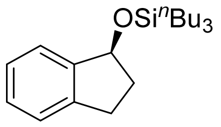

(*S*)-**3rh**

$C_{21}H_{36}OSi$

M = 332.60 g/mol

1st run: Prepared according to GP1 from *rac*-2,3-dihydro-1*H*-inden-1-ol (*rac*-**1r**, 27.0 mg, 200 µmol, 1.00 equiv.), $^{n}Bu_3SiH$ (**2h**, 22.0 mg, 110 µmol, 0.550 equiv.). Reaction was stopped after 18 h, and the crude product was purified by flash-column chromatography using *n*-pentane/diethyl ethyl (100:1) as eluent. The title compound (*S*)-**3rh** was isolated as colorless oil (31.4 mg, 47.2% yield, 76.4% ee).

2nd run: Prepared according to GP1 from *rac*-2,3-dihydro-1*H*-inden-1-ol (*rac*-**1r**, 54.0 mg, 400 µmol, 1.00 equiv.), $^{n}Bu_3SiH$ (**2h**, 48.0 mg, 240 µmol, 0.600 equiv.). Reaction was stopped after 18 h, and the crude product was purified by flash-column chromatography using *n*-pentane/diethyl ethyl (100:1) as eluent. The title compound (*S*)-**3rh** was isolated as colorless oil (63.4 mg, 47.7% yield, 71.4% ee).

$\mathbf{R}_f$ = 0.10 (*n*-pentane). **^{1}H NMR** (500 MHz, C_6D_6): δ/ppm = 7.51 (d, *J* = 7.3 Hz, 1H), 7.18–7.17 (m, 1H), 7.14–7.11 (m, 1H), 7.09 (d, *J* = 7.2 Hz, 1H), 5.20 (t, *J* = 6.6 Hz, 1H), 2.84–2.79 (m, 1H), 2.58-2.51 (m, 1H), 2.23–2.17 (m, 1H), 1.98–1.91 (m, 1H), 1.52–1.37 (m, 12H), 0.95 (t, *J* = 7.2 Hz, 9H), 0.77–0.74 (m, 6H). **^{13}C NMR** (126 MHz, C_6D_6): δ/ppm = 146.2, 142.9, 128.0, 126.8, 124.9, 124.6, 76.8, 37.1, 30.0, 27.1, 26.0, 14.5, 14.1. **HRMS** (EI) for $C_{17}H_{27}OSi^+$ $[M–C_4H_9]^+$ calcd *m/z* 275.1826 found 275.1829. **IR** (ATR): $\tilde{\nu}$/cm^{-1} = 2954, 2919, 2854, 1606, 1459, 1408, 1353, 1192, 1108, 1072, 1029, 984, 885, 787, 739, 713. **Optical rotation** was measured as the deprotected and purified alcohol: $[\alpha]_D^{20}$ = +19.5 (*c* 0.87, $CHCl_3$, 71.4% ee). The enantiomeric excess of **3rh** was determined after deprotection using the same HPLC setup as for **1r** (Daicel Chiralcel OD-H column, column temperature 20°C, solvent *n*-heptane:isopropanol = 96:4, flow rate 0.8 mL/min, λ = 210 nm): t_R = 28.6 min for (*S*)-**1r**, t_R = 31.8 min for (*R*)-**1r**.

Kinetic resolution data for **1r**

Run	Conversion of *rac*-**1r** [%]	ee of silyl ether (*S*)-**3rh** [%]	ee of unreacted (*R*)-**1r** [%]	Selectivity factor s
1	53.5	76.4	88.0	21.4
2	57.8	71.4	97.7	25.5

(*R*)-1,2,3,4-Tetrahydronaphthalen-1-ol [(*R*)-1s]

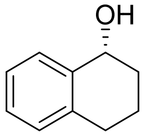

(*R*)-**1s**

$C_{10}H_{12}O$

M = 148.21 g/mol

1st run: Prepared according to GP1 from *rac*-1,2,3,4-tetrahydronaphthalen-1-ol (*rac*-**1s**, 30.0 mg, 200 µmol, 1.00 equiv.), $^{n}Bu_3SiH$ (**2h**, 22.0 mg, 110 µmol, 0.550 equiv.). Reaction was stopped after 18 h, and the crude product was purified by flash-column chromatography using *n*-pentane/diethyl ethyl (4:1) as eluent. The title compound (*R*)-**1s** was isolated as colorless oil (11.8 mg, 39.3% yield, 94.6% ee).

2nd run: Prepared according to GP1 from *rac*-1,2,3,4-tetrahydronaphthalen-1-ol (*rac*-**1s**, 60.0 mg, 400 µmol, 1.00 equiv.), $^{n}Bu_3SiH$ (**2h**, 44.0 mg, 220 µmol, 0.550 equiv.). Reaction was stopped after 18 h, and the crude product was purified by flash-column chromatography using *n*-pentane/diethyl ethyl (4:1) as eluent. The title compound (*R*)-**1s** was isolated as colorless oil (24.1 mg, 40.2% yield, 94.8% ee).

$\mathbf{R}_f$ = 0.15 (*n*-pentane/diethyl ethyl 4:1). **^{1}H NMR** (500 MHz, C_6D_6): δ/ppm = 7.38–7.36 (m, 1H), 7.10–7.03 (m, 2H), 6.91–6.89 (m, 1H), 4.51 (t, *J* = 5.2 Hz, 1H), 2.55–2.49 (m, 1H), 2.44–2.38 (m, 1H), 1.76–1.67 (m, 1H), 1.66–1.62 (m, 2H), 1.45–1.37 (m, 1H), 1.30 (s_{br}, 1H). **^{13}C NMR** (126 MHz, C_6D_6): δ/ppm = 139.8, 137.1, 129.0, 129.0, 127.5, 126.3, 68.1, 32.7, 29.5, 19.3. **HRMS** (APCI) for $C_{10}H_{11}O^+$ $[M–H]^+$ calcd *m/z* 147.0804 found 147.0805. **IR** (ATR): $\tilde{\nu}/cm^{-1}$ = 3308, 3018, 2930, 2861, 1488, 1452, 1338, 1270, 1202, 1152, 1064, 1036, 999, 961, 910, 843, 770, 735. **Optical rotation**: $[\alpha]_D^{20}$ = –30.1 (*c* 0.55, $CHCl_3$, 94.8% ee); $[\alpha]_D^{20}$ = –28.6 (*c* 0.88, $CHCl_3$, 94.4% ee) reported for (*R*)-enantiomer.[10] The enantiomeric excess of (*R*)-**1s** was determined by HPLC analysis on a chiral stationary phase (Daicel Chiralcel OD-H column, column temperature 20°C, solvent *n*-heptane:isopropanol = 98:2, flow rate 0.8 mL/min, λ = 210 nm): t_R = 19.0 min for (*S*)-**1s**, t_R = 20.6 min for (*R*)-**1s.**

The analytical data are in accordance with those reported.[10]

(*S*)-Tributyl((1,2,3,4-tetrahydronaphthalen-1-yl)oxy)silane [(*S*)-3sh]

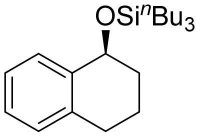

(*S*)-**3sh**

$C_{22}H_{38}OSi$

M = 346.63 g/mol

1st run: Prepared according to GP1 from *rac*-1,2,3,4-tetrahydronaphthalen-1-ol (*rac*-**1s**, 30.0 mg, 200 µmol, 1.00 equiv.), ^{n}Bu$_3$SiH (**2h**, 22.0 mg, 110 µmol, 0.550 equiv.). Reaction was stopped after 18 h, and the crude product was purified by flash-column chromatography using *n*-pentane/diethyl ethyl (50:1) as eluent. The title compound (*S*)-**3sh** was isolated as colorless oil (35.3 mg, 50.9% yield, 82.4% ee).

2nd run: Prepared according to GP1 from *rac*-1,2,3,4-tetrahydronaphthalen-1-ol (*rac*-**1s**, 60.0 mg, 400 µmol, 1.00 equiv.), ^{n}Bu$_3$SiH (**2h**, 44.0 mg, 220 µmol, 0.550 equiv.). Reaction was stopped after 18 h, and the crude product was purified by flash-column chromatography using *n*-pentane/diethyl ethyl (4:1) as eluent. The title compound (*S*)-**3sh** was isolated as colorless oil (72.8 mg, 51.6% yield, 83.4% ee).

R_f = 0.15 (*n*-pentane). **^{1}H NMR** (500 MHz, C_6D_6): δ/ppm = 7.58 (d, *J* = 7.7 Hz, 1H), 7.20–7.16 (m, 1H), 7.10–7.06 (m, 1H), 6.95 (d, *J* = 7.5 Hz, 1H), 4.83 (t, *J* = 5.6 Hz, 1H), 2.63 (dt, *J* = 16.2, 6.4 Hz, 1H), 2.50 (dt, *J* = 13.4, 6.4 Hz, 1H), 1.97–1.89 (m, 1H), 1.86–1.82 (m, 2H), 1.58–1.52 (m, 1H), 1.51–1.36 (m, 12H), 0.94 (t, *J* = 7.2 Hz, 9H), 0.78–0.74 (m, 6H). **^{13}C NMR** (126 MHz, C_6D_6): δ/ppm = 140.2, 137.0, 129.0, 128.6, 127.3, 126.0, 69.6, 33.4, 29.3, 27.1, 26.1, 19.6, 14.7, 14.1. **HRMS** (EI) for $C_{18}H_{29}OSi^+$ $[M–C_4H_9]^+$ calcd *m/z* 289.1982 found 289.1989. **IR** (ATR): $\tilde{\nu}$/cm^{-1} = 2953, 2919, 1869, 1856, 1455, 1408, 1375, 1344, 1295, 1193, 1121, 1074, 1021, 986, 883, 786, 755, 736, 716. **Optical rotation**: $[\alpha]_D^{20}$ = +11.9 (*c* 1.20, $CHCl_3$, 83.4% ee). The enantiomeric excess of **3sh** was determined after deprotection using the same HPLC setup as for **1s** (Daicel Chiralcel OD-H column, column temperature 20°C, solvent *n*-heptane:isopropanol = 98:2, flow rate 0.8 mL/min, λ = 210 nm): t_R = 19.0 min for (*S*)-**1s**, t_R = 20.6 min for (*R*)-**1s.**

Kinetic resolution data for **1s**

Run	Conversion of *rac*-**1s** [%]	ee of silyl ether (*S*)-**3sh** [%]	ee of unreacted (*R*)-**1s** [%]	Selectivity factor s
1	53.4	82.4	94.6	37.6
2	53.2	83.4	94.8	40.1

(*R*)-6,7,8,9-Tetrahydro-5*H*-benzo[7]annulen-5-ol [(*R*)-1t]

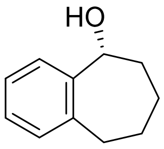

(*R*)-**1t**

$C_{11}H_{14}O$

M = 162.23 g/mol

Prepared according to GP1 from *rac*-6,7,8,9-tetrahydro-5*H*-benzo[7]annulen-5-ol (*rac*-**1t**, 65.0 mg, 400 µmol, 1.00 equiv.), $^{n}Bu_3SiH$ (**2h**, 52.0 mg, 260 µmol, 0.650 equiv.). Reaction was stopped after 12 h, and the crude product was purified by flash-column chromatography using *n*-pentane/diethyl ether (5:1) as eluent. The title compound (*R*)-**1t** was isolated as white solid (24.4 mg, 37.5% yield, 87.4% ee).

M.p.: 103–104°C. R_f = 0.36 (*n*-pentane/diethyl ether 4:1). **^{1}H NMR** (500 MHz, $CDCl_3$): δ/ppm = 7.43 (d, *J* = 7.5 Hz, 1H), 7.22–7.19 (m, 1H), 7.17–7.14 (m, 1H), 7.09 (d, *J* = 7.3 Hz, 1H), 4.94–4.92 (m, 1H), 2.92 (dd, *J* = 14.1, 8.4 Hz, 1H), 2.74–2.69 (m, 1H), 2.08–2.03 (m, 1H), 1.97–1.94 (m, 1H), 1.85–1.74 (m, 4H), 1.50–1.45 (m, 1H). **^{13}C NMR** (126 MHz, $CDCl_3$): δ/ppm = 144.4, 141.0, 129.6, 127.1, 126.2, 124.7, 74.2, 36.7, 35.9, 27.9, 27.7. **HRMS** (APCI) for $C_{11}H_{13}O^+$ $[M–H]^+$ calcd *m/z* 161.0961 found 161.0960. **IR** (ATR): $\tilde{\nu}$/cm^{-1} = 3271, 2923, 2848, 1477, 1447, 1339, 1301, 1197, 1103, 1040, 1014, 938, 760, 734. **Optical rotation**: $[\alpha]_D^{20}$ = +30.6 (*c* 0.95, $CHCl_3$, 87.4% ee); $[\alpha]_D^{20}$ = +31.5 (*c* 0.99, $CHCl_3$, 99.6% ee) reported for (*R*)-enantiomer.[10] The enantiomeric excess of (*R*)-**1t** was determined by HPLC analysis on a chiral stationary phase (Daicel Chiralcel OD-H column, column temperature 20°C, solvent *n*-heptane:isopropanol = 98:2, flow rate 0.8 mL/min, λ = 210 nm): t_R = 27.3 min for (*R*)-**1t**, t_R = 29.9 min for (*S*)-**1t**.

The analytical data are in accordance with those reported.[10]

(*S*)-Tributyl((6,7,8,9-tetrahydro-5*H*-benzo[7]annulen-5-yl)oxy)silane [(*S*)-3th]

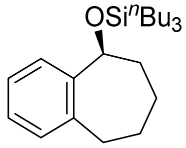

(*S*)-**3th**

$C_{23}H_{40}OSi$

M = 360.66 g/mol

Prepared according to GP1 from *rac*-6,7,8,9-tetrahydro-5*H*-benzo[7]annulen-5-ol (*rac*-**1t**, 65.0 mg, 400 μmol, 1.00 equiv.), $^{n}Bu_3SiH$ (**2h**, 52.0 mg, 260 μmol, 0.650 equiv.). Reaction was stopped after 12 h, and the crude product was purified by flash-column chromatography using *n*-pentane/diethyl ether (50:1) as eluent. The title compound (*S*)-**3th** was isolated as colorless oil (78.3 mg, 54.3% yield, 64.8% ee).

$\mathbf{R_f}$ = 0.44 (*n*-pentane). **^{1}H NMR** (500 MHz, C_6D_6): δ/ppm = 7.53 (m, 1H), 7.14–7.12 (m, 1H), 7.07–7.04 (m, 1H), 6.99 (d, *J* = 7.3 Hz, 1H), 4.94–4.92 (m, 1H), 3.02 (m, 1H), 2.11–2.06 (m, 1H), 1.94–1.85 (m, 1H), 1.94–1.82 (m, 2H), 1.69–1.62 (m, 1H), 1.54–1.48 (m, 2H), 1.45–1.31 (m, 12H), 0.91 (t, *J* = 7.1 Hz, 9H), 0.71–0.68 (m, 6H). **^{13}C NMR** (126 MHz, C_6D_6): δ/ppm = 145.2, 141.5, 129.8, 127.3, 126.4, 126.2, 75.4, 37.7, 36.1, 28.4, 27.6, 27.1, 26.0, 14.3, 14.0. **HRMS** (EI) for $C_{19}H_{31}OSi^+$ $[M–C_4H_9]^+$ calcd *m/z* 303.2139 found 303.2140. **IR** (ATR): $\tilde{\nu}/cm^{-1}$ = 2954, 2918, 2869, 2853, 1453, 1375, 1194, 1108, 1075, 1039, 1010, 962, 936, 885, 786, 746. **Optical rotation**: $[\alpha]_D^{20}$ = –20.8 (*c* 1.1, $CHCl_3$, 64.8% ee). The enantiomeric excess of **3th** was determined after deprotection using the same HPLC setup as for **1t** (Daicel Chiralcel OD-H column, column temperature 20°C, solvent *n*-heptane:isopropanol = 98:2, flow rate 0.8 mL/min, λ = 210 nm): t_R = 27.3 min for (*R*)-**1t**, t_R = 29.9 min for (*S*)-**1t**.

Kinetic resolution data for **1t**

Run	Conversion of *rac*-**1t** [%]	ee of silyl ether (*S*)-**3th** [%]	ee of unreacted (*R*)-**1t**[%]	Selectivity factor s
1	57.4	64.8	87.4	12.9

(*R*)-Chroman-4-ol [(*R*)-1u]

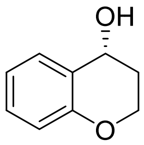

(*R*)-**1u**

$C_9H_{10}O_2$

M = 150.18 g/mol

Prepared according to GP1 from *rac*-chroman-4-ol (*rac*-**1u**, 60.0 mg, 400 µmol, 1.00 equiv.), $^{n}Bu_3SiH$ (**2h**, 52.0 mg, 260 µmol, 0.650 equiv.). Reaction was stopped after 36 h, and the crude product was purified by flash-column chromatography using *n*-pentane/diethyl ethyl (4:1) as eluent. The title compound (*R*)-**1u** was isolated as colorless oil (18.4 mg, 30.7% yield, >99.9% ee).

$\mathbf{R_f}$ = 0.23 (*n*-pentane/diethyl ethyl 4:1). **^{1}H NMR** (500 MHz, $CDCl_3$): δ/ppm = 7.31–7.29 (m, 1H), 7.22–7.18 (m, 1H), 6.93–6.90 (m, 1H), 6.85–6.83 (m, 1H), 4.77 (t, *J* = 3.7 Hz, 1H), 4.27–4.24 (m, 2H), 2.16–2.08 (m, 1H), 2.05–1.99 (m, 2H). **^{13}C NMR** (126 MHz, $CDCl_3$): δ/ppm = 154.7, 129.8, 129.8, 124.4, 120.7, 117.2, 63.4, 62.0, 30.9. **HRMS** (APCI) for $C_9H_9O_2^+$ $[M–H]^+$ calcd *m/z* 149.0597 found 149.0593. **IR** (ATR): $\tilde{\nu}/cm^{-1}$ = 3340, 2956, 2880, 1582, 1486, 1453, 1265, 1219, 1116, 1059, 1014, 971, 883, 831, 751, 718. **Optical rotation**: $[\alpha]_D^{20}$ = +64.6 (*c* 1.5, $CHCl_3$, >99.9% % ee); $[\alpha]_D^{20}$ = +65 (*c* 1.0, $CHCl_3$, >99% ee) reported for (*R*)-enantiomer.[15] The enantiomeric excess of (*R*)-**1u** was determined by HPLC analysis on a chiral stationary phase (Daicel Chiralcel OD-H column, column temperature 20°C, solvent *n*-heptane:isopropanol = 95:5, flow rate 0.8 mL/min, λ = 280 nm): t_R = 16.8 min for (*S*)-**1u**, t_R = 19.3 min for (*R*)-**1u**.

The analytical data are in accordance with those reported.[15]

(*S*)-Tributyl(chroman-4-yloxy)silane [(*S*)-3uh]

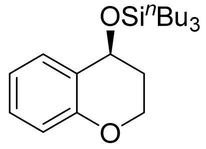

(*S*)-**3uh**

$C_{21}H_{36}O_2Si$

M = 348.60 g/mol

Prepared according to GP1 from *rac*-chroman-4-ol (*rac*-**1u**, 60.0 mg, 400 µmol, 1.00 equiv.), ^{n}Bu$_3$SiH (**2h**, 52.0 mg, 260 µmol, 0.650 equiv.). Reaction was stopped after 36 h, and the crude product was purified by flash-column chromatography using *n*-pentane/diethyl ethyl (50:1) as eluent. The title compound (*S*)-**3uh** was isolated as colorless oil (77.1 mg, 55.3% yield, 67.0% ee).

R_f = 0.57(*n*-pentane/diethyl ethyl 20:1). **^{1}H NMR** (500 MHz, C_6D_6): δ/ppm = 7.35–7.33 (m, 1H), 7.06–7.03 (m, 1H), 7.00–6.98 (m, 1H), 6.86–6.83 (m, 1H), 4.66 (t, *J* = 4.3 Hz, 1H), 4.30–4.25 (m, 1H), 3.95–3.90 (m, 1H), 1.74–1.71 (m, 2H), 1.43–1.33 (m, 12H), 0.92 (t, *J* = 7.1 Hz, 9H), 0.70–0.67 (m, 6H). **^{13}C NMR** (126 MHz, C_6D_6): δ/ppm = 155.3, 130.0, 129.5, 125.4, 120.2, 117.4, 64.3, 62.2, 32.3, 27.1, 25.9, 14.5, 14.0. **HRMS** (EI) for $C_{17}H_{27}O_2Si^+$ $[M–C_4H_9]^+$ calcd *m/z* 291.1775 found 291.1785. **IR** (ATR): $\tilde{\nu}$/cm^{-1} = 2955, 2920, 2869, 1711, 1487, 1454, 1267, 1225, 1194, 1117, 1069, 1037, 1002, 962, 920, 884, 808, 750, 726. **Optical rotation**: $[\alpha]_D^{20}$ = –21.0 (*c* 1.0, $CHCl_3$, 67.0% ee). The enantiomeric excess of **3uh** was determined after deprotection using the same HPLC setup as for **1u** (Daicel Chiralcel OD-H column, column temperature 20°C, solvent *n*-heptane:isopropanol = 95:5, flow rate 0.8 mL/min, λ = 280 nm): t_R = 16.8 min for (*S*)-**1u**, t_R = 19.3 min for (*R*)-**1u**.

Kinetic resolution data for **1u**

Run	Conversion of *rac*-**1u** [%]	ee of silyl ether (*S*)-**3uh** [%]	ee of unreacted (*R*)-**1u** [%]	Selectivity factor s
1	59.9	67.0	>99.9	35.3

(*R*)-Thiochroman-4-ol [(*R*)-1v]

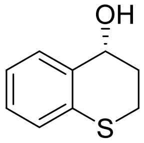

(*R*)-**1v**

$C_9H_{10}OS$

M = 166.24 g/mol

Prepared according to GP1 from *rac*-thiochroman-4-ol (*rac*-**1v**, 67.0 mg, 400 μmol, 1.00 equiv.), $^{n}Bu_3SiH$ (**2h**, 52.0 mg, 260 μmol, 0.650 equiv.). Reaction was stopped after 36 h, and the crude product was purified by flash-column chromatography using *n*-pentane/diethyl ethyl (4:1) as eluent. The title compound (*R*)-**1v** was isolated as white solid (23.0 mg, 34.3% yield, 99.7% ee).

M.p.: 71–73°C. R_f = 0.28 (*n*-pentane/diethyl ethyl 4:1). **^{1}H NMR** (400 MHz, $CDCl_3$): δ/ppm = 7.32–7.31 (m, 1H), 7.18–7.12 (m, 2H), 7.08–7.04 (m, 1H), 4.81 (dd, *J* = 4.8, 3.0 Hz, 1H), 3.32 (td, *J* = 12.3, 3.0 Hz, 1H), 2.86 (ddd, *J* = 12.6, 5.6, 3.6 Hz, 1H), 2.39–2.32 (m, 1H), 2.10–2.02 (m, 1H), 1.72 (s_{br}, 1H). **^{13}C NMR** (126 MHz, $CDCl_3$): δ/ppm = 134.8, 133.4, 130.5, 128.6, 126.9, 124.4, 66.70, 30.2, 21.7. **HRMS** (APCI) for $C_9H_9OS^+$ $[M–H]^+$ calcd *m/z* 165.0369 found 165.0363. **IR** (ATR): $\tilde{\nu}$/cm^{-1} = 3265, 3167, 3060, 2933, 2848, 1587, 1430, 1349, 1279, 1206, 1064, 1024, 958, 839, 786, 741, 725. **Optical rotation**: $[\alpha]_D^{20}$ = +134.9 (*c* 1.1, $CHCl_3$, 99.7% ee); $[\alpha]_D^{20}$ = +137.3 (*c* 1.0, $CHCl_3$, 99.7% ee) reported for (*R*)-enantiomer.[23] The enantiomeric excess of (*R*)-**1v** was determined by HPLC analysis on a chiral stationary phase (Daicel Chiralcel OD-H column, column temperature 20°C, solvent *n*-heptane:isopropanol = 95:5, flow rate 0.8 mL/min, λ = 210 nm): t_R = 20.6 min for (*S*)-**1v**, t_R = 25.4 min for (*R*)-**1v**.

The analytical data are in accordance with those reported.[23]

(S)-Tributyl(thiochroman-4-yloxy)silane [(S)-3vh]

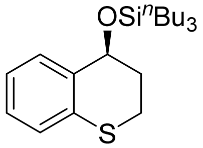

(*S*)-**3vh**

$C_{21}H_{36}OSSi$

M = 364.66 g/mol

Prepared according to GP1 from *rac*-thiochroman-4-ol (*rac*-**1v**, 67.0 mg, 400 µmol, 1.00 equiv.), $^{n}Bu_3SiH$ (**2h**, 52.0 mg, 260 µmol, 0.650 equiv.). Reaction was stopped after 36 h, and the crude product was purified by flash-column chromatography using *n*-pentane/diethyl ethyl (50:1) as eluent. The title compound (*S*)-**3vh** was isolated as colorless oil (84.0 mg, 57.1% yield, 67.0% ee).

$\mathbf{R_f}$ = 0.67(*n*-pentane/diethyl ethyl 20:1). ^{1}**H NMR** (500 MHz, C_6D_6): δ/ppm = 7.32–7.30 (m, 1H), 7.16–7.13 (m, 1H), 6.93–6.89 (m, 2H), 4.67 (dd, *J* = 6.0, 2.6 Hz, 1H), 3.26–3.21 (m, 1H), 2.49 (ddd, *J* = 12.0, 6.0, 4.2 Hz, 1H), 1.98–1.82(m, 1H), 1.80–1.74 (m, 1H), 1.39–1.31 (m, 12H), 0.93 (t, *J* = 7.1 Hz, 9H), 0.66–0.63 (m, 6H). ^{13}**C NMR** (126 MHz, C_6D_6): δ/ppm = 136.3, 134.1, 129.9, 127.0, 123.9, 68.1, 30.2, 27.0, 25.9, 22.0, 14.5, 14.0. **HRMS** (EI) for $C_{17}H_{27}OSSi^+$ $[M–C_4H_9]^+$ calcd *m/z* 307.1546 found 307.1550. **IR** (ATR): $\tilde{\nu}$ /cm^{-1} = 2954, 2918, 2853, 1462, 1440, 1408, 1196, 1174, 1124, 1072, 1044, 965, 885, 790, 744, 726. **Optical rotation**: $[\alpha]_D^{20}$ = –38.4 (*c* 2.2, $CHCl_3$, 67% ee). The enantiomeric excess of **3vh** was determined after deprotection using the same HPLC setup as for **1v** (Daicel Chiralcel OD-H column, column temperature 20°C, solvent *n*-heptane:isopropanol = 95:5, flow rate 0.8 mL/min, λ = 210 nm): t_R = 20.6 min for (*S*)-**1v**, t_R = 25.4 min for (*R*)-**1v**.

Kinetic resolution data for **1v**

Run	Conversion of *rac*-**1v** [%]	ee of silyl ether (*S*)-**3vh** [%]	ee of unreacted (*R*)-**1v** [%]	Selectivity factor s
1	59.8	67.0	99.7	30.5

tert-Butyl (R)-4-hydroxy-3,4-dihydroquinoline-1(2H)-carboxylate [(R)-1w]

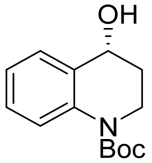

(*R*)-**1w**

$C_{14}H_{19}NO_3$

M = 249.31 g/mol

Prepared according to GP1 from *tert*-butyl *rac*-4-hydroxy-3,4-dihydroquinoline-1(2*H*)-carboxylate (*rac*-**1w**, 100.0 mg, 400 μmol, 1.00 equiv.), $^{n}Bu_3SiH$ (**2h**, 44.0 mg, 220 μmol, 0.550 equiv.). Reaction was stopped after 36 h, and the crude product was purified by flash-column chromatography using *n*-pentane/diethyl ethyl (1:1) as eluent. The title compound (*R*)-**1w** was isolated as white solid (46.6 mg, 46.6% yield, 86.2% ee).

M.p.: 102–103°C. $\mathbf{R_f}$ = 0.34 (*n*-pentane/diethyl ethyl 1:1). **^{1}H NMR** (400 MHz, $CDCl_3$): δ/ppm = 7.73 (d, *J* = 8.3 Hz, 1H), 7.34–7.32 (m, 1H), 7.23–7.18 (m, 1H), 7.05–7.01 (m, 1H), 4.64 (t, *J* = 4.7 Hz, 1H), 3.96 (ddd, *J* = 13.0, 6.0, 4.5 Hz, 1H), 3.57–3.50 (m, 1H), 2.66 (s_{br}, 1H), 2.04–1.88 (m, 2H), 1.51 (s, 9H). **^{13}C NMR** (101 MHz, $CDCl_3$): δ/ppm = 153.7, 138.0, 130.7, 128.3, 128.1, 123.8, 123.6, 81.3, 66.0, 40.6, 32.1, 28.5. **HRMS** (APCI) for $C_{14}H_{18}NO_2^+$ $[M–OH]^+$ calcd *m/z* 232.1332 found 232.1329. **HRMS** (APCI) for $C_{10}H_{10}NO_2^+$ $[M–C_4H_9O]^+$ calcd *m/z* 176.0706 found 176.0701. **IR** (ATR): $\tilde{\nu}$/cm^{-1} = 3316, 2979, 1685, 1606, 1577, 1488, 1362, 1347, 1327, 1246, 1224, 1160, 1118, 1048, 1014, 953, 924, 856, 797, 751, 682. **Optical rotation**: $[\alpha]_D^{20}$ = +27.6 (*c* 1.9, $CHCl_3$, 86.2% ee); $[\alpha]_D^{25}$ = –24.1 (*c* 1.0, $CHCl_3$, 94% ee) reported for (*S*)-enantiomer.[24] The enantiomeric excess of (*R*)-**1w** was determined by HPLC analysis on a chiral stationary phase (Daicel Chiralcel OD-H column, column temperature 20°C, solvent *n*-heptane:isopropanol = 97:3, flow rate 0.8 mL/min, λ = 250 nm): t_R = 25.1 min for (*S*)-**1w**, t_R = 27.4 min for (*R*)-**1w**.

The analytical data are in accordance with those reported.[24]

tert-Butyl (S)-4-((tributylsilyl)oxy)-3,4-dihydroquinoline-1(2H)-carboxylate [(S)-3wh]

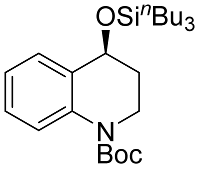

(S)-**3wh**

$C_{26}H_{45}NO_3Si$

M = 447.74

Prepared according to GP1 from *tert*-butyl *rac*-4-hydroxy-3,4-dihydroquinoline-1(2*H*)-carboxylate (*rac*-**1w**, 100.0 mg, 400 μmol, 1.00 equiv.), $^nBu_3SiH$ (**2h**, 44.0 mg, 220 μmol, 0.550 equiv.). Reaction was stopped after 36 h, and the crude product was purified by flash-column chromatography using *n*-pentane/diethyl ethyl (40:1) as eluent. The title compound (*S*)-**3wh** was isolated as colorless oil (79.6 mg, 44.4% yield, 86.2% ee).

$\mathbf{R_f}$ = 0.39 (*n*-pentane/diethyl ethyl 20:1). **^{1}H NMR** (500 MHz, C_6D_6): δ/ppm = 8.11 (d, *J* = 8.0 Hz, 1H), 7.43–7.41 (m, 1H), 7.17–7.14 (m, 1H), 6.99–6.96 (m, 1H), 4.62 (t, *J* = 5.2 Hz, 1H), 3.86–3.71 (m, 2H), 1.76 (dd, *J* = 11.9, 6.3 Hz, 2H), 1.45 (s, 9H), 1.41–1.31 (m, 12H), 0.91 (t, *J* = 7.1 Hz, 9H), 0.67–0.64 (m, 6H). **^{13}C NMR** (126 MHz, C_6D_6): δ/ppm = 153.7, 138.1, 132.5, 127.8, 127.3, 124.2, 123.0, 80.4, 67.5, 41.4, 33.3, 28.4, 27.0, 25.9, 14.4, 14.0. **HRMS** (EI) for $C_{26}H_{45}NO_3Si^+$ $[M]^+$ calcd *m/z* 446.3163 found 446.3174. **IR** (ATR): $\tilde{\nu}$ /cm^{-1} = 2955, 2920, 2870, 1697, 1604, 1489, 1455, 1365, 1330, 1295, 1246, 1164, 1134, 1077, 1017, 961, 885, 810, 786, 752. **Optical rotation**: $[\alpha]_D^{20}$ = –25.7 (*c* 0.80, $CHCl_3$, 86.2% ee). The enantiomeric excess of **3wh** was determined after deprotection using the same HPLC setup as for **1w** (Daicel Chiralcel OD-H column, column temperature 20°C, solvent *n*-heptane:isopropanol = 97:3, flow rate 0.8 mL/min, λ = 250 nm): t_R = 25.1 min for (*S*)-**1w**, t_R = 27.4 min for (*R*)-**1w**.

Kinetic resolution data for **1w**

Run	Conversion of *rac*-**1w** [%]	ee of silyl ether (*S*)-**3wh** [%]	ee of unreacted (*R*)-**1w** [%]	Selectivity factor s
1	50.0	86.2	86.2	37.3

(1*R*,2*S*)-2-Phenylcyclohexan-1-ol [(1*R*,2*S*)-4]

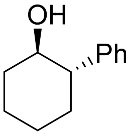

(1*R*,2*S*)-**4**

$C_{12}H_{16}O$

M = 176.26 g/mol

Prepared according to GP1 from *rac-trans*-2-phenylcyclohexan-1-ol (*rac-trans*-**4**, 71.0 mg, 400 µmol, 1.00 equiv.), $^{n}Bu_3SiH$ (**2h**, 44.0 mg, 220 µmol, 0.550 equiv.). Reaction was stopped after 108 h, and the crude product was purified by flash-column chromatography using *n*-pentane/diethyl ethyl (4:1) as eluent. The title compound (1*R*,2*S*)-**4** was isolated as white solid (26.3 mg, 37.0% yield, 81.0% ee).

M.p.: 57–58°C. $\mathbf{R_f}$ = 0.33 (*n*-pentane/diethyl ethyl 4:1). **^{1}H NMR** (500 MHz, C_6D_6): δ/ppm = 7.18–7.15 (m, 2H), 7.09–7.06 (m, 3H), 3.42 (td, *J* = 10.3, 4.2 Hz, 1H), 2.32–2.27 (m, 1H), 2.07–2.04 (m, 1H), 1.70–1.65 (m, 1H), 1.63–1.58 (m, 1H), 1.51–1.48 (m, 1H), 1.40–1.03 (m, 5H). **^{13}C NMR** (126 MHz, C_6D_6): δ/ppm = 144.1, 128.9, 128.3, 126.9, 74.3, 53.7, 35.2, 33.6, 26.4, 25.4. **HRMS** (APCI) for $C_{12}H_{15}O^{+}$ $[M–H]^{+}$ calcd *m/z* 175.1117 found 175.1113. **IR** (ATR): $\tilde{\nu}$/cm^{-1} = 3294, 2928, 2852, 1490, 1444, 1334, 1304, 1228, 1129, 1048, 1000, 961, 892, 775, 745, 693. **Optical rotation**: $[\alpha]_D^{20}$ = –51.3 (*c* 1.1, MeOH, 81% ee); $[\alpha]_D^{23}$ = +59.3 (*c* 1.83, MeOH, 98% ee) reported for (1*S*,2*R*)-enantiomer.[25] The enantiomeric excess of (1*R*,2*S*)-**4** was determined by HPLC analysis on a chiral stationary phase (Daicel Chiralcel OD-H column, column temperature 20°C, solvent *n*-heptane:isopropanol = 98:2, flow rate 0.8 mL/min, λ = 210 nm): t_R = 16.7 min for (1*S*,2*R*)-**4**, t_R = 18.8 min for (1*R*,2*S*)-**4**.

The analytical data are in accordance with those reported.[25,26]

Tributyl(((1*S*,2*R*)-2-phenylcyclohexyl)oxy)silane [(1*S*,2*R*)-5h]

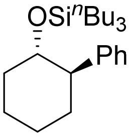

(1*S*,2*R*)-**5h**

$C_{24}H_{42}OSi$

M = 374.68 g/mol

Prepared according to GP1 from *rac-trans*-2-phenylcyclohexan-1-ol (*rac-trans*-**4**, 71.0 mg, 400 µmol, 1.00 equiv.), $^{n}Bu_3SiH$ (**2h**, 44.0 mg, 220 µmol, 0.550 equiv.). Reaction was stopped after 108 h, and the crude product was purified by flash-column chromatography using *n*-pentane/diethyl ethyl (50:1) as eluent. The title compound (1*S*,2*R*)-**5h** was isolated as colorless oil (56.2 mg, 37.4% yield, 84.4% ee).

R_f = 0.21 (*n*-pentane). **^{1}H NMR** (500 MHz, C_6D_6): δ/ppm = 7.23–7.18 (m, 4H), 7.12–7.09 (m, 1H), 3.61 (td, *J* = 10.1, 4.4 Hz, 1H), 2.49–2.44 (m, 1H), 2.06–2.03 (m, 1H), 1.77–1.73 (m, 1H), 1.67–1.63 (m, 1H), 1.55–1.10 (m, 17H), 0.92 (t, *J* = 7.2 Hz, 9H), 0.50–0.35 (m, 6H). **^{13}C NMR** (126 MHz, C_6D_6): δ/ppm = 145.5, 128.6, 128.3, 126.4, 76.0, 53.6, 37.4, 33.3, 27.2, 26.4, 25.9, 25.6, 14.3, 14.1. **HRMS** (EI) for $C_{20}H_{33}OSi^+$ $[M–C_4H_9]^+$ calcd *m/z* 317.2295 found 317.2291. **IR** (ATR): $\tilde{\nu}$/cm^{-1} = 2953, 2920, 2854, 1447, 1408, 1375, 1192, 1096, 1078, 980, 881, 794, 772, 752, 696. **Optical rotation**: $[\alpha]_D^{20}$ = +16.5 (*c* 1.8, $CHCl_3$, 84.4% ee). The enantiomeric excess of (1*S*,2*R*)-**5h** was determined after deprotection using the same HPLC setup as for *trans*-**4** (Daicel Chiralcel OD-H column, column temperature 20°C, solvent *n*-heptane:isopropanol = 98:2, flow rate 0.8 mL/min, λ = 210 nm): t_R = 16.7 min for (1*S*,2*R*)-**4**, t_R = 18.8 min for (1*R*,2*S*)-**4**.

Kinetic resolution data for *trans*-**4**

Run	Conversion of *rac-trans*-**4** [%]	ee of silyl ether (1*S*,2*R*)-**5h** [%]	ee of unreacted (1*R*,2*S*)-**4** [%]	Selectivity factor s
1	49.0	84.4	81.0	29.1

(1*R*,2*R*)-2-Phenylcyclohexan-1-ol [(1*R*,2*R*)-4]

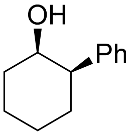

(1*R*,2*R*)-**4**

$C_{12}H_{16}O$

M = 176.26 g/mol

Prepared according to GP1 from *rac-cis*-2-phenylcyclohexan-1-ol (*rac-cis*-**4**, 71.0 mg, 400 µmol, 1.00 equiv.), $^{n}Bu_3SiH$ (**2h**, 44.0 mg, 220 µmol, 0.550 equiv.). Reaction was stopped after 156 h, and the crude product was purified by flash-column chromatography using *n*-pentane/diethyl ethyl (40:1) as eluent. The title compound (1*R*,2*R*)-**4** was isolated as white solid (39.0 mg, 54.9% yield, 45.2% ee).

M.p.: 45–46°C. R_f = 0.10 (*n*-pentane/diethyl ethyl 40:1). **^{1}H NMR** (500 MHz, $CDCl_3$): δ/ppm = 7.27–7.24 (m, 2H), 7.19–7.14 (m, 3H), 3.94 (m, 1H), 2.68–2.65 (m, 1H), 2.03–1.95 (m, 1H), 1.92–1.89 (m, 1H), 1.83–1.79 (m, 1H), 1.67–1.53 (m, 3H), 1.48–1.44 (m, 1H), 1.36–1.26 (m, 1H), 1.18 (s_{br}, 1H). **^{13}C NMR** (126 MHz, $CDCl_3$): δ/ppm = 144.1, 128.7, 127.9, 126.6, 70.7, 48.2, 33.1, 26.4, 24.5, 19.7. **HRMS** (APCI) for $C_{12}H_{15}O^+$ $[M–H]^+$ calcd *m/z* 175.1117 found 175.1111. **IR** (ATR): $\tilde{\nu}$/cm^{-1} = 3540, 3431, 2930, 2859, 1598, 1494, 1446, 1328, 1281, 1226, 1179, 1119, 1048, 992, 965, 774, 745, 701, 661. **Optical rotation**: $[\alpha]_D^{20}$ = –31.4 (*c* 1.2, MeOH, 45.2% ee); $[\alpha]_D^{27}$ = +202.3 (*c* 0.11, MeOH, 99% ee) reported for (1*S*,2*S*)-enantiomer.[26a] The enantiomeric excess of (1*R*,2*R*)-**4** was determined by HPLC analysis on a chiral stationary phase (Daicel Chiralcel OD-H column, column temperature 20°C, solvent *n*-heptane:isopropanol = 98:2, flow rate 0.8 mL/min, λ = 210 nm): t_R = 14.3 min for (1*S*,2*S*)-**4**, t_R = 18.3 min for (1*R*,2*R*)-**4.**

The analytical data are in accordance with those reported.[27]

Tributyl(((1*S*,2S)-2-phenylcyclohexyl)oxy)silane [(1*S*,2*S*)-5h]

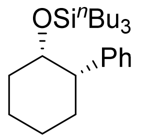

(1*S*,2*S*)-**5h**

$C_{24}H_{42}OSi$

M = 374.68 g/mol

Prepared according to GP1 from *rac-cis*-2-phenylcyclohexan-1-ol (*rac-cis*-**4**, 71.0 mg, 400 µmol, 1.00 equiv.), $^{n}Bu_3SiH$ (**2h**, 44.0 mg, 220 µmol, 0.550 equiv.). Reaction was stopped after 156 h, and the crude product was purified by flash-column chromatography using *n*-pentane as eluent. The title compound (1*S*,2*S*)-**5h** was isolated as colorless oil (51.2 mg, 34.1% yield, 69.6% ee).

R_f = 0.60 (*n*-pentane). **^{1}H NMR** (500 MHz, C_6D_6): δ/ppm = 7.25–7.20 (m, 4H), 7.14–7.10 (m, 1H), 3.98 (m, 1H), 2.50–2.46 (m, 1H), 2.30–2.22 (m, 1H), 1.94–1.79 (m, 3H), 1.58–1.55 (m, 1H), 1.47–1.44 (m, 2H), 1.36–1.20 (m, 13H), 0.92 (t, *J* = 7.2 Hz, 9H), 0.48–0.35 (m, 6H). **^{13}C NMR** (126 MHz, C_6D_6): δ/ppm = 145.5, 128.7, 128.1, 126.3, 72.0, 49.3, 35.2, 27.2, 26.8, 25.9, 25.2, 20.2, 14.2, 14.0. **HRMS** (EI) for $C_{20}H_{33}OSi^+$ $[M–C_4H_9]^+$ calcd *m/z* 317.2295 found 317.2295. **IR** (ATR): $\tilde{\nu}/cm^{-1}$ = 2953, 2920, 2865, 1602, 1454, 1375, 1183, 1117, 1077, 1056, 1019, 890, 767, 749, 696. **Optical rotation**: $[\alpha]_D^{20}$ = +39.3 (*c* 1.8, $CHCl_3$, 69.6% ee). The enantiomeric excess of (1*S*,2*S*)-**5h** was determined after deprotection using the same HPLC setup as for *cis*-**4** (Daicel Chiralcel OD-H column, column temperature 20°C, solvent *n*-heptane:isopropanol = 98:2, flow rate 0.8 mL/min, λ = 210 nm): t_R = 14.3 min for (1*S*,2*S*)-**4**, t_R = 18.3 min for (1*R*,2*R*)-**4**.

Kinetic resolution data for *cis*-**4**

Run	Conversion of *rac-cis*-**4** [%]	ee of silyl ether (1*S*,2*S*)-**5h** [%]	ee of unreacted (1*R*,2*R*)-**4** [%]	Selectivity factor s
1	39.4	69.6	45.2	8.61

(*R*,*E*)-3-Methyl-4-phenylbut-3-en-2-ol [(*R*)-6a]

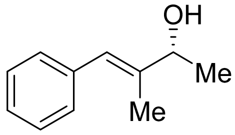

(*R*)-**6a**

$C_{11}H_{14}O$

M = 162.23 g/mol

1st run: Prepared according to GP2 from *rac*-(*E*)-3-methyl-4-phenylbut-3-en-2-ol (*rac*-**6a**, 65.0 mg, 400 µmol, 1.00 equiv.), $^nBu_3SiH$ (**2h**, 44.0 mg, 220 µmol, 0.550 equiv.), styrene (25.0 mg, 240 µmol, 0.600 equiv.). Reaction was stopped after 12 h, and the crude product was purified by flash-column chromatography using *n*-pentane/diethyl ethyl (4:1) as eluent. The title compound (*R*)-**6a** was isolated as pale yellow oil (28.5 mg, 43.8% yield, 82.6% ee).

2nd run: Prepared according to GP2 from *rac*-(*E*)-3-methyl-4-phenylbut-3-en-2-ol (*rac*-**6a**, 65.0 mg, 400 µmol, 1.00 equiv.), $^nBu_3SiH$ (**2h**, 44.0 mg, 220 µmol, 0.550 equiv.), styrene (25.0 mg, 240 µmol, 0.600 equiv.). Reaction was stopped after 18 h, and the crude product was purified by flash-column chromatography using *n*-pentane/diethyl ethyl (5:1) as eluent. The title compound (*R*)-**6a** was isolated as pale yellow oil (22.4 mg, 39.1% yield, 87.4% ee).

R_f = 0.15 (*n*-pentane/diethyl ethyl 4:1). **^{1}H NMR** (500 MHz, C_6D_6): δ/ppm = 7.24–7.18 (m, 4H), 7.09–7.05 (m, 1H), 6.46 (s, 1H), 4.04 (q, *J* = 6.4 Hz, 1H), 1.72 (d, *J* = 1.4 Hz, 3H), 1.17 (d, *J* = 6.4 Hz, 3H), 1.05 (s_{br}, 1H). **^{13}C NMR** (126 MHz, C_6D_6): δ/ppm = 142.3, 138.4, 129.4, 128.4, 126.6, 124.4, 73.4, 22.0, 13.5. **HRMS** (APCI) for $C_{11}H_{13}O^+$ $[M–H]^+$ calcd *m/z* 161.0961 found 161.0957. **IR** (ATR): $\tilde{\nu}$/cm^{-1} = 3338, 3054, 2972, 2862, 1653, 1598, 1490, 1441, 1366, 1315, 1285, 1177, 1071, 1027, 963, 906, 864, 741, 696. **Optical rotation**: $[\alpha]_D^{20}$ = –12.1 (*c* 0.77, $CHCl_3$, 87.4% ee); $[\alpha]_D^{20}$ = +6.2 (*c* 0.34, $CHCl_3$, 91% ee) reported for (*S*)-enantiomer.[28] The enantiomeric excess of (*R*)-**6a** was determined by HPLC analysis on a chiral stationary phase (Daicel Chiralcel OD-H column, column temperature 20°C, solvent *n*-heptane:isopropanol = 95:5, flow rate 0.8 mL/min, λ = 254 nm): t_R = 11.3 min for (*R*)-**6a**, t_R = 12.9 min for (*S*)-**6a**.

The analytical data are in accordance with those reported.[28]

(*S*,*E*)-Tributyl((3-methyl-4-phenylbut-3-en-2-yl)oxy)silane [(*S*)-7ah]

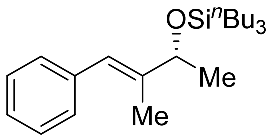

(*S*)-**7ah**

$C_{23}H_{40}OSi$

M = 360.66 g/mol

1st run: Prepared according to GP2 from *rac*-(*E*)-3-methyl-4-phenylbut-3-en-2-ol (*rac*-**6a**, 65.0 mg, 400 µmol, 1.00 equiv.), $^{n}Bu_3SiH$ (**2h**, 44.0 mg, 220 µmol, 0.550 equiv.), styrene (25.0 mg, 240 µmol, 0.600 equiv.). Reaction was stopped after 12 h, and the crude product was purified by flash-column chromatography using *n*-pentane/diethyl ethyl (100:1) as eluent. The title compound (*S*)-**7ah** was isolated as colorless oil (65.0 mg, 44.8% yield, 79.2% ee).

2nd run: Prepared according to GP2 from *rac*-(*E*)-3-methyl-4-phenylbut-3-en-2-ol (*rac*-**6a**, 65.0 mg, 400 µmol, 1.00 equiv.), $^{n}Bu_3SiH$ (**2h**, 44.0 mg, 220 µmol, 0.550 equiv.), styrene (25.0 mg, 240 µmol, 0.600 equiv.). Reaction was stopped after 18 h, and the crude product was purified by flash-column chromatography using *n*-pentane/diethyl ethyl (50:1) as eluent. The title compound (*S*)-**7ah** was isolated as colorless oil (73.4 mg, 50.9% yield, 74.6% ee).

R_f = 0.17 (*n*-pentane). **^{1}H NMR** (400 MHz, C_6D_6): δ/ppm = 7.30–7.28 (m, 2H), 7.21–7.17 (m, 2H), 7.08–7.04 (m, 1H), 6.59 (s, 1H), 4.33 (q, *J* = 6.3 Hz, 1H), 1.86 (d, *J* = 1.3 Hz, 3H), 1.51–1.35 (m, 12H), 1.33 (d, *J* = 6.3 Hz, 3H), 0.94 (t, *J* = 7.1 Hz, 9H), 0.75–0.71 (m, 6H). **^{13}C NMR** (126 MHz, C_6D_6): δ/ppm = 142.4, 138.5, 129.3, 128.5, 126.6, 124.4, 74.6, 27.1, 26.1, 23.7, 14.3, 14.1, 13.3. **HRMS** (EI) for $C_{23}H_{40}OSi^+$ $[M]^+$ calcd *m/z* 360.2843 found 360.2850. **IR** (ATR): $\tilde{\nu}/cm^{-1}$ = 2954, 2919, 2869, 2856, 1492, 1458, 1374, 1296, 1191, 1107, 1077, 1050, 982, 962, 883, 740, 696. **Optical rotation** was measured as the deprotected and purified alcohol: $[\alpha]_D^{20}$ = +9.6 (*c* 1.0, $CHCl_3$, 74.6% ee). The enantiomeric excess of (*S*)-**7ah** was determined after deprotection using the same HPLC setup as for **6a** (Daicel Chiralcel OD-H column, column temperature 20°C, solvent *n*-heptane:isopropanol = 95:5, flow rate 0.8 mL/min, λ = 254 nm): t_R = 11.3 min for (*R*)-**6a**, t_R = 12.9 min for (*S*)-**6a**.

Kinetic resolution data for **6a**

Run	Conversion of *rac*-**6a** [%]	ee of silyl ether (*S*)-**7ah** [%]	ee of unreacted (*R*)-**6a** [%]	Selectivity factor s
1	51.1	79.2	82.6	21.7
2	54.0	74.6	87.4	19.1

(*R*,*E*)-3-(4-Methoxyphenyl)-4-phenylbut-3-en-2-ol [(*R*)-6b]

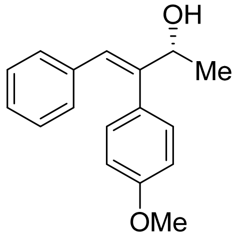

(*R*)-**6b**

$C_{17}H_{18}O_2$

M = 254.33 g/mol

Prepared according to GP2 from *rac-(E)-3-*(4-methoxyphenyl)-4-phenylbut-3-en-2-ol (*rac*-**6b**, 102.0 mg, 400 µmol, 1.00 equiv.), ^{n}Bu$_3$SiH (**2h**, 44.0 mg, 220 µmol, 0.550 equiv.), styrene (25.0 mg, 240 µmol, 0.600 equiv.). Reaction was stopped after 18 h, and the crude product was purified by flash-column chromatography using *n*-pentane/diethyl ethyl (3:2) as eluent. The title compound (*R*)-**6b** was isolated as pale yellow oil (32.1 mg, 31.5% yield, 91.6% ee).

R_f = 0.43 (*n*-pentane/ethyl acetate 3:1). **^{1}H NMR** (500 MHz, $CDCl_3$): δ/ppm = 7.12–7.07 (m, 5H), 6.97–6.95 (m, 2H), 6.89–6.86 (m, 2H), 6.66 (s, 1H), 4.64 (q, *J* = 6.2 Hz, 1H), 3.82 (s, 3H), 1.78 (s_{br}, 1H), 1.30 (d, *J* = 5.5 Hz, 3H). **^{13}C NMR** (126 MHz, $CDCl_3$): δ/ppm = 159.0, 145.9, 136.9, 130.7, 130.5, 129.3, 128.1, 126.7, 125.7, 114.3, 73.2, 55.3, 22.4. **HRMS** (APCI) for $C_{17}H_{17}O_2^+$ $[M–H]^+$ calcd *m/z* 253.1223 found 253.1219. **IR** (ATR): $\tilde{\nu}$/cm^{-1} = 3335, 2968, 2928, 2834, 1604, 1508, 1443, 1283, 1241, 1175, 1108, 1029, 919, 890, 828, 802, 754, 694, 672. **Optical rotation**: $[\alpha]_D^{20}$ = –20.9 (*c* 1.4, $CHCl_3$, 91.6% ee); absolute configuration was assigned by comparison with reported optical rotation for Ar = Ph not 4-anisyl: $[\alpha]_D^{20}$ = –55.5 (*c* 1.0, $CHCl_3$, 99.4% ee) for (*R*)-enantiomer.[29] The enantiomeric excess of (*R*)-**6b** was determined by HPLC analysis on a chiral stationary phase (Daicel Chiralcel OD-H column, column temperature 20°C, solvent *n*-heptane:isopropanol = 95:5, flow rate 0.8 mL/min, λ = 254 nm): t_R = 22.4 min for (*R*)-**6b**, t_R = 27.1 min for (*S*)-**6b**.

The analytical data are in accordance with those reported.[29

(*S*,*E*)-Tributyl((3-(4-methoxyphenyl)-4-phenylbut-3-en-2-yl)oxy)silane [(*S*)-7bh]

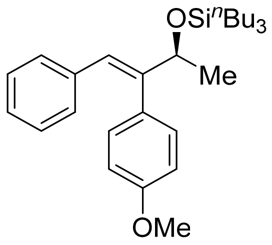

(*S*)-**7bh**

$C_{29}H_{44}O_2Si$

M = 452.75 g/mol

Prepared according to GP2 from *rac-(E)-3-*(4-methoxyphenyl)-4-phenylbut-3-en-2-ol (*rac*-**6b**, 102.0 mg, 400 μmol, 1.00 equiv.), $^nBu_3SiH$ (**2h**, 44.0 mg, 220 μmol, 0.550 equiv.), styrene (25.0 mg, 240 μmol, 0.600 equiv.). Reaction was stopped after 18 h, and the crude product was purified by flash-column chromatography using *n*-pentane/diethyl ethyl (50:1) as eluent. The title compound (*S*)-**7bh** was isolated as colorless oil (83.8 mg, 46.3% yield, 80.6% ee).

$\mathbf{R_f}$ = 0.54 (*n*-pentane/ethyl acetate 20:1). **^{1}H NMR** (500 MHz, C_6D_6): δ/ppm = 7.24–7.19 (m, 4H), 7.02–6.97 (m, 3H), 6.92–6.88 (m, 1H), 6.79–6.75 (m, 2H), 4.76–4.71 (m, 1H), 3.26 (s, 3H), 1.57–1.49 (m, 6H), 1.46–1.37 (m, 9H), 0.94 (t, *J* = 7.3 Hz, 9H), 0.82–0.78 (m, 6H). **^{13}C NMR** (126 MHz, C_6D_6): δ/ppm = 159.5, 146.7, 137.9, 131.8, 130.8, 129.6, 128.3, 126.8, 125.7, 114.5, 73.9, 54.7, 27.2, 26.1, 24.1, 14.4, 14.1. **HRMS** (EI) for $C_{29}H_{44}O_2Si^+$ $[M]^+$ calcd *m/z* 452.3105 found 452.3105. **IR** (ATR): $\tilde{\nu}$/cm^{-1} = 2954, 2919, 2869, 1606, 1509, 1460, 1285, 1243, 1175, 1132, 1077, 1054, 1035, 993, 949, 883, 830, 786, 752, 694. **Optical rotation**: $[\alpha]_D^{20}$ = +46.4 (*c* 1.6, $CHCl_3$, 80.6% ee). The enantiomeric excess of (*S*)-**7bh** was determined after deprotection using the same HPLC setup as for **6b** (Daicel Chiralcel OD-H column, column temperature 20°C, solvent *n*-heptane:isopropanol = 95:5, flow rate 0.8 mL/min, λ = 254 nm): t_R = 22.4 min for (*R*)-**6b**, t_R = 27.1 min for (*S*)-**6b**.

Kinetic resolution data for **6b**

Run	Conversion of *rac*-**6b** [%]	ee of silyl ether (*S*)-**7bh** [%]	ee of unreacted (*R*)-**6b** [%]	Selectivity factor s
1	53.2	80.6	91.6	29.6

(*R*,*Z*)-3-Bromo-4-phenylbut-3-en-2-ol [(*R*)-6c]

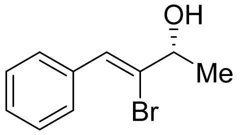

(*R*)-**6c**

$C_{10}H_{11}BrO$

M = 227.10 g/mol

1st run: Prepared according to GP2 from *rac*-(*Z*)-3-bromo-4-phenylbut-3-en-2-ol (*rac*-**6c**, 91.0 mg, 400 μmol, 1.00 equiv.), $^{n}Bu_3SiH$ (**2h**, 44.0 mg, 220 μmol, 0.550 equiv.), styrene (25.0 mg, 240 μmol, 0.600 equiv.). Reaction was stopped after 36 h, and the crude product was purified by flash-column chromatography using *n*-pentane/diethyl ethyl (9:1) as eluent. The title compound (*R*)-**6c** was isolated as pale yellow oil (33.7 mg, 37.0% yield, 78.0% ee).

2nd run: Prepared according to GP2 from *rac*-(*Z*)-3-bromo-4-phenylbut-3-en-2-ol (*rac*-**6c**, 91.0 mg, 400 μmol, 1.00 equiv.), $^{n}Bu_3SiH$ (**2h**, 44.0 mg, 220 μmol, 0.550 equiv.), styrene (25.0 mg, 240 μmol, 0.600 equiv.). Reaction was stopped after 36 h, and the crude product was purified by flash-column chromatography using *n*-pentane/diethyl ethyl (6:1) as eluent. The title compound (*R*)-6c was isolated as pale yellow oil (41.4 mg, 45.5% yield, 86.4% ee).

$\mathbf{R}_f$ = 0.31 (*n*-pentane/diethyl ethyl 4:1). **^{1}H NMR** (500 MHz, $CDCl_3$): δ/ppm = 7.62–7.59 (m, 2H), 7.38–7.35 (m, 2H), 7.33–7.29 (m, 1H), 7.08 (s, 1H), 4.49 (q, *J* = 6.2 Hz, 1H), 2.17 (s_{br}, 1H), 1.48 (d, *J* = 6.3 Hz, 3H). **^{13}C NMR** (126 MHz, $CDCl_3$): δ/ppm = 135.3, 131.6, 129.2, 128.3, 128.2, 127.1, 73.8, 22.7. **HRMS** (APCI) for $C_{10}H_{10}BrO^+$ $[M–H]^+$ calcd *m/z* 224.9910 found 224.9907. **IR** (ATR): $\tilde{\nu}/cm^{-1}$ = 3325, 3055, 2976, 2928, 2873, 1637, 1597, 1490, 1443, 1367, 1257, 1129, 1067, 1050, 961, 879, 861, 822, 750, 691, 672. **Optical rotation**: $[\alpha]_D^{20}$ = +14.3 (*c* 0.95, acetone, 78.0% ee). Absolute configuration was assigned based on analogy. The enantiomeric excess of (*R*)-**6c** was determined by HPLC analysis on a chiral stationary phase (Daicel Chiralcel OD-H column, column temperature 20°C, solvent *n*-heptane:isopropanol = 98:2, flow rate 0.8 mL/min, λ = 254 nm): t_R = 38.0 min for (*R*)-**6c**, t_R = 42.7 min for (*S*)-**6c**.

The analytical data are in accordance with those reported.[28]

(*S*,*Z*)-((3-Bromo-4-phenylbut-3-en-2-yl)oxy)tributylsilane [(*S*)-7ch]

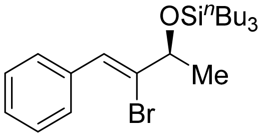

(*S*)-**7ch**

$C_{22}H_{37}BrOSi$

M = 425.53 g/mol

1st run: Prepared according to GP2 from *rac*-(*Z*)-3-bromo-4-phenylbut-3-en-2-ol (*rac*-**6c**, 91.0 mg, 400 µmol, 1.00 equiv.), ^{n}Bu$_3$SiH (**2h**, 44.0 mg, 220 µmol, 0.550 equiv.), styrene (25.0 mg, 240 µmol, 0.600 equiv.). Reaction was stopped after 36 h, and the crude product was purified by flash-column chromatography using *n*-pentane/diethyl ethyl (100:1) as eluent. The title compound (*S*)-**7ch** was isolated as pale yellow oil (67.0 mg, 39.4% yield, 88.4% ee).

2nd run: Prepared according to GP2 from *rac*-(*Z*)-3-bromo-4-phenylbut-3-en-2-ol (*rac*-**6c**, 91.0 mg, 400 µmol, 1.00 equiv.), ^{n}Bu$_3$SiH (**2h**, 44.0 mg, 220 µmol, 0.550 equiv.), styrene (25.0 mg, 240 µmol, 0.600 equiv.). Reaction was stopped after 36 h, and the crude product was purified by flash-column chromatography using *n*-pentane/diethyl ethyl (100:1) as eluent. The title compound (*S*)-**7ch** was isolated as pale yellow oil (81.7 mg, 47.8% yield, 88.0% ee).

R_f = 0.49 (*n*-pentane/diethyl ethyl 50:1). **^{1}H NMR** (500 MHz, C_6D_6): δ/ppm = 7.68–7.66 (m, 2H), 7.21 (s, 1H), 7.17–7.14 (m, 2H), 7.06–7.03 (m, 1H), 4.51–7.47 (m, 1H), 1.48 (d, *J* = 6.2 Hz, 3H), 1.47–1.33 (m, 12H), 0.92 (t, *J* = 7.2 Hz, 9H), 0.72–0.67 (m, 6H). **^{13}C NMR** (126 MHz, C_6D_6): δ/ppm = 136.0, 132.0, 129.4, 128.5, 128.3, 126.4, 74.6, 27.0, 26.0, 24.2, 14.1, 14.0. **HRMS** (EI) for $C_{18}H_{28}BrOSi^+$ $[M–C_4H_9]^+$ calcd *m/z* 367.1087 found 367.1089. **IR** (ATR): $\tilde{\nu}$/cm^{-1} = 2954, 2920, 2869, 1456, 1429, 1256, 1193, 1137, 1095, 1076, 1061, 1028, 998, 934, 883, 776, 749, 691. **Optical rotation**: $[\alpha]_D^{20}$ = –26.4 (*c* 1.5, $CHCl_3$, 88.0% ee). The enantiomeric excess of (*S*)-**7ch** was determined after deprotection using the same HPLC setup as for **6c** (Daicel Chiralcel OD-H column, column temperature 20°C, solvent *n*-heptane:isopropanol = 98:2, flow rate 0.8 mL/min, λ = 254 nm): t_R = 38.0 min for (*R*)-**6c**, t_R = 42.7 min for (*S*)-**6c**.

Kinetic resolution data for **6c**

Run	Conversion of *rac*-**6c** [%]	ee of silyl ether (*S*)-**7ch** [%]	ee of unreacted (*R*)-**6c** [%]	Selectivity factor s
1	46.9	88.4	78.0	38.0
2	49.5	88.0	86.4	44.2

(*R*)-1-(Cyclohex-1-en-1-yl)ethan-1-ol [(*R*)-6d]

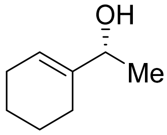

(*R*)-**6d**

$C_8H_{14}O$

M = 126.20 g/mol

Prepared according to GP2 from *rac*-1-(cyclohex-1-en-1-yl)ethan-1-ol (*rac*-**6d**, 126.0 mg, 1.00 mmol, 1.00 equiv.), ^{n}Bu$_3$SiH (**2h**, 110 mg, 550 µmol, 0.550 equiv.), styrene (63.0 mg, 600 µmol, 0.600 equiv.). Reaction was stopped after 18 h, and the crude product was purified by flash-column chromatography using *n*-pentane/diethyl ethyl (4:1) as eluent. The title compound (*R*)-**6d** was isolated as colorless oil (40.0 mg, 31.7% yield, 68.0% ee).

R_f = 0.26 (*n*-pentane/diethyl ethyl 4:1). **^{1}H NMR** (500 MHz, $CDCl_3$): δ/ppm = 5.66–5.64 (m, 1H), 4.15 (q, *J* = 6.4 Hz, 1H), 2.07–1.94 (m, 4H), 1.69–1.50 (m, 4H), 1.46 (s_{br}, 1H), 1.24 (d, *J* = 6.5 Hz, 3H). **^{13}C NMR** (126 MHz, $CDCl_3$): δ/ppm = 141.4, 121.6, 72.3, 25.0, 23.8, 22.8, 22.7, 21.6. **HRMS** (APCI) for $C_8H_{13}O^+$ $[M–H]^+$ calcd *m/z* 125.0961 found 125.0957. **IR** (ATR): $\tilde{\nu}$/cm^{-1} = 3330, 2971, 2924, 2856, 1667, 1437, 1365, 1291, 1164, 1136, 1095, 1070, 1058, 1006, 917, 886, 843, 801, 750. **Optical rotation**: $[\alpha]_D^{20}$ = +6.4 (*c* 0.91, $CHCl_3$, 68.0% ee); $[\alpha]_D^{25}$ = –9.5 (*c* 1.2, $CHCl_3$, 97% ee) reported for (*S*)-enantiomer.[30] The enantiomeric excess of (*R*)-**6d** was determined by HPLC analysis on a chiral stationary phase (Daicel Chiralcel AD-H column, column temperature 20°C, solvent *n*-heptane:isopropanol = 98:2, flow rate 0.8 mL/min, λ = 210 nm): t_R = 13.4 min for (*S*)-**6d**, t_R = 14.3 min for (*R*)-**6d**.

The analytical data are in accordance with those reported.[30]

(*S*)-Tributyl(1-(cyclohex-1-en-1-yl)ethoxy)silane [(*S*)-7dh]

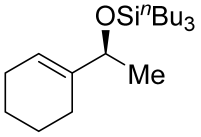

(*S*)-**7dh**

$C_{20}H_{40}OSi$

M = 324.62 g/mol

Prepared according to GP2 from *rac*-1-(cyclohex-1-en-1-yl)ethan-1-ol (*rac*-**6d**, 126.0 mg, 1.00 mmol, 1.00 equiv.), $^{n}Bu_3SiH$ (**2h**, 110 mg, 550 µmol, 0.550 equiv.), styrene (63.0 mg, 600 µmol, 0.600 equiv.). Reaction was stopped after 18 h, and the crude product was purified by flash-column chromatography using *n*-pentane/diethyl ethyl (100:1) as eluent. The title compound (*S*)-**7dh** was isolated as colorless oil (139.7 mg, 43.0% yield, 75.6% ee).

$\mathbf{R}_f$ = 0.23 (*n*-pentane). **^{1}H NMR** (500 MHz, C_6D_6): δ/ppm = 5.67–5.66 (m, 1H), 4.22 (q, *J* = 6.3 Hz, 1H), 2.23–2.18 (m, 1H), 2.01–1.92 (m, 3H), 1.65–1.50 (m, 3H), 1.50–1.37 (m, 13H), 1.30 (d, *J* = 6.4 Hz, 3H), 0.95 (t, *J* = 7.2 Hz, 9H), 0.72–0.69 (m, 6H). **^{13}C NMR** (126 MHz, C_6D_6): δ/ppm = 142.2, 120.9, 73.3, 27.2, 26.1, 25.3, 23.6, 23.5, 23.2, 23.2, 14.4, 14.1. **HRMS** (EI) for $C_{20}H_{40}OSi^+$ $[M]^+$ calcd *m/z* 324.2843 found 324.2844. **IR** (ATR): $\tilde{\nu}$ /cm^{-1} = 2955, 2920, 2870, 1459, 1375, 1291, 1192, 1071, 1022, 973, 935, 883, 786, 757, 730. **Optical rotation**: $[\alpha]_D^{20}$ = –0.92 (*c* 1.7, $CHCl_3$, 75.6% ee). The enantiomeric excess of (*S*)-**7dh** was determined after deprotection using the same HPLC setup as for **6d** (Daicel Chiralcel AD-H column, column temperature 20°C, solvent *n*-heptane:isopropanol = 98:2, flow rate 0.8 mL/min, λ = 210 nm): t_R = 13.4 min for (*S*)-**6d**, t_R = 14.3 min for (*R*)-**6d**.

Kinetic resolution data for **6d**

Run	Conversion of *rac*-**6d** [%]	ee of silyl ether (*S*)-**7dh** [%]	ee of unreacted (*R*)-**6d** [%]	Selectivity factor s
1	47.4	75.6	68.0	14.4

(*R*,*E*)-2-Benzylidenecyclopentan-1-ol [(*R*)-6e]

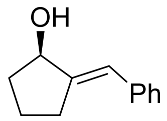

(*R*)-**6e**

$C_{12}H_{14}O$

M = 174.24 g/mol

1st run: Prepared according to GP2 from *rac*-(*E*)-2-benzylidenecyclopentan-1-ol (*rac*-**6e**, 70.0 mg, 400 µmol, 1.00 equiv.), $^{n}Bu_3SiH$ (**2h**, 44.0 mg, 220 µmol, 0.550 equiv.), styrene (42.0 mg, 400 µmol, 1.00 equiv.). Reaction was stopped after 12 h, and the crude product was purified by flash-column chromatography using *n*-pentane/diethyl ethyl (4:1) as eluent. The title compound (*R*)-**6e** was isolated as white solid (24.8 mg, 35.4% yield, 93.2% ee).

2nd run: Prepared according to GP2 from *rac*-(*E*)-2-benzylidenecyclopentan-1-ol (*rac*-**6e**, 70.0 mg, 400 µmol, 1.00 equiv.), $^{n}Bu_3SiH$ (**2h**, 44.0 mg, 220 µmol, 0.550 equiv.), styrene (25.0 mg, 240 µmol, 0.600 equiv.). Reaction was stopped after 8 h, and the crude product was purified by flash-column chromatography using *n*-pentane/diethyl ethyl (5:1) as eluent. The title compound (*R*)-**6e** was isolated as colorless oil, and solidified upon standing (30.0 mg, 42.9% yield, 99.0% ee).

M.p.: 85–86°C. $\mathbf{R_f}$ = 0.23 (*n*-pentane/diethyl ethyl 3:1). **^{1}H NMR** (400 MHz, C_6D_6): δ/ppm = 7.30–7.29 (m, 2H), 7.23–7.20 (m, 2H), 7.10–7.06 (m, 1H), 6.50 (m, 1H), 4.31 (t, *J* = 5.9 Hz, 1H), 2.46–2.37 (m, 1H), 2.28–2.20 (m, 1H), 1.72–1.58 (m, 2H), 1.46–1.31 (m, 2H), 0.78 (s_{br}, 1H). **^{13}C NMR** (126 MHz, $CDCl_3$): δ/ppm = 147.9, 137.9, 128.5, 128.4, 126.7, 123.8, 77.4, 34.9, 29.4, 22.6. **HRMS** (APCI) for $C_{12}H_{13}O^+$ $[M–H]^+$ calcd *m/z* 173.0961 found 173.0956. **IR** (ATR): $\tilde{\nu}$/cm^{-1} = 3266, 2963, 2871, 1488, 1443, 1424, 1324, 1275, 1170, 1096, 1030, 909, 876, 820, 769, 750, 687. **Optical rotation**: $[\alpha]_D^{20}$ = –49.9 (*c* 0.60, $CHCl_3$, 93.2% ee). Absolute configuration was assigned based on analogy. The enantiomeric excess of (*R*)-**6e** was determined by HPLC analysis on a chiral stationary phase (Daicel Chiralcel OD-H column, column temperature 20°C, solvent *n*-heptane:isopropanol = 95:5, flow rate 0.8 mL/min, λ = 254 nm): t_R = 22.4 min for (*R*)-**6e**, t_R = 24.6 min for (*S*)-**6e**.

The analytical data are in accordance with those reported.[31]

(*S*,*E*)-((2-Benzylidenecyclopentyl)oxy)tributylsilane [(*S*)-7eh]

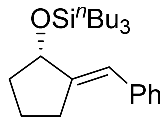

(*S*)-**7eh**

$C_{24}H_{40}OSi$

M = 372.67 g/mol

1st run: Prepared according to GP2 from *rac*-(*E*)-2-benzylidenecyclopentan-1-ol (*rac*-**6e**, 70.0 mg, 400 µmol, 1.00 equiv.), $^nBu_3SiH$ (**2h**, 44.0 mg, 220 µmol, 0.550 equiv.), styrene (42.0 mg, 400 µmol, 1.00 equiv.). Reaction was stopped after 12 h, and the crude product was purified by flash-column chromatography using *n*-pentane/diethyl ethyl (50:1) as eluent. The title compound (*S*)-**7eh** was isolated as colorless oil (64.1 mg, 43.0% yield, 90.6% ee).

2nd run: Prepared according to GP2 from *rac*-(*E*)-2-benzylidenecyclopentan-1-ol (*rac*-**6e**, 70.0 mg, 400 µmol, 1.00 equiv.), $^nBu_3SiH$ (**2h**, 44.0 mg, 220 µmol, 0.550 equiv.), styrene (25.0 mg, 240 µmol, 0.600 equiv.). Reaction was stopped after 8 h, and the crude product was purified by flash-column chromatography using *n*-pentane/diethyl ethyl (50:1) as eluent. The title compound (*S*)-**7eh** was isolated as colorless oil (79.6 mg, 53.4% yield, 81.0% ee).

$\mathbf{R_f}$ = 0.30 (*n*-pentane). **^{1}H NMR** (500 MHz, C_6D_6): δ/ppm = 7.39 (d, *J* = 7.6 Hz, 2H), 7.21 (t, *J* = 7.7 Hz, 2H), 7.07 (t, *J* = 7.4 Hz, 1H), 6.77 (m, 1H), 4.57 (t, *J* = 6.2 Hz, 1H), 2.56–2.33 (m, 2H), 1.82–1.74 (m, 2H), 1.66–1.59 (m, 1H), 1.55–1.36 (m, 13H), 0.95 (t, *J* = 7.3 Hz, 9H), 0.79–0.76 (m, 6H). **^{13}C NMR** (126 MHz, C_6D_6): δ/ppm = 147.3, 138.7, 128.9, 128.6, 126.6, 123.1, 77.8, 35.4, 28.7, 27.1, 26.1, 22.1, 14.5, 14.1. **HRMS** (EI) for $C_{20}H_{31}OSi^+$ $[M–C_4H_9]^+$ calcd *m/z* 315.2139 found 315.2145. **IR** (ATR): $\tilde{\nu}$/cm^{-1} = 2954, 2918, 2869, 1599, 1459, 1407, 1374, 1342, 1293, 1192, 1153, 1115, 1061, 1041, 1002, 964, 886, 832, 750, 692. **Optical rotation**: $[\alpha]_D^{20}$ = +22.3 (*c* 3.2, $CHCl_3$, 81.0% ee). The enantiomeric excess of (*S*)-**7eh** was determined after deprotection using the same HPLC setup as for **6e** (Daicel Chiralcel OD-H column, column temperature 20°C, solvent *n*-heptane:isopropanol = 95:5, flow rate 0.8 mL/min, λ = 254 nm): t_R = 22.4 min for (*R*)-**6e**, t_R = 24.6 min for (*S*)-**6e**.

Kinetic resolution data for **6e**

Run	Conversion of *rac*-**6e** [%]	ee of silyl ether (*S*)-**7eh** [%]	ee of unreacted (*R*)-**6e** [%]	Selectivity factor s
1	50.8	90.6	93.2	66.9
2	49.5	81.0	99.0	48.9

(*R*,*E*)-2-Benzylidenecyclohexan-1-ol [(*R*)-6f]

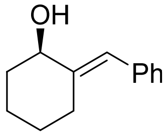

(*R*)-**6f**

$C_{13}H_{16}O$

M = 188.27 g/mol

Prepared according to GP2 from *rac*-(*E*)-2-benzylidenecyclohexan-1-ol (*rac*-**6f**, 76.0 mg, 0.400 mmol, 1.00 equiv.), $^{n}Bu_3SiH$ (**2h**, 44 mg, 220 µmol, 0.550 equiv.), styrene (25.0 mg, 240 µmol, 0.600 equiv.). Reaction was stopped after 12 h, and the crude product was purified by flash-column chromatography using *n*-pentane/diethyl ethyl (4:1) as eluent. The title compound (*R*)-**6f** was isolated as white solid (35.1 mg, 46.2% yield, 98.6% ee).

M.p.: 62–63°C. R_f = 0.33 (cyclohexane/ethyl acetate 3:1). **^{1}H NMR** (400 MHz, $CDCl_3$): δ/ppm = 7.34–7.31 (m, 2H), 7.23–7.19 (m, 3H), 6.52 (s, 1H), 4.26–4.22 (m, 1H), 2.76–2.67 (m, 1H), 2.17–2.09 (m, 1H), 2.04–1.97 (m, 1H), 1.90–1.84 (m, 1H), 1.81 (s_{br}, 1H), 1.67–1.43 (m, 4H). **^{13}C NMR** (126 MHz, $CDCl_3$): δ/ppm = 144.5, 137.8, 129.1, 128.2, 126.4, 121.0, 73.9, 36.7, 27.5, 27.1, 23.3. **HRMS** (APCI) for $C_{13}H_{15}O^+$ $[M–H]^+$ calcd *m/z* 187.1117 found 187.1112. **IR** (ATR): $\tilde{\nu}$/cm^{-1} = 3402, 3345, 3056, 3020, 2923, 2850, 1658, 1597, 1489, 1441, 1348, 1323, 1257, 1072, 1028, 950, 913, 883, 853, 777, 731, 695. **Optical rotation**: $[\alpha]_D^{20}$ = +44.3 (*c* 1.6, $CHCl_3$, 98.6% ee); $[\alpha]_D^{20}$ = –43.0 (*c* 0.76, $CHCl_3$, 97% ee) reported for (*S*)-enantiomer.[31] The enantiomeric excess of (*R*)-**6f** was determined by HPLC analysis on a chiral stationary phase (Daicel Chiralcel OD-H column, column temperature 20°C, solvent *n*-heptane:isopropanol = 95:5, flow rate 0.8 mL/min, λ = 254 nm): t_R = 15.8 min for (*R*)-**6f**, t_R = 20.7 min for (*S*)-**6f**.

The analytical data are in accordance with those reported.[31]

(*S*,*E*)-((2-Benzylidenecyclohexyl)oxy)tributylsilane [(*S*)-7fh]

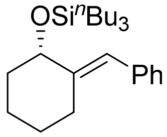

(*S*)-**7fh**

$C_{25}H_{42}OSi$

M = 386.70 g/mol

Prepared according to GP2 from *rac*-(*E*)-2-benzylidenecyclohexan-1-ol (*rac*-**6f**, 76.0 mg, 0.400 mmol, 1.00 equiv.), $^{n}Bu_3SiH$ (**2h**, 44 mg, 220 µmol, 0.550 equiv.), styrene (25.0 mg, 240 µmol, 0.600 equiv.). Reaction was stopped after 12 h, and the crude product was purified by flash-column chromatography using *n*-pentane/diethyl ethyl (100:1) as eluent. The title compound (*S*)-**7fh** was isolated as colorless oil (75.7 mg, 48.5% yield, 94.0% ee). **R_f** = 0.22 (*n*-pentane). **^{1}H NMR** (500 MHz, C_6D_6): δ/ppm = 7.30–7.28 (m, 2H), 7.21–7.18 (m, 2H), 7.07–7.05 (m, 1H), 6.74 (s, 1H), 4.27–4.25 (m, 1H), 2.79–2.74 (m, 1H), 2.16–2.11 (m, 1H), 1.89–1.80 (m, 2H), 1.79–1.74 (m, 1H), 1.53–1.37 (m, 15H), 0.94 (t, *J* = 7.3 Hz, 9H), 0.78–0.74 (m, 6H). **^{13}C NMR** (126 MHz, C_6D_6): δ/ppm = 144.9, 138.5, 129.3, 128.5, 126.5, 121.6, 74.8, 38.1, 27.9, 27.3, 27.1, 26.1, 23.4, 14.4, 14.1. **HRMS** (EI) for $C_{25}H_{42}OSi^+$ $[M]^+$ calcd *m/z* 386.2999 found 386.3003. **IR** (ATR): $\tilde{\nu}$/cm^{-1} = 2953, 2920, 2855, 1458, 1408, 1375, 1340, 1193, 1126, 1102, 1079, 1038, 1016, 962, 905, 882, 829, 783, 735, 697. **Optical rotation**: $[\alpha]_D^{20}$ = –39.7 (*c* 1.2, $CHCl_3$, 94.0% ee). The enantiomeric excess of (*S*)-**7fh** was determined after deprotection using the same HPLC setup as for **6f** (Daicel Chiralcel OD-H column, column temperature 20°C, solvent *n*-heptane:isopropanol = 95:5, flow rate 0.8 mL/min, λ = 254 nm): t_R = 15.8 min for (*R*)-**6f**, t_R = 20.7 min for (*S*)-**6f**.

Kinetic resolution data for **6f**

	Conversion of *rac*-**6f** [%]	ee of silyl ether (*S*)-**7fh** [%]	ee of unreacted (*R*)-**6f** [%]	Selectivity factor s
1	51.2	94.0	98.6	159

(*R*)-2,3,4,5-Tetrahydro-[1,1'-biphenyl]-2-ol [(*R*)-8]

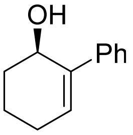

(*R*)-**8**

$C_{12}H_{14}O$

M = 174.24 g/mol

1st run: Prepared according to GP2 from *rac*-2,3,4,5-tetrahydro-[1,1'-biphenyl]-2-ol (*rac*-**8**, 70.0 mg, 400 µmol, 1.00 equiv.), $^{n}Bu_3SiH$ (**2h**, 44.0 mg, 220 µmol, 0.550 equiv.), styrene (25.0 mg, 240 µmol, 0.600 equiv.). Reaction was stopped after 12 h, and the crude product was purified by flash-column chromatography using *n*-pentane/diethyl ethyl (5:1) as eluent. The title compound (*R*)-**8** was isolated as colorless oil (28.6 mg, 40.9% yield, 76.8% ee).

2nd run: Prepared according to GP2 from *rac*-2,3,4,5-tetrahydro-[1,1'-biphenyl]-2-ol (*rac*-**8**, 70.0 mg, 400 µmol, 1.00 equiv.), $^{n}Bu_3SiH$ (**2h**, 44.0 mg, 220 µmol, 0.550 equiv.), styrene (25.0 mg, 240 µmol, 0.600 equiv.). Reaction was stopped after 12 h, and the crude product was purified by flash-column chromatography using *n*-pentane/diethyl ethyl (6:1) as eluent. The title compound (*R*)-**8** was isolated as colorless oil (38.0 mg, 54.3% yield, 66.6% ee).

$\mathbf{R_f}$ = 0.20 (*n*-pentane/diethyl ethyl 5:1). **^{1}H NMR** (400 MHz, C_6D_6): δ/ppm = 7.46–7.44 (m, 2H), 7.22–7.18 (m, 2H), 7.13–7.09 (m, 1H), 5.93 (dd, *J* = 4.7, 3.3 Hz, 1H), 4.46 (t, *J* = 3.4 Hz, 1H), 1.96–1.89 (m, 1H), 1.85–1.75 (m, 2H), 1.66–1.50 (m, 2H), 1.40–1.33 (m, 1H), 1.18 (s_{br}, 1H). **^{13}C NMR** (126 MHz, $CDCl_3$): δ/ppm = 140.3, 139.3, 128.8, 128.7, 127.2, 126.1, 65.6, 31.7, 26.2, 17.5. **HRMS** (APCI) for $C_{12}H_{13}O^+$ $[M–H]^+$ calcd *m/z* 173.0961 found 173.0956. **IR** (ATR): $\tilde{\nu}/cm^{-1}$ = 3555, 3322, 2930, 2862, 2825, 1596, 1492, 1437, 1273, 1157, 1057, 970, 915, 754, 693. **Optical rotation**: $[\alpha]_D^{20}$ = +69.8 (*c* 1.0, $CHCl_3$, 66.6% ee); $[\alpha]_D^{20}$ = +67.2 (*c* 0.36, CH_2Cl_2, 99% ee) reported for (*R*)-enantiomer.[32] The enantiomeric excess of (*R*)-**8** was determined by HPLC analysis on a chiral stationary phase (Daicel Chiralcel OD-H column, column temperature 20°C, solvent *n*-heptane:isopropanol = 95:5, flow rate 0.8 mL/min, λ = 254 nm): t_R = 12.2 min for (*R*)-**8**, t_R = 16.5 min for (*S*)-**8**.

The analytical data are in accordance with those reported.[32]

(*S*)-Tributyl((2,3,4,5-tetrahydro-[1,1'-biphenyl]-2-yl)oxy)silane [(*S*)-9h]

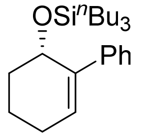

(*S*)-**9h**

$C_{24}H_{40}OSi$

M = 372.67 g/mol

1st run: Prepared according to GP2 from *rac*-2,3,4,5-tetrahydro-[1,1'-biphenyl]-2-ol (*rac*-**8**, 70.0 mg, 400 µmol, 1.00 equiv.), $^{n}Bu_3SiH$ (**2h**, 44.0 mg, 220 µmol, 0.550 equiv.), styrene (25.0 mg, 240 µmol, 0.600 equiv.). Reaction was stopped after 12 h, and the crude product was purified by flash-column chromatography using *n*-pentane as eluent. The title compound (*S*)-**9h** was isolated as colorless oil (56.5 mg, 37.9% yield, 92.0% ee).

2nd run: Prepared according to GP2 from *rac*-2,3,4,5-tetrahydro-[1,1'-biphenyl]-2-ol (*rac*-**8**, 70.0 mg, 400 µmol, 1.00 equiv.), $^{n}Bu_3SiH$ (**2h**, 44.0 mg, 220 µmol, 0.550 equiv.), styrene (25.0 mg, 240 µmol, 0.600 equiv.). Reaction was stopped after 12 h, and the crude product was purified by flash-column chromatography using *n*-pentane/diethyl ethyl (50:1) as eluent. The title compound (*S*)-**9h** was isolated as colorless oil (58.2 mg, 39.0% yield, 94.2% ee).

$\mathbf{R}_f$ = 0.10 (*n*-pentane). **^{1}H NMR** (400 MHz, C_6D_6): δ/ppm = 7.39–7.36 (m, 2H), 7.23–7.20 (m, 2H), 7.14–7.09 (m, 1H), 5.85–5.83 (m, 1H), 4.74 (t, *J* = 3.9 Hz, 1H), 2.07–1.88 (m, 4H), 1.72–1.64 (m, 1H), 1.53–1.47 (m, 1H), 1.36–1.24 (m, 12H), 0.96–0.85 (m, 9H), 0.63–0.51 (m, 6H). **^{13}C NMR** (126 MHz, C_6D_6): δ/ppm = 142.9, 141.4, 128.3, 127.5, 127.0, 66.9, 33.4, 27.1, 26.3, 25.9, 17.6, 14.6, 14.1. **HRMS** (EI) for $C_{20}H_{31}OSi^+$ $[M–C_4H_9]^+$ calcd *m/z* 315.2139 found 315.2136. **IR** (ATR): $\tilde{\nu}$/cm^{-1} = 2952, 2919, 2868, 1458, 1408, 1374, 1192, 1161, 1066, 1074, 1016, 901, 802, 754, 736, 695. **Optical rotation**: $[\alpha]_D^{20}$ = –74.6 (*c* 1.5, $CHCl_3$, 94.2% ee). The enantiomeric excess of (*S*)-**9h** was determined after deprotection using the same HPLC setup as for **8** (Daicel Chiralcel OD-H column, column temperature 20°C, solvent *n*-heptane:isopropanol = 95:5, flow rate 0.8 mL/min, λ = 254 nm): t_R = 12.2 min for (*R*)-**8**, t_R = 16.5 min for (*S*)-**8**.

Kinetic resolution data for **8**

Run	Conversion of *rac*-**8** [%]	ee of silyl ether (*S*)-**9h** [%]	ee of unreacted (*R*)-**8** [%]	Selectivity factor s
1	45.5	92.0	76.8	55.7
2	41.4	94.2	66.6	67.9

(*R*)-1-Cyclohexylethan-1-ol [(*R*)-10]

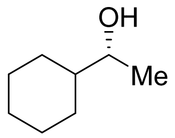

(*R*)-**10**

$C_8H_{16}O$

M = 128.12 g/mol

Prepared according to GP1 from *rac*-1-cyclohexylethan-1-ol (*rac*-**10**, 128.0 mg, 1.00 mmol, 1.00 equiv.), $^{n}Bu_3SiH$ (**2h**, 110 mg, 550 µmol, 0.550 equiv.). Reaction was stopped after 18 h, and the crude product was purified by flash-column chromatography using *n*-pentane/diethyl ethyl (10:1) as eluent. The title compound (*R*)-**10** was isolated as colorless oil (45.4 mg, 35.4% yield, 74.2% ee).

$\mathbf{R_f}$ = 0.25 (*n*-pentane/diethyl ethyl 5:1). **^{1}H NMR** (400 MHz, $CDCl_3$): δ/ppm = 3.57–3.51 (m, 1H), 1.88–1.81 (m, 1H), 1.78–1.72 (m, 2H), 1.69–1.64 (m, 2H), 1.37 (s, 1H), 1.31–1.17 (m, 4H), 1.15 (d, *J* = 3.6 Hz, 2H), 1.12–0.90 (m, 3H). ^{13}C NMR (101 MHz, $CDCl_3$): δ/ppm = 72.4, 45.3, 28.9, 28.5, 26.7, 26.4, 26.3, 20.5. **HRMS** (APCI) for $C_8H_{15}^+$ $[M–OH]^+$ calcd *m/z* 111.1168 found 111.1166. **IR** (ATR): $\tilde{\nu}$/cm^{-1} = 3346, 2968, 2920, 2850, 1447, 1371, 1126, 1062, 1041, 937, 890, 834, 770. **Optical rotation**: $[\alpha]_D^{20}$ = –1.1 (*c* 1.2, $CHCl_3$, 74.2% ee); $[\alpha]_D^{20}$ = +3.7 (*c* 0.70, $CHCl_3$, 91% ee) reported for (*S*)-enantiomer.[30] The enantiomeric excess of (*R*)-**10** was determined by HPLC analysis of the correspongding 1-cyclohexylethyl 4-nitrobenzoate derivative on a chiral stationary phase (Daicel Chiralcel ID column, column temperature 20°C, solvent *n*-heptane:isopropanol = 99:1, flow rate 0.8 mL/min, λ = 254 nm): t_R = 10.7 min for (*S*)-**10**, t_R = 11.8 min for (*R*)-**10**.

The analytical data are in accordance with those reported.[30]

(*S*)-Tributyl(1-cyclohexylethoxy)silane [(*S*)-11h]

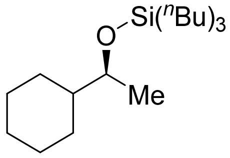

(*S*)-**11h**

$C_{20}H_{42}OSi$

M = 326.64 g/mol

Prepared according to GP1 from *rac*-1-cyclohexylethan-1-ol (*rac*-**10**, 128.0 mg, 1.00 mmol, 1.00 equiv.), $^{n}Bu_3SiH$ (**2h**, 110 mg, 550 µmol, 0.550 equiv.). Reaction was stopped after 18 h, and the crude product was purified by flash-column chromatography using *n*-pentane/diethyl ethyl (100:1) as eluent. The title compound (*R*)-**11h** was isolated as colorless oil (158.9 mg, 48.6% yield, 63.2% ee).

R*f* = 0.62 (*n*-pentane). **^{1}H NMR** (400 MHz, C_6D_6): δ/ppm = 3.74–3.68 (m, 1H), 2.00–1.97 (m, 1H), 1.89–1.80 (m, 3H), 1.76–1.72 (m, 1H), 1.61–1.48 (m, 12H), 1.46–1.27 (m, 4H), 1.23 (d, *J* = 6.2 Hz, 3H), 1.24–1.12 (m, 2H), 1.07–1.03 (m, 9H), 0.83–0.78 (m, 6H). **^{13}C NMR** (101 MHz, C_6D_6): δ/ppm = 72.8, 46.2, 29.4, 28.9, 27.2, 26.9, 26.1, 21.1, 14.6, 14.1. **HRMS** (EI) for $C_{16}H_{33}OSi^+$ $[M–C_4H_9]^+$ calcd *m/z* 269.2295 found 269.2299. **IR** (ATR): $\tilde{\nu}$ /cm^{-1} = 2955, 2919, 2853, 1450, 1374, 1190, 1137, 1070, 1023, 966, 911, 884, 842, 758, 730. **Optical rotation**: $[\alpha]_D^{20}$ = +4.1 (*c* 2.3, $CHCl_3$, 63.2% ee). The enantiomeric excess of (*S*)-**11h** was determined by corresponding 1-cyclohexylethyl 4-nitrobenzoate derivative using the following HPLC setup (Daicel Chiralcel ID column, column temperature 20°C, solvent *n*-heptane:isopropanol = 99:1, flow rate 0.8 mL/min, λ = 254 nm): t_R = 10.8 min for (*S*)-**10**, t_R = 11.8 min for (*R*)-**10**.

Kinetic resolution data for **10**

Run	Conversion of *rac*-**10** [%]	ee of silyl ether (*S*)-**11h** [%]	ee of unreacted (*R*)-**10** [%]	Selectivity factor s
1	54.0	63.2	74.2	9.62

6 HPLC Traces of Alcohols (1a–w, *trans*-4/*cis*-4, 6a–f, 8 and 10) and Silyl ethers (3ah–wh, *trans*-5h/*cis*-5h, 7ah–fh, 9h and 11h)

(*R*)-1-([1,1'-Biphenyl]-4-yl)ethan-1-ol [(*R*)-1a]

The enantiomeric excess of (*R*)-**1a** was determined by HPLC analysis on a chiral stationary phase (Daicel Chiralcel AD-H column, column temperature 20°C, solvent *n*-heptane:isopropanol = 95:5, flow rate 0.8 mL/min, λ = 254 nm): t_R = 16.3 min for (*S*)-**1a**, t_R = 17.9 min for (*R*)-**1a**.

Supplementary Figure 1. *rac-1-([1,1'-Biphenyl]-4-yl)ethan-1-ol [rac-**1a**]*

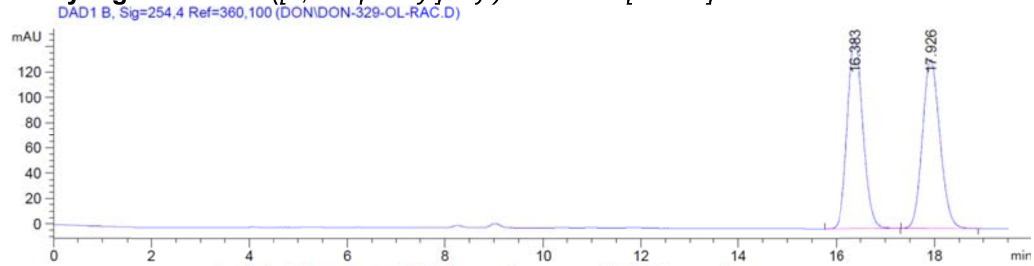

Peak #	RetTime [min]	Type	Width [min]	Area [mAU*s]	Height [mAU]	Area %
1	16.383	BB	0.3421	3318.78345	150.02032	50.2601
2	17.926	BB	0.3877	3284.43896	133.18712	49.7399

Supplementary Figure 2. *(R)-1-([1,1'-Biphenyl]-4-yl)ethan-1-ol [(R)-**1a**, 81.2% ee]*

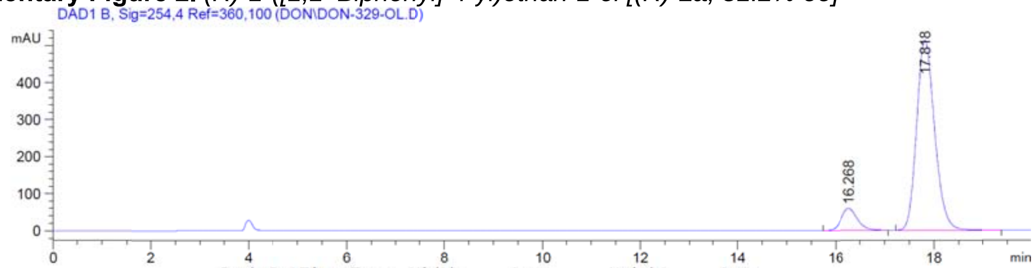

Peak #	RetTime [min]	Type	Width [min]	Area [mAU*s]	Height [mAU]	Area %
1	16.268	BB	0.3460	1343.88257	58.93392	9.3703
2	17.818	BB	0.3912	1.29981e4	513.76691	90.6297

(*S*)-(1-([1,1'-Biphenyl]-4-yl) ethoxy)tributylsilane [(*S*)-3ah, 70.8% ee]

The enantiomeric excess of (*S*)-**3ah** was determined by the deprotected alcohol, which was hydrolyzed and purified by preparative TLC according to GP5, using the same HPLC analysis condition with **1a**.

Supplementary Figure 3.

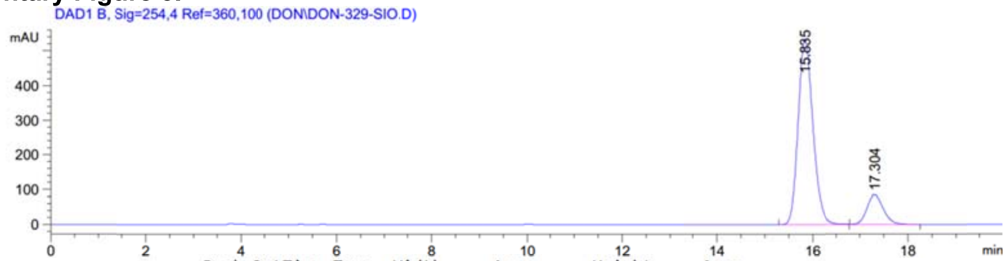

Peak #	RetTime [min]	Type	Width [min]	Area [mAU*s]	Height [mAU]	Area %
1	15.835	BB	0.3311	1.14906e4	533.97876	85.3621
2	17.304	BB	0.3616	1970.41699	84.03168	14.6379

(*R*)-1-([1,1'-Biphenyl]-4-yl)ethan-1-ol [(*R*)-1a]

The enantiomeric excess was determined by HPLC analysis on a chiral stationary phase (Daicel Chiralcel AD-H column, column temperature 20°C, solvent *n*-heptane:isopropanol = 95:5, flow rate 0.8 mL/min, λ = 254 nm): t_R = 21.8 min for (*S*)-**1a**, t_R = 23.8 min for (*R*)-**1a**.

Supplementary Figure 4. *rac-1-([1,1'-Biphenyl]-4-yl)ethan-1-ol [rac-**1a**]*

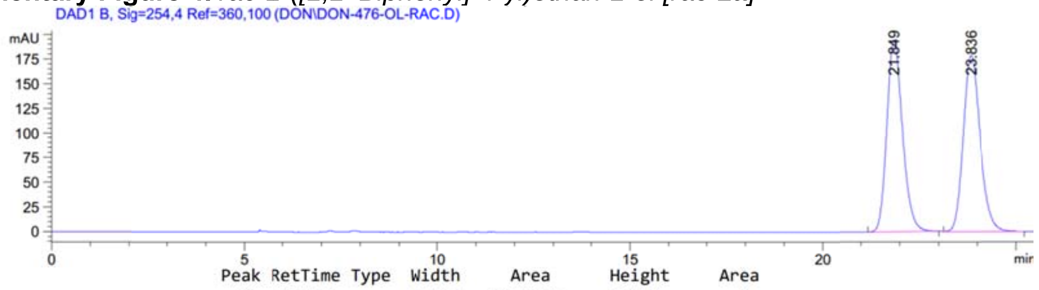

Peak #	RetTime [min]	Type	Width [min]	Area [mAU*s]	Height [mAU]	Area %
1	21.849	BB	0.4301	5443.68701	194.74149	49.8684
2	23.836	BB	0.4690	5472.42090	178.81830	50.1316

Supplementary Figure 5. *(R)-1-([1,1'-Biphenyl]-4-yl)ethan-1-ol [(R)-**1a**, 98.8% ee]*

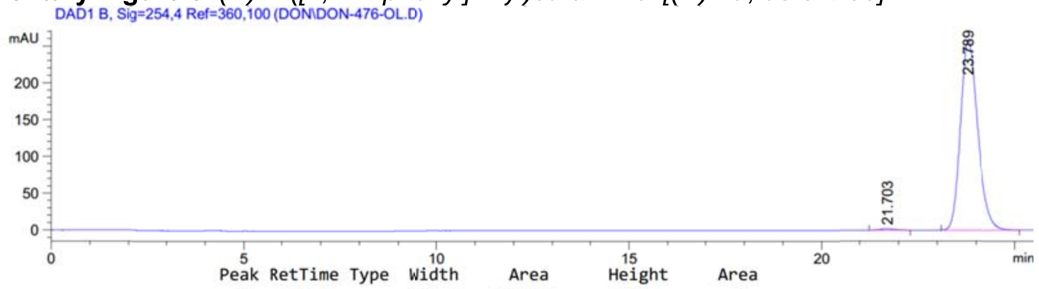

Peak #	RetTime [min]	Type	Width [min]	Area [mAU*s]	Height [mAU]	Area %
1	21.703	BB	0.3291	46.34628	1.70529	0.5710
2	23.789	BB	0.4800	8069.72803	258.62714	99.4290

(*S*)-(1-([1,1'-Biphenyl]-4-yl)ethoxy)tributylsilane [(*S*)-3ah, 46.6% ee]

The enantiomeric excess of **(*S*)-3ah** was determined by the deprotected alcohol, which was hydrolyzed and purified by preparative TLC according to GP5, using the same HPLC analysis condition with **1a**.

Supplementary Figure 6.

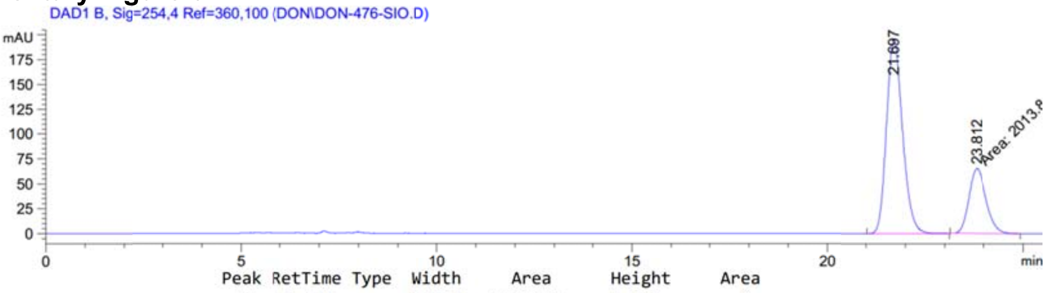

Peak #	RetTime [min]	Type	Width [min]	Area [mAU*s]	Height [mAU]	Area %
1	21.697	BB	0.4337	5521.98584	195.40106	73.2767
2	23.812	MM	0.5151	2013.80957	65.16019	26.7233

(*R*)-1-Phenylethan-1-ol [(*R*)-1b]

The enantiomeric excess of (*R*)-**1b** was determined by HPLC analysis on a chiral stationary phase (Daicel Chiralcel OD-H column, column temperature 20°C, solvent *n*-heptane:isopropanol = 95:5, flow rate 0.8 mL/min, λ = 210 nm): t_R = 11.2 min for (*R*)-**1b**, t_R = 13.0 min for (*S*)-**1b**.

Supplementary Figure 7. *rac-1-Phenylethan-1-ol [rac-**1b**]*

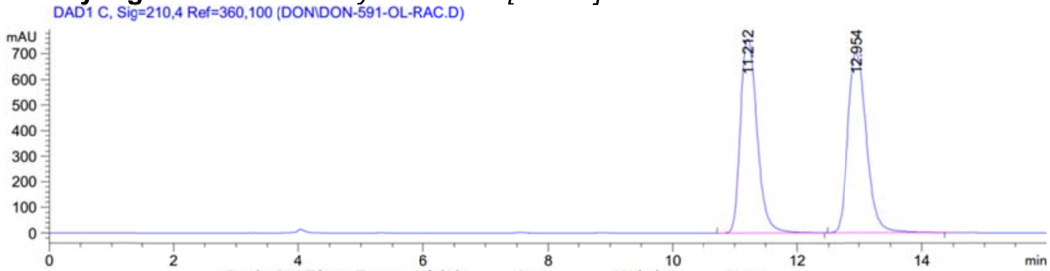

Peak #	RetTime [min]	Type	Width [min]	Area [mAU*s]	Height [mAU]	Area %
1	11.212	BB	0.2921	1.40485e4	758.58160	49.0139
2	12.954	BB	0.3286	1.46138e4	702.86237	50.9861

Supplementary Figure 8. *(R)-1-Phenylethan-1-ol [(R)-**1b**, 95.8% ee]*

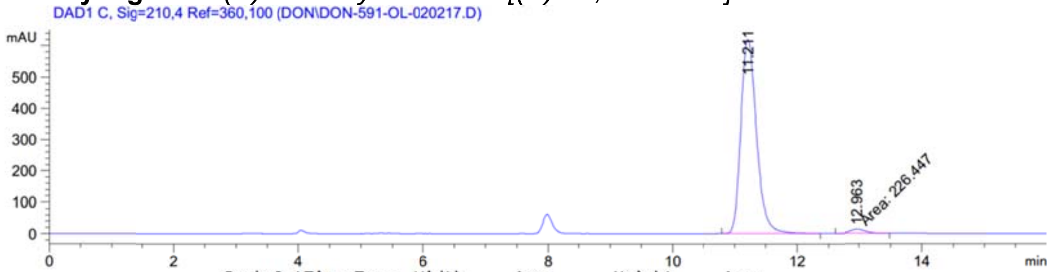

Peak #	RetTime [min]	Type	Width [min]	Area [mAU*s]	Height [mAU]	Area %
1	11.211	BB	0.2711	1.07989e4	620.16919	97.9461
2	12.963	MM	0.2862	226.44720	13.18532	2.0539

(*S*)-Tributyl(1-phenylethoxy)silane [(*S*)-3bh, 56.4% ee]

The enantiomeric excess of (*S*)-**3bh** was determined by the deprotected alcohol, which was hydrolyzed and purified by preparative TLC according to GP5, using the same HPLC analysis condition with **1b**.

Supplementary Figure 9.

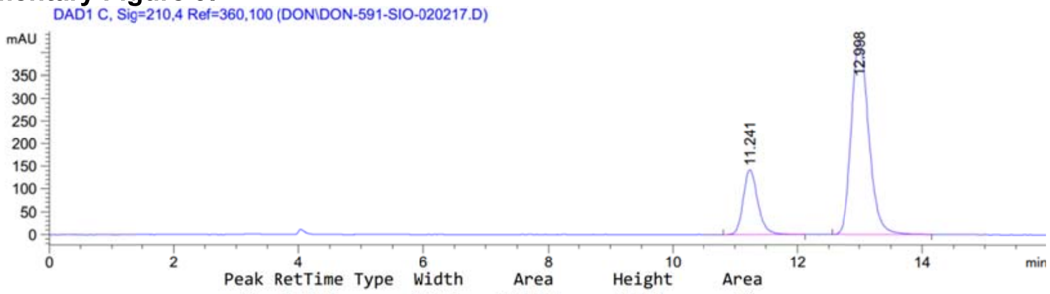

Peak #	RetTime [min]	Type	Width [min]	Area [mAU*s]	Height [mAU]	Area %
1	11.241	BB	0.2484	2311.75439	141.68758	21.8415
2	12.998	BV	0.3026	8272.45801	425.91367	78.1585

(*R*)-1-(*p*-Tolyl)ethan-1-ol [(*R*)-1c]

The enantiomeric excess of (*R*)-**1c** was determined by HPLC analysis on a chiral stationary phase (Daicel Chiralcel AD-H column, column temperature 20°C, solvent *n*-heptane:isopropanol = 98:2, flow rate 0.8 mL/min, λ = 210 nm): t_R = 27.3 min for (*R*)-**1c**, t_R = 29.0 min for (*S*)-**1c**.

Supplementary Figure 10. *rac-1-(p-Tolyl)ethan-1-ol [rac-**1c**]*

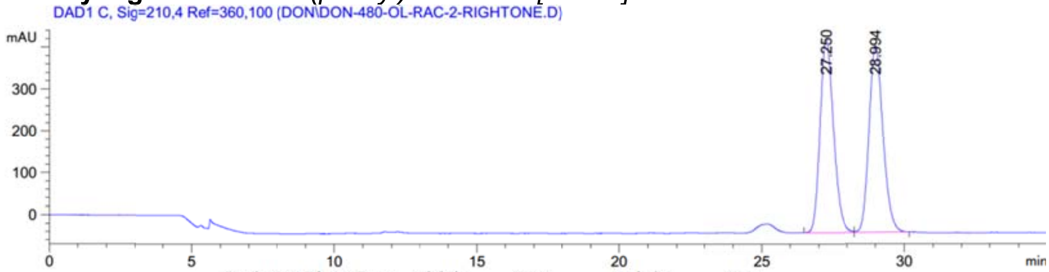

Peak #	RetTime [min]	Type	Width [min]	Area [mAU*s]	Height [mAU]	Area %
1	27.250	BV	0.5026	1.50366e4	460.94656	49.9608
2	28.994	VB	0.5127	1.50602e4	445.09924	50.0392

Supplementary Figure 11. *(R)-1-(p-Tolyl)ethan-1-ol [(R)-**1c**, 92.6% ee]*

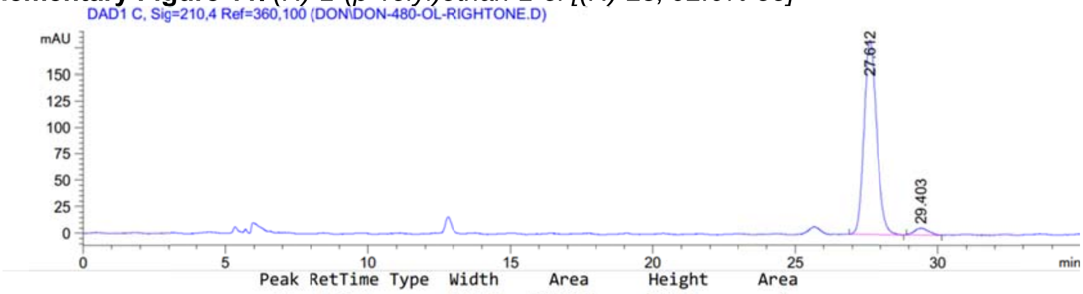

Peak #	RetTime [min]	Type	Width [min]	Area [mAU*s]	Height [mAU]	Area %
1	27.612	BB	0.4741	5714.33496	183.09381	96.3165
2	29.403	VB	0.4068	218.53525	6.36117	3.6835

(*S*)-Tributyl(1-(*p*-tolyl)ethoxy)silane [(*S*)-3ch, 64.4% ee]

The enantiomeric excess of (*S*)-**3ch** was determined after deprotection to the alcohol [according to GP5 without purification by preparative TLC] using the HPLC setup for **1c**.

Supplementary Figure 12.

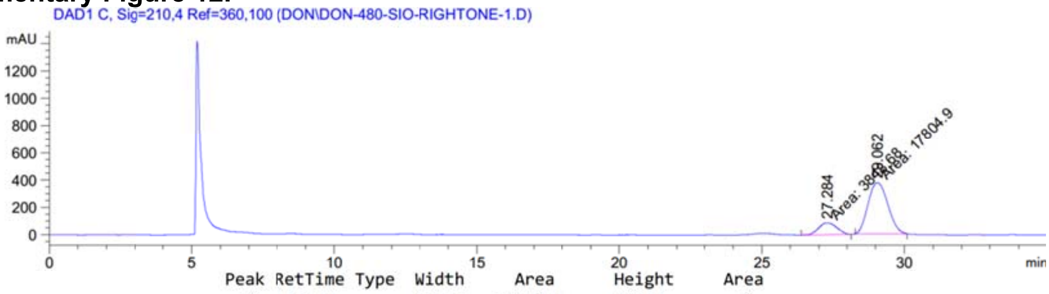

Peak #	RetTime [min]	Type	Width [min]	Area [mAU*s]	Height [mAU]	Area %
1	27.284	MM	0.7339	3848.68359	87.40350	17.7739
2	29.062	MM	0.7939	1.78049e4	373.79694	82.2261

(*R*)-1-(*m*-Tolyl)ethan-1-ol [(*R*)-1d]

The enantiomeric excess of (*R*)-**1d** was determined by HPLC analysis on a chiral stationary phase (Daicel Chiralcel OD-H column, column temperature 20°C, solvent *n*-heptane:isopropanol = 95:5, flow rate 0.8 mL/min, λ = 210 nm): t_R = 13.1 min for (*R*)-**1d**, t_R = 15.2 min for (*S*)-**1d.**

Supplementary Figure 13. *rac*-1-(*m*-Tolyl)ethan-1-ol *[rac-**1d**]*

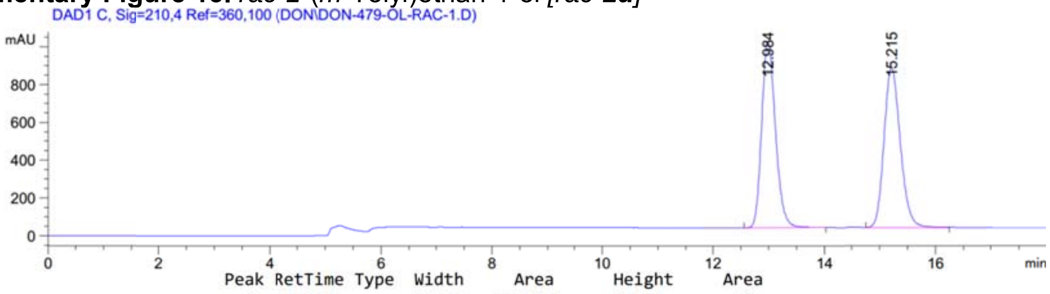

Peak #	RetTime [min]	Type	Width [min]	Area [mAU*s]	Height [mAU]	Area %
1	12.984	BB	0.2648	1.69587e4	985.41028	49.7824
2	15.215	BV	0.3142	1.71070e4	844.87933	50.2176

Supplementary Figure 14. *(R)-1-(m-Tolyl)ethan-1-ol [(R)-**1d**, 95.0% ee]*

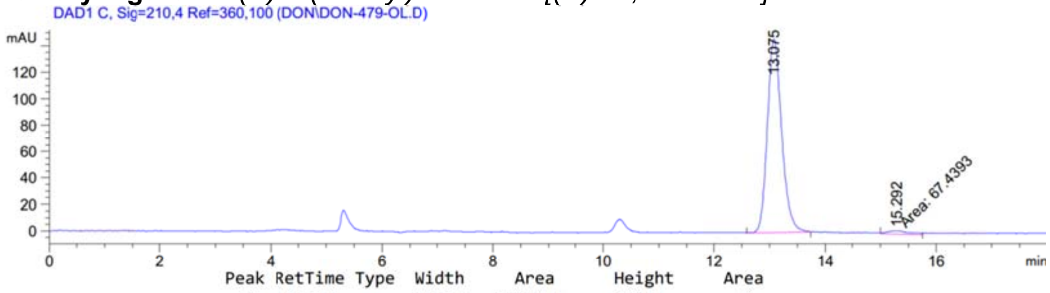

Peak #	RetTime [min]	Type	Width [min]	Area [mAU*s]	Height [mAU]	Area %
1	13.075	BB	0.2745	2607.45239	145.89946	97.4788
2	15.292	MM	0.4817	67.43927	2.33316	2.5212

(*S*)-Tributyl(1-(*m*-tolyl)ethoxy)silane [(*S*)-3dh, 64.8% ee]

The enantiomeric excess of (*S*)-**3dh** was determined after deprotection to the alcohol [according to GP5 without purification by preparative TLC] using the HPLC setup for **1d**.

Supplementary Figure 15.

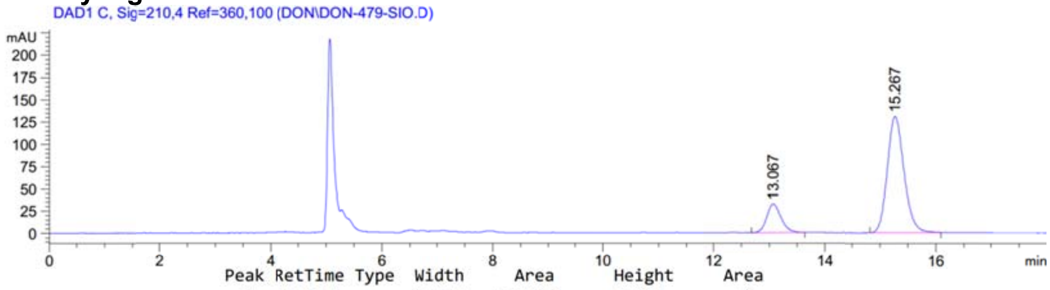

Peak #	RetTime [min]	Type	Width [min]	Area [mAU*s]	Height [mAU]	Area %
1	13.067	BB	0.2708	578.66400	32.32966	17.5594
2	15.267	BB	0.3145	2716.80640	130.67982	82.4406

(*R*)-1-(*o*-Tolyl)ethan-1-ol [(*R*)-1e]

The enantiomeric excess of (*R*)-**1e** was determined by HPLC analysis on a chiral stationary phase (Daicel Chiralcel AD-H column, column temperature 20°C, solvent *n*-heptane:isopropanol = 95:5, flow rate 0.8 mL/min, λ = 210 nm): t_R = 13.2 min for (*R*)-**1e**, t_R = 14.6 min for (*S*)-**1e.**

Supplementary Figure 16. *rac-1-(o-*Tolyl)ethan-1-ol *[rac-**1e**]*

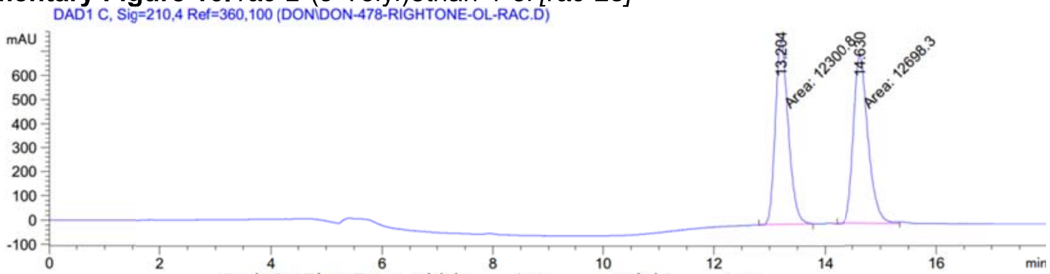

Peak #	RetTime [min]	Type	Width [min]	Area [mAU*s]	Height [mAU]	Area %
1	13.204	MM	0.2706	1.23008e4	757.60345	49.2049
2	14.630	MM	0.3044	1.26983e4	695.20978	50.7951

Supplementary Figure 17. *(R)-1-(o-Tolyl)ethan-1-ol [(R)-**1e**, 97.0% ee]*

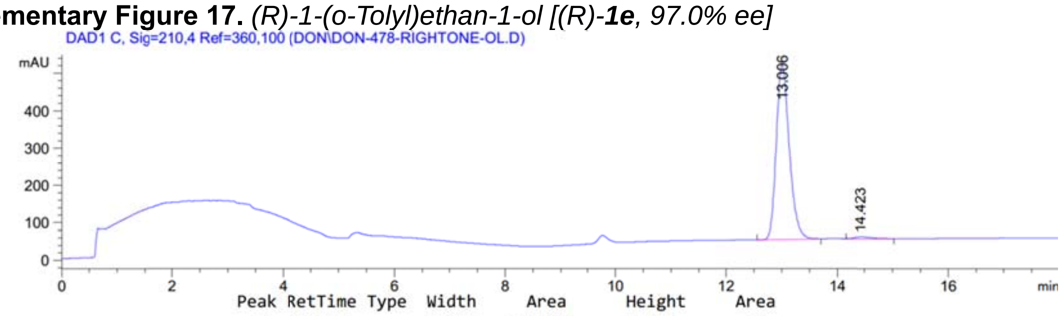

Peak #	RetTime [min]	Type	Width [min]	Area [mAU*s]	Height [mAU]	Area %
1	13.006	BV	0.2503	7652.31836	469.16125	98.4547
2	14.423	BB	0.2801	120.10396	5.22659	1.5453

(*S*)-Tributyl(1-(*o*-tolyl)ethoxy)silane [(*S*)-3eh, 60.8% ee]

The enantiomeric excess of (*S*)-**3eh** was determined after deprotection to the alcohol [according to GP5 without purification by preparative TLC] using the HPLC setup for **1e**.

Supplementary Figure 18.

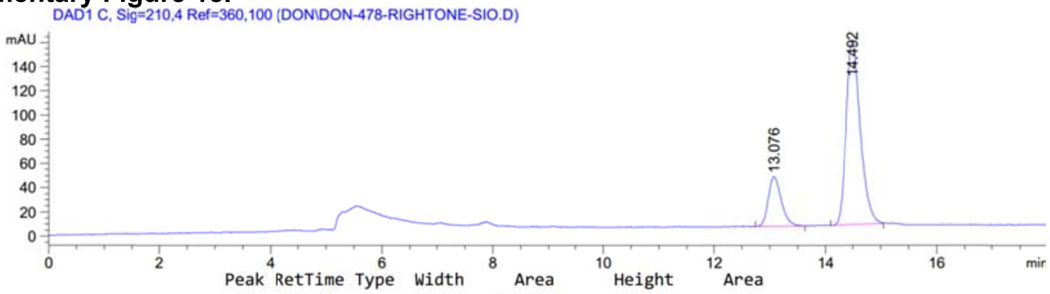

Peak #	RetTime [min]	Type	Width [min]	Area [mAU*s]	Height [mAU]	Area %
1	13.076	BB	0.2525	661.84686	40.53450	19.5783
2	14.492	BV	0.2711	2718.65991	151.69244	80.4217

(*R*)-1-(2,4-Dimethylphenyl)ethan-1-ol [(*R*)-1f]

The enantiomeric excess of (*R*)-**1f** was determined by HPLC analysis on a chiral stationary phase (Daicel Chiralcel OD-H column, column temperature 20°C, solvent *n*-heptane:isopropanol = 95:5, flow rate 0.8 mL/min, λ = 210 nm): t_R = 12.1 min for (*R*)-**1f**, t_R = 13.9 min for (*S*)-**1f.**

Supplementary Figure 19. *rac-1-(2,4-Dimethylphenyl)ethan-1-ol [rac-**1f**]*

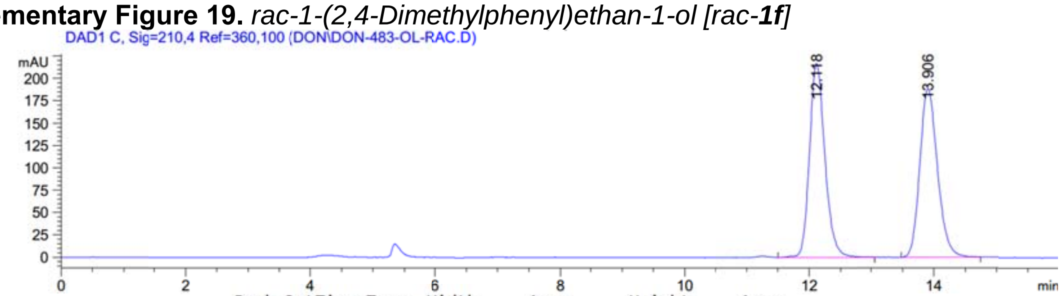

Peak #	RetTime [min]	Type	Width [min]	Area [mAU*s]	Height [mAU]	Area %
1	12.118	BV	0.2635	3705.60083	216.64926	50.1323
2	13.906	BV	0.2958	3686.03882	188.75594	49.8677

Supplementary Figure 20. *(R)-1-(2,4-Dimethylphenyl)ethan-1-ol [(R)-**1f**, 85.8% ee]*

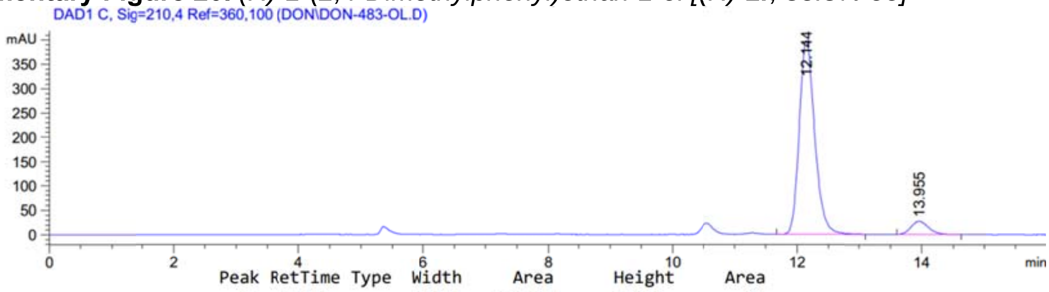

Peak #	RetTime [min]	Type	Width [min]	Area [mAU*s]	Height [mAU]	Area %
1	12.144	BV	0.2661	6845.89941	399.04556	92.8726
2	13.955	BB	0.2861	525.37695	27.10655	7.1274

(*S*)-Tributyl(1-(2,4-dimethylphenyl)ethoxy)silane [(*S*)-3fh, 72.2% ee]

The enantiomeric excess of (*S*)-**3fh** was determined after deprotection to the alcohol [according to GP5 without purification by preparative TLC] using the HPLC setup for **1f**.

Supplementary Figure 21.

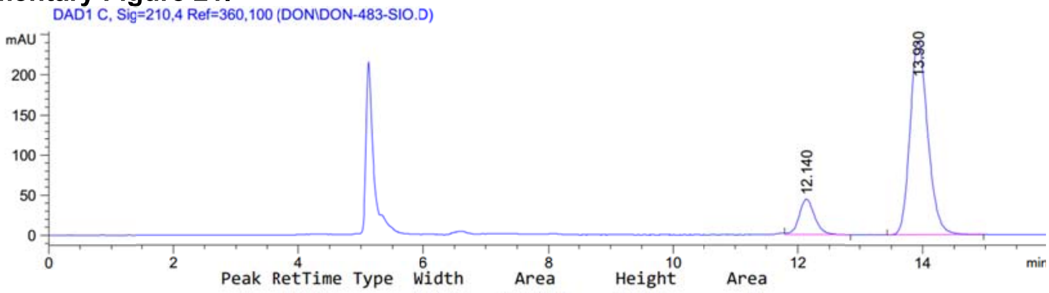

Peak #	RetTime [min]	Type	Width [min]	Area [mAU*s]	Height [mAU]	Area %
1	12.140	VB	0.2642	766.26770	44.21994	13.8980
2	13.930	BB	0.3030	4747.25000	241.83202	86.1020

(*R*)-1-(3,5-Dimethylphenyl)ethan-1-ol [(*R*)-1g]

The enantiomeric excess of (*R*)-**1g** was determined by HPLC analysis on a chiral stationary phase (Daicel Chiralcel OD-H column, column temperature 20°C, solvent *n*-heptane:isopropanol = 99:1, flow rate 0.8 mL/min, λ = 210 nm): t_R = 30.5 min for (*R*)-**1g**, t_R = 33.5 min for (*S*)-**1g.**

Supplementary Figure 22. *rac-1-(3,5-Dimethylphenyl)ethan-1-ol [rac-**1g**]*

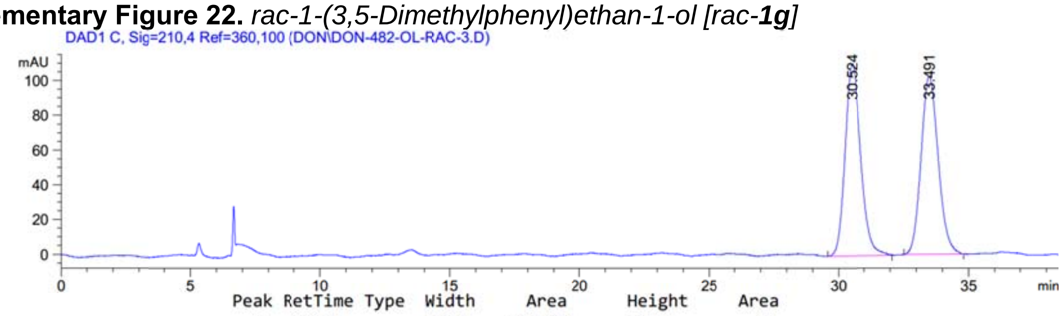

Peak #	RetTime [min]	Type	Width [min]	Area [mAU*s]	Height [mAU]	Area %
1	30.524	BV	0.5599	4745.46240	110.75079	50.4006
2	33.491	BB	0.6656	4670.01660	102.48130	49.5994

Supplementary Figure 23. *(R)-1-(3,5-Dimethylphenyl)ethan-1-ol [(R)-**1g**, 89.9% ee]*

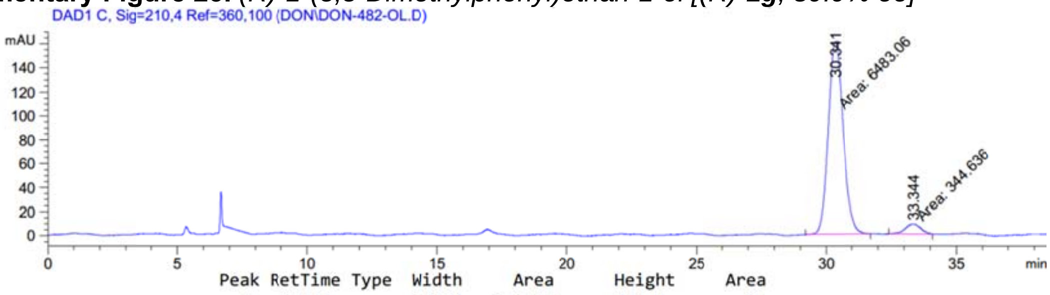

Peak #	RetTime [min]	Type	Width [min]	Area [mAU*s]	Height [mAU]	Area %
1	30.341	MM	0.6721	6483.05811	160.76421	94.9524
2	33.344	MM	0.6850	344.63565	8.38496	5.0476

(*S*)-Tributyl(1-(3,5-dimethylphenyl)ethoxy)silane [(*S*)-3gh, 73.2% ee]

The enantiomeric excess of (*S*)-**3gh** was determined after deprotection to the alcohol [according to GP5 without purification by preparative TLC] using the HPLC setup for **1g**.

Supplementary Figure 24.

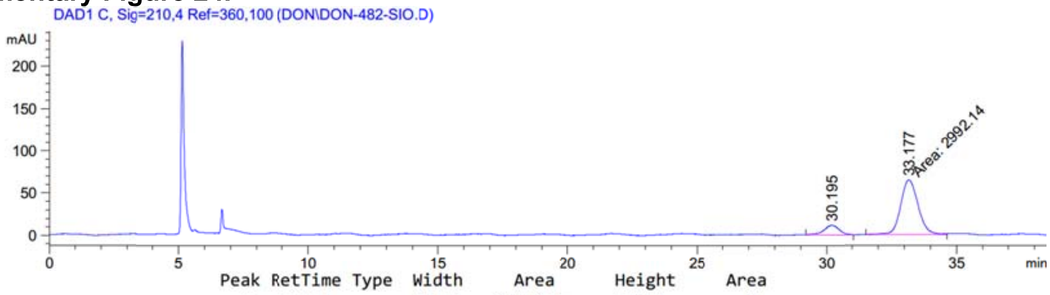

Peak #	RetTime [min]	Type	Width [min]	Area [mAU*s]	Height [mAU]	Area %
1	30.195	BB	0.5065	461.28369	11.38989	13.3573
2	33.177	MM	0.7768	2992.13965	64.19473	86.6427

(*R*)-1-(3,5-Bis(trifluoromethyl)phenyl)ethan-1-ol [(*R*)-1h]

The enantiomeric excess of (*R*)-**1h** was determined by HPLC analysis on a chiral stationary phase (Daicel Chiralcel OD-H column, column temperature 20°C, solvent *n*-heptane:isopropanol = 98:2, flow rate 0.8 mL/min, λ = 210 nm): t_R = 10.4 min for (*S*)-**1h**, t_R = 12.0 min for (*R*)-**1h.**

Supplementary Figure 25. *rac-1-(3,5-Bis(trifluoromethyl)phenyl)ethan-1-ol [rac-**1h**]*

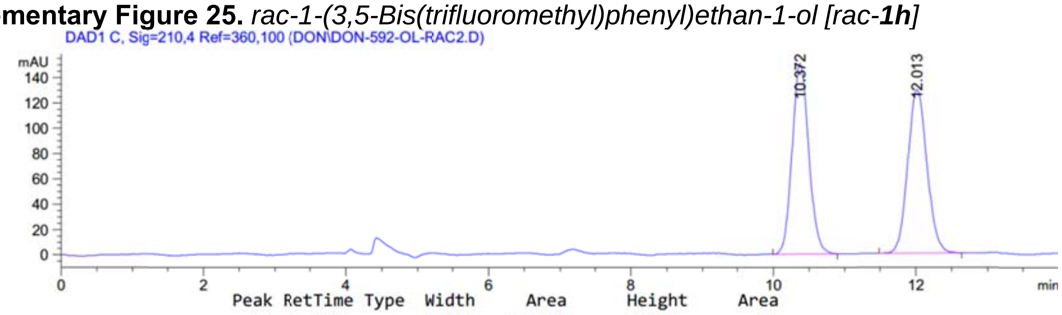

Peak #	RetTime [min]	Type	Width [min]	Area [mAU*s]	Height [mAU]	Area %
1	10.372	BV	0.2526	2432.84302	150.46361	50.2638
2	12.013	BB	0.2915	2407.30981	129.13702	49.7362

Supplementary Figure 26. *(R)-1-(3,5-Bis(trifluoromethyl)phenyl)ethan-1-ol [(R)-**1h**, 79.6% ee]*

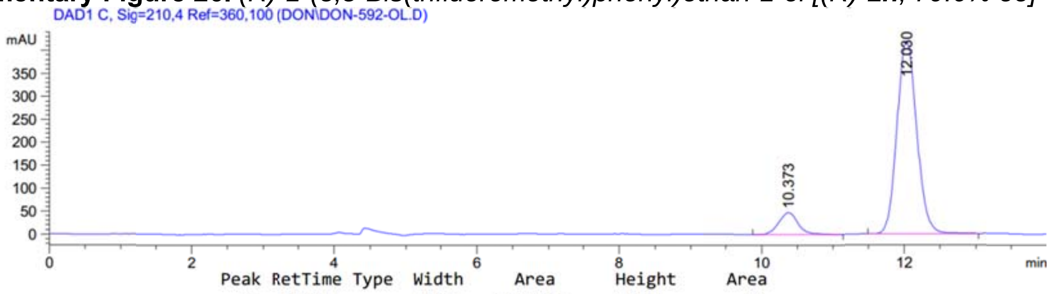

Peak #	RetTime [min]	Type	Width [min]	Area [mAU*s]	Height [mAU]	Area %
1	10.373	BB	0.2874	911.49786	48.03992	10.1996
2	12.030	BB	0.2990	8025.12402	419.77054	89.8004

(*S*)-1-(3,5-Bis(trifluoromethyl)phenyl)ethan-1-ol [(*S*)-3hh, 85.8% ee]

The enantiomeric excess of (*S*)-**3hh** was determined by the deprotected alcohol, which was hydrolyzed and purified by preparative TLC according to GP5, using the same HPLC analysis condition with **1h**.

Supplementary Figure 27.

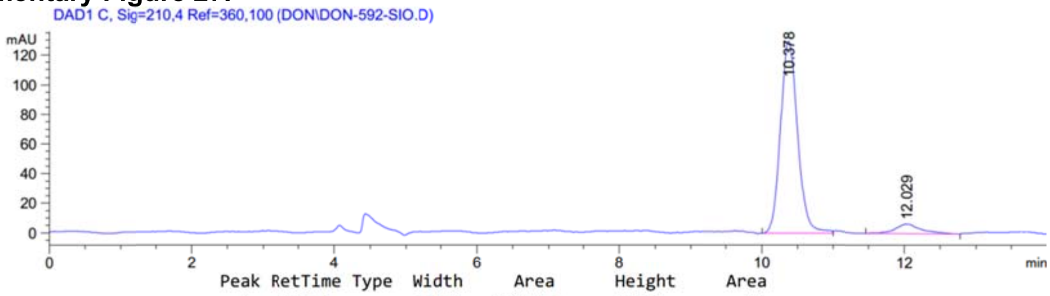

Peak #	RetTime [min]	Type	Width [min]	Area [mAU*s]	Height [mAU]	Area %
1	10.378	BV	0.2562	2164.58789	130.05791	92.8532
2	12.029	BB	0.3123	166.60596	6.51618	7.1468

(*R*)-1-(3,5-Dimethoxyphenyl)ethan-1-ol [(*R*)-1i]

The enantiomeric excess of (*R*)-**1f** was determined by HPLC analysis on a chiral stationary phase (Daicel Chiralcel OD-H column, column temperature 20°C, solvent *n*-heptane:isopropanol = 90:10, flow rate 0.8 mL/min, λ = 210 nm): t_R = 15.5 min for (*R*)-**1i**, t_R = 23.1 min for (*S*)-**1i.**

Supplementary Figure 28. *rac-1-(3,5-Dimethoxyphenyl)ethan-1-ol [rac-**1i**]*

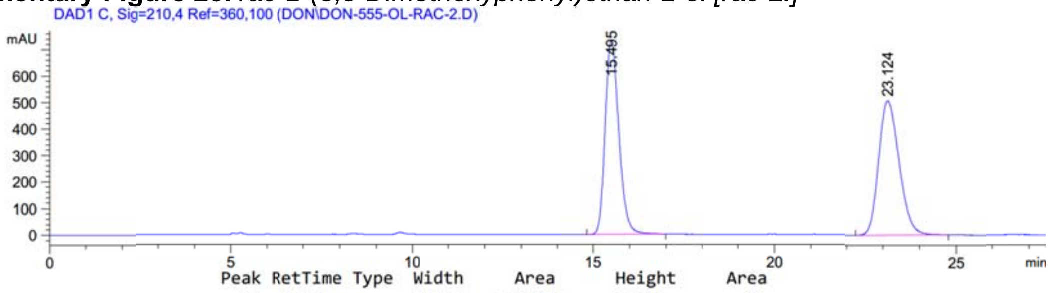

Peak #	RetTime [min]	Type	Width [min]	Area [mAU*s]	Height [mAU]	Area %
1	15.495	VV	0.4020	1.93015e4	735.80951	49.4392
2	23.124	BV	0.5801	1.97394e4	507.18835	50.5608

Supplementary Figure 29. *(R)-1-(3,5-Dimethoxyphenyl)ethan-1-ol [(R)-**1i**, 80.4% ee]*

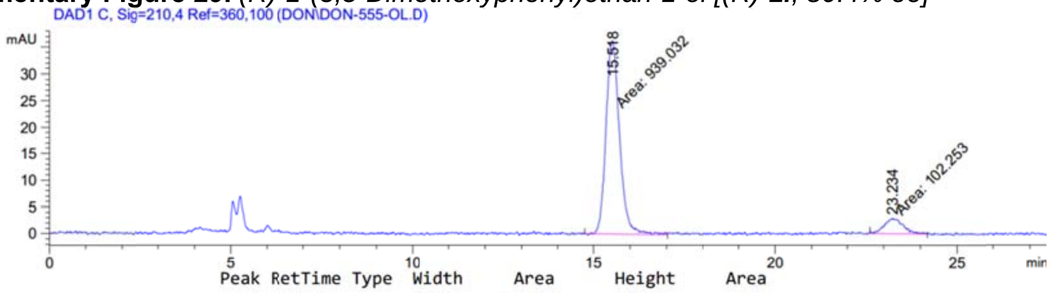

Peak #	RetTime [min]	Type	Width [min]	Area [mAU*s]	Height [mAU]	Area %
1	15.518	MM	0.4303	939.03204	36.37248	90.1801
2	23.234	MM	0.6011	102.25291	2.83524	9.8199

(*S*)-Tributyl(1-(3,5-dimethoxyphenyl)ethoxy)silane [(*S*)-3ih, 91.4% ee]

The enantiomeric excess of (*S*)-**3ih** was determined by the deprotected alcohol, which was hydrolyzed and purified by preparative TLC according to GP5, using the same HPLC analysis condition with **1i**.

Supplementary Figure 30.

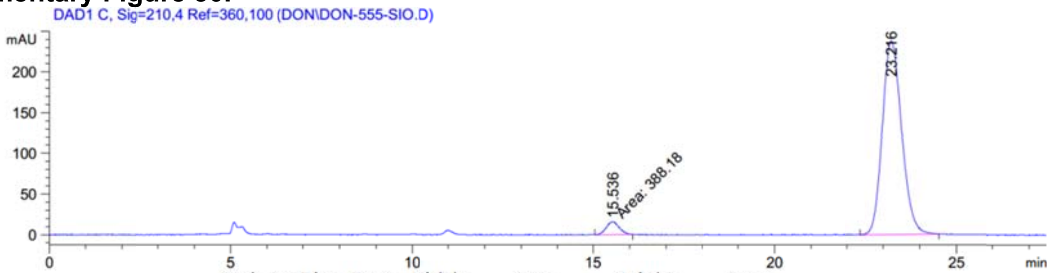

Peak #	RetTime [min]	Type	Width [min]	Area [mAU*s]	Height [mAU]	Area %
1	15.536	MM	0.4104	388.18036	15.76505	4.2622
2	23.216	VB	0.5736	8719.24707	237.89333	95.7378

(*R*)-1-(2,6-Dimethylphenyl)ethan-1-ol [(*R*)-1j]

The enantiomeric excess of (*R*)-**1j** was determined by HPLC analysis on a chiral stationary phase (Daicel Chiralcel OD-H column, column temperature 20°C, solvent *n*-heptane:isopropanol = 95:5, flow rate 0.8 mL/min, λ = 210 nm): t_R = 8.8 min for (*S*)-**1j**, t_R = 10.9 min for (*R*)-**1j.**

Supplementary Figure 31. *rac-1-(2,4-Dimethylphenyl)ethan-1-ol [rac-**1j**]*

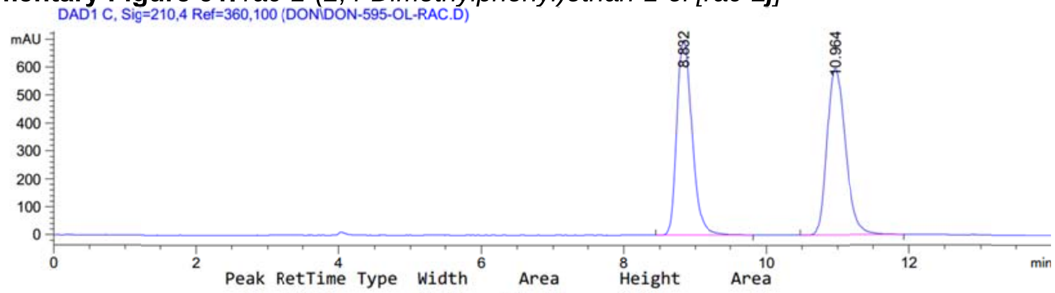

Peak #	RetTime [min]	Type	Width [min]	Area [mAU*s]	Height [mAU]	Area %
1	8.832	BB	0.2340	1.03493e4	694.11450	48.9189
2	10.964	BB	0.2840	1.08067e4	594.75574	51.0811

Supplementary Figure 32. *(R)-1-(2,6-Dimethylphenyl)ethan-1-ol [(R)-**1j**, 99.6% ee]*

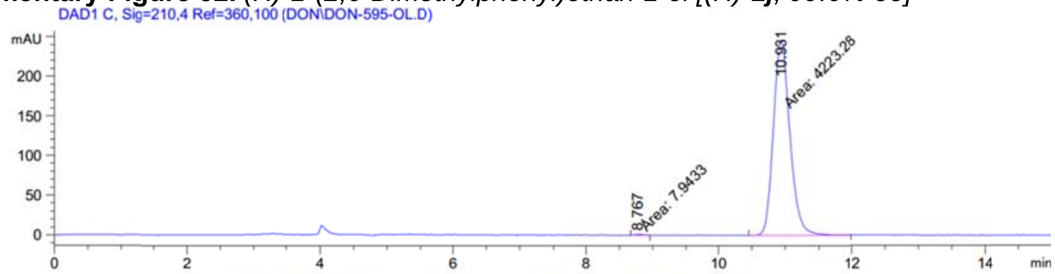

Peak #	RetTime [min]	Type	Width [min]	Area [mAU*s]	Height [mAU]	Area %
1	8.767	MM	0.1614	7.94330	8.20079e-1	0.1877
2	10.931	MM	0.2869	4223.27539	245.31996	99.8123

(*S*)-Tributyl(1-(2,6-dimethylphenyl)ethoxy)silane [(*S*)-3jh, 89.2% ee]

The enantiomeric excess of (*S*)-**3jh** was determined by the deprotected alcohol, which was hydrolyzed and purified by preparative TLC according to GP5, using the same HPLC analysis condition with **1j**.

Supplementary Figure 33.

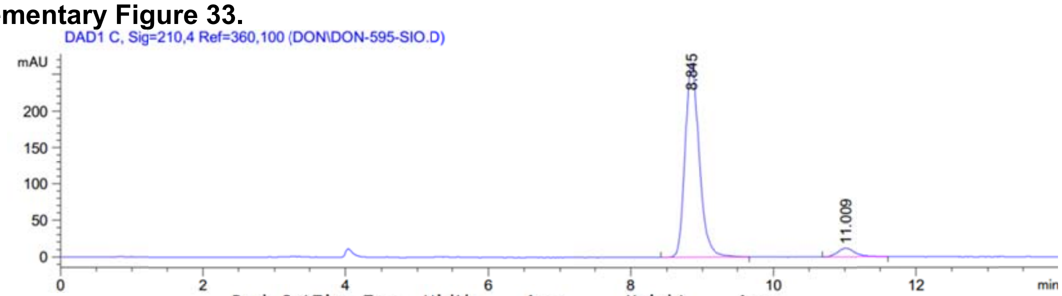

Peak #	RetTime [min]	Type	Width [min]	Area [mAU*s]	Height [mAU]	Area %
1	8.845	BB	0.2103	3646.99316	265.35968	94.5987
2	11.009	BB	0.2592	208.23288	11.83988	5.4013

(*R*)-1-(2,6-Dimethylphenyl)ethan-1-ol [(*R*)-1j]

The enantiomeric excess of (*R*)-**1j** was determined by HPLC analysis on a chiral stationary phase (Daicel Chiralcel OD-H column, column temperature 20°C, solvent *n*-heptane:isopropanol = 95:5, flow rate 0.8 mL/min, λ = 210 nm): t_R = 11.8 min for (*S*)-**1j**, t_R = 14.7 min for (*R*)-**1j.**

Supplementary Figure 34. *rac-1-(2,4-Dimethylphenyl)ethan-1-ol [rac-**1j**]*

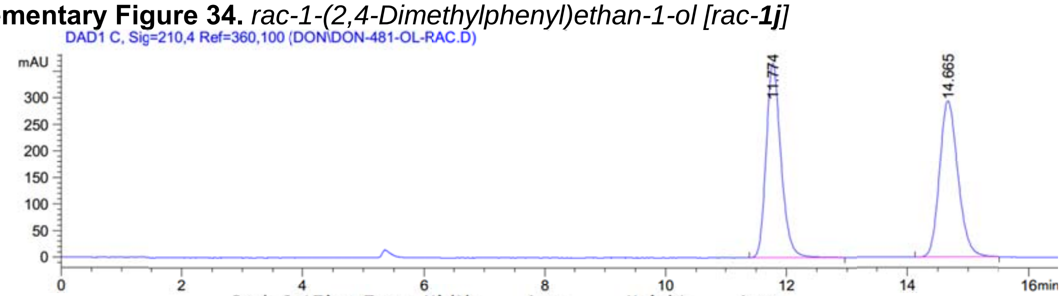

Peak #	RetTime [min]	Type	Width [min]	Area [mAU*s]	Height [mAU]	Area %
1	11.774	BB	0.2597	6179.17090	364.55865	50.0327
2	14.665	BB	0.3246	6171.10400	294.28949	49.9673

Supplementary Figure 35. *(R)-1-(2,6-Dimethylphenyl)ethan-1-ol [(R)-**1j**, 91.4% ee]*

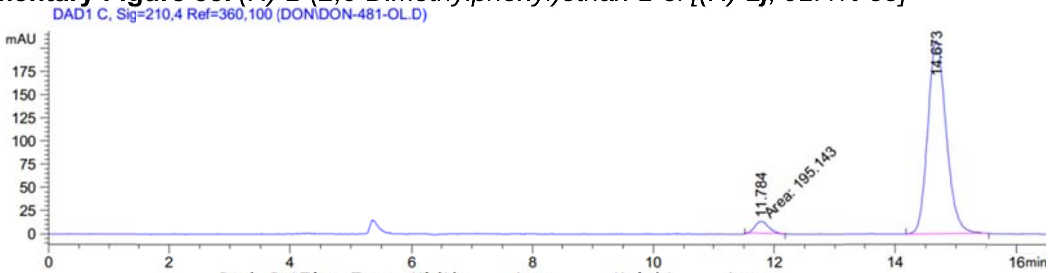

Peak #	RetTime [min]	Type	Width [min]	Area [mAU*s]	Height [mAU]	Area %
1	11.784	MM	0.2540	195.14308	12.80697	4.2833
2	14.673	BB	0.3182	4360.79053	208.35063	95.7167

(*S*)-Tributyl(1-(2,6-dimethylphenyl)ethoxy)silane [(*S*)-3jh, 96.2% ee]

The enantiomeric excess of (*S*)-**3jh** was determined after deprotection to the alcohol [according to GP5 without purification by preparative TLC] using the HPLC setup for **1j**.

Supplementary Figure 36.

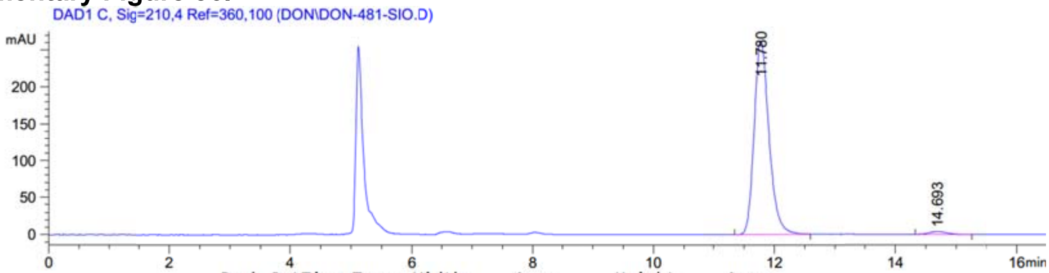

Peak #	RetTime [min]	Type	Width [min]	Area [mAU*s]	Height [mAU]	Area %
1	11.780	BB	0.2582	4452.54785	262.15219	98.0970
2	14.693	VB	0.2508	86.37435	4.18394	1.9030

(*R*)-1-Mesitylethan-1-ol [(*R*)-1k]

The enantiomeric excess of (*R*)-**1k** was determined by HPLC analysis on a chiral stationary phase (Daicel Chiralcel OD-H column, column temperature 20°C, solvent *n*-heptane:isopropanol = 99:1, flow rate 0.8 mL/min, λ = 210 nm): t_R = 29.2 min for (*R*)-**1k**, t_R = 34.3 min for (*S*)-**1k.**

Supplementary Figure 37. *rac-1-Mesitylethan-1-ol [rac-**1k**]*

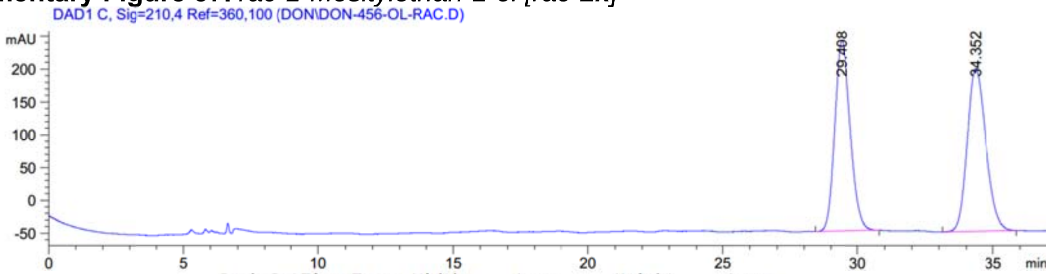

Peak #	RetTime [min]	Type	Width [min]	Area [mAU*s]	Height [mAU]	Area %
1	29.408	BB	0.6302	1.18789e4	289.83292	49.5613
2	34.352	BB	0.7446	1.20892e4	248.04256	50.4387

Supplementary Figure 38. *(R)-1-Mesitylethan-1-ol [(R)-**1k**, 99.6% ee]*

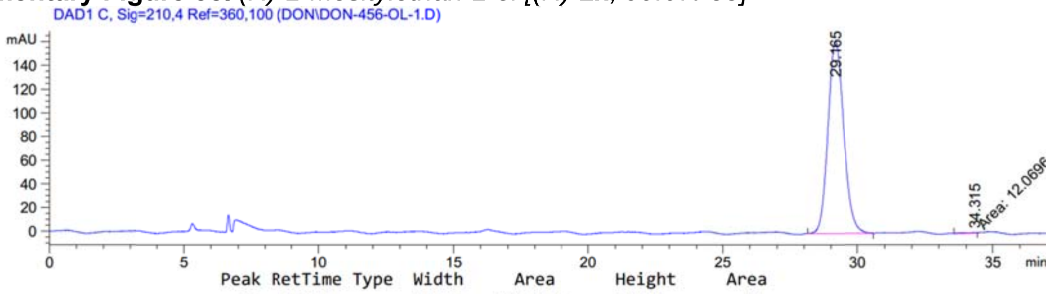

Peak #	RetTime [min]	Type	Width [min]	Area [mAU*s]	Height [mAU]	Area %
1	29.165	BB	0.6188	6681.38574	162.81244	99.8197
2	34.315	MM	0.6367	12.06956	3.15928e-1	0.1803

(*S*)-Tributyl(mesitylethoxy)silane [(*S*)-3kh, 81.8% ee]

The enantiomeric excess of (*S*)-**3kh** was determined after deprotection to the alcohol [according to GP5 without purification by preparative TLC] using the HPLC setup for **1k**.

Supplementary Figure 39.

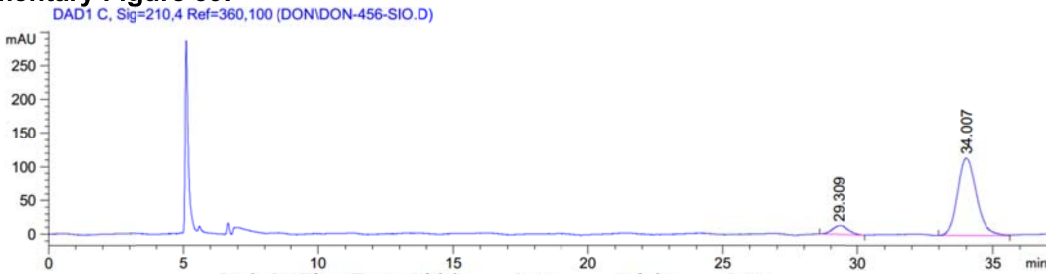

Peak #	RetTime [min]	Type	Width [min]	Area [mAU*s]	Height [mAU]	Area %
1	29.309	BB	0.5054	557.24982	13.22919	9.0509
2	34.007	BB	0.6574	5599.56445	114.72355	90.9491

(*R*)-1-Mesitylethan-1-ol [(*R*)-1k]

The enantiomeric excess of (*R*)-**1k** was determined by HPLC analysis on a chiral stationary phase (Daicel Chiralcel OD-H column, column temperature 20°C, solvent *n*-heptane:isopropanol = 95:5, flow rate 0.8 mL/min, λ = 210 nm): t_R = 9.2 min for (*R*)-**1k**, t_R = 10.7 min for (*S*)-**1k.**

Supplementary Figure 40. *rac-1-Mesitylethan-1-ol [rac-**1k**]*

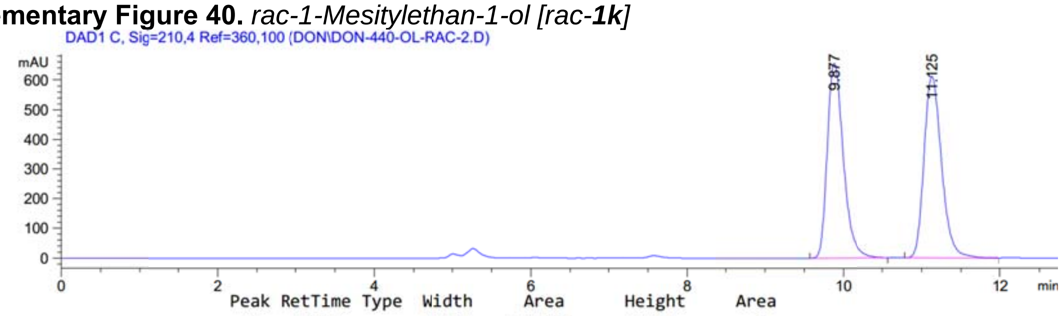

Peak #	RetTime [min]	Type	Width [min]	Area [mAU*s]	Height [mAU]	Area %
1	9.877	BB	0.1988	3497.42139	267.11661	50.0718
2	11.125	BB	0.2191	3487.39624	243.53241	49.9282

Supplementary Figure 41. *(R)-1-Mesitylethan-1-ol [(R)-**1k**, 97.4% ee]*

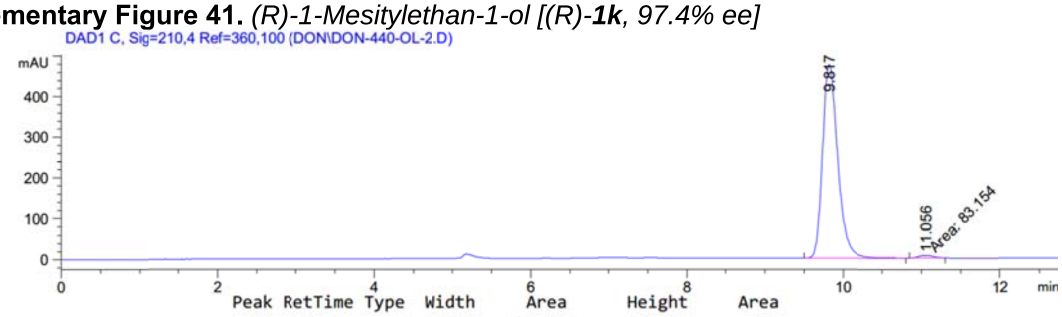

Peak #	RetTime [min]	Type	Width [min]	Area [mAU*s]	Height [mAU]	Area %
1	9.817	BV	0.2078	6442.30273	476.30615	98.7257
2	11.056	MM	0.2117	83.15401	6.54587	1.2743

(*S*)-Tributyl(mesitylethoxy)silane [(*S*)-3kh, 92.4% ee]

The enantiomeric excess of (*S*)-**3kh** was determined after deprotection to the alcohol [according to GP5 without purification by preparative TLC] using the HPLC setup for **1k**.

Supplementary Figure 42.

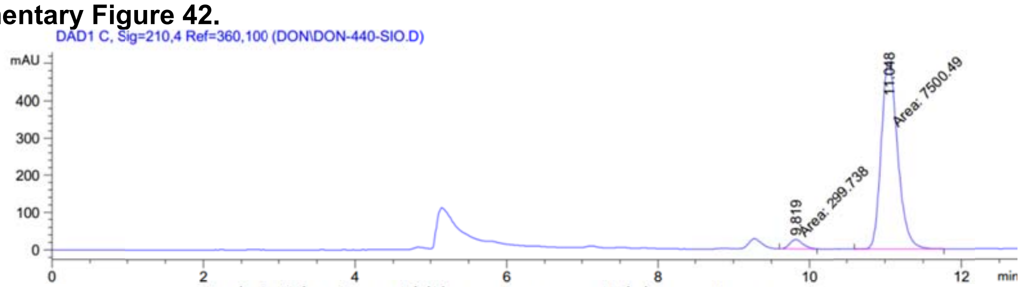

Peak #	RetTime [min]	Type	Width [min]	Area [mAU*s]	Height [mAU]	Area %
1	9.819	MM	0.1999	299.73770	24.99540	3.8427
2	11.048	MM	0.2485	7500.49023	503.03702	96.1573

(*R*)-1-([1,1'-Biphenyl]-4-yl)propan-1-ol [(*R*)-1l]

The enantiomeric excess of (*R*)-**1l** was determined by HPLC analysis on a chiral stationary phase (Daicel Chiralcel AD-H column, column temperature 20°C, solvent *n*-heptane:isopropanol = 95:5, flow rate 0.8 mL/min, λ = 250 nm): t_R = 22.9 min for (*S*)-**1l**, t_R = 25.4 min for (*R*)-**1l.**

Supplementary Figure 43. *rac-1-([1,1'-Biphenyl]-4-yl)propan-1-ol [rac-**1l**]*

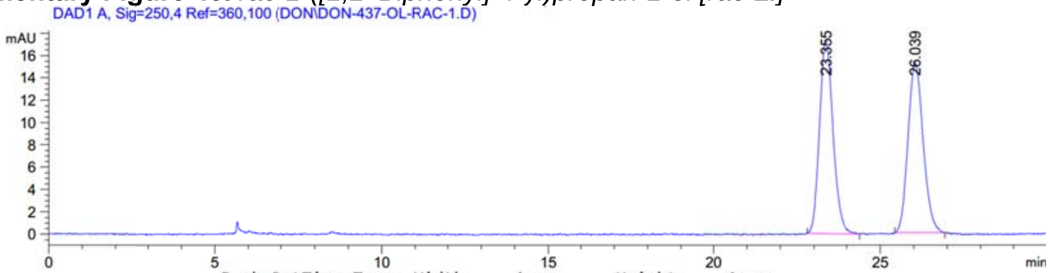

Peak #	RetTime [min]	Type	Width [min]	Area [mAU*s]	Height [mAU]	Area %
1	23.355	BB	0.4074	488.74472	17.30880	50.4602
2	26.039	BB	0.4563	479.83075	15.28192	49.5398

Supplementary Figure 44. *(R)-1-([1,1'-Biphenyl]-4-yl)propan-1-ol [(R)-**1l**, 76.4% ee]*

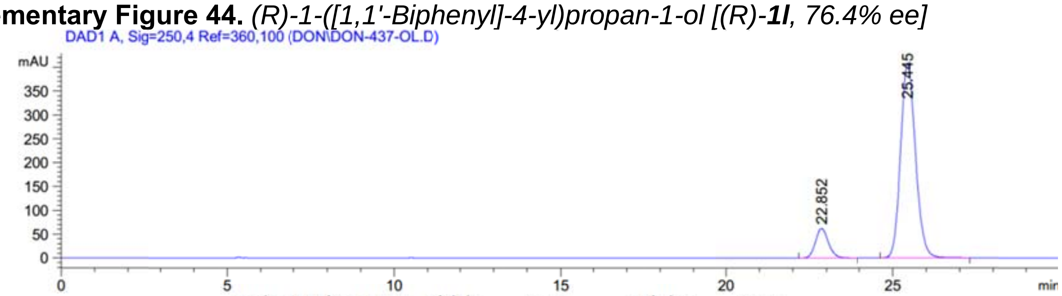

Peak #	RetTime [min]	Type	Width [min]	Area [mAU*s]	Height [mAU]	Area %
1	22.852	BB	0.4139	1680.03198	62.45081	11.7792
2	25.445	BB	0.4774	1.25826e4	408.47974	88.2208

(*S*)-(1-([1,1'-Biphenyl]-4-yl)propoxy)tributylsilane [(*S*)-3lh, 67.8% ee]

The enantiomeric excess of (*S*)-**3lh** was determined by the deprotected alcohol, which was hydrolyzed and purified by preparative TLC according to GP5, using the same HPLC analysis condition with **1l**.

Supplementary Figure 45.

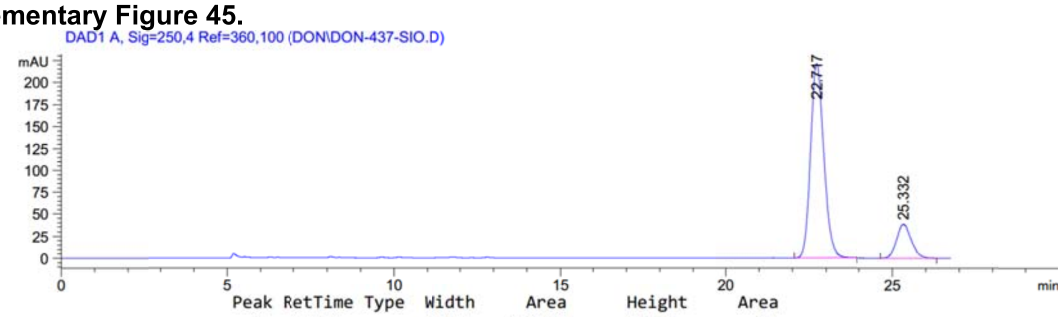

Peak #	RetTime [min]	Type	Width [min]	Area [mAU*s]	Height [mAU]	Area %
1	22.717	BB	0.4255	6091.47217	221.01631	83.9123
2	25.332	BB	0.4578	1167.85889	38.08024	16.0877

(*R*)-1,2-Diphenylethan-1-ol [(*R*)-1m]

The enantiomeric excess of (*R*)-**1m** was determined by HPLC analysis on a chiral stationary phase (Daicel Chiralcel OD-H column, column temperature 20°C, solvent *n*-heptane:isopropanol = 95:5, flow rate 0.8 mL/min, λ = 210 nm): t_R = 20.8 min for (*R*)-**1m**, t_R = 24.7 min for (*S*)-**1m.**

Supplementary Figure 46. *rac-1,2-Diphenylethan-1-ol [rac-**1m**]*

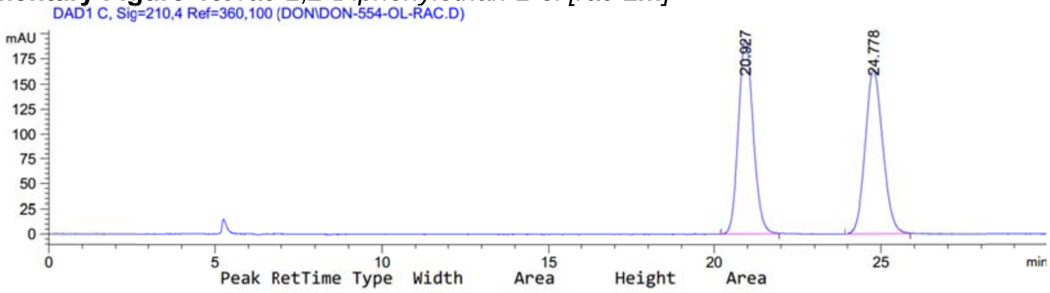

Peak #	RetTime [min]	Type	Width [min]	Area [mAU*s]	Height [mAU]	Area %
1	20.927	BB	0.4698	5950.54541	194.00725	49.9011
2	24.778	BV	0.5408	5974.12695	163.30875	50.0989

Supplementary Figure 47. *(R)-1,2-Diphenylethan-1-ol [(R)-**1m**, 54.4% ee]*

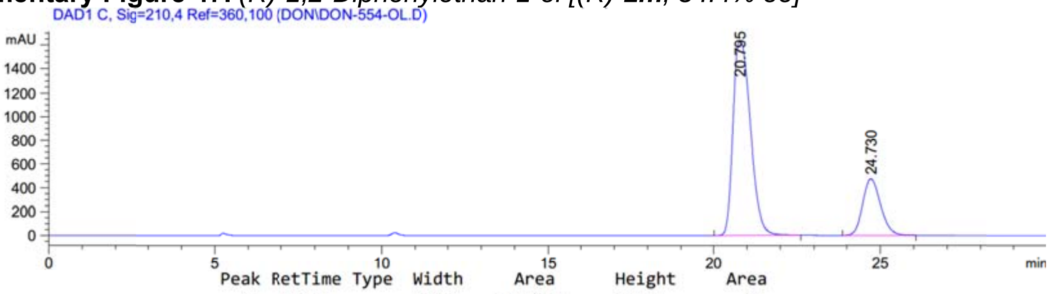

Peak #	RetTime [min]	Type	Width [min]	Area [mAU*s]	Height [mAU]	Area %
1	20.795	BV	0.4392	5.92688e4	1634.03857	77.1918
2	24.730	BV	0.5646	1.75124e4	472.29227	22.8082

(*S*)-Tributyl(1,2-diphenylethoxy)silane [(*S*)-3mh, 70.6% ee]

The enantiomeric excess of (*S*)-**3mh** was determined by the deprotected alcohol, which was hydrolyzed and purified by preparative TLC according to GP5, using the same HPLC analysis condition with **1m**.

Supplementary Figure 48.

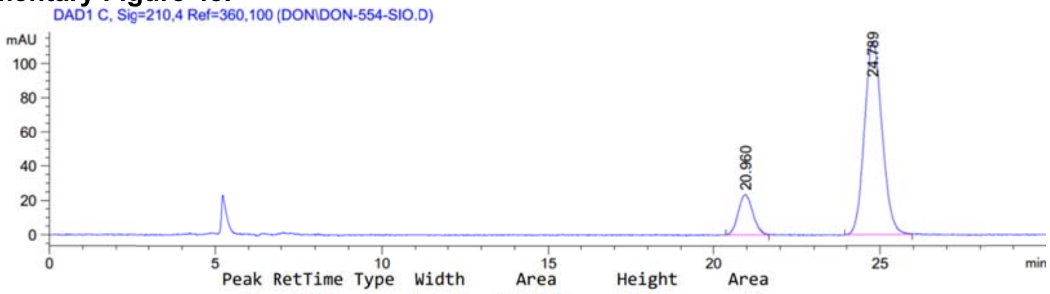

Peak #	RetTime [min]	Type	Width [min]	Area [mAU*s]	Height [mAU]	Area %
1	20.960	BV	0.3699	708.29449	23.48533	14.6709
2	24.789	BB	0.5115	4119.59668	112.95312	85.3291

(*R*)-1-([1,1'-Biphenyl]-4-yl)-2-methylpropan-1-ol [(*R*)-1n]

The enantiomeric excess of (*R*)-**1n** was determined by HPLC analysis on a chiral stationary phase (Daicel Chiralcel AD-H column, column temperature 20°C, solvent *n*-heptane:isopropanol = 95:5, flow rate 0.8 mL/min, λ = 250 nm): t_R = 20.2 min for (*S*)-**1n**, t_R = 24.1 min for (*R*)-**1n.**

Supplementary Figure 49. *rac-1-([1,1'-Biphenyl]-4-yl)-2-methylpropan-1-ol [rac-**1n**]*

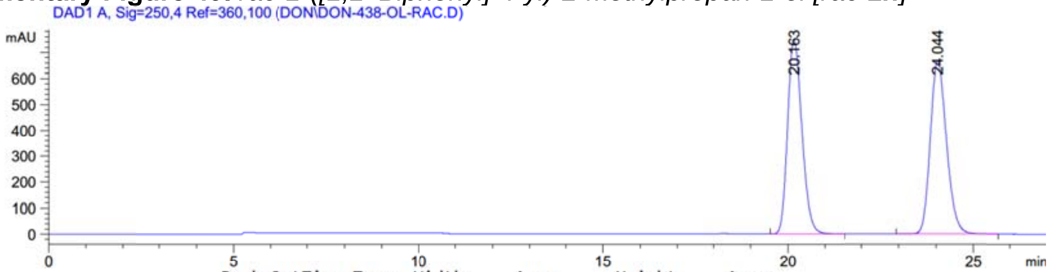

Peak #	RetTime [min]	Type	Width [min]	Area [mAU*s]	Height [mAU]	Area %
1	20.163	BB	0.4199	2.00480e4	749.78046	49.8509
2	24.044	BB	0.4690	2.01679e4	666.56250	50.1491

Supplementary Figure 50. *(R)-1-([1,1'-Biphenyl]-4-yl)-2-methylpropan-1-ol [(R)-**1n**, 34.0% ee]*

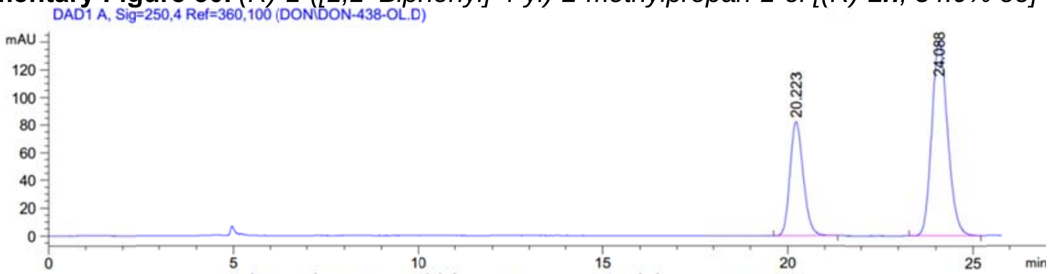

Peak #	RetTime [min]	Type	Width [min]	Area [mAU*s]	Height [mAU]	Area %
1	20.223	BB	0.3815	2052.75244	82.71884	33.0432
2	24.088	BB	0.4588	4159.58447	140.73891	66.9568

(*S*)-(1-([1,1'-Biphenyl]-4-yl)-2-methylpropoxy)tributylsilane [(*S*)-3nh, 37.4% ee]

The enantiomeric excess of (*S*)-**3nh** was determined by the deprotected alcohol, which was hydrolyzed and purified by preparative TLC according to GP5, using the same HPLC analysis condition with **1n**.

Supplementary Figure 51.

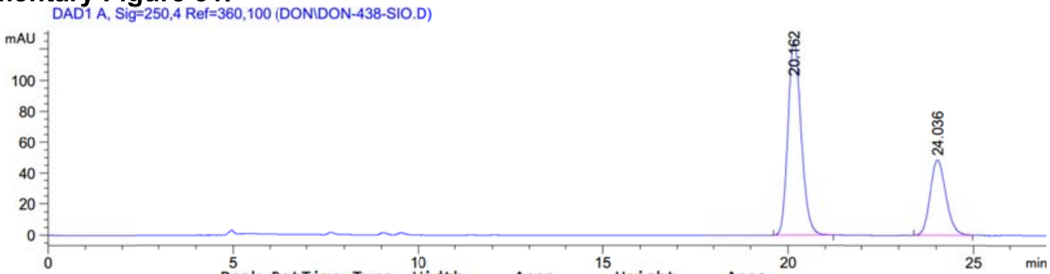

Peak #	RetTime [min]	Type	Width [min]	Area [mAU*s]	Height [mAU]	Area %
1	20.162	BB	0.3828	3114.95874	125.85235	68.7433
2	24.036	BB	0.4515	1416.33472	48.38800	31.2567

(*R*)-1-Mesitylpropan-1-ol [(*R*)-1o]

The enantiomeric excess of (*R*)-**1o** was determined by HPLC analysis on a chiral stationary phase (Daicel Chiralcel AD-H column, column temperature 20°C, solvent *n*-heptane:isopropanol = 95:5, flow rate 0.8 mL/min, λ = 230 nm): t_R = 7.1 min for (*R*)-**1o**, t_R = 8.2 min for (*S*)-**1o**.

Supplementary Figure 52. *rac-1-Mesitylpropan-1-ol [rac-**1o**]*

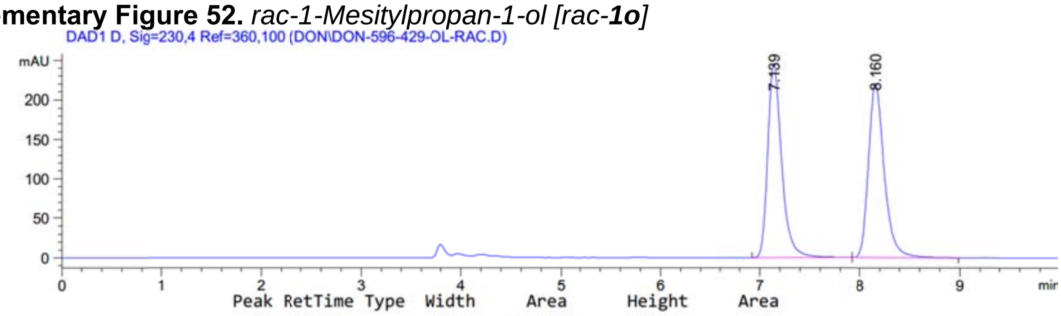

Peak #	RetTime [min]	Type	Width [min]	Area [mAU*s]	Height [mAU]	Area %
1	7.139	BB	0.1450	2387.39600	246.51132	49.8552
2	8.160	BB	0.1652	2401.26294	220.19409	50.1448

Supplementary Figure 53. *(R)-1-1-Mesitylpropan-1-ol [(R)-**1o**, 99.6% ee]*

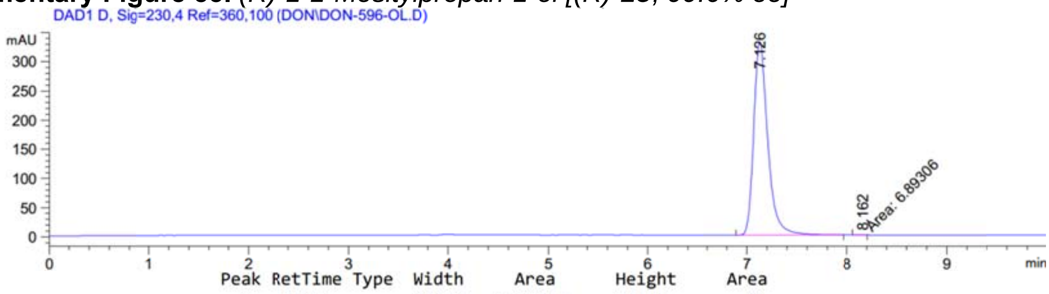

Peak #	RetTime [min]	Type	Width [min]	Area [mAU*s]	Height [mAU]	Area %
1	7.126	BB	0.1475	3217.30103	330.69406	99.7862
2	8.162	MM	0.1367	6.89306	8.40395e-1	0.2138

(*S*)-Tributyl(1-mesitylpropoxy)silane [(*S*)-3oh, 84.2% ee]

The enantiomeric excess of (*S*)-**3oh** was determined by the deprotected alcohol, which was hydrolyzed and purified by preparative TLC according to GP5, using the same HPLC analysis condition with **1o**.

Supplementary Figure 54.

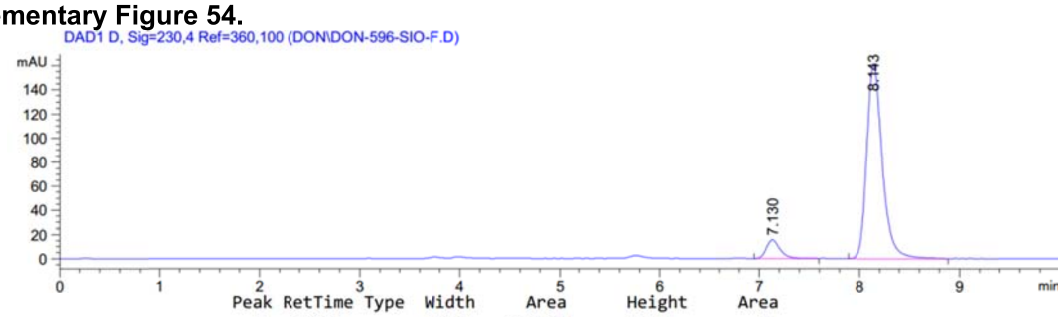

Peak #	RetTime [min]	Type	Width [min]	Area [mAU*s]	Height [mAU]	Area %
1	7.130	BB	0.1435	149.40196	15.63791	7.8624
2	8.143	BB	0.1643	1750.81299	161.78409	92.1376

(*R*)-1-Mesitylpropan-1-ol [(*R*)-1o]

The enantiomeric excess of (*R*)-**1o** was determined by HPLC analysis on a chiral stationary phase (Daicel Chiralcel AD-H column, column temperature 20°C, solvent *n*-heptane:isopropanol = 95:5, flow rate 0.8 mL/min, λ = 210 nm): t_R = 9.4 min for (*R*)-**1o**, t_R = 10.7 min for (*S*)-**1o**.

Supplementary Figure 55. *rac-1-Mesitylpropan-1-ol [rac-**1o**]*

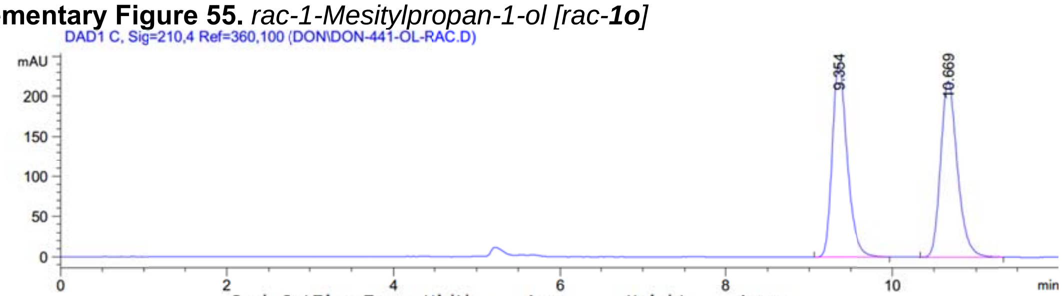

Peak #	RetTime [min]	Type	Width [min]	Area [mAU*s]	Height [mAU]	Area %
1	9.354	BB	0.1900	3037.96143	242.72441	49.8320
2	10.669	BB	0.2123	3058.44531	219.85468	50.1680

Supplementary Figure 56. *(R)-1-1-Mesitylpropan-1-ol [(R)-**1o**, 95.0% ee]*

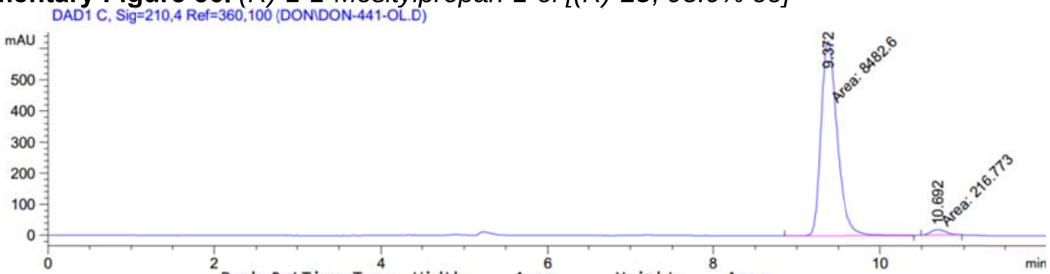

Peak #	RetTime [min]	Type	Width [min]	Area [mAU*s]	Height [mAU]	Area %
1	9.372	MM	0.2266	8482.60449	623.87738	97.5082
2	10.692	MM	0.2041	216.77312	17.70208	2.4918

(*S*)-Tributyl(1-mesitylpropoxy)silane [(*S*)-3oh, 91.2% ee]

The enantiomeric excess of (*S*)-**3oh** was determined by the deprotected alcohol, which was hydrolyzed and purified by preparative TLC according to GP5, using the same HPLC analysis condition with **1o**.

Supplementary Figure 57.

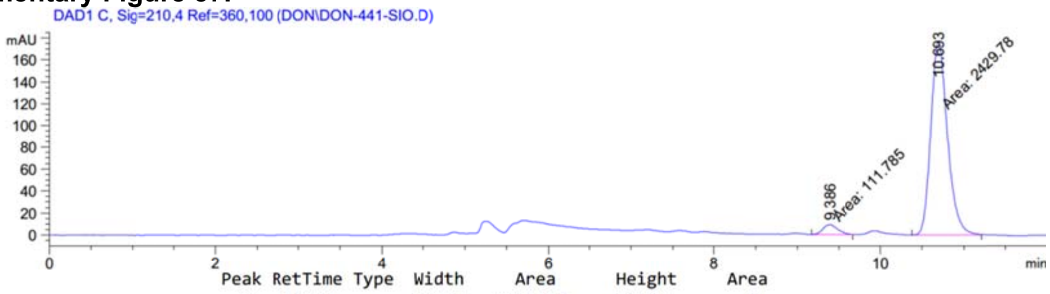

Peak #	RetTime [min]	Type	Width [min]	Area [mAU*s]	Height [mAU]	Area %
1	9.386	MM	0.2067	111.78467	9.01440	4.3983
2	10.693	MM	0.2293	2429.77563	176.61310	95.6017

(*R*)-1-(Naphthalen-2-yl)ethan-1-ol [(*R*)-1p]

The enantiomeric excess of (*R*)-**1p** was determined by HPLC analysis on a chiral stationary phase (Daicel Chiralcel AD-H column, column temperature 20°C, solvent *n*-heptane:isopropanol = 90:10, flow rate 0.8 mL/min, λ = 210 nm): t_R = 14.5 min for (*S*)-**1p**, t_R = 17.8 min for (*R*)-**1p.**

Supplementary Figure 58. *rac-1-(Naphthalen-2-yl)ethan-1-ol [rac-**1p**]*

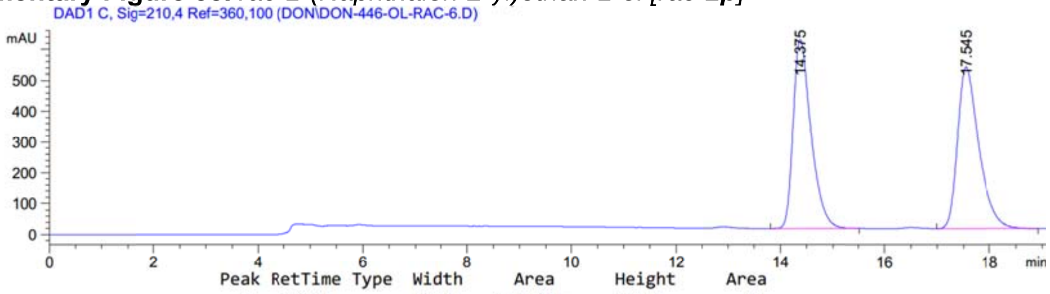

Peak #	RetTime [min]	Type	Width [min]	Area [mAU*s]	Height [mAU]	Area %
1	14.375	BB	0.3542	1.44858e4	611.82147	49.3145
2	17.545	VB	0.4278	1.48885e4	523.53735	50.6855

Supplementary Figure 59. *(R)-1-(Naphthalen-2-yl)ethan-1-ol [(R)-**1p**, 85.1% ee]*

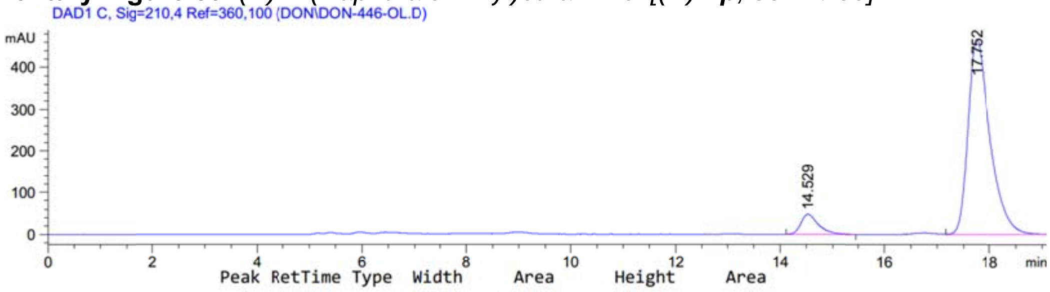

Peak #	RetTime [min]	Type	Width [min]	Area [mAU*s]	Height [mAU]	Area %
1	14.529	BB	0.3402	1102.92798	48.34426	7.4330
2	17.752	VB	0.4410	1.37354e4	467.25433	92.5670

(*S*)-Tributyl(1-(naphthalen-2-yl)ethoxy)silane [(*S*)-3ph, 74.0% ee]

The enantiomeric excess of (*S*)-**3ph** was determined by the deprotected alcohol, which was hydrolyzed and purified by preparative TLC according to GP5, using the same HPLC analysis condition with **1p**.

Supplementary Figure 60.

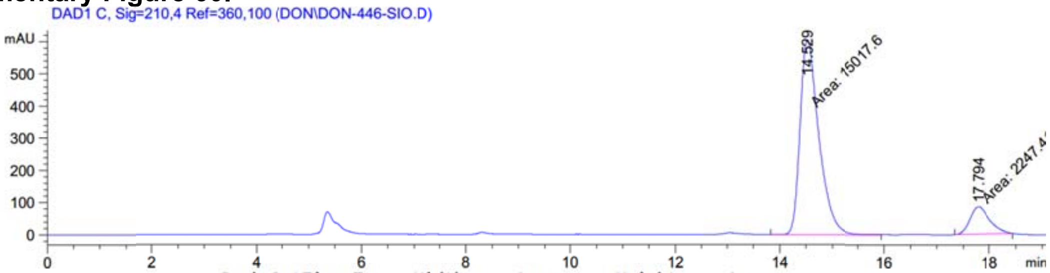

Peak #	RetTime [min]	Type	Width [min]	Area [mAU*s]	Height [mAU]	Area %
1	14.529	MM	0.4135	1.50176e4	605.37341	86.9829
2	17.794	MM	0.4370	2247.41431	85.70974	13.0171

(*R*)-1-(Naphthalen-1-yl)ethan-1-ol [(*R*)-1q]

The enantiomeric excess of (*R*)-**1q** was determined by HPLC analysis on a chiral stationary phase (Daicel Chiralcel OD-H column, column temperature 20°C, solvent *n*-heptane:isopropanol = 90:10, flow rate 0.8 mL/min, λ = 280 nm): t_R = 16.4 min for (*S*)-**1q**, t_R = 25.9 min for (*R*)-**1q**.

Supplementary Figure 61. *rac-1-(Naphthalen-1-yl)ethan-1-ol [rac-**1q**]*

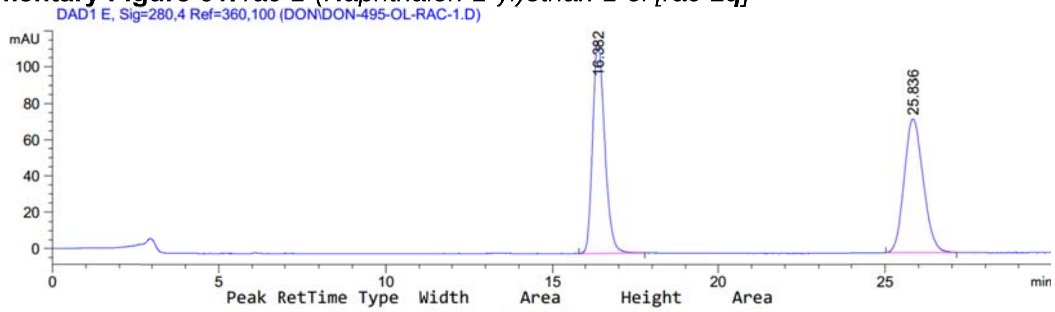

Peak #	RetTime [min]	Type	Width [min]	Area [mAU*s]	Height [mAU]	Area %
1	16.382	BB	0.3839	2918.16138	116.65205	50.1763
2	25.836	BB	0.5906	2897.65503	73.71857	49.8237

Supplementary Figure 62. *(R)-1-(Naphthalen-1-yl)ethan-1-ol [(R)-**1q**, 85.8% ee]*

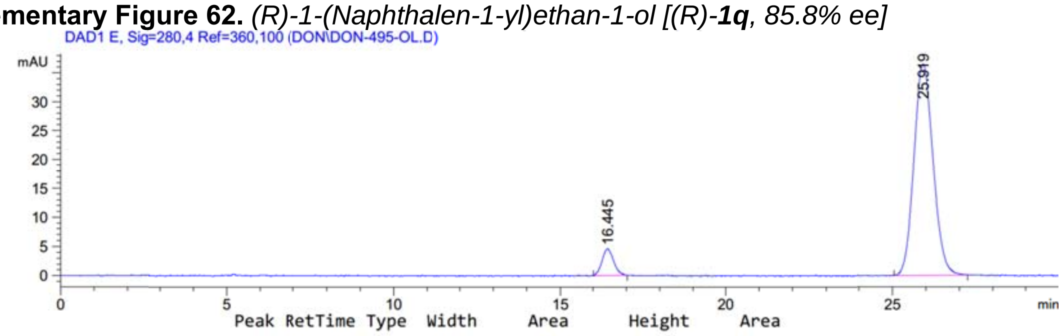

Peak #	RetTime [min]	Type	Width [min]	Area [mAU*s]	Height [mAU]	Area %
1	16.445	BB	0.3321	110.17017	4.58936	7.0988
2	25.919	BB	0.5852	1441.77380	36.46982	92.9012

(*S*)-Tributyl(1-(naphthalen-1-yl)ethoxy)silane [(*S*)-3qh, 70.4% ee]

The enantiomeric excess of (*S*)-**3qh** was determined by the deprotected alcohol, which was hydrolyzed and purified by preparative TLC according to GP5, using the same HPLC analysis condition with **1q**.

Supplementary Figure 63.

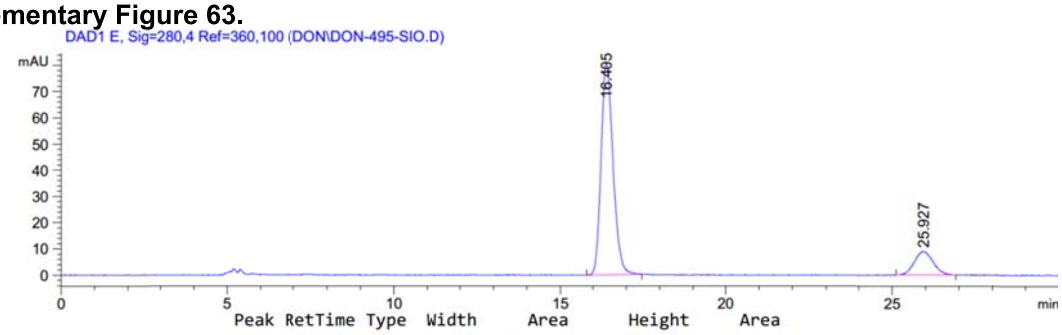

Peak #	RetTime [min]	Type	Width [min]	Area [mAU*s]	Height [mAU]	Area %
1	16.405	BB	0.3823	2005.84973	80.60561	85.1837
2	25.927	BB	0.4776	348.88574	8.97378	14.8163

(*R*)-2,3-Dihydro-1*H*-inden-1-ol [(*R*)-1r]

The enantiomeric excess of (*R*)-**1r** was determined by HPLC analysis on a chiral stationary phase (Daicel Chiralcel OD-H column, column temperature 20°C, solvent *n*-heptane:isopropanol = 96:4, flow rate 0.8 mL/min, λ = 210 nm): t_R = 28.6 min for (*S*)-**1r**, t_R = 31.8 min for (*R*)-**1r**.

Supplementary Figure 64. *rac-2,3-Dihydro-1H-inden-1-ol [rac-**1r**]*

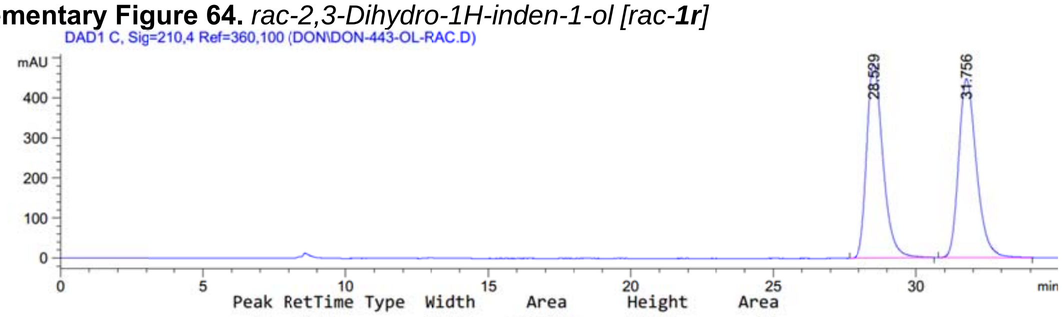

Peak #	RetTime [min]	Type	Width [min]	Area [mAU*s]	Height [mAU]	Area %
1	28.529	BB	0.5951	1.90941e4	485.22372	49.8893
2	31.756	BB	0.6360	1.91789e4	445.74051	50.1107

Supplementary Figure 65. *(R)-2,3-Dihydro-1H-inden-1-ol [(R)-**1r**, 88.0% ee]*

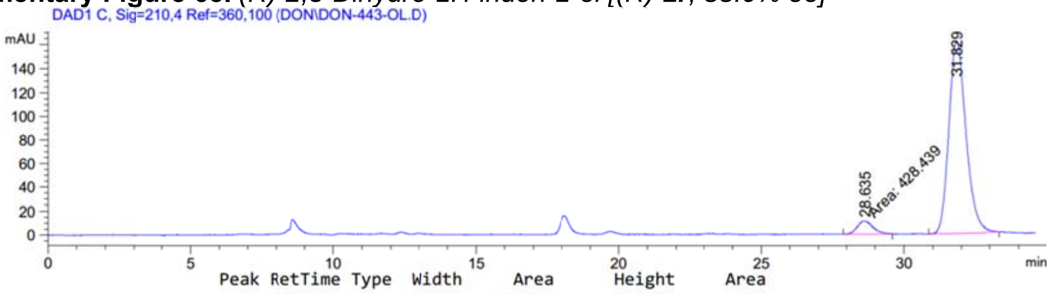

Peak #	RetTime [min]	Type	Width [min]	Area [mAU*s]	Height [mAU]	Area %
1	28.635	MM	0.6383	428.43915	11.18781	5.9915
2	31.829	BB	0.6197	6722.31885	162.15540	94.0085

(*S*)-Tributyl((2,3-dihydro-1*H*-inden-1-yl)oxy)silane [(*S*)-3rh, 76.4% ee]

The enantiomeric excess of (*S*)-**3rh** was determined after deprotection to the alcohol [according to GP5 without purification by preparative TLC] using the HPLC setup for **1r**.

Supplementary Figure 66.

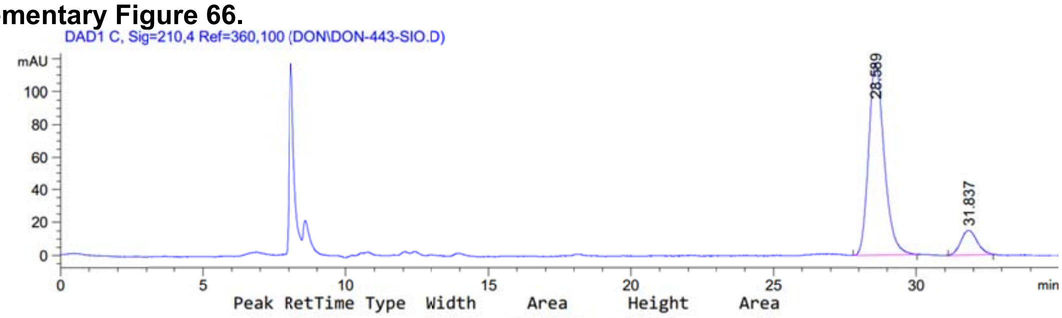

Peak #	RetTime [min]	Type	Width [min]	Area [mAU*s]	Height [mAU]	Area %
1	28.589	BB	0.5480	4432.82666	117.53780	88.1728
2	31.837	BB	0.4729	594.60162	15.11650	11.8272

(*R*)-1,2,3,4-Tetrahydronaphthalen-1-ol [(*R*)-1s]

The enantiomeric excess of (*R*)-**1s** was determined by HPLC analysis on a chiral stationary phase (Daicel Chiralcel OD-H column, column temperature 20°C, solvent *n*-heptane:isopropanol = 98:2, flow rate 0.8 mL/min, λ = 210 nm): t_R = 19.0 min for (*S*)-**1s**, t_R = 20.6 min for (*R*)-**1s.**

Supplementary Figure 67. *rac-1,2,3,4-Tetrahydronaphthalen-1-ol [rac-**1s**]*

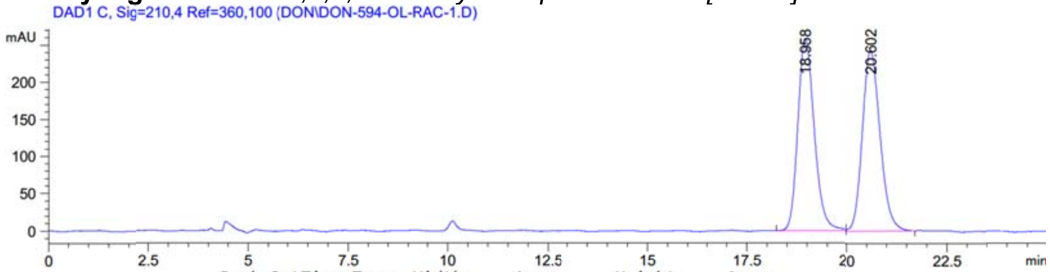

Peak #	RetTime [min]	Type	Width [min]	Area [mAU*s]	Height [mAU]	Area %
1	18.958	BV	0.4376	7415.65088	259.35776	49.9917
2	20.602	VB	0.4629	7418.11865	242.51489	50.0083

Supplementary Figure 68. *(R)-1,2,3,4-Tetrahydronaphthalen-1-ol [(R)-**1s**, 94.8% ee]*

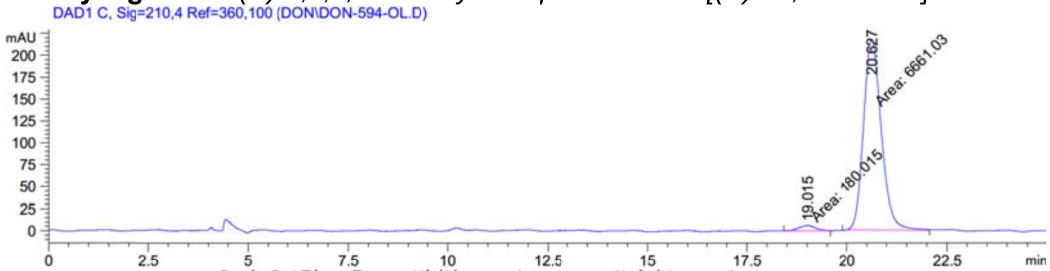

Peak #	RetTime [min]	Type	Width [min]	Area [mAU*s]	Height [mAU]	Area %
1	19.015	MM	0.4883	180.01541	6.14452	2.6314
2	20.627	MM	0.5104	6661.03125	217.51074	97.3686

(*S*)-Tributyl((1,2,3,4-tetrahydronaphthalen-1-yl)oxy)silane [(*S*)-3sh, 83.4% ee]

The enantiomeric excess of (*S*)-**3sh** was determined by the deprotected alcohol, which was hydrolyzed and purified by preparative TLC according to GP5, using the same HPLC analysis condition with **1s**.

Supplementary Figure 69.

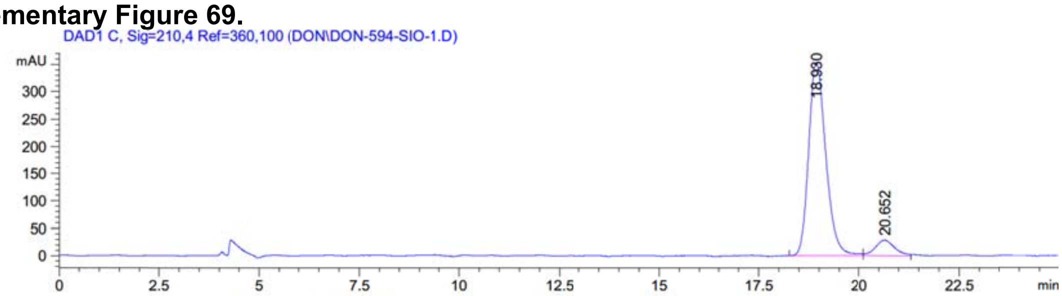

Peak #	RetTime [min]	Type	Width [min]	Area [mAU*s]	Height [mAU]	Area %
1	18.930	BV	0.4450	1.05271e4	353.95081	91.7355
2	20.652	VV	0.4033	948.39606	28.56910	8.2645

(*R*)-6,7,8,9-Tetrahydro-5H-benzo[7]annulen-5-ol [(*R*)-1t]
The enantiomeric excess of (*R*)-**1t** was determined by HPLC analysis on a chiral stationary phase (Daicel Chiralcel OD-H column, column temperature 20°C, solvent *n*-heptane:isopropanol = 98:2, flow rate 0.8 mL/min, λ = 210 nm): t_R = 27.3 min for (*R*)-**1t**, t_R = 29.9 min for (*S*)-**1t**.
Supplementary Figure 70. *rac-6,7,8,9-Tetrahydro-5H-benzo[7]annulen-5-ol [rac-**1t**]*

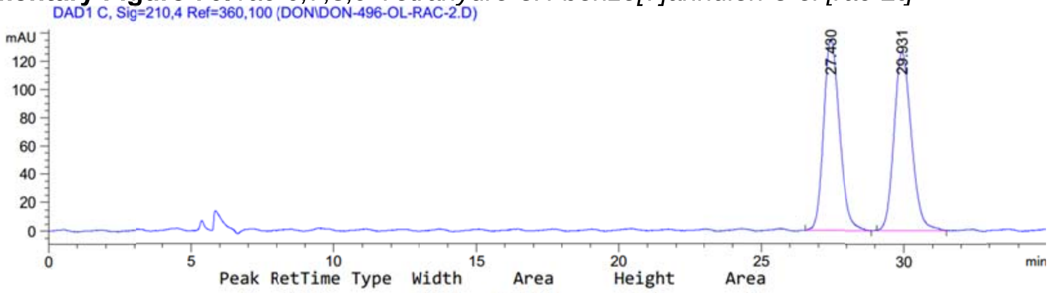

Peak #	RetTime [min]	Type	Width [min]	Area [mAU*s]	Height [mAU]	Area %
1	27.430	BB	0.5726	5287.43848	135.68053	49.9013
2	29.931	VV	0.6022	5308.34473	126.34800	50.0987

Supplementary Figure 71. *(R)-6,7,8,9-Tetrahydro-5H-benzo[7]annulen-5-ol [(R)-**1t**, 87.4% ee]*

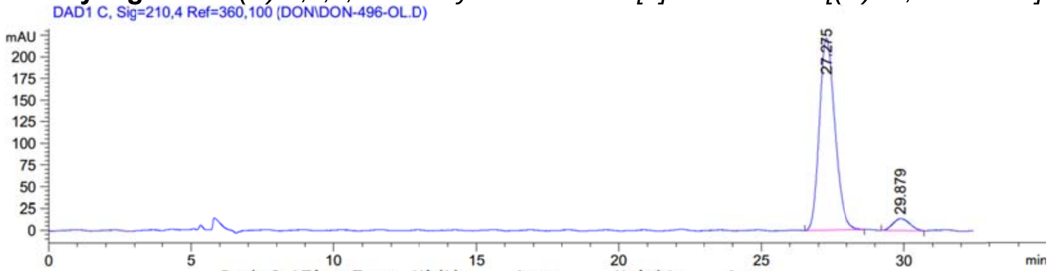

Peak #	RetTime [min]	Type	Width [min]	Area [mAU*s]	Height [mAU]	Area %
1	27.275	BB	0.5948	8413.78320	221.69667	93.7095
2	29.879	BB	0.4868	564.79346	13.93663	6.2905

(*S*)-Tributyl((6,7,8,9-tetrahydro-5H-benzo[7]annulen-5-yl)oxy)silane [(*S*)-3th, 64.8% ee]
The enantiomeric excess of (*S*)-**3th** was determined after deprotection to the alcohol [according to GP5 without purification by preparative TLC] using the HPLC setup for **1t**.
Supplementary Figure 72.

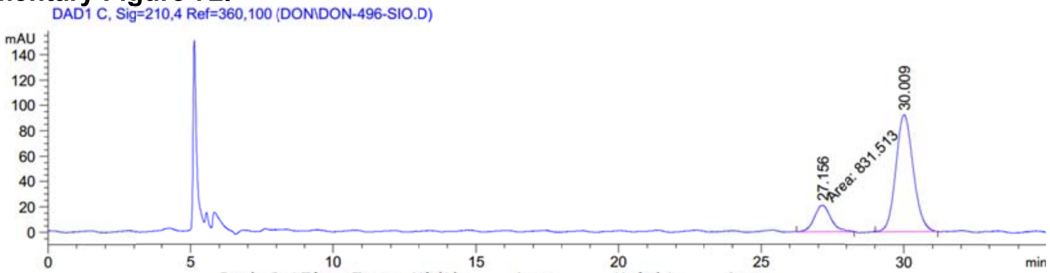

Peak #	RetTime [min]	Type	Width [min]	Area [mAU*s]	Height [mAU]	Area %
1	27.156	MM	0.6684	831.51312	20.73374	17.6153
2	30.009	VB	0.6107	3888.88232	92.45730	82.3847

(*R*)-Chroman-4-ol [(*R*)-1u]

The enantiomeric excess of (*R*)-**1u** was determined by HPLC analysis on a chiral stationary phase (Daicel Chiralcel OD-H column, column temperature 20°C, solvent *n*-heptane:isopropanol = 95:5, flow rate 0.8 mL/min, λ = 280 nm): t_R = 16.8 min for (*S*)-**1u**, t_R = 19.3 min for (*R*)-**1u**.

Supplementary Figure 73. *rac-Chroman-4-ol [rac-**1u**]*

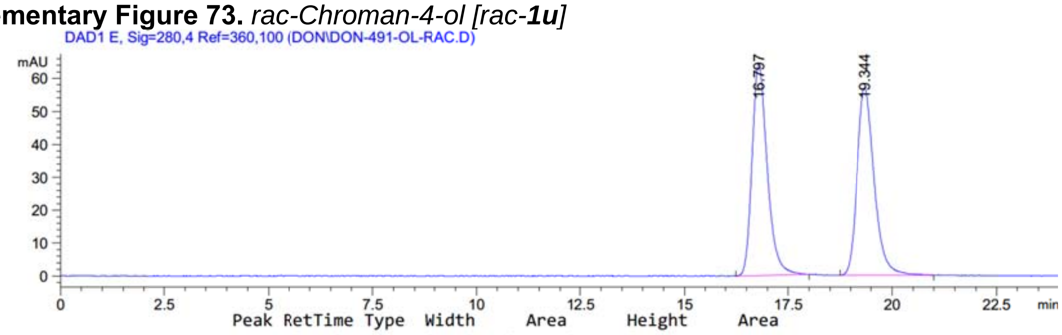

Peak #	RetTime [min]	Type	Width [min]	Area [mAU*s]	Height [mAU]	Area %
1	16.797	BB	0.3844	1630.31360	64.16837	49.7636
2	19.344	BB	0.4374	1645.80359	56.90595	50.2364

Supplementary Figure 74. *(R)-Chroman-4-ol [(R)-**1u**, >99.9% ee]*

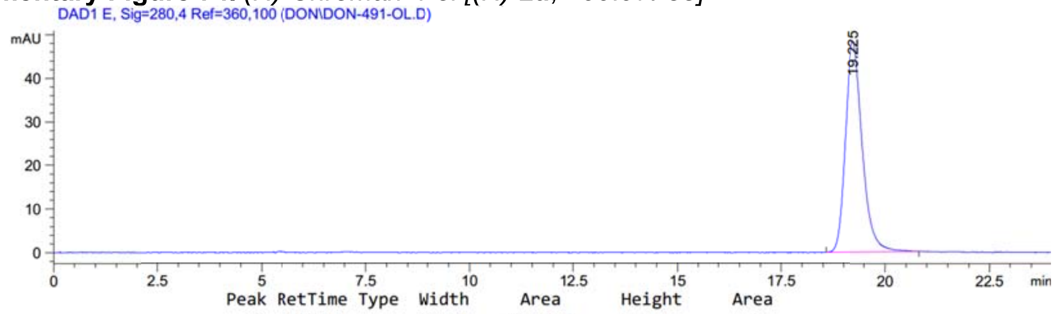

Peak #	RetTime [min]	Type	Width [min]	Area [mAU*s]	Height [mAU]	Area %
1	19.225	BB	0.4269	1389.26025	48.39035	100.0000

(*S*)-Tributyl(chroman-4-yloxy)silane [(*S*)-3uh, 67.0% ee]

The enantiomeric excess of (*S*)-**3uh** was determined by the deprotected alcohol, which was hydrolyzed and purified by preparative TLC according to GP5, using the same HPLC analysis condition with **1u**.

Supplementary Figure 75.

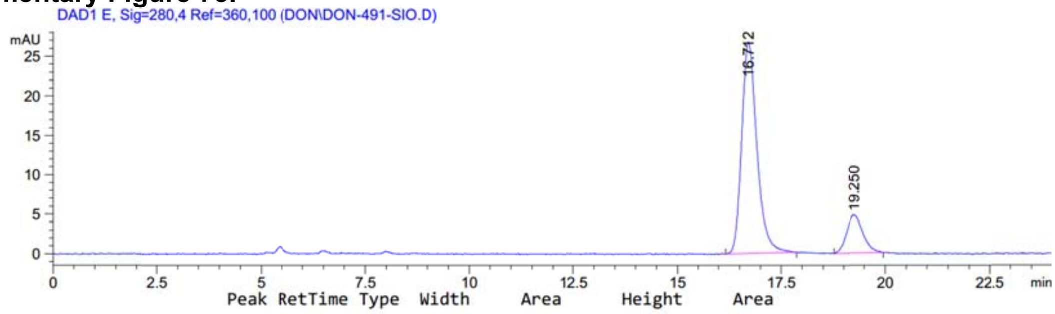

Peak #	RetTime [min]	Type	Width [min]	Area [mAU*s]	Height [mAU]	Area %
1	16.712	BB	0.3869	676.10400	26.74893	83.4931
2	19.250	BB	0.3548	133.66800	4.83036	16.5069

(*R*)-Thiochroman-4-ol [(*R*)-1v]

The enantiomeric excess of (*R*)-**1v** was determined by HPLC analysis on a chiral stationary phase (Daicel Chiralcel OD-H column, column temperature 20°C, solvent *n*-heptane:isopropanol = 95:5, flow rate 0.8 mL/min, λ = 210 nm): t_R = 20.6 min for (*S*)-**1v**, t_R = 25.4 min for (*R*)-**1v.**

Supplementary Figure 76. *rac-Thiochroman-4-ol [rac-**1v**]*

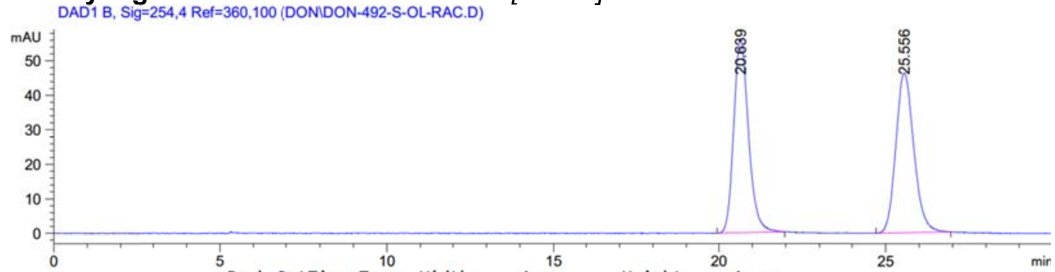

Peak #	RetTime [min]	Type	Width [min]	Area [mAU*s]	Height [mAU]	Area %
1	20.639	BB	0.4778	1725.83557	56.27243	49.9418
2	25.556	BB	0.5610	1729.85974	46.38994	50.0582

Supplementary Figure 77. *(R)-Thiochroman-4-ol [(R)-**1v**, 99.7% ee]*

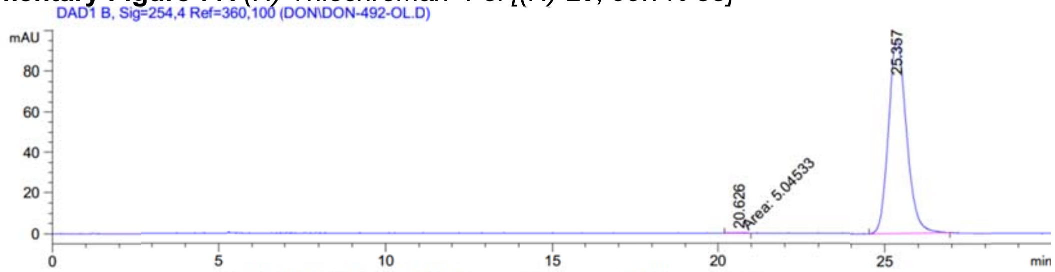

Peak #	RetTime [min]	Type	Width [min]	Area [mAU*s]	Height [mAU]	Area %
1	20.626	MM	0.3996	5.04533	2.10456e-1	0.1421
2	25.357	BB	0.5608	3544.74536	95.54396	99.8579

(*S*)-Tributyl(thiochroman-4-yloxy)silane [(*S*)-3vh, 67.0% ee]

The enantiomeric excess of (*S*)-**3vh** was determined by the deprotected alcohol, which was hydrolyzed and purified by preparative TLC according to GP5, using the same HPLC analysis condition with **1v**.

Supplementary Figure 78.

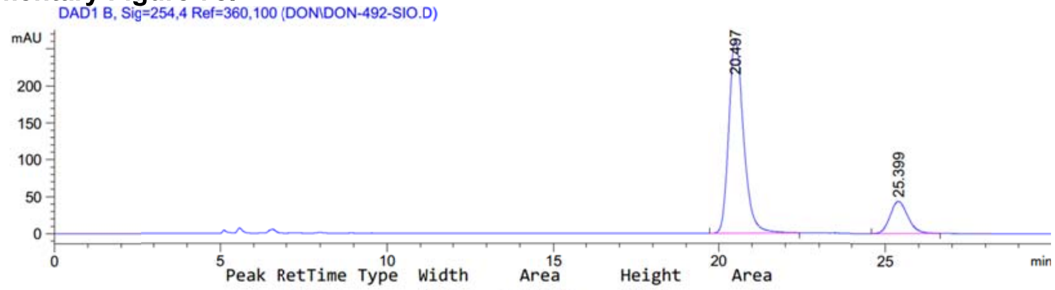

Peak #	RetTime [min]	Type	Width [min]	Area [mAU*s]	Height [mAU]	Area %
1	20.497	BB	0.4694	8039.02783	262.43539	83.4617
2	25.399	BB	0.5573	1592.96851	43.29497	16.5383

(*R*)-*tert*-Butyl 4-hydroxy-3,4-dihydroquinoline-1(2*H*)-carboxylate [(*R*)-1w]

The enantiomeric excess of (*R*)-**1w** was determined by HPLC analysis on a chiral stationary phase (Daicel Chiralcel OD-H column, column temperature 20°C, solvent *n*-heptane:isopropanol = 97:3, flow rate 0.8 mL/min, λ = 250 nm): t_R = 25.1 min for (*S*)-**1w**, t_R = 27.4 min for (*R*)-**1w**.

Supplementary Figure 79. *rac-tert-Butyl 4-hydroxy-3,4-dihydroquinoline-1(2H)-carboxylate [rac-**1w**]*

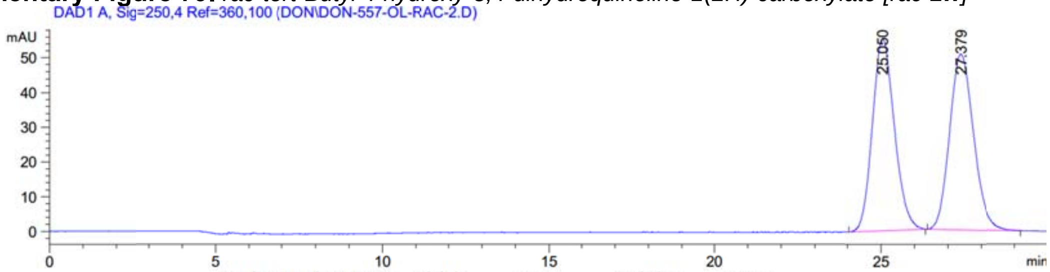

Peak #	RetTime [min]	Type	Width [min]	Area [mAU*s]	Height [mAU]	Area %
1	25.050	BB	0.6754	2517.68652	55.27530	49.9514
2	27.379	BB	0.7273	2522.58765	50.62732	50.0486

Supplementary Figure 80. *(R)-tert-Butyl 4-hydroxy-3,4-dihydroquinoline-1(2H)-carboxylate [(R)-**1w**, 86.2% ee]*

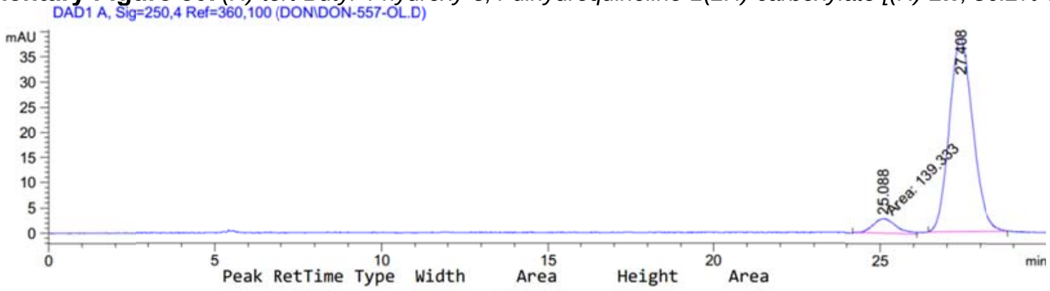

Peak #	RetTime [min]	Type	Width [min]	Area [mAU*s]	Height [mAU]	Area %
1	25.088	MM	0.7953	139.33344	2.91975	6.9172
2	27.408	BB	0.7163	1874.97998	38.22384	93.0828

(*S*)-*tert*-Butyl 4-((tributylsilyl)oxy)-3,4-dihydroquinoline-1(2*H*)-carboxylate [(*S*)-3wh, 86.2% ee]

The enantiomeric excess of (*S*)-**3wh** was determined by the deprotected alcohol, which was hydrolyzed and purified by preparative TLC according to GP5, using the same HPLC analysis condition with **1w**.

Supplementary Figure 81.

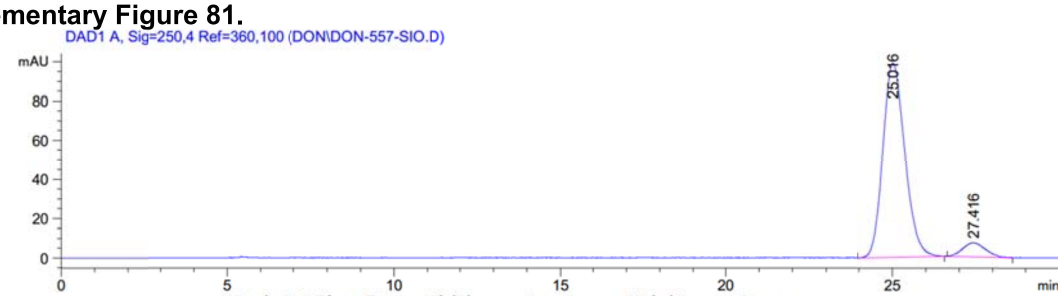

Peak #	RetTime [min]	Type	Width [min]	Area [mAU*s]	Height [mAU]	Area %
1	25.016	BB	0.6908	4497.90674	98.86665	93.0876
2	27.416	BB	0.5745	334.00156	7.13029	6.9124

(1*R*,2*S*)-2-Phenylcyclohexan-1-ol [(1*R*,2*S*)-4]

The enantiomeric excess of (1*R*,2*S*)-**4** was determined by HPLC analysis on a chiral stationary phase (Daicel Chiralcel OD-H column, column temperature 20°C, solvent *n*-heptane:isopropanol = 98:2, flow rate 0.8 mL/min, λ = 210 nm): t_R = 16.7 min for (1*S*,2*R*)-**4**, t_R = 18.8 min for (1*R*,2*S*)-**4**.

Supplementary Figure 82. *rac-(1R,2S)-2-Phenylcyclohexan-1-ol [rac-trans-**4**]*

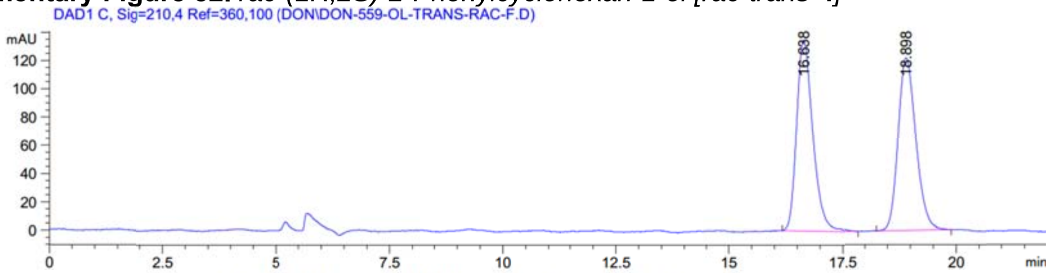

Peak #	RetTime [min]	Type	Width [min]	Area [mAU*s]	Height [mAU]	Area %
1	16.638	BB	0.3832	3331.89893	134.42079	50.2288
2	18.898	BB	0.4131	3301.54346	122.23403	49.7712

Supplementary Figure 83. *(1R,2S)-2-Phenylcyclohexan-1-ol* [(1*R*,2*S*)-**4**, *81.0% ee]*

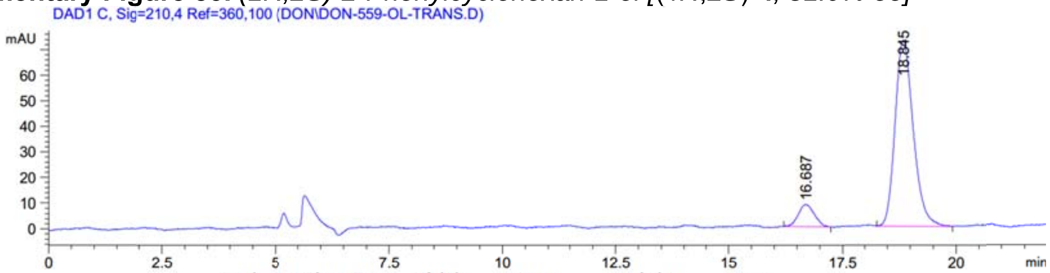

Peak #	RetTime [min]	Type	Width [min]	Area [mAU*s]	Height [mAU]	Area %
1	16.687	BB	0.3009	211.25610	8.65002	9.5261
2	18.845	BB	0.4258	2006.38879	72.74754	90.4739

Tributyl(((1S,2R)-2-phenylcyclohexyl)oxy)silane [(1*S*,2*R*)-5h, 84.4% ee]

The enantiomeric excess of (1*S*,2*R*)-**5h** was determined by the deprotected alcohol, which was hydrolyzed and purified by preparative TLC according GP5, using the same HPLC analysis condition with *trans*-**4**.

Supplementary Figure 84.

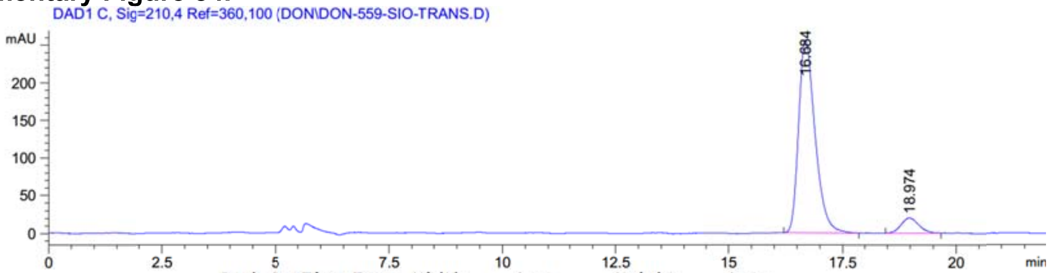

Peak #	RetTime [min]	Type	Width [min]	Area [mAU*s]	Height [mAU]	Area %
1	16.684	BV	0.3840	6416.41846	256.36560	92.1830
2	18.974	VB	0.3286	544.10522	20.30856	7.8170

(1*R*,2*R*)-2-Phenylcyclohexan-1-ol [(1*R*,2*R*)-4]

The enantiomeric excess of (1*R*,2*R*)-**4** was determined by HPLC analysis on a chiral stationary phase (Daicel Chiralcel OD-H column, column temperature 20°C, solvent *n*-heptane:isopropanol = 98:2, flow rate 0.8 mL/min, λ = 210 nm): t_R = 14.3 min for (1*S*,2*S*)-**4**, t_R = 18.3 min for (1*R*,2*R*)-**4.**

Supplementary Figure 85. *rac-cis-2-Phenylcyclohexan-1-ol [rac-cis-**4**]*

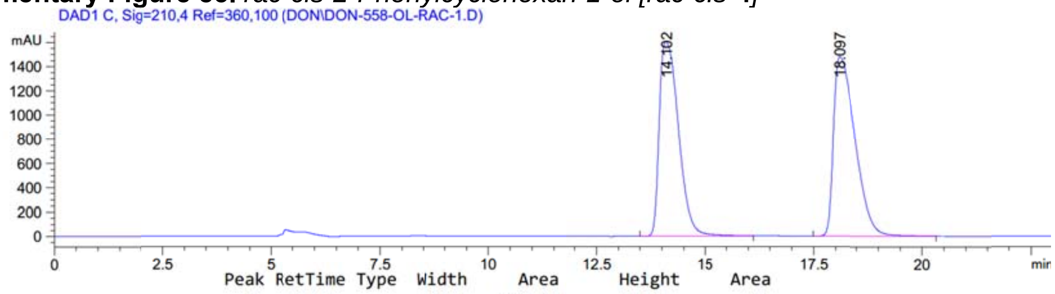

Peak #	RetTime [min]	Type	Width [min]	Area [mAU*s]	Height [mAU]	Area %
1	14.102	BV	0.4225	4.94095e4	1607.89172	48.7381
2	18.097	BB	0.4597	5.19680e4	1479.76929	51.2619

Supplementary Figure 86. *(1R,2R)-2-Phenylcyclohexan-1-ol [(1R,2R)-**4**, 45.2% ee]*

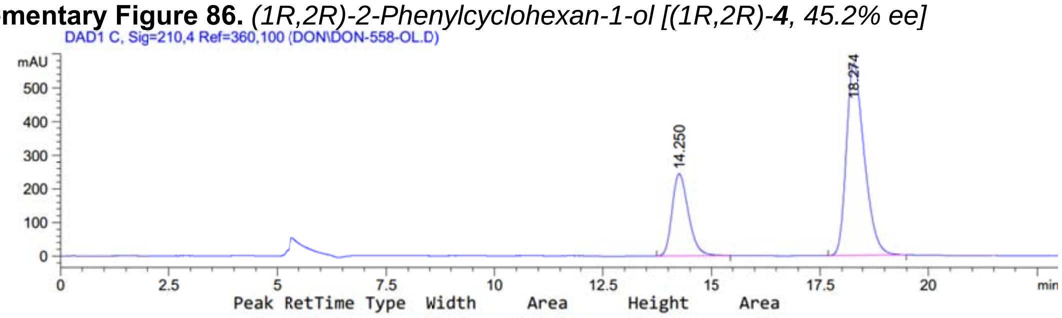

Peak #	RetTime [min]	Type	Width [min]	Area [mAU*s]	Height [mAU]	Area %
1	14.250	BB	0.4033	6339.59619	243.83829	27.3523
2	18.274	BB	0.4465	1.68380e4	570.22473	72.6477

Tributyl(((1S,2S)-2-phenylcyclohexyl)oxy)silane [(1*S*,2*S*)-5h, 69.6% ee]

The enantiomeric excess of (1*S*,2*S*)-**5h** was determined by the deprotected alcohol, which was hydrolyzed and purified by preparative TLC according to GP5, using the same HPLC analysis condition with *cis*-**4**.

Supplementary Figure 87.

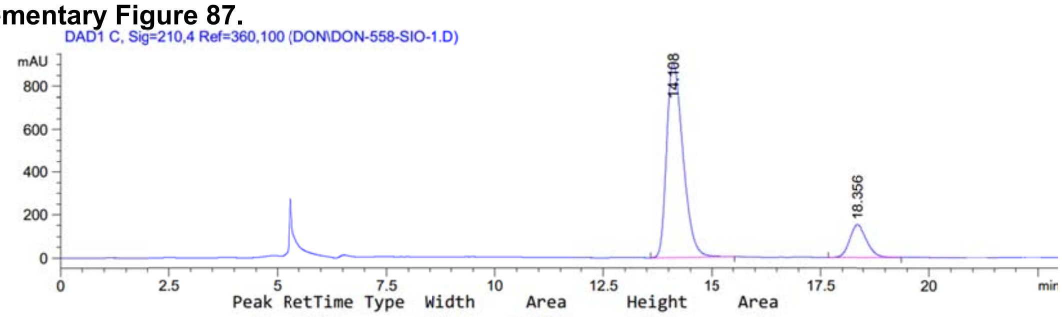

Peak #	RetTime [min]	Type	Width [min]	Area [mAU*s]	Height [mAU]	Area %
1	14.108	BB	0.4065	2.37049e4	908.09143	84.8371
2	18.356	BB	0.4223	4236.74902	152.40012	15.1629

(*R*,*E*)-3-Methyl-4-phenylbut-3-en-2-ol [(*R*)-6a]

The enantiomeric excess of (*R*)-**6a** was determined by HPLC analysis on a chiral stationary phase (Daicel Chiralcel OD-H column, column temperature 20°C, solvent *n*-heptane:isopropanol = 95:5, flow rate 0.8 mL/min, λ = 254 nm): t_R = 11.3 min for (*R*)-**6a**, t_R = 12.9 min for (*S*)-**6a**.

Supplementary Figure 88. *rac-(E)-3-Methyl-4-phenylbut-3-en-2-ol [rac-***6a***]*

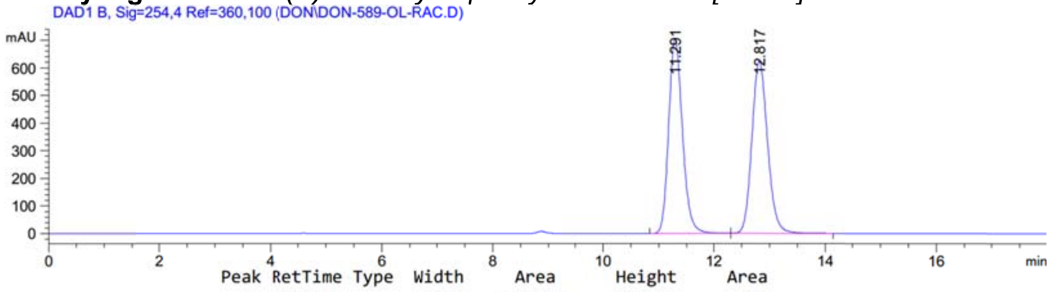

Peak #	RetTime [min]	Type	Width [min]	Area [mAU*s]	Height [mAU]	Area %
1	11.291	BB	0.2669	1.21360e4	704.62848	49.9083
2	12.817	BB	0.3022	1.21805e4	622.82336	50.0917

Supplementary Figure 89. *(R,E)-3-Methyl-4-phenylbut-3-en-2-ol [(R)-***6a***, 87.4% ee]*

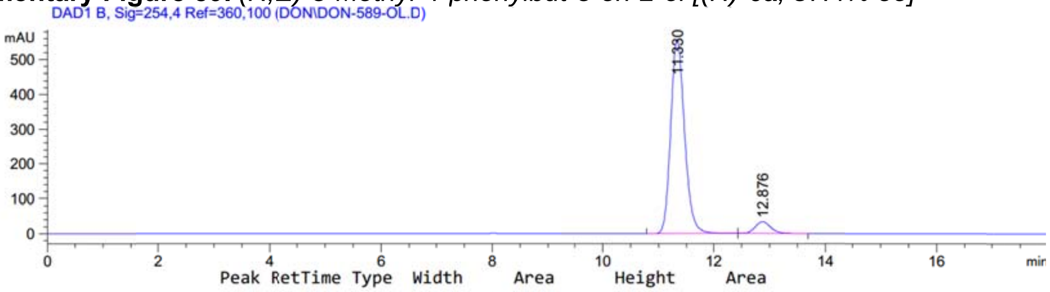

Peak #	RetTime [min]	Type	Width [min]	Area [mAU*s]	Height [mAU]	Area %
1	11.330	BB	0.2674	9650.07422	559.12451	93.7003
2	12.876	BB	0.2980	648.79559	33.79542	6.2997

(*S*,*E*)-Tributyl((3-methyl-4-phenylbut-3-en-2-yl)oxy)silane [(*S*)-7ah, 74.8% ee]

The enantiomeric excess of (*S*)-**7ah** was determined by the deprotected alcohol, which was hydrolyzed and purified by preparative TLC according to GP5, using the same HPLC analysis condition with **6a**.

Supplementary Figure 90.

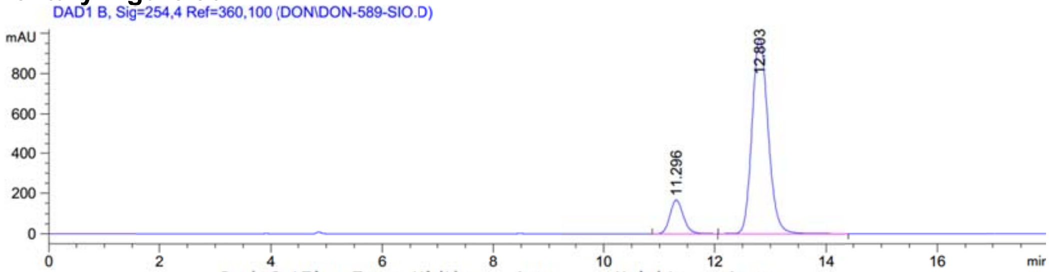

Peak #	RetTime [min]	Type	Width [min]	Area [mAU*s]	Height [mAU]	Area %
1	11.296	BB	0.2599	2829.20435	168.45386	12.6557
2	12.803	BB	0.3163	1.95260e4	972.22821	87.3443

(*R*,*E*)-3-Methyl-4-phenylbut-3-en-2-ol [(*R*)-6a, 82.6% *ee*]

The enantiomeric excess of (*R*)-**6a** was determined by HPLC analysis on a chiral stationary phase (Daicel Chiralcel OD-H column, column temperature 20°C, solvent *n*-heptane:isopropanol = 95:5, flow rate 0.8 mL/min, λ = 254 nm): t_R = 14.8 min for (*R*)-**6a**, t_R = 16.9 min for (*S*)-**6a**.

Supplementary Figure 91.

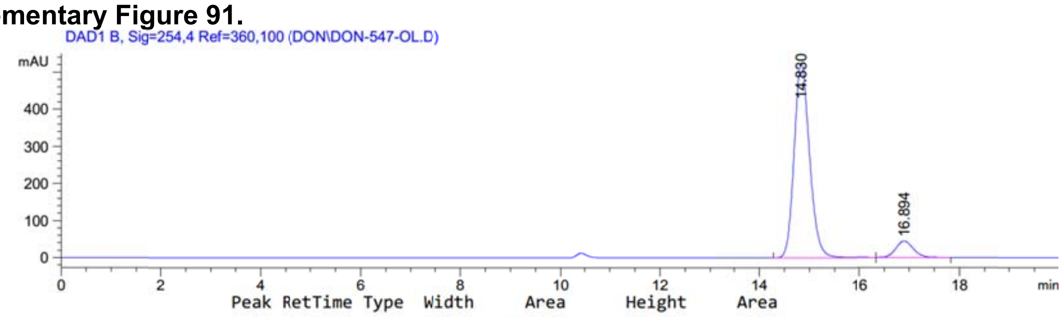

Peak #	RetTime [min]	Type	Width [min]	Area [mAU*s]	Height [mAU]	Area %
1	14.830	BB	0.3293	1.11946e4	523.91504	91.3490
2	16.894	BB	0.3746	1060.16431	44.07546	8.6510

(*S*,*E*)-Tributyl((3-methyl-4-phenylbut-3-en-2-yl)oxy)silane [(*S*)-7ah, 79.2% ee]

The enantiomeric excess of (*S*)-**7ah** was determined by the deprotected alcohol, which was hydrolyzed and purified by preparative TLC according to GP5, using the same HPLC analysis condition with **6a**.

Supplementary Figure 92.

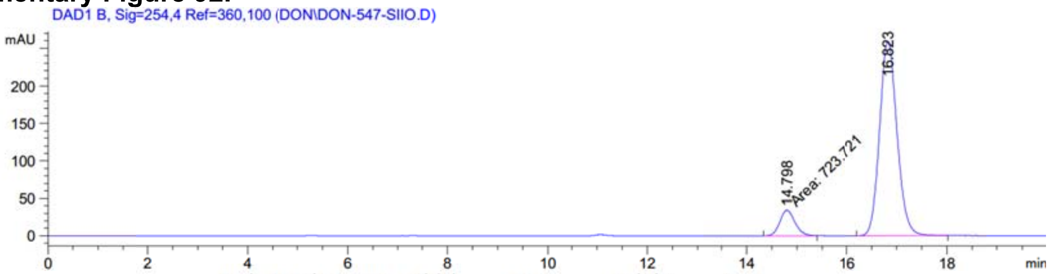

Peak #	RetTime [min]	Type	Width [min]	Area [mAU*s]	Height [mAU]	Area %
1	14.798	MM	0.3509	723.72089	34.37938	10.4023
2	16.823	BB	0.3742	6233.61621	259.51495	89.5977

(*R*,*E*)-3-(4-Methoxyphenyl)-4-phenylbut-3-en-2-ol [(*R*)-6b]

The enantiomeric excess of (*R*)-**6b** was determined by HPLC analysis on a chiral stationary phase (Daicel Chiralcel OD-H column, column temperature 20°C, solvent *n*-heptane:isopropanol = 95:5, flow rate 0.8 mL/min, λ = 254 nm): t_R = 22.4 min for (*R*)-**6b**, t_R = 27.1 min for (*S*)-**6b**.

Supplementary Figure 93. *rac-(E)-3-(4-Methoxyphenyl)-4-phenylbut-3-en-2-ol [rac-***6b***]*

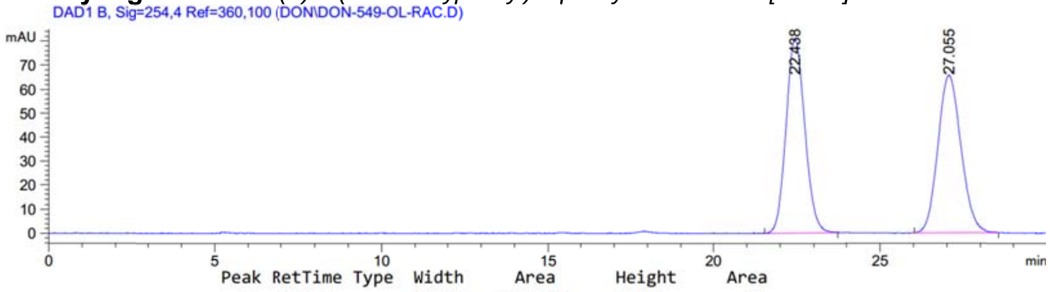

Peak #	RetTime [min]	Type	Width [min]	Area [mAU*s]	Height [mAU]	Area %
1	22.438	BB	0.5991	3155.96826	80.90125	50.1041
2	27.055	BB	0.7232	3142.85229	65.80540	49.8959

Supplementary Figure 94. *(R,E)-3-(4-Methoxyphenyl)-4-phenylbut-3-en-2-ol [(R)-***6b***, 91.6% ee]*

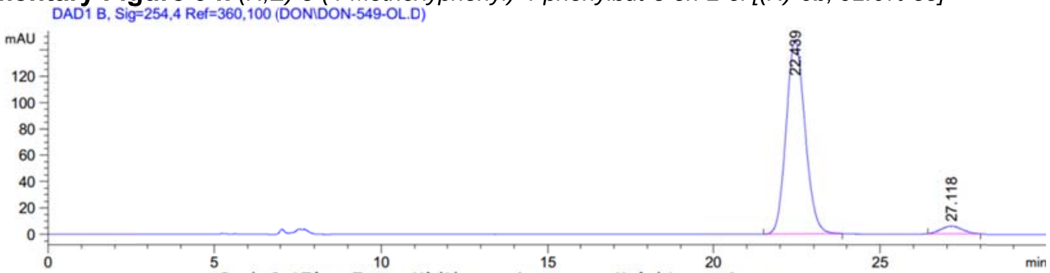

Peak #	RetTime [min]	Type	Width [min]	Area [mAU*s]	Height [mAU]	Area %
1	22.439	BB	0.6075	5765.19189	147.02908	95.8271
2	27.118	BB	0.5280	251.05151	5.69882	4.1729

(*S*,*E*)-Tributyl((3-(4-methoxyphenyl)-4-phenylbut-3-en-2-yl)oxy)silane [(*S*)-7bh, 80.6% ee]

The enantiomeric excess of (*S*)-**7bh** was determined by the deprotected alcohol, which was hydrolyzed and purified by preparative TLC according to GP5, using the same HPLC analysis condition with **6b**.

Supplementary Figure 95.

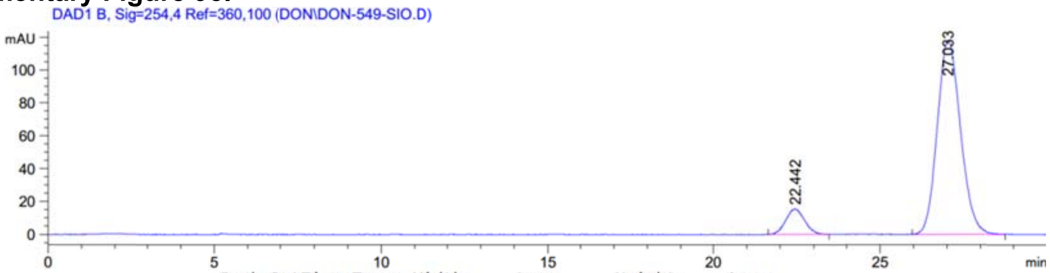

Peak #	RetTime [min]	Type	Width [min]	Area [mAU*s]	Height [mAU]	Area %
1	22.442	BB	0.4793	608.71918	15.46322	9.6915
2	27.033	BB	0.7211	5672.20801	117.93541	90.3085

(*R*,*Z*)-3-Bromo-4-phenylbut-3-en-2-ol [(*R*)-6c]

The enantiomeric excess of (*R*)-**6c** was determined by HPLC analysis on a chiral stationary phase (Daicel Chiralcel OD-H column, column temperature 20°C, solvent *n*-heptane:isopropanol = 98:2, flow rate 0.8 mL/min, λ = 254 nm): t_R = 38.0 min for (*R*)-**6c**, t_R = 42.7 min for (*S*)-**6c**.

Supplementary Figure 96. *rac-(Z)-3-Bromo-4-phenylbut-3-en-2-ol [rac-**6c**]*

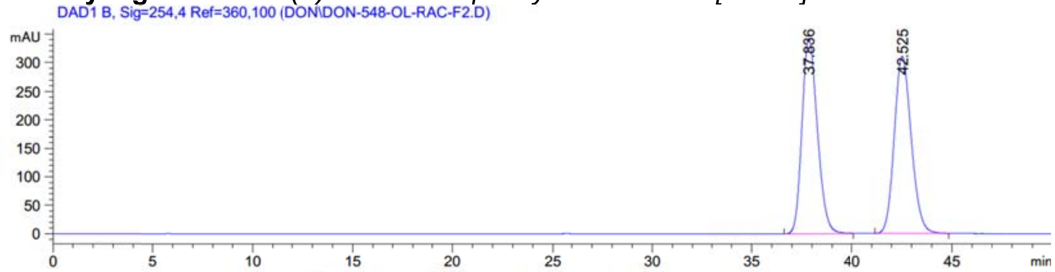

Peak #	RetTime [min]	Type	Width [min]	Area [mAU*s]	Height [mAU]	Area %
1	37.836	BB	0.8168	1.82397e4	341.69983	50.0322
2	42.525	BB	0.8942	1.82162e4	309.71793	49.9678

Supplementary Figure 97. *(R,Z)-3-Bromo-4-phenylbut-3-en-2-ol [(R)-**6c**, 78.0% ee]*

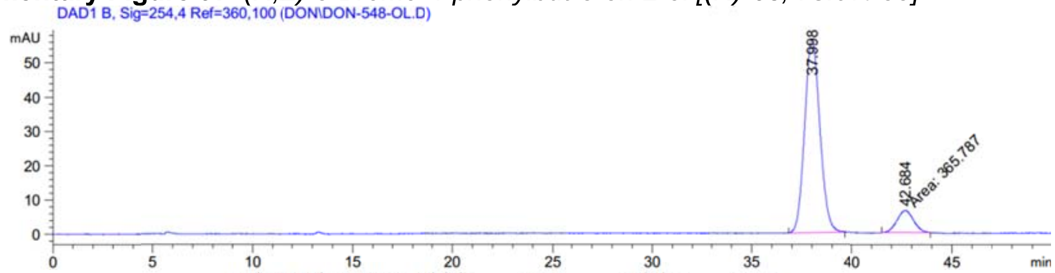

Peak #	RetTime [min]	Type	Width [min]	Area [mAU*s]	Height [mAU]	Area %
1	37.998	BB	0.7909	2972.55835	56.40126	89.0429
2	42.684	MM	0.9495	365.78714	6.42048	10.9571

(*S*,*Z*)-((3-Bromo-4-phenylbut-3-en-2-yl)oxy)tributylsilane [(*S*)-7ch, 88.4% ee]

The enantiomeric excess of (*S*)-**7ch** was determined by the deprotected alcohol, which was hydrolyzed and purified by preparative TLC according to GP5, using the same HPLC analysis condition with **6c**.

Supplementary Figure 98.

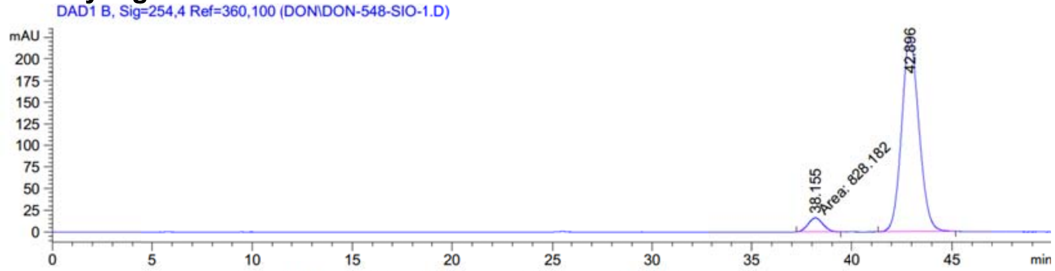

Peak #	RetTime [min]	Type	Width [min]	Area [mAU*s]	Height [mAU]	Area %
1	38.155	MM	0.8536	828.18152	16.17029	5.8059
2	42.896	BB	0.8896	1.34364e4	224.70935	94.1941

(*R*,*Z*)-3-Bromo-4-phenylbut-3-en-2-ol [(*R*)-6c]

The enantiomeric excess of (*R*)-**6c** was determined by HPLC analysis on a chiral stationary phase (Daicel Chiralcel OD-H column, column temperature 20°C, solvent *n*-heptane:isopropanol = 98:2, flow rate 0.8 mL/min, λ = 254 nm): t_R = 29.2 min for (*R*)-**6c**, t_R = 32.7 min for (*S*)-**6c**.

Supplementary Figure 99. *rac-(Z)-3-Bromo-4-phenylbut-3-en-2-ol [rac-***6c***]*

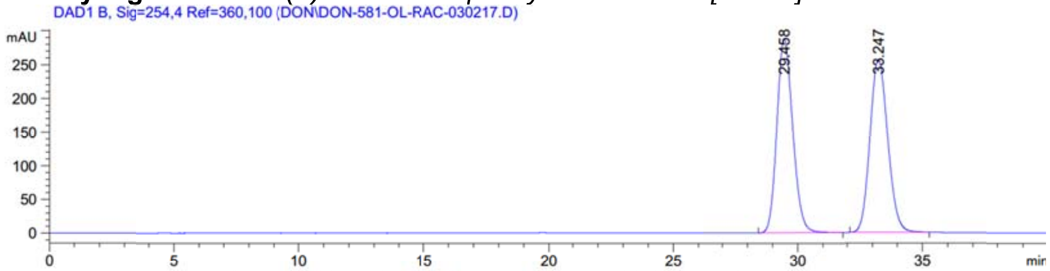

Peak #	RetTime [min]	Type	Width [min]	Area [mAU*s]	Height [mAU]	Area %
1	29.458	BB	0.6821	1.26651e4	287.39868	50.1303
2	33.247	BB	0.7577	1.25992e4	256.21005	49.8697

Supplementary Figure 100. *(R,Z)-3-Bromo-4-phenylbut-3-en-2-ol [(R)-***6c***, 86.4% ee]*

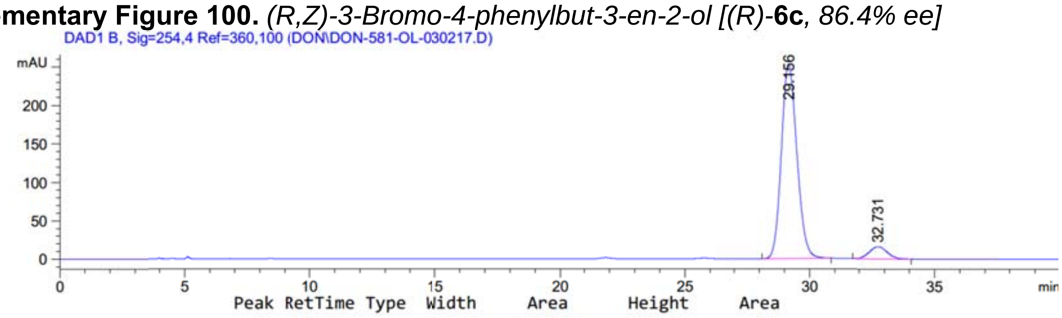

Peak #	RetTime [min]	Type	Width [min]	Area [mAU*s]	Height [mAU]	Area %
1	29.156	BB	0.6616	1.08889e4	253.28510	93.1834
2	32.731	BB	0.6130	796.55682	15.88463	6.8166

(*S*,*Z*)-((3-Bromo-4-phenylbut-3-en-2-yl)oxy)tributylsilane [(*S*)-7ch, 88.0% ee]

The enantiomeric excess of (*S*)-**7ch** was determined by the deprotected alcohol, which was hydrolyzed and purified by preparative TLC according to GP5, using the same HPLC analysis condition with **6c**.

Supplementary Figure 101.

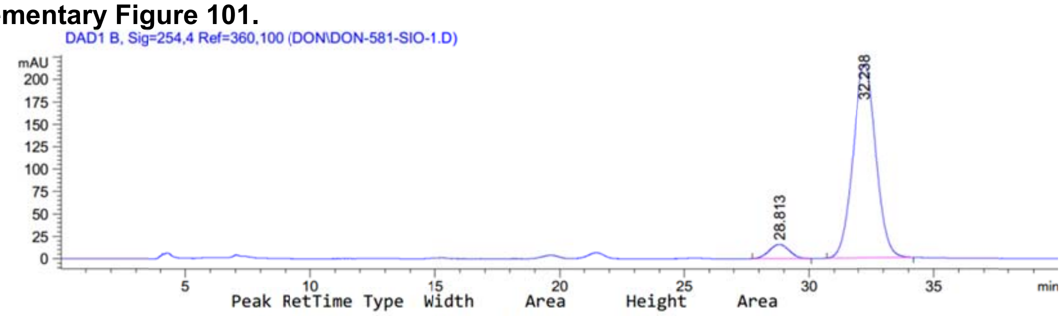

Peak #	RetTime [min]	Type	Width [min]	Area [mAU*s]	Height [mAU]	Area %
1	28.813	BB	0.6561	887.39050	15.87684	6.0246
2	32.238	BB	0.9559	1.38420e4	215.92123	93.9754

(*R*)-1-(Cyclohex-1-en-1-yl)ethan-1-ol [(*R*)-6d]

The enantiomeric excess of (*R*)-**6d** was determined by HPLC analysis on a chiral stationary phase (Daicel Chiralcel AD-H column, column temperature 20°C, solvent *n*-heptane:isopropanol = 98:2, flow rate 0.8 mL/min, λ = 210 nm): t_R = 13.4 min for (*S*)-**6d**, t_R = 14.3 min for (*R*)-**6d**.

Supplementary Figure 102. *rac-1-(Cyclohex-1-en-1-yl)ethan-1-ol [rac-***6d***]*

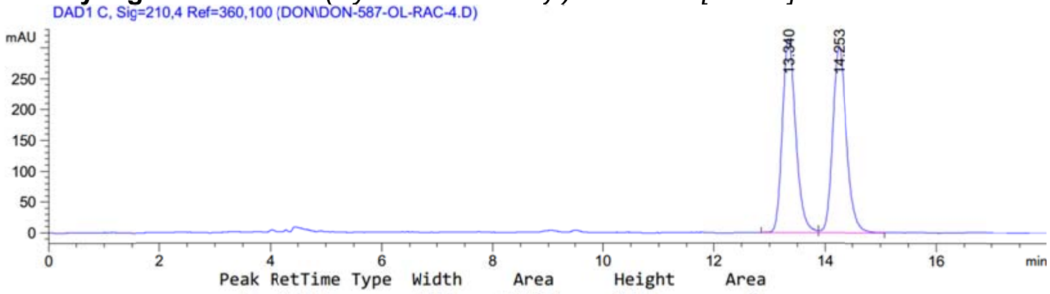

Peak #	RetTime [min]	Type	Width [min]	Area [mAU*s]	Height [mAU]	Area %
1	13.340	BV	0.2563	5245.58691	314.99350	49.6871
2	14.253	VV	0.2733	5311.66455	301.91083	50.3129

Supplementary Figure 103. *(R)-1-(Cyclohex-1-en-1-yl)ethan-1-ol [(R)-***6d***, 68.0% ee]*

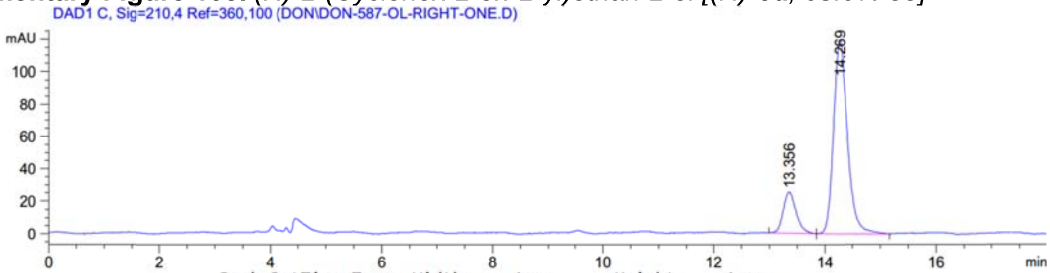

Peak #	RetTime [min]	Type	Width [min]	Area [mAU*s]	Height [mAU]	Area %
1	13.356	BB	0.2405	402.77759	25.19739	16.0452
2	14.269	BB	0.2715	2107.48535	119.65067	83.9548

(*S*)-Tributyl(1-(cyclohex-1-en-1-yl)ethoxy)silane [(*S*)-7dh, 75.6% ee]

The enantiomeric excess of (*S*)-**7dh** was determined by the deprotected alcohol, which was hydrolyzed and purified by preparative TLC according to GP5, using the same HPLC analysis condition with **6d**.

Supplementary Figure 104.

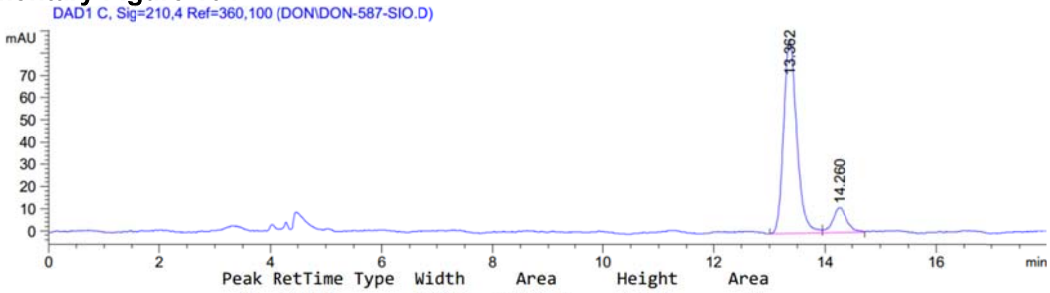

Peak #	RetTime [min]	Type	Width [min]	Area [mAU*s]	Height [mAU]	Area %
1	13.362	BV	0.2490	1429.68542	87.33870	87.8304
2	14.260	VB	0.2370	198.09476	11.05923	12.1696

(*R*,*E*)-2-Benzylidenecyclopentan-1-ol [(*R*)-6e]

The enantiomeric excess of (*R*)-**6e** was determined by HPLC analysis on a chiral stationary phase (Daicel Chiralcel OD-H column, column temperature 20°C, solvent *n*-heptane:isopropanol = 95:5, flow rate 0.8 mL/min, λ = 254 nm): t_R = 22.4 min for (*R*)-**6e**, t_R = 24.6 min for (*S*)-**6e**.

Supplementary Figure 105. *rac-(E)-2-Benzylidenecyclopentan-1-ol [rac-***6e***]*

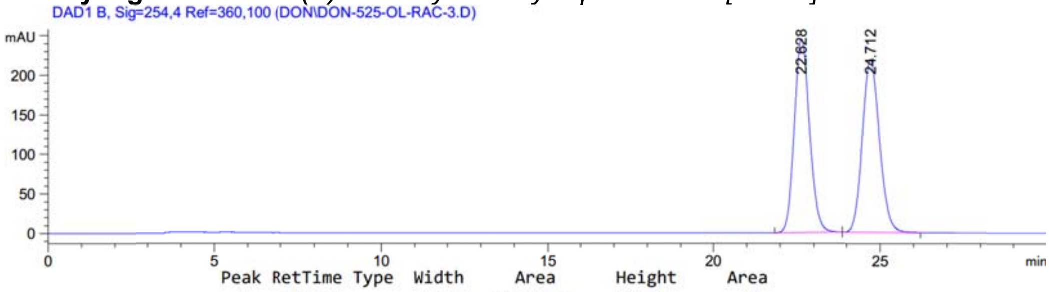

Peak #	RetTime [min]	Type	Width [min]	Area [mAU*s]	Height [mAU]	Area %
1	22.628	BB	0.4923	7782.62305	245.22208	50.0315
2	24.712	BB	0.5509	7772.81885	218.56743	49.9685

Supplementary Figure 106. *(R,E)-2-Benzylidenecyclopentan-1-ol [(R)-***6e***, 93.2% ee]*

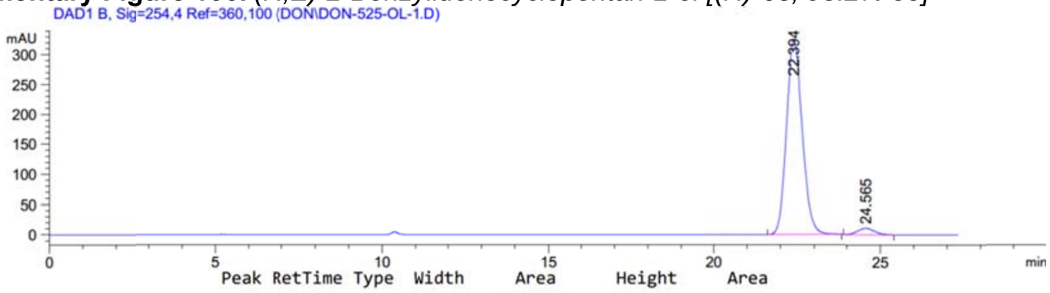

Peak #	RetTime [min]	Type	Width [min]	Area [mAU*s]	Height [mAU]	Area %
1	22.394	BB	0.5096	1.06182e4	324.63034	96.6417
2	24.565	BB	0.4214	368.98526	10.56666	3.3583

(*S*,*E*)-((2-Benzylidenecyclopentyl)oxy)tributylsilane [(*S*)-7eh, 90.6% ee]

The enantiomeric excess of (*S*)-**7eh** was determined by the deprotected alcohol, which was hydrolyzed and purified by preparative TLC according to GP5, using the same HPLC analysis condition with **6e**.

Supplementary Figure 107.

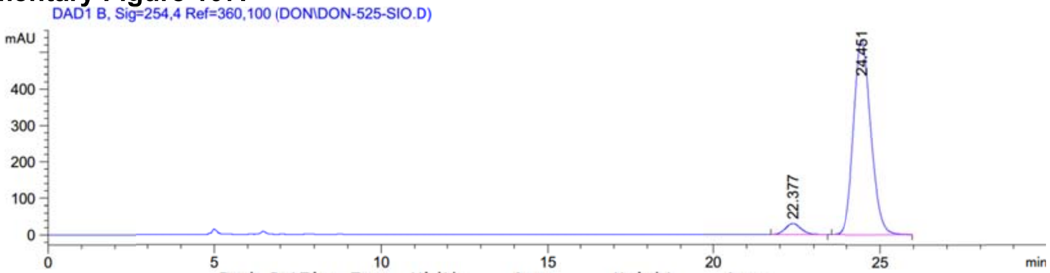

Peak #	RetTime [min]	Type	Width [min]	Area [mAU*s]	Height [mAU]	Area %
1	22.377	BB	0.4886	958.35236	29.84472	4.7437
2	24.451	BBA	0.5612	1.92442e4	532.99902	95.2563

(*R*,*E*)-2-Benzylidenecyclopentan-1-ol [(*R*)-6e]

The enantiomeric excess of (*R*)-**6e** was determined by HPLC analysis on a chiral stationary phase (Daicel Chiralcel OD-H column, column temperature 20°C, solvent *n*-heptane:isopropanol = 95:5, flow rate 0.8 mL/min, λ = 254 nm): t_R = 22.3 min for (*R*)-**6e**, t_R = 24.5 min for (*S*)-**6e**.

Supplementary Figure 108. *rac-(E)-2-Benzylidenecyclopentan-1-ol [rac-***6e***]*

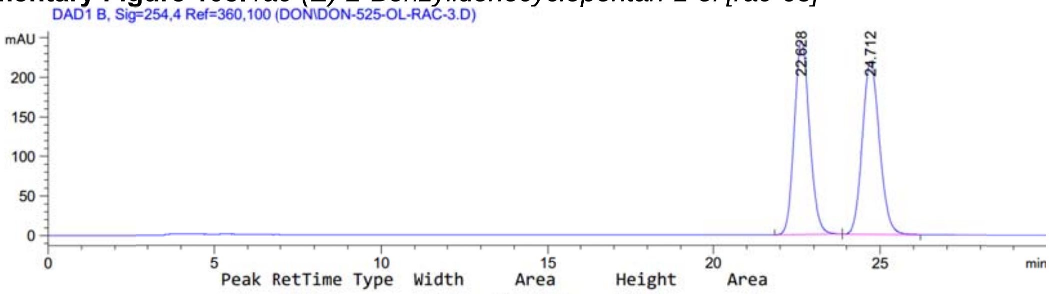

Peak #	RetTime [min]	Type	Width [min]	Area [mAU*s]	Height [mAU]	Area %
1	22.628	BB	0.4923	7782.62305	245.22208	50.0315
2	24.712	BB	0.5509	7772.81885	218.56743	49.9685

Supplementary Figure 109. *(R,E)-2-Benzylidenecyclopentan-1-ol [(R)-***6e***, 99.0% ee]*

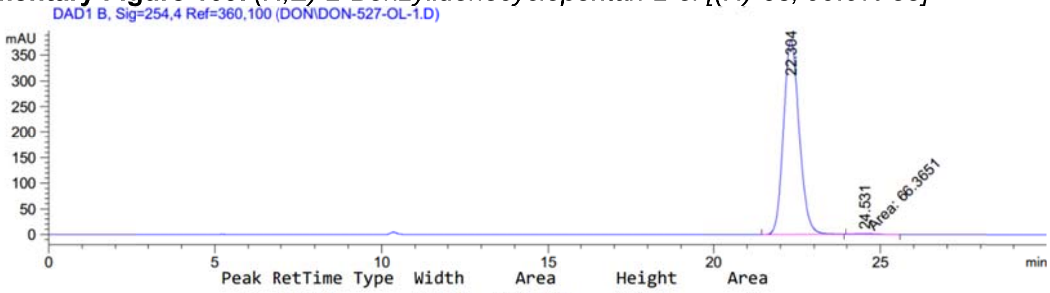

Peak #	RetTime [min]	Type	Width [min]	Area [mAU*s]	Height [mAU]	Area %
1	22.304	BB	0.5009	1.24019e4	379.86530	99.4677
2	24.531	MM	0.6407	66.36509	1.72642	0.5323

(*S*,*E*)-((2-Benzylidenecyclopentyl)oxy)tributylsilane [(*S*)-7eh, 81.0% ee]

The enantiomeric excess of (*S*)-**7eh** was determined by the deprotected alcohol, which was hydrolyzed and purified by preparative TLC according to GP5, using the same HPLC analysis condition with **6e**.

Supplementary Figure 110.

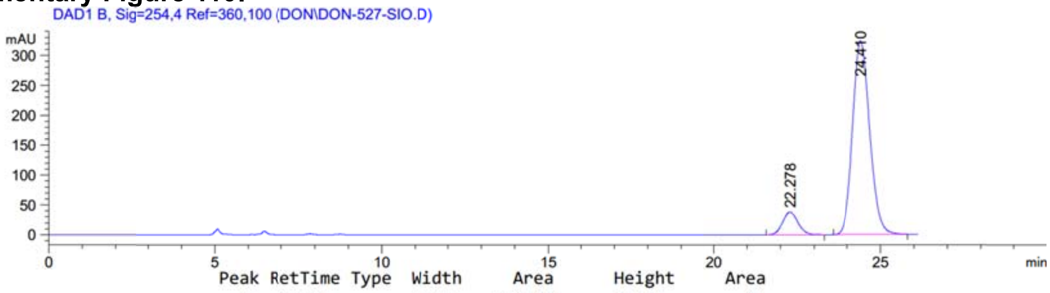

Peak #	RetTime [min]	Type	Width [min]	Area [mAU*s]	Height [mAU]	Area %
1	22.278	BB	0.4793	1219.55493	37.91250	9.5413
2	24.410	BB	0.5545	1.15623e4	323.87396	90.4587

(*R*,*E*)-2-Benzylidenecyclohexan-1-ol [(*R*)-6f]

The enantiomeric excess of (*R*)-**6f** was determined by HPLC analysis on a chiral stationary phase (Daicel Chiralcel OD-H column, column temperature 20°C, solvent *n*-heptane:isopropanol = 95:5, flow rate 0.8 mL/min, λ = 254 nm): t_R = 15.8 min for (*R*)-**6f**, t_R = 20.7 min for (*S*)-**6f**.

Supplementary Figure 111. *rac-(E)-2-Benzylidenecyclohexan-1-ol [rac-***6f***]*

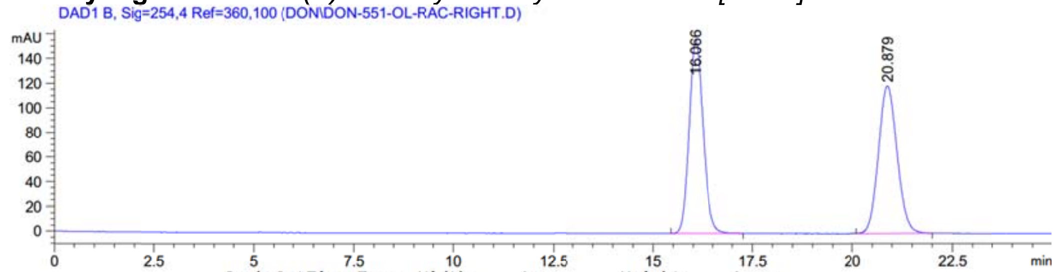

Peak #	RetTime [min]	Type	Width [min]	Area [mAU*s]	Height [mAU]	Area %
1	16.066	BB	0.3806	3859.81567	157.10033	50.0637
2	20.879	BB	0.4976	3849.99390	119.57866	49.9363

Supplementary Figure 112. *(R,E)-2-Benzylidenecyclohexan-1-ol [(R)-***6f***, 98.6% ee]*

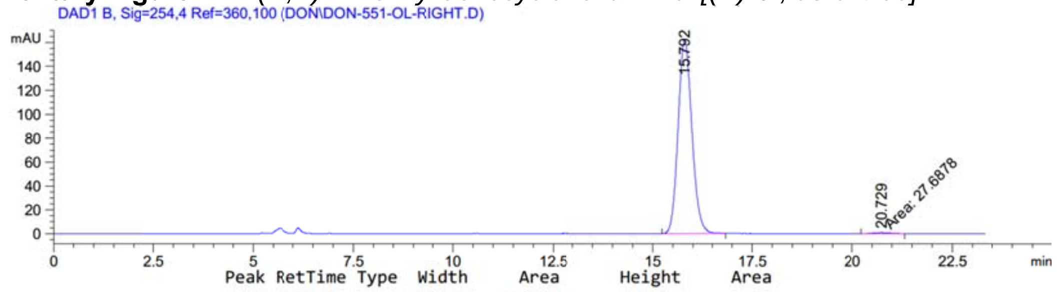

Peak #	RetTime [min]	Type	Width [min]	Area [mAU*s]	Height [mAU]	Area %
1	15.792	BB	0.3707	3871.23828	162.07228	99.2899
2	20.729	MM	0.4942	27.68783	9.33676e-1	0.7101

(*S*,*E*)-((2-Benzylidenecyclohexyl)oxy)tributylsilane [(*S*)-7fh, 94.0% ee]

The enantiomeric excess of (*S*)-**7fh** was determined by the deprotected alcohol, which was hydrolyzed and purified by preparative TLC according to GP5, using the same HPLC analysis condition with **6f**.

Supplementary Figure 113.

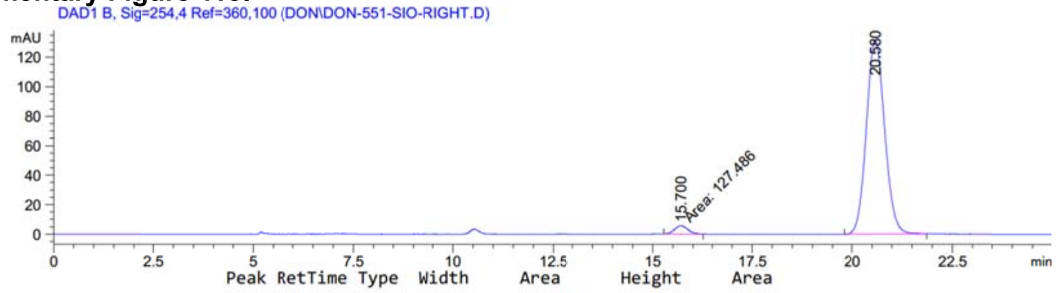

Peak #	RetTime [min]	Type	Width [min]	Area [mAU*s]	Height [mAU]	Area %
1	15.700	MM	0.3796	127.48616	5.59704	2.9765
2	20.580	BB	0.4938	4155.59277	131.82275	97.0235

(*R*)-2,3,4,5-Tetrahydro-[1,1'-biphenyl]-2-ol [(*R*)-8]

The enantiomeric excess of (*R*)-**8** was determined by HPLC analysis on a chiral stationary phase (Daicel Chiralcel OD-H column, column temperature 20°C, solvent *n*-heptane:isopropanol = 95:5, flow rate 0.8 mL/min, λ = 254 nm): t_R = 12.2 min for (*R*)-**8**, t_R = 16.5 min for (*S*)-**8**.

Supplementary Figure 114. *rac-2,3,4,5-Tetrahydro-[1,1'-biphenyl]-2-ol [rac-**8**]*

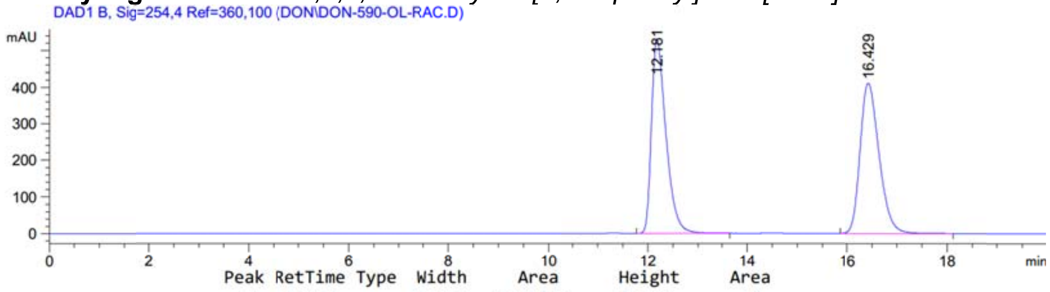

Peak #	RetTime [min]	Type	Width [min]	Area [mAU*s]	Height [mAU]	Area %
1	12.181	BB	0.3049	1.07138e4	532.16376	49.9003
2	16.429	BB	0.4031	1.07566e4	411.35016	50.0997

Supplementary Figure 115. *(R)-2,3,4,5-Tetrahydro-[1,1'-biphenyl]-2-ol [(R)-**8**, 66.6% ee]*

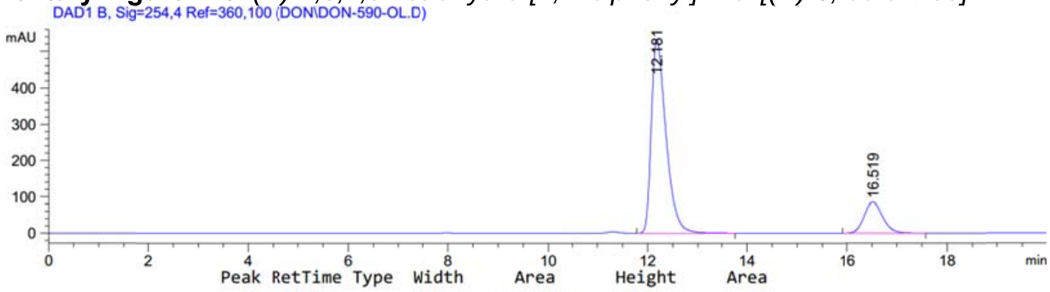

Peak #	RetTime [min]	Type	Width [min]	Area [mAU*s]	Height [mAU]	Area %
1	12.181	BB	0.3089	1.08480e4	534.29492	83.2848
2	16.519	BB	0.3941	2177.18750	85.22038	16.7152

(*S*)-Tributyl((2,3,4,5-tetrahydro-[1,1'-biphenyl]-2-yl)oxy)silane [(*S*)-9h, 94.2% ee]

The enantiomeric excess of (*S*)-**9h** was determined by the deprotected alcohol, which was hydrolyzed and purified by preparative TLC according to GP5, using the same HPLC analysis condition with **8**.

Supplementary Figure 116.

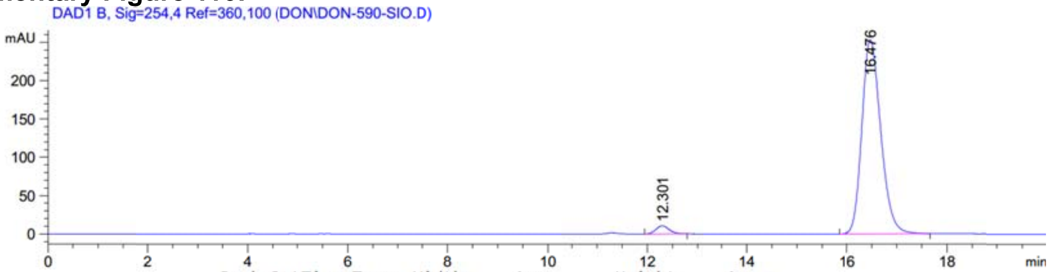

Peak #	RetTime [min]	Type	Width [min]	Area [mAU*s]	Height [mAU]	Area %
1	12.301	BB	0.2785	195.16020	10.61708	2.8752
2	16.476	BB	0.4015	6592.52588	253.41852	97.1248

(*R*)-2,3,4,5-Tetrahydro-[1,1'-biphenyl]-2-ol [(*R*)-8]

The enantiomeric excess of (*R*)-**8** was determined by HPLC analysis on a chiral stationary phase (Daicel Chiralcel OD-H column, column temperature 20°C, solvent *n*-heptane:isopropanol = 95:5, flow rate 0.8 mL/min, λ = 254 nm): t_R = 16.0 min for (*R*)-**8**, t_R = 21.5 min for (*S*)-**8**.

Supplementary Figure 117. *rac-2,3,4,5-Tetrahydro-[1,1'-biphenyl]-2-ol [rac-**8**]*

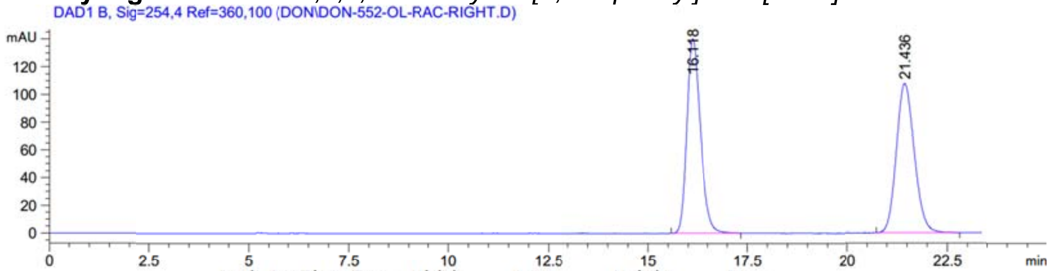

Peak #	RetTime [min]	Type	Width [min]	Area [mAU*s]	Height [mAU]	Area %
1	16.118	BB	0.3685	3343.58105	140.06668	50.0059
2	21.436	BB	0.4780	3342.79175	107.72184	49.9941

Supplementary Figure 118. *(R)-2,3,4,5-Tetrahydro-[1,1'-biphenyl]-2-ol [(R)-**8**, 76.8% ee]*

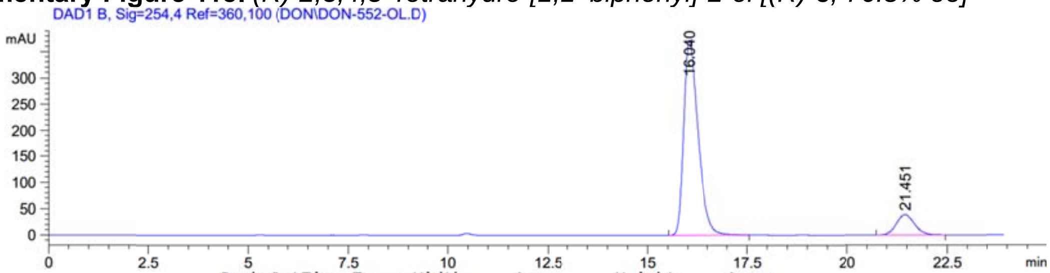

Peak #	RetTime [min]	Type	Width [min]	Area [mAU*s]	Height [mAU]	Area %
1	16.040	BB	0.3847	9316.48633	371.29388	88.3866
2	21.451	BB	0.4651	1224.11841	39.11933	11.6134

(*S*)-Tributyl((2,3,4,5-tetrahydro-[1,1'-biphenyl]-2-yl)oxy)silane [(*S*)-9h, 92.0% ee]

The enantiomeric excess of (*S*)-**9h** was determined by the deprotected alcohol, which was hydrolyzed and purified by preparative TLC according to GP5, using the same HPLC analysis condition with **8**.

Supplementary Figure 119.

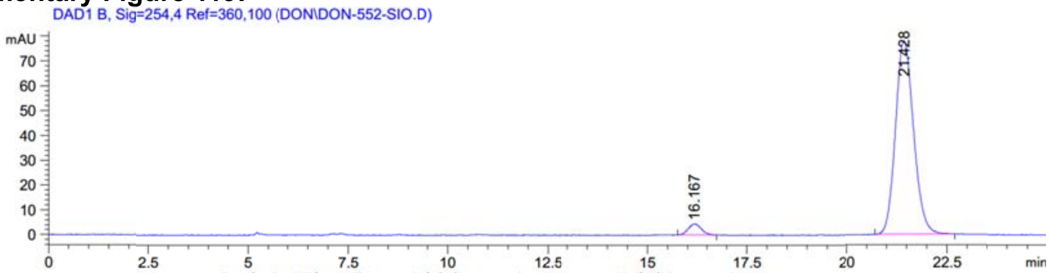

Peak #	RetTime [min]	Type	Width [min]	Area [mAU*s]	Height [mAU]	Area %
1	16.167	BB	0.2912	102.44660	4.44064	4.0241
2	21.428	BB	0.4834	2443.39917	78.00597	95.9759

(*R*)-1-Cyclohexylethan-1-ol [(*R*)-10]

The enantiomeric excess of (*R*)-**10** was determined by HPLC analysis of the correspongding 1-cyclohexylethyl 4-nitrobenzoate derivative on a chiral stationary phase (Daicel Chiralcel ID column, column temperature 20°C, solvent *n*-heptane:isopropanol = 99:1, flow rate 0.8 mL/min, λ = 254 nm): t_R = 10.7 min for (*S*)-**10**, t_R = 11.8 min for (*R*)-**10**.

Supplementary Figure 120. *rac-1-cyclohexylethan-1-ol [rac-10]*

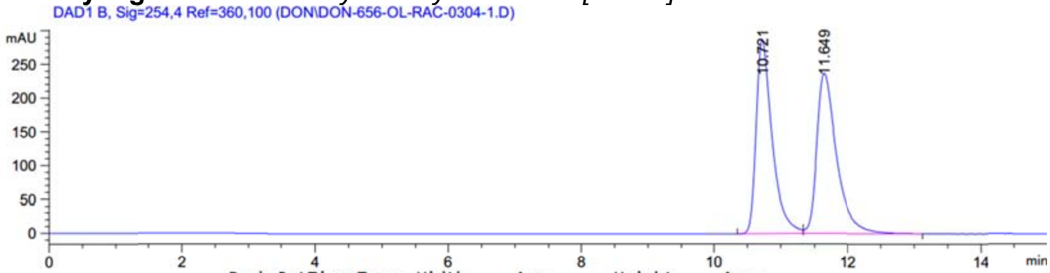

Peak #	RetTime [min]	Type	Width [min]	Area [mAU*s]	Height [mAU]	Area %
1	10.721	BV	0.2508	4813.73584	288.32990	49.3557
2	11.649	VB	0.3086	4939.41553	237.56720	50.6443

Supplementary Figure 121. *(R)-1-Cyclohexylethan-1-ol [(R)-10, 74.2% ee]*

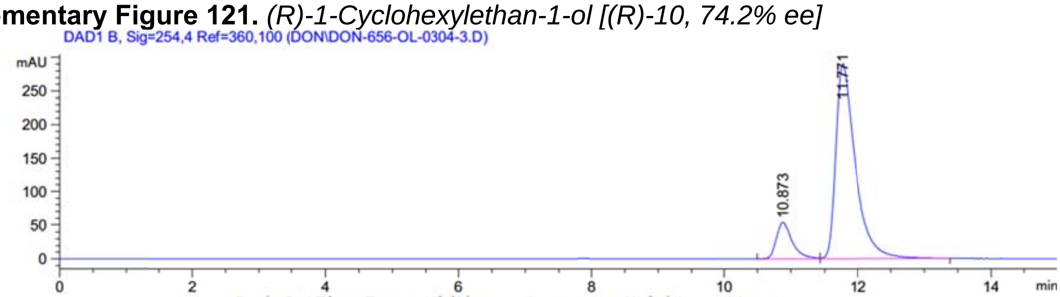

Peak #	RetTime [min]	Type	Width [min]	Area [mAU*s]	Height [mAU]	Area %
1	10.873	BV	0.2474	887.68396	53.55872	12.9249
2	11.771	VB	0.3061	5980.31348	290.61038	87.0751

(*S*)-Tributyl(1-cyclohexylethoxy)silane [(*S*)-11h, 63.2% ee]

The enantiomeric excess of (*S*)-**11h** was determined by corresponding 1-cyclohexylethyl 4-nitrobenzoate derivative using the following HPLC setup (Daicel Chiralcel ID column, column temperature 20°C, solvent *n*-heptane:isopropanol = 99:1, flow rate 0.8 mL/min, λ = 254 nm): t_R = 10.7 min for (*S*)-**10**, t_R = 11.8 min for (*R*)-**10**.

Supplementary Figure 122.

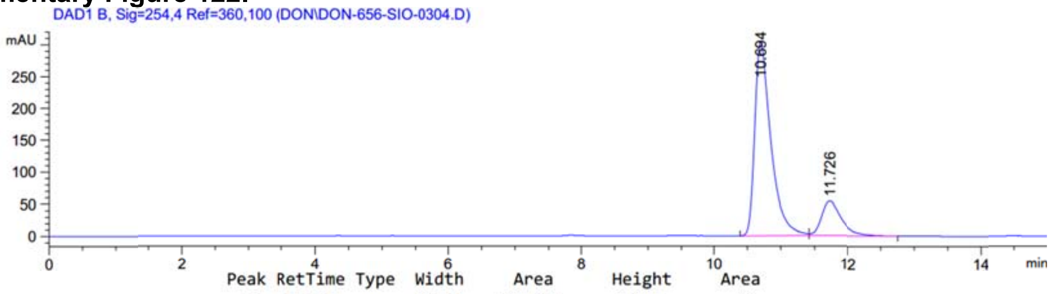

Peak #	RetTime [min]	Type	Width [min]	Area [mAU*s]	Height [mAU]	Area %
1	10.694	BV	0.2523	5140.08545	305.42062	81.7691
2	11.726	VB	0.3098	1146.01465	54.83178	18.2309

7 NMR Spectra

Supplementary Figure 123. ^{1}H **NMR (400 MHz,** C_6D_6**) of (*R*)-1-([1,1'-Biphenyl]-4-yl)ethan-1-ol [(*R*)-1a]**

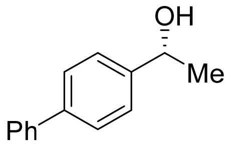

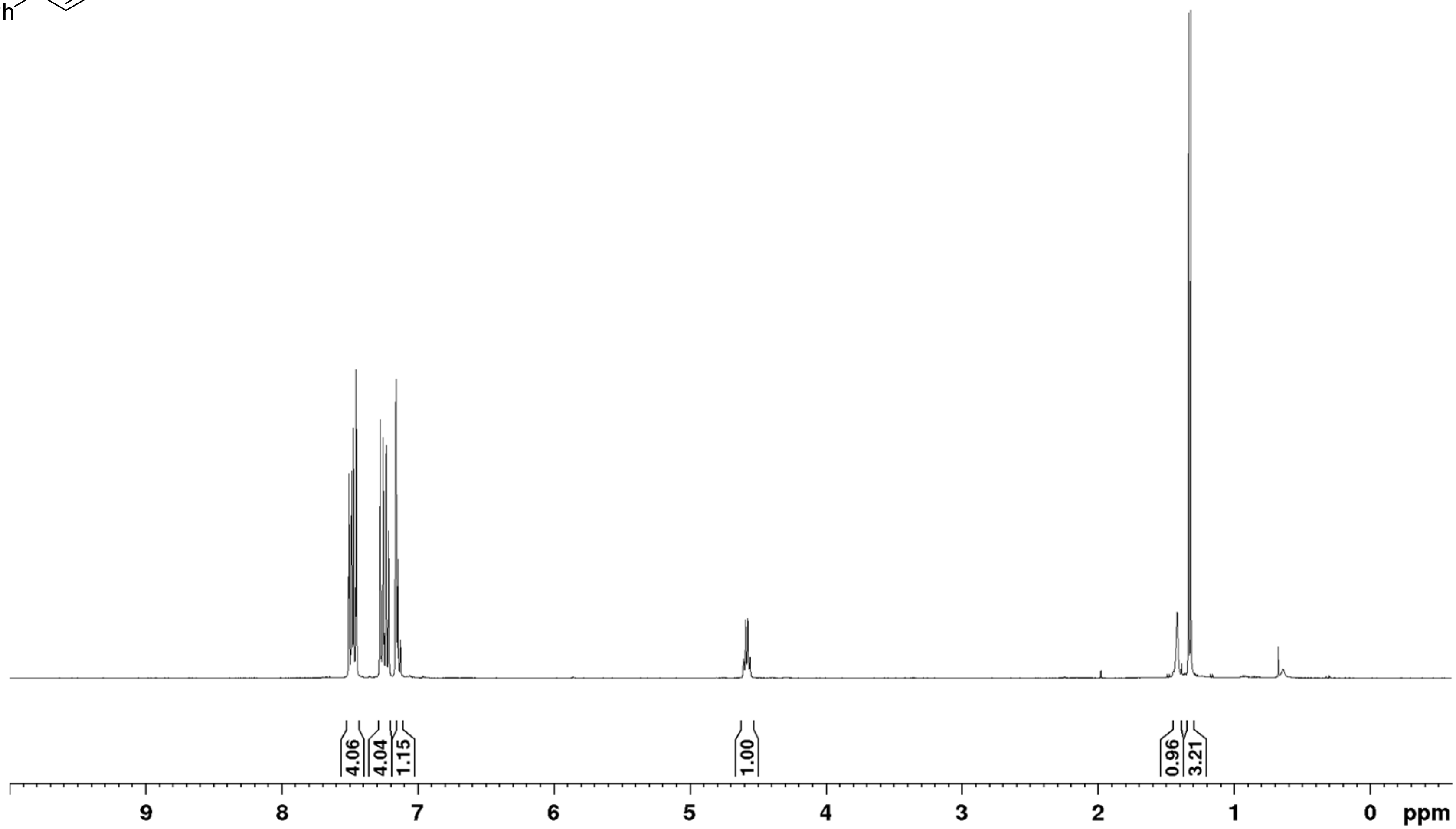

Supplementary Figure 124. ^{13}C NMR (126 MHz, $CDCl_3$) of (*R*)-1-([1,1'-Biphenyl]-4-yl)ethan-1-ol [(*R*)-1a]

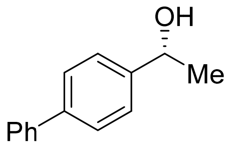

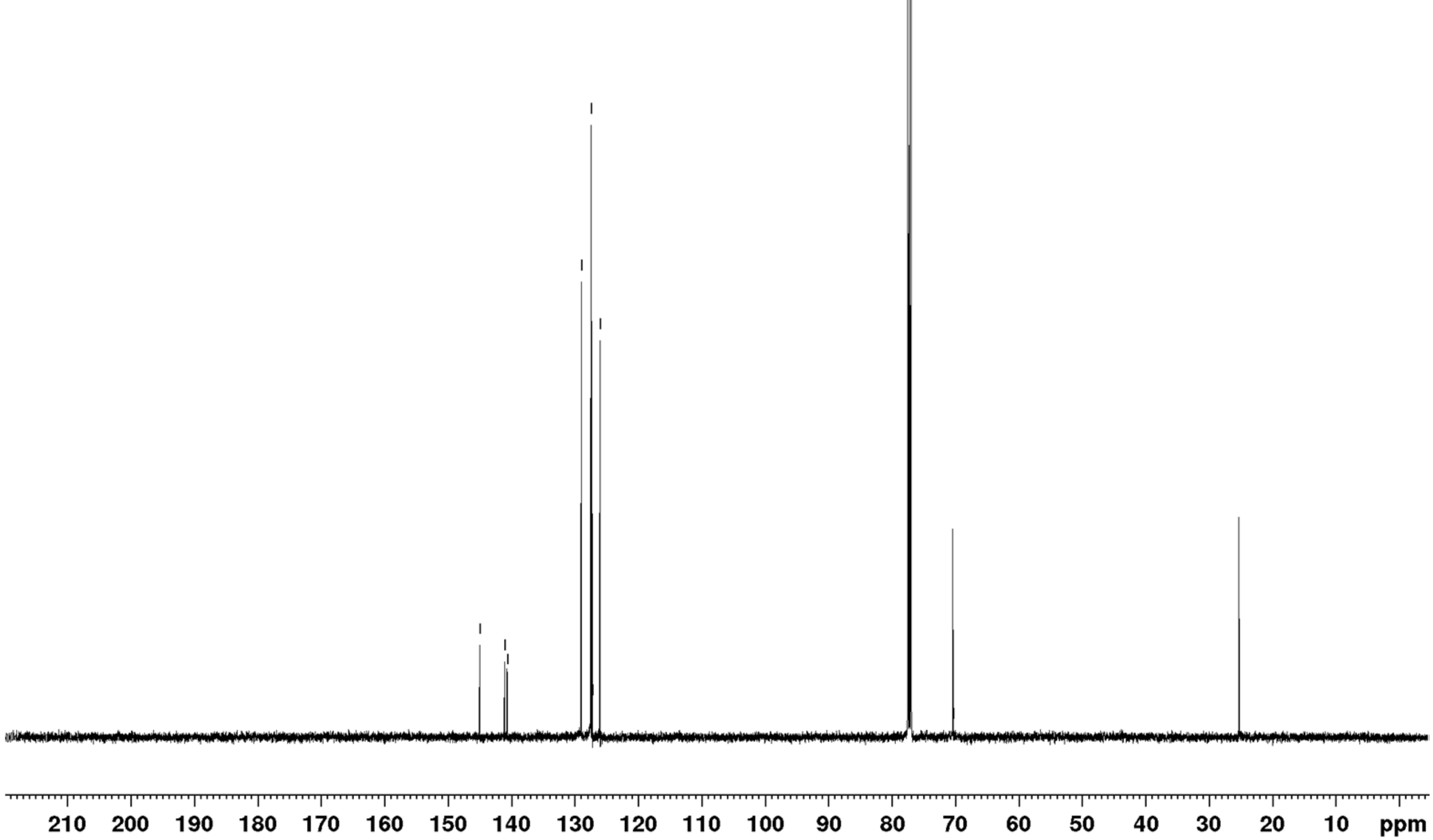

Supplementary Figure 125. ^{1}H NMR (400 MHz, C_6D_6) of (S)-(1-([1,1'-Biphenyl]-4-yl)ethoxy)tributylsilane [(*S*)-3ah]

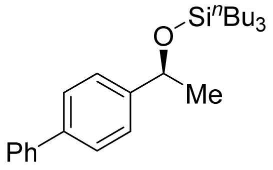

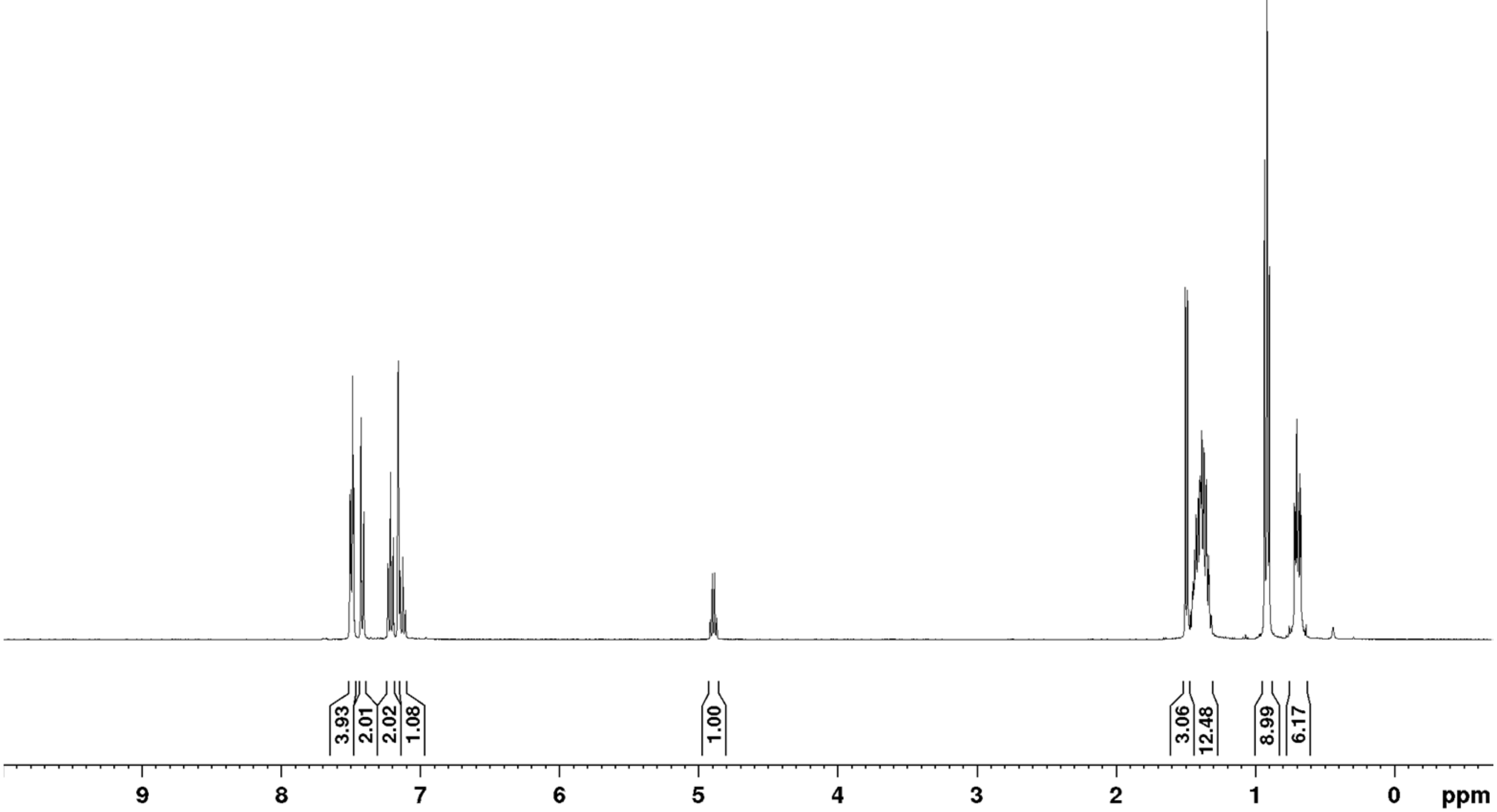

Supplementary Figure 126. ^{13}**C NMR (126 MHz, C_6D_6) of (*S*)-(1-([1,1'-Biphenyl]-4-yl)ethoxy)tributylsilane [(*S*)-3ah]**

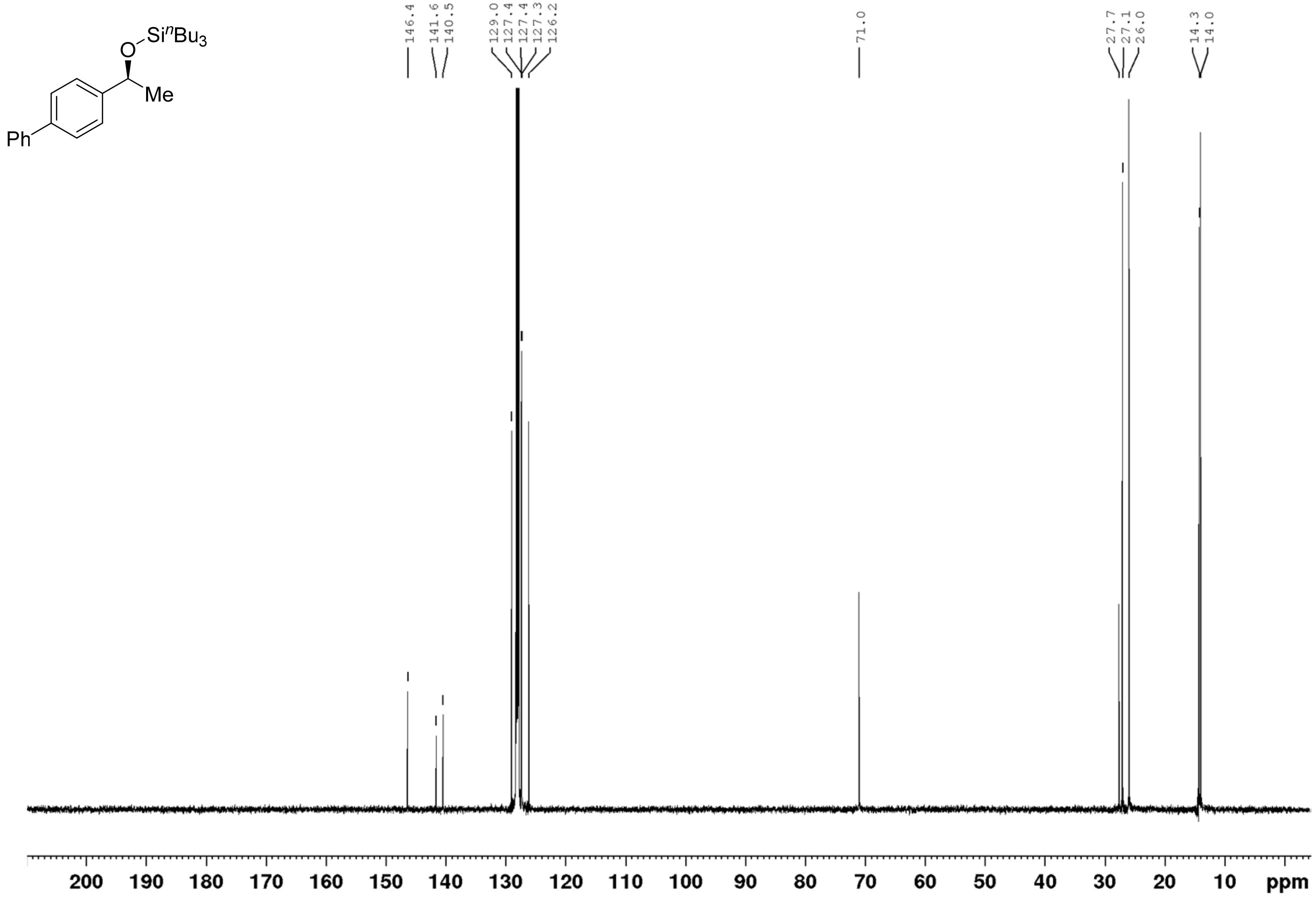

Supplementary Figure 127. ^{1}H NMR (500 MHz, $CDCl_3$) of (*R*)-1-Phenylethan-1-ol [(*R*)-1b]

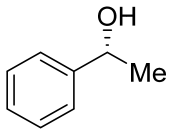

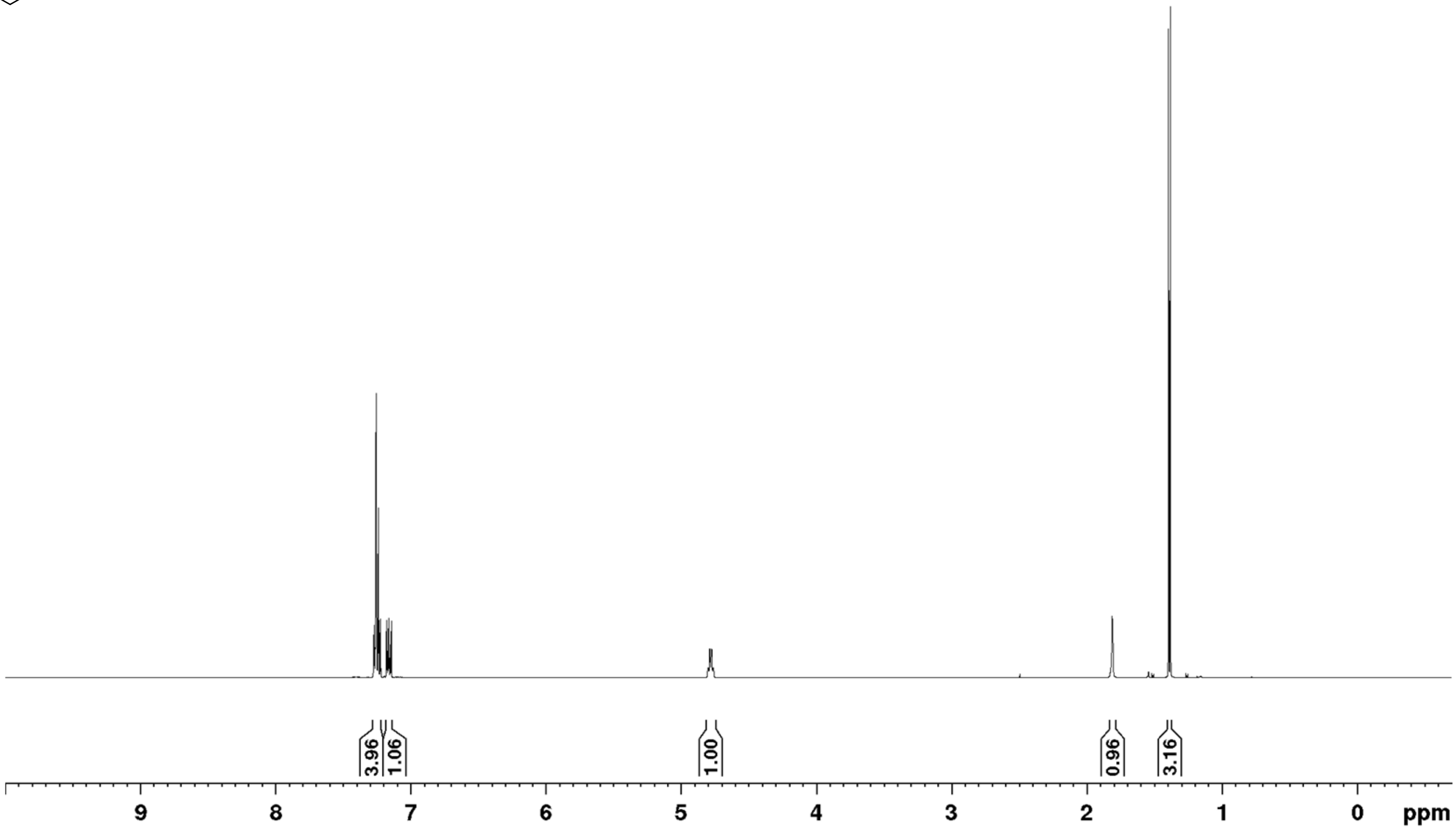

Supplementary Figure 128. ^{13}C NMR (126 MHz, $CDCl_3$) of (R)-1-Phenylethan-1-ol [(R)-1b]

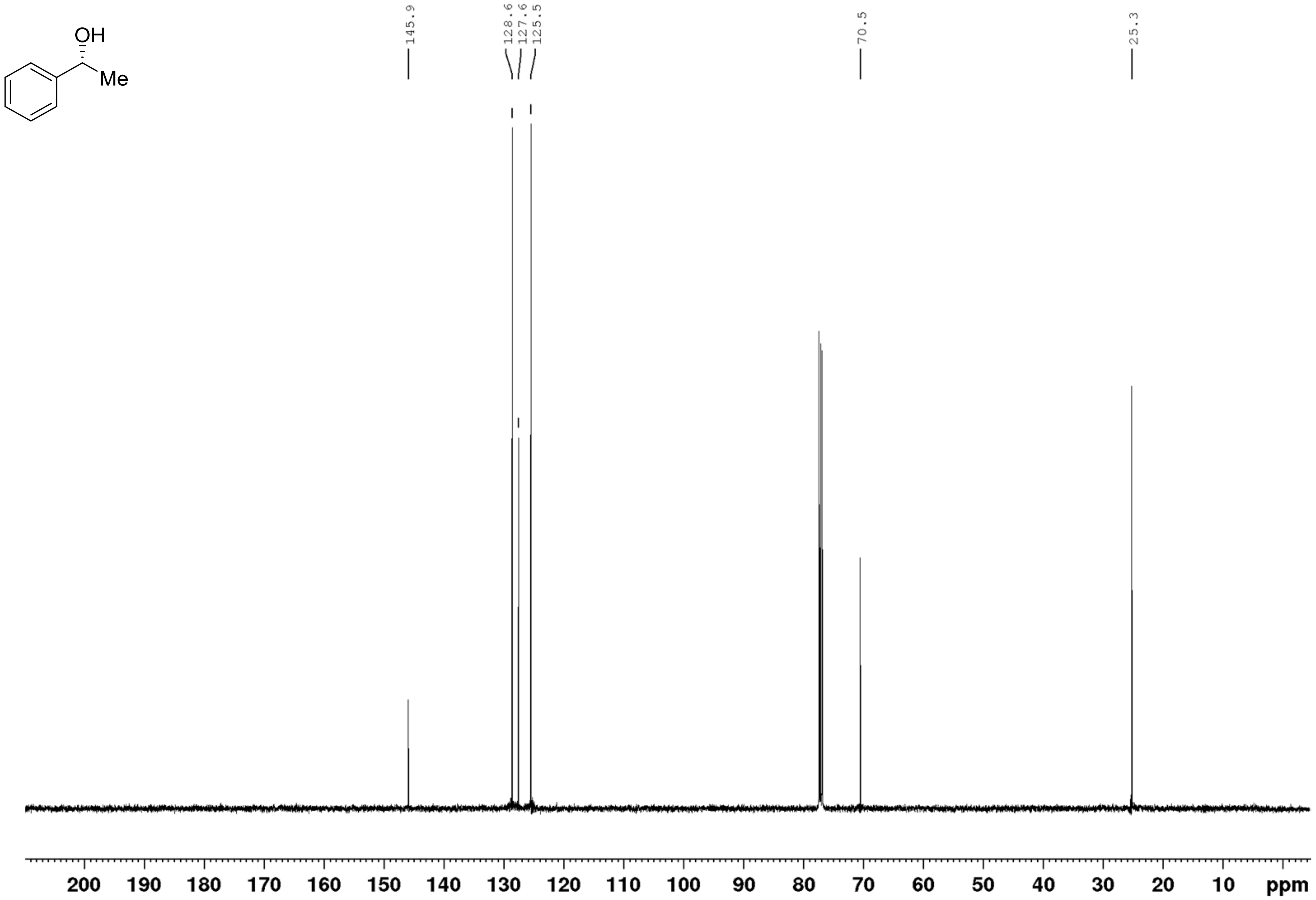

Supplementary Figure 129. **^{1}H NMR (400 MHz, C_6D_6) of (*S*)-Tributyl(1-phenylethoxy)silane [(*S*)-3bh]**

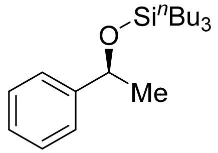

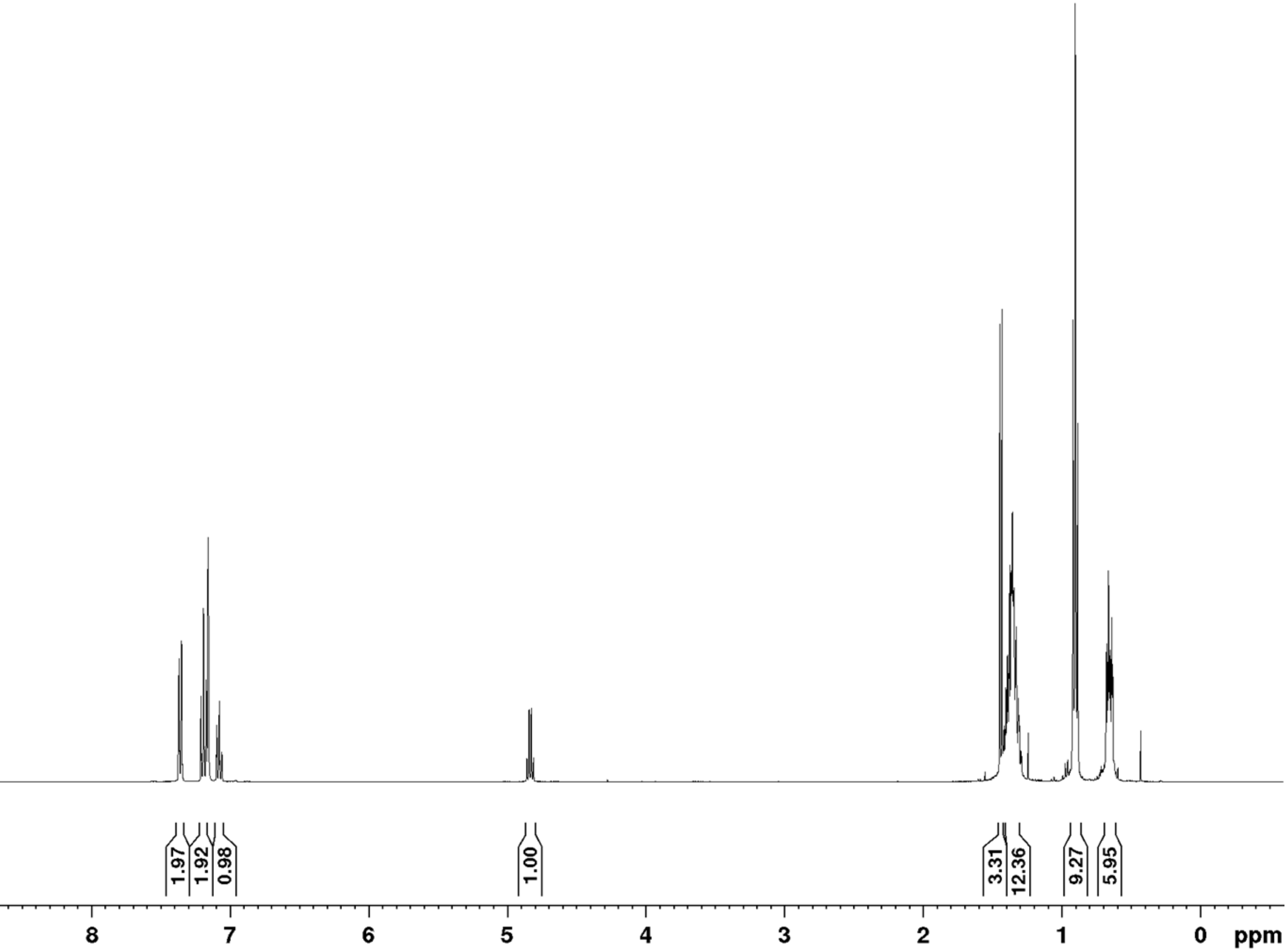

Supplementary Figure 130. ^{13}C NMR (126 MHz, C_6D_6) of (*S*)-Tributyl(1-phenylethoxy)silane [(*S*)-3bh]

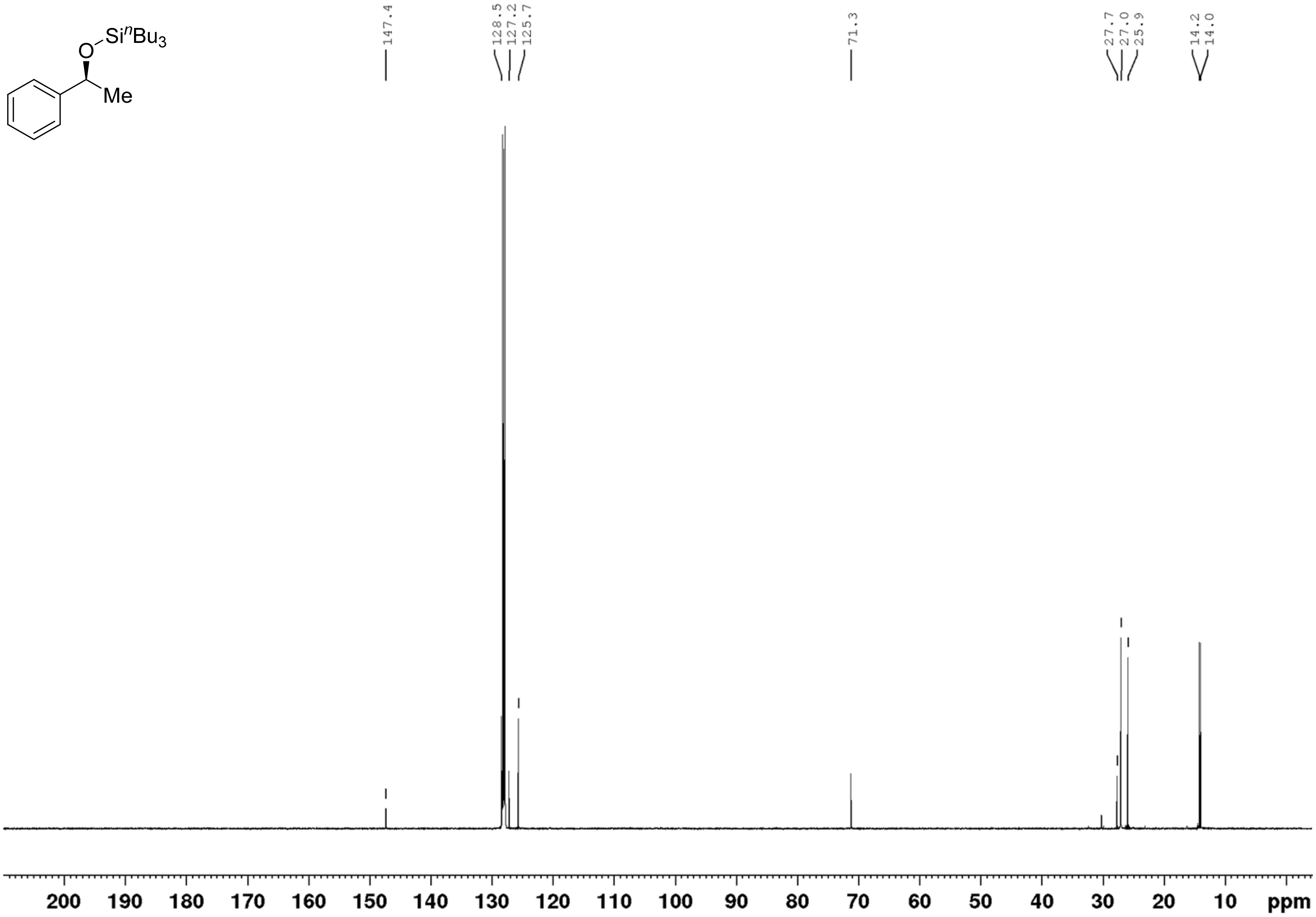

Supplementary Figure 131. ^{1}H NMR (500 MHz, $CDCl_3$) of (*R*)-1-(*p*-Tolyl)ethan-1-ol [(*R*)-1c]

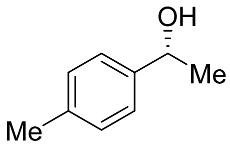

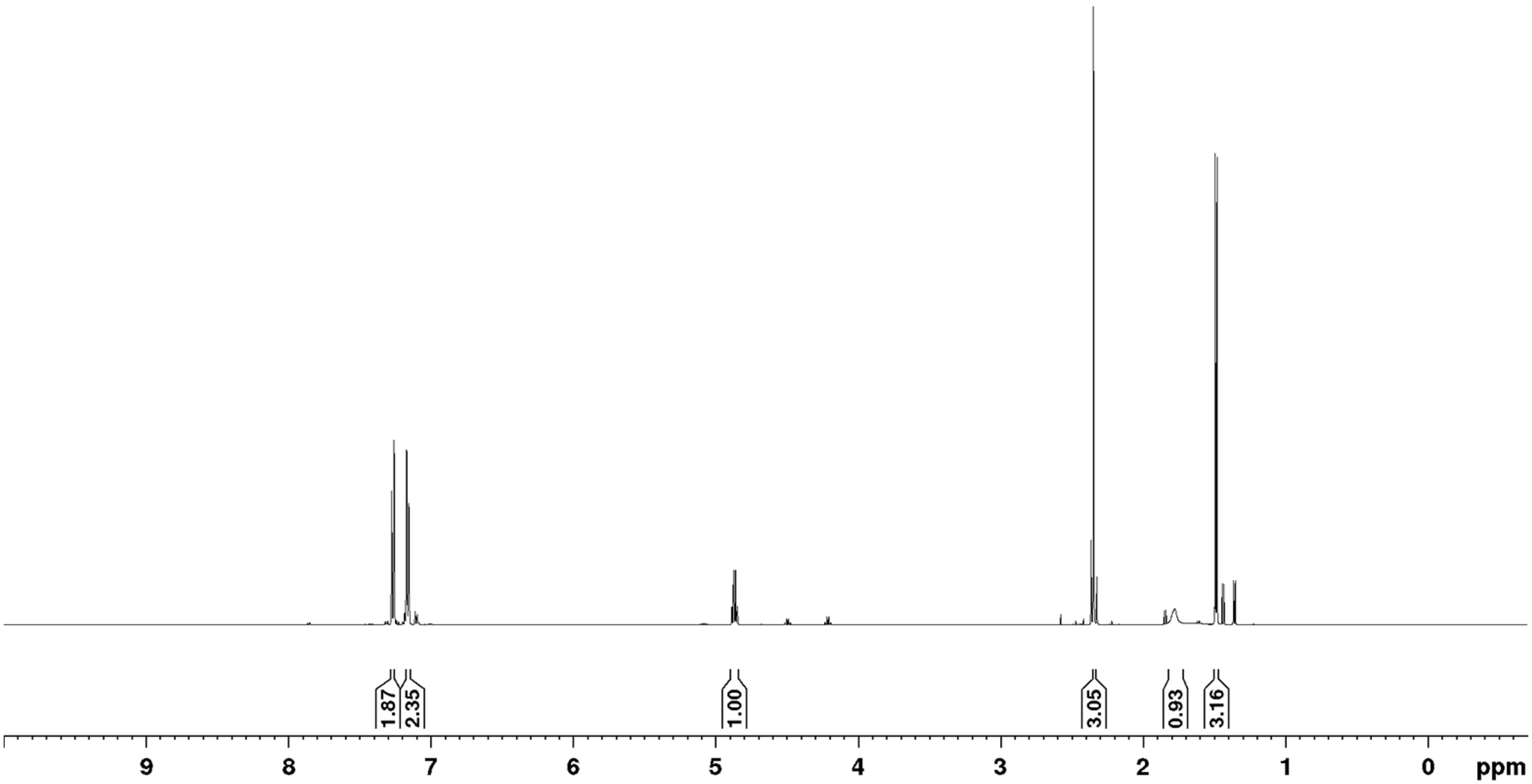

Supplementary Figure 132. **^{13}C NMR (126 MHz, $CDCl_3$) of (*R*)-1-(*p*-Tolyl)ethan-1-ol [(*R*)-1c]**

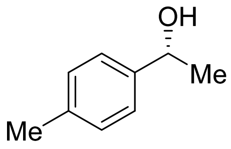

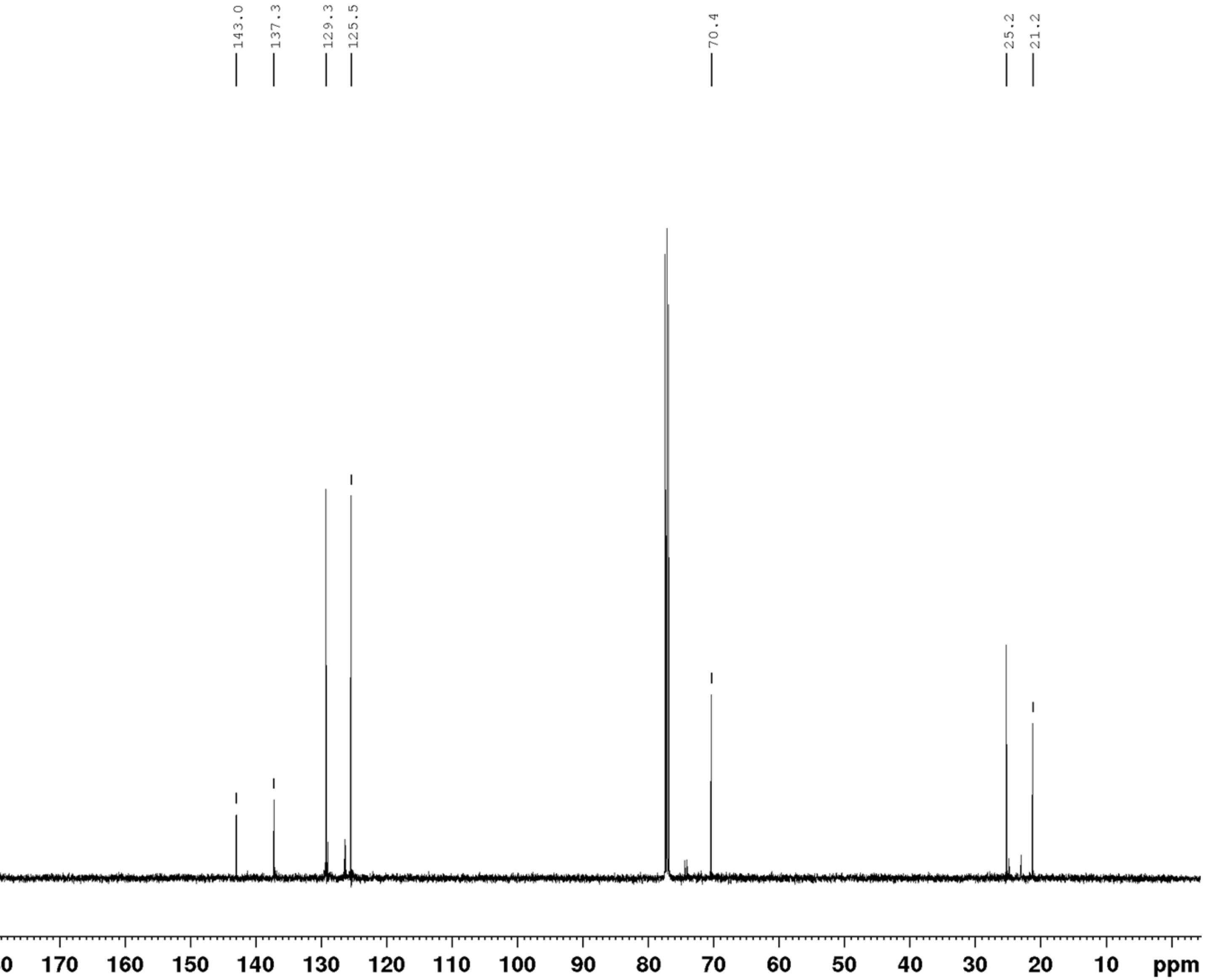

Supplementary Figure 133. **^{1}H NMR (500 MHz, C_6D_6) of (*S*)-Tributyl(1-(*p*-tolyl)ethoxy)silane [(*S*)-3ch]**

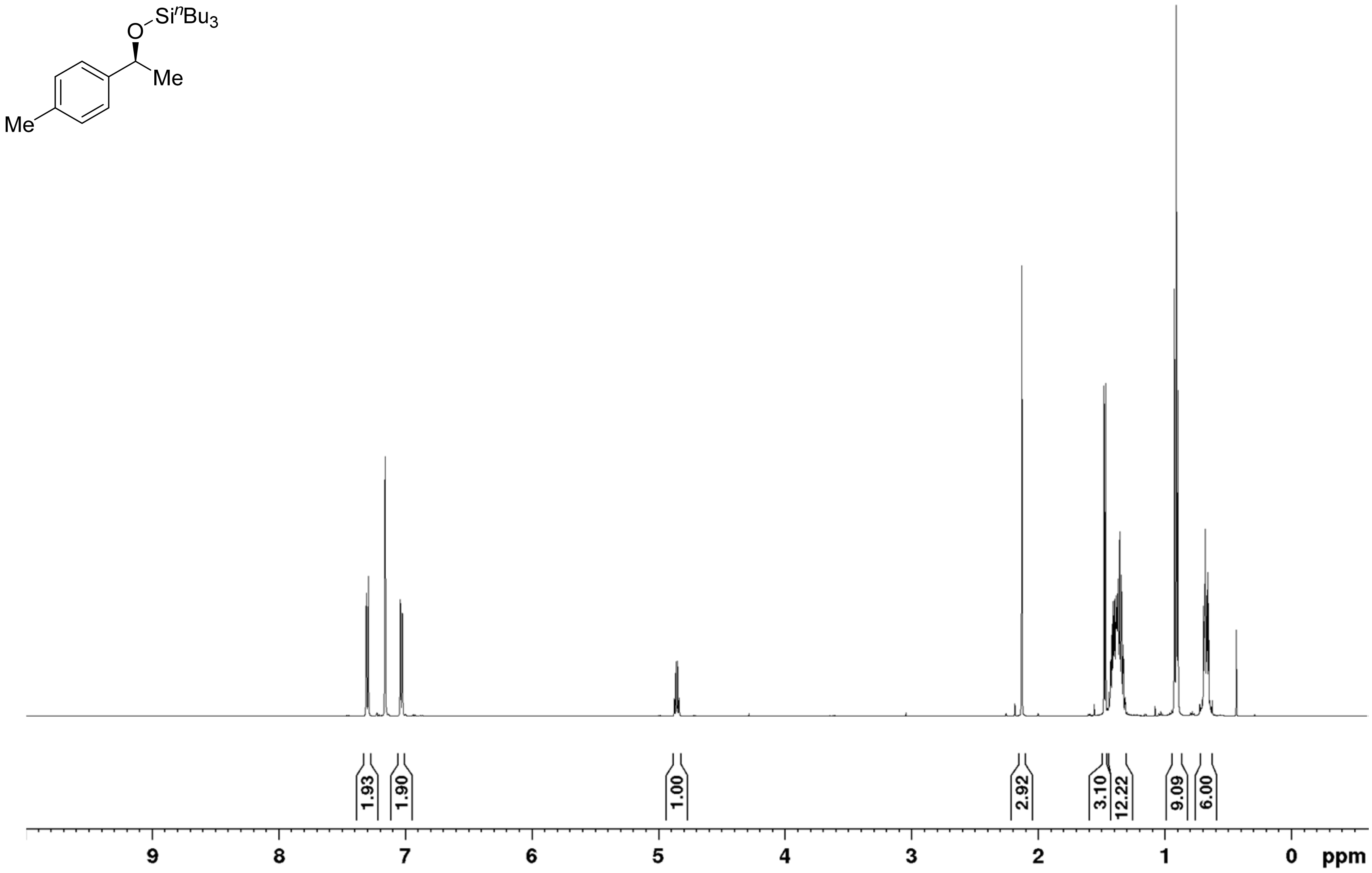

Supplementary Figure 134. ^{13}**C NMR (126 MHz, C_6D_6) of (*S*)-Tributyl(1-(*p*-tolyl)ethoxy)silane [(*S*)-3ch]**

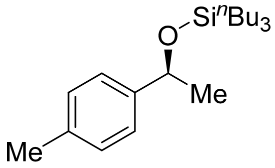

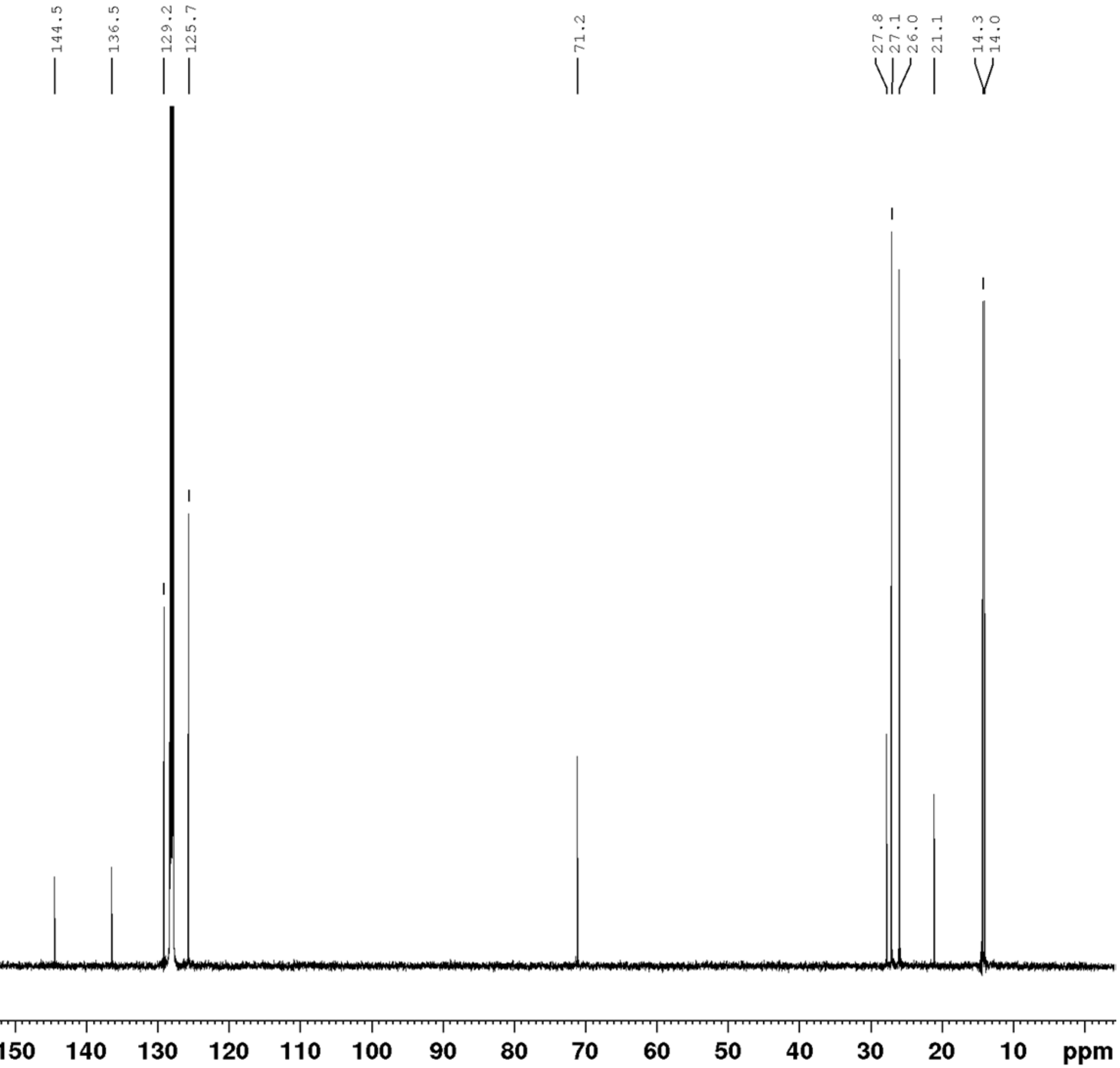

Supplementary Figure 135. ^{1}**H NMR (500 MHz, C_6D_6) of (*R*)-1-(*m*-Tolyl)ethan-1-ol [(*R*)-1d]**

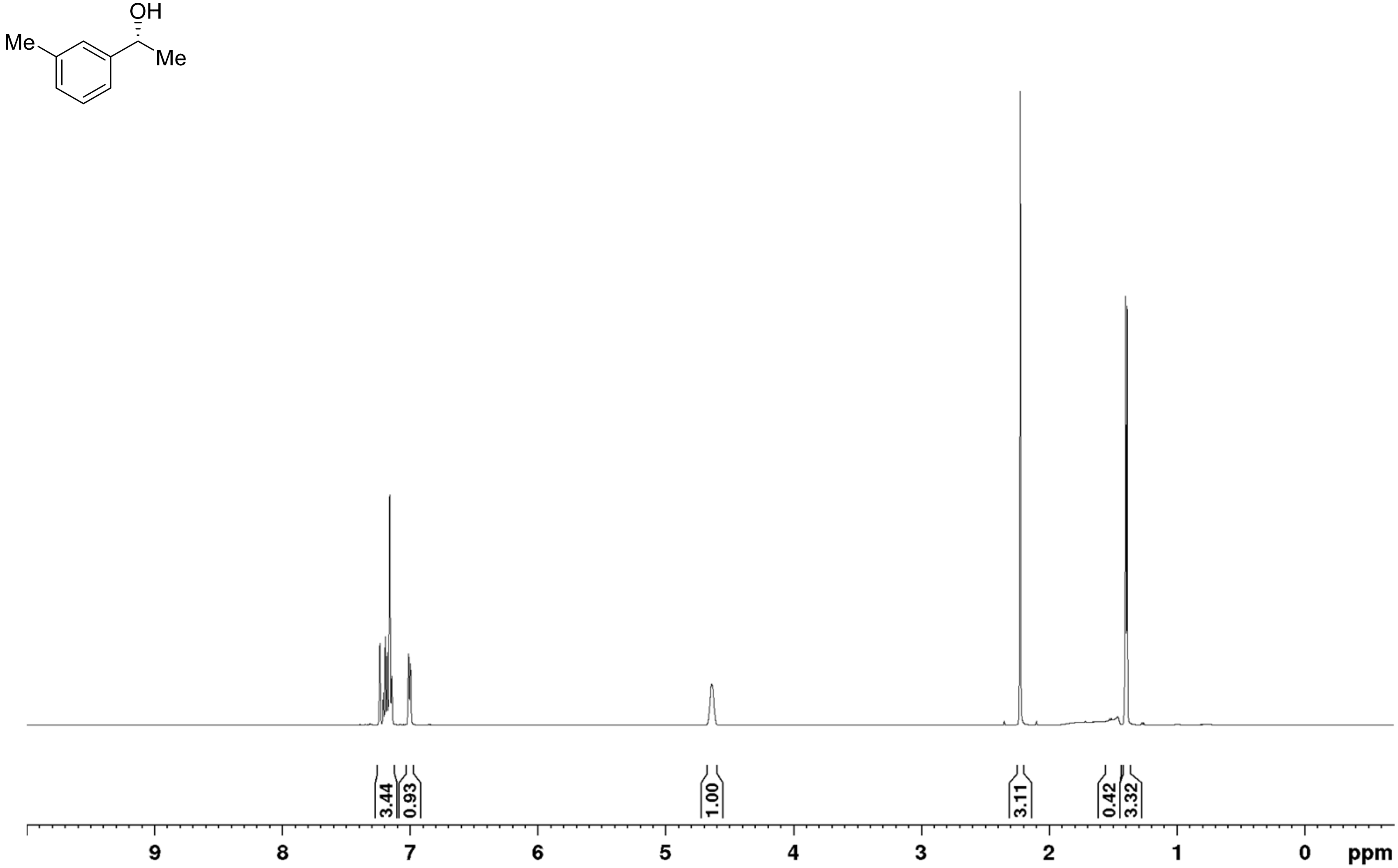

Supplementary Figure 136. ^{13}C NMR (126 MHz, C_6D_6) of (*R*)-1-(*m*-Tolyl)ethan-1-ol [(*R*)-1d]

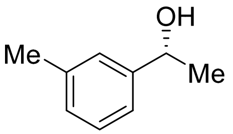

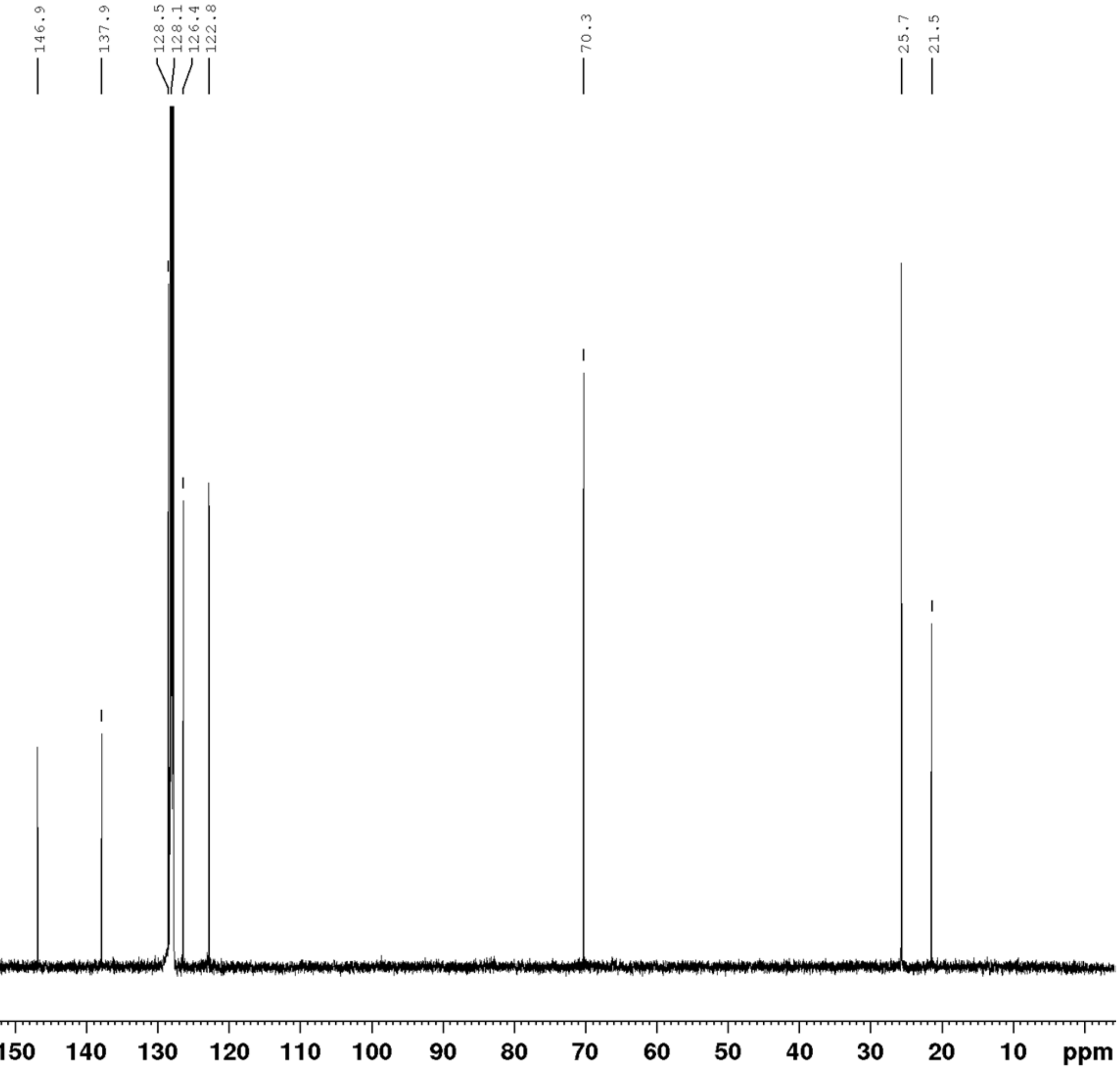

Supplementary Figure 137. ^{1}**H NMR (400 MHz,** C_6D_6**) of (*S*)-Tributyl(1-(*m*-tolyl)ethoxy)silane [(*S*)-3dh]**

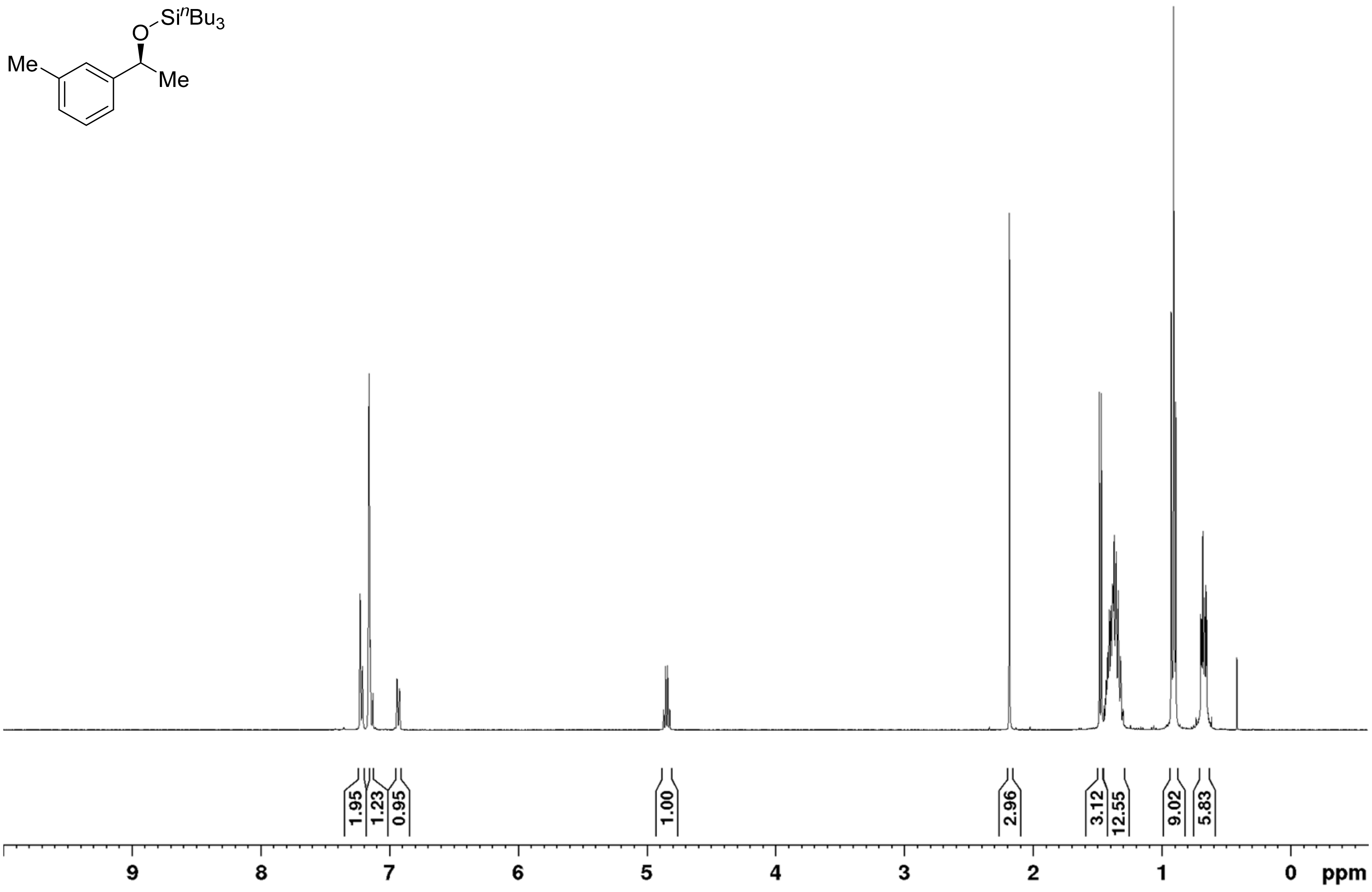

Supplementary Figure 138. **^{13}C NMR (126 MHz, C_6D_6) of (*S*)-Tributyl(1-(*m*-tolyl)ethoxy)silane [(*S*)-3dh]**

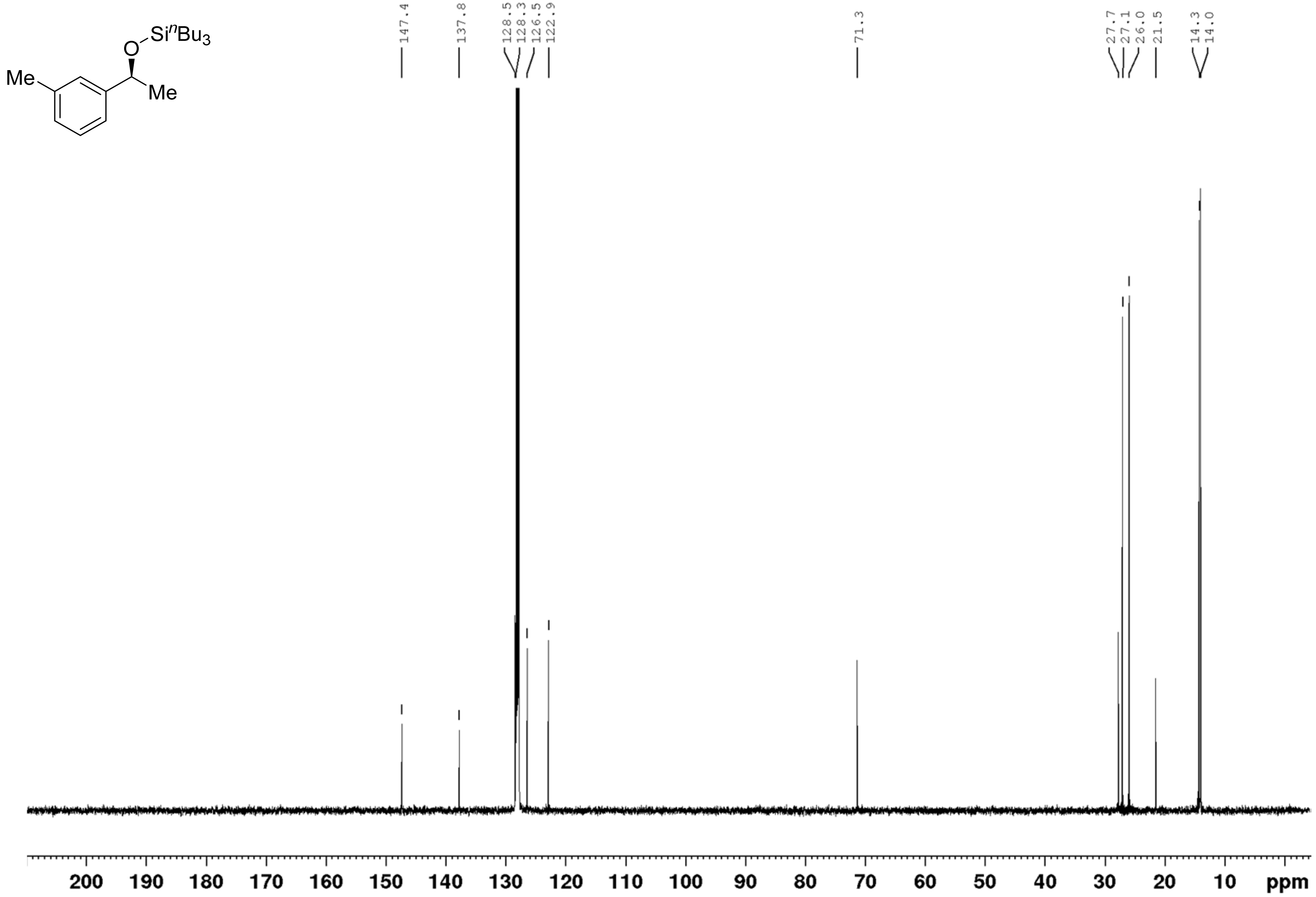

Supplementary Figure 139. ^{1}H NMR (500 MHz, $CDCl_3$) of (*R*)-1-(*o*-Tolyl)ethan-1-ol [(*R*)-1e]

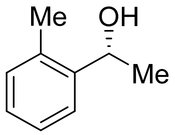

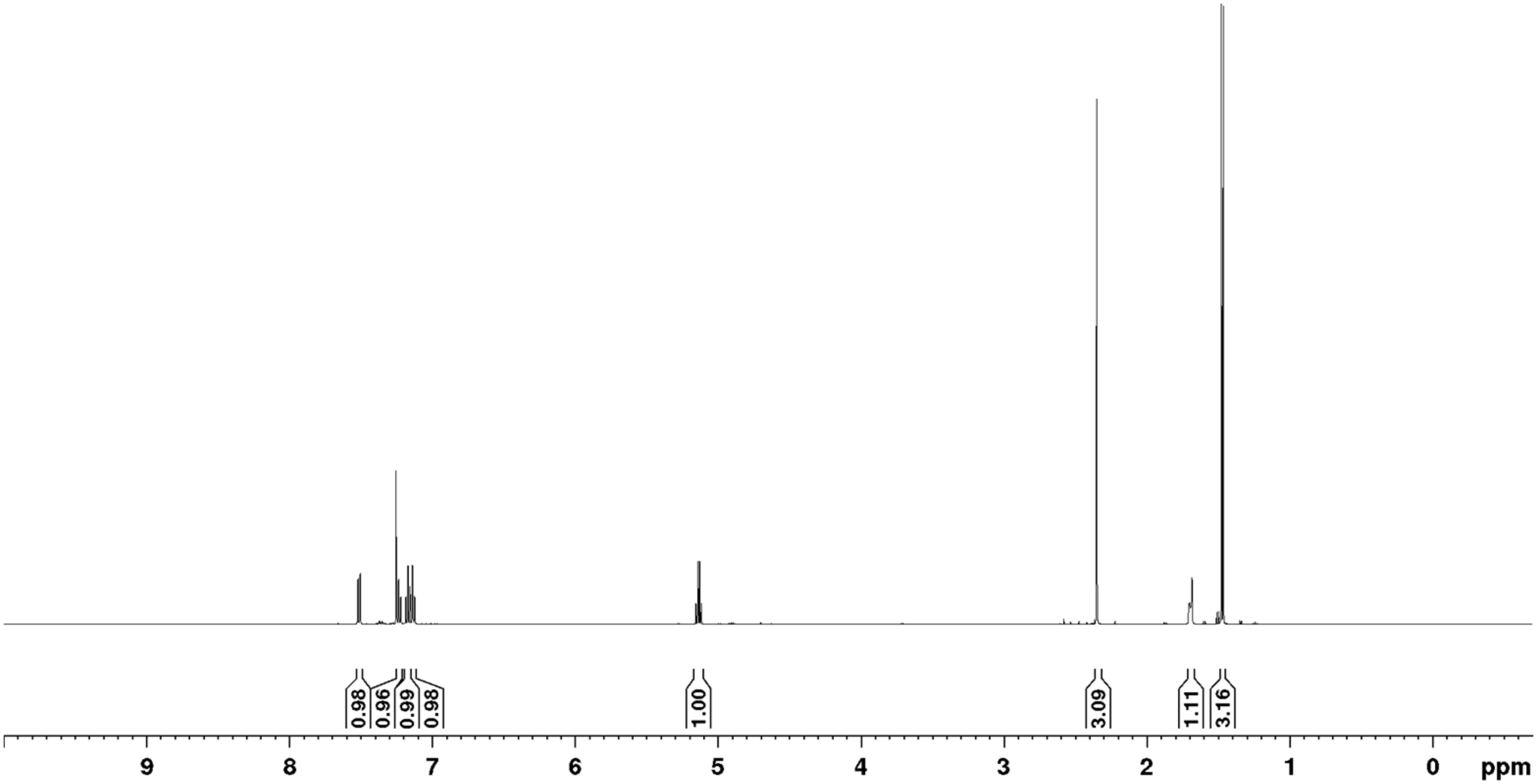

Supplementary Figure 140. ^{13}**C NMR (126 MHz, CDCl$_3$) of (*R*)-1-(*o*-Tolyl)ethan-1-ol [(*R*)-1e]**

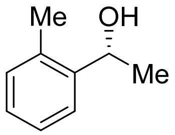

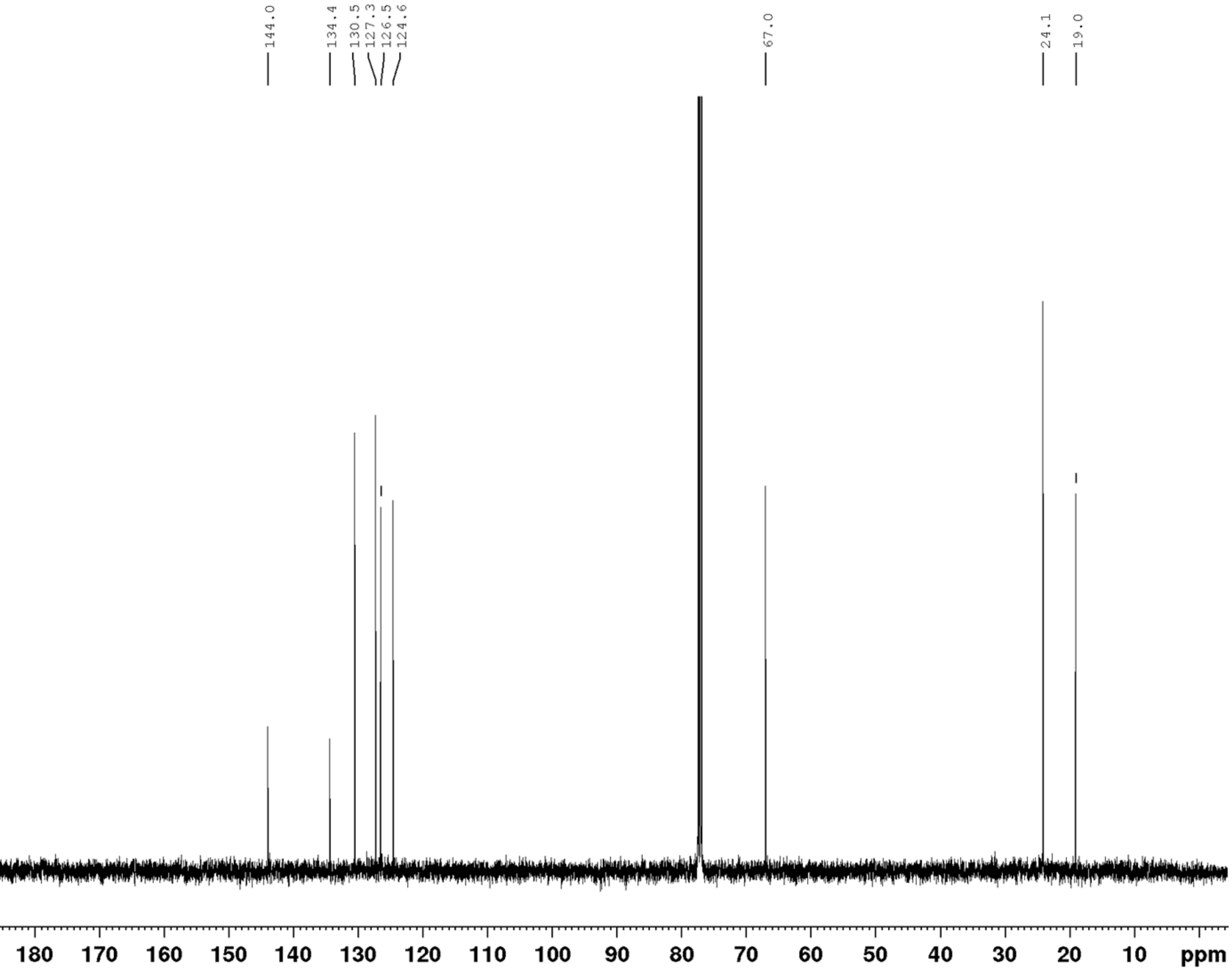

Supplementary Figure 141. **^{1}H NMR (500 MHz, C_6D_6) of (*S*)-Tributyl(1-(*o*-tolyl)ethoxy)silane [(*S*)-3eh]**

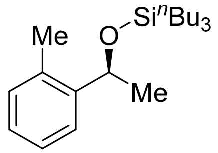

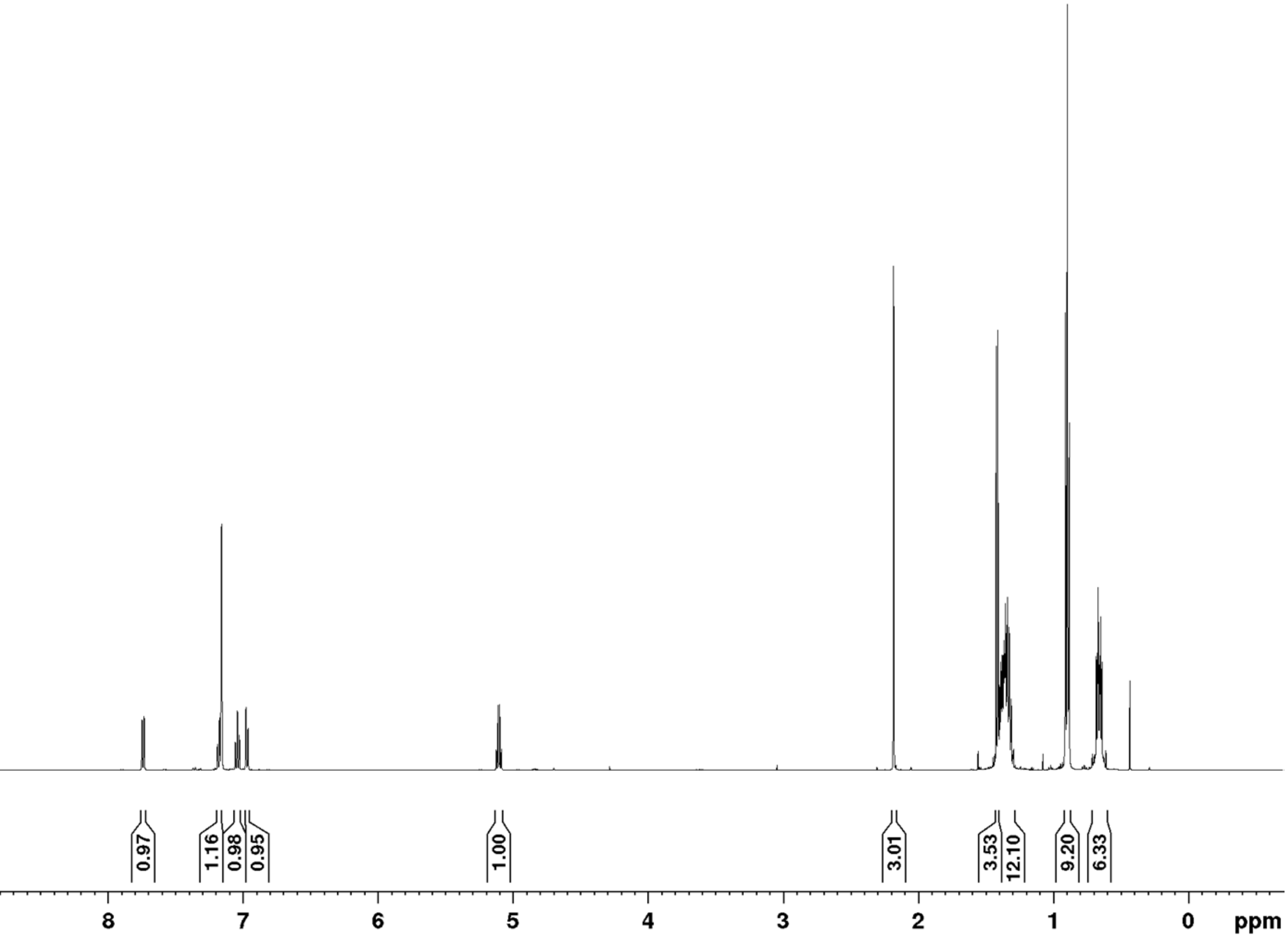

Supplementary Figure 142. ^{13}**C NMR (126 MHz, C_6D_6) of (*S*)-Tributyl(1-(*o*-tolyl)ethoxy)silane [(*S*)-3eh]**

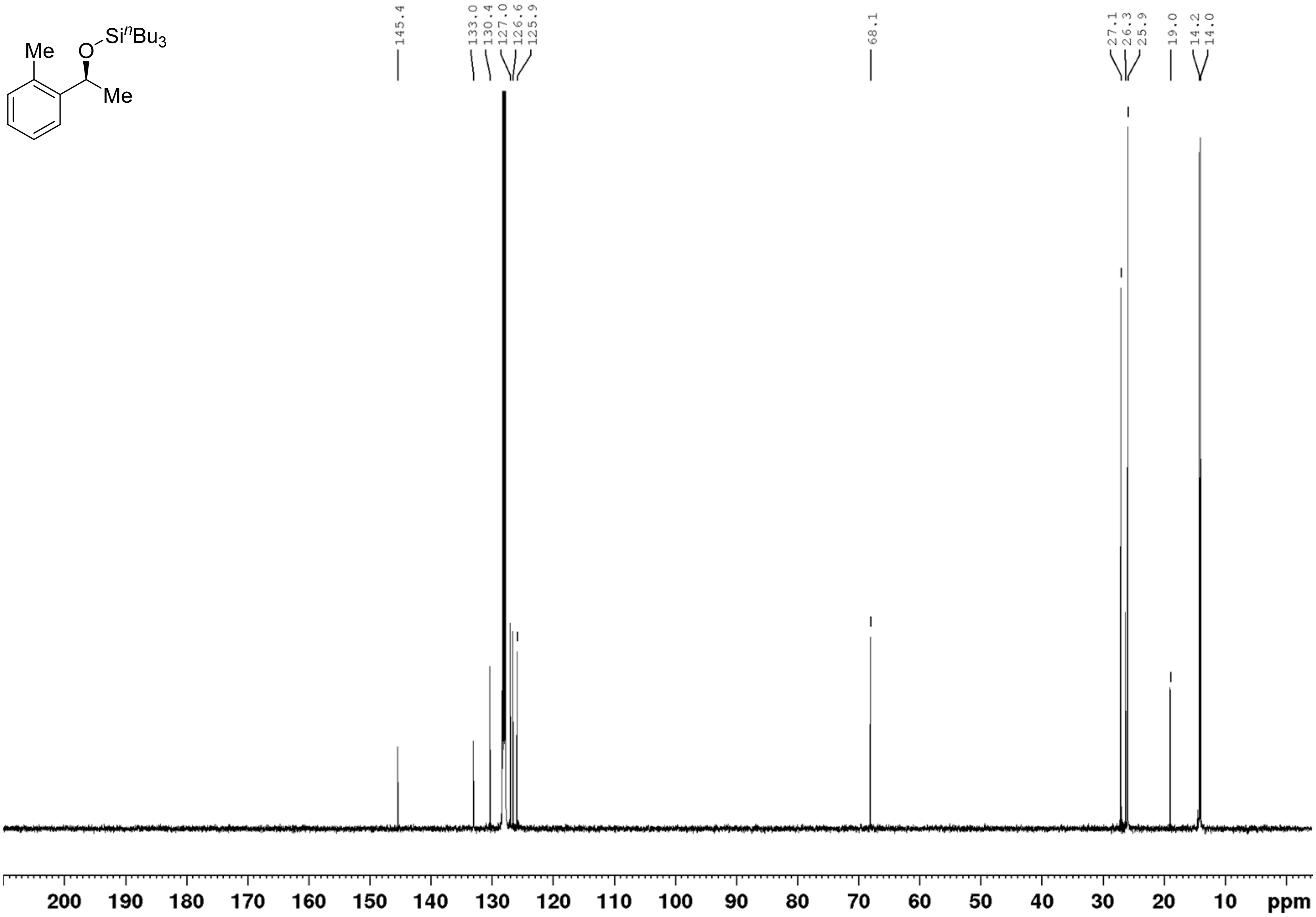

Supplementary Figure 143. ^{1}H NMR (400 MHz, C_6D_6) of (*R*)-1-(2,4-Dimethylphenyl)ethan-1-ol [(*R*)-1f]

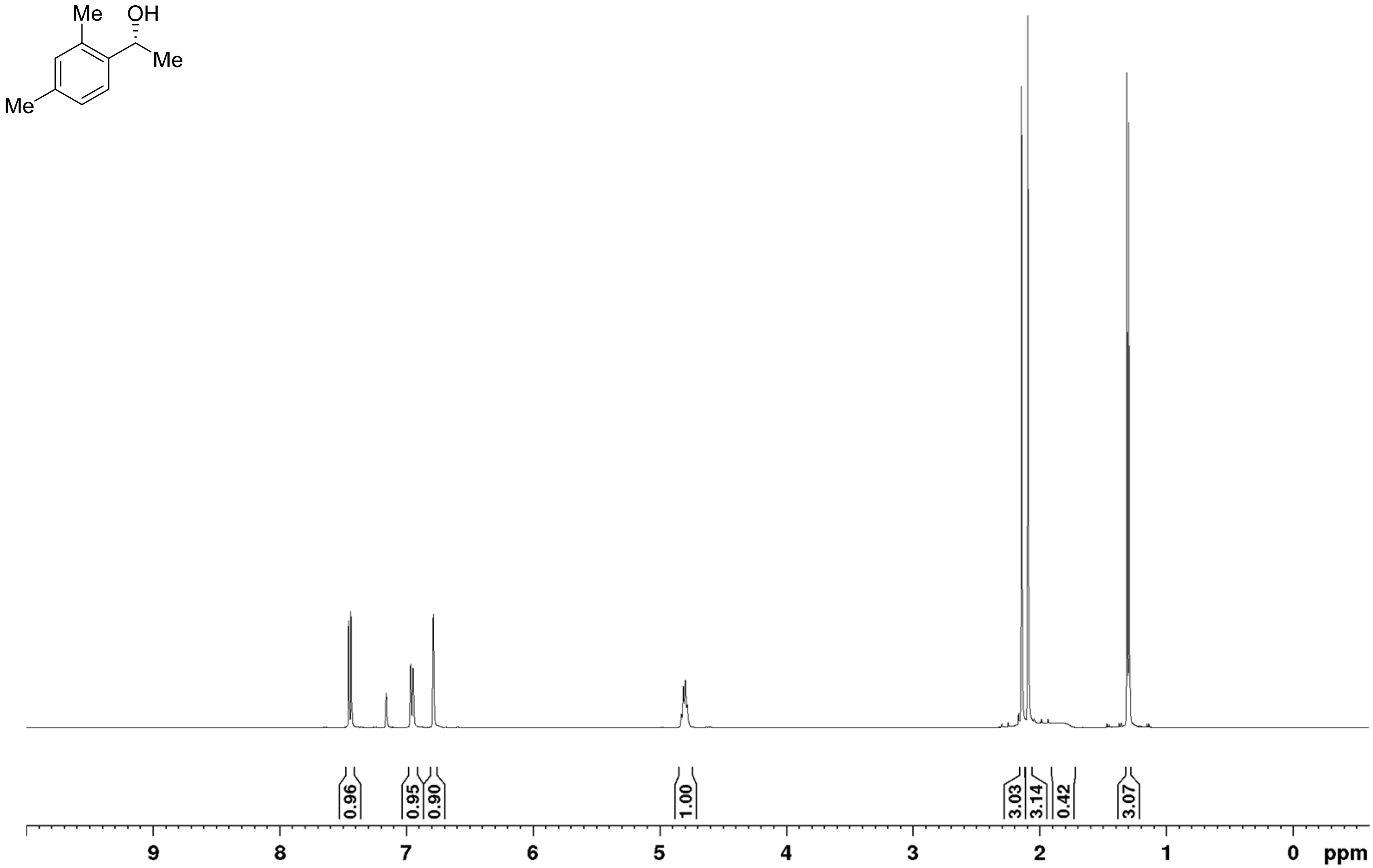

Supplementary Figure 144. ^{13}C NMR (126 MHz, C_6D_6) of (*R*)-1-(2,4-Dimethylphenyl)ethan-1-ol [(*R*)-1f]

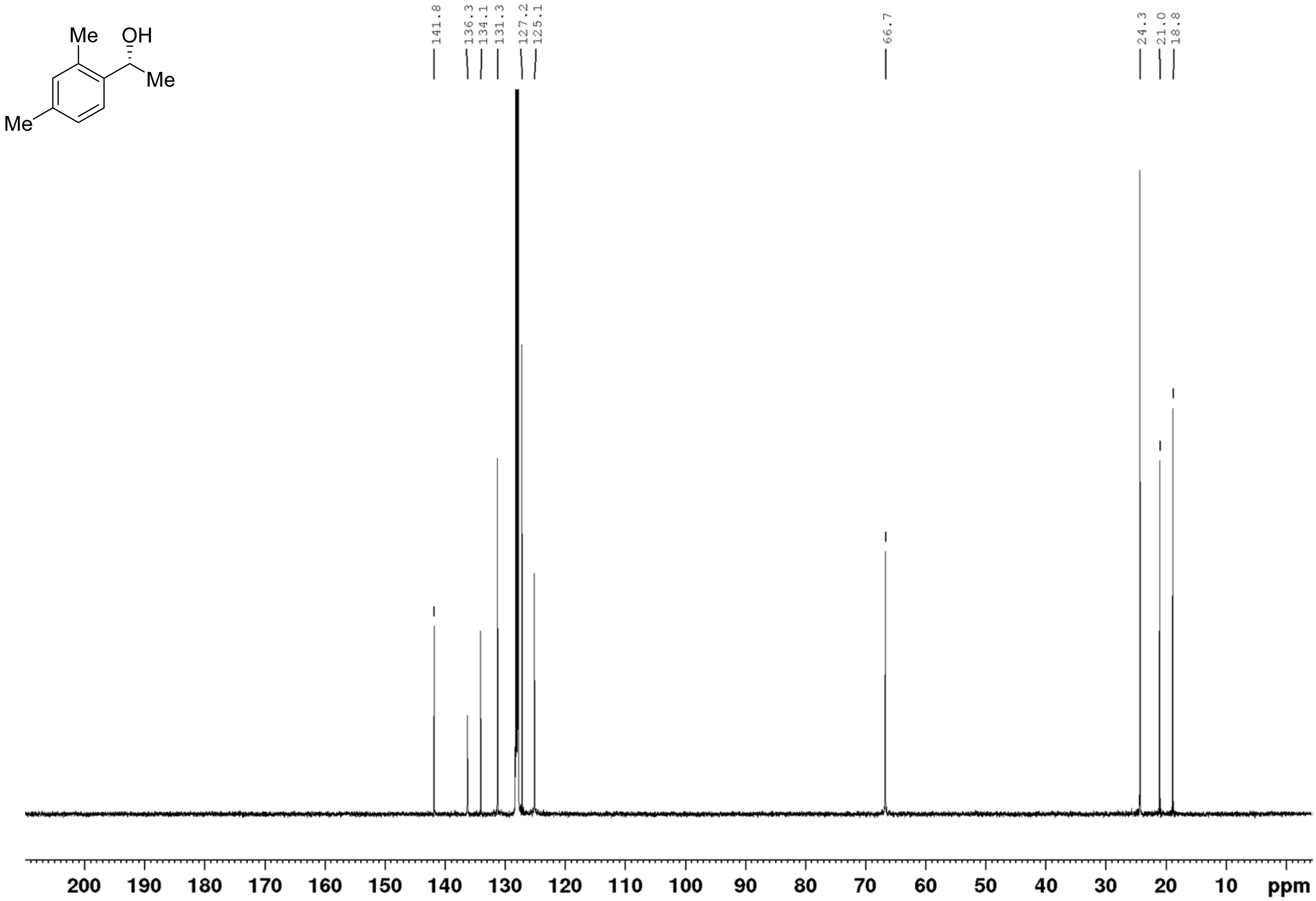

Supplementary Figure 145. ^{1}**H NMR (500 MHz, C_6D_6) of (*S*)-Tributyl(1-(2,4-dimethylphenyl)ethoxy)silane [(*S*)-3fh]**

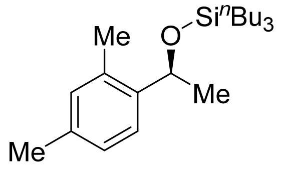

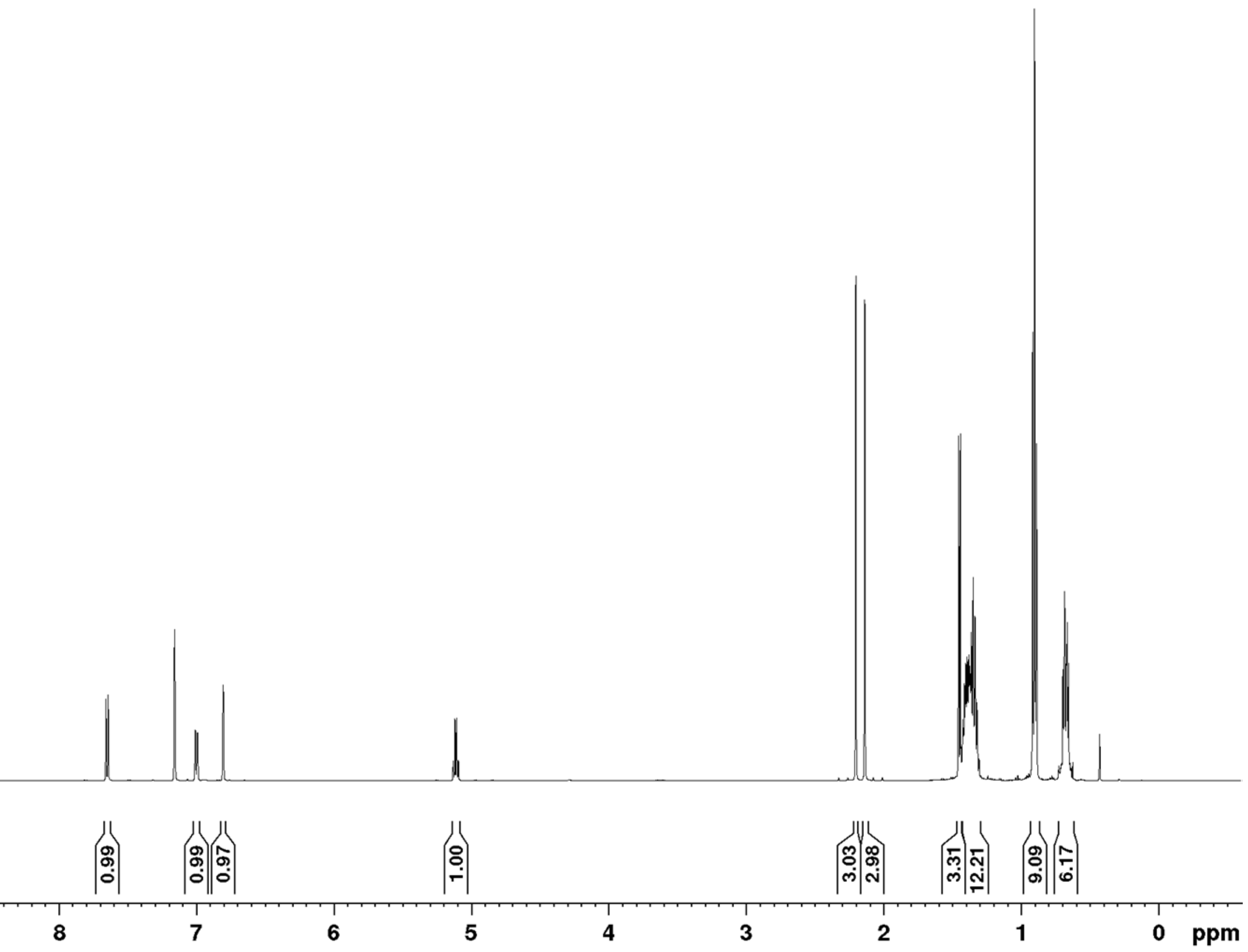

Supplementary Figure 146. ^{13}**C NMR (126 MHz, C_6D_6) of (*S*)-Tributyl(1-(2,4-dimethylphenyl)ethoxy)silane [(*S*)-3fh**

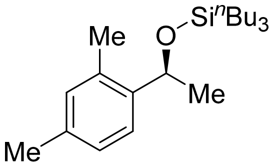

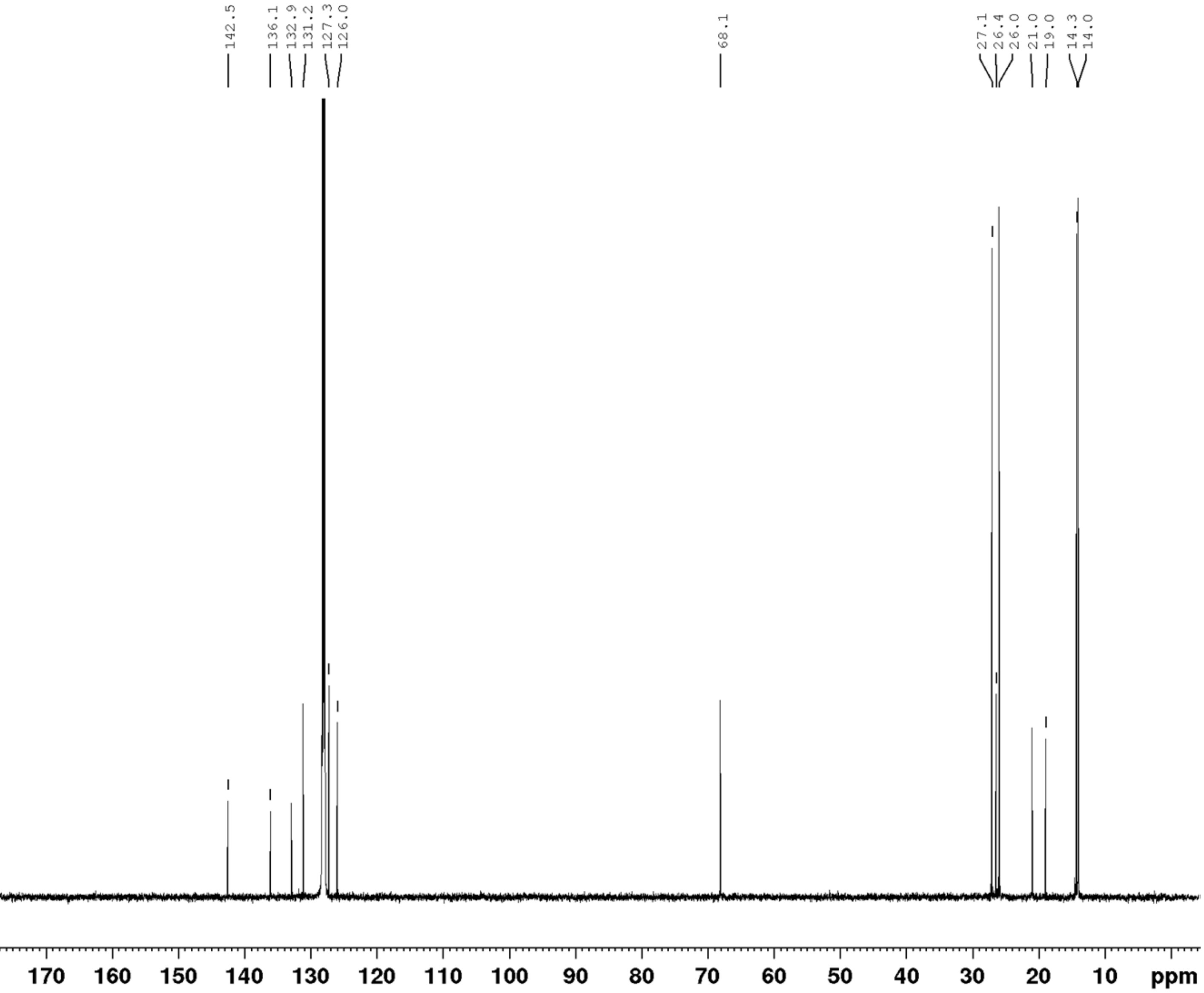

Supplementary Figure 147. **^{1}H NMR (400 MHz, C_6D_6) of (*R*)-1-(3,5-Dimethylphenyl)ethan-1-ol [(*R*)-1g]**

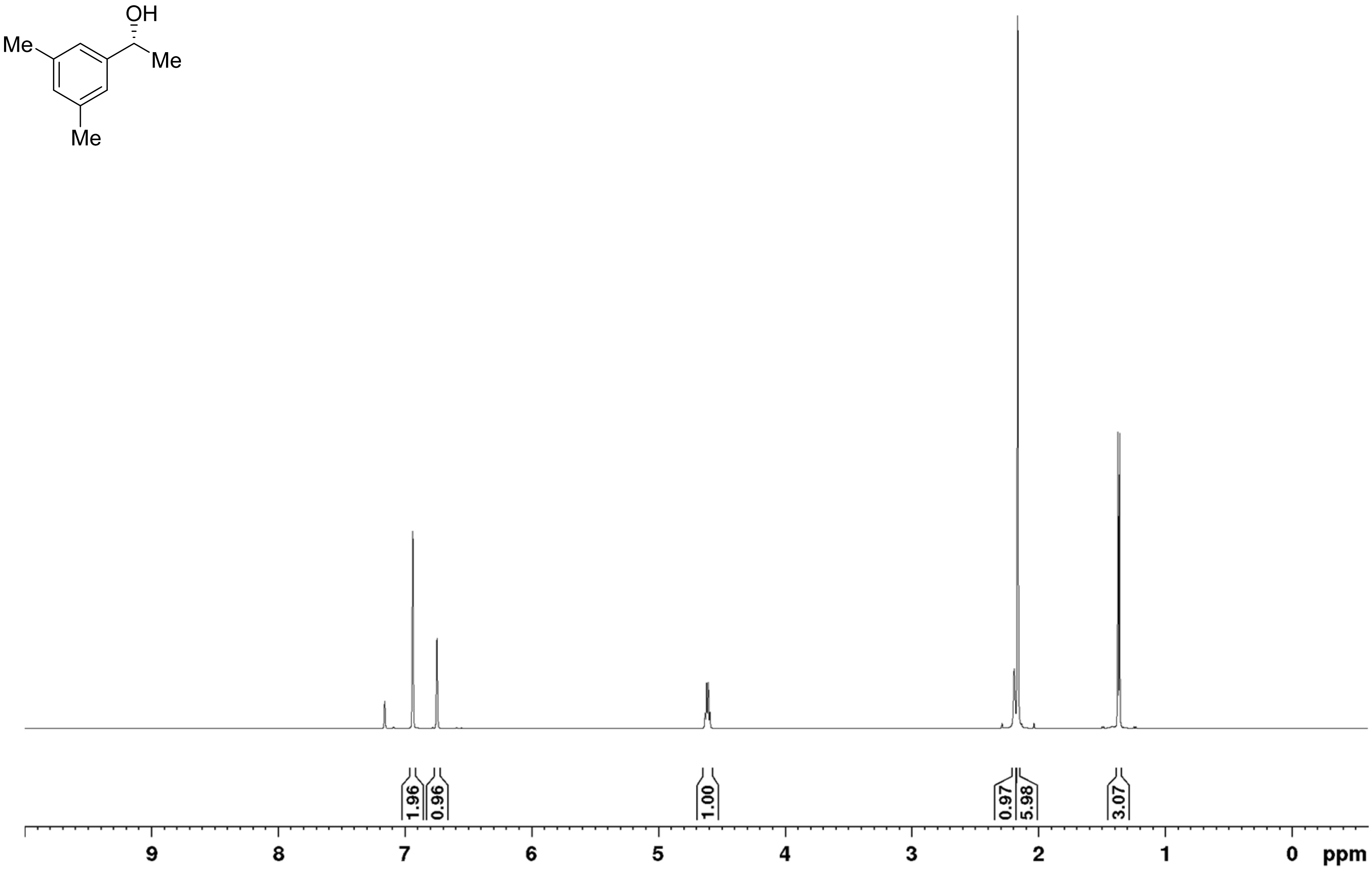

Supplementary Figure 148. ^{13}**C NMR (126 MHz, C_6D_6) of (*R*)-1-(3,5-Dimethylphenyl)ethan-1-ol [(*R*)-1g]**

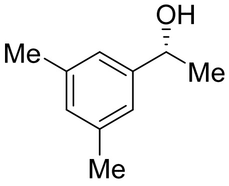

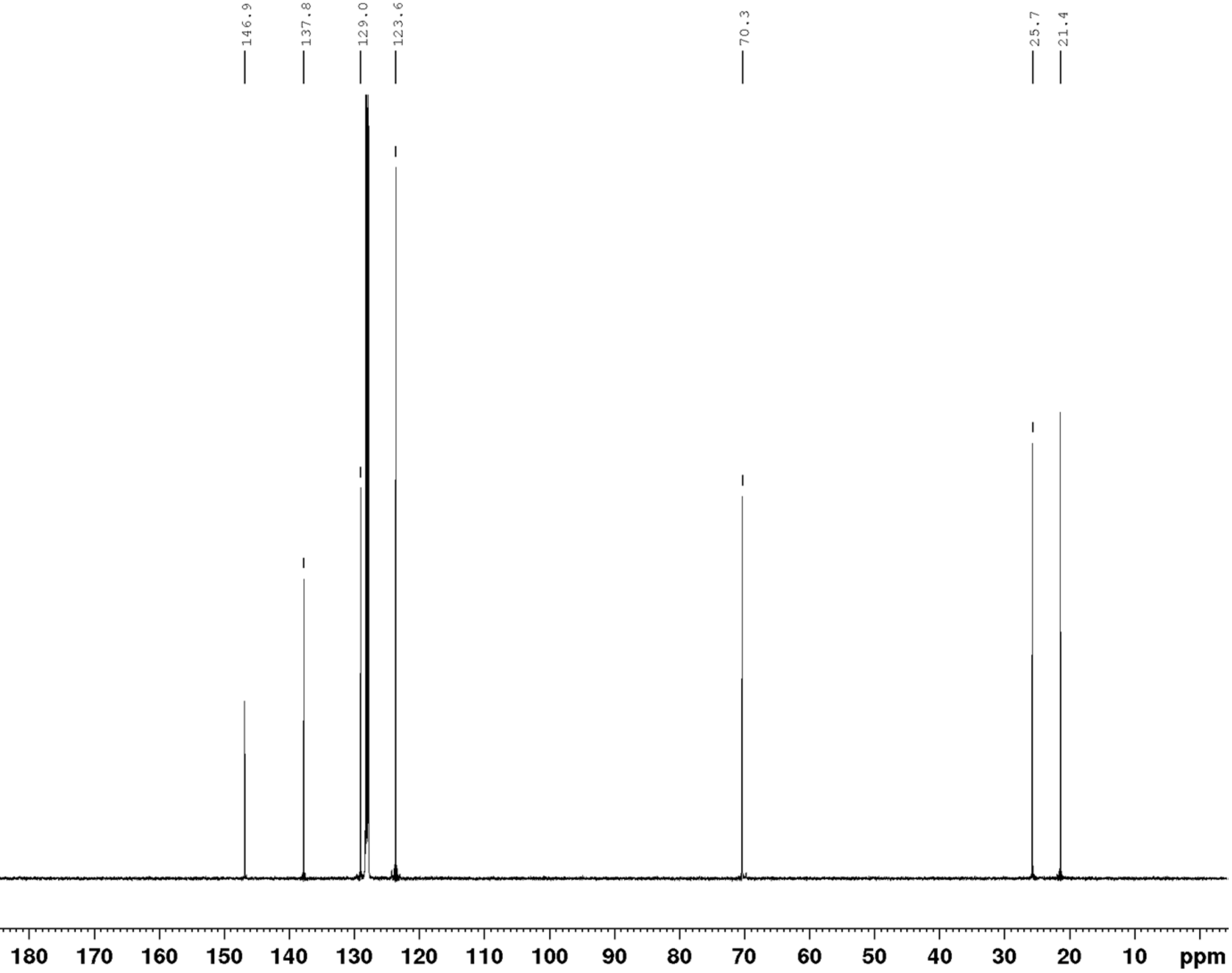

Supplementary Figure 149. ^{1}H NMR (500 MHz, C_6D_6) of (*S*)-Tributyl(1-(3,5-dimethylphenyl)ethoxy)silane [(*S*)-3gh]

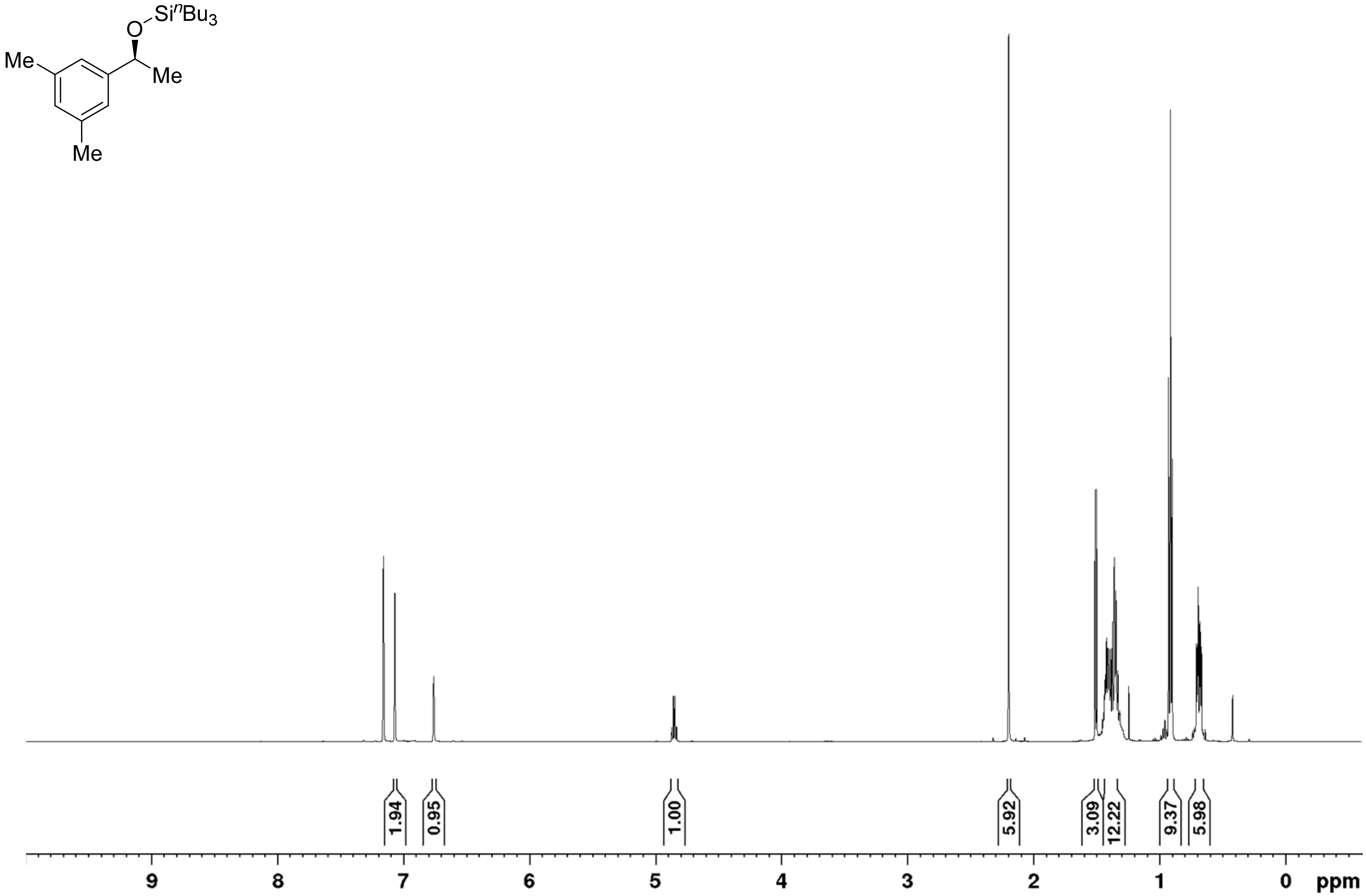

Supplementary Figure 150. ^{13}C NMR (126 MHz, C_6D_6) of (*S*)-Tributyl(1-(3,5-dimethylphenyl)ethoxy)silane [(*S*)-3gh]

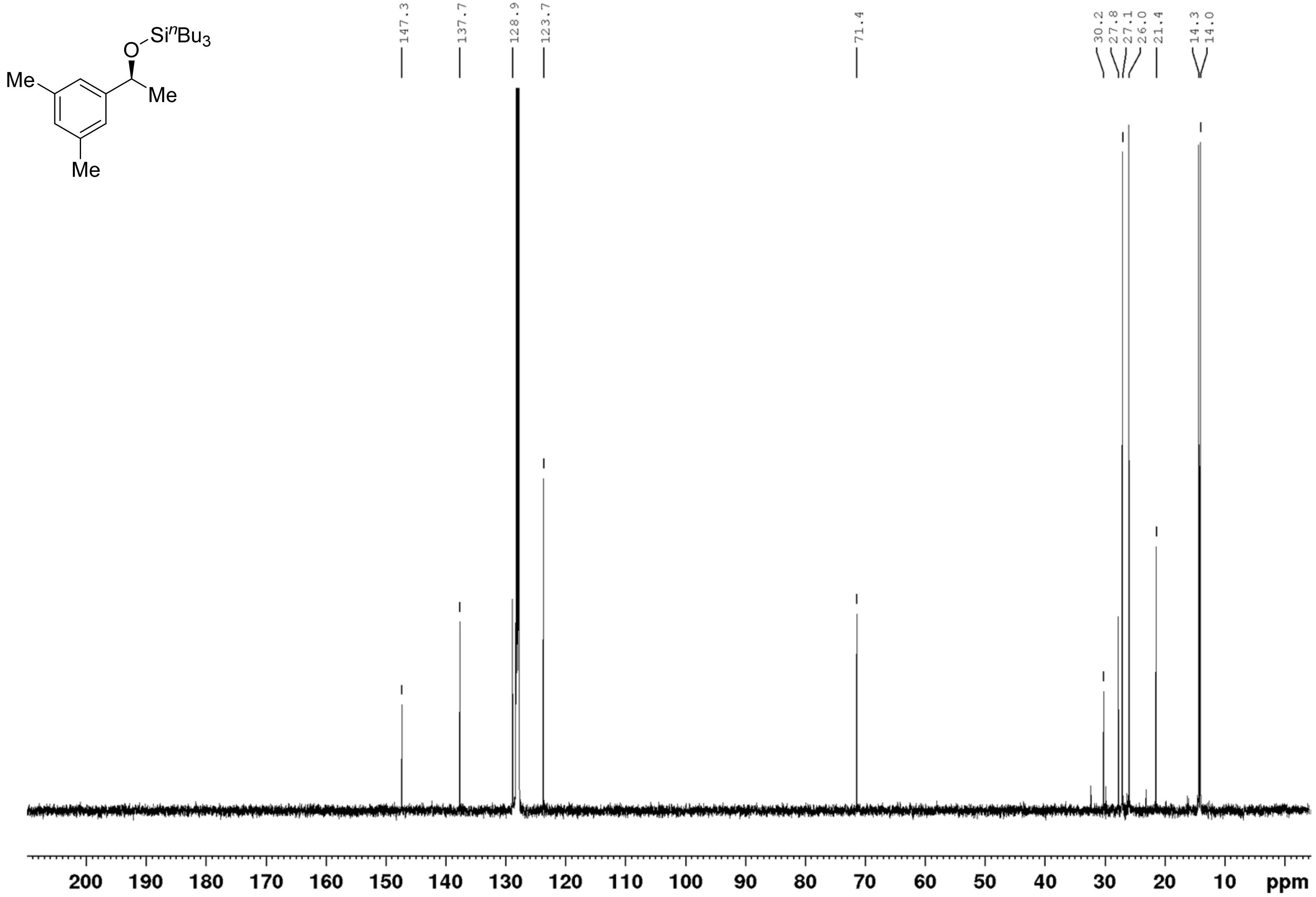

Supplementary Figure 151. **^{1}H NMR (400 MHz, C_6D_6) of (*R*)-1-(3,5-Bis(trifluoromethyl)phenyl)ethan-1-ol [(*R*)-1h]**

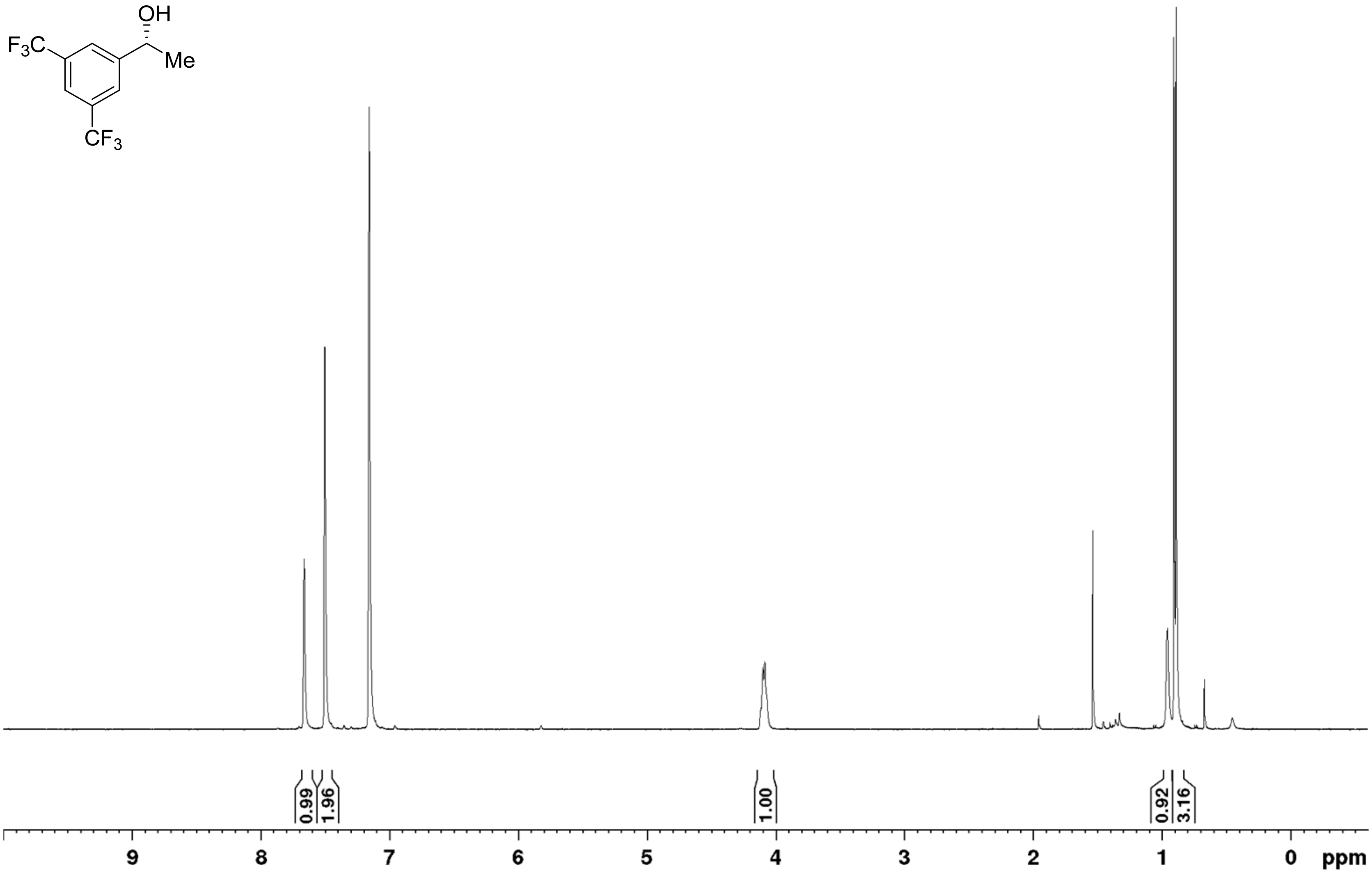

Supplementary Figure 152. ^{13}**C NMR (101 MHz,** C_6D_6**) of (*R*)-1-(3,5-Bis(trifluoromethyl)phenyl)ethan-1-ol [(*R*)-1h]**

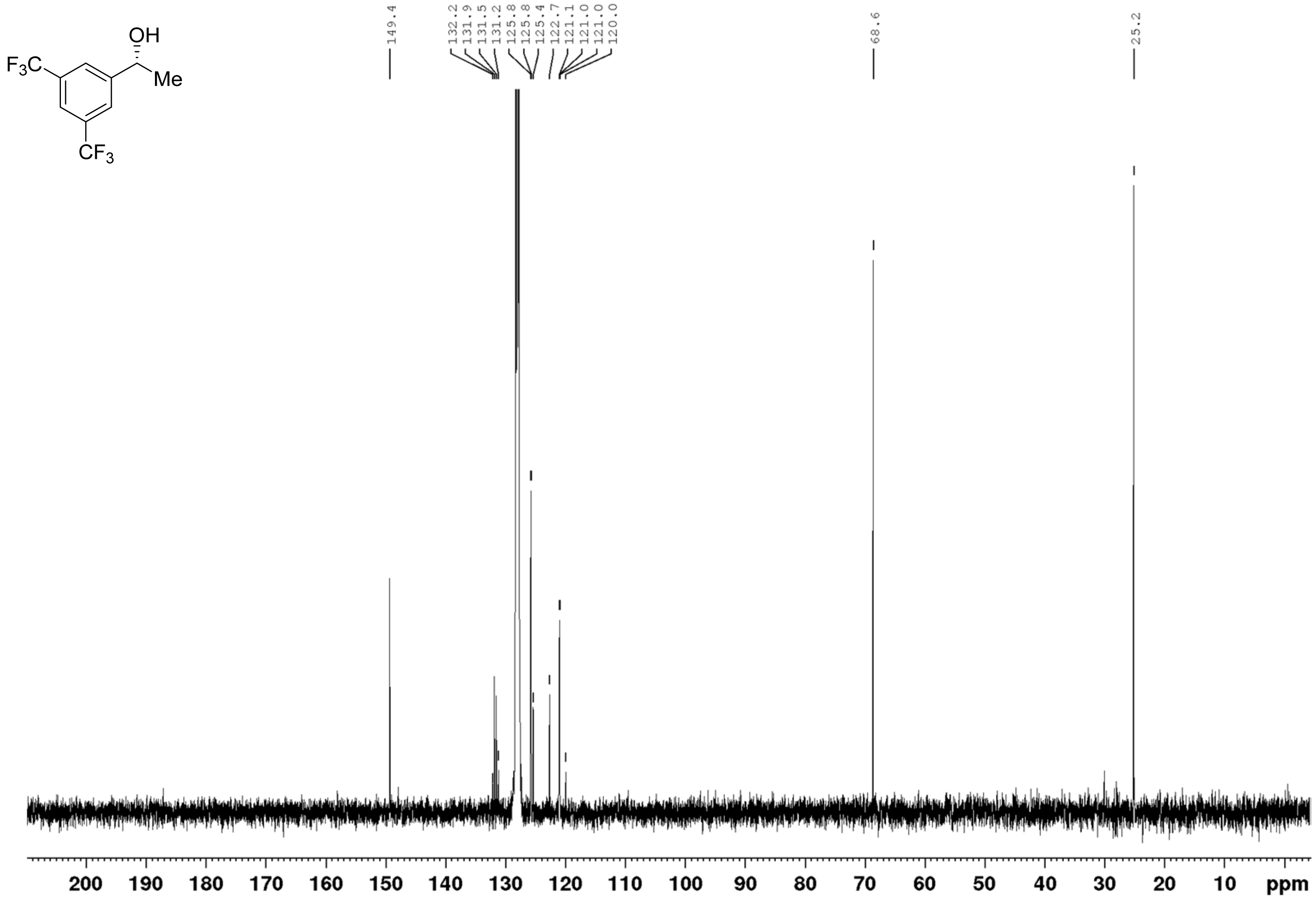

Supplementary Figure 153. **^{19}F NMR (188 MHz, C_6D_6) of (*R*)-1-(3,5-Bis(trifluoromethyl)phenyl)ethan-1-ol [(*R*)-1h]**

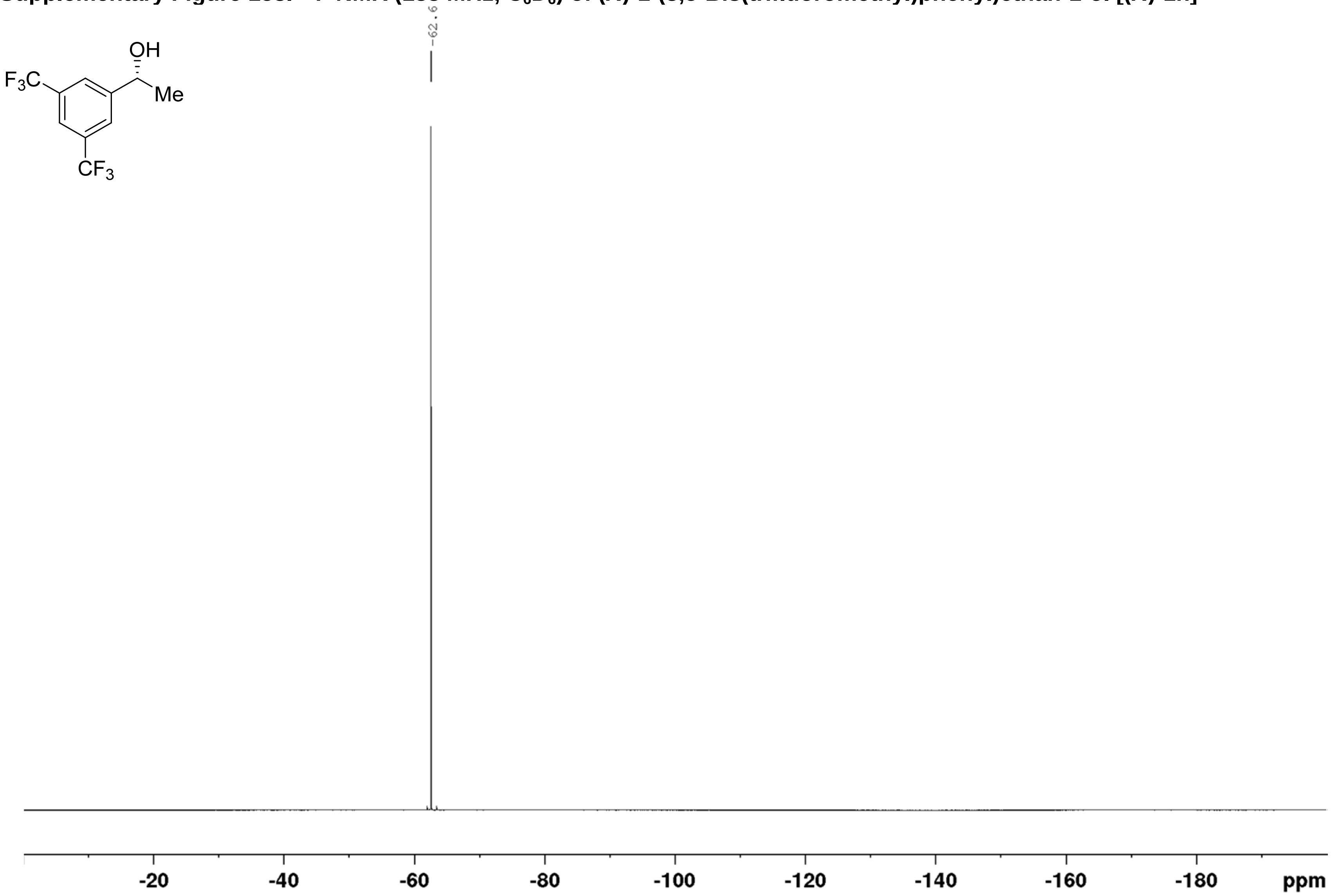

Supplementary Figure 154. **^{1}H NMR (500 MHz, C_6D_6) of (*S*)-(1-(3,5-Bis(trifluoromethyl)phenyl)ethoxy)tributylsilane [(*S*)-3hh]**

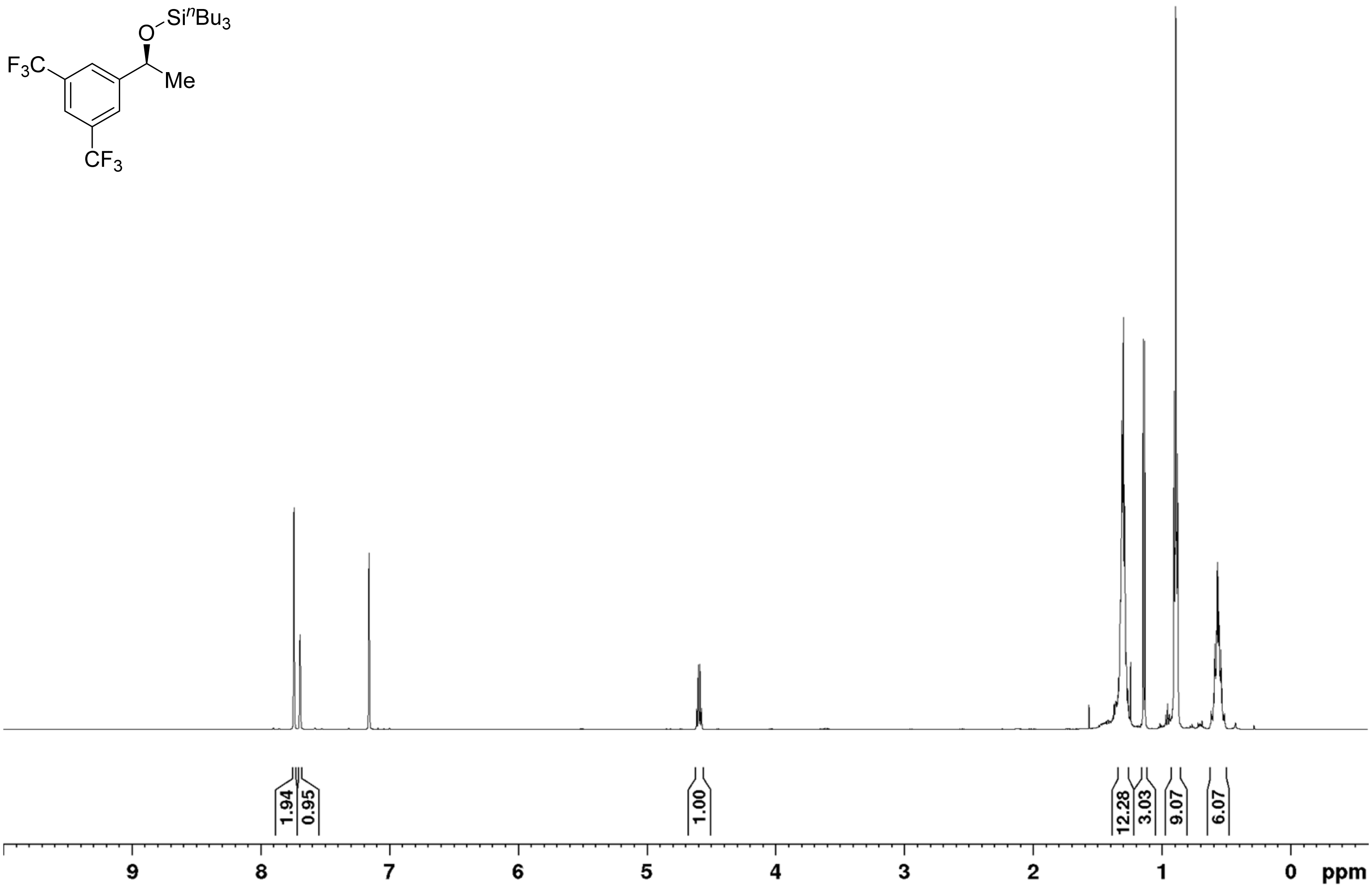

Supplementary Figure 155. **^{13}C NMR (126 MHz, C_6D_6) of (*S*)-(1-(3,5-Bis(trifluoromethyl)phenyl)ethoxy)tributylsilane [(*S*)-3hh]**

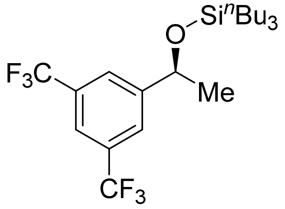

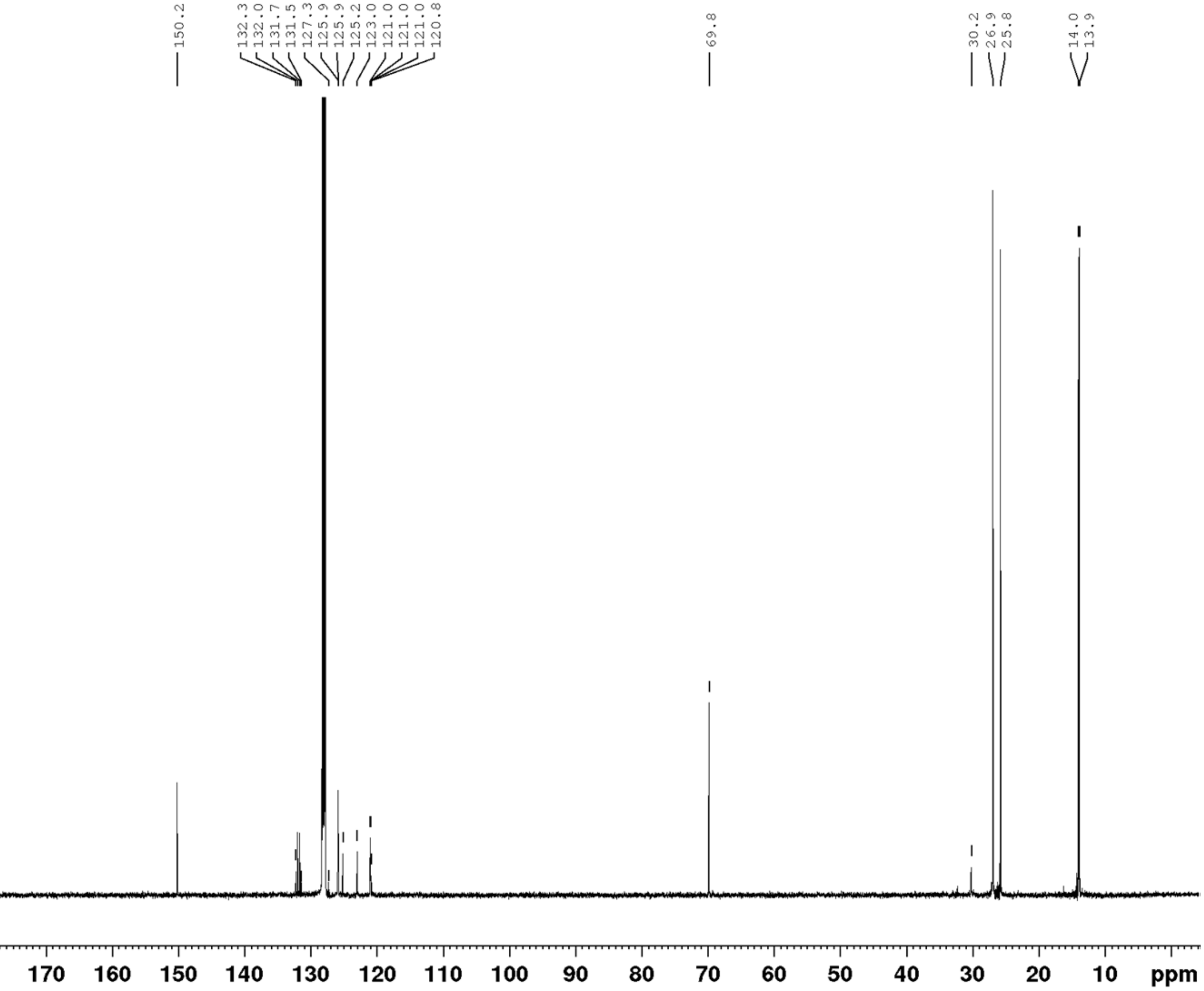

Supplementary Figure 156. ^{19}F NMR (188 MHz, C_6D_6) of (*S*)-(1-(3,5-Bis(trifluoromethyl)phenyl)ethoxy)tributylsilane [(*S*)-3hh]

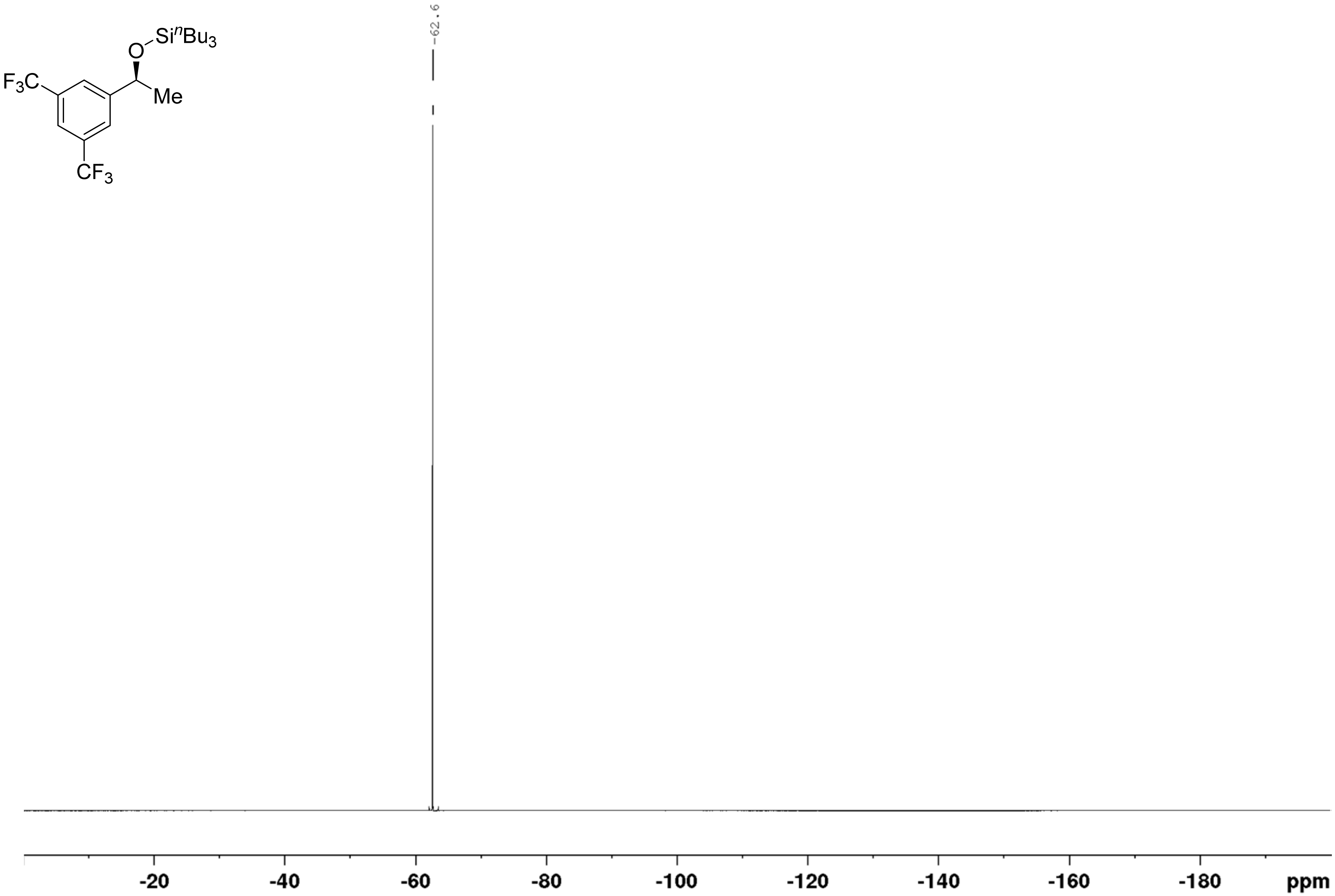

Supplementary Figure 157. ^{1}H NMR (500 MHz, $CDCl_3$) of (*R*)-1-(3,5-Dimethoxyphenyl)ethan-1-ol [(*R*)-1i]

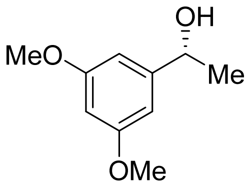

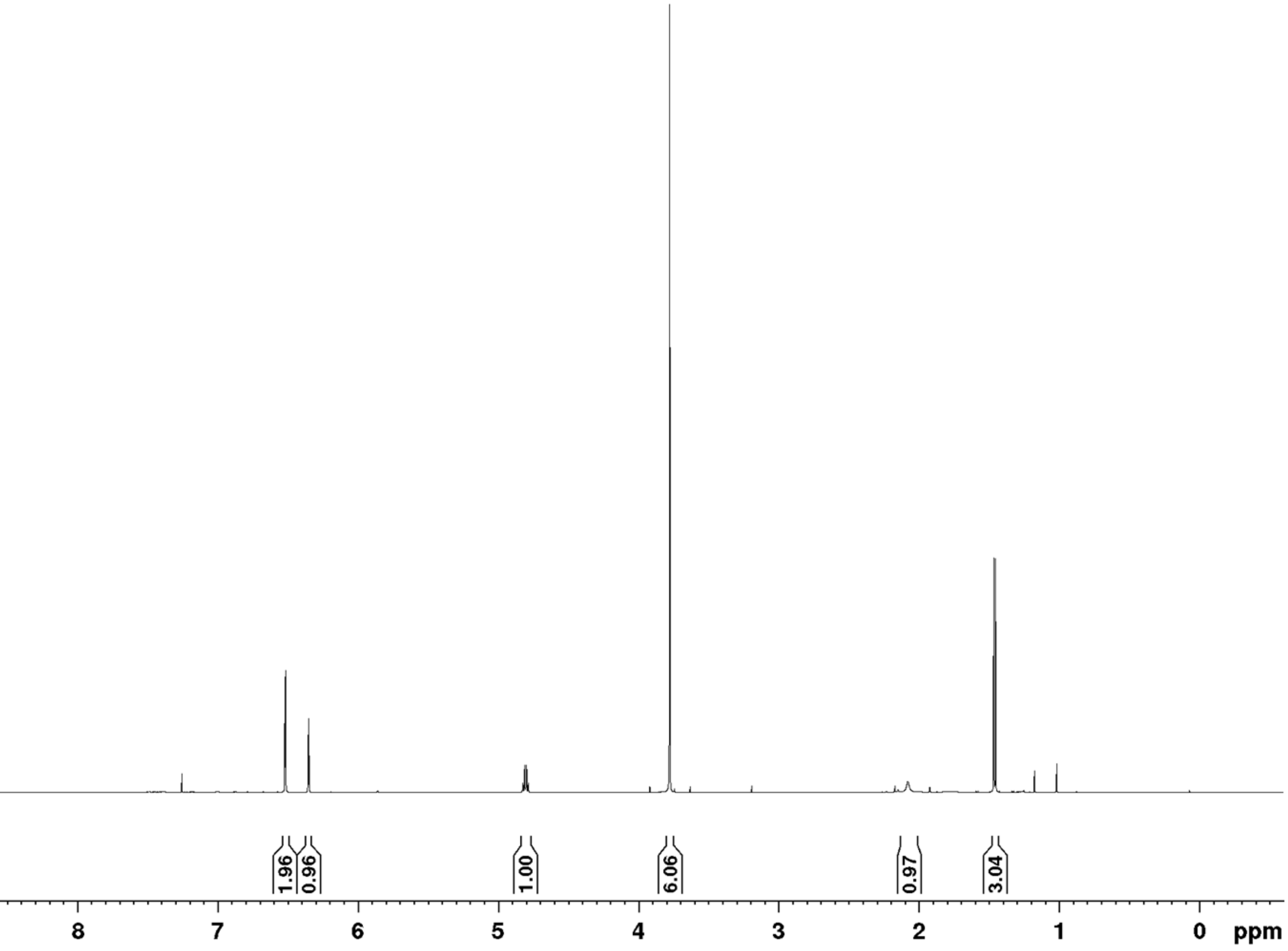

Supplementary Figure 158. ^{13}**C NMR (101 MHz, CDCl$_3$) of (*R*)-1-(3,5-Dimethoxyphenyl)ethan-1-ol [(*R*)-1i]**

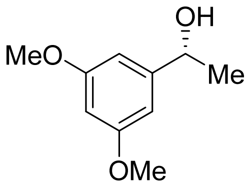

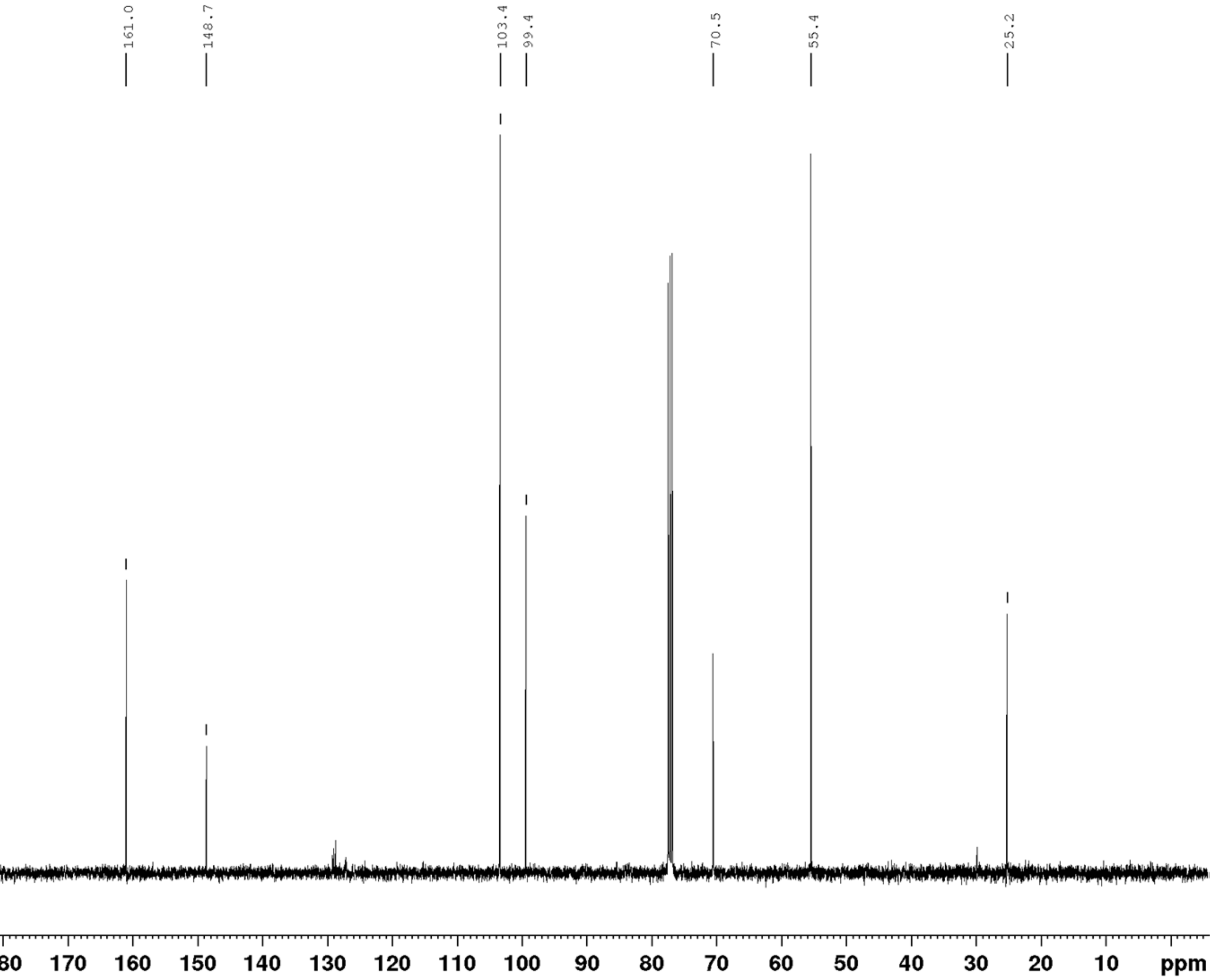

Supplementary Figure 159. **^{1}H NMR (500 MHz, C_6D_6) of (*S*)-Tributyl(1-(3,5-dimethoxyphenyl)ethoxy)silane [(*S*)-3ih]**

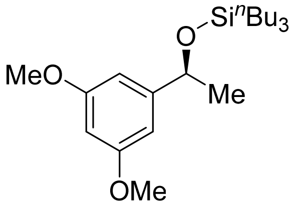

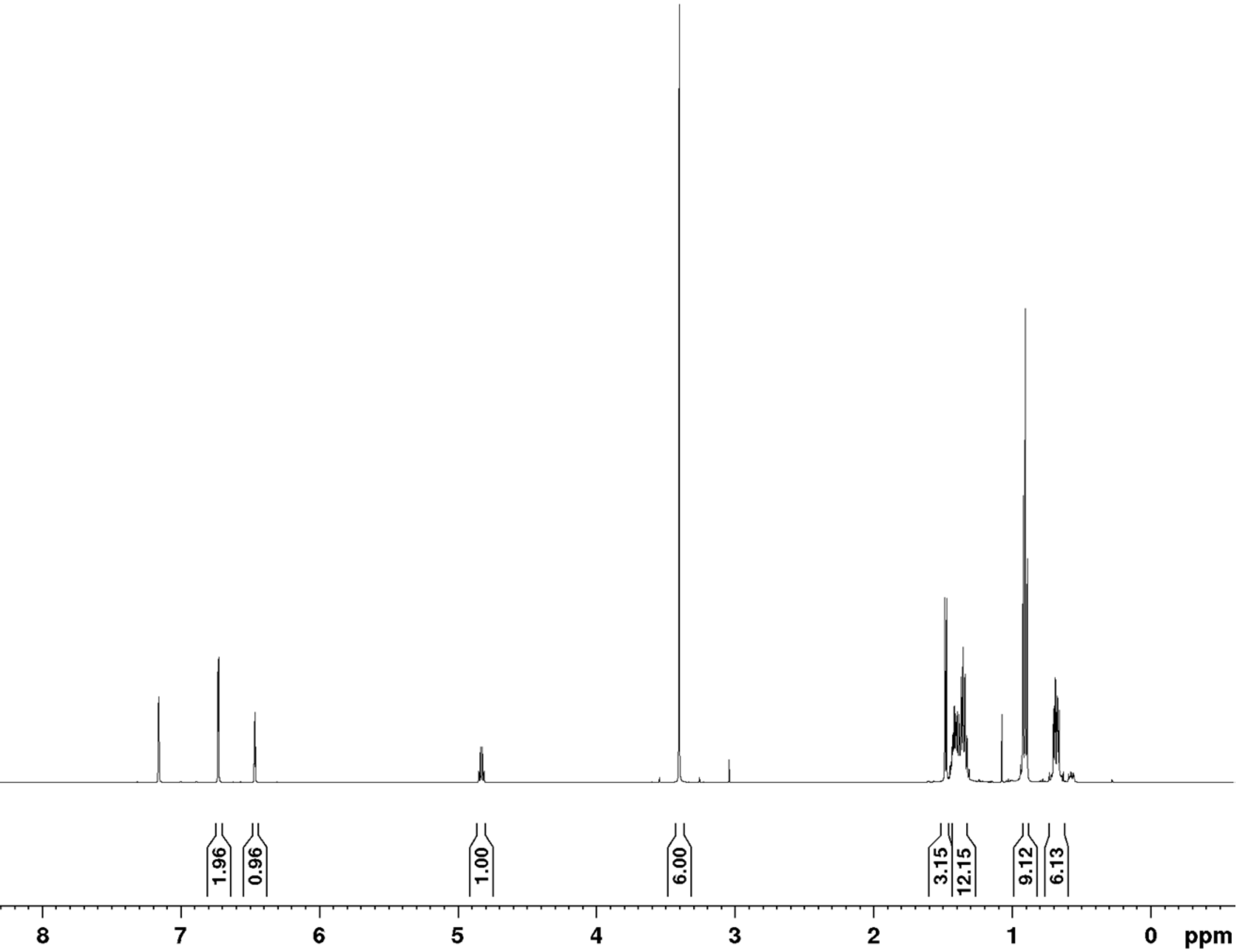

Supplementary Figure 160. ^{13}C NMR (126 MHz, C_6D_6) of (*S*)-Tributyl(1-(3,5-dimethoxyphenyl)ethoxy)silane [(*S*)-3ih]

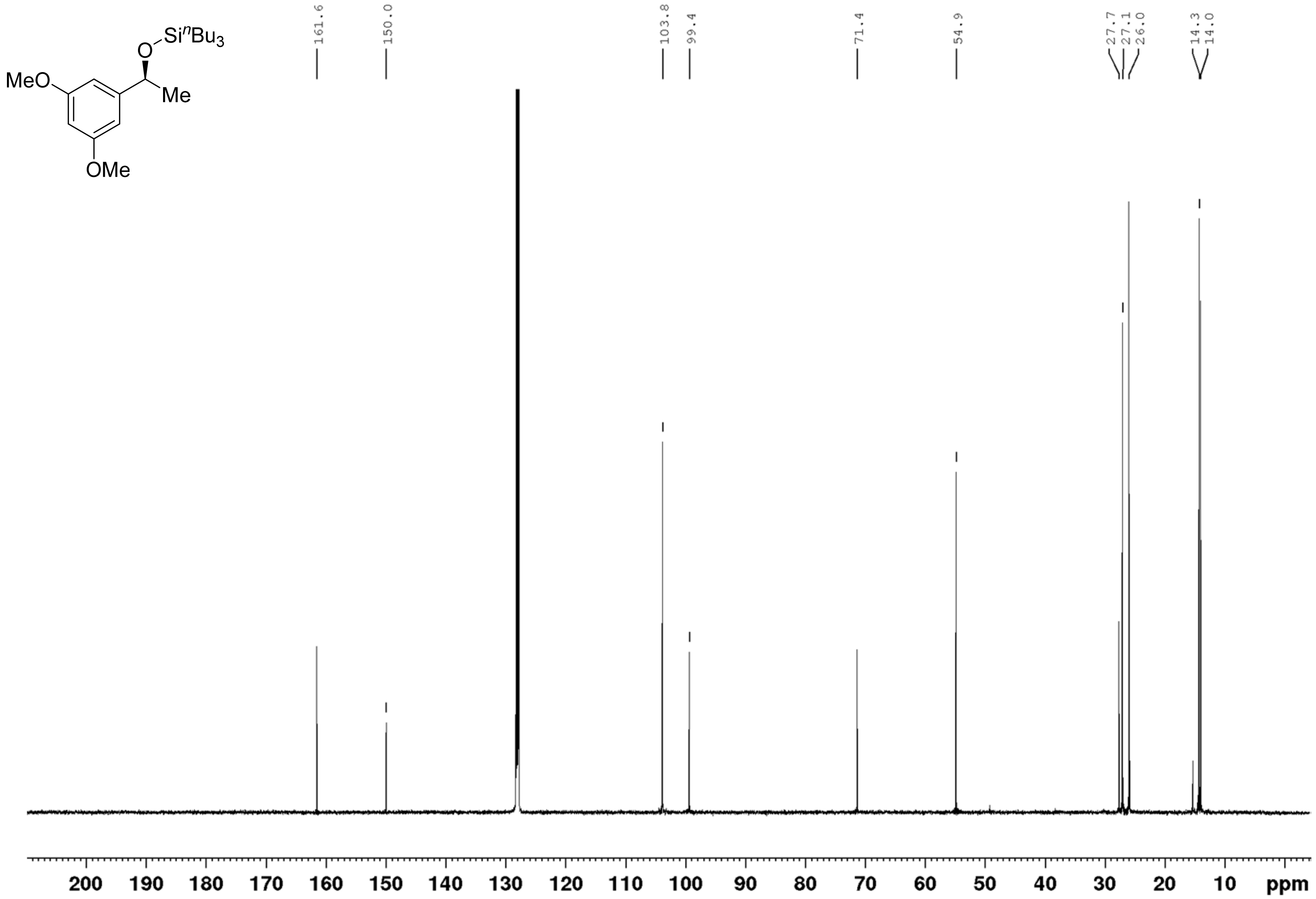

Supplementary Figure 161. ^{1}**H NMR (500 MHz,** C_6D_6**) of (*R*)-1-(2,6-Dimethylphenyl)ethan-1-ol [(*R*)-1j]**

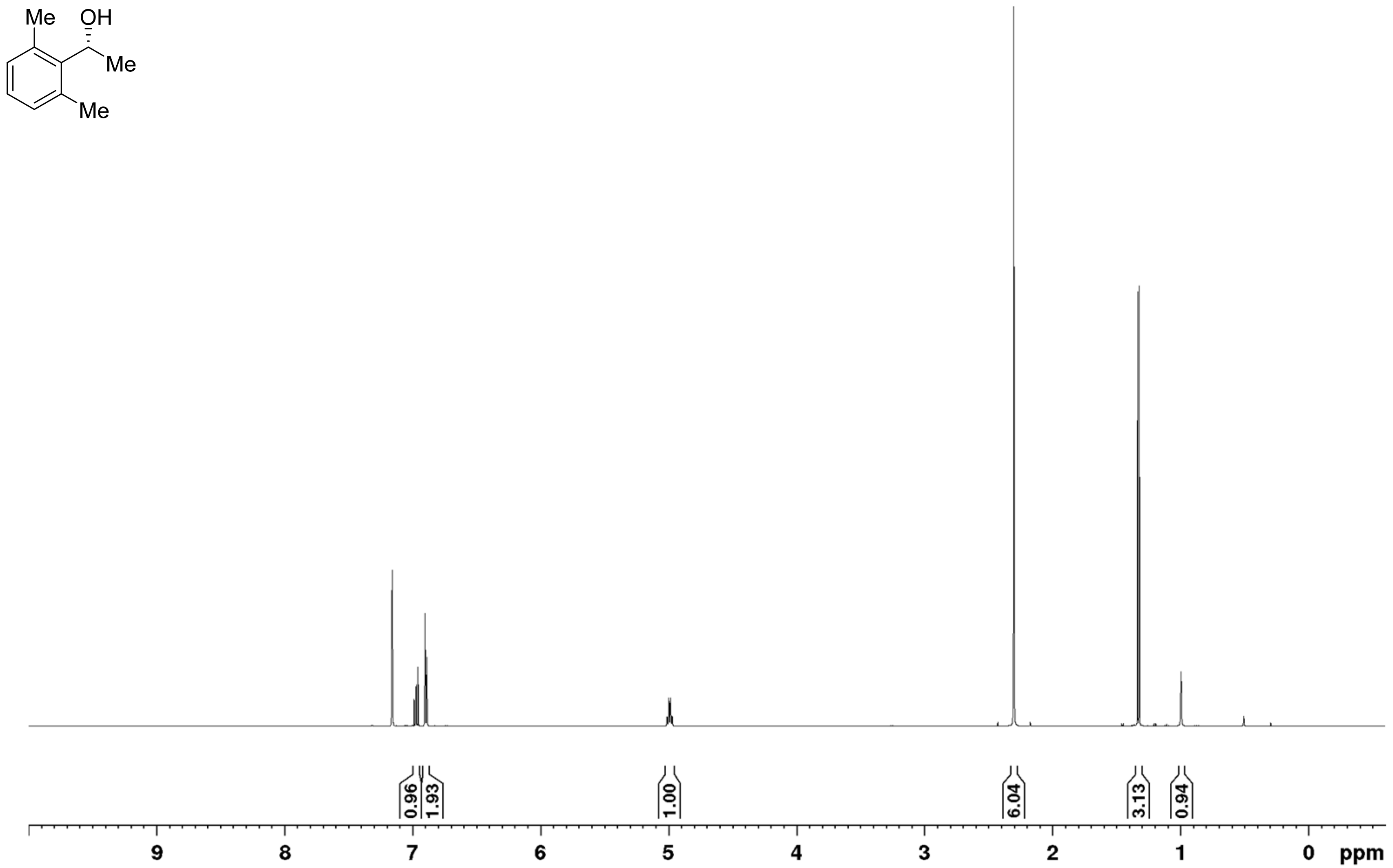

Supplementary Figure 162. ^{13}**C NMR (126 MHz, C_6D_6) of (*R*)-1-(2,6-Dimethylphenyl)ethan-1-ol [(*R*)-1j]**

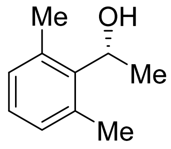

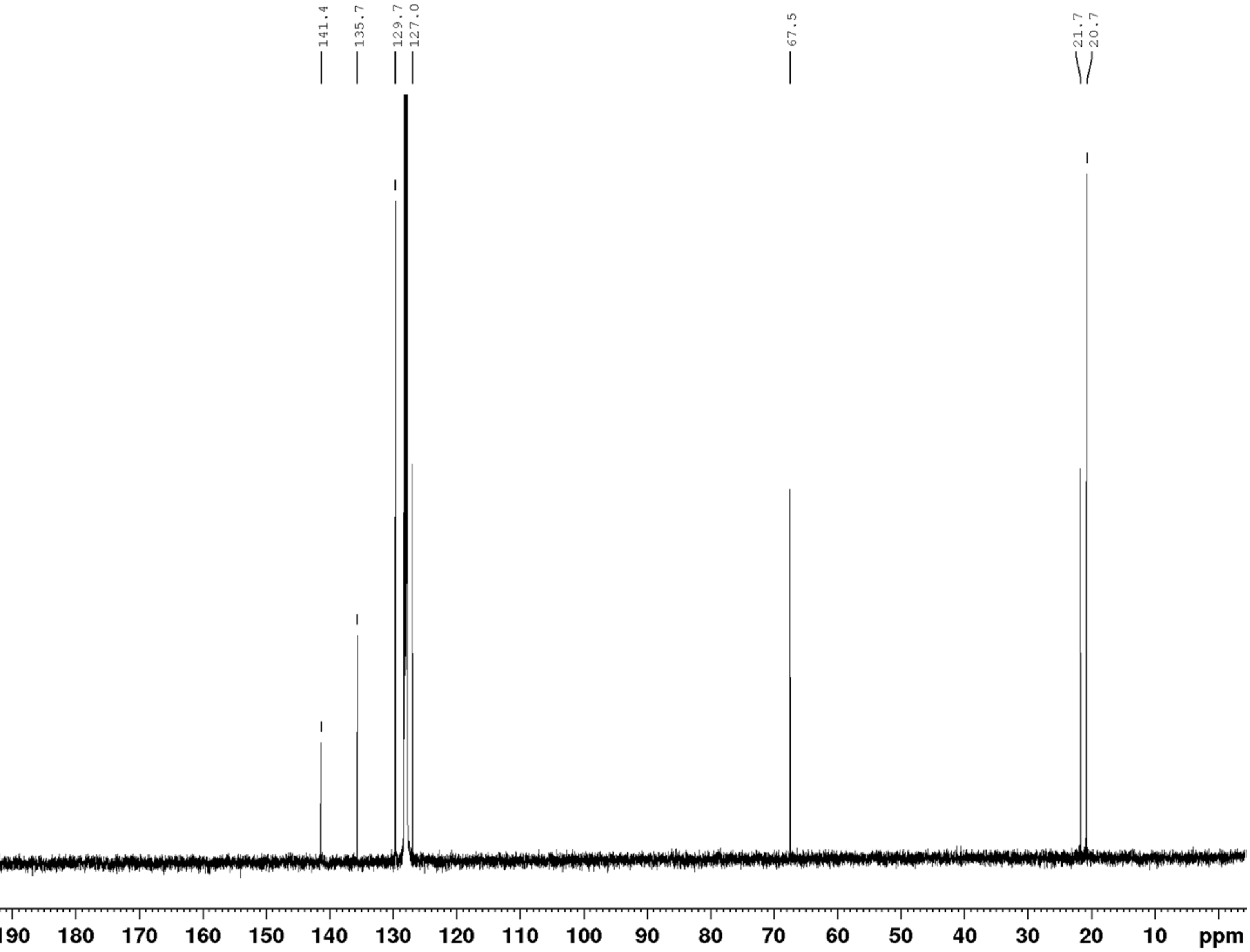

Supplementary Figure 163. ^{1}H NMR (500 MHz, C_6D_6) of (*S*)-Tributyl(1-(2,6-dimethylphenyl)ethoxy)silane [(*S*)-3jh]

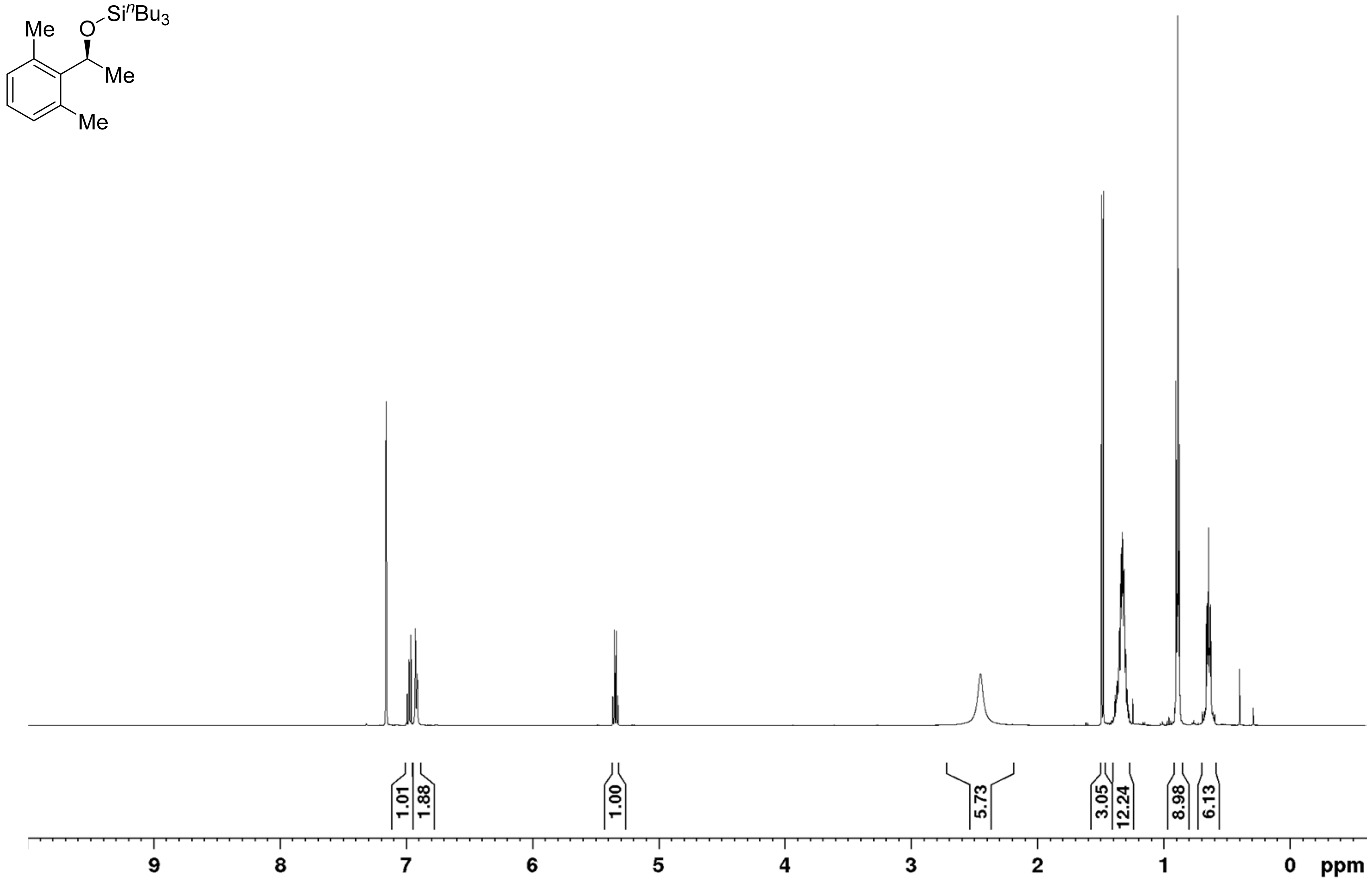

Supplementary Figure 164. ^{13}**C NMR (126 MHz, C_6D_6) of (*S*)-Tributyl(1-(2,6-dimethylphenyl)ethoxy)silane [(*S*)-3jh]**

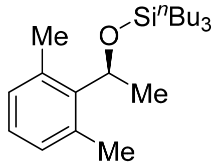

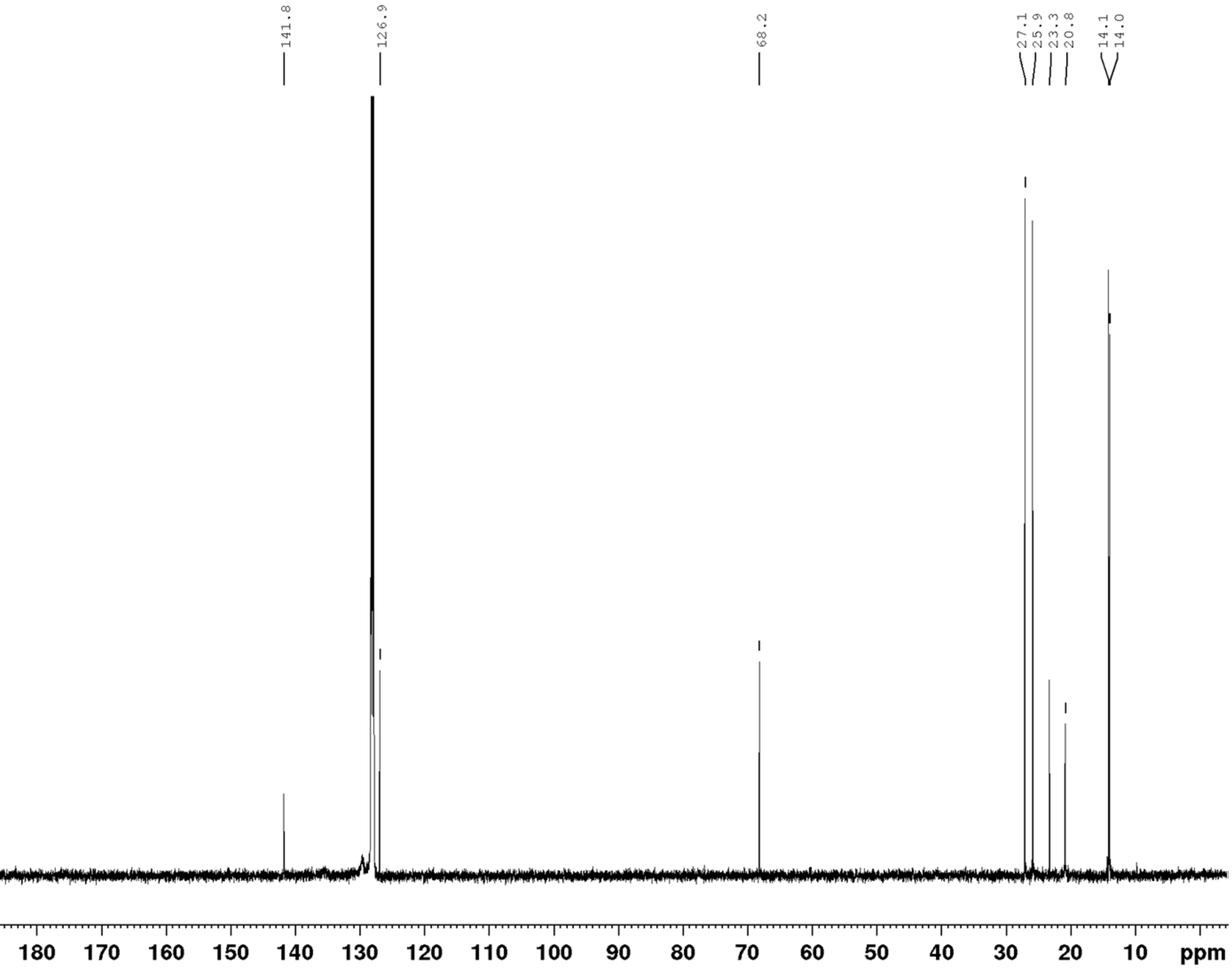

Supplementary Figure 165. **^{1}H NMR (500 MHz, $CDCl_3$) of (*R*)-1-Mesitylethan-1-ol [(*R*)-1k]**

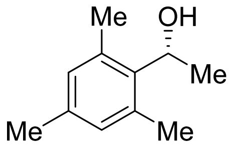

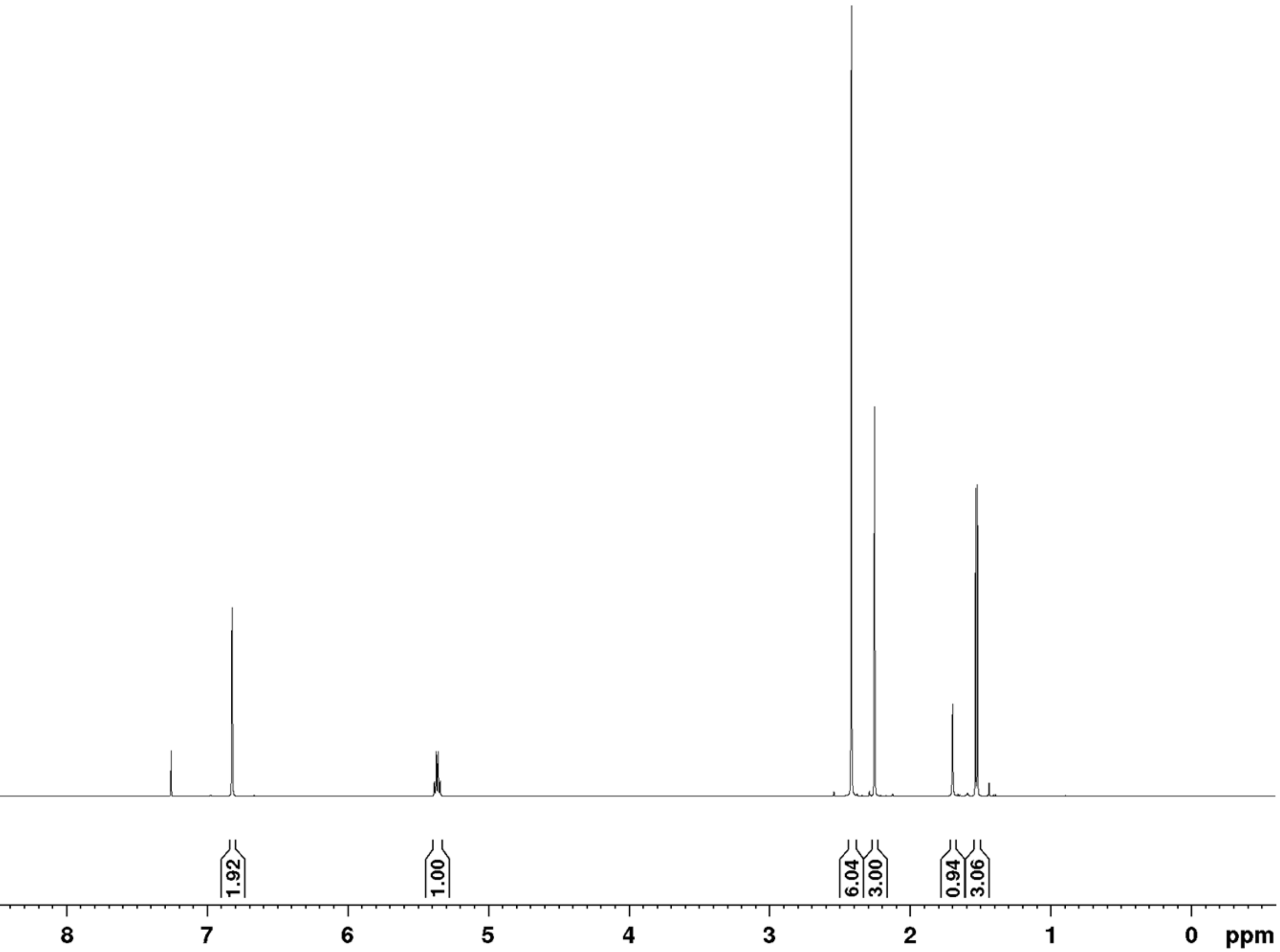

Supplementary Figure 166. ^{13}C **NMR (126 MHz,** $CDCl_3$**) of (*R*)-1-Mesitylethan-1-ol [(*R*)-1k]**

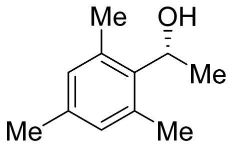

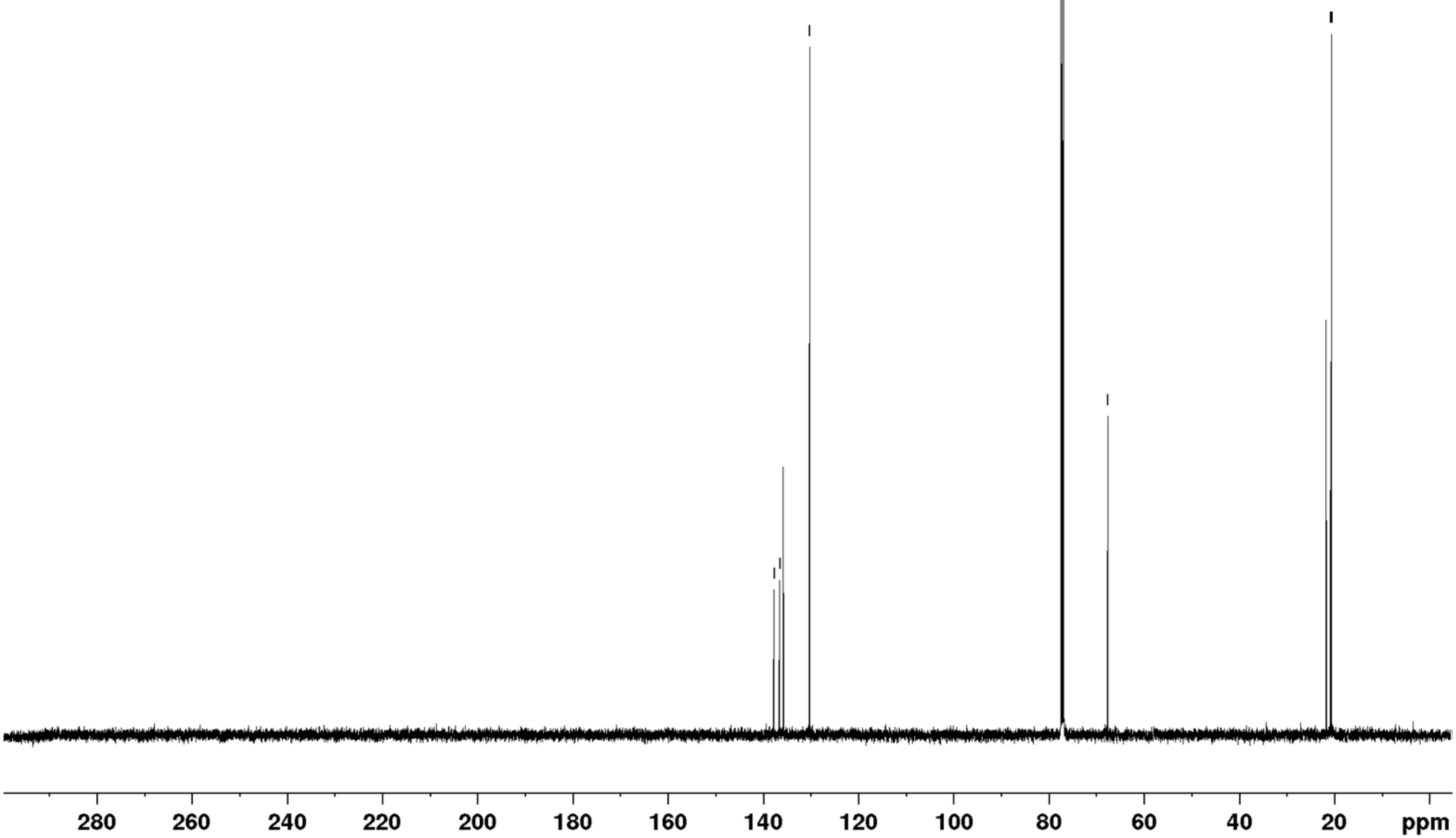

Supplementary Figure 167. ^{1}H NMR (500 MHz, C_6D_6) of (*S*)-Tributyl(mesitylethoxy)silane [(*S*)-3kh]

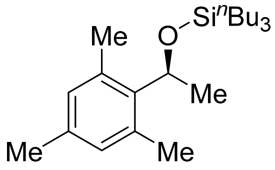

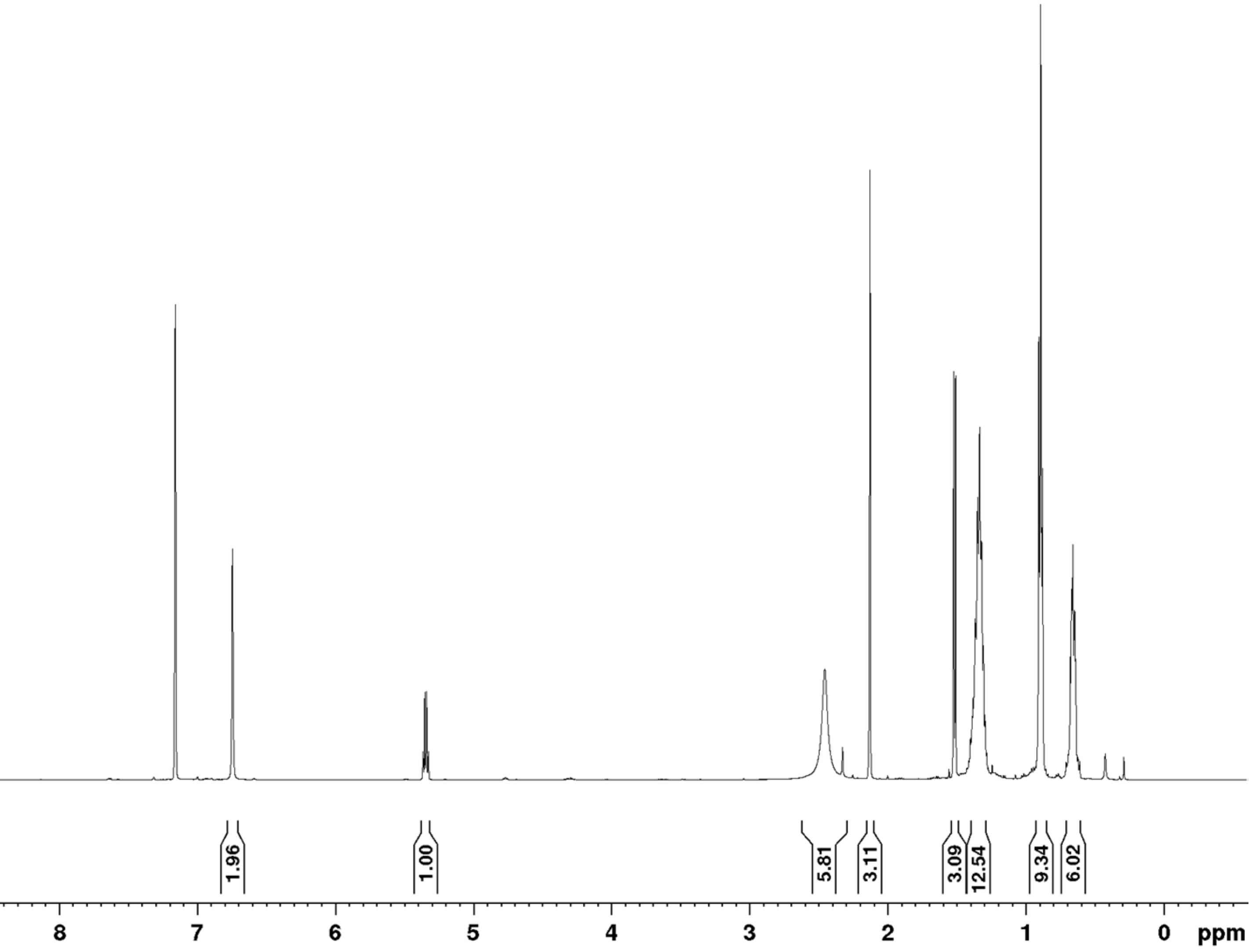

Supplementary Figure 168. ^{13}**C NMR (126 MHz, C_6D_6) of (*S*)-Tributyl(mesitylethoxy)silane [(*S*)-3kh]**

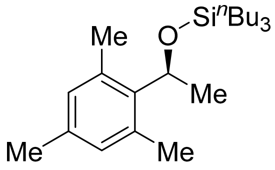

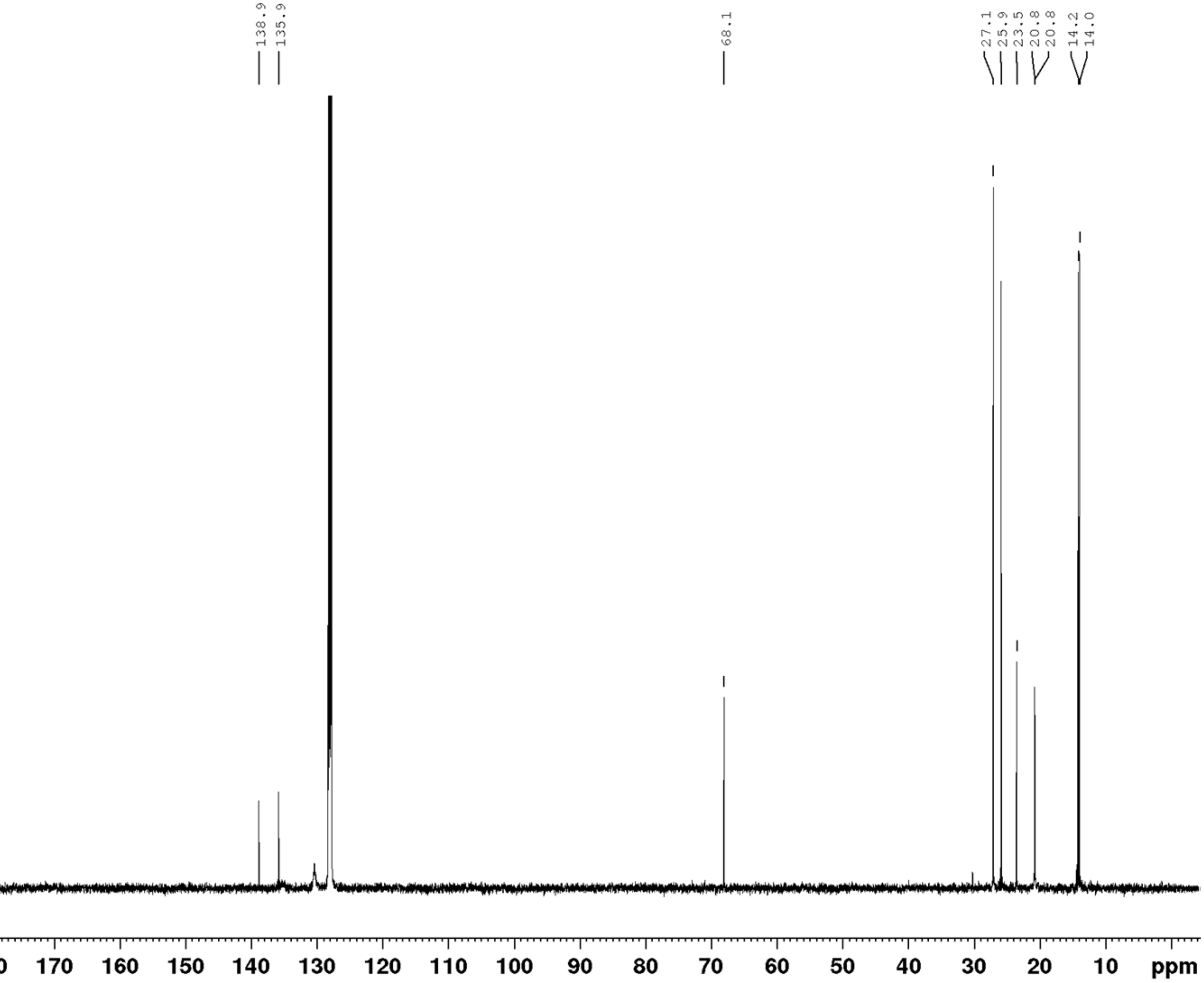

Supplementary Figure 169. ^{1}H NMR (500 MHz, $CDCl_3$) of (*R*)-1-([1,1'-Biphenyl]-4-yl)propan-1-ol [(*R*)-1l]

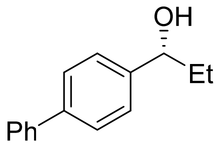

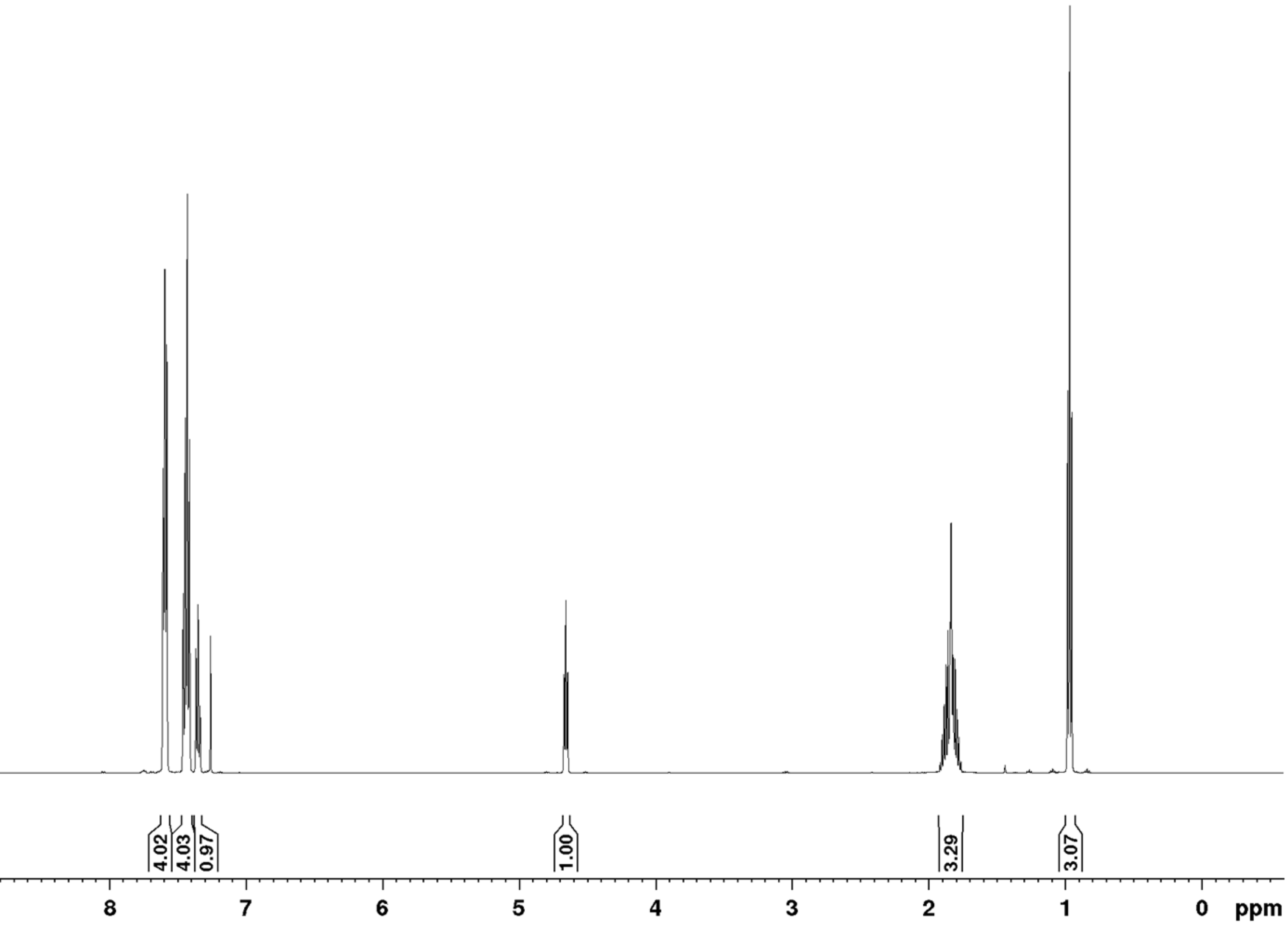

Supplementary Figure 170. ^{13}C NMR (126 MHz, $CDCl_3$) of (*R*)-1-([1,1'-Biphenyl]-4-yl)propan-1-ol [(*R*)-1l]

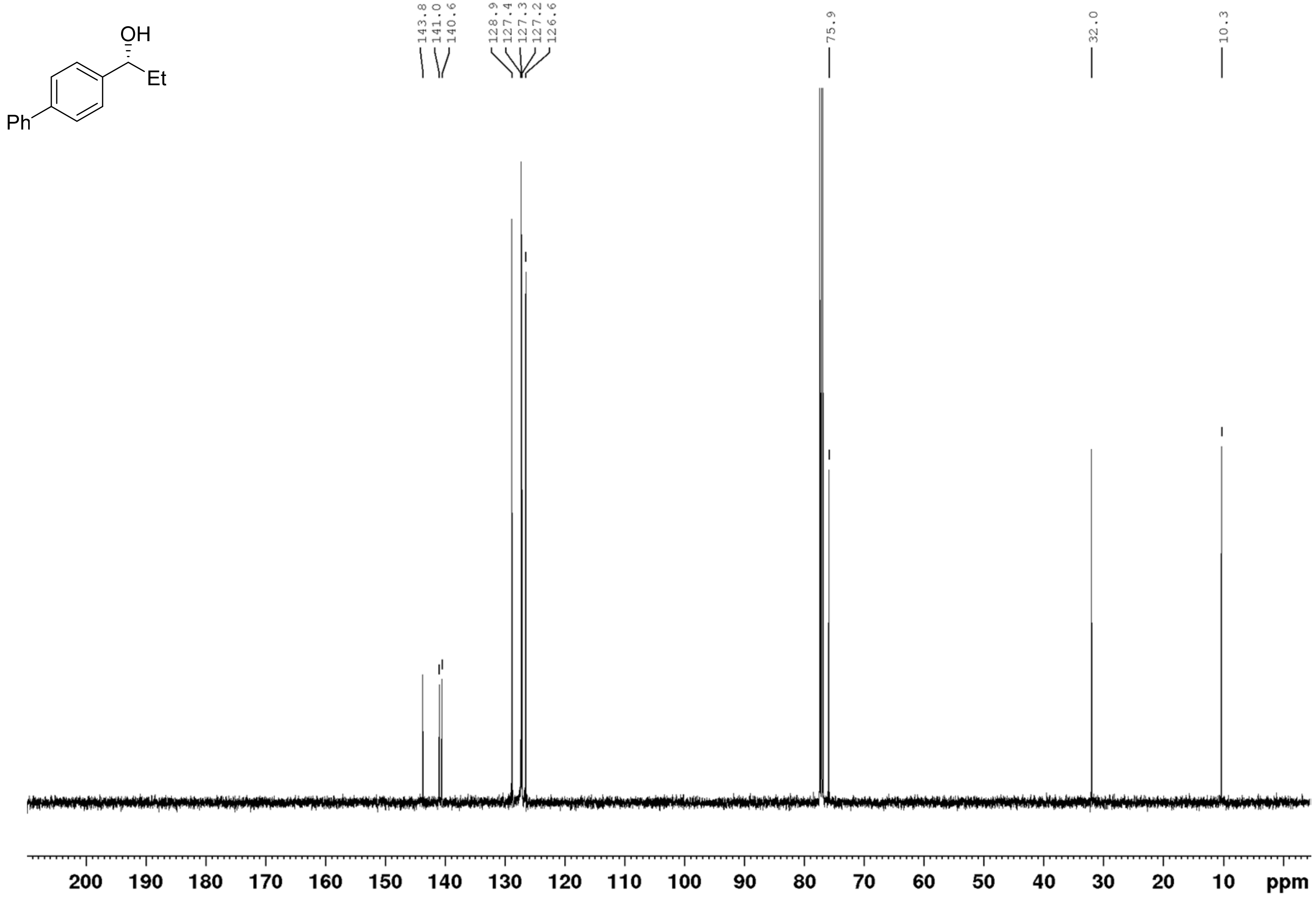

Supplementary Figure 171. ^{1}H NMR (500 MHz, C_6D_6) of (*S*)-(1-([1,1'-Biphenyl]-4-yl)propoxy)tributylsilane [(*S*)-3lh]

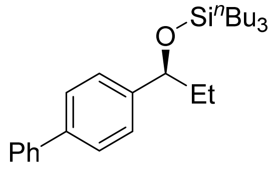

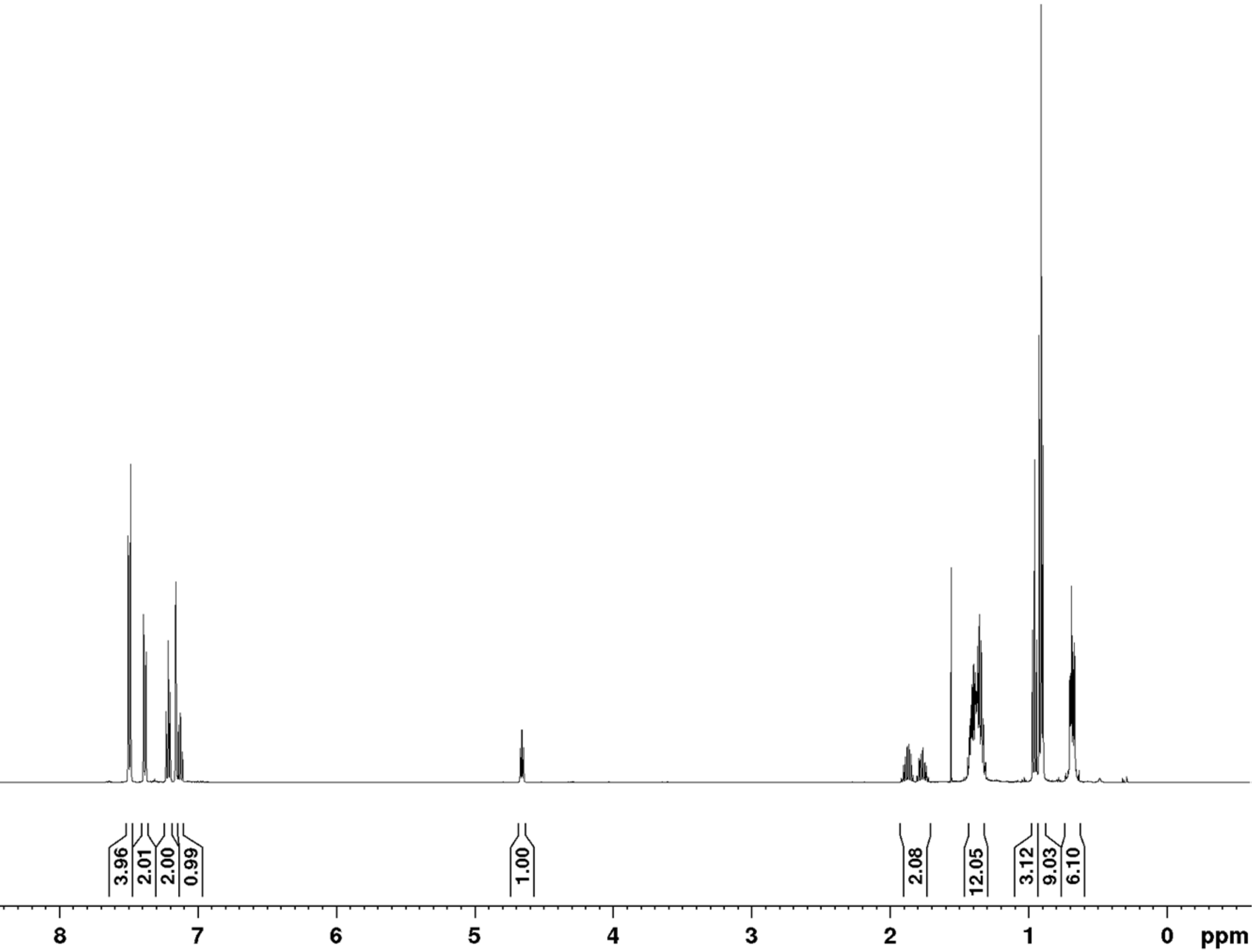

Supplementary Figure 172. ^{13}**C NMR (126 MHz,** C_6D_6**) of (*S*)-(1-([1,1'-Biphenyl]-4-yl)propoxy)tributylsilane [(*S*)-3lh]**

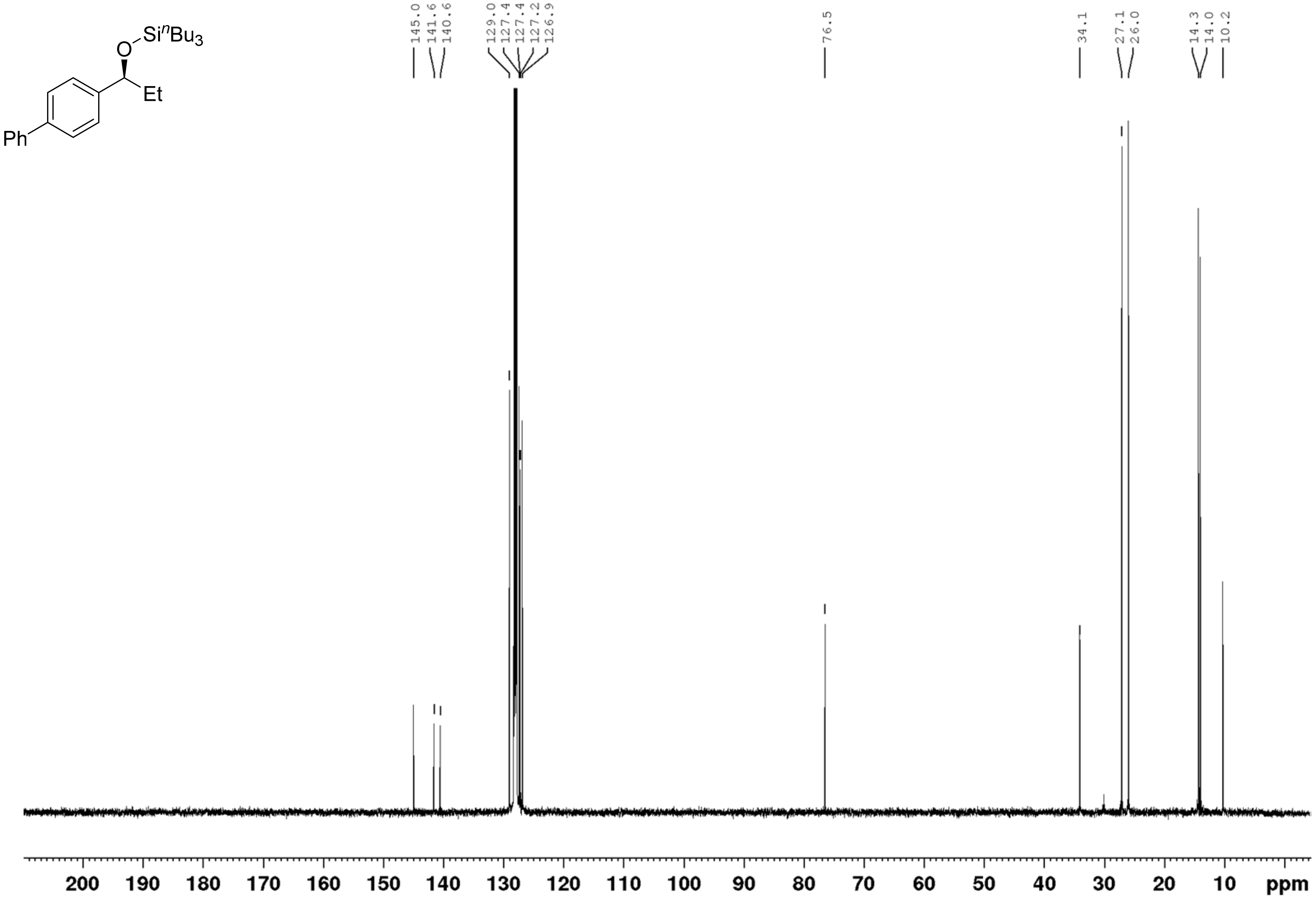

Supplementary Figure 173. ^{1}H NMR (400 MHz, $CDCl_3$) of (*R*)-1,2-Diphenylethan-1-ol [(*R*)-1m]

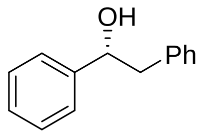

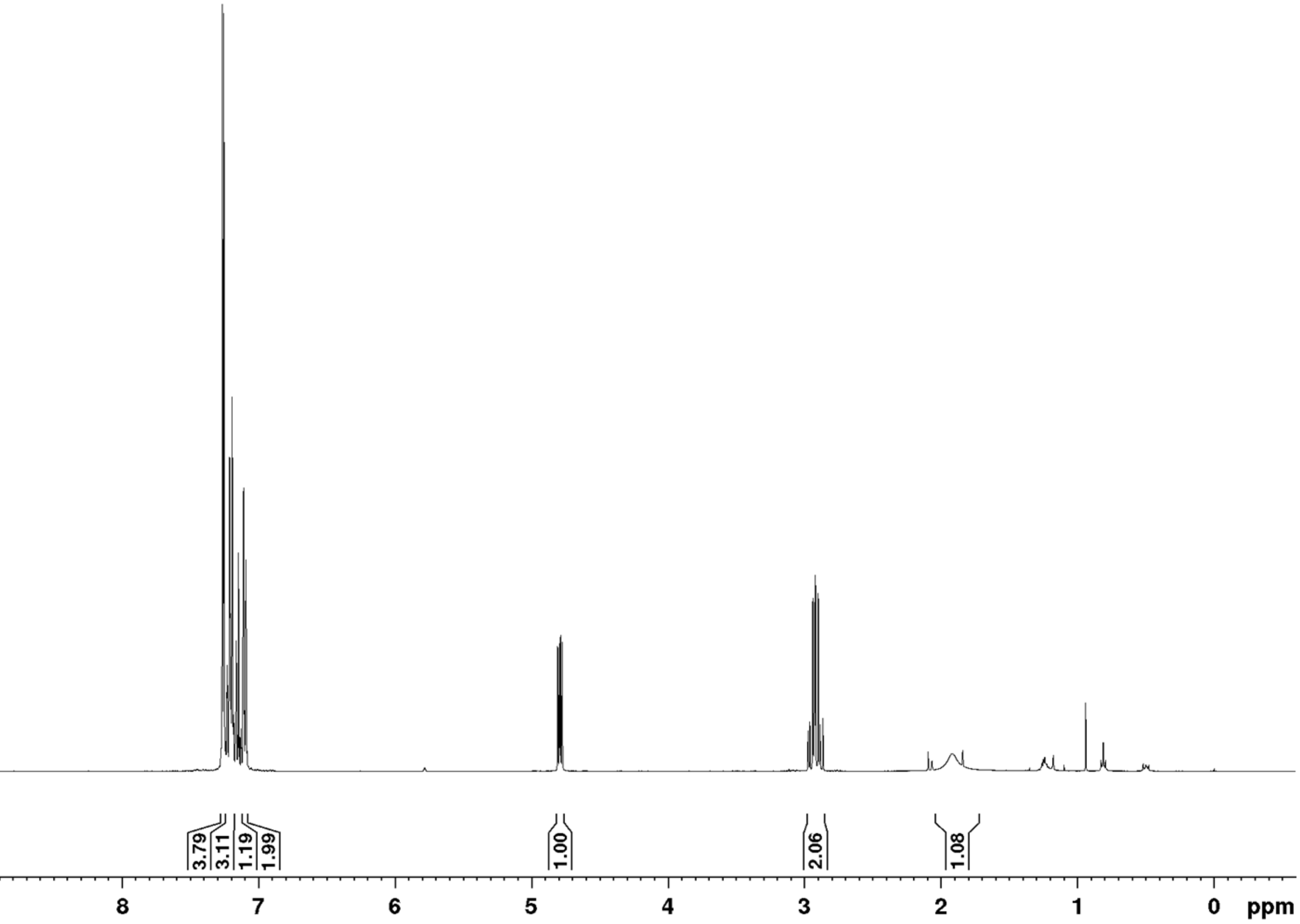

Supplementary Figure 174. ^{13}C NMR (126 MHz, $CDCl_3$) of (*R*)-1,2-Diphenylethan-1-ol [(*R*)-1m]

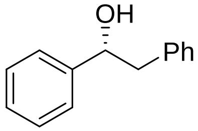

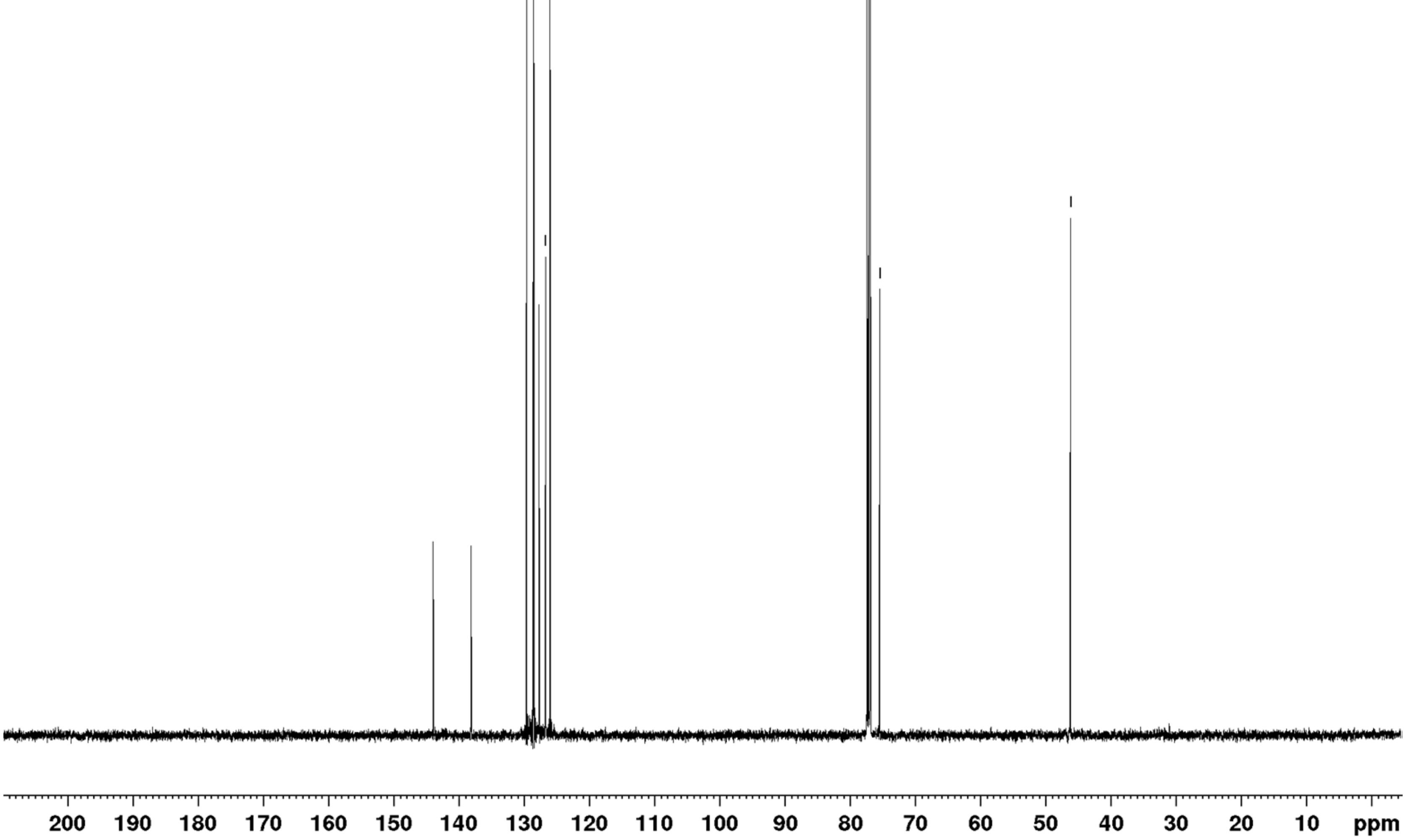

Supplementary Figure 175. ^{1}**H NMR (500 MHz, C_6D_6) of (*S*)-Tributyl(1,2-diphenylethoxy)silane [(*S*)-3mh]**

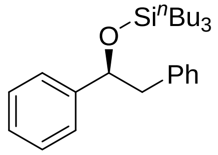

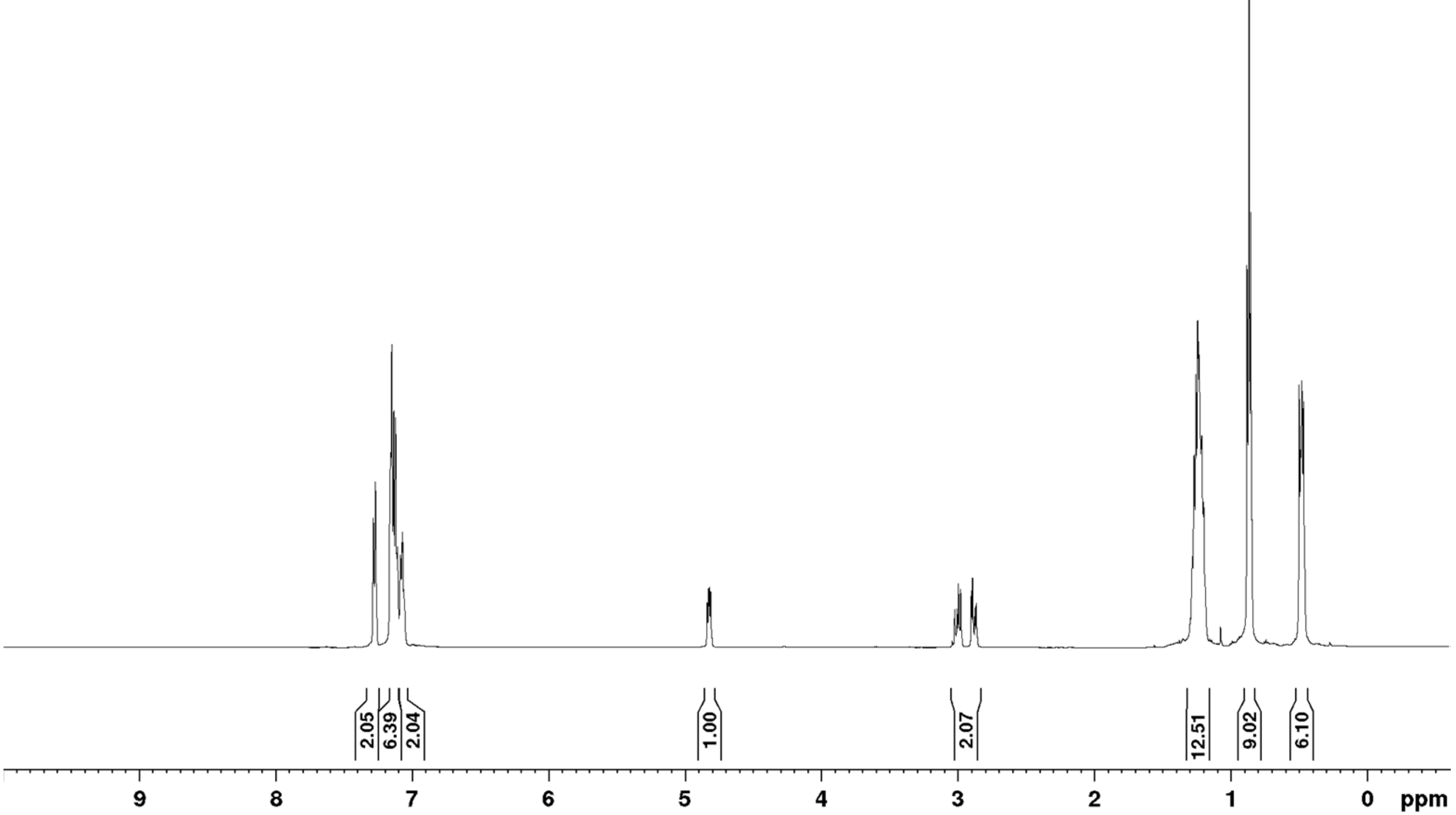

Supplementary Figure 176. ^{13}C NMR (126 MHz, C_6D_6) of (*S*)-Tributyl(1,2-diphenylethoxy)silane [(*S*)-3mh]

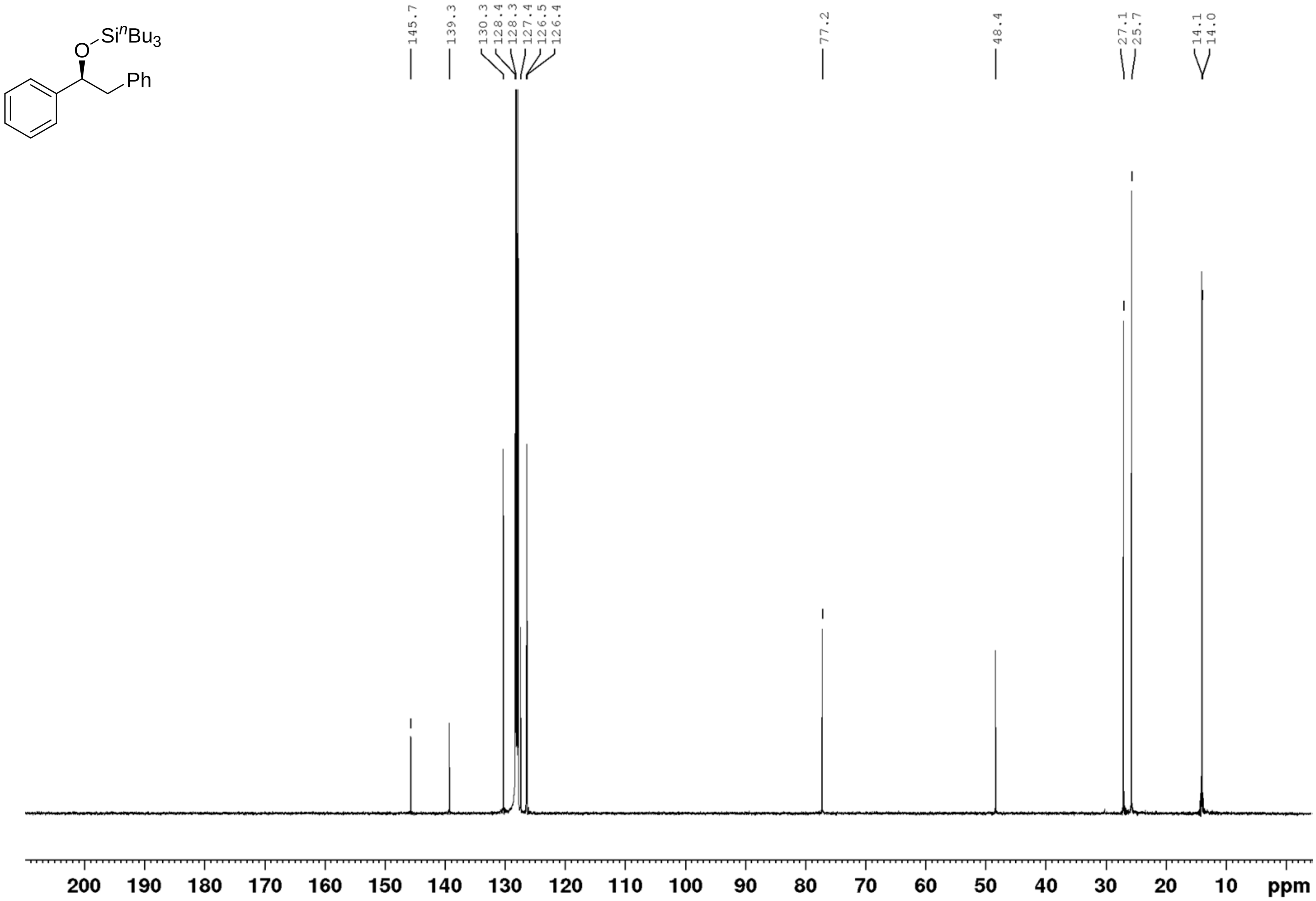

Supplementary Figure 177. ^{1}H NMR (500 MHz, $CDCl_3$) of (*R*)-1-([1,1'-Biphenyl]-4-yl)-2-methylpropan-1-ol [(*R*)-1n]

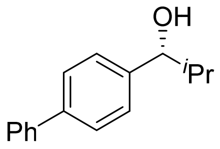

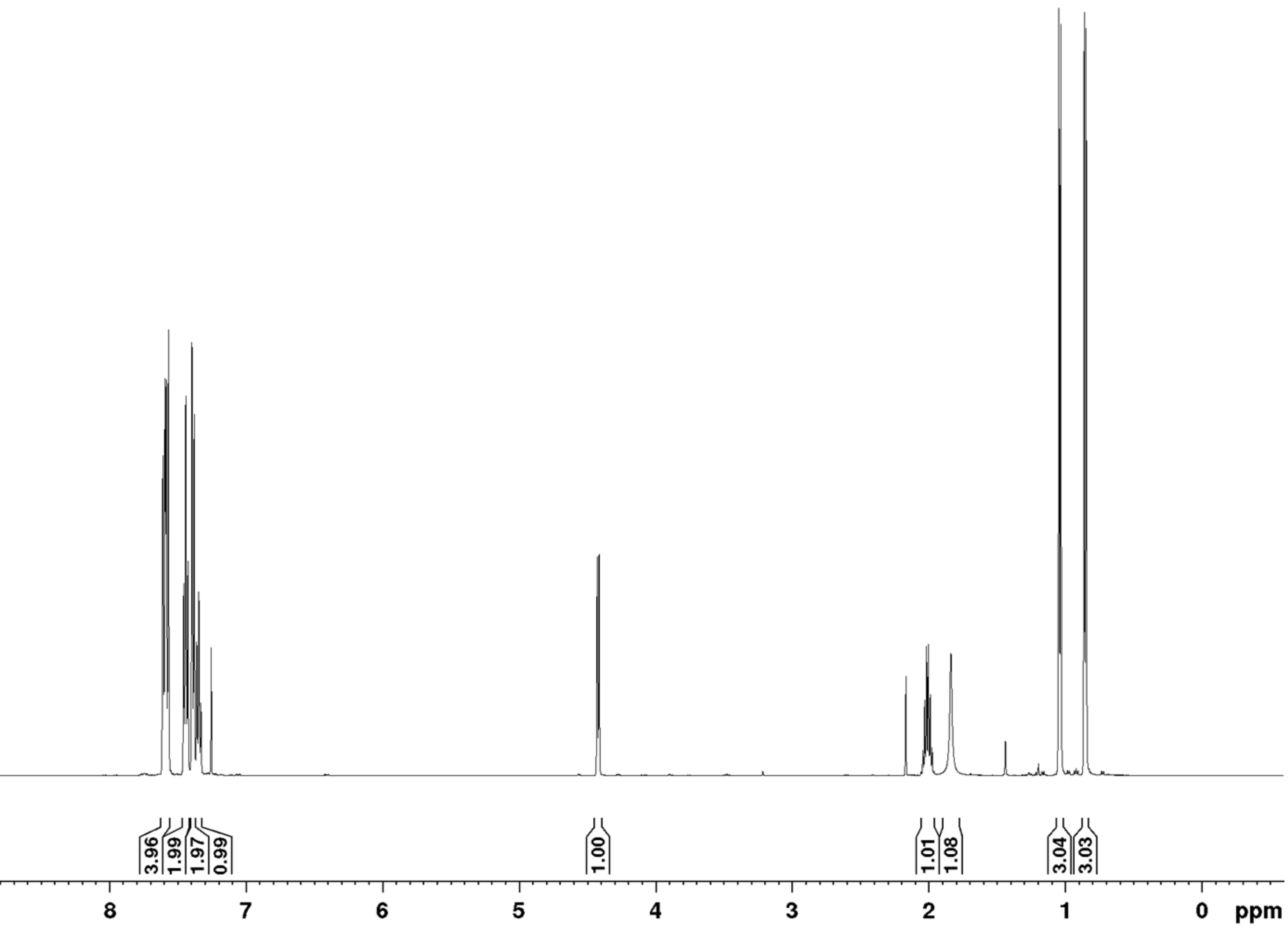

Supplementary Figure 178. ^{13}C NMR (126 MHz, $CDCl_3$) of (*R*)-1-([1,1'-Biphenyl]-4-yl)-2-methylpropan-1-ol [(*R*)-1n]

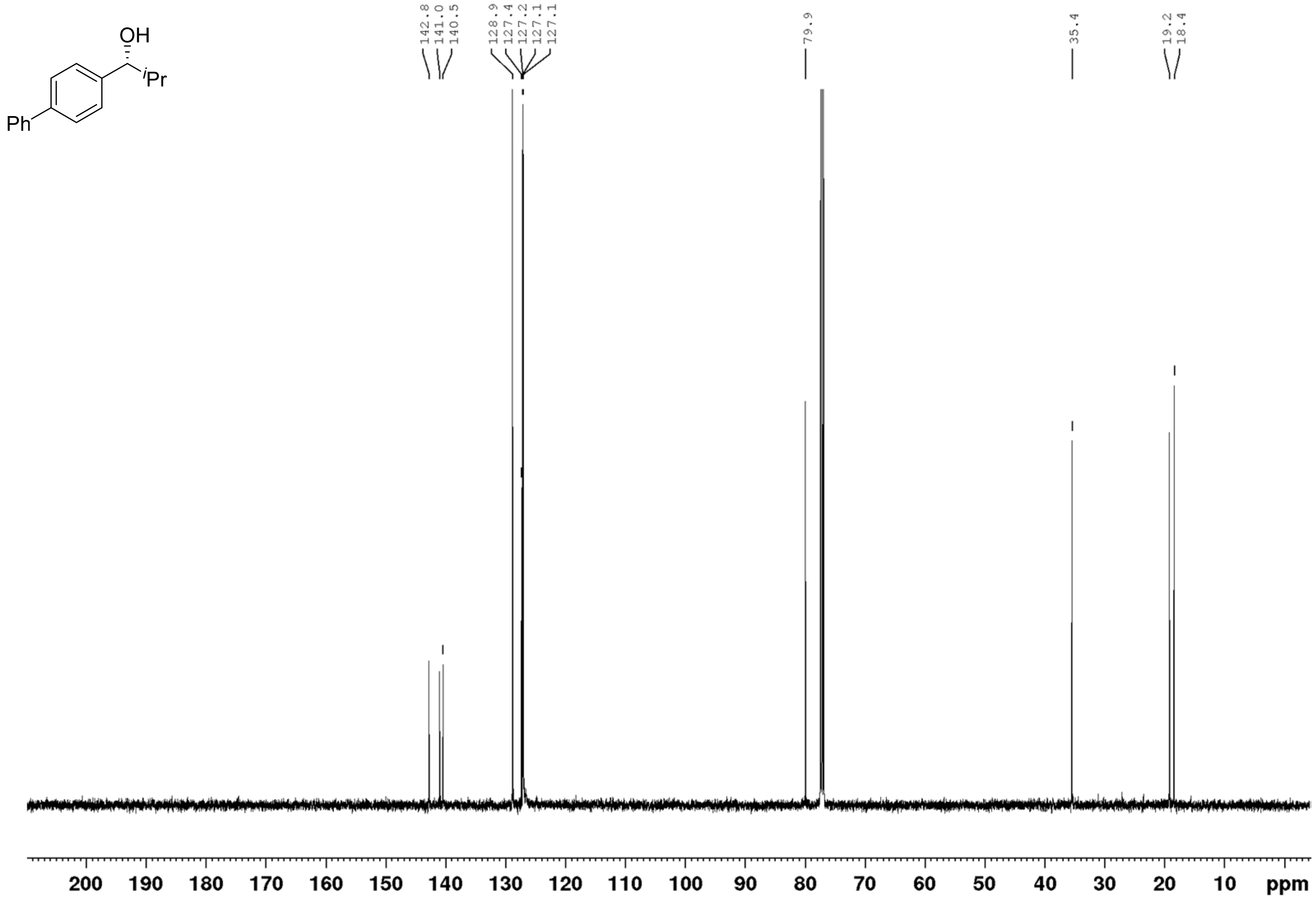

Supplementary Figure 179. ^{1}H NMR (500 MHz, C_6D_6) of (*S*)-(1-([1,1'-Biphenyl]-4-yl)-2-methylpropoxy)tributylsilane [(*S*)-3nh]

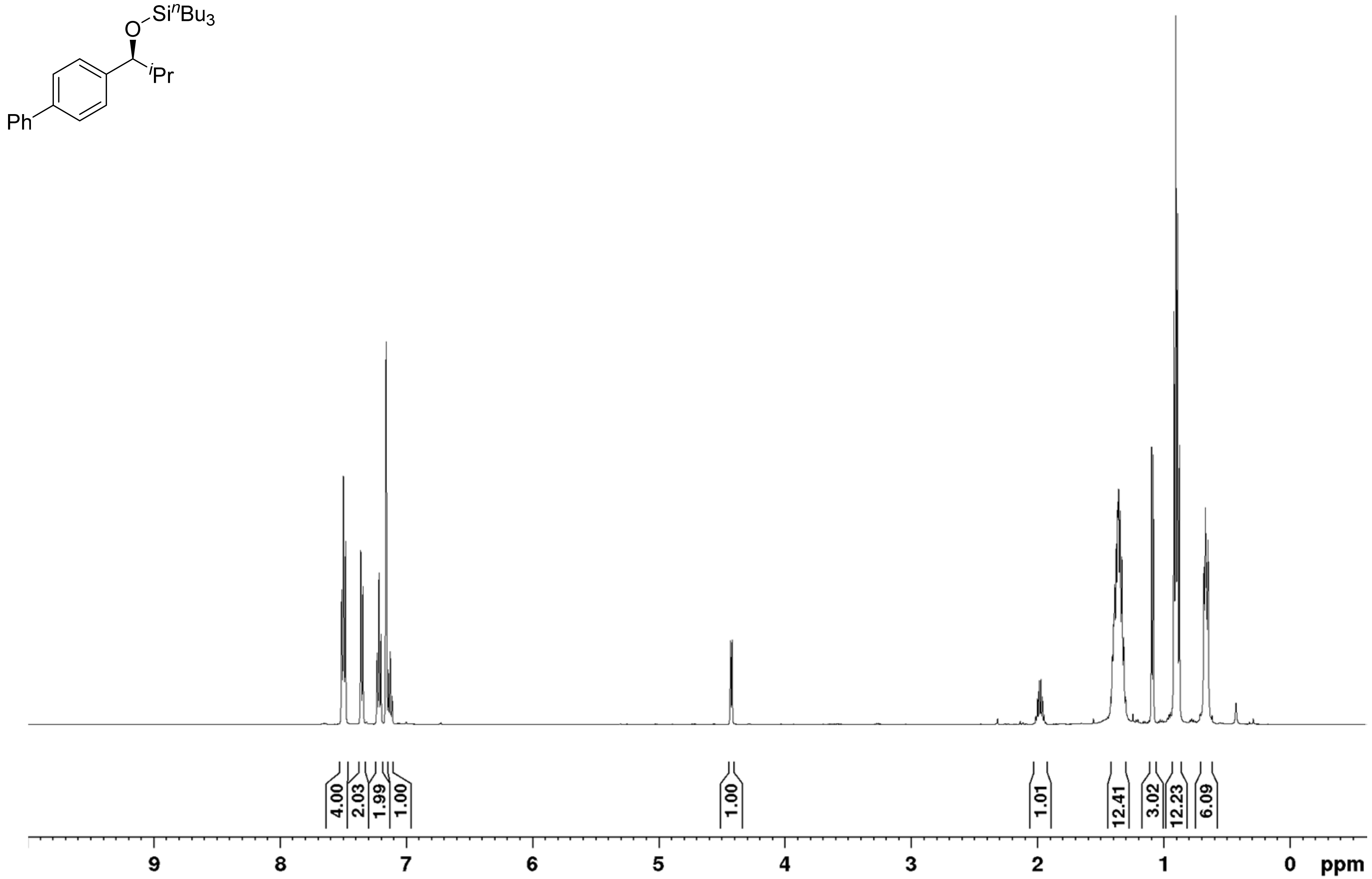

Supplementary Figure 180. ^{13}C NMR (126 MHz, C_6D_6) of (*S*)-(1-([1,1'-Biphenyl]-4-yl)-2-methylpropoxy)tributylsilane [(*S*)-3nh]

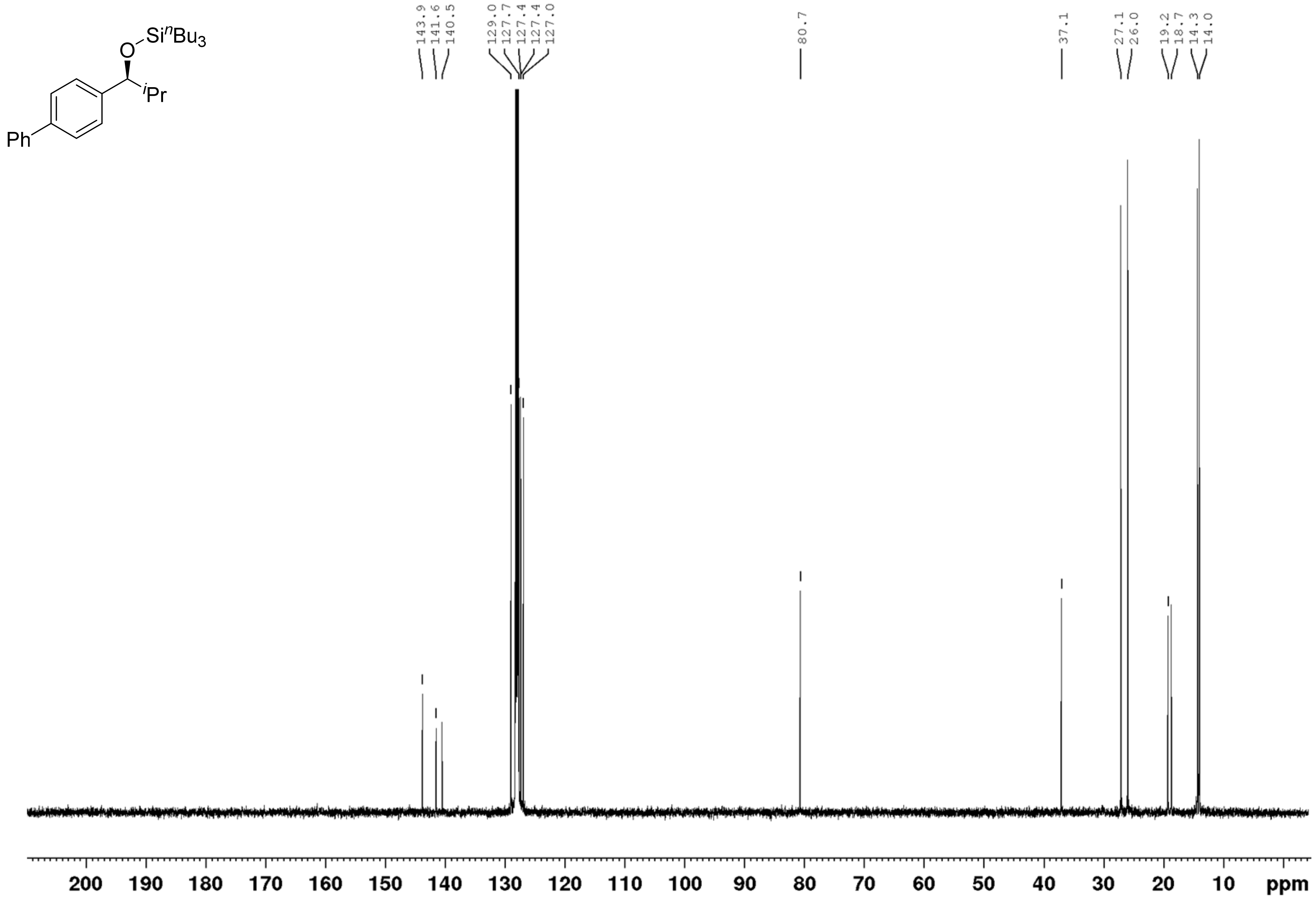

Supplementary Figure 181. ^{1}H NMR (400 MHz, C_6D_6) of (*R*)-1-Mesitylpropan-1-ol [(*R*)-1o]

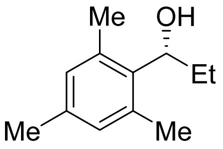

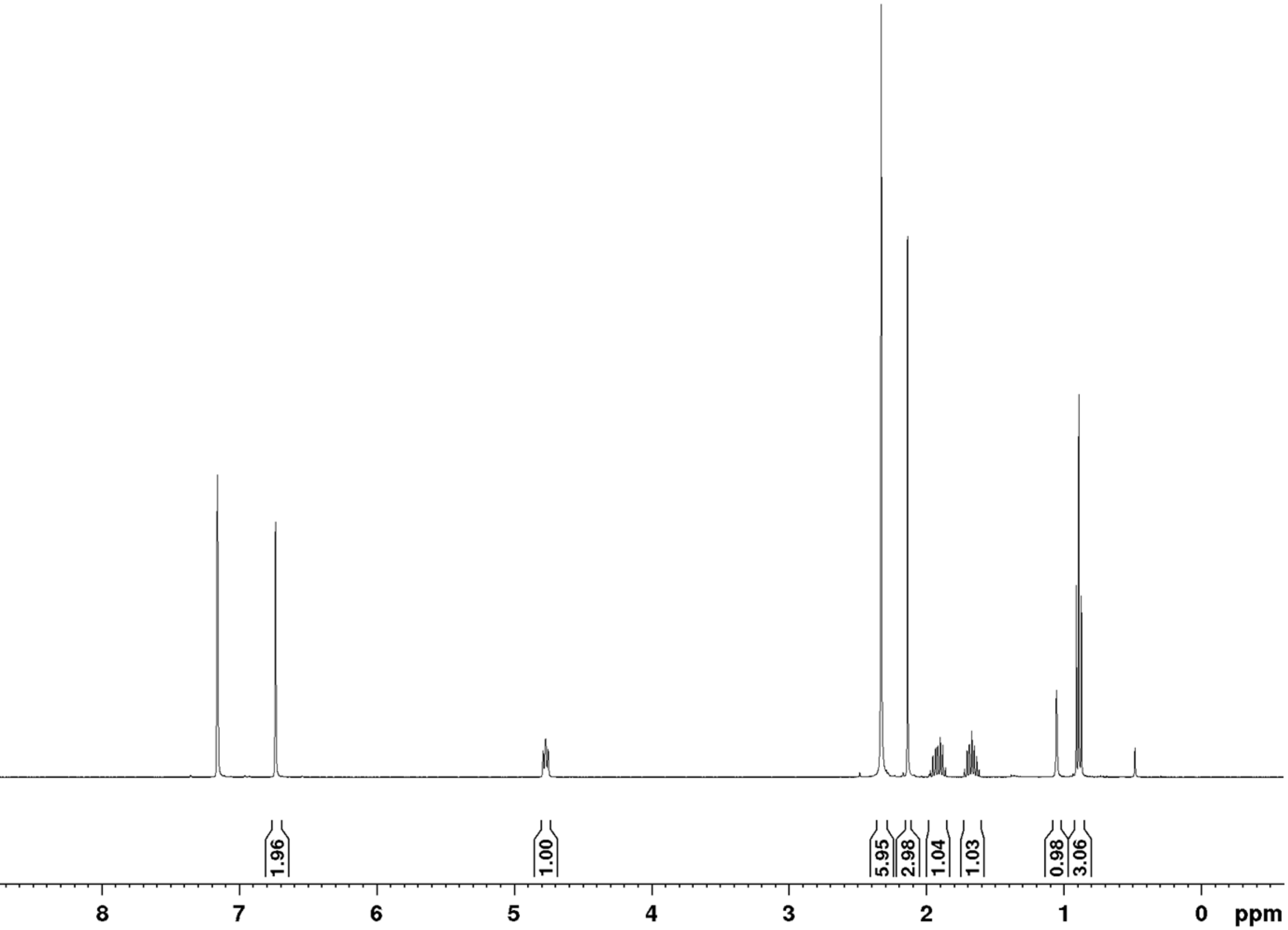

Supplementary Figure 182. ^{13}C NMR (126 MHz, C_6D_6) of (*R*)-1-Mesitylpropan-1-ol [(*R*)-1o]

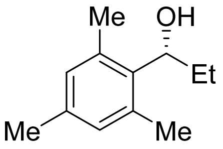

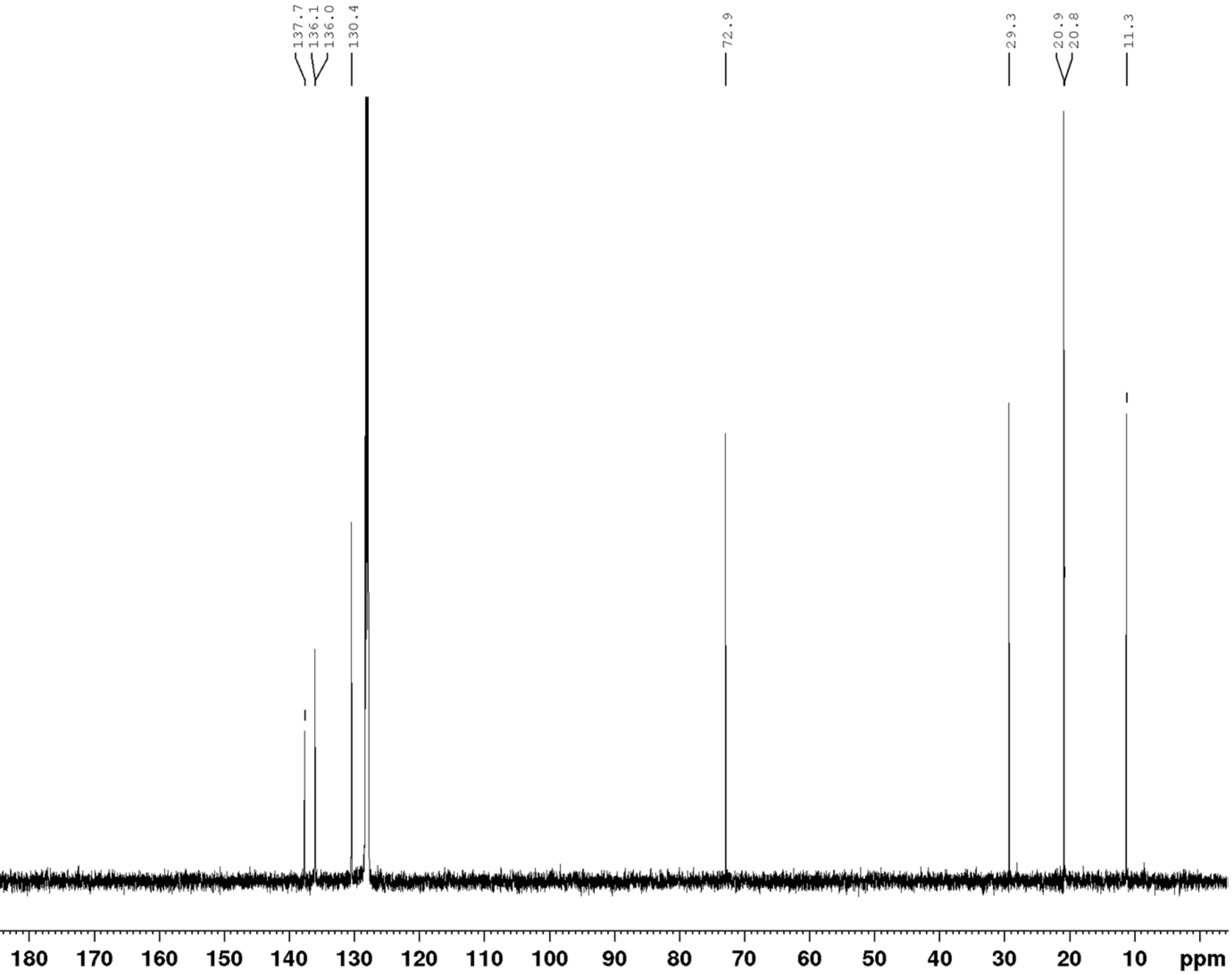

Supplementary Figure 183. ^{1}H NMR (500 MHz, C_6D_6) of (*S*)-Tributyl(1-mesitylpropoxy)silane [(*S*)-3oh]

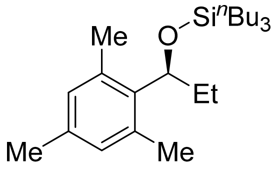

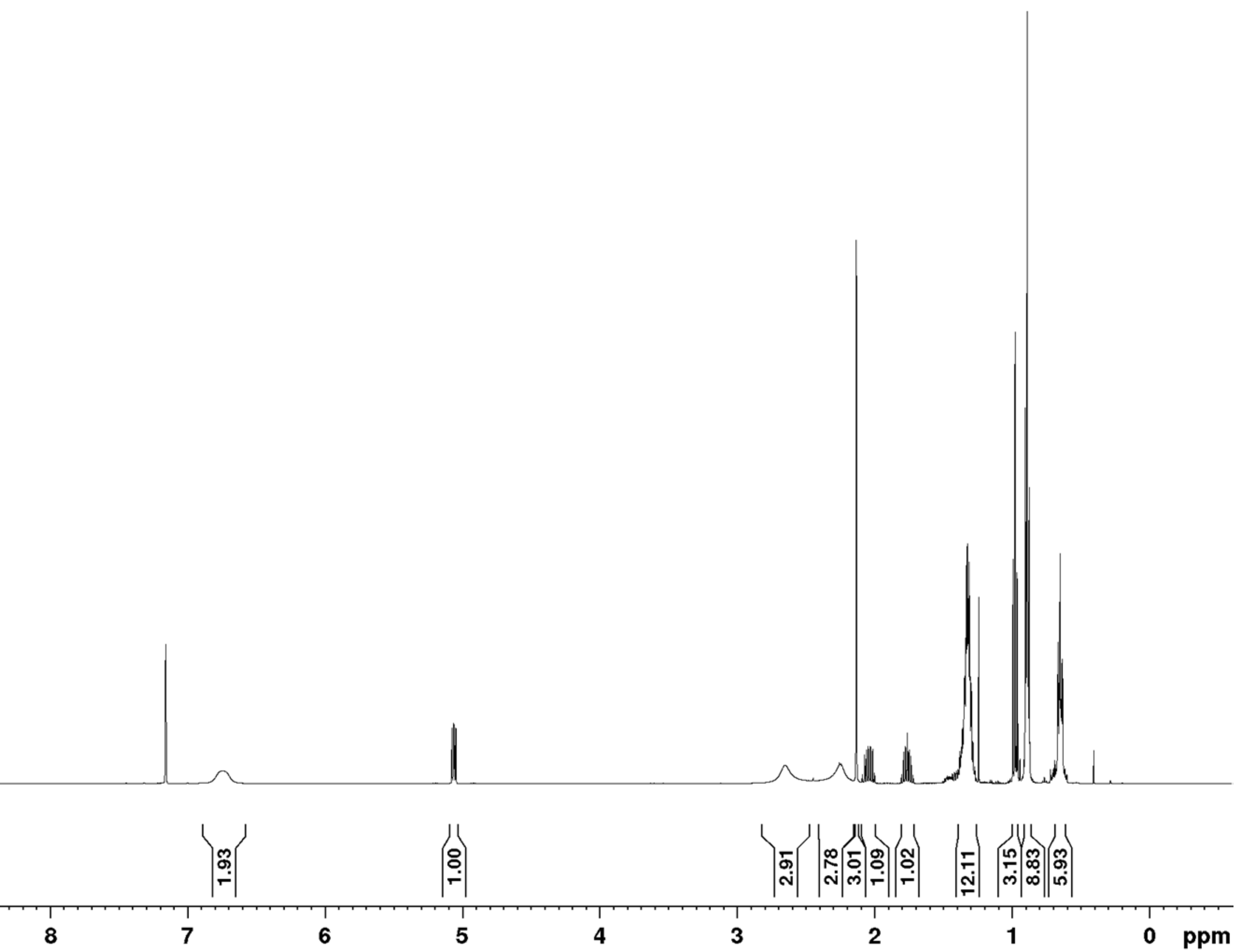

Supplementary Figure 184. ^{13}**C NMR (126 MHz,** C_6D_6**) of (*S*)-Tributyl(1-mesitylpropoxy)silane [(*S*)-3oh]**

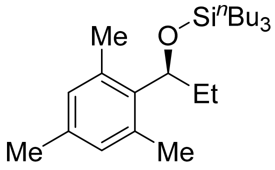

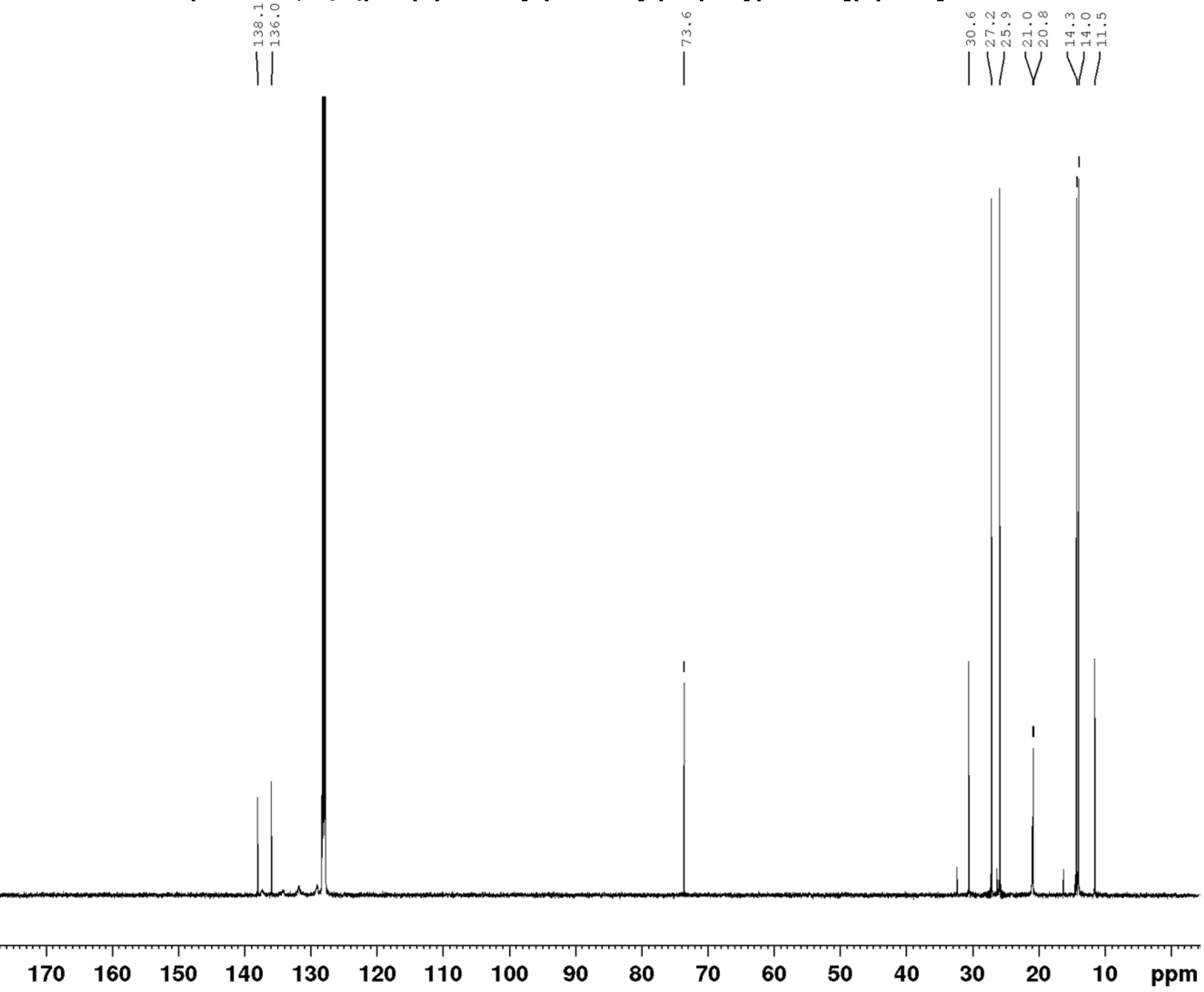

Supplementary Figure 185. ^{1}H NMR (500 MHz, $CDCl_3$) of (*R*)-1-(Naphthalen-2-yl)ethan-1-ol [(*R*)-1p]

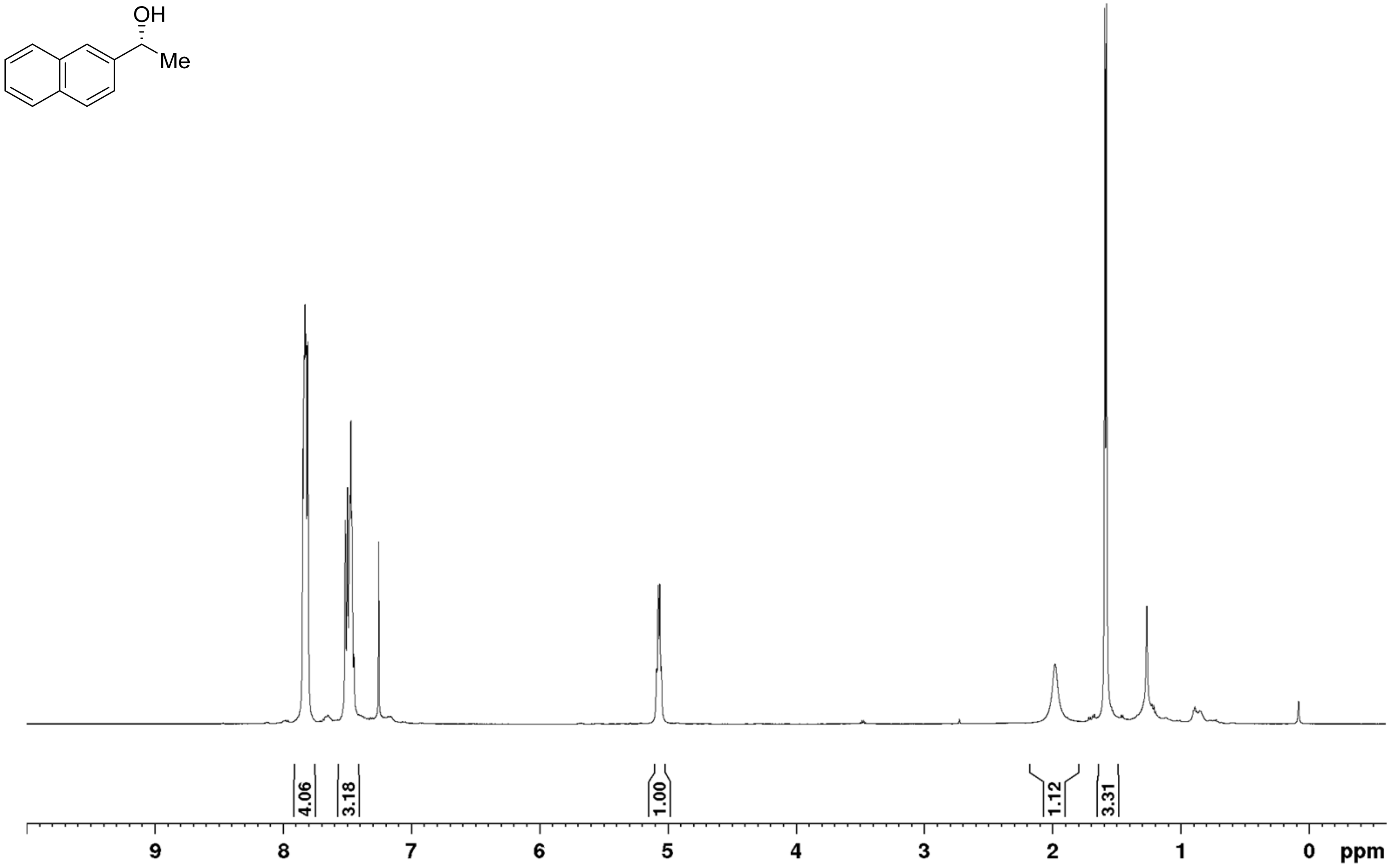

Supplementary Figure 186. ^{13}**C NMR (126 MHz, CDCl$_3$) of (*R*)-1-(Naphthalen-2-yl)ethan-1-ol [(*R*)-1p]**

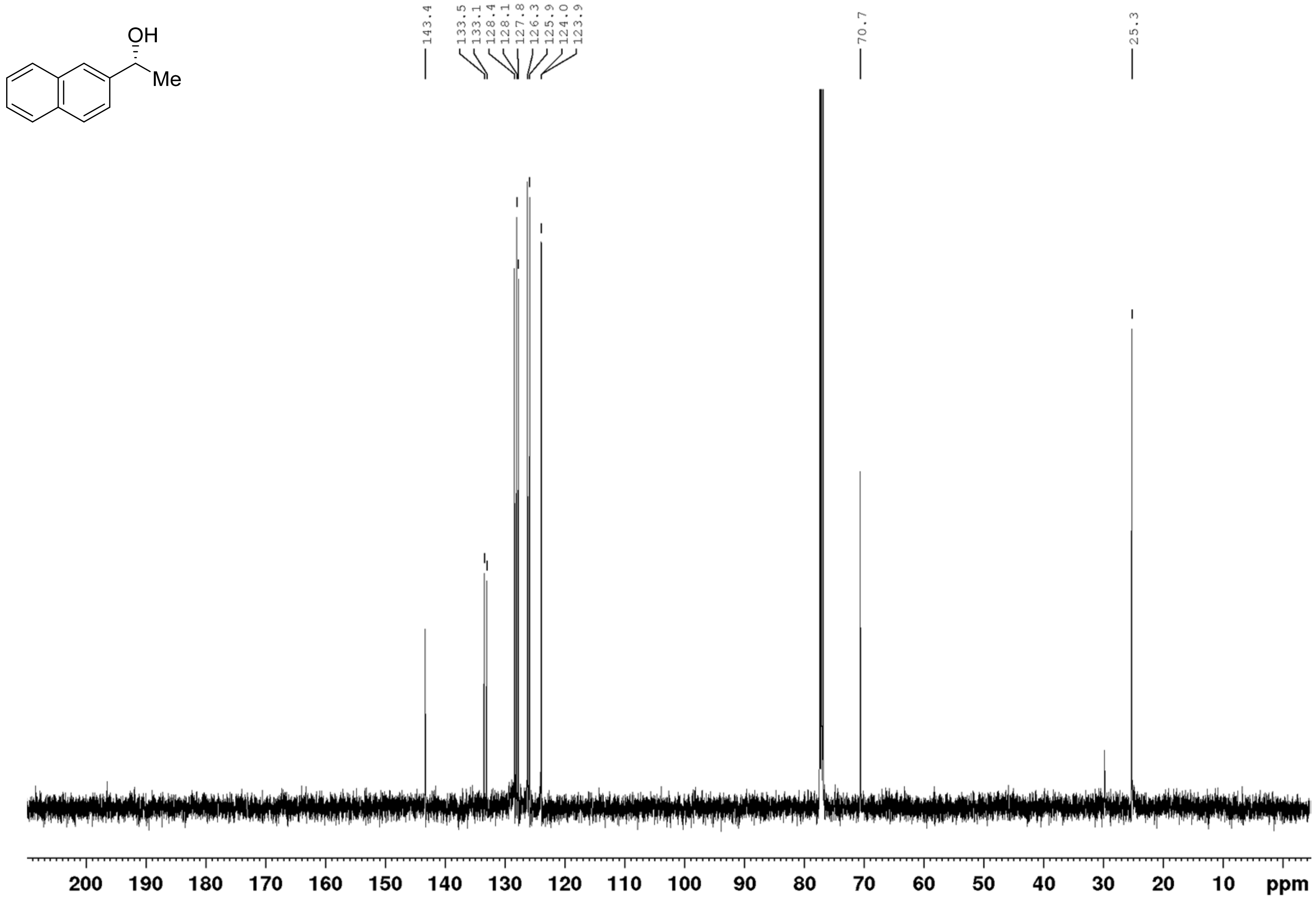

Supplementary Figure 187. ^{1}**H NMR (500 MHz, C_6D_6) of (*S*)-Tributyl(1-(naphthalen-2-yl)ethoxy)silane [(*S*)-3ph]**

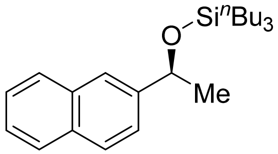

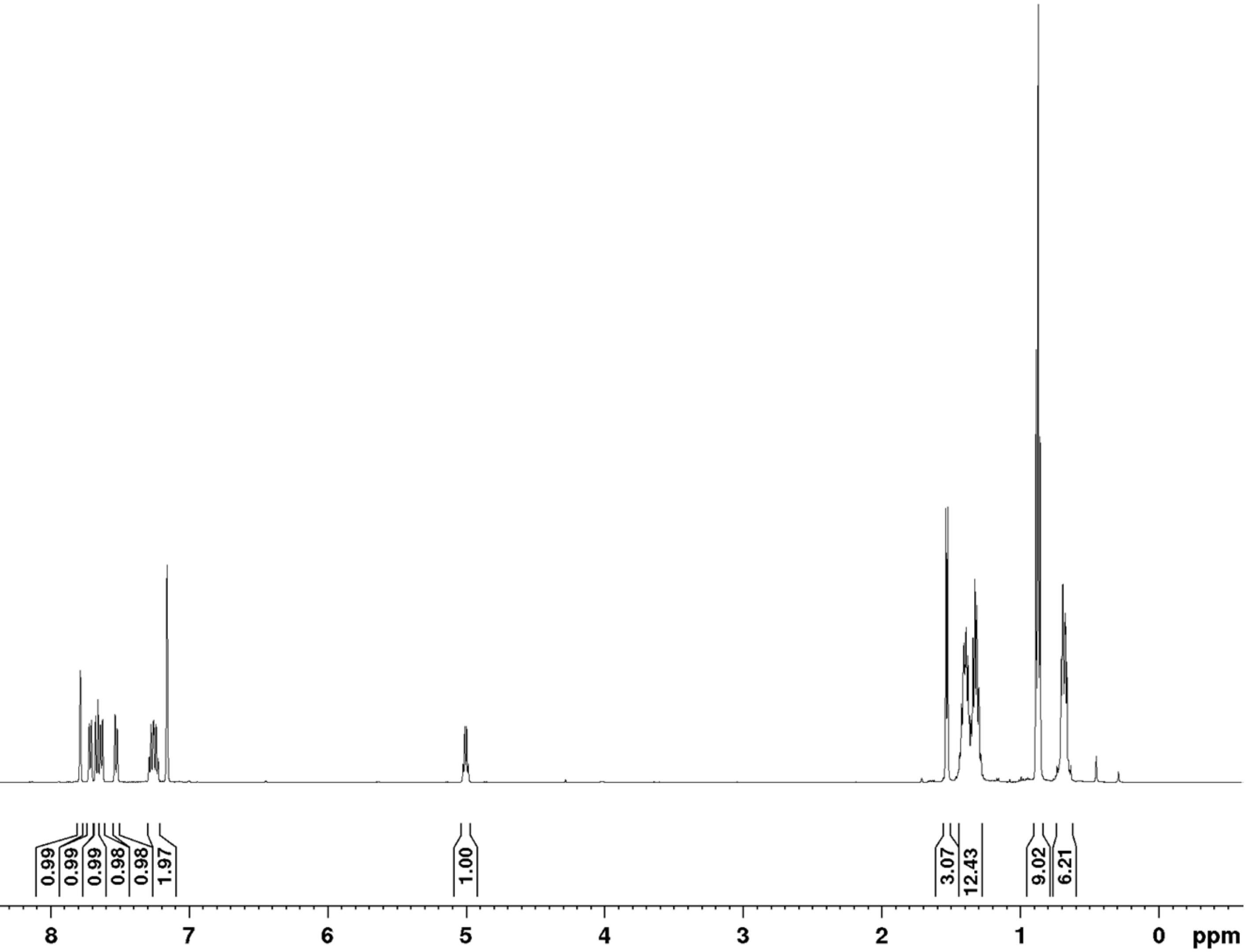

Supplementary Figure 188. ^{13}**C NMR (126 MHz, C_6D_6) of (*S*)-Tributyl(1-(naphthalen-2-yl)ethoxy)silane [(*S*)-3ph]**

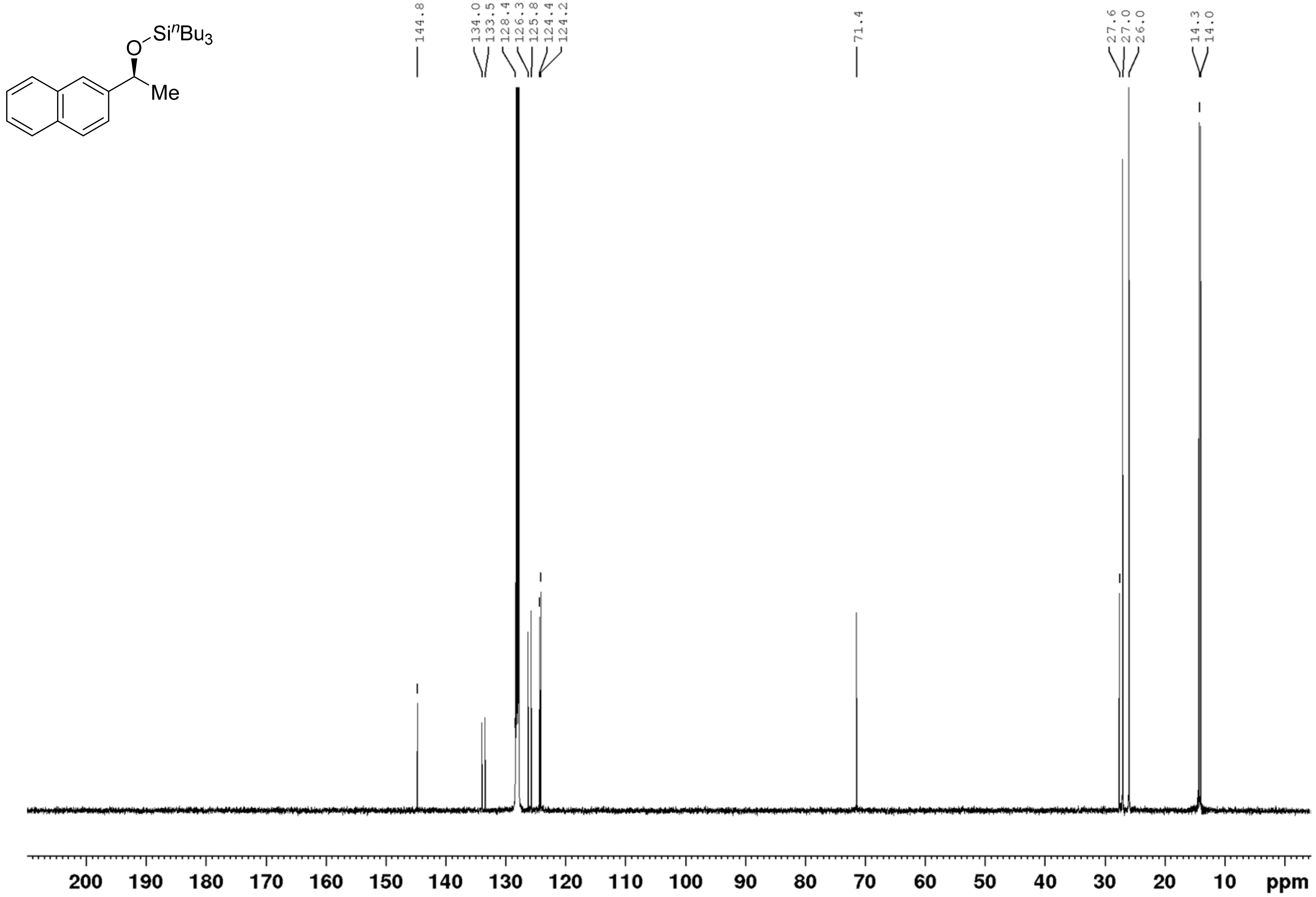

Supplementary Figure 189. ^{1}**H NMR (500 MHz, CDCl$_3$) of (*R*)-1-(Naphthalen-1-yl)ethan-1-ol [(*R*)-1q]**

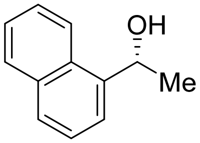

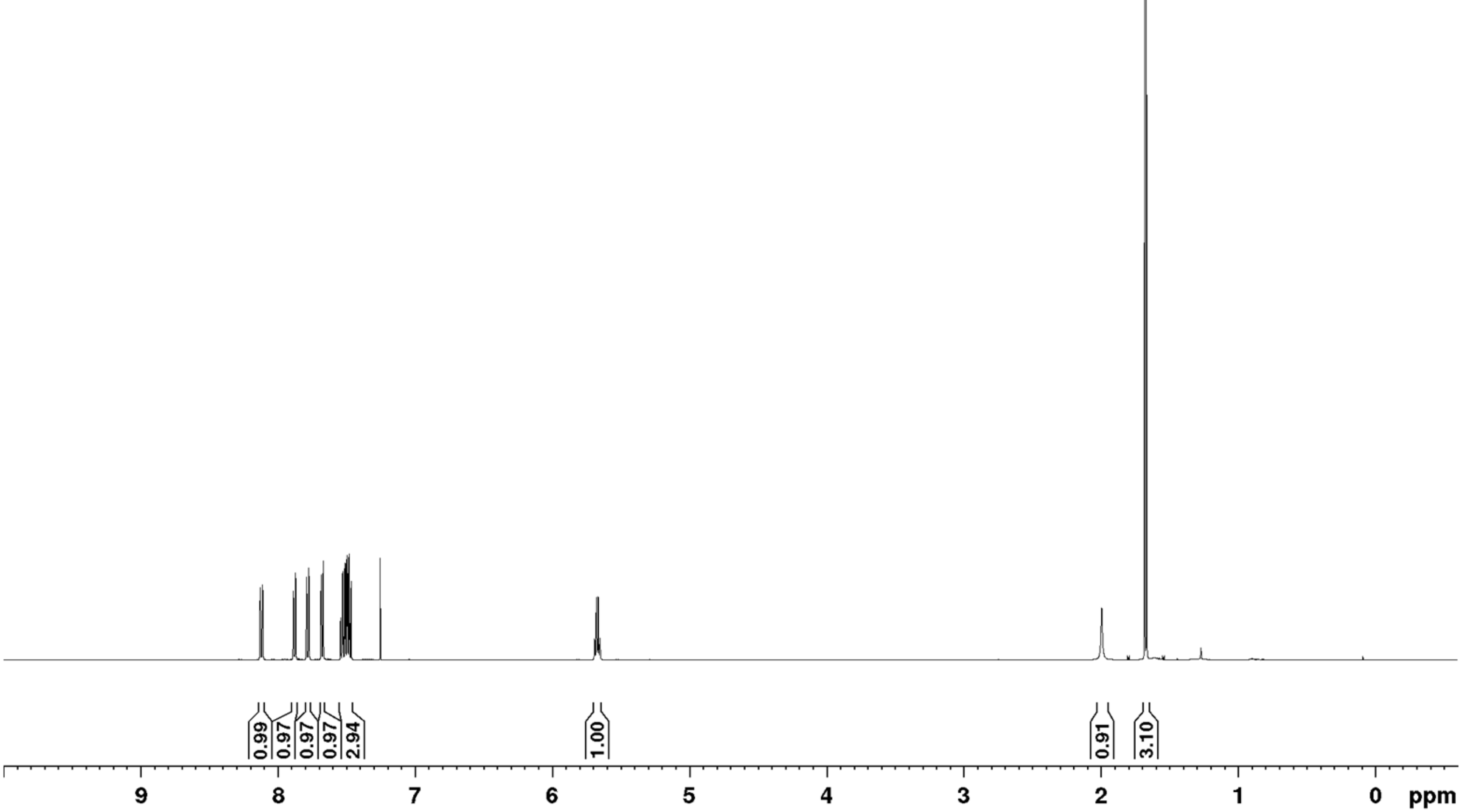

Supplementary Figure 190. ^{13}C NMR (126 MHz, $CDCl_3$) of (*R*)-1-(Naphthalen-1-yl)ethan-1-ol [(*R*)-1q]

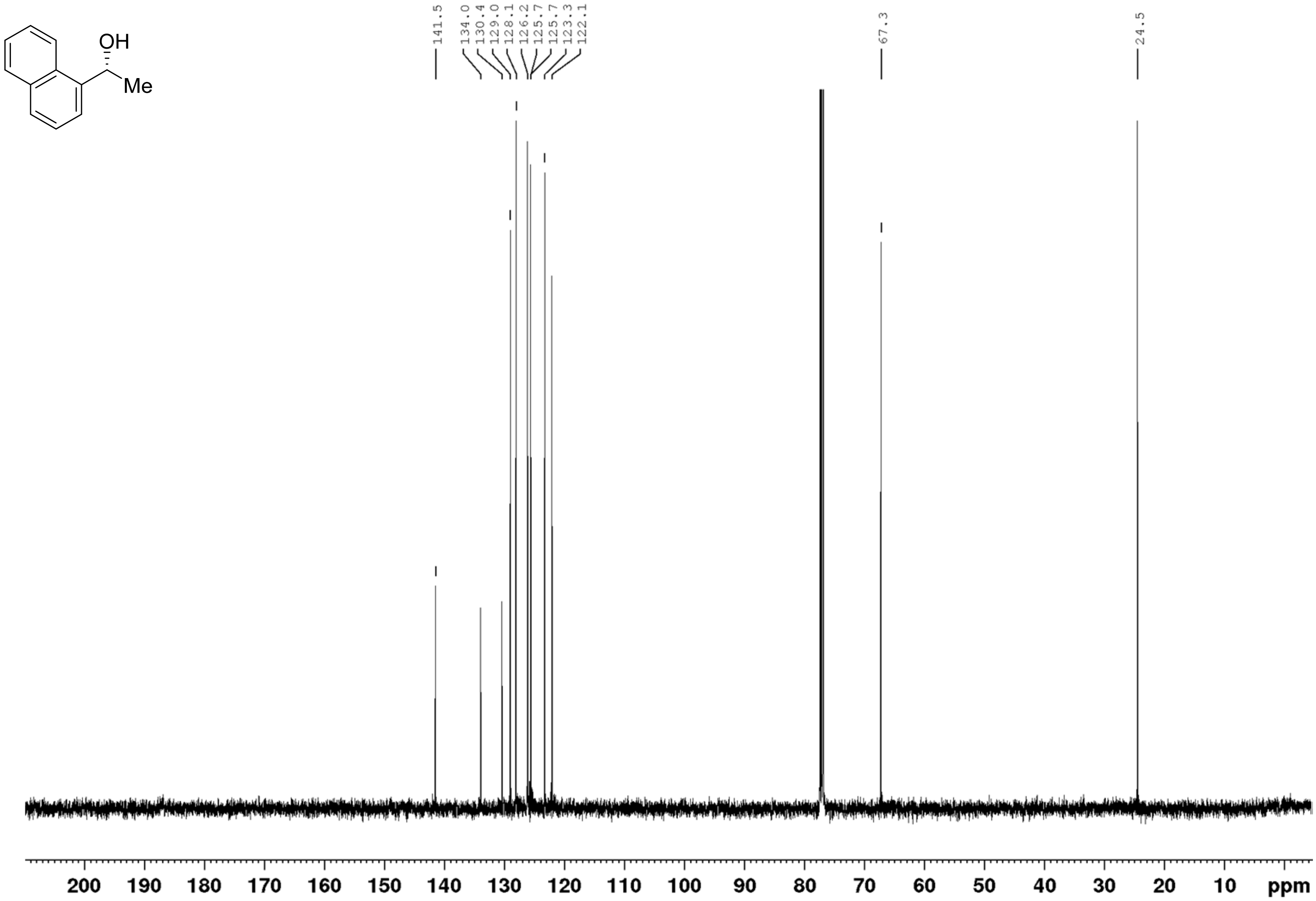

Supplementary Figure 191. ¹H NMR (500 MHz, C_6D_6) of (*S*)-Tributyl(1-(naphthalen-1-yl)ethoxy)silane [(*S*)-3qh]

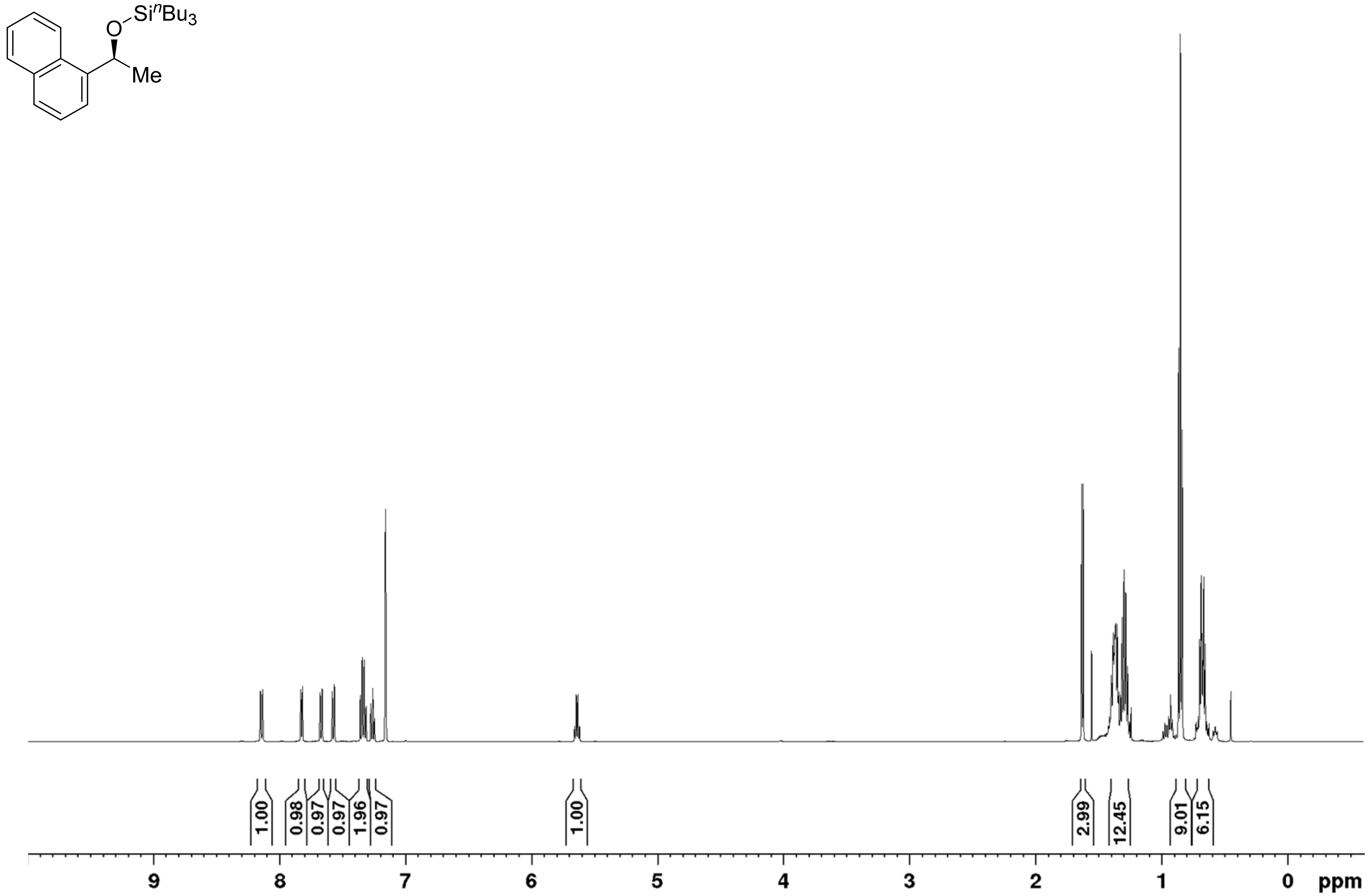

Supplementary Figure 192. ^{13}C NMR (126 MHz, C_6D_6) of (*S*)-Tributyl(1-(naphthalen-1-yl)ethoxy)silane [(*S*)-3qh]

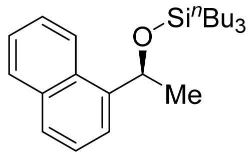

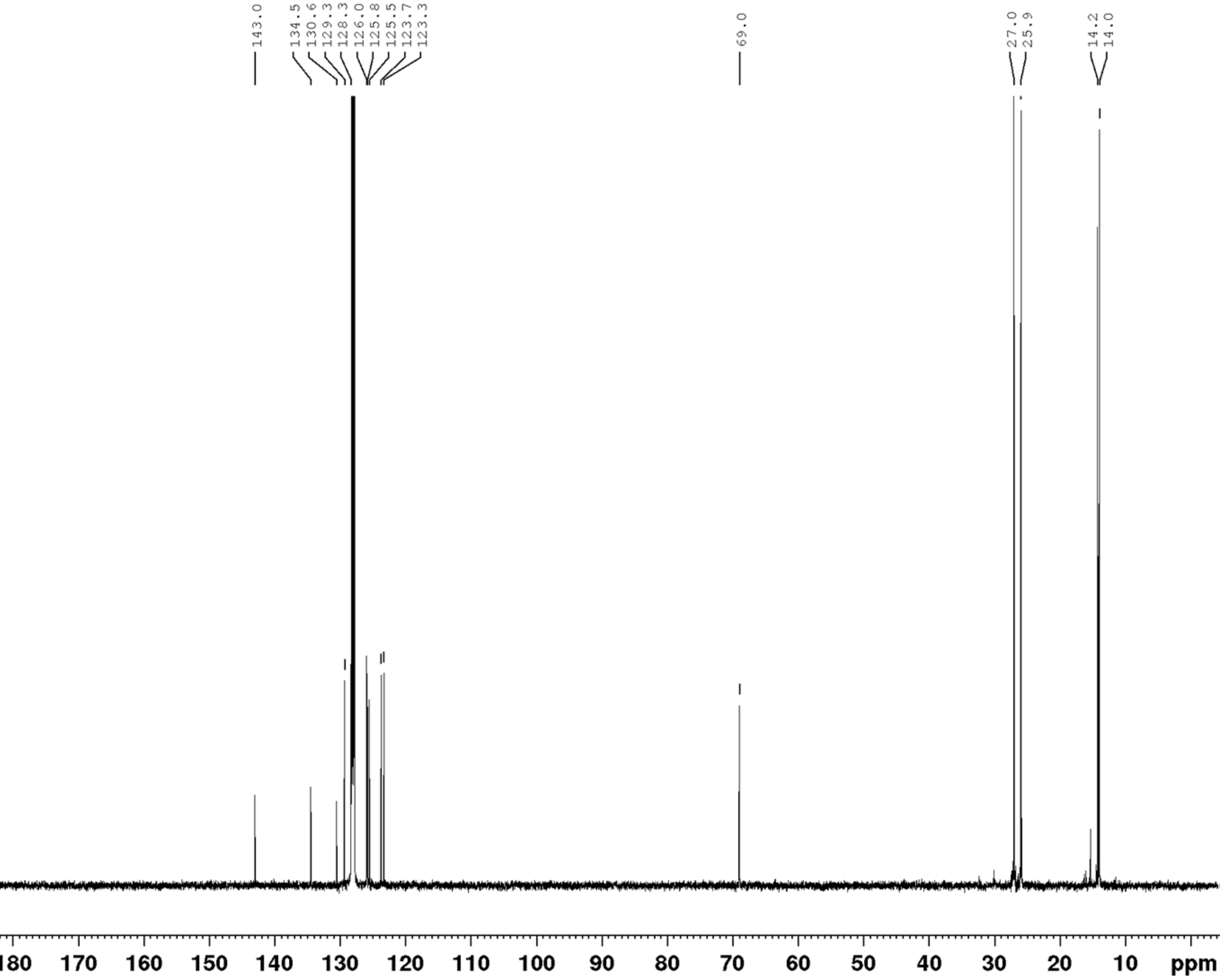

Supplementary Figure 193. ^{1}H NMR (500 MHz, $CDCl_3$) of (*R*)-2,3-Dihydro-1*H*-inden-1-ol [(*R*)-1r]

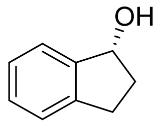

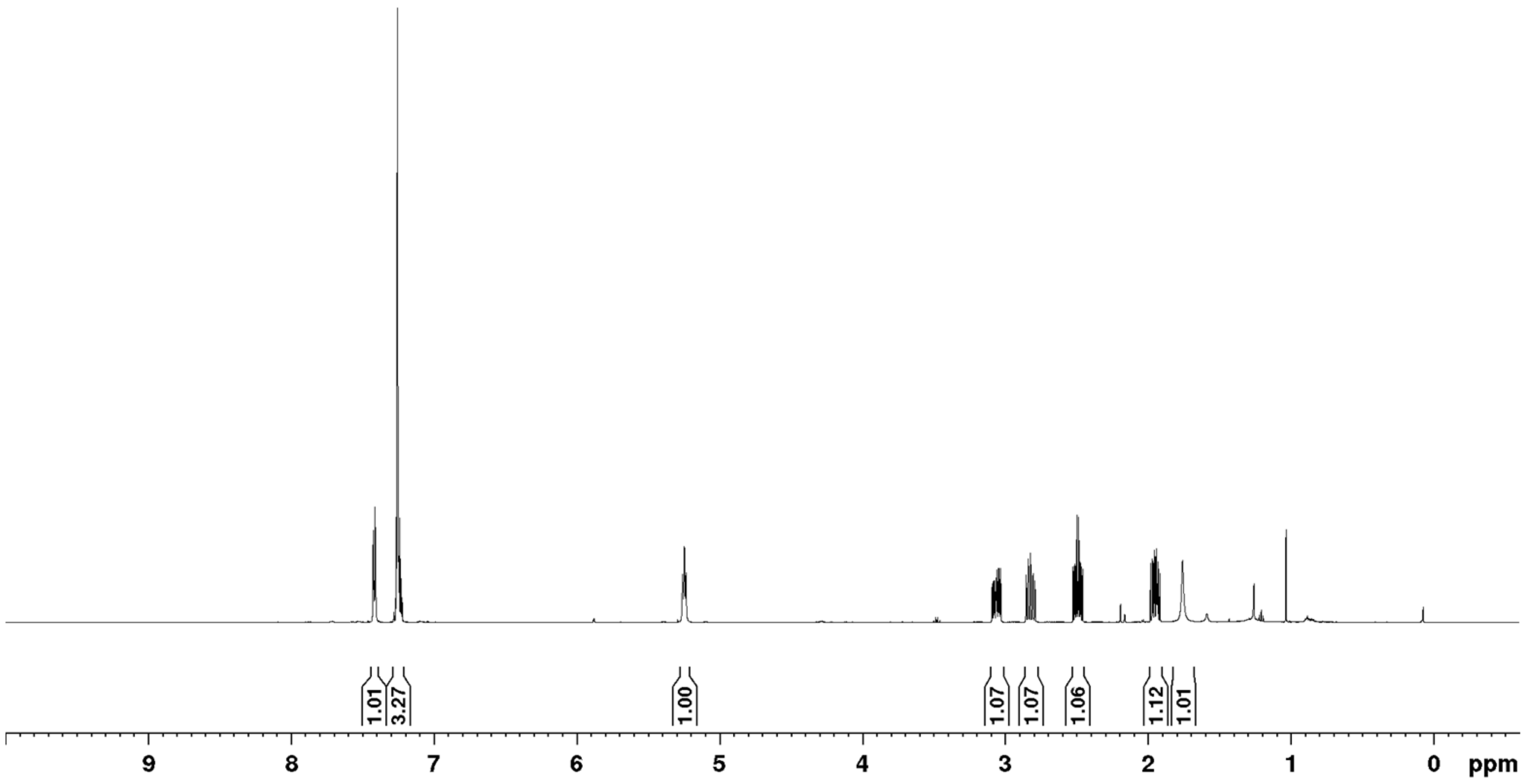

Supplementary Figure 194. ^{13}C NMR (126 MHz, $CDCl_3$) of (*R*)-2,3-Dihydro-1*H*-inden-1-ol [(*R*)-1r]

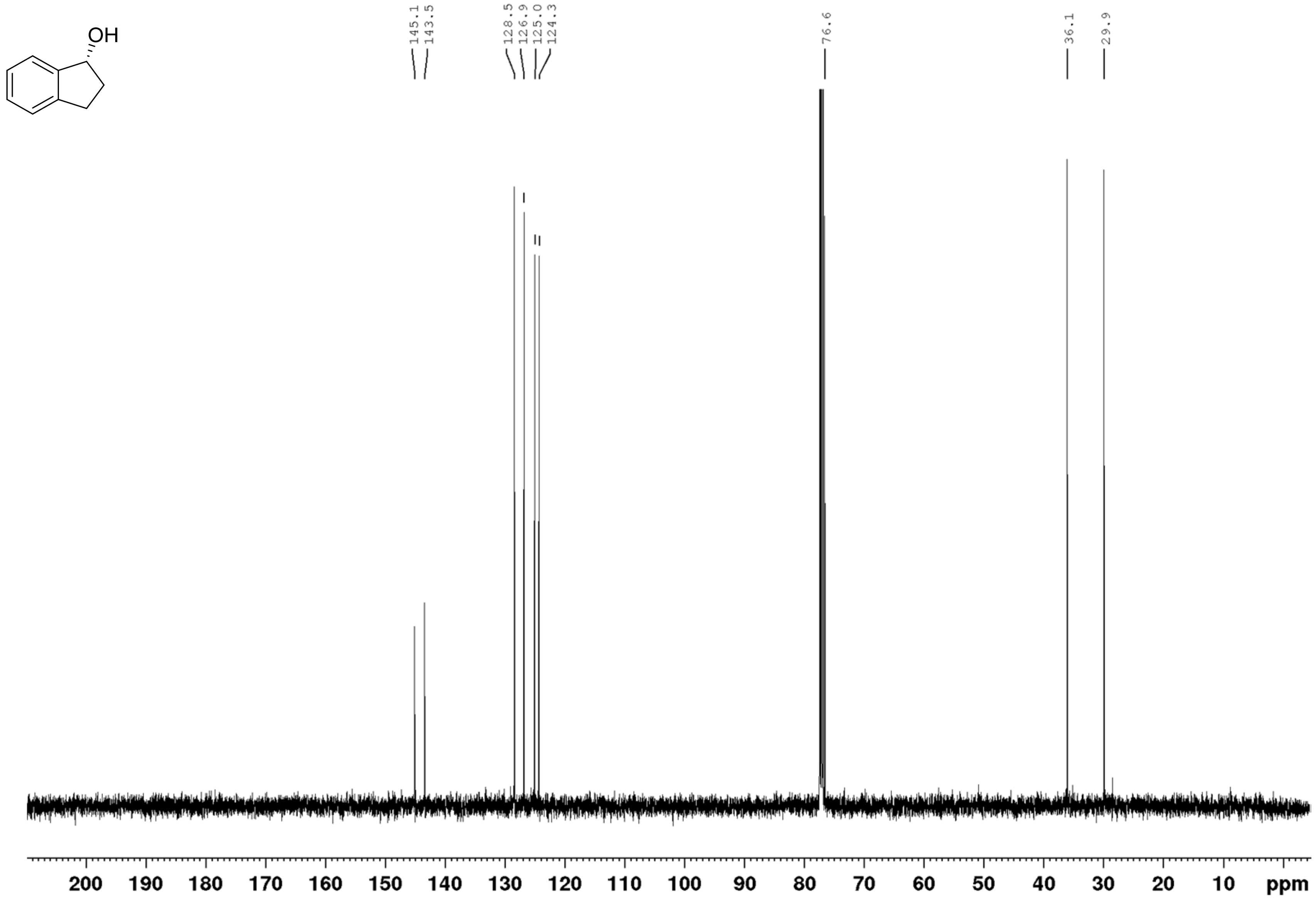

Supplementary Figure 195. ^{1}H NMR (500 MHz, C_6D_6) of (*S*)-Tributyl((2,3-dihydro-1*H*-inden-1-yl)oxy)silane [(*S*)-3rh]

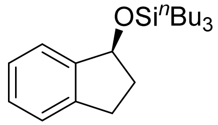

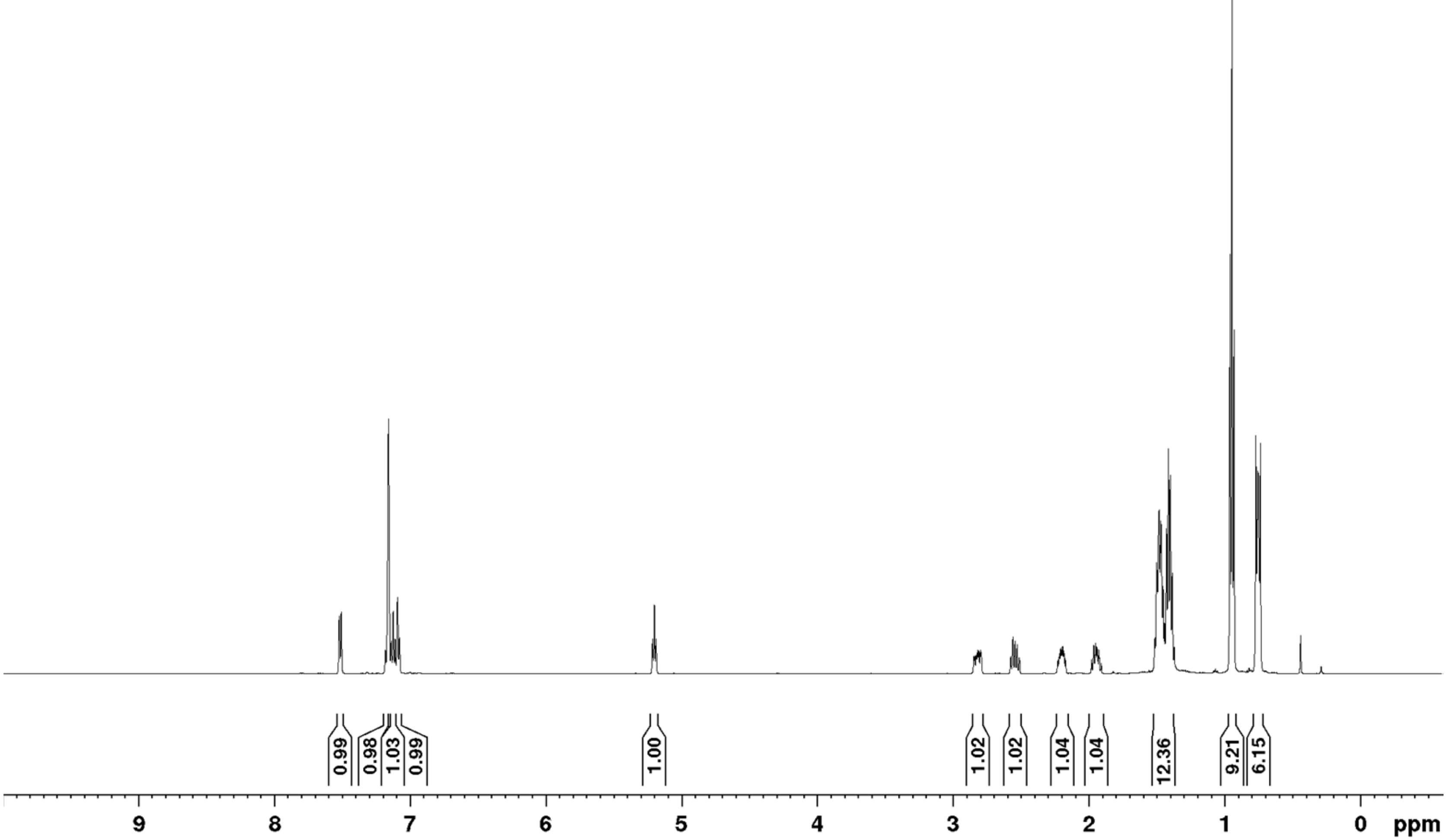

Supplementary Figure 196. ^{13}**C NMR (126 MHz,** C_6D_6**) of (*S*)-Tributyl((2,3-dihydro-1*H*-inden-1-yl)oxy)silane [(*S*)-3rh]**

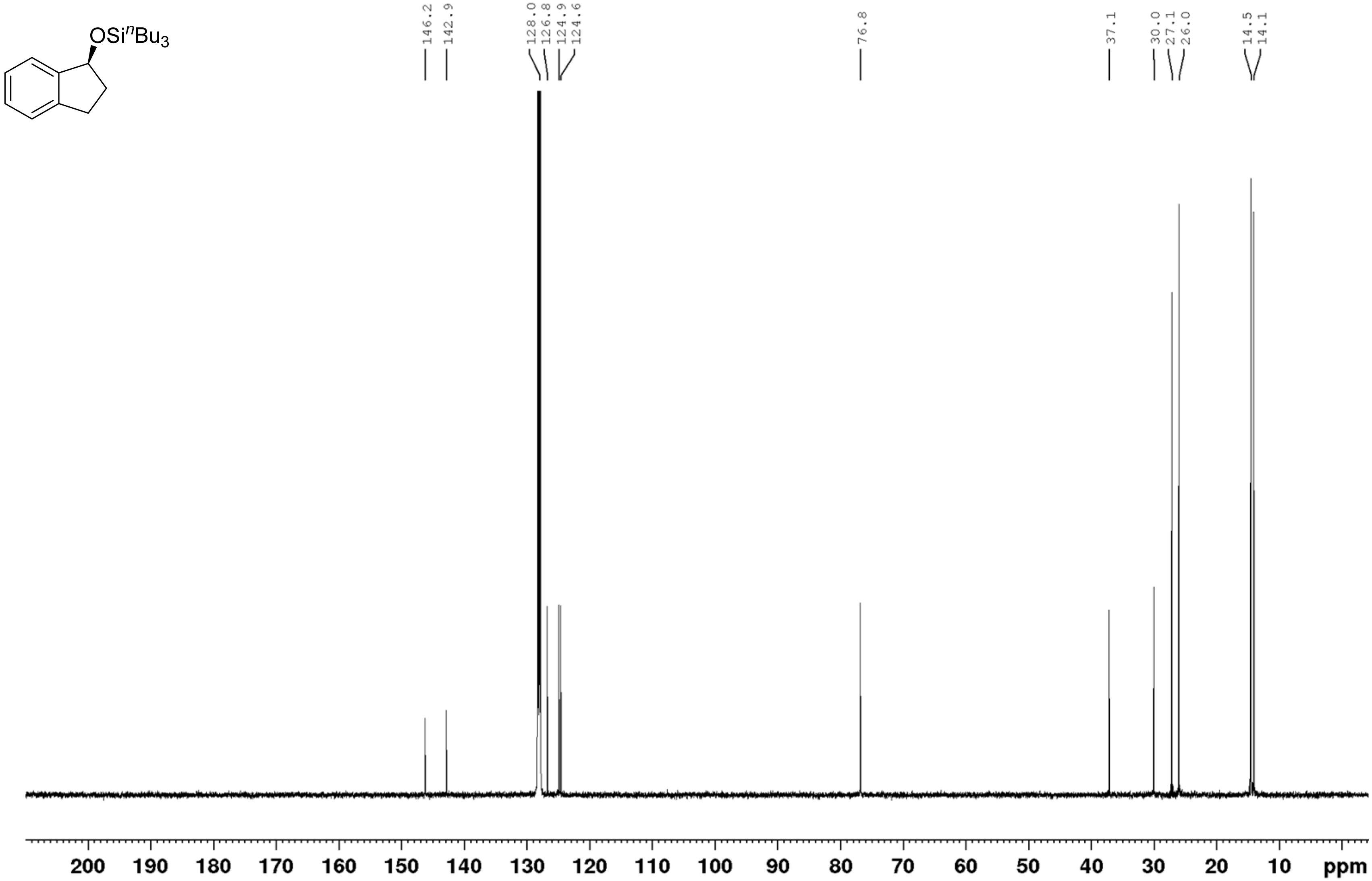

Supplementary Figure 197. ^{1}H **NMR (500 MHz,** C_6D_6**) of (*R*)-1,2,3,4-Tetrahydronaphthalen-1-ol [(*R*)-1s]**

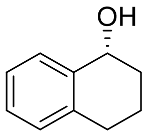

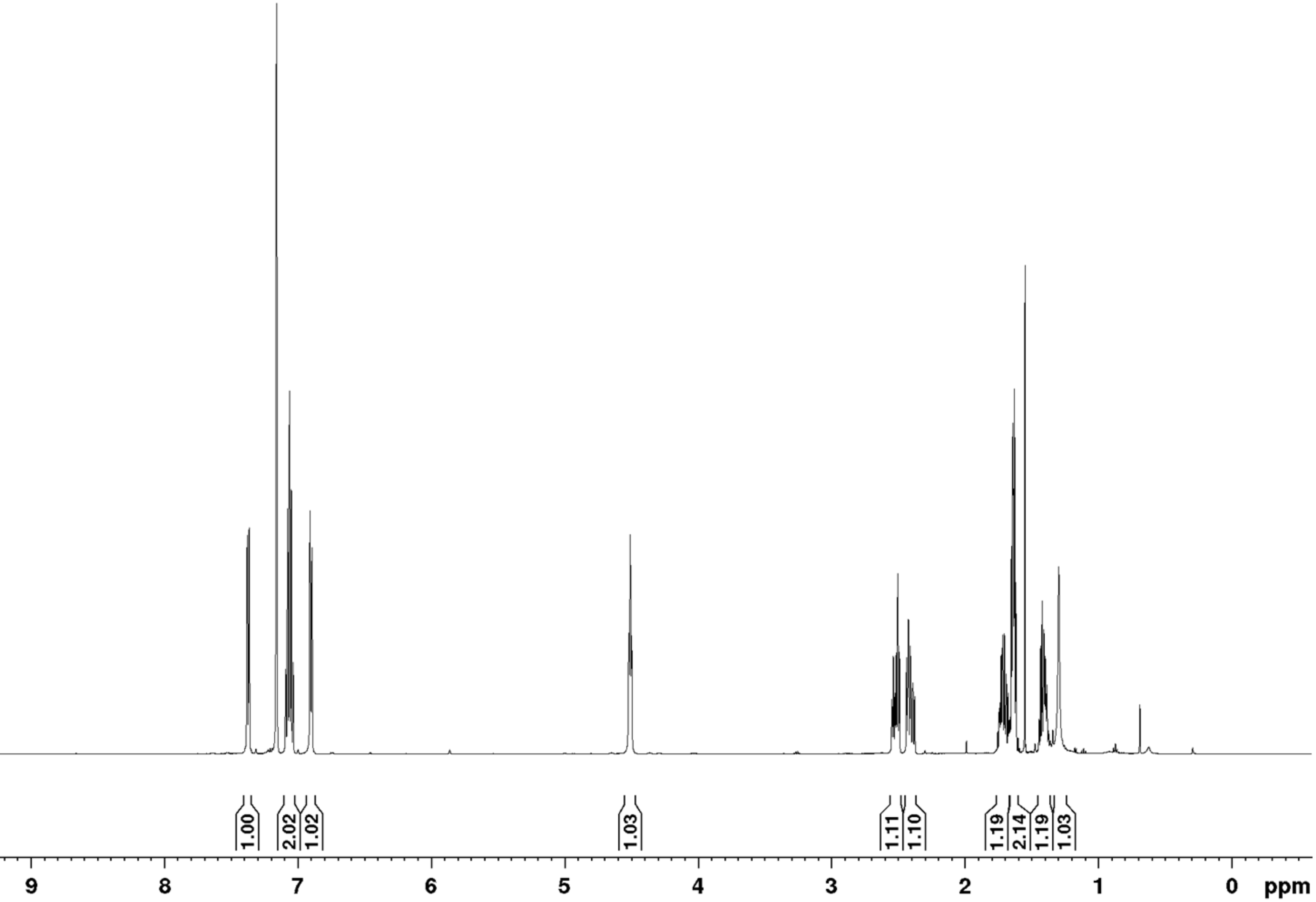

Supplementary Figure 198. ^{13}**C NMR (126 MHz,** C_6D_6**) of (*R*)-1,2,3,4-Tetrahydronaphthalen-1-ol [(*R*)-1s]**

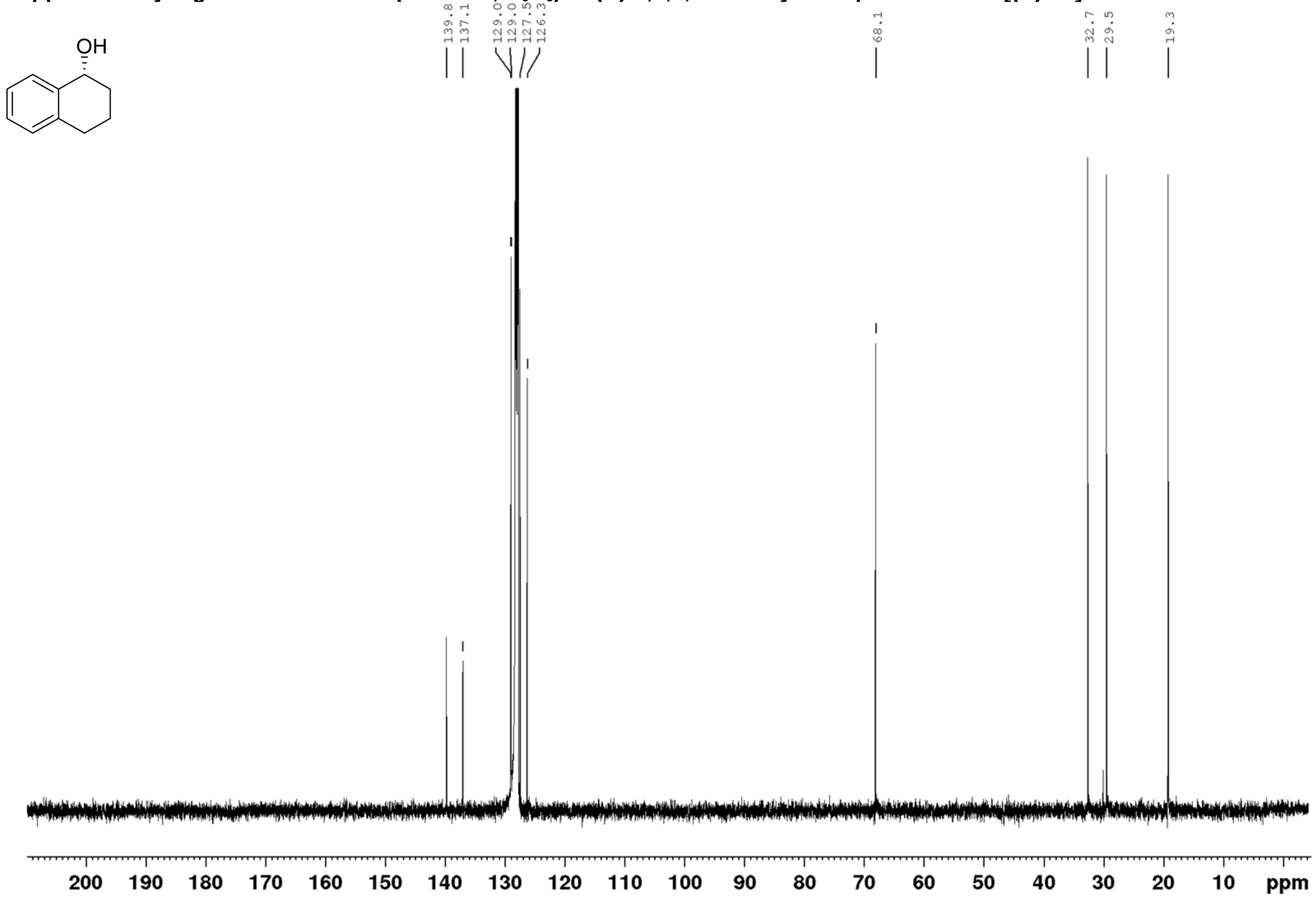

Supplementary Figure 199. **^{1}H NMR (500 MHz, C_6D_6) of (*S*)-Tributyl((1,2,3,4-tetrahydronaphthalen-1-yl)oxy)silane [(*S*)-3sh]**

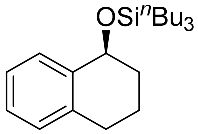

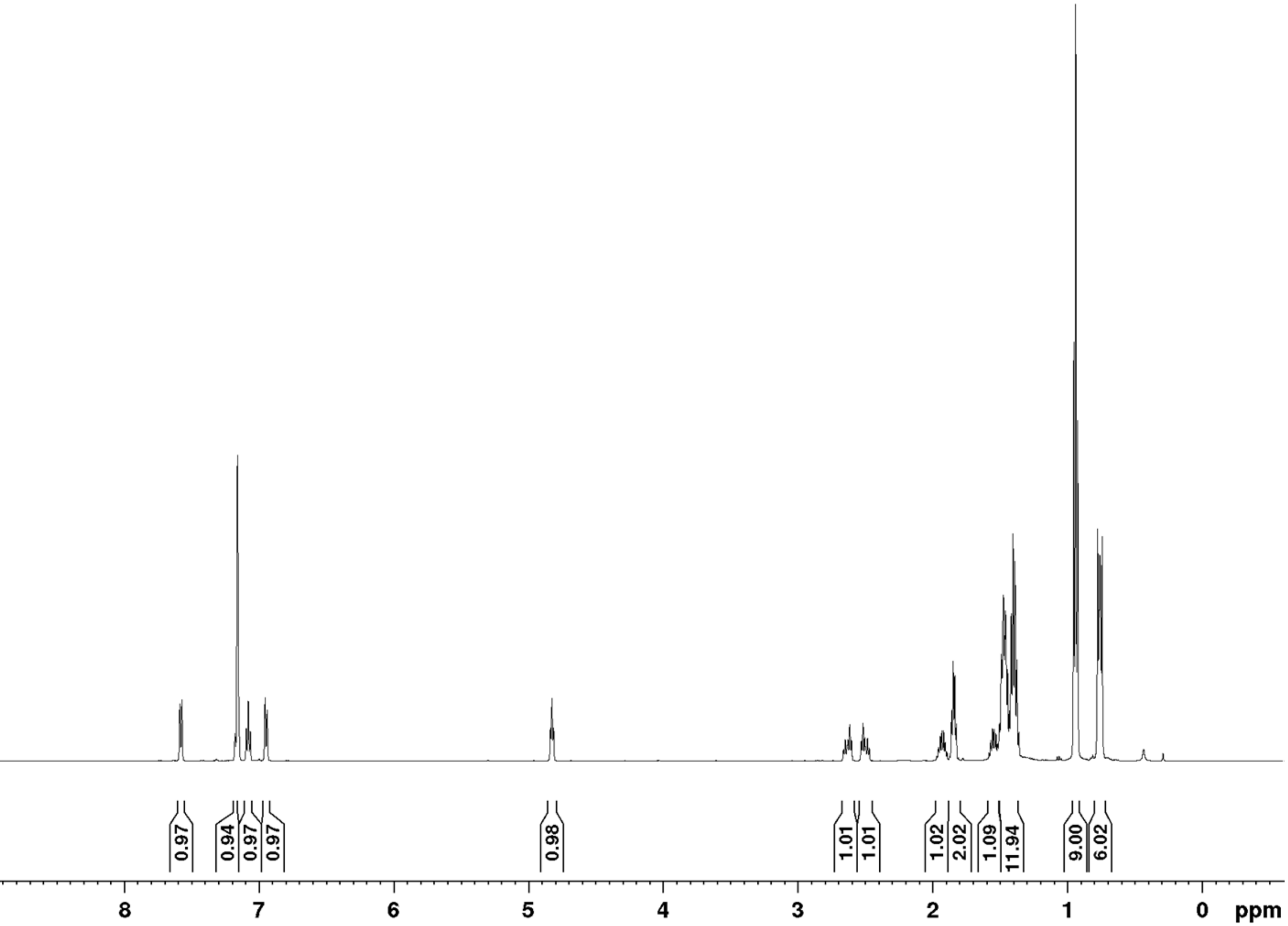

Supplementary Figure 200. ^{13}C NMR (126 MHz, C_6D_6) of (*S*)-Tributyl((1,2,3,4-tetrahydronaphthalen-1-yl)oxy)silane [(*S*)-3sh]

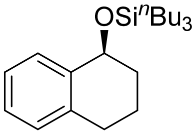

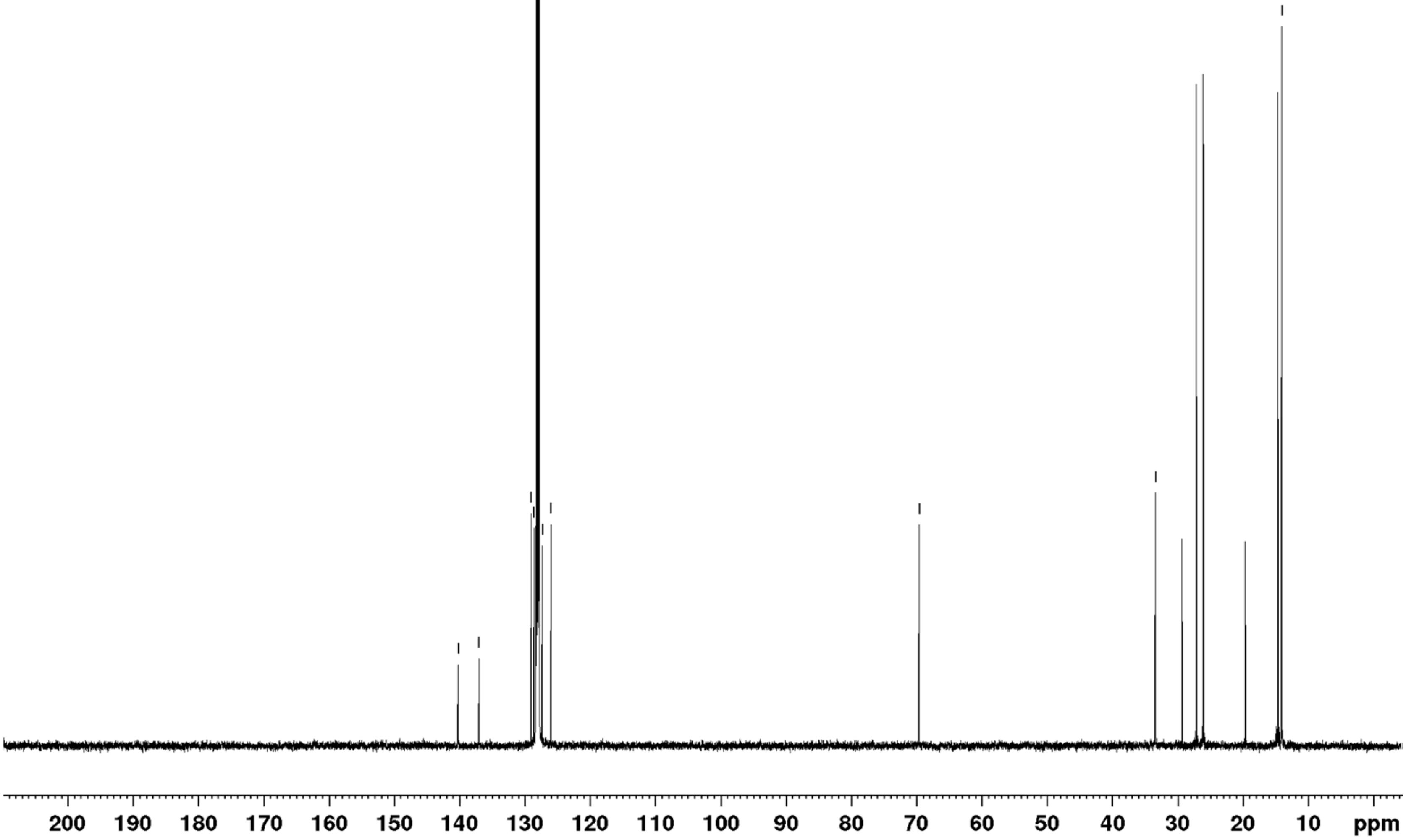

Supplementary Figure 201. ^{1}H NMR (500 MHz, $CDCl_3$) of (*R*)-6,7,8,9-Tetrahydro-5*H*-benzo[7]annulen-5-ol [(*R*)-1t]

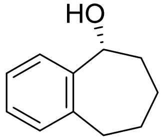

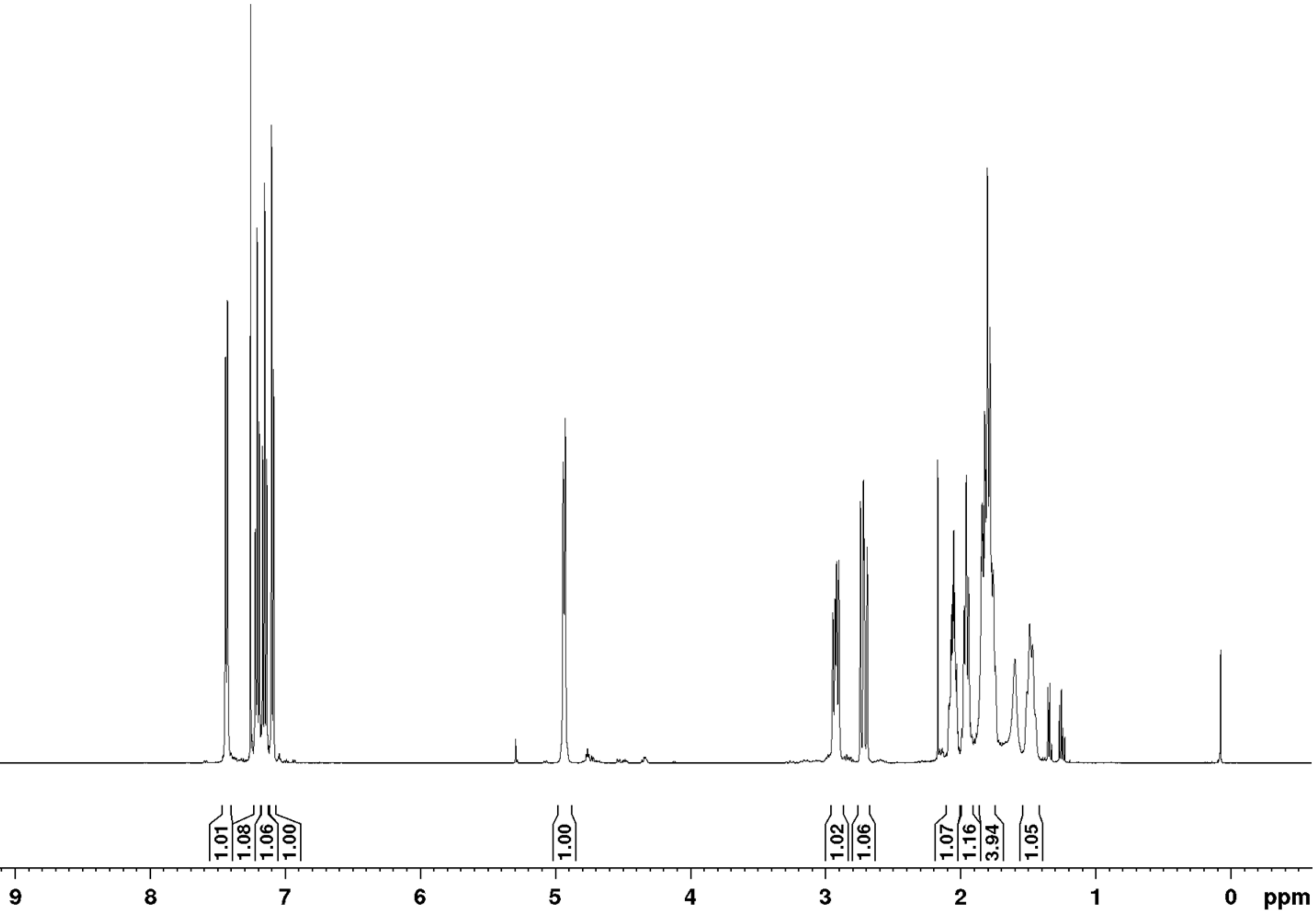

Supplementary Figure 202. ^{13}**C NMR (126 MHz, CDCl$_3$) of (*R*)-6,7,8,9-Tetrahydro-5*H*-benzo[7]annulen-5-ol [(*R*)-1t]**

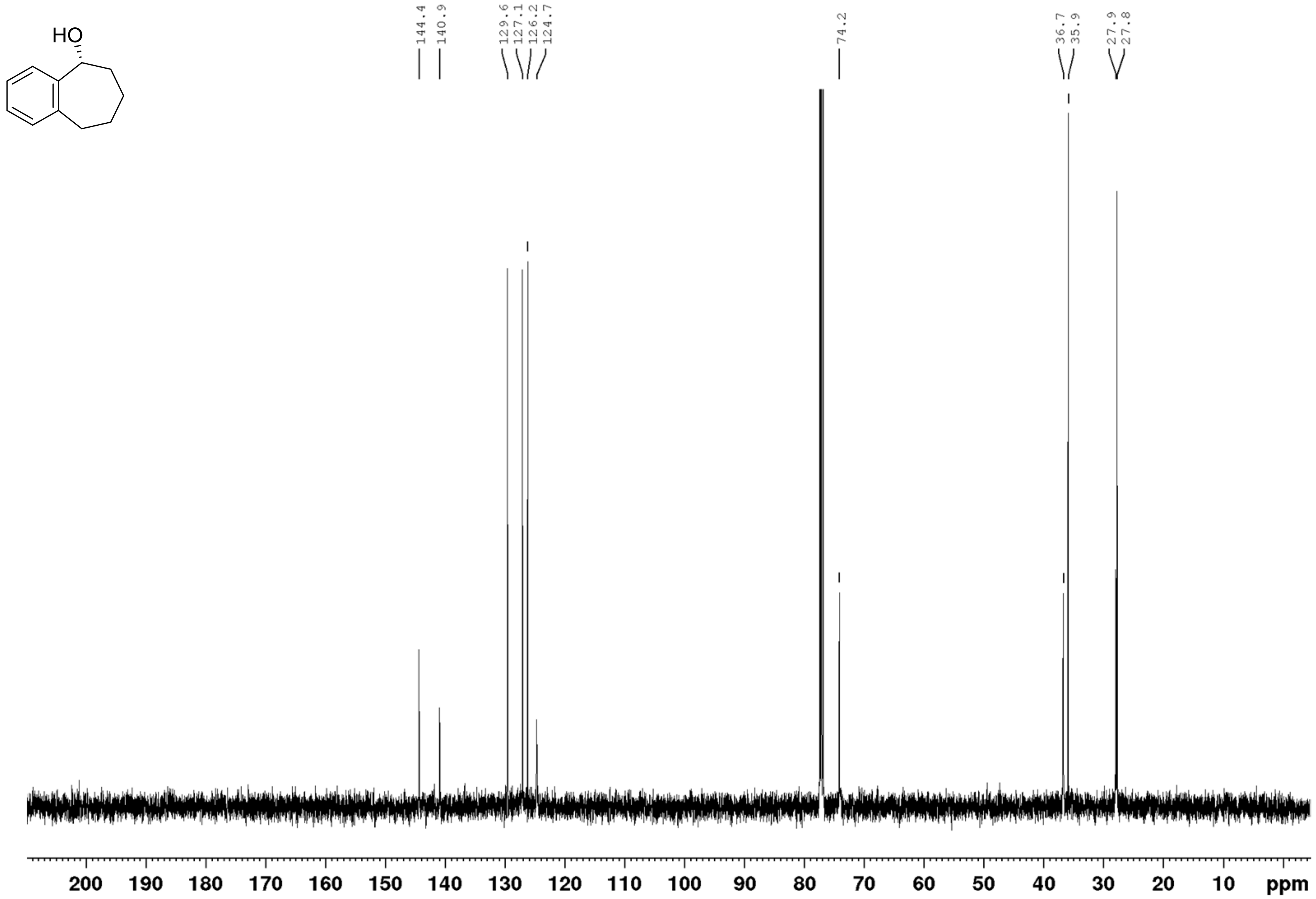

Supplementary Figure 203. **^{1}H NMR (500 MHz, C_6D_6) of (*S*)-Tributyl((6,7,8,9-tetrahydro-5*H*-benzo[7]annulen-5-yl)oxy)silane [(*S*)-3th]**

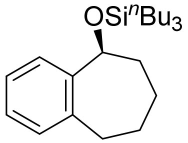

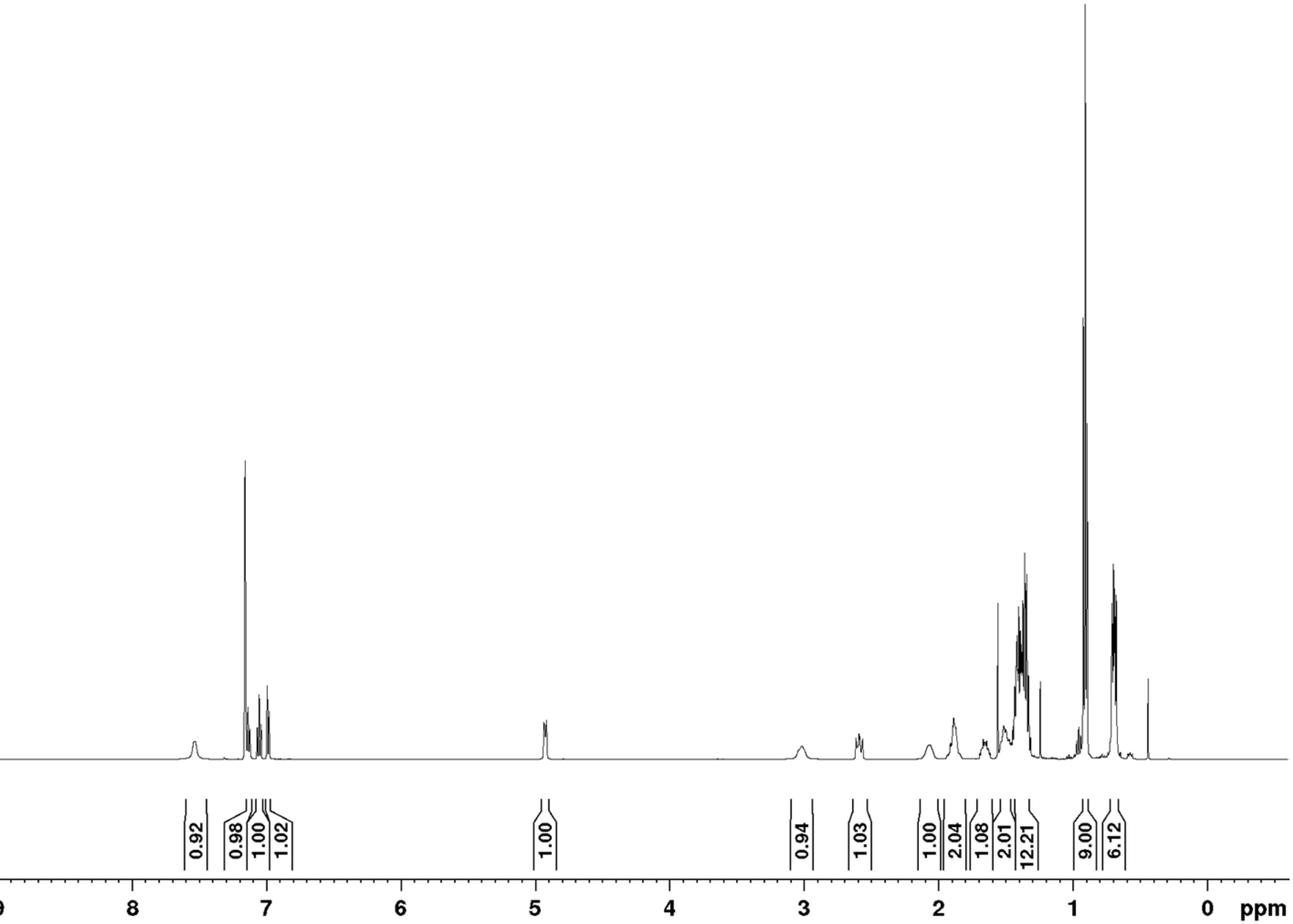

Supplementary Figure 204. ^{13}**C NMR (126 MHz,** C_6D_6**) of (*S*)-Tributyl((6,7,8,9-tetrahydro-5*H*-benzo[7]annulen-5-yl)oxy)silane [(*S*)-3th]**

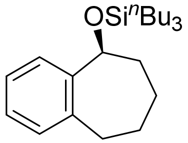

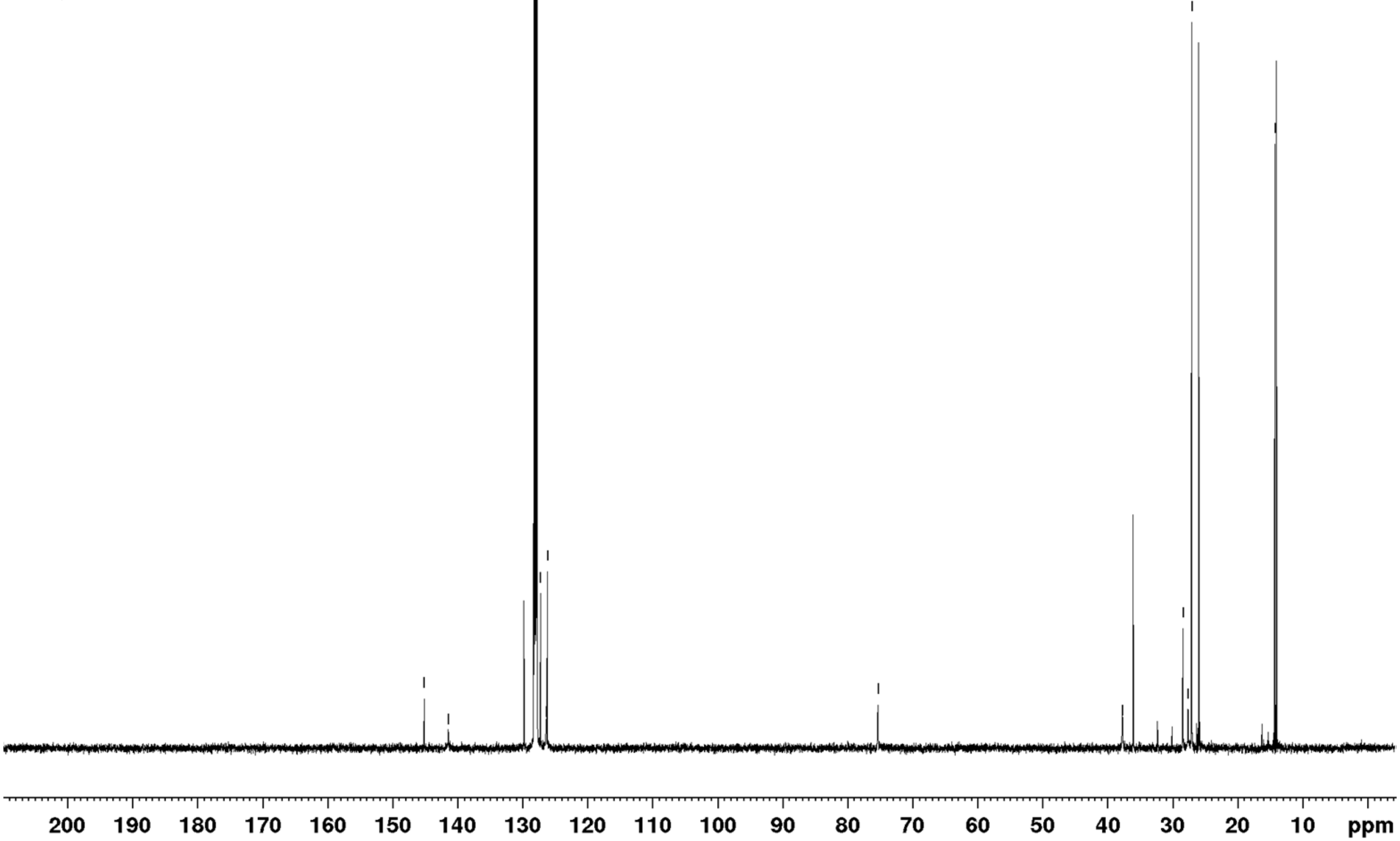

Supplementary Figure 205. ^{1}H NMR (500 MHz, $CDCl_3$) of (*R*)-Chroman-4-ol [(*R*)-1u]

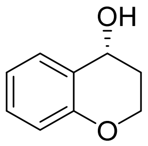

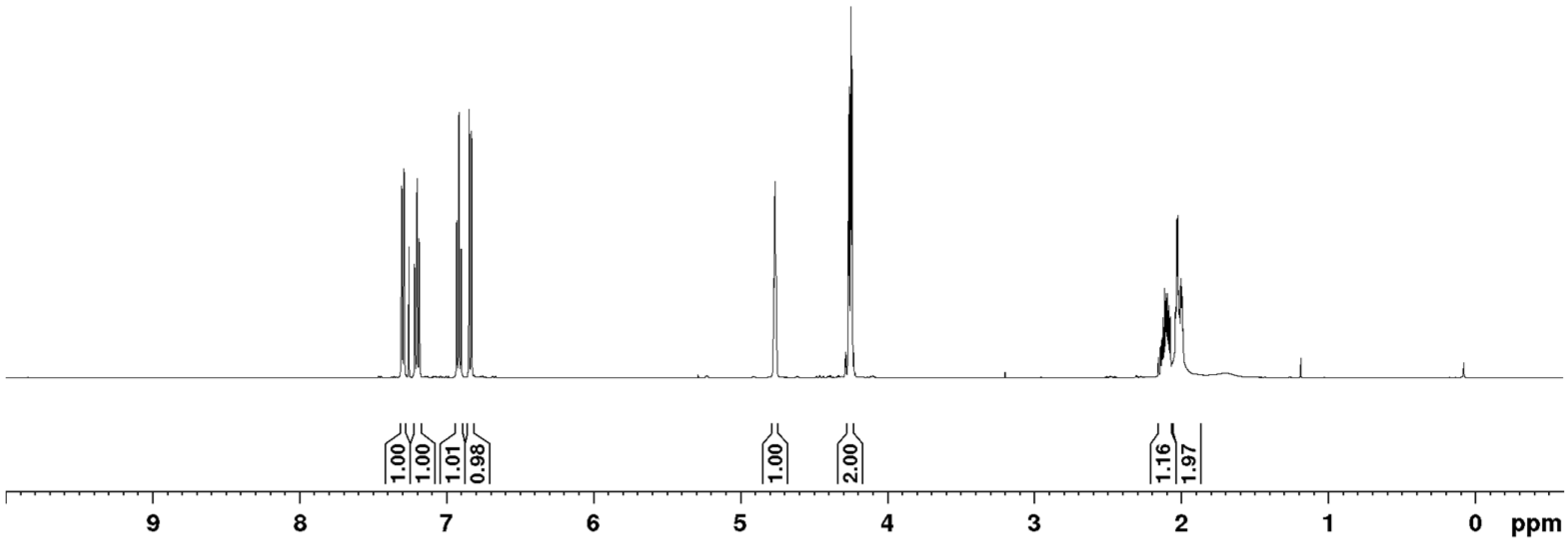

Supplementary Figure 206. ^{13}**C NMR (126 MHz, CDCl$_3$) of (*R*)-Chroman-4-ol [(*R*)-1u]**

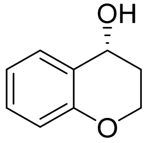

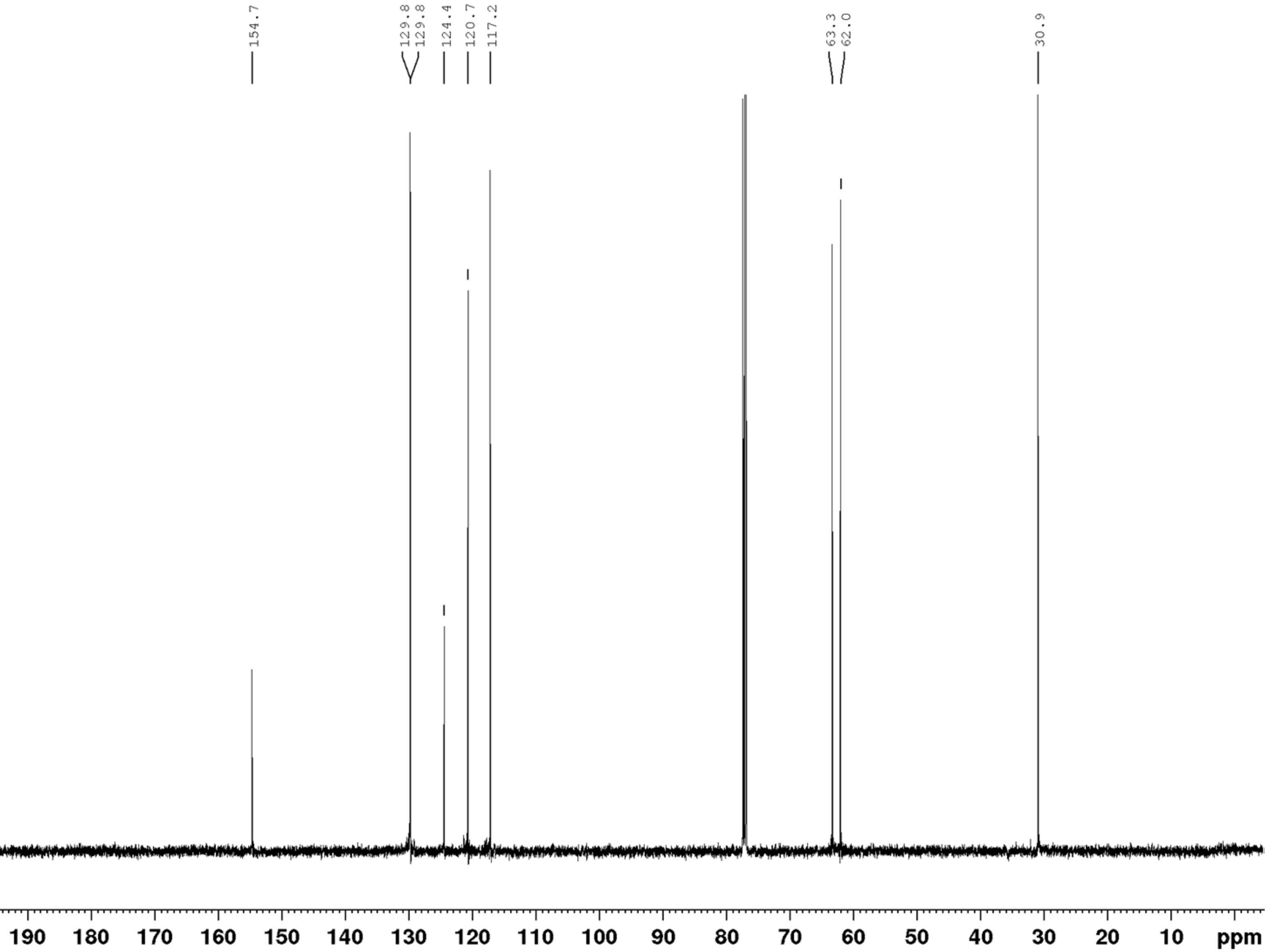

Supplementary Figure 207. **^{1}H NMR (500 MHz, C_6D_6) of (*S*)-Tributyl(chroman-4-yloxy)silane [(*S*)-3uh]**

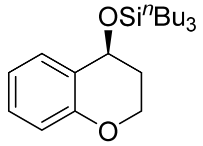

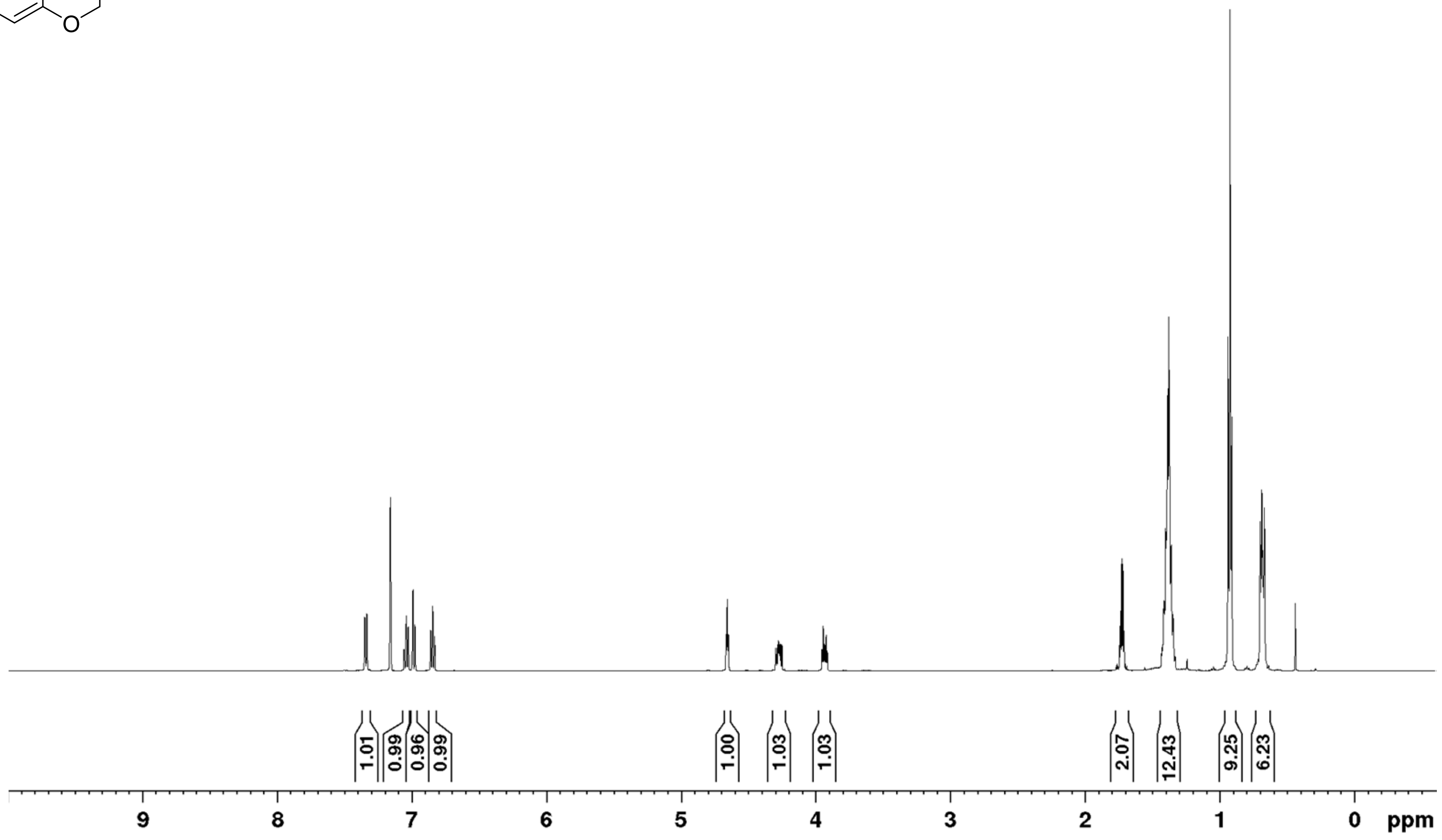

Supplementary Figure 208. ^{13}C NMR (126 MHz, C_6D_6) of (*S*)-Tributyl(chroman-4-yloxy)silane [(*S*)-3uh]

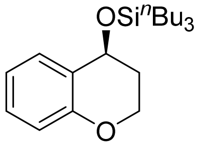

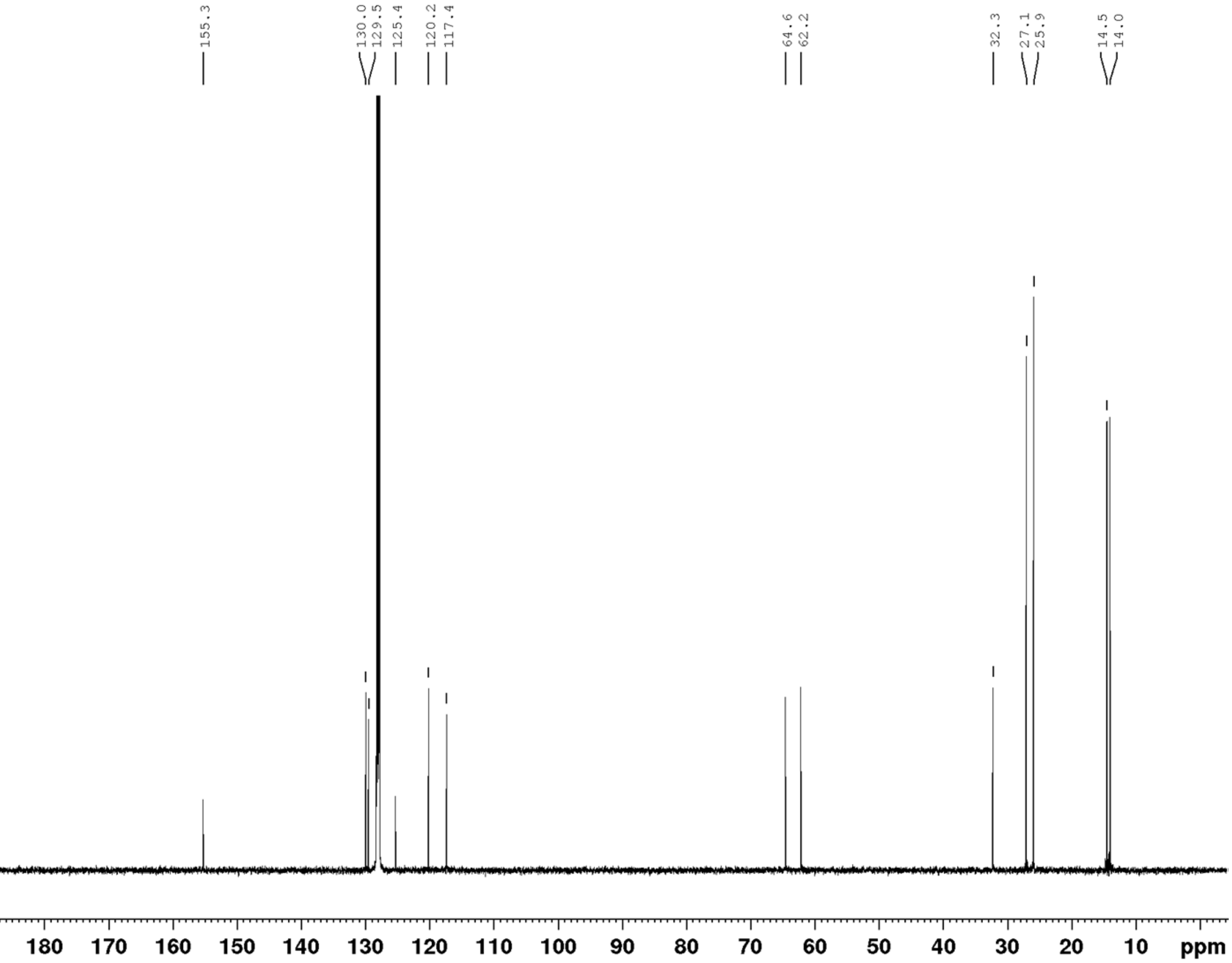

Supplementary Figure 209. ^{1}H NMR (400 MHz, $CDCl_3$) of (*R*)-Thiochroman-4-ol [(*R*)-1v]

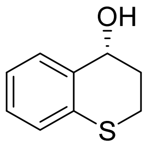

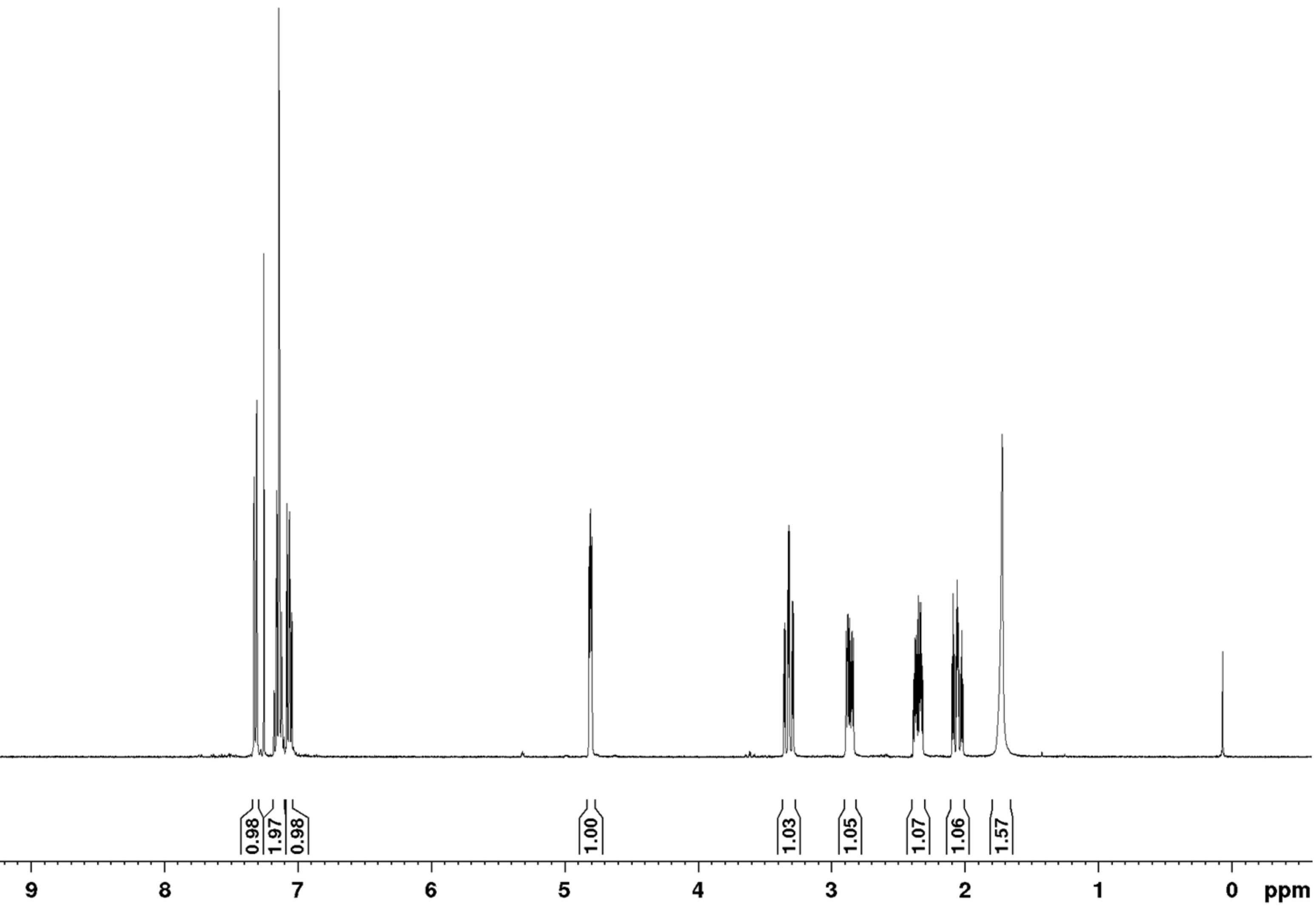

Supplementary Figure 210. ^{13}**C NMR (126 MHz, CDCl$_3$) of (*R*)-Thiochroman-4-ol [(*R*)-1v]**

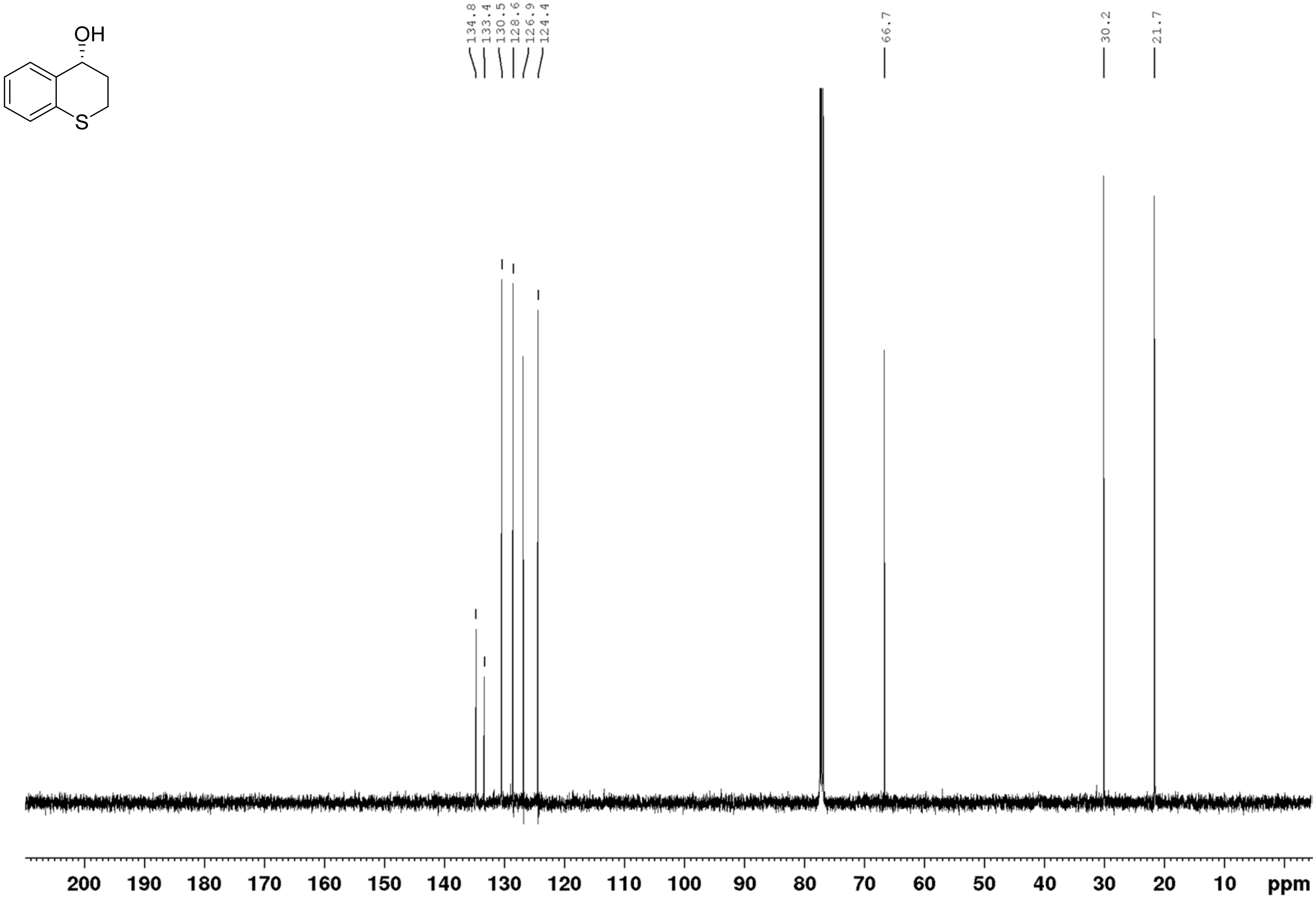

Supplementary Figure 211. ^{1}H NMR (500 MHz, C_6D_6) of (*S*)-Tributyl(thiochroman-4-yloxy)silane [(*S*)-3vh]

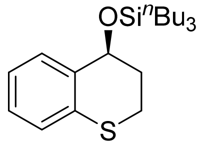

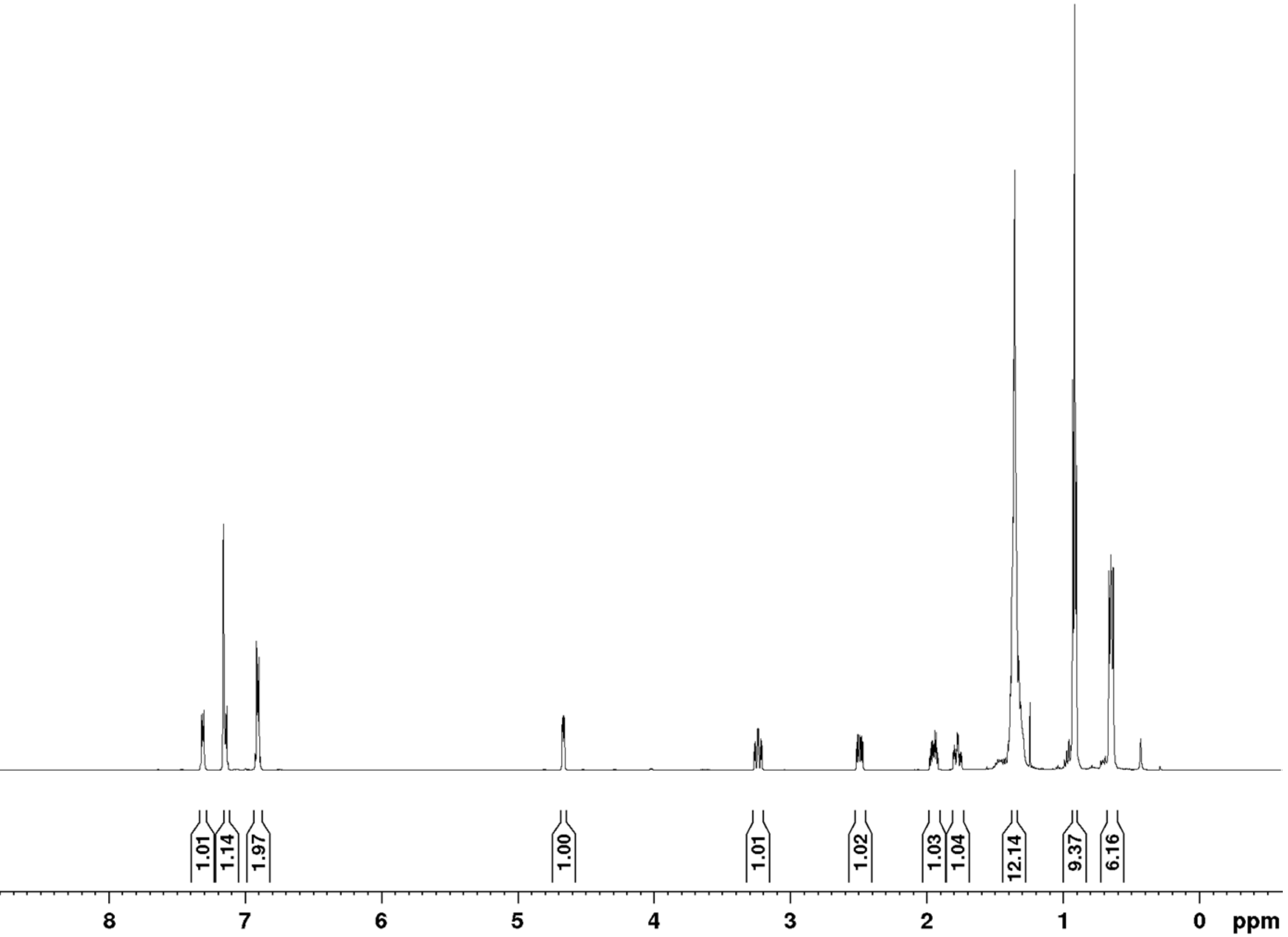

Supplementary Figure 212. ^{13}**C NMR (126 MHz, C_6D_6) of (*S*)-Tributyl(thiochroman-4-yloxy)silane [(*S*)-3vh]**

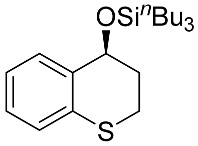

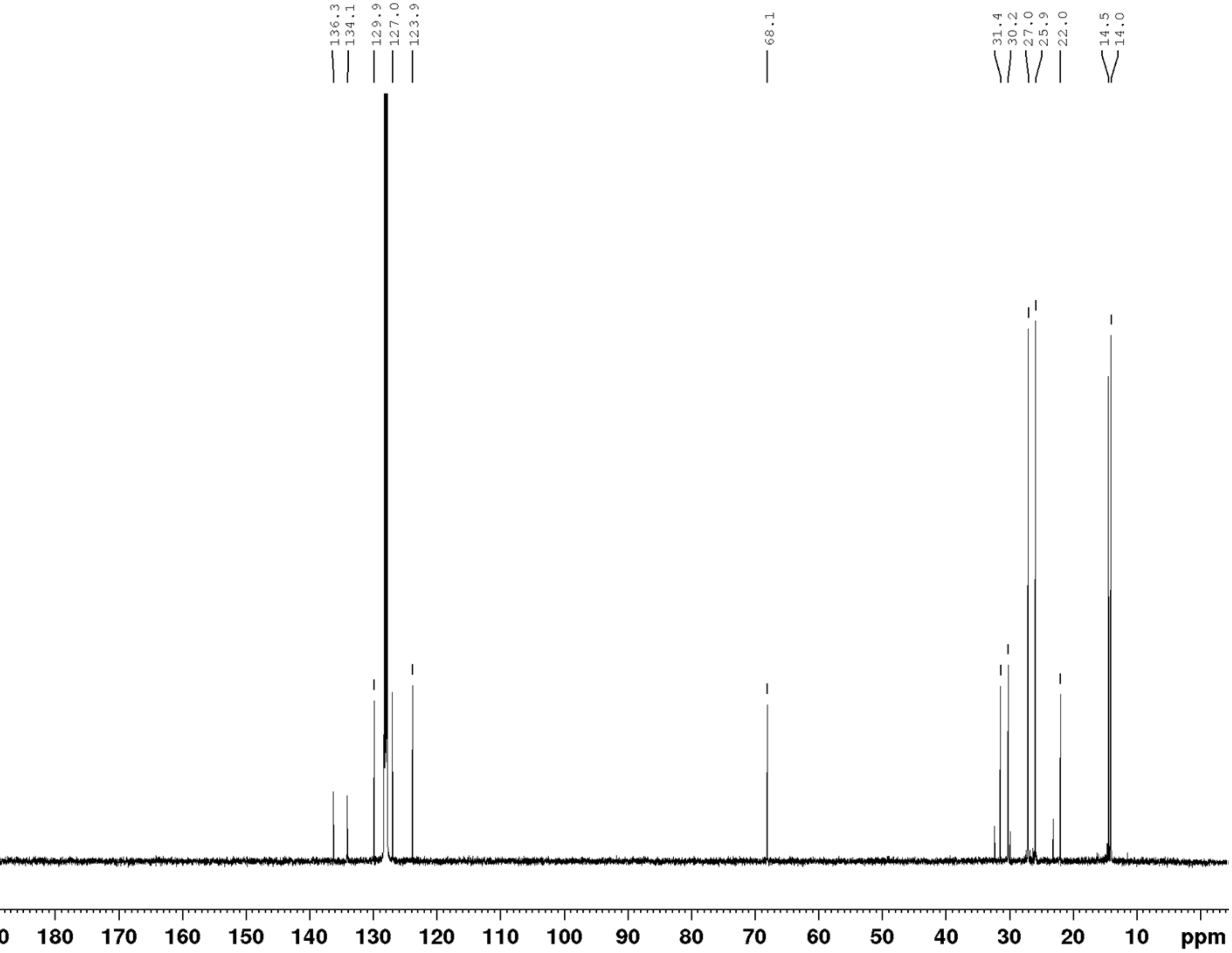

Supplementary Figure 213. ^{1}H NMR (400 MHz, $CDCl_3$) of (*R*)-*tert*-Butyl 4-hydroxy-3,4-dihydroquinoline-1(2*H*)-carboxylate [(*R*)-1w]

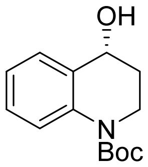

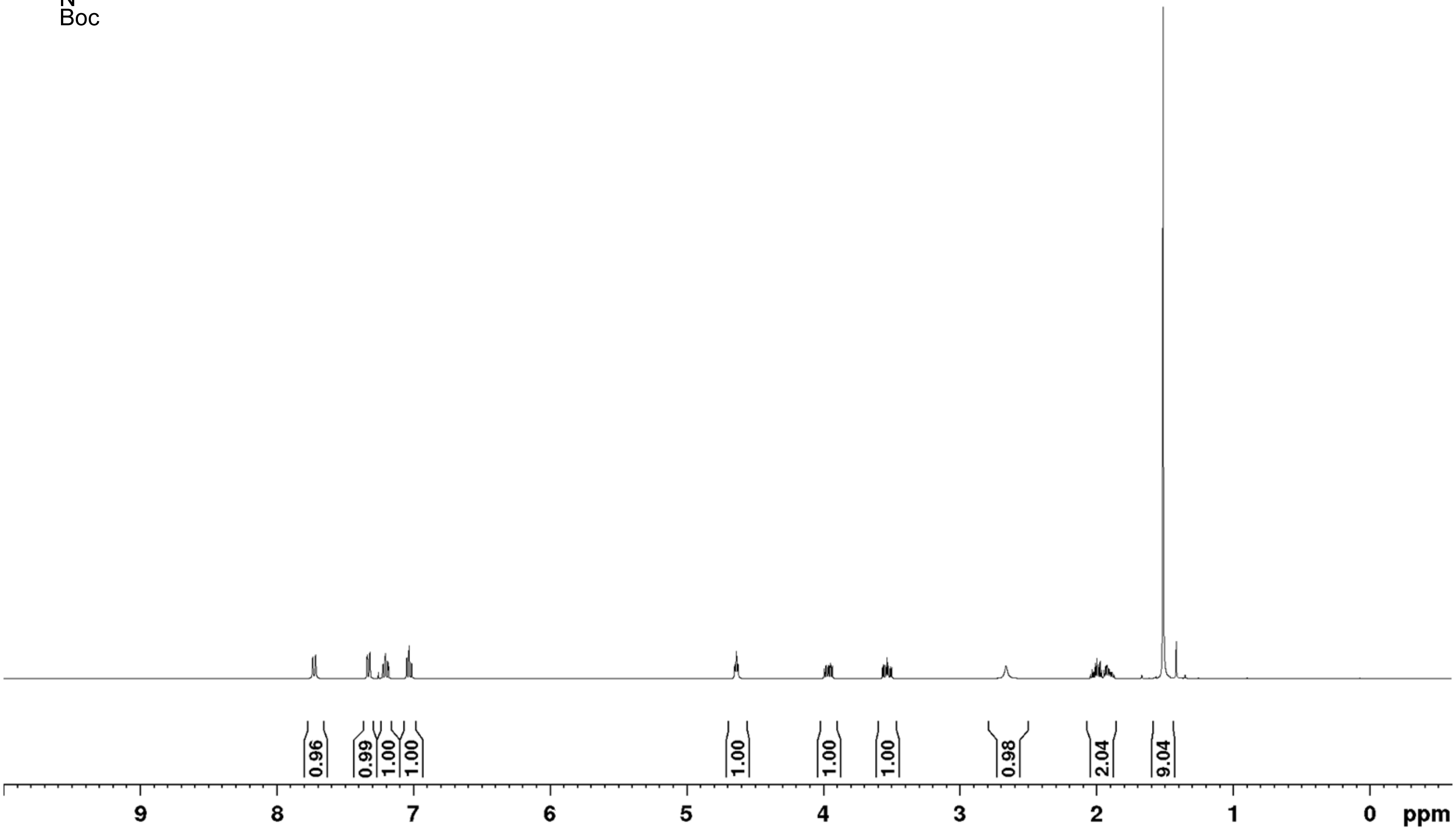

Supplementary Figure 214. ^{13}C NMR (101 MHz, $CDCl_3$) of (*R*)-*tert*-Butyl 4-hydroxy-3,4-dihydroquinoline-1(2*H*)-carboxylate [(*R*)-1w]

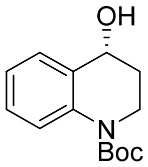

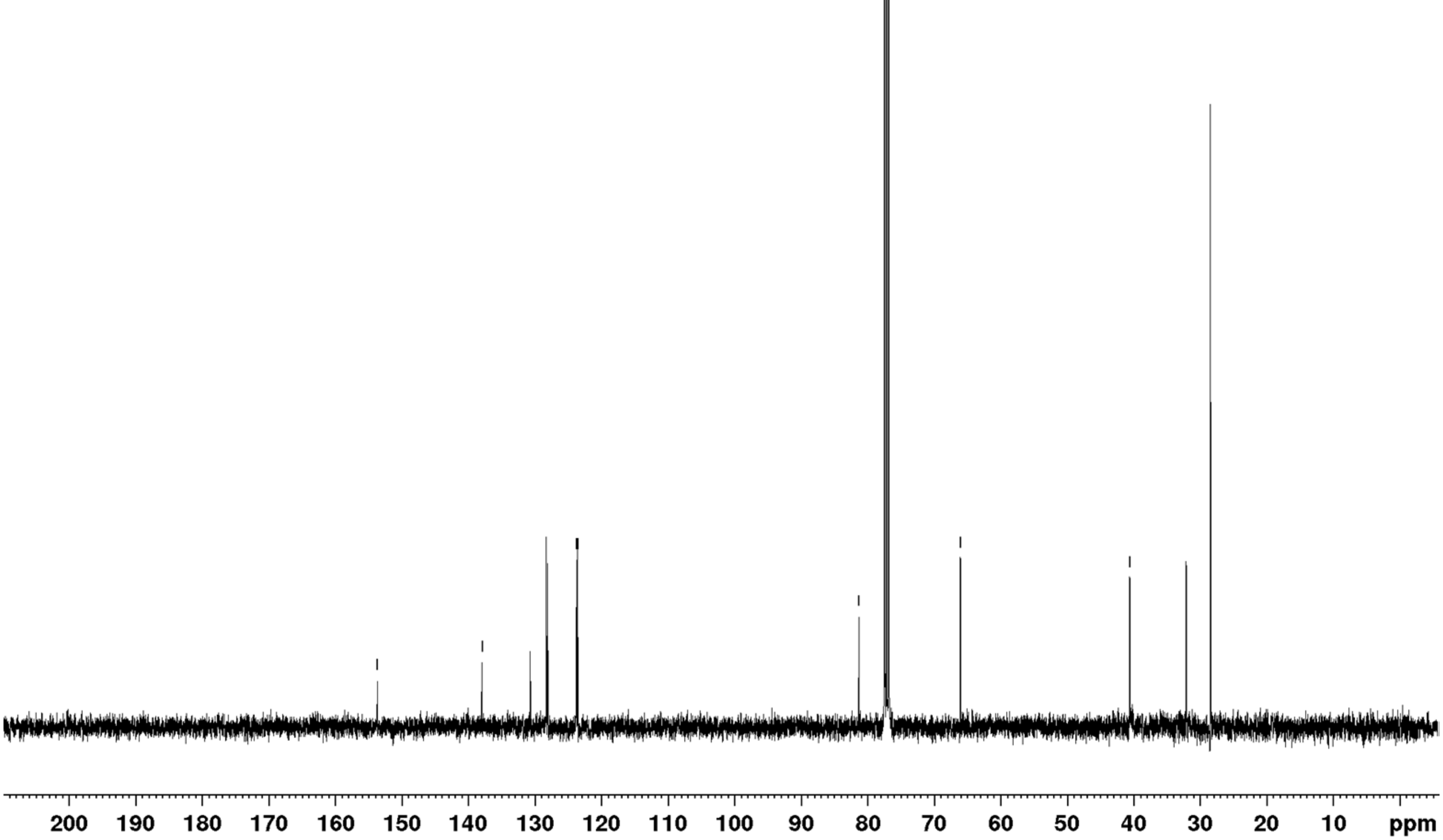

Supplementary Figure 215. **^{1}H NMR (500 MHz, C_6D_6) of (*S*)-*tert*-Butyl 4-((tributylsilyl)oxy)-3,4-dihydroquinoline-1(2*H*)-carboxylate [(*S*)-3wh]**

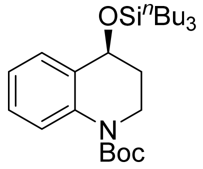

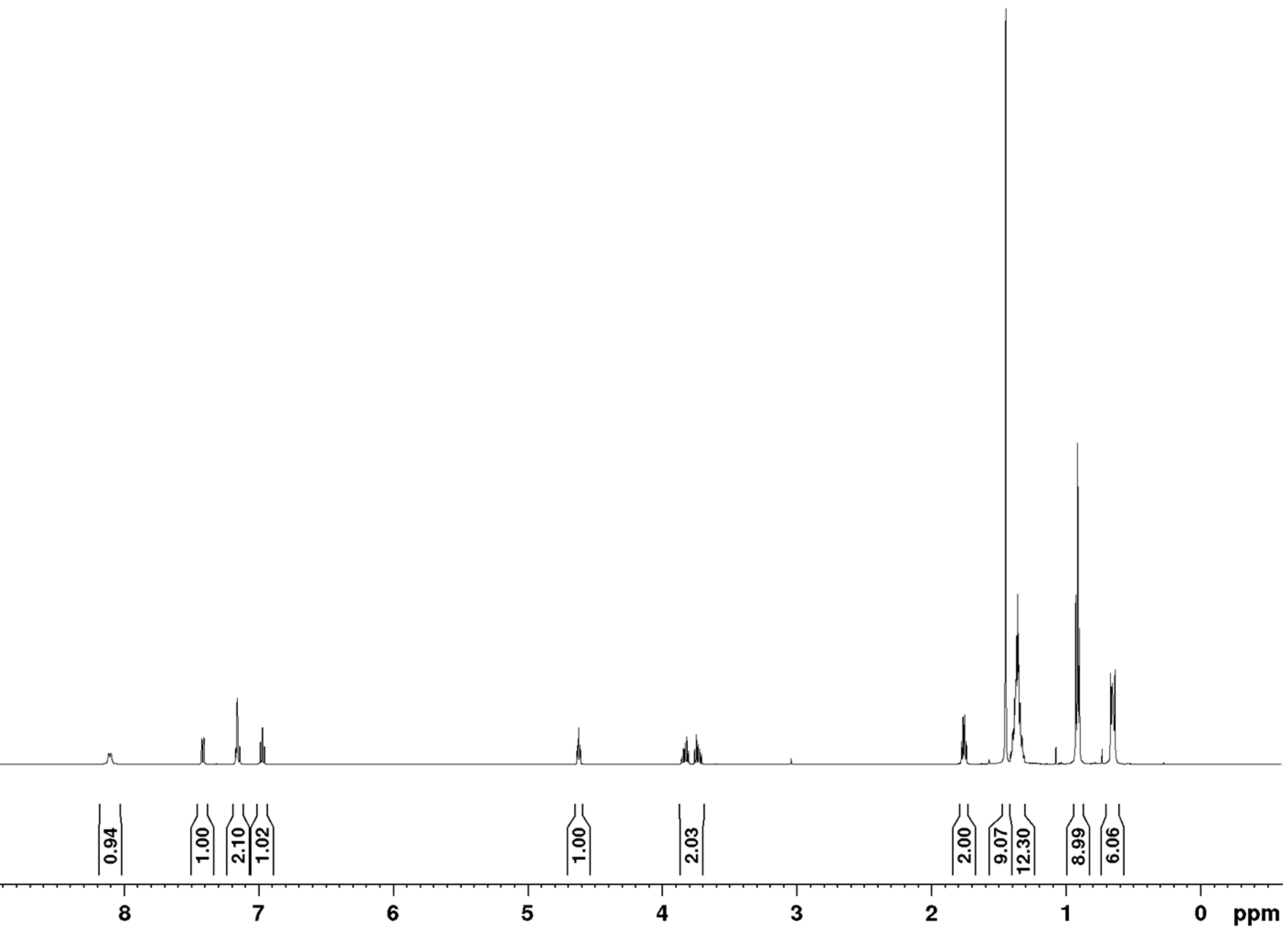

Supplementary Figure 216. **^{13}C NMR (126 MHz, C_6D_6) of (*S*)-*tert*-Butyl 4-((tributylsilyl)oxy)-3,4-dihydroquinoline-1(2*H*)-carboxylate [(*S*)-3wh]**

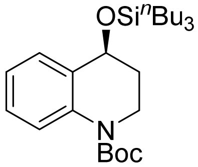

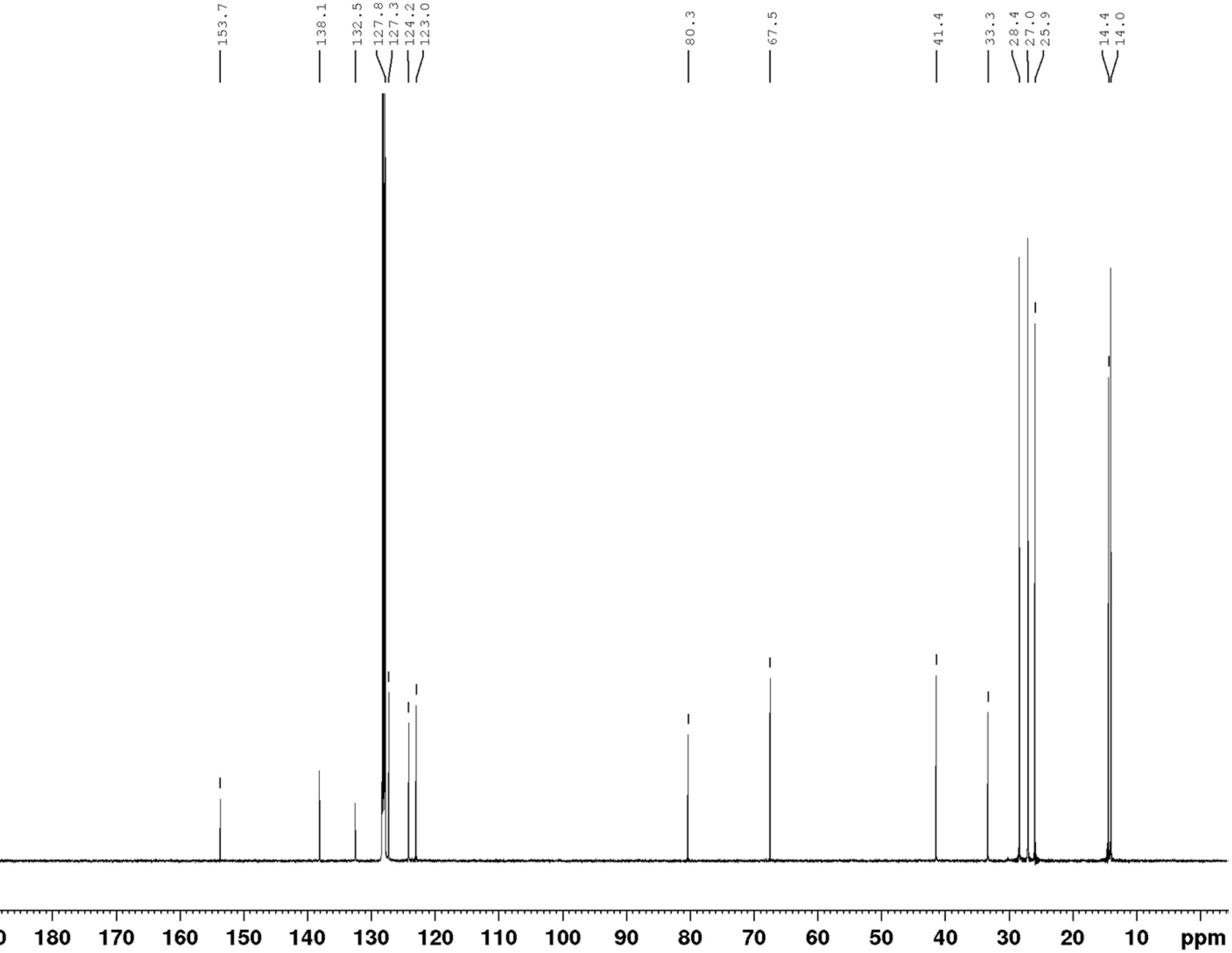

Supplementary Figure 217. ^{1}**H NMR (500 MHz,** C_6D_6**) of (1*R*,2*S*)-2-Phenylcyclohexan-1-ol [(1*R*,2*S*)-4]**

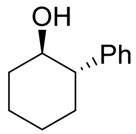

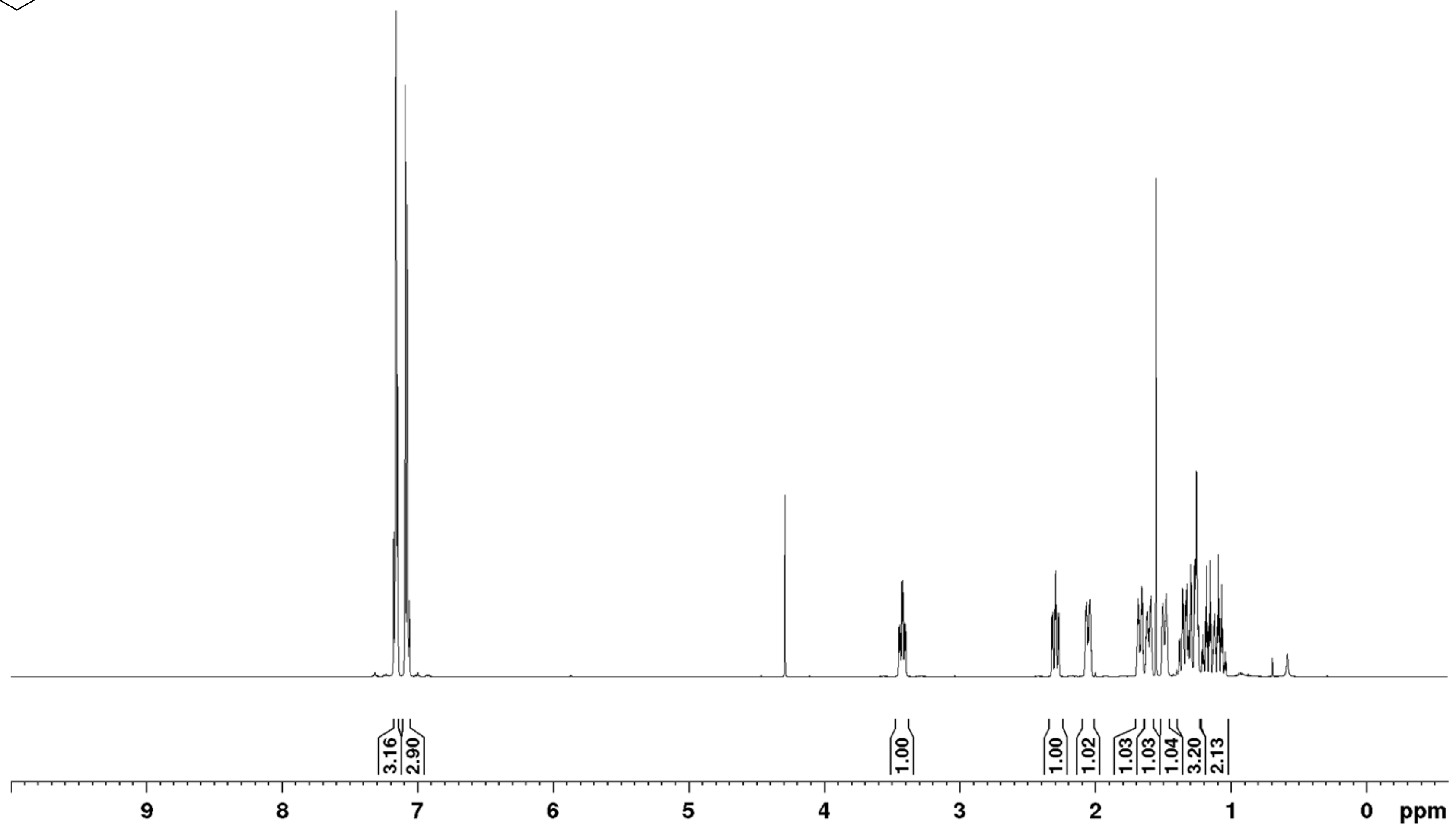

Supplementary Figure 218. ^{13}**C NMR (126 MHz,** C_6D_6**) of (1*R*,2*S*)-2-Phenylcyclohexan-1-ol [(1*R*,2*S*)-4]**

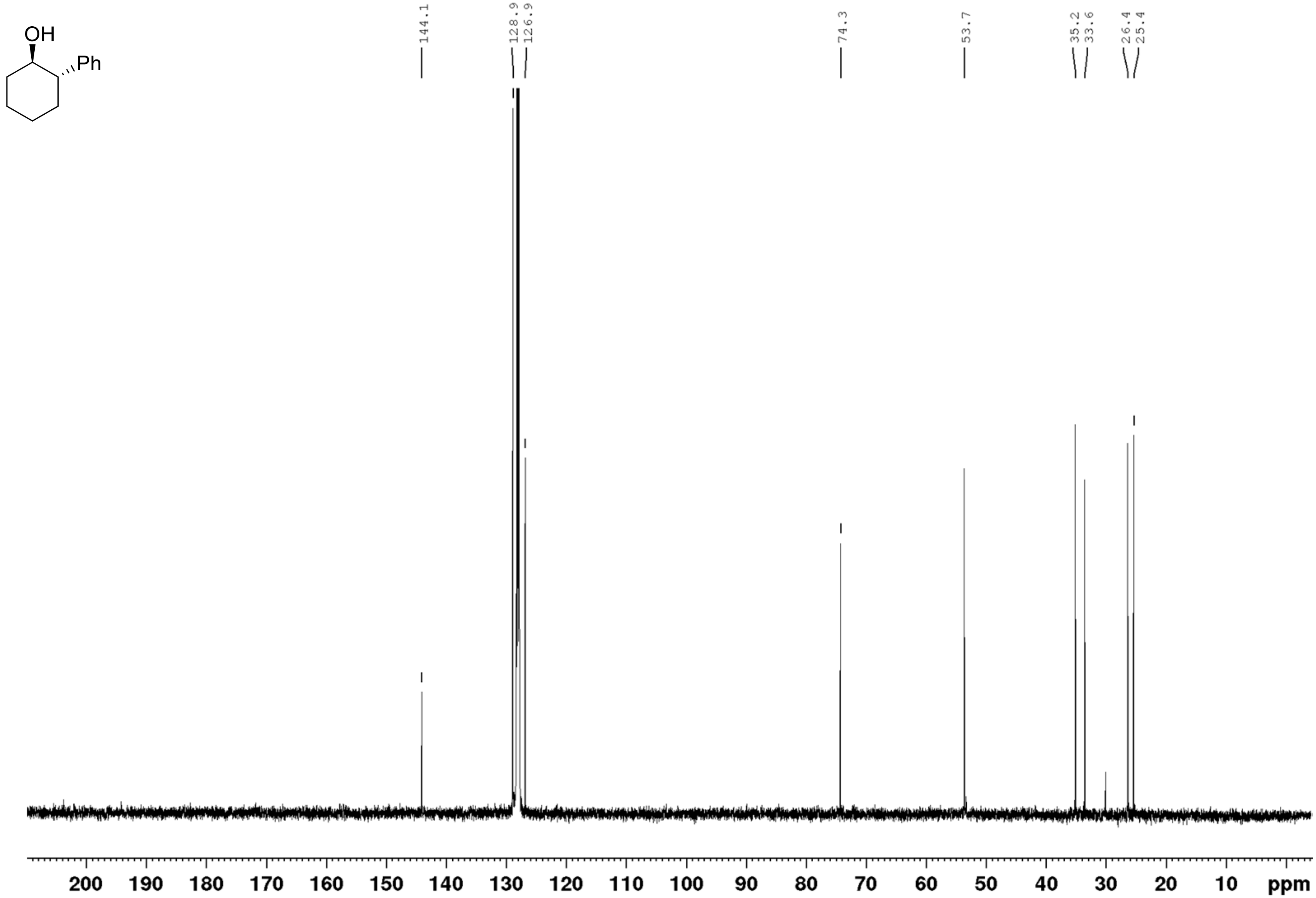

Supplementary Figure 219. ^{1}H NMR (500 MHz, C_6D_6) of Tributyl(((1*S*,2*R*)-2-phenylcyclohexyl)oxy)silane [(1*S*,2*R*)-5h]

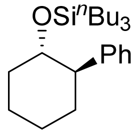

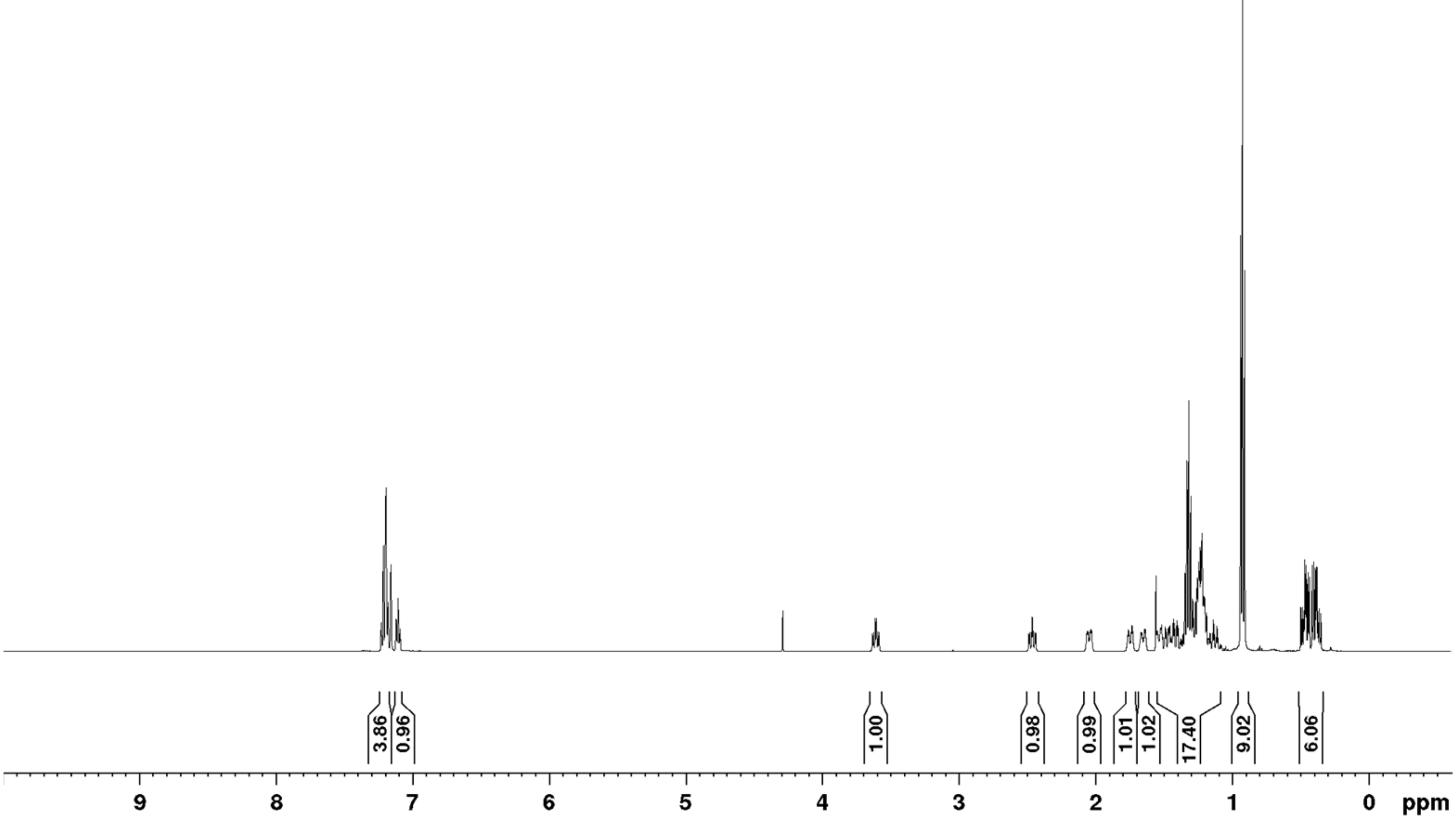

Supplementary Figure 220. ^{13}C NMR (126 MHz, C_6D_6) of Tributyl(((1*S*,2*R*)-2-phenylcyclohexyl)oxy)silane [(1*S*,2*R*)-5h]

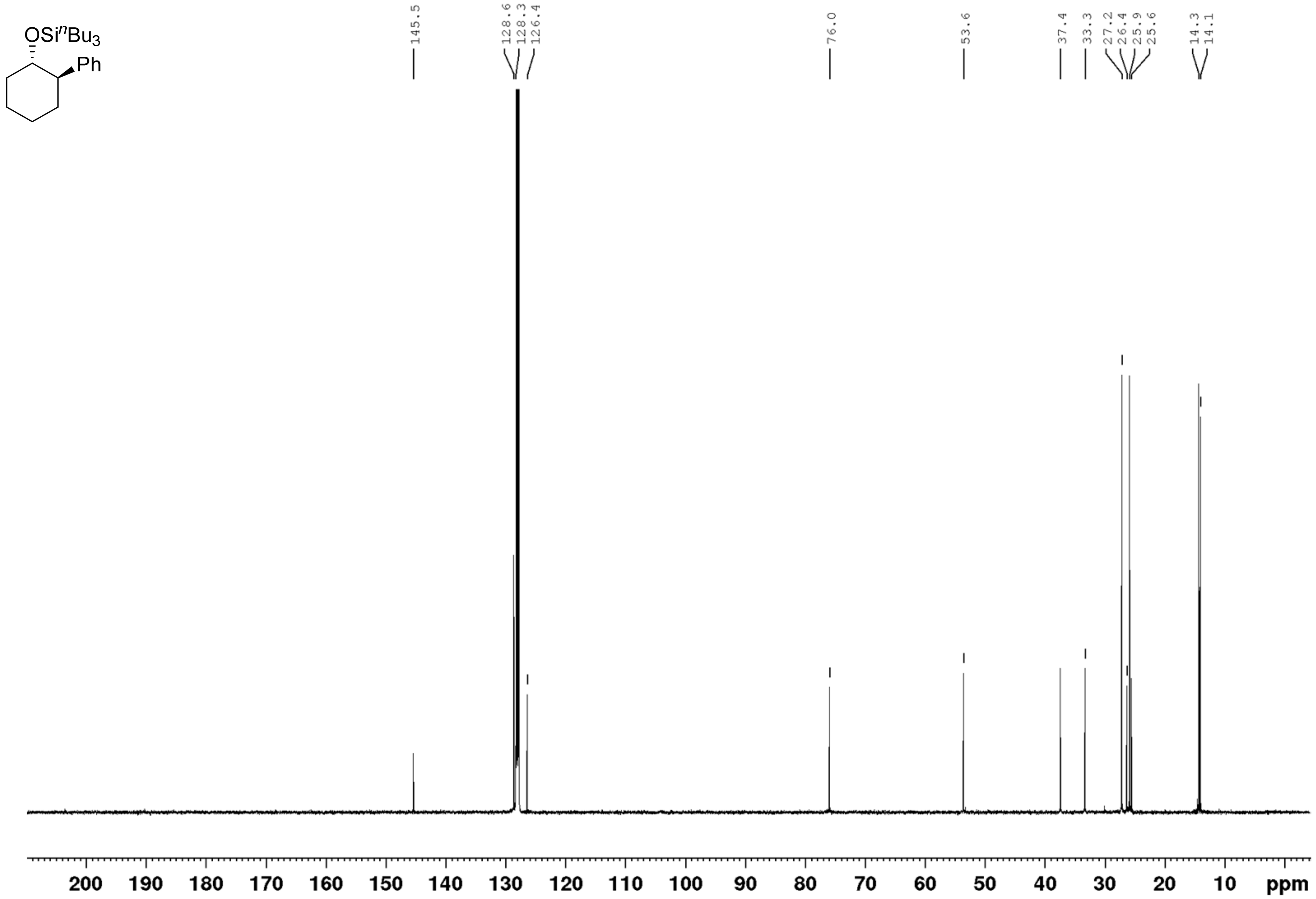

Supplementary Figure 221. ^{1}H NMR (500 MHz, $CDCl_3$) of (1*R*,2*R*)-2-Phenylcyclohexan-1-ol [(1*R*,2*R*)-4]

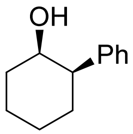

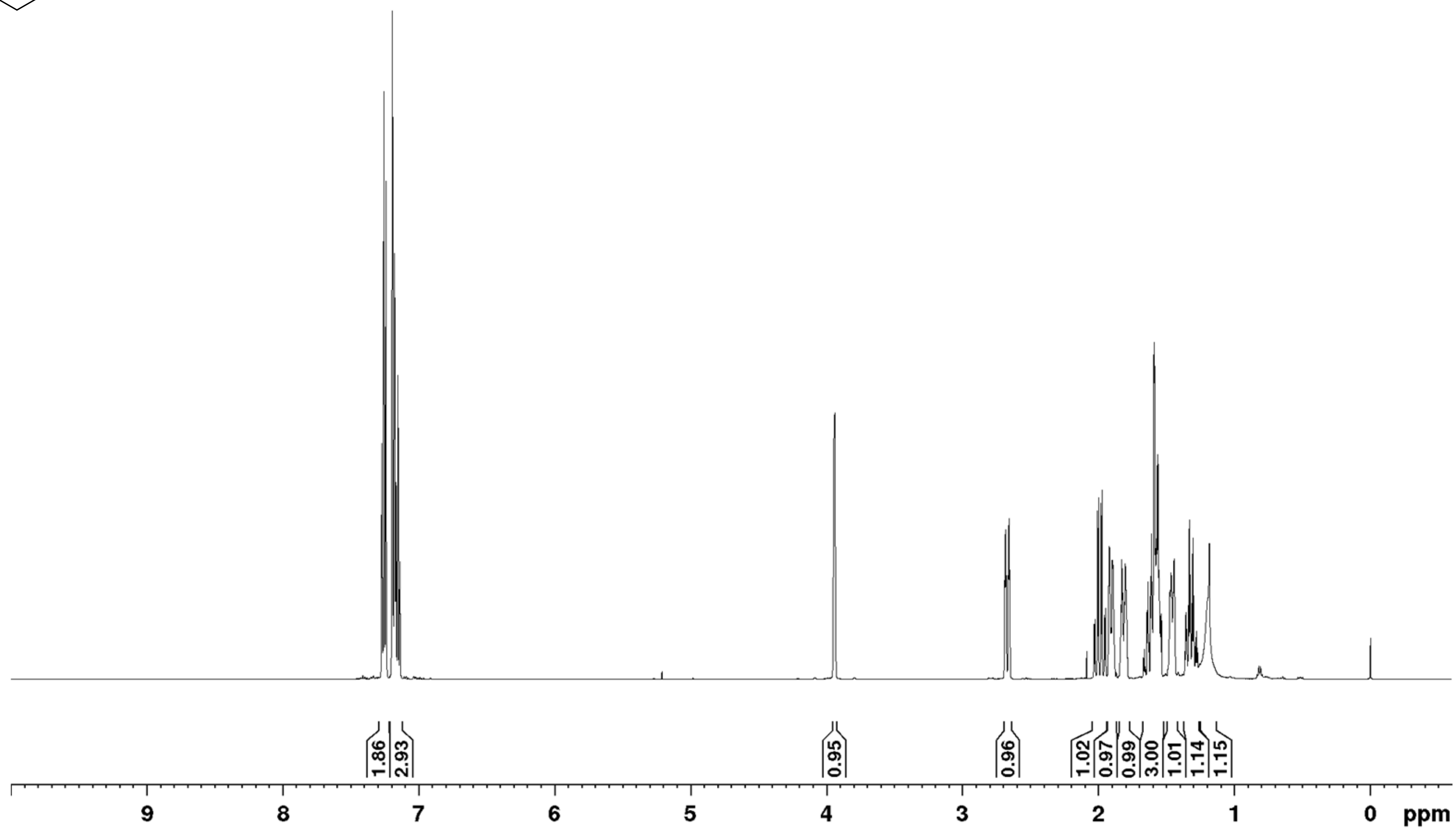

Supplementary Figure 222. ^{13}**C NMR (126 MHz, CDCl$_3$) of (1*R*,2*R*)-2-Phenylcyclohexan-1-ol [(1*R*,2*R*)-4]**

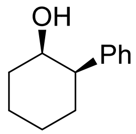

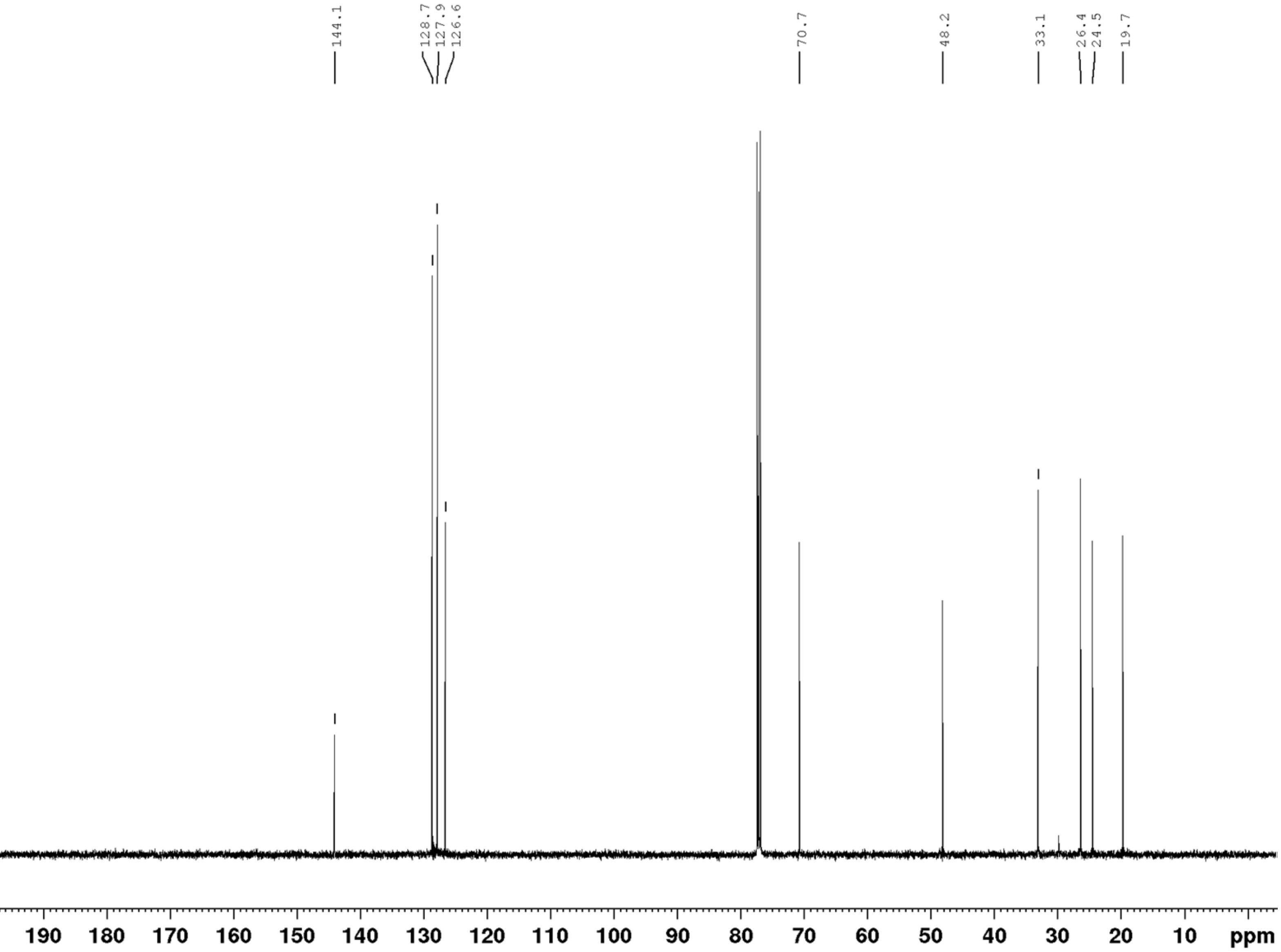

Supplementary Figure 223. ^{1}H NMR (500 MHz, C_6D_6) of Tributyl(((1*S*,2*S*)-2-phenylcyclohexyl)oxy)silane [(1*S*,2*S*)-5h]

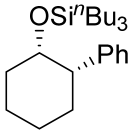

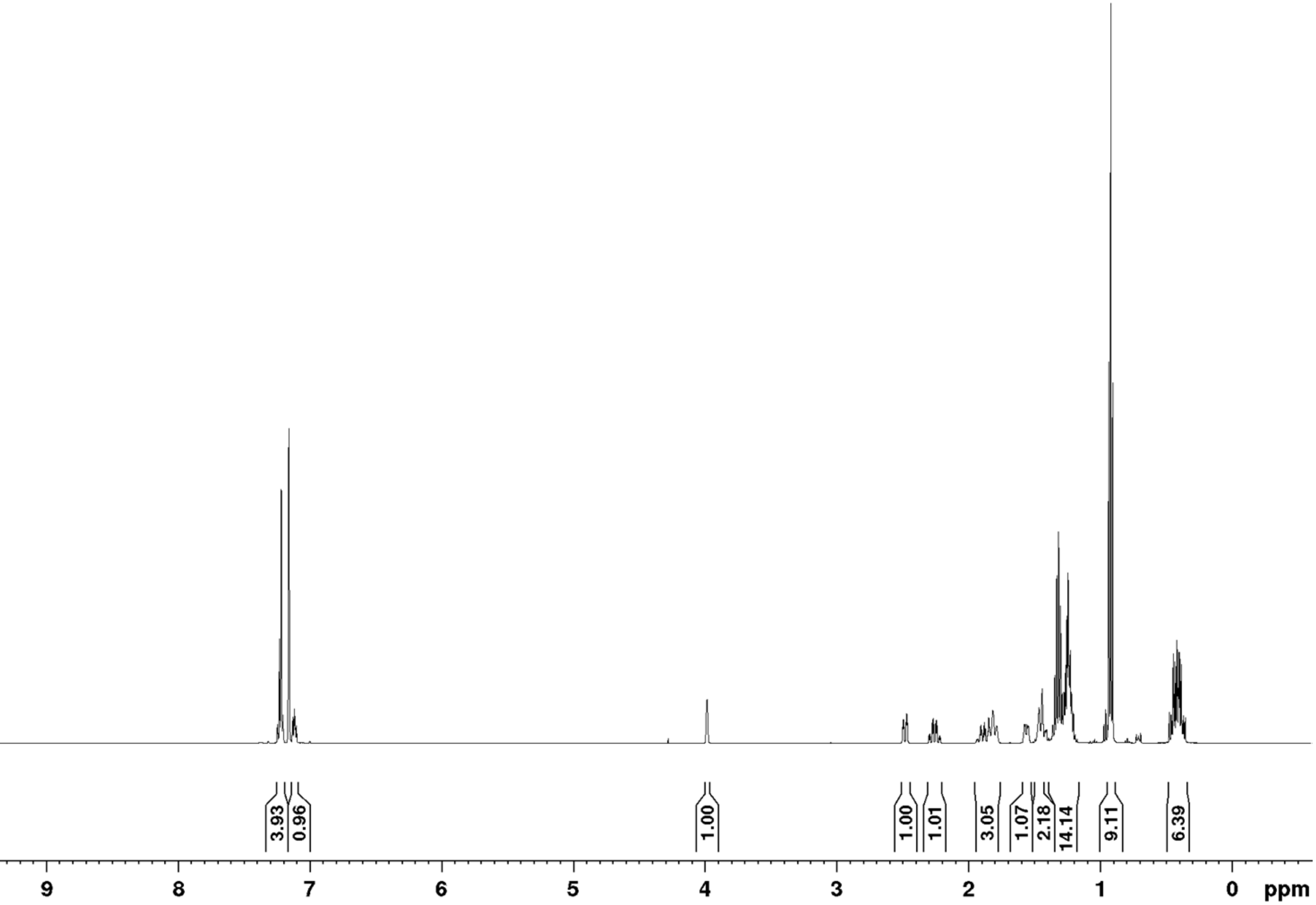

Supplementary Figure 224. ^{13}**C NMR (126 MHz,** C_6D_6**) of Tributyl(((1S,2S)-2-phenylcyclohexyl)oxy)silane [(1*S*,2*S*)-5h]**

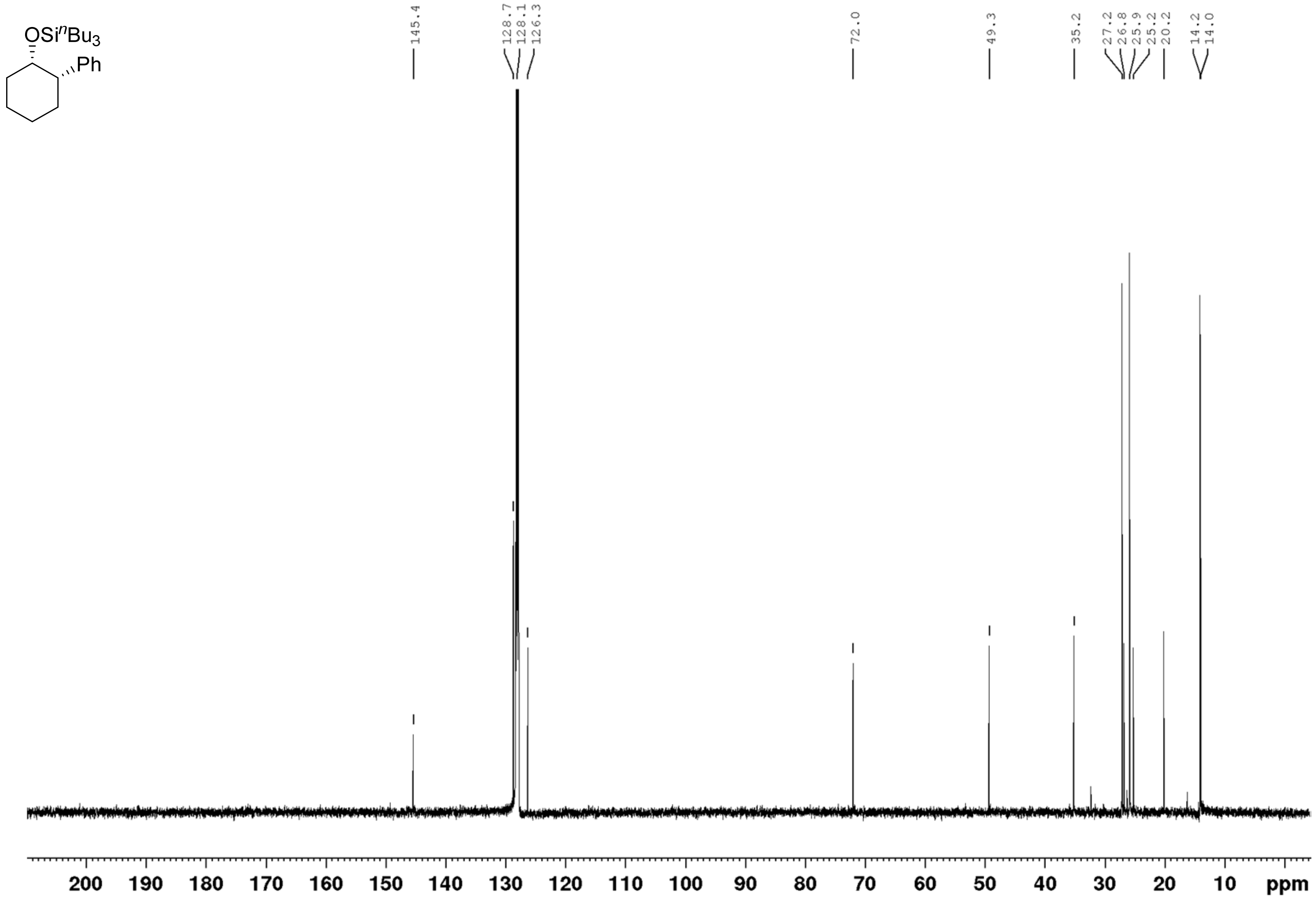

Supplementary Figure 225. **^{1}H NMR (500 MHz, C_6D_6) of (*R*,*E*)-3-Methyl-4-phenylbut-3-en-2-ol [(*R*)-6a]**

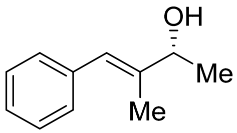

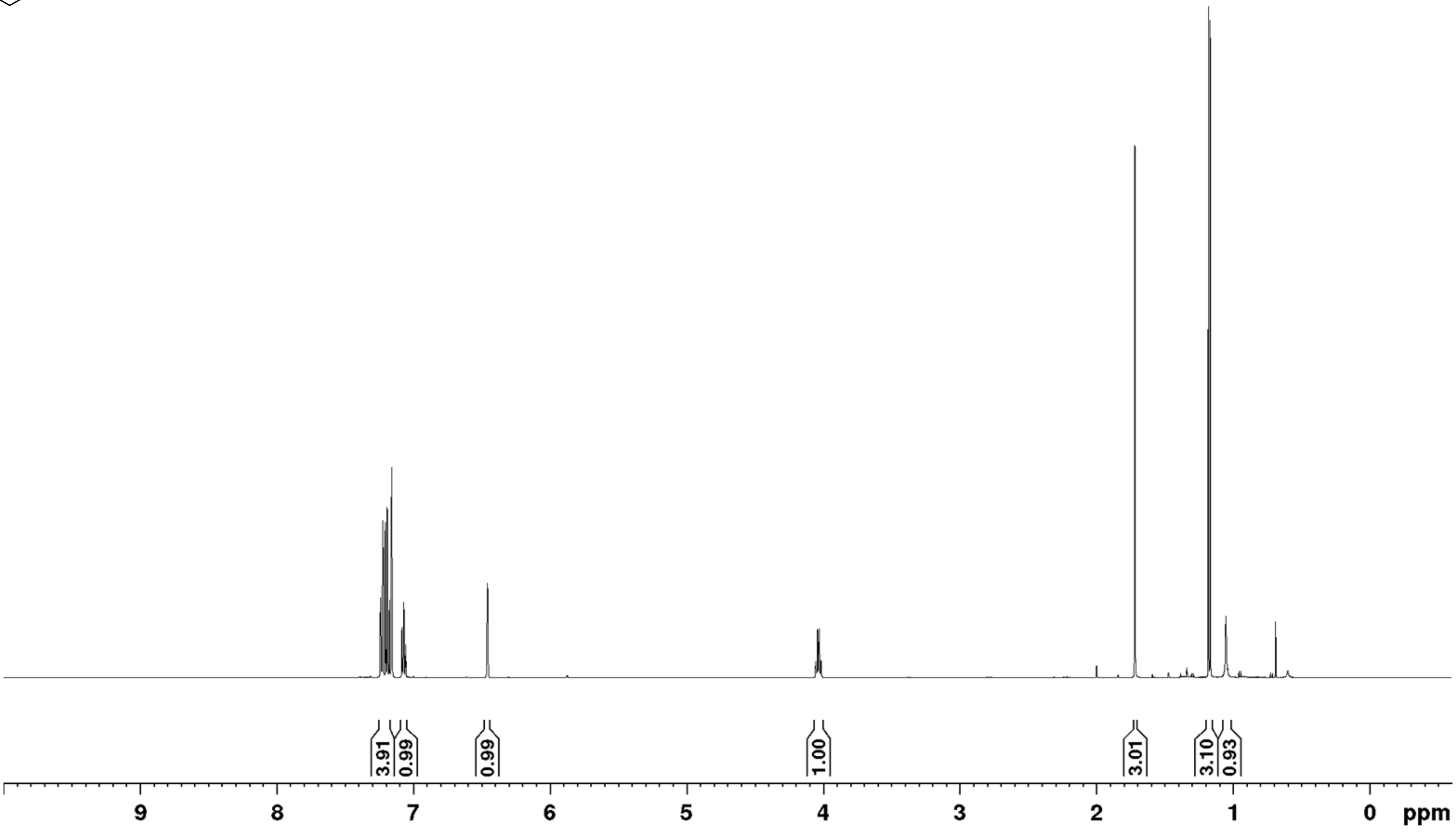

Supplementary Figure 226. ^{13}C NMR (126 MHz, C_6D_6) of (*R*,*E*)-3-Methyl-4-phenylbut-3-en-2-ol [(*R*)-6a]

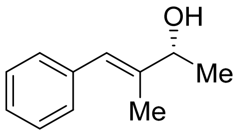

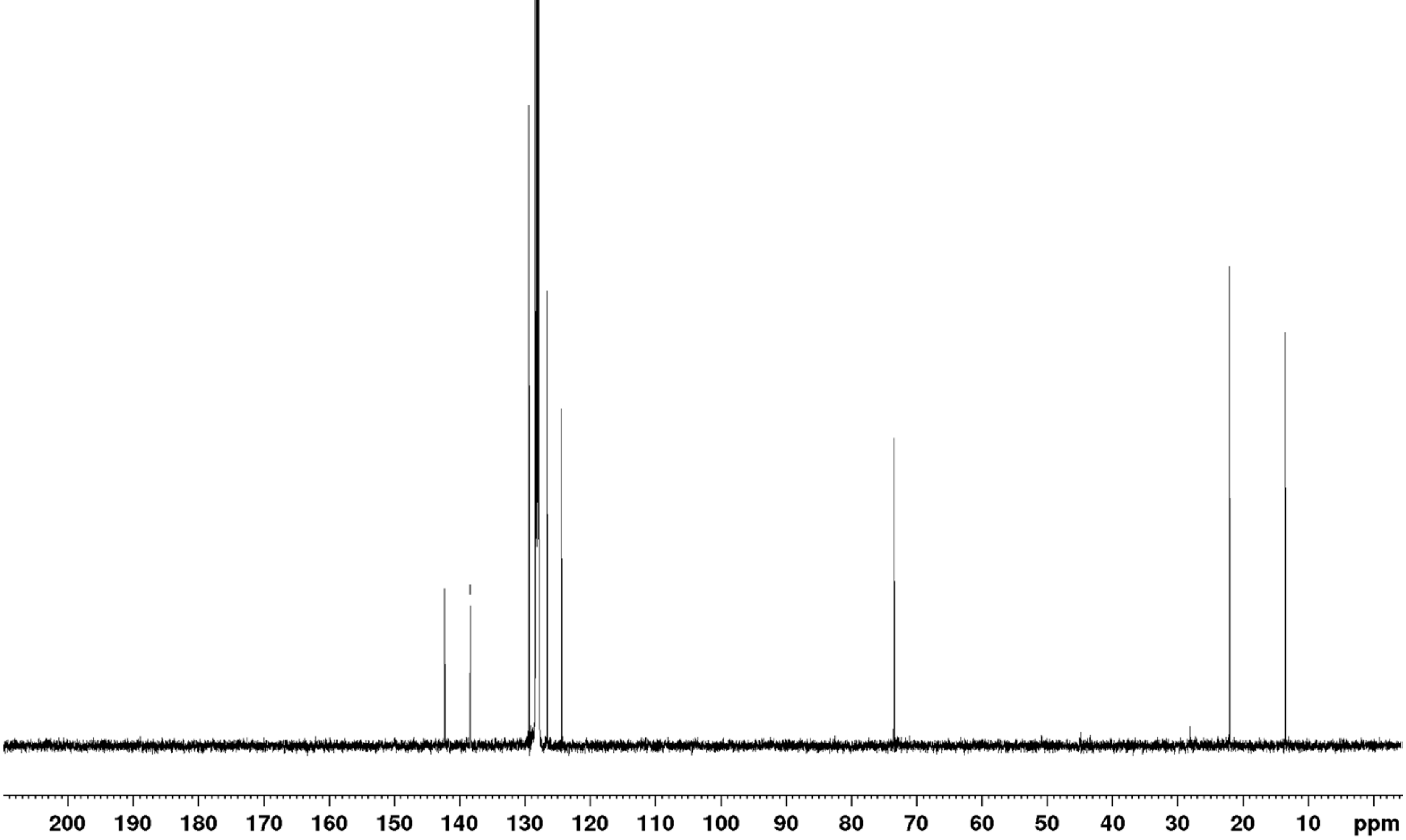

Supplementary Figure 227. ^{1}H **NMR (400 MHz,** C_6D_6**) of (*S*,*E*)-Tributyl((3-methyl-4-phenylbut-3-en-2-yl)oxy)silane [(*S*)-7ah]**

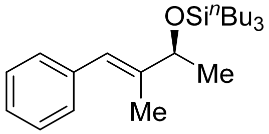

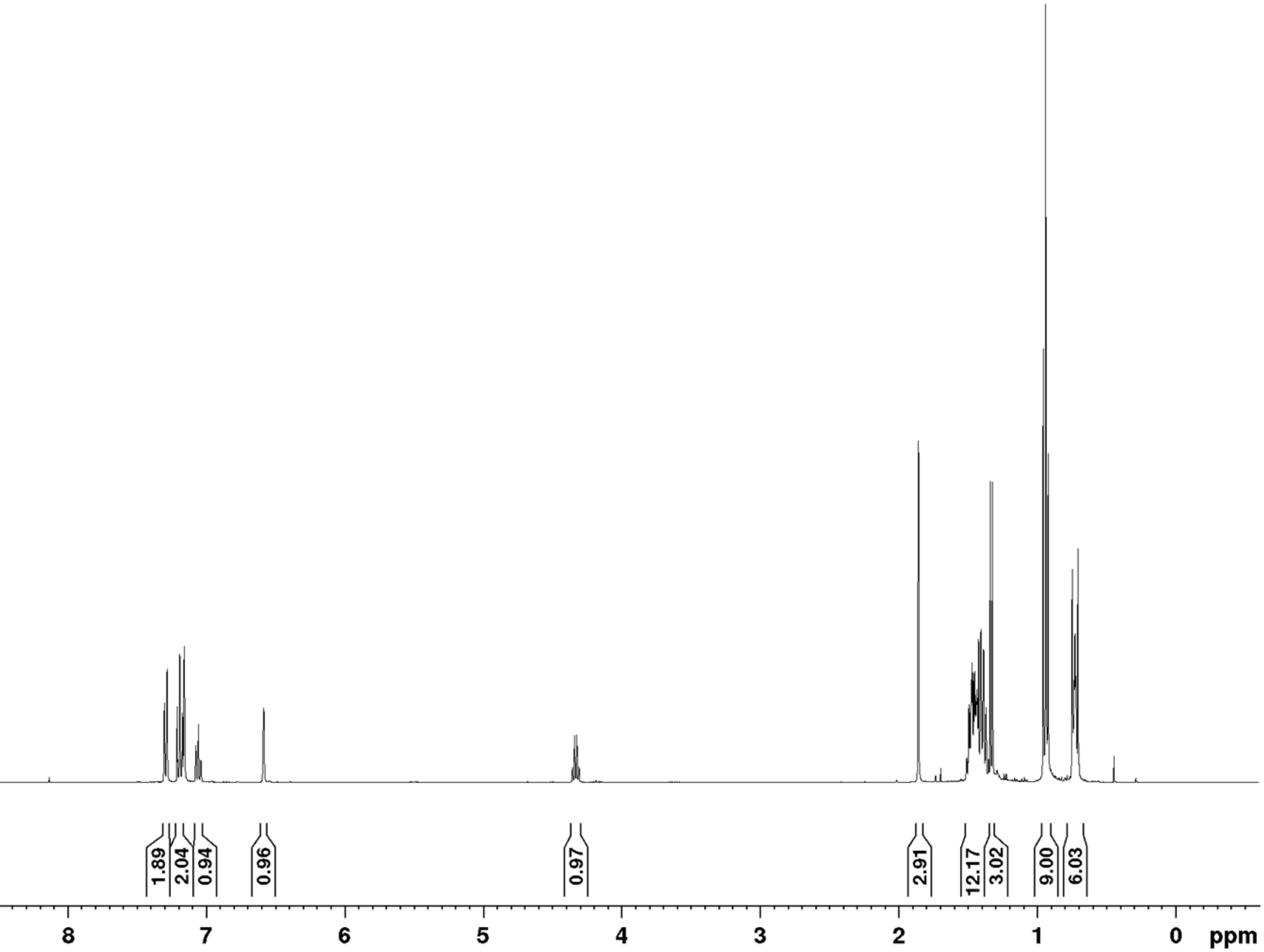

Supplementary Figure 228. ^{13}**C NMR (126 MHz, C_6D_6) of (*S*,*E*)-Tributyl((3-methyl-4-phenylbut-3-en-2-yl)oxy)silane [(*S*)-7ah]**

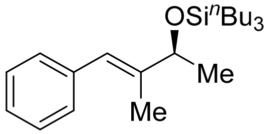

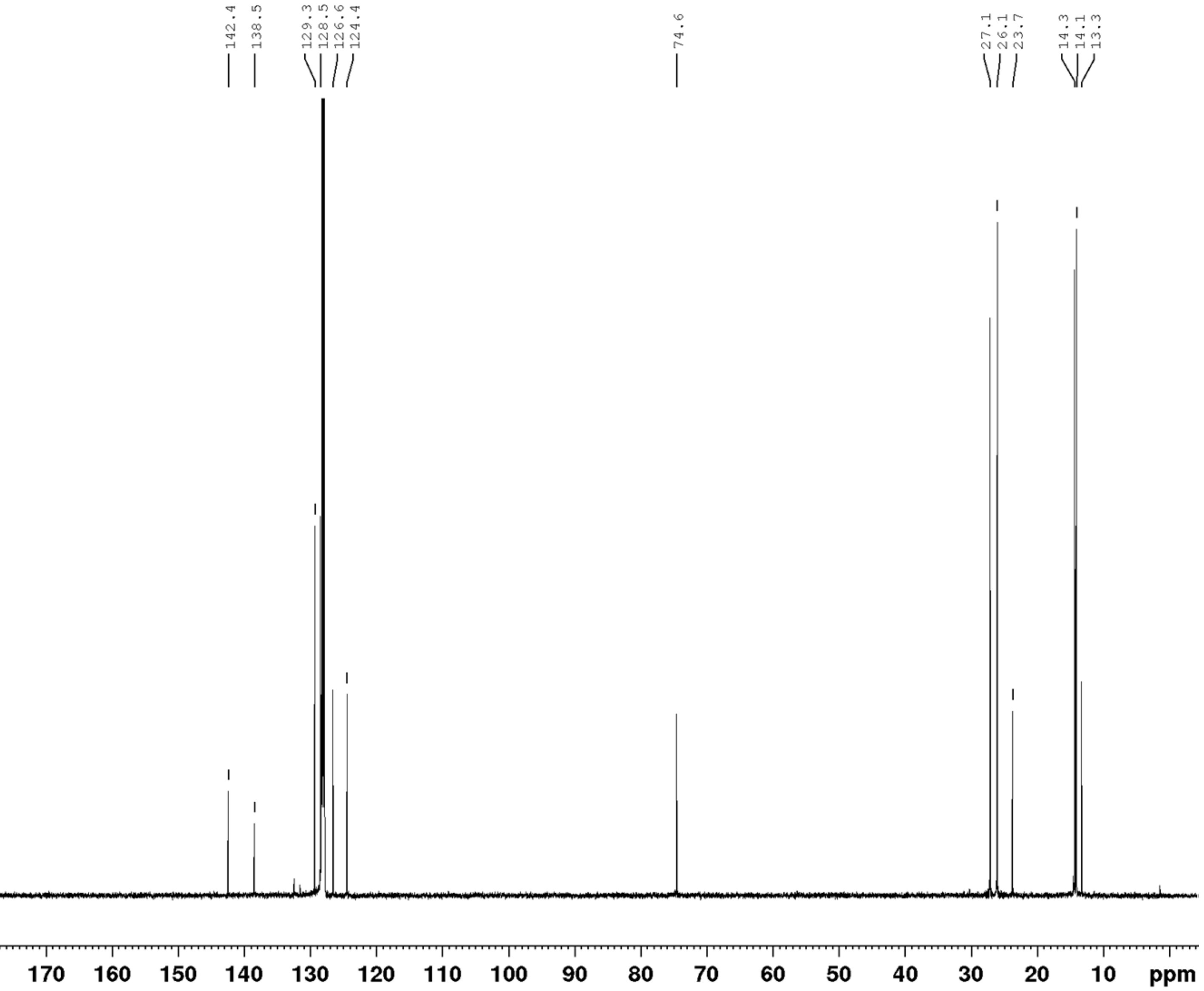

Supplementary Figure 229. **^{1}H NMR (500 MHz, $CDCl_3$) of (*R*,*E*)-3-(4-Methoxyphenyl)-4-phenylbut-3-en-2-ol [(*R*)-6b]**

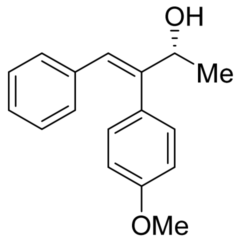

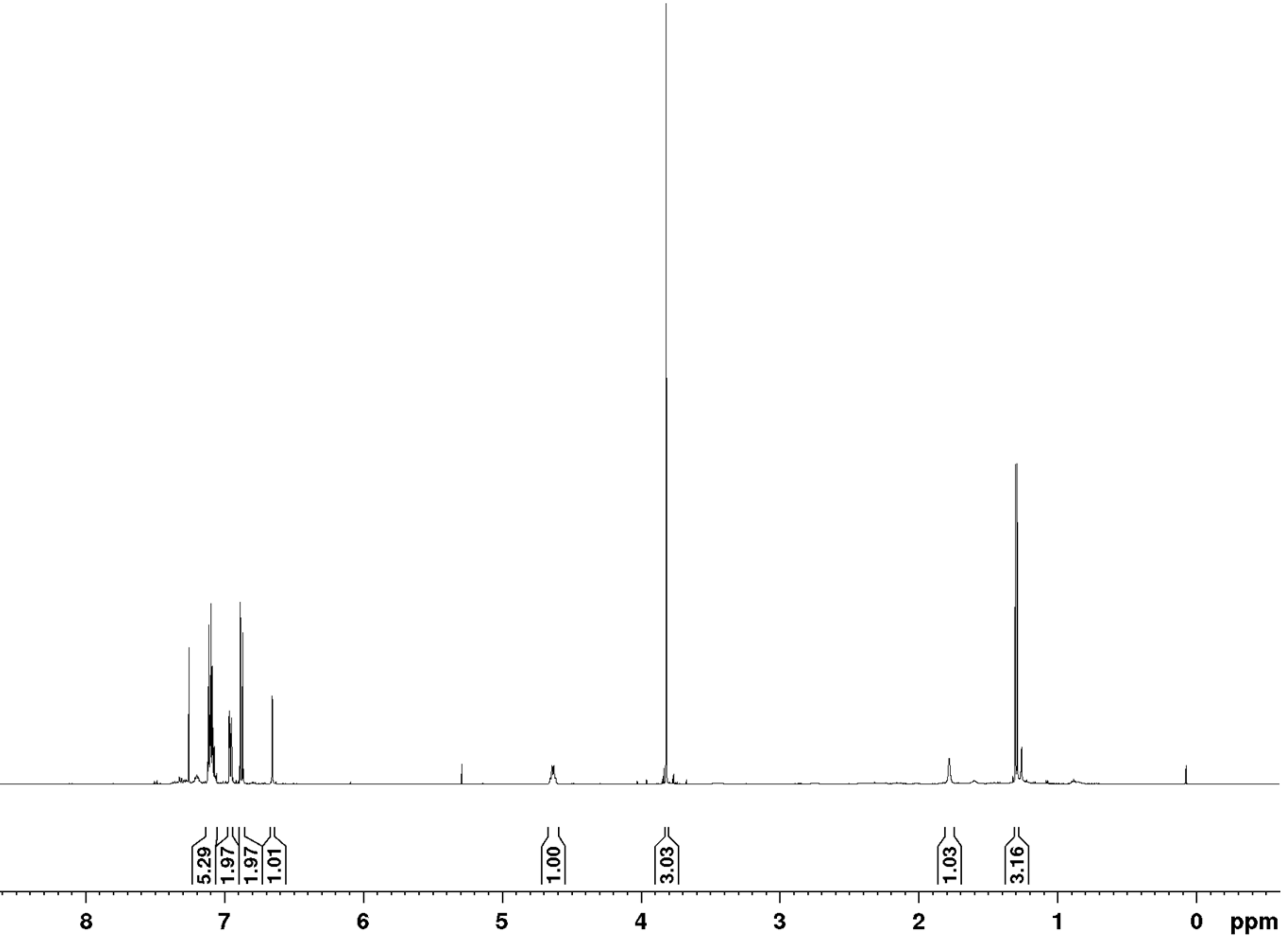

Supplementary Figure 230. ^{13}**C NMR (126 MHz, CDCl$_3$) of (*R*,*E*)-3-(4-Methoxyphenyl)-4-phenylbut-3-en-2-ol [(*R*)-6b]**

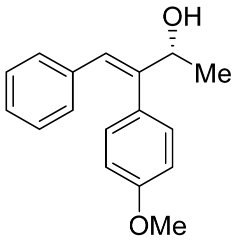

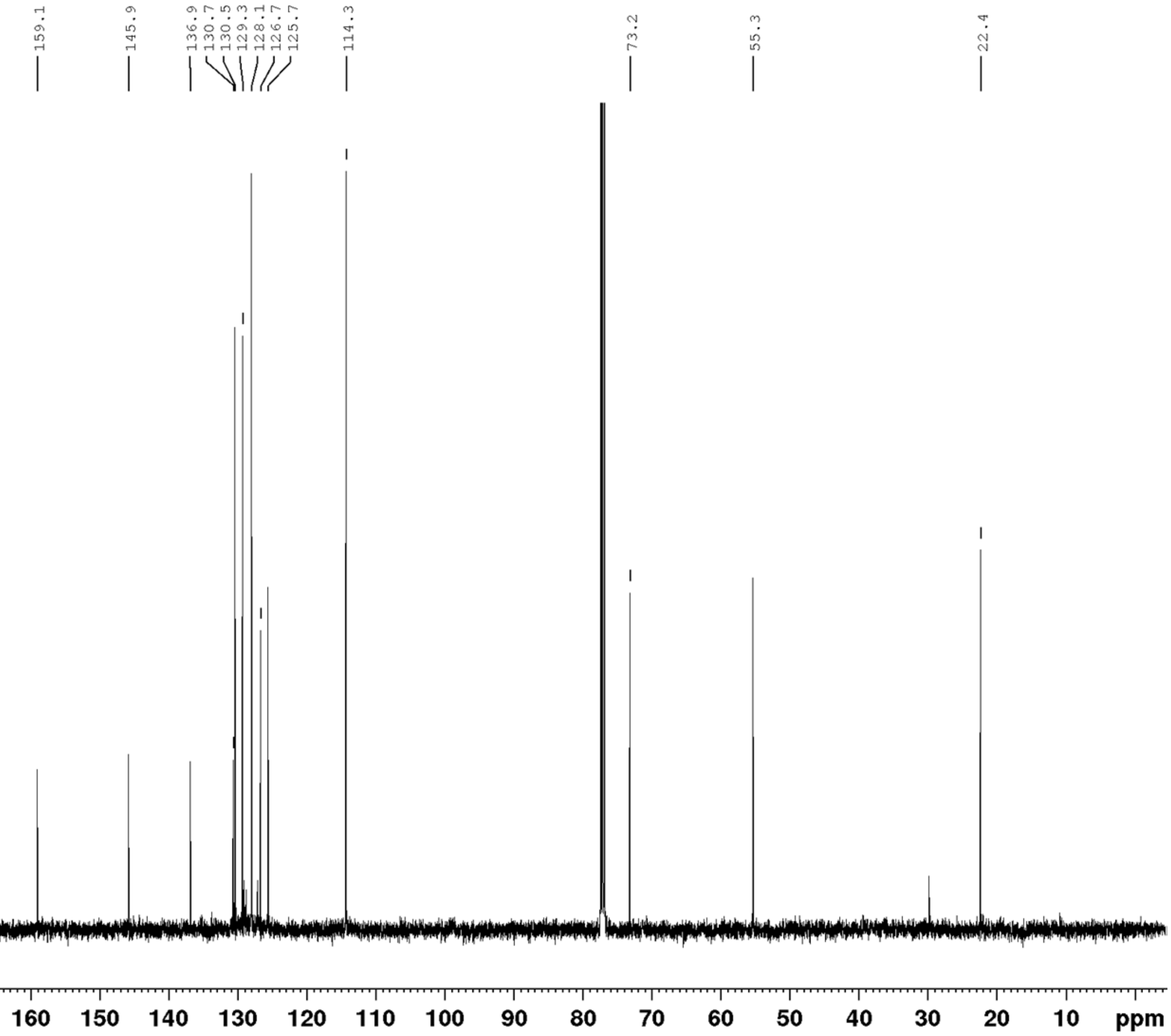

Supplementary Figure 231. ^{1}**H NMR (500 MHz, C_6D_6) of (*S*,*E*)-Tributyl((3-(4-methoxyphenyl)-4-phenylbut-3-en-2-yl)oxy)silane [(*S*)-7bh]**

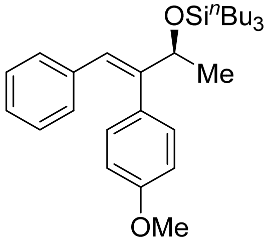

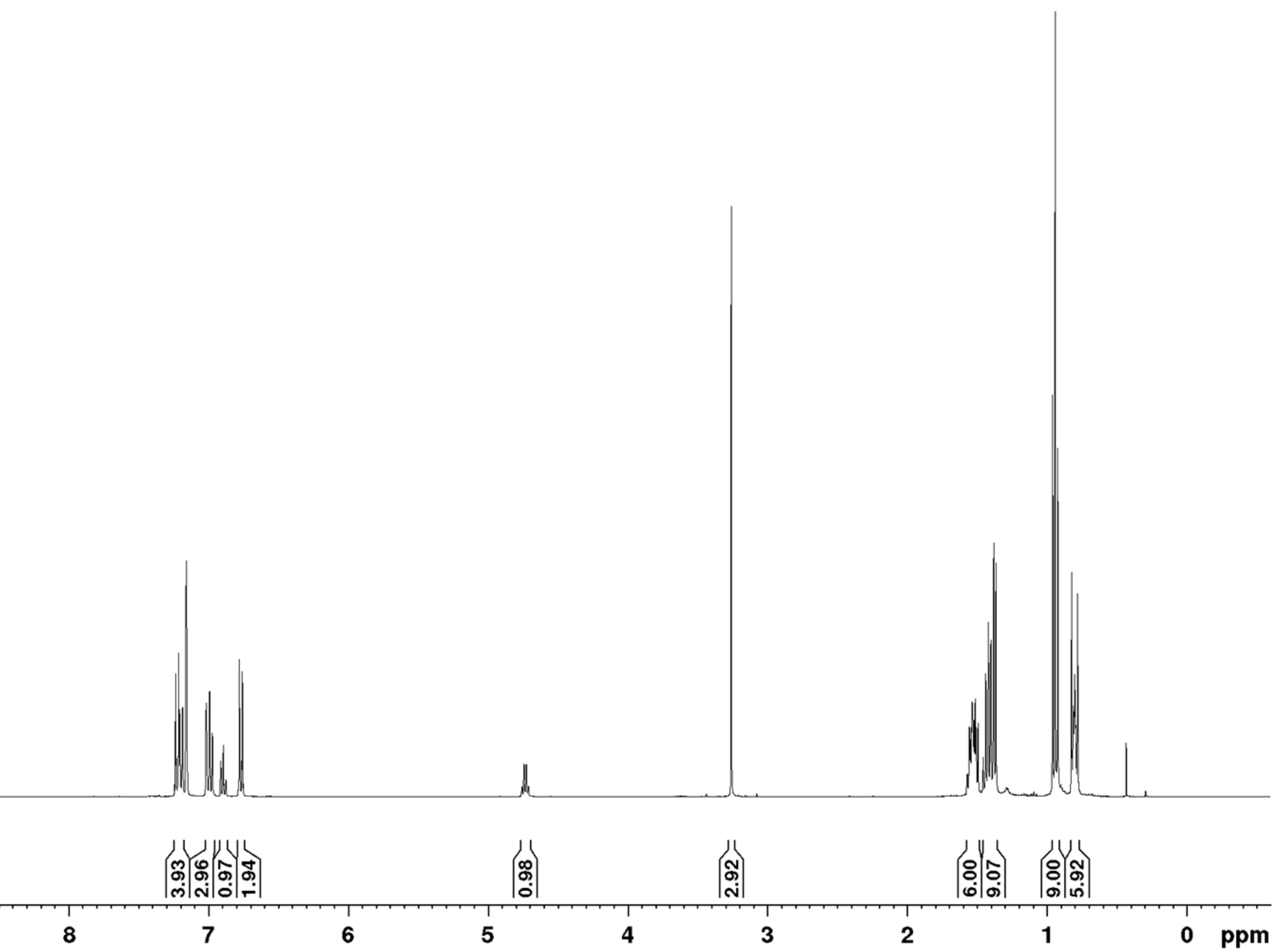

Supplementary Figure 232. ^{13}C NMR (126 MHz, C_6D_6) of (*S*,*E*)-Tributyl((3-(4-methoxyphenyl)-4-phenylbut-3-en-2-yl)oxy)silane [(*S*)-7bh]

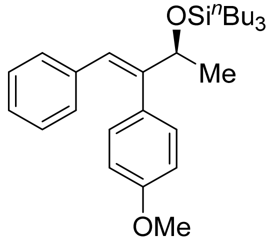

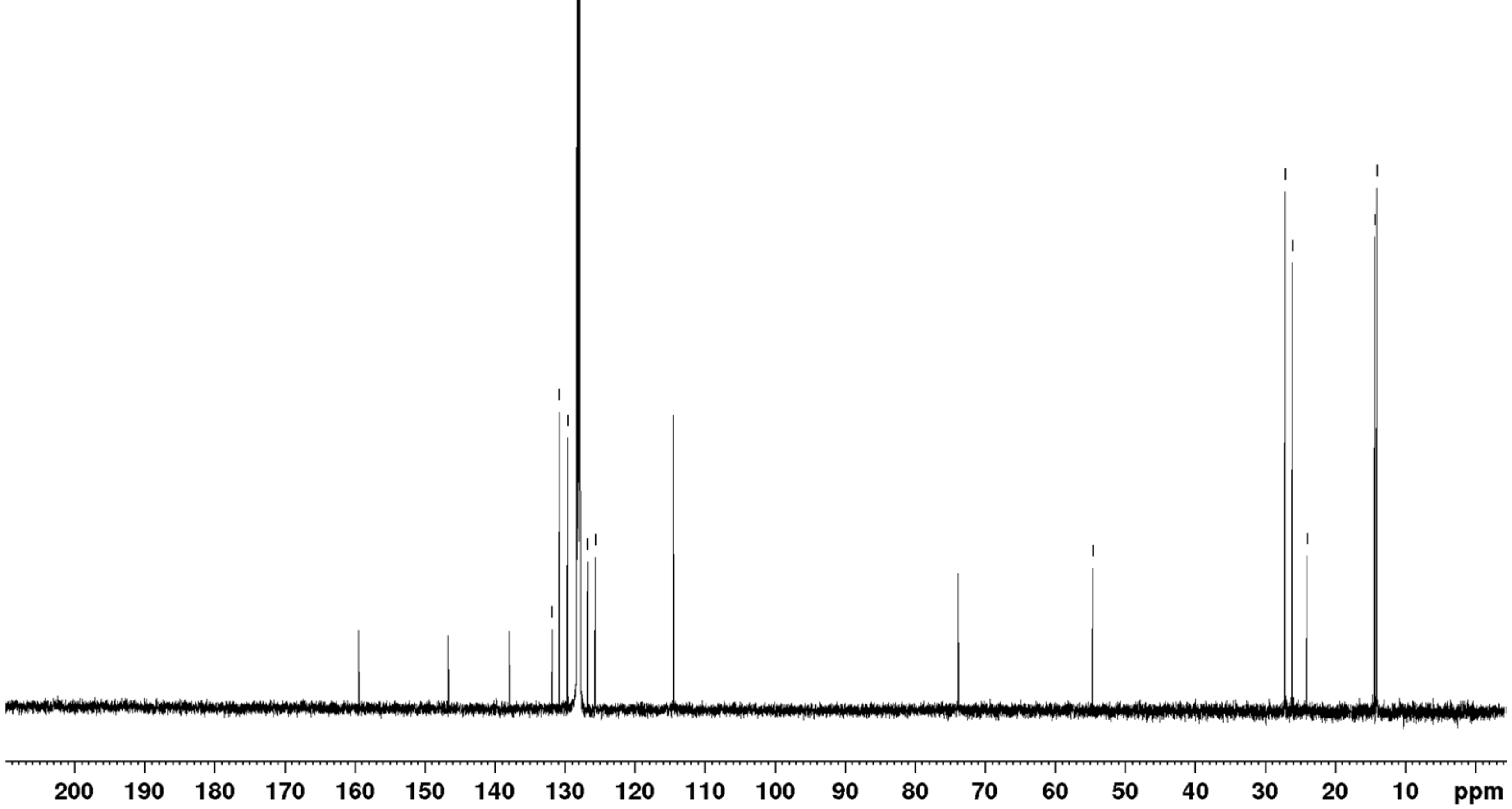

Supplementary Figure 233. [1]**H NMR (500 MHz, CDCl$_3$) of (*R*,*Z*)-3-Bromo-4-phenylbut-3-en-2-ol [(*R*)-6c]**

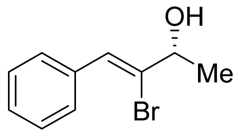

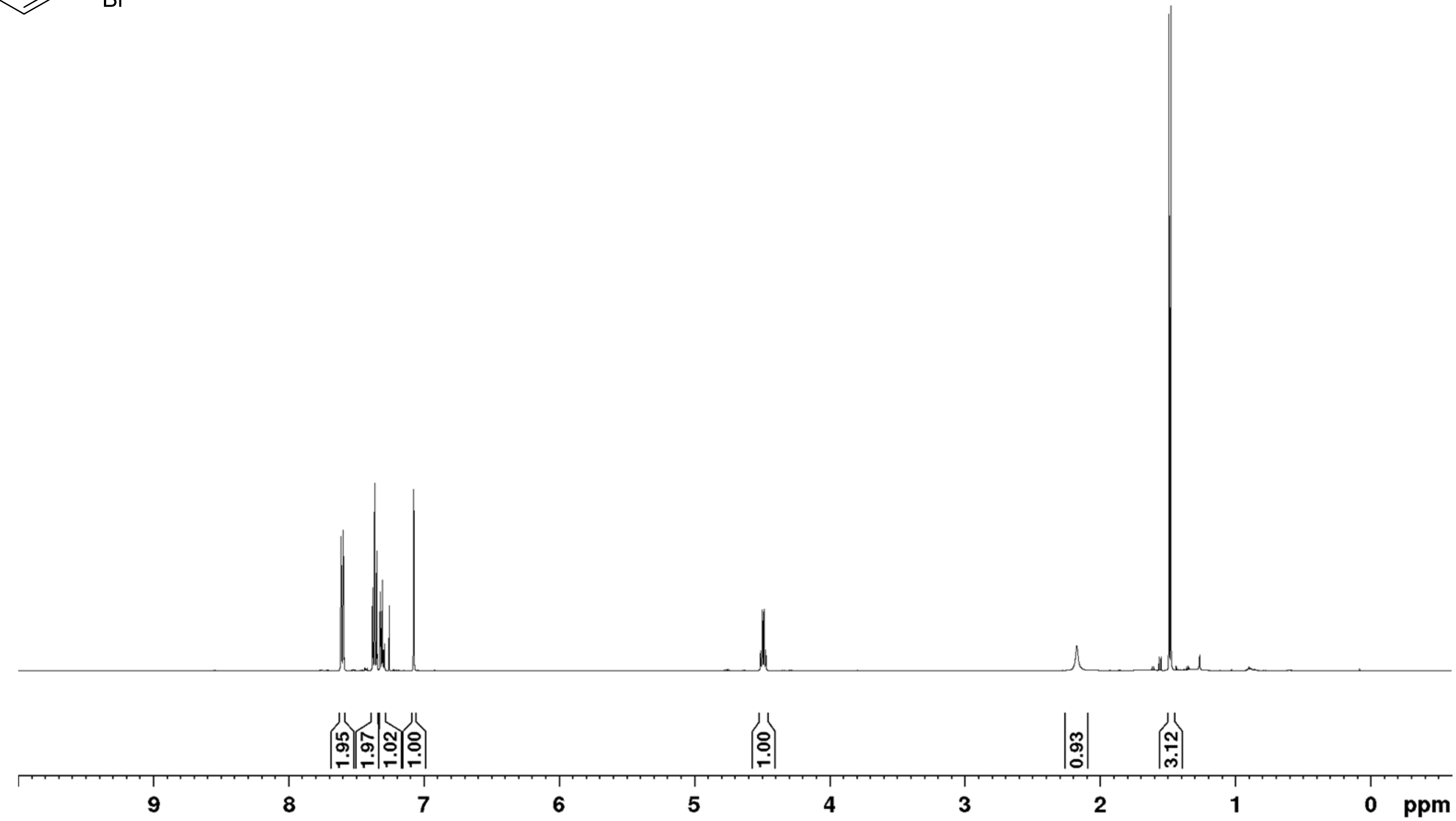

Supplementary Figure 234. ^{13}**C NMR (126 MHz, CDCl$_3$) of (*R*,*Z*)-3-Bromo-4-phenylbut-3-en-2-ol [(*R*)-6c]**

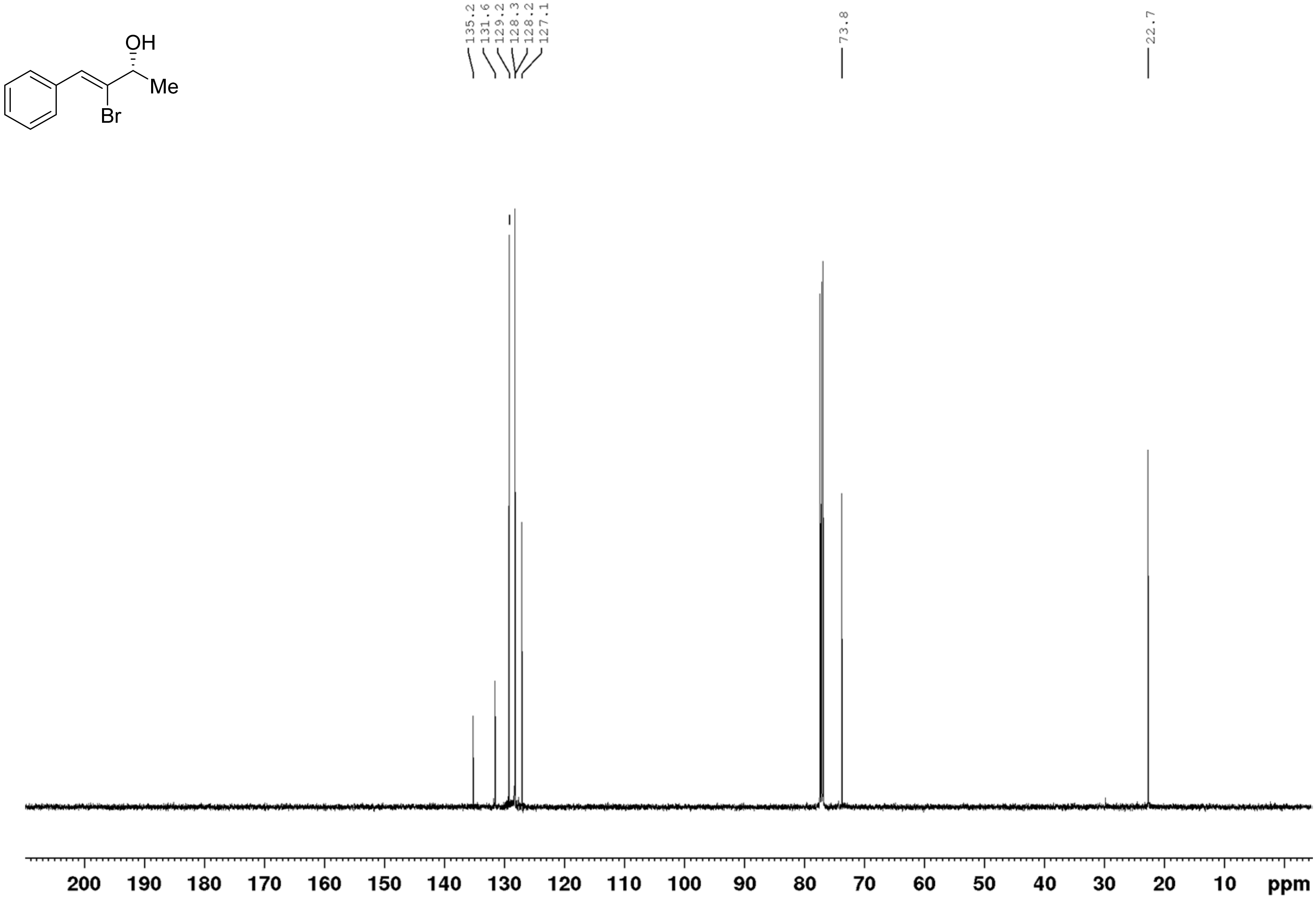

Supplementary Figure 235. ^{1}H NMR (500 MHz, C_6D_6) of (*S*,*Z*)-((3-Bromo-4-phenylbut-3-en-2-yl)oxy)tributylsilane [(*S*)-7ch]

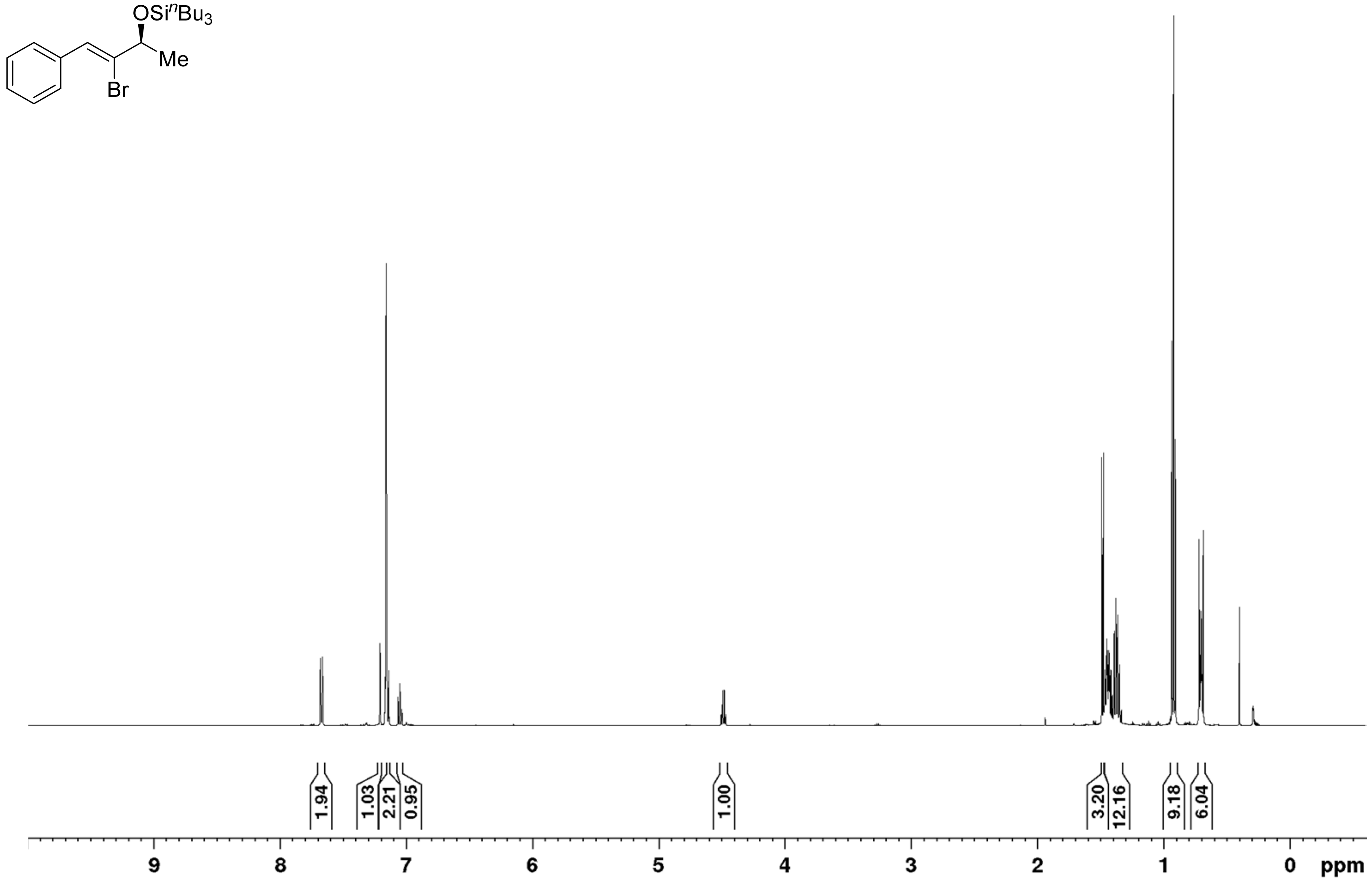

Supplementary Figure 236. ^{13}**C NMR (126 MHz,** C_6D_6**) of (*S*,*Z*)-((3-Bromo-4-phenylbut-3-en-2-yl)oxy)tributylsilane [(*S*)-7ch]**

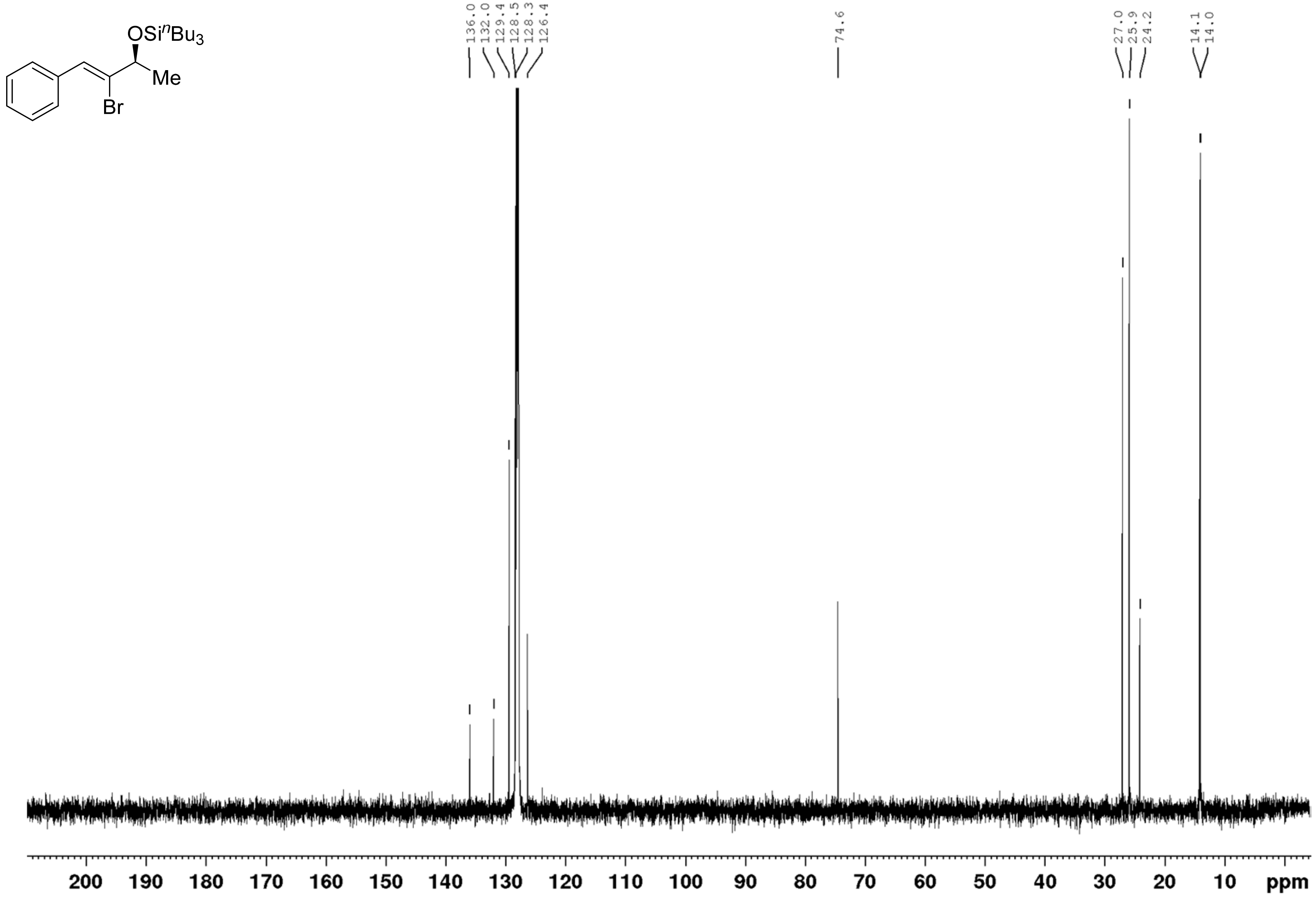

Supplementary Figure 237. ^{1}**H NMR (500 MHz,** $CDCl_3$**) of (*R*)-1-(Cyclohex-1-en-1-yl)ethan-1-ol [(*R*)-6d]**

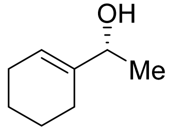

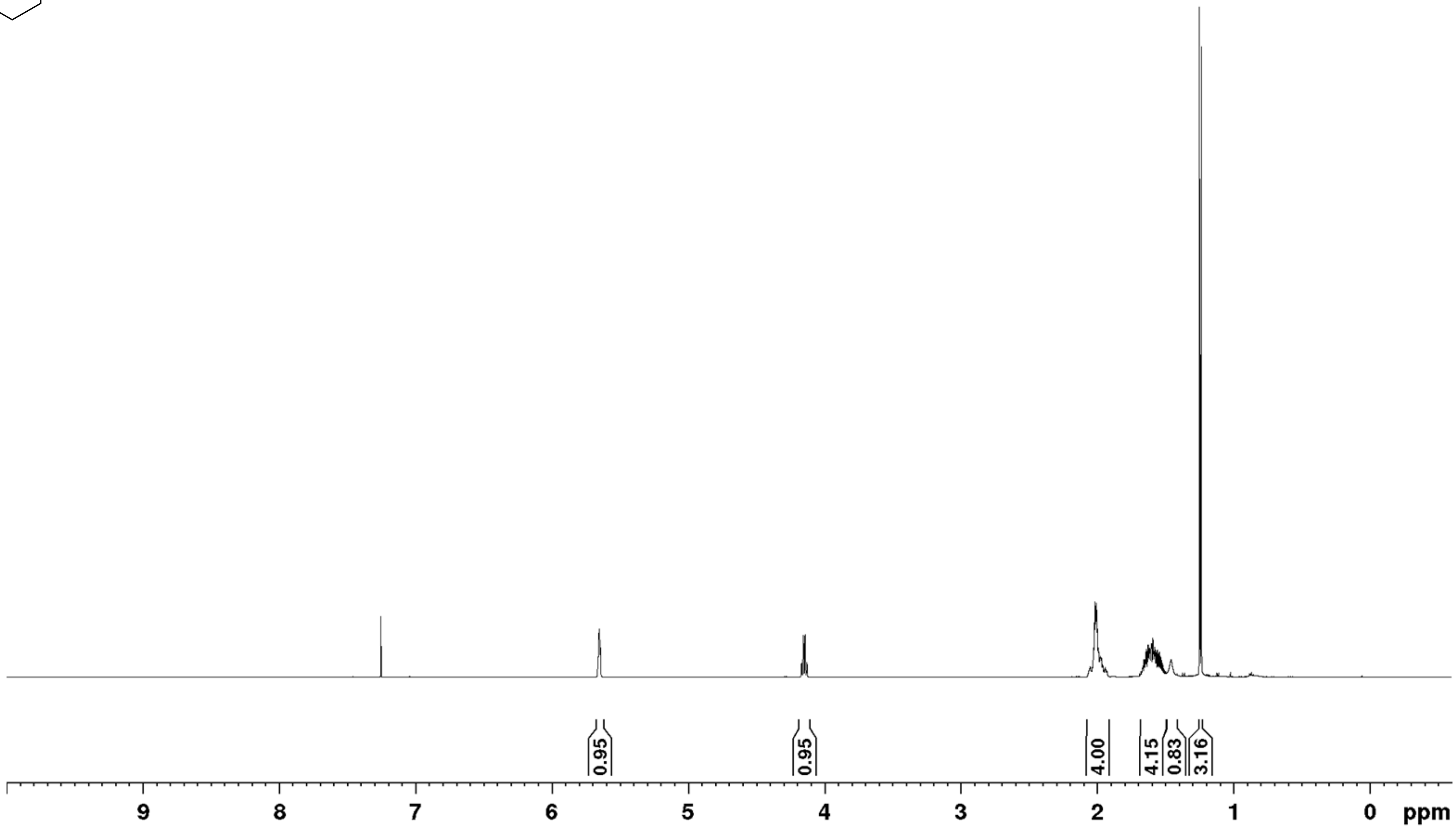

Supplementary Figure 238. ^{13}**C NMR (126 MHz, CDCl$_3$) of (*R*)-1-(Cyclohex-1-en-1-yl)ethan-1-ol [(*R*)-6d]**

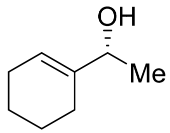

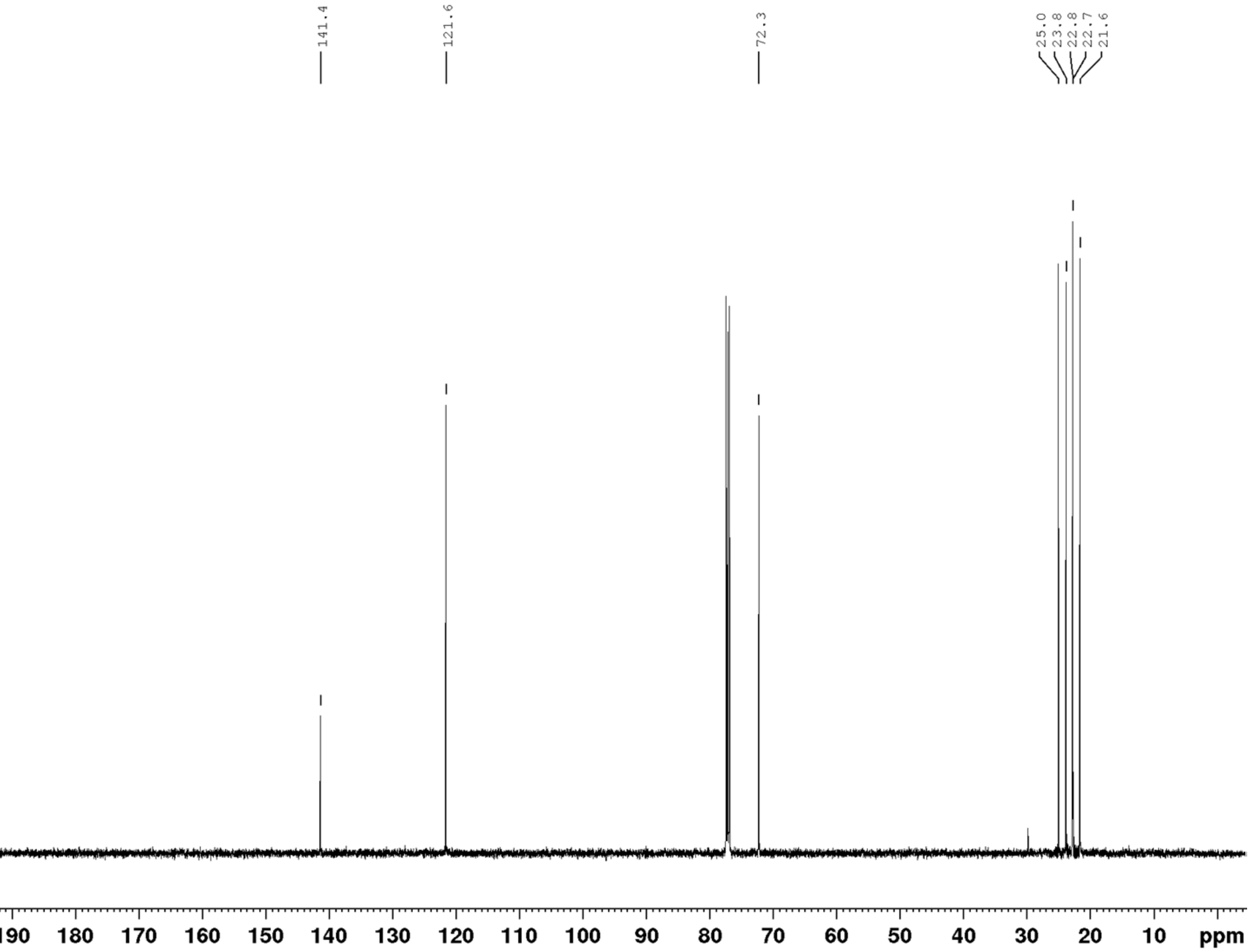

Supplementary Figure 239. ^{1}H NMR (500 MHz, C_6D_6) of (*S*)-Tributyl(1-(cyclohex-1-en-1-yl)ethoxy)silane [(*S*)-7dh]

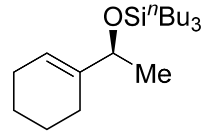

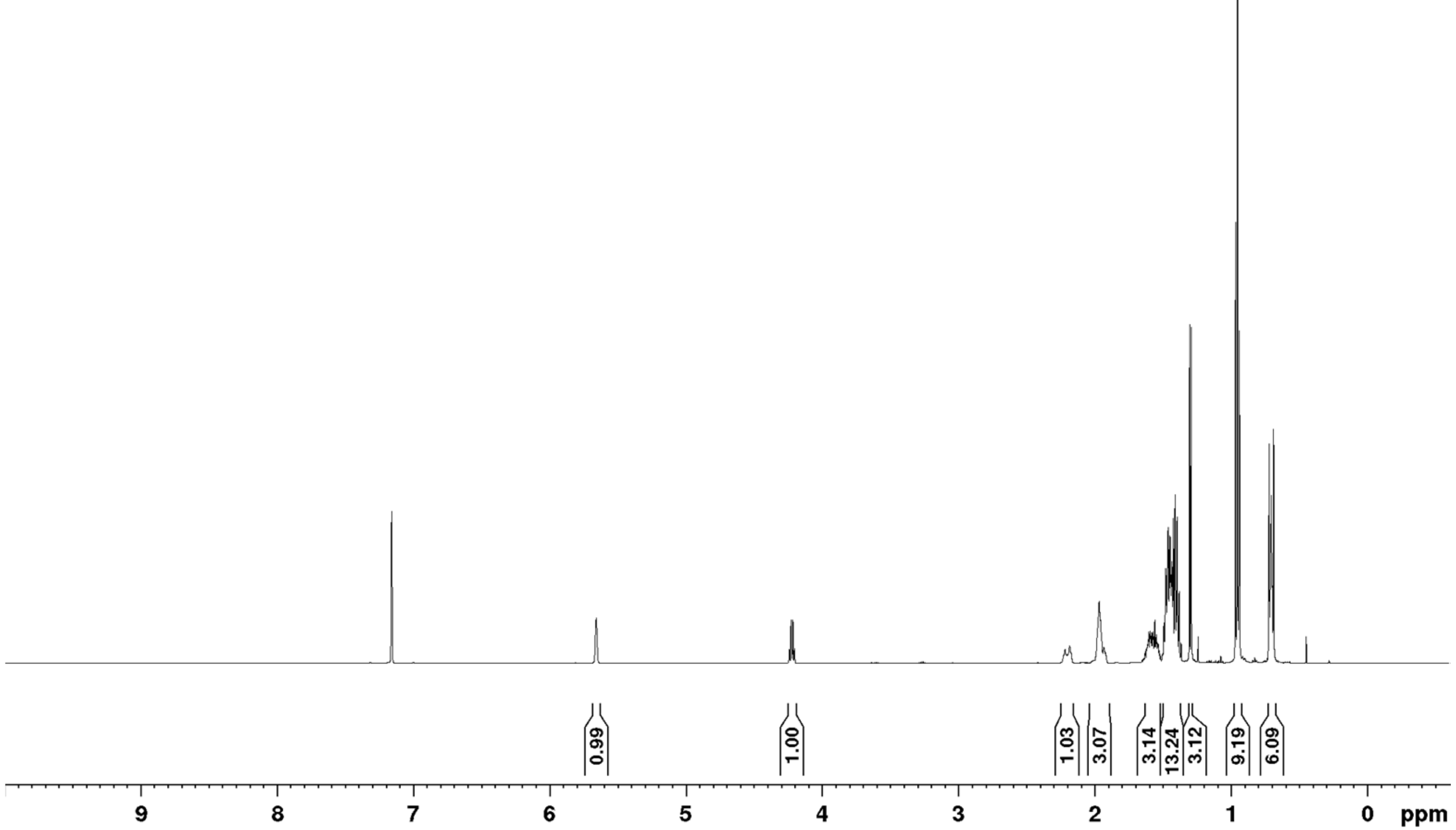

Supplementary Figure 240. ^{13}C NMR (126 MHz, C_6D_6) of (*S*)-Tributyl(1-(cyclohex-1-en-1-yl)ethoxy)silane [(*S*)-7dh]

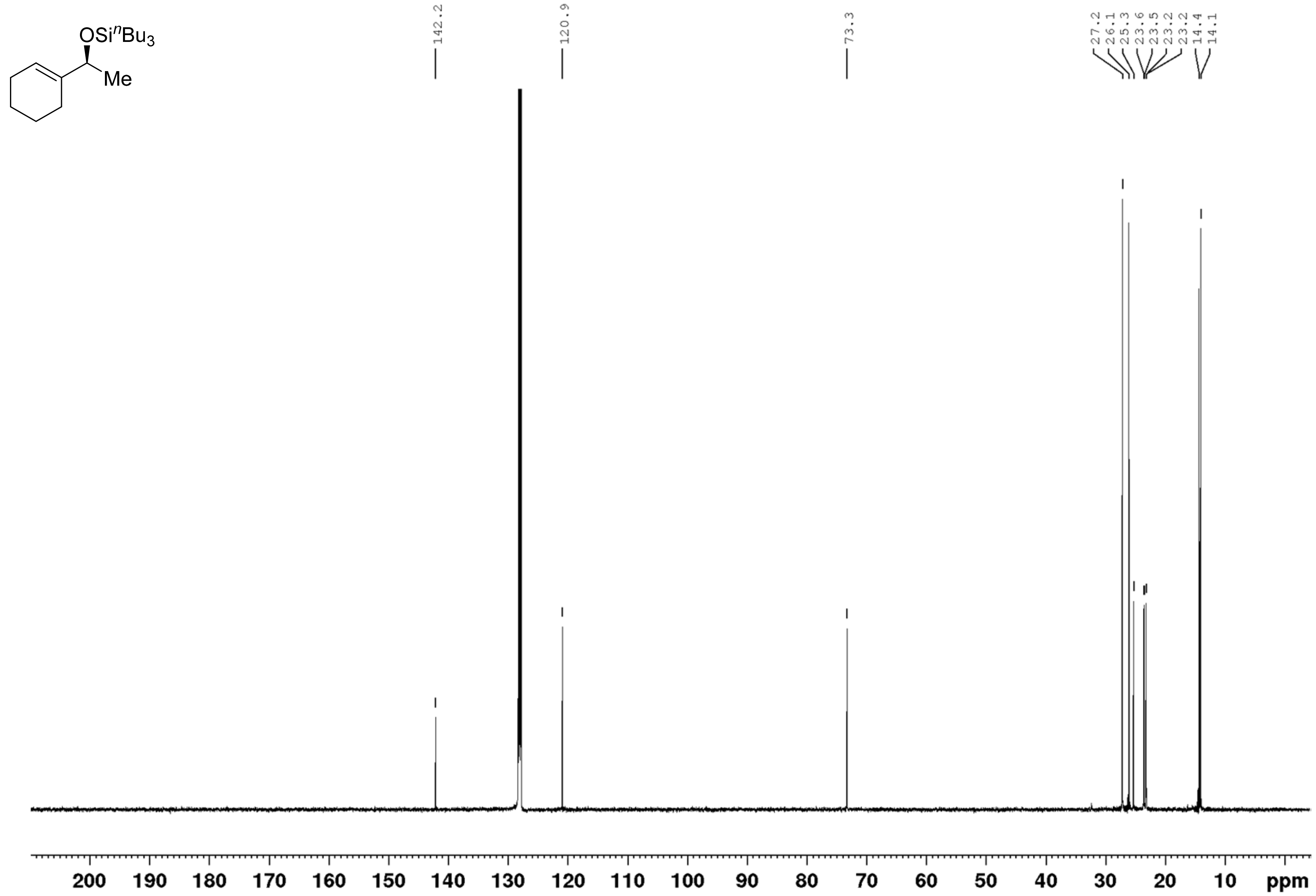

Supplementary Figure 241. ^{1}H NMR (400 MHz, C_6D_6) of (*R*,*E*)-2-Benzylidenecyclopentan-1-ol [(*R*)-6e]

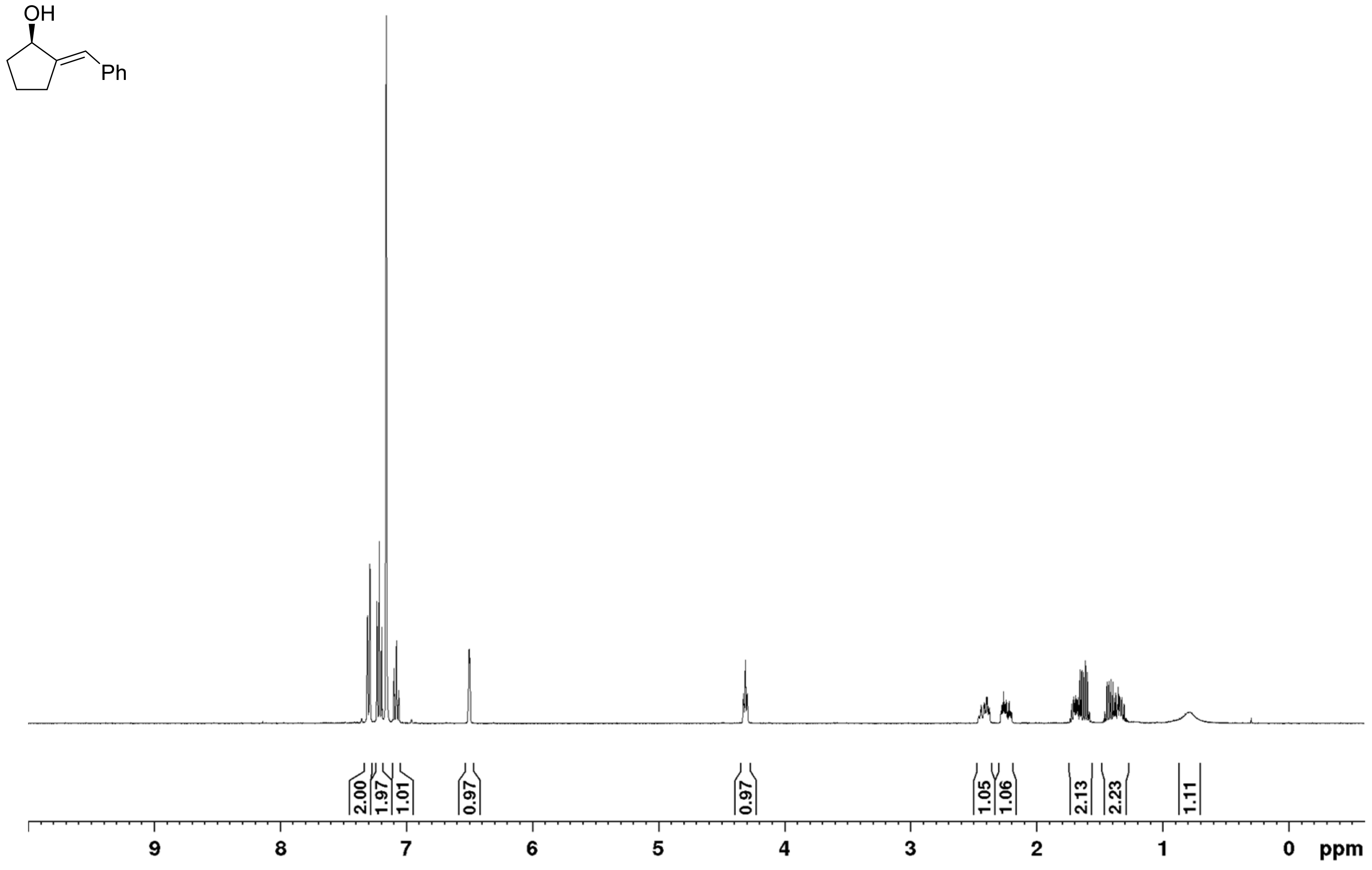

Supplementary Figure 242. ^{13}**C NMR (126 MHz, CDCl$_3$) of (*R*,*E*)-2-Benzylidenecyclopentan-1-ol [(*R*)-6e]**

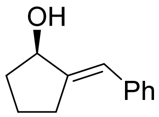

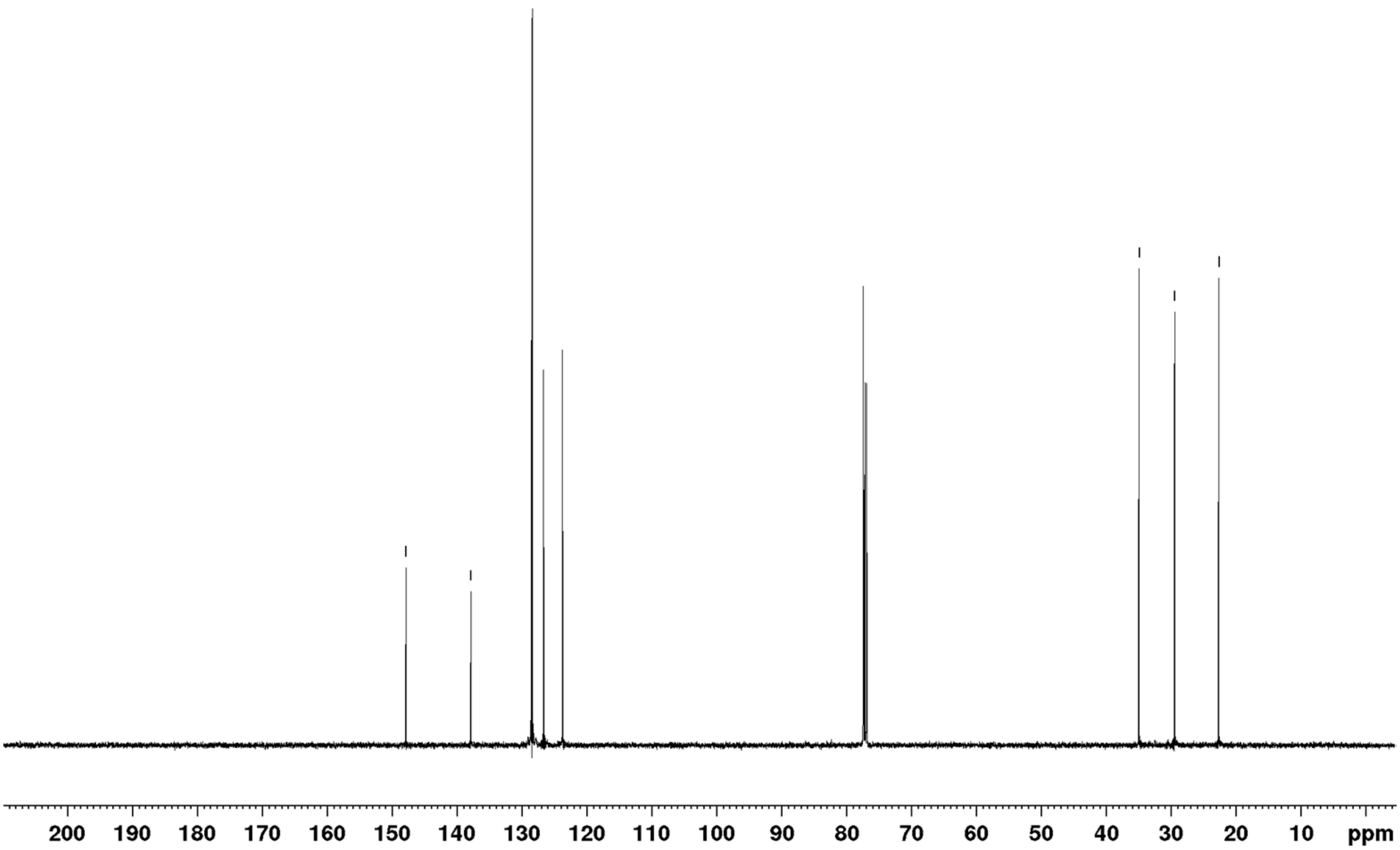

Supplementary Figure 243. ^{1}H NMR (500 MHz, C_6D_6) of (*S*,*E*)-((2-Benzylidenecyclopentyl)oxy)tributylsilane [(*S*)-7eh]

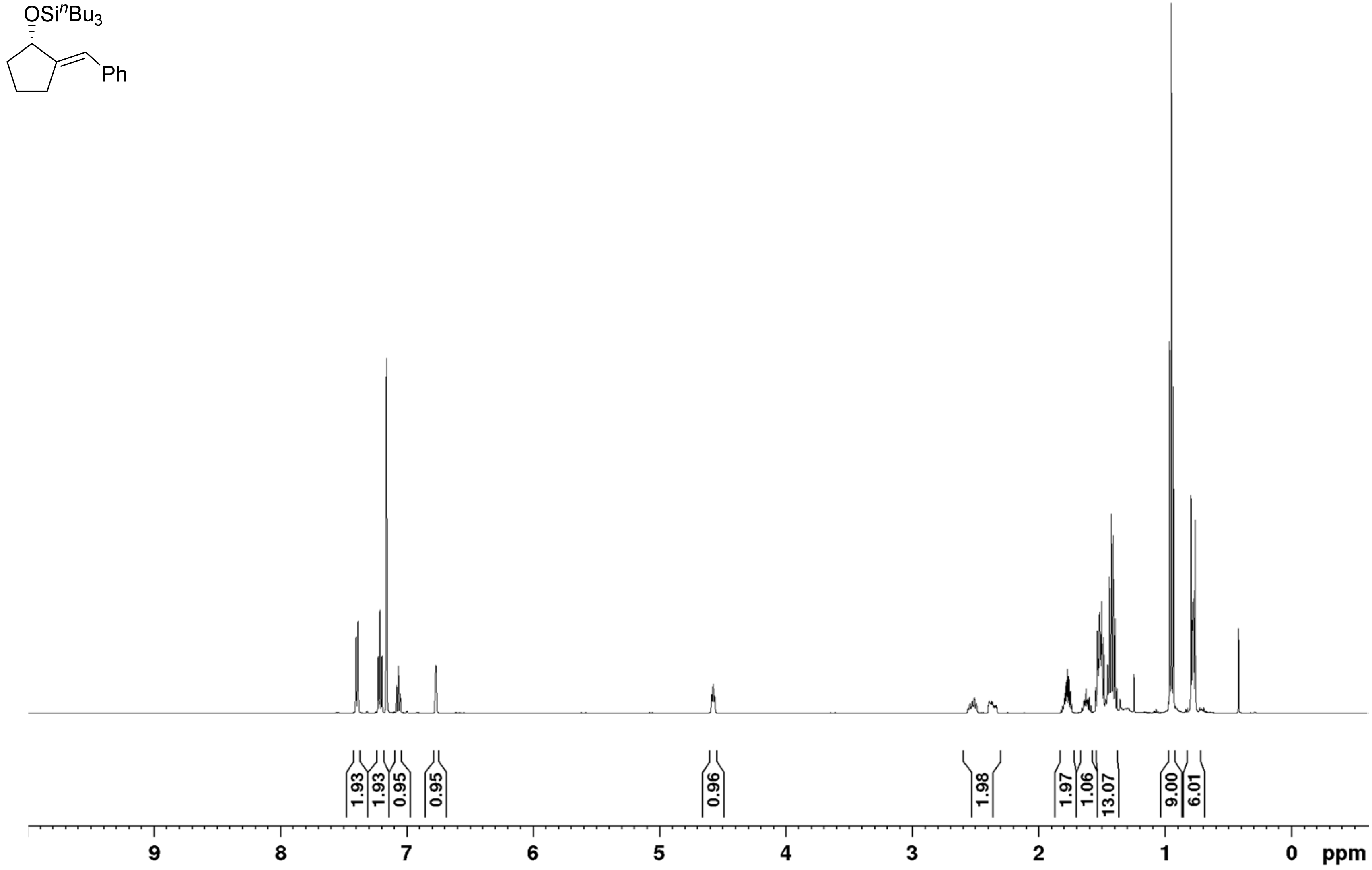

Supplementary Figure 244. ^{13}**C NMR (126 MHz,** C_6D_6**) of (*S*,*E*)-((2-Benzylidenecyclopentyl)oxy)tributylsilane [(*S*)-7eh]**

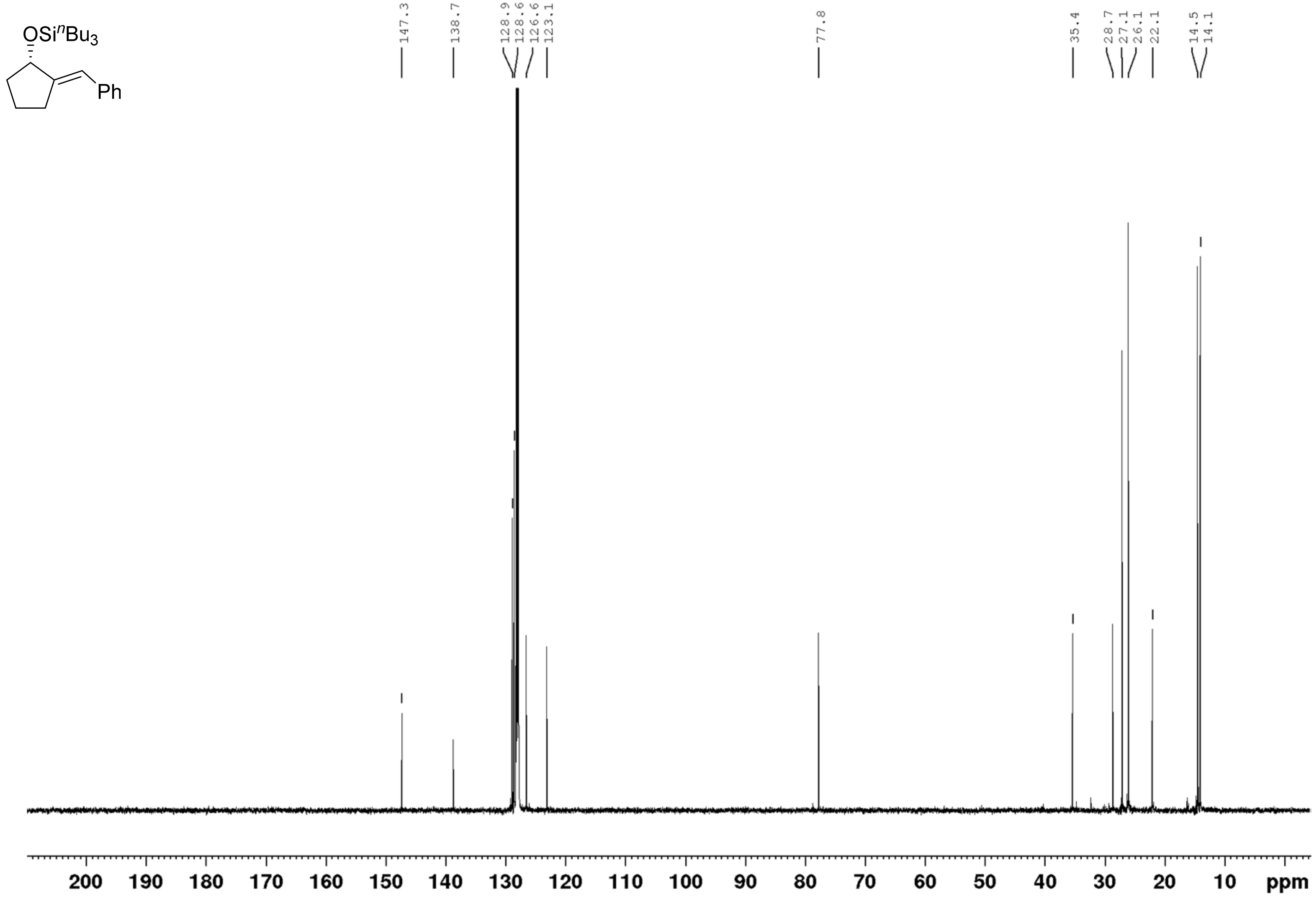

Supplementary Figure 245. ^{1}H NMR (400 MHz, $CDCl_3$) of (*R*,*E*)-2-Benzylidenecyclohexan-1-ol [(*R*)-6f]

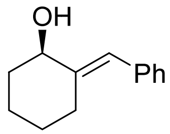

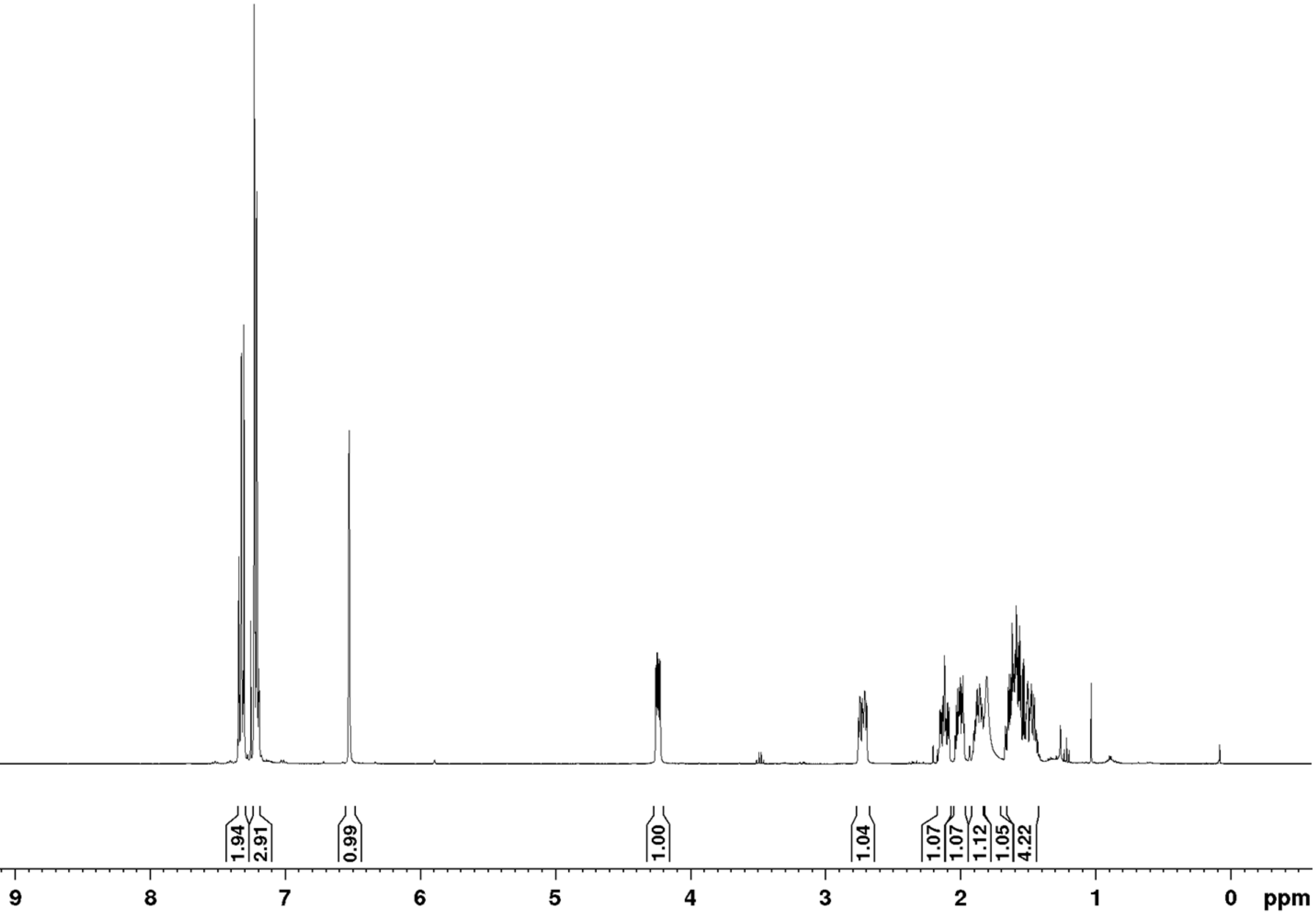

Supplementary Figure 246. ^{13}C NMR (126 MHz, $CDCl_3$) of (*R*,*E*)-2-Benzylidenecyclohexan-1-ol [(*R*)-6f]

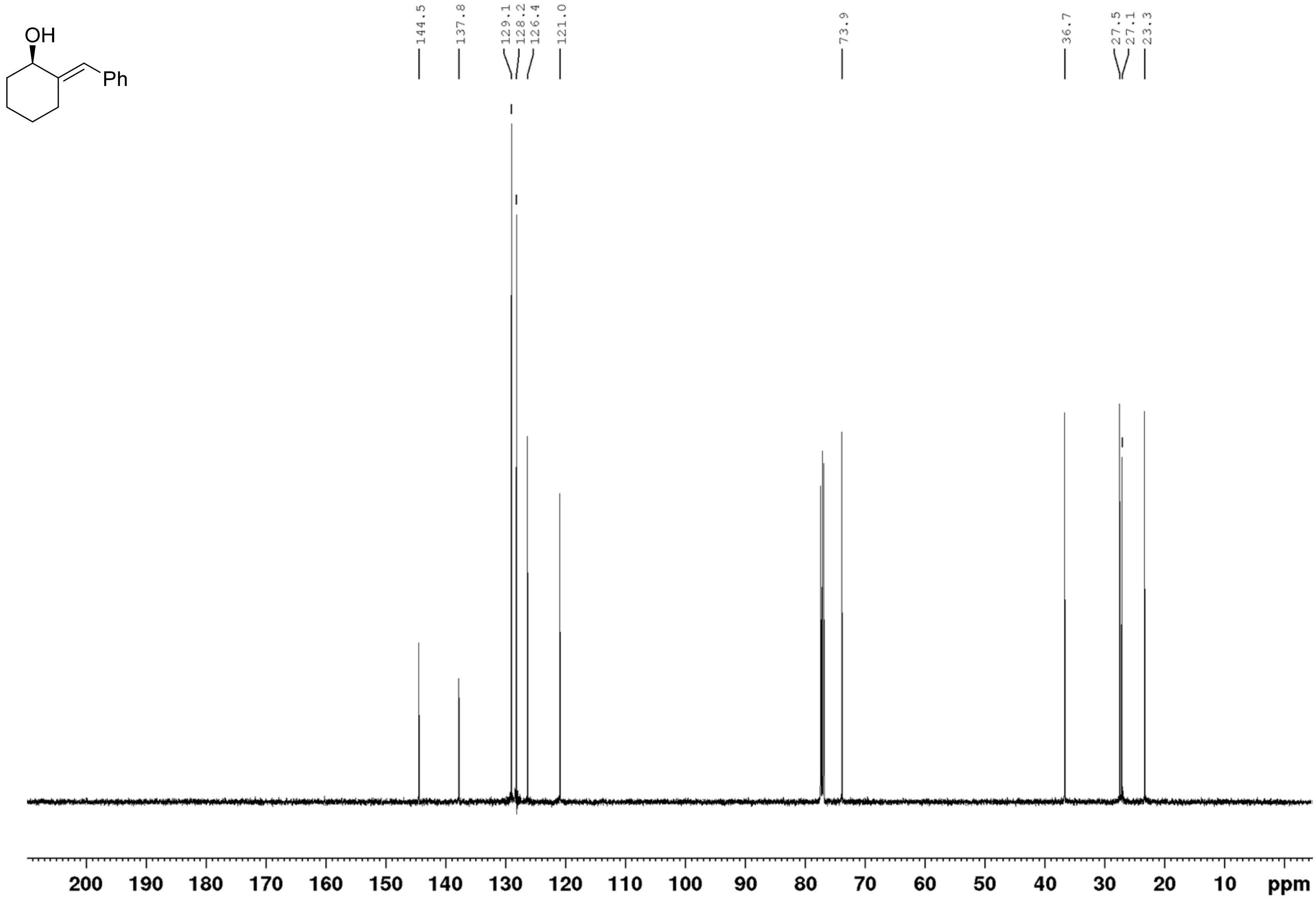

Supplementary Figure 247. [1]**H NMR (500 MHz, C_6D_6) of (*S*,*E*)-((2-Benzylidenecyclohexyl)oxy)tributylsilane [(*S*)-7fh]**

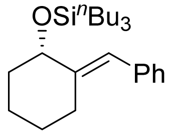

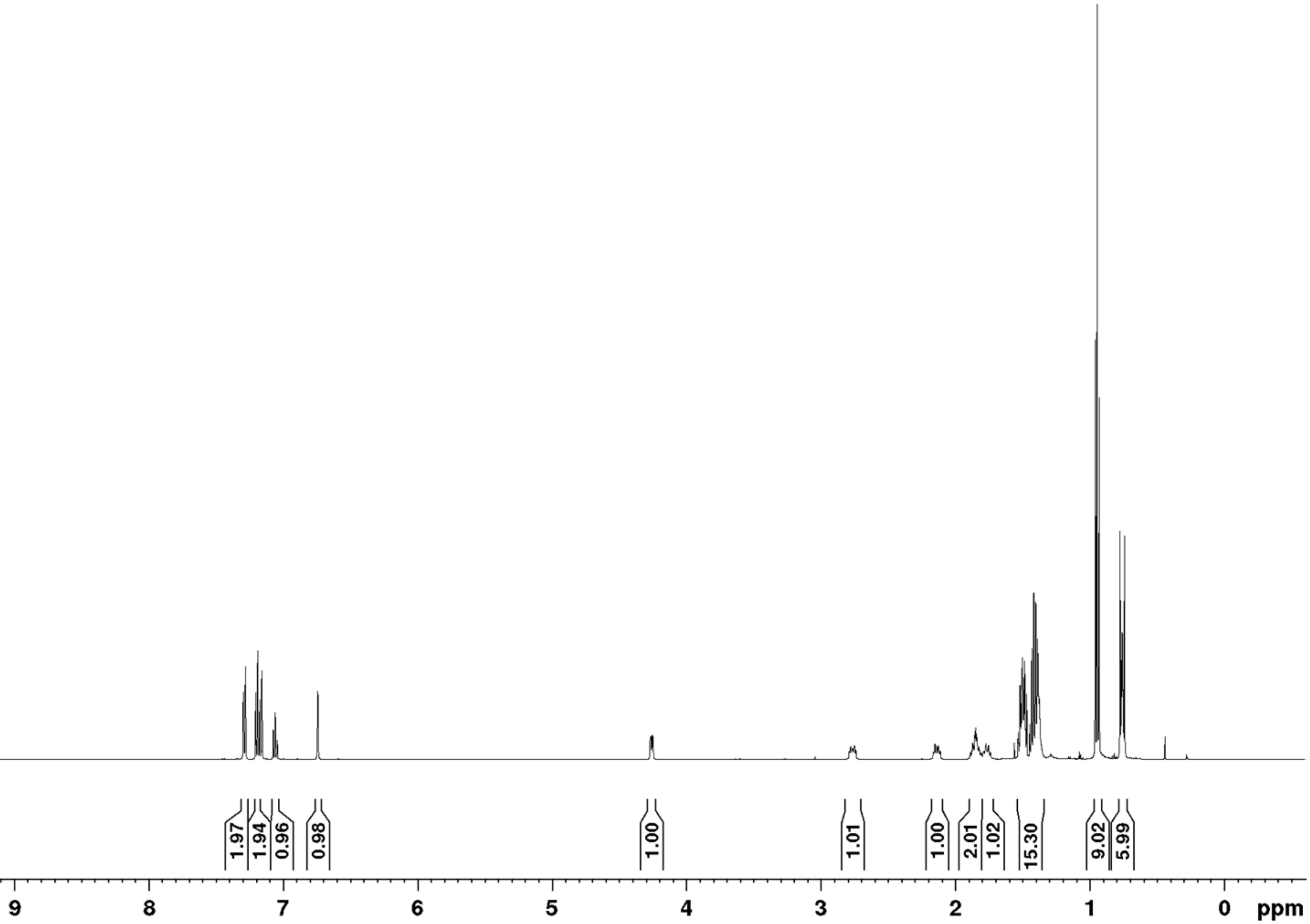

Supplementary Figure 248. ^{13}**C NMR (126 MHz, C_6D_6) of (*S*,*E*)-((2-Benzylidenecyclohexyl)oxy)tributylsilane [(*S*)-7fh]**

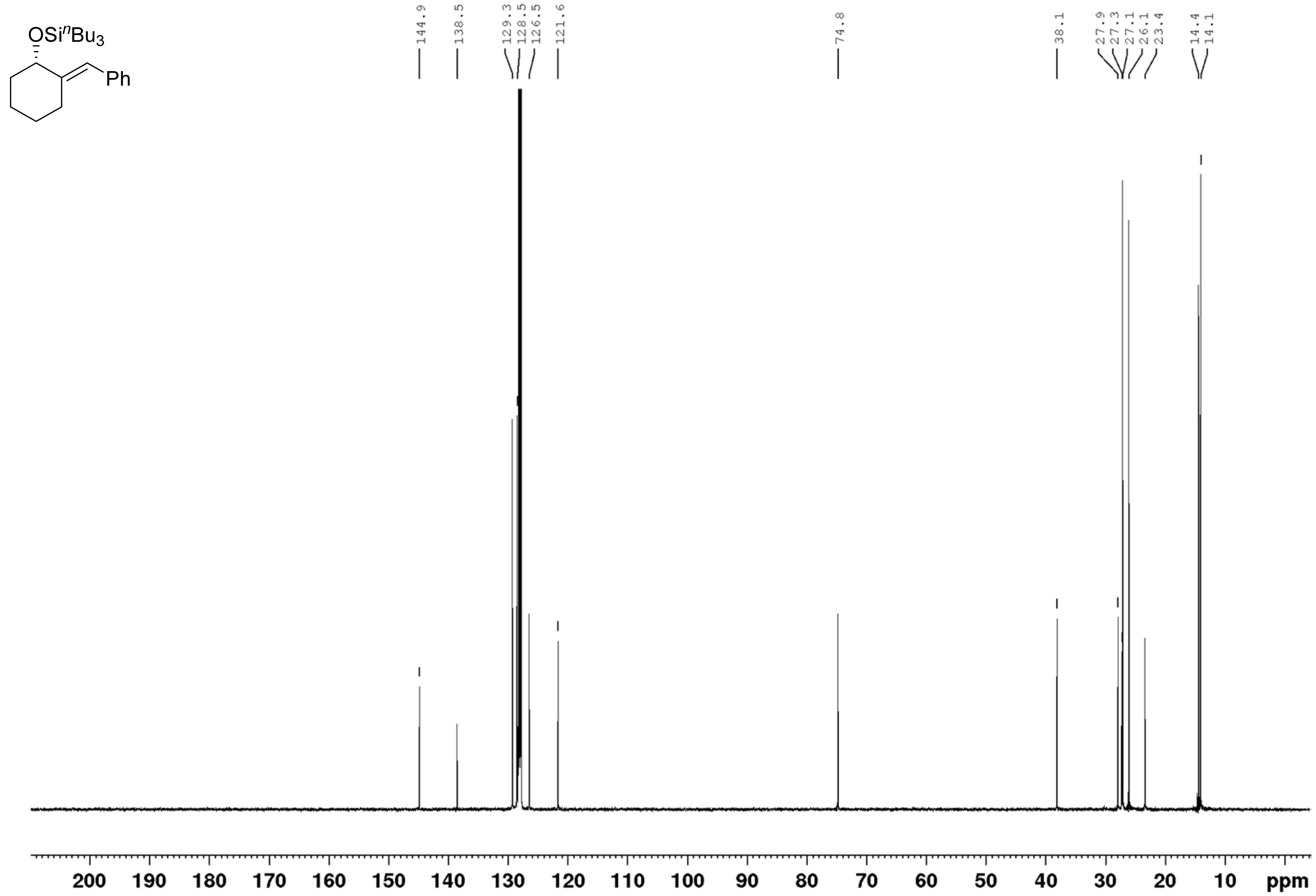

Supplementary Figure 249. [1]**H NMR (400 MHz, C_6D_6) of (*R*)-2,3,4,5-Tetrahydro-[1,1'-biphenyl]-2-ol [(*R*)-8]**

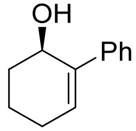

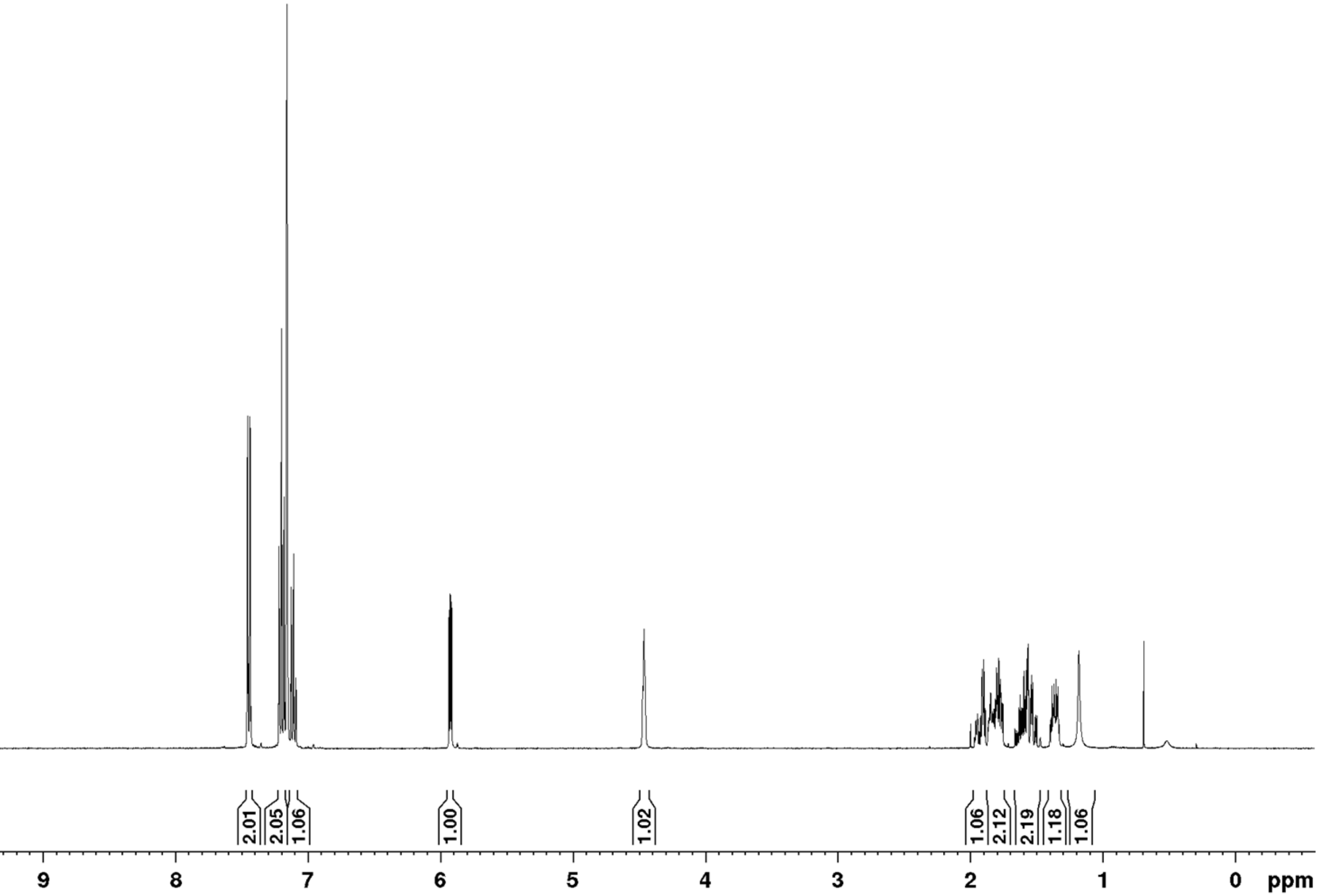

Supplementary Figure 250. ^{13}C NMR (126 MHz, $CDCl_3$) of (*R*)-2,3,4,5-Tetrahydro-[1,1'-biphenyl]-2-ol [(*R*)-8]

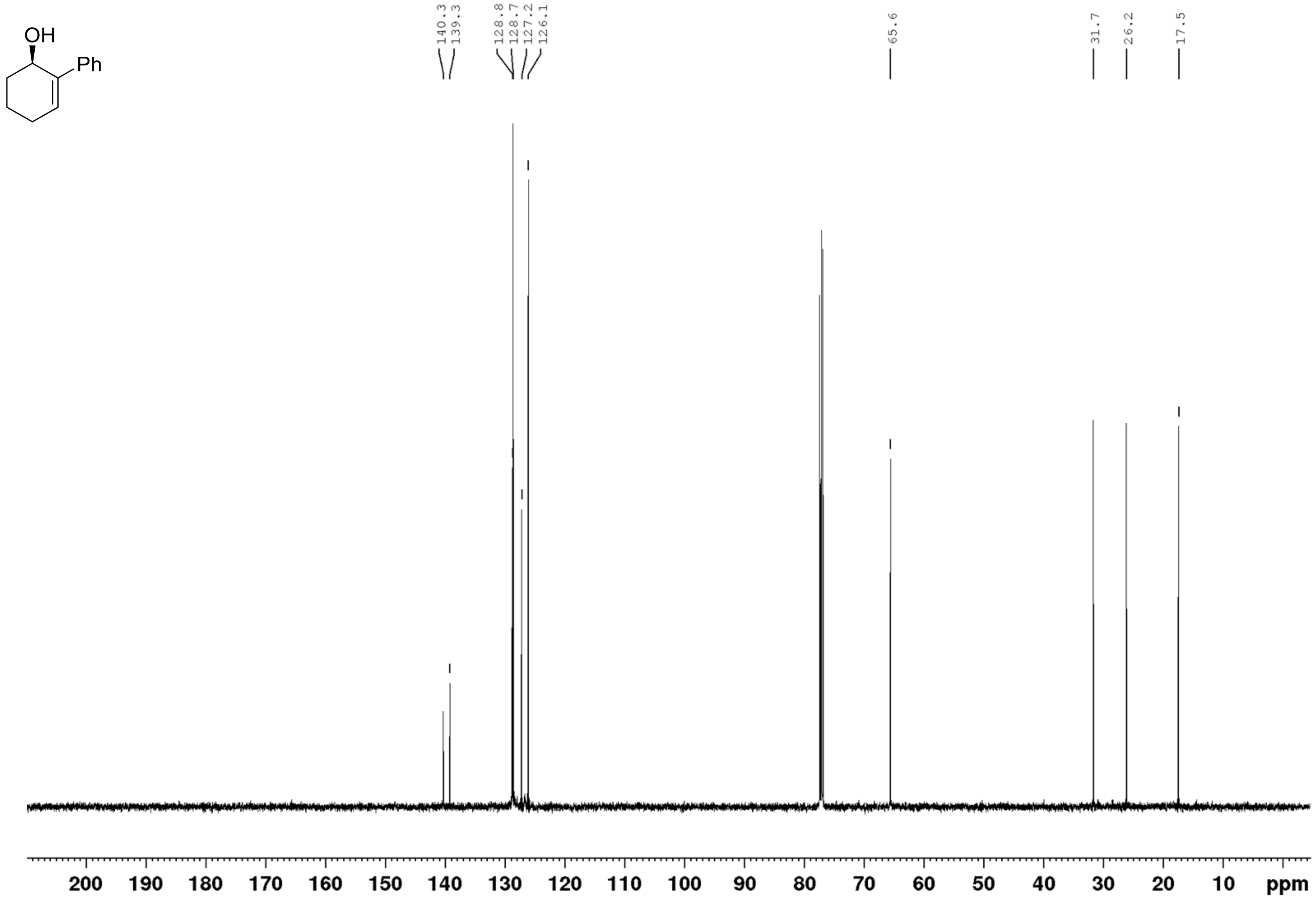

Supplementary Figure 251. ^{1}H NMR (400 MHz, C_6D_6) of (*S*)-Tributyl((2,3,4,5-tetrahydro-[1,1'-biphenyl]-2-yl)oxy)silane [(*S*)-9h]

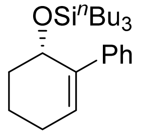

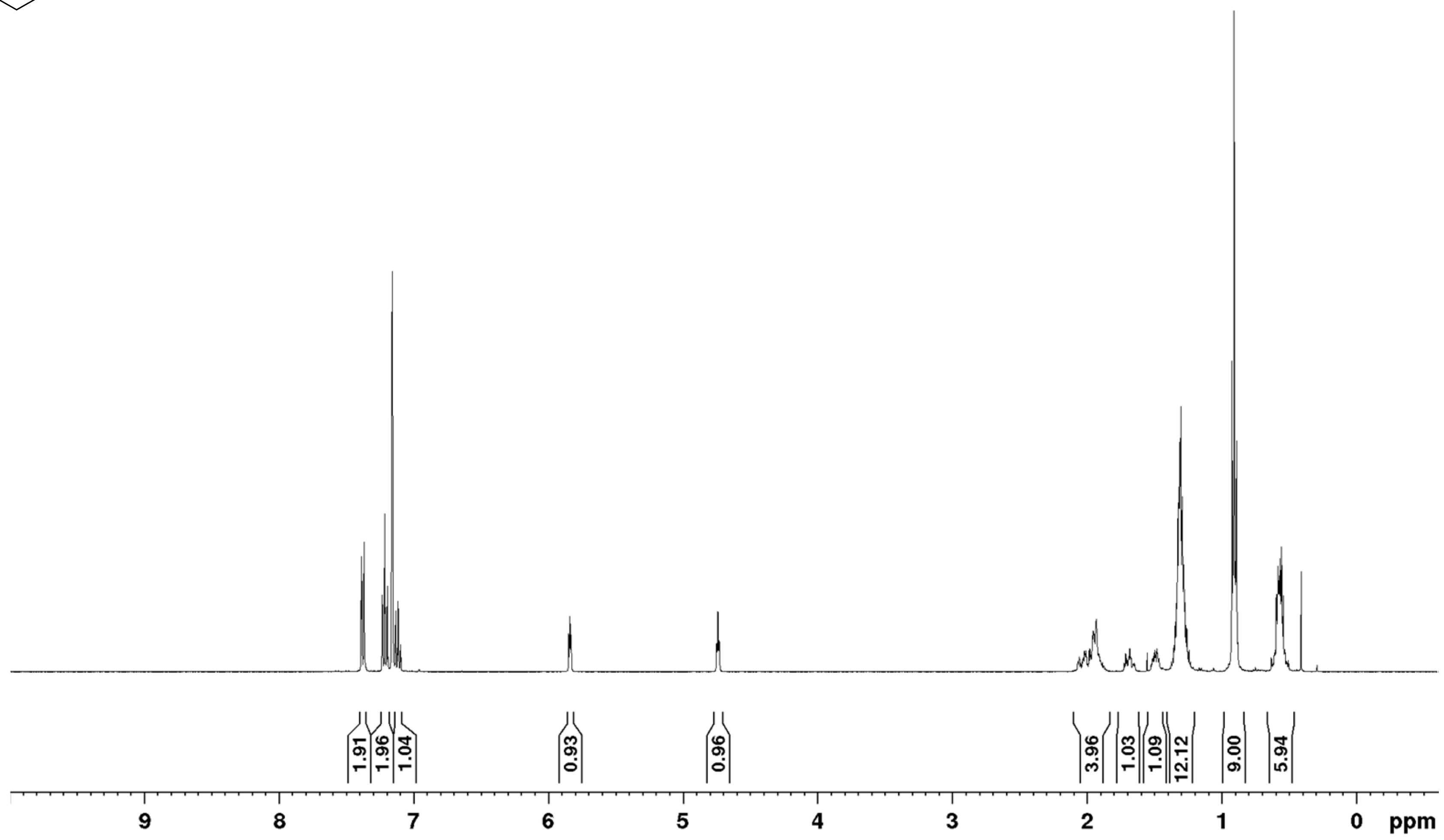

Supplementary Figure 252. ^{13}C NMR (126 MHz, C_6D_6) of (*S*)-Tributyl((2,3,4,5-tetrahydro-[1,1'-biphenyl]-2-yl)oxy)silane [(*S*)-9h]

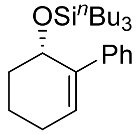

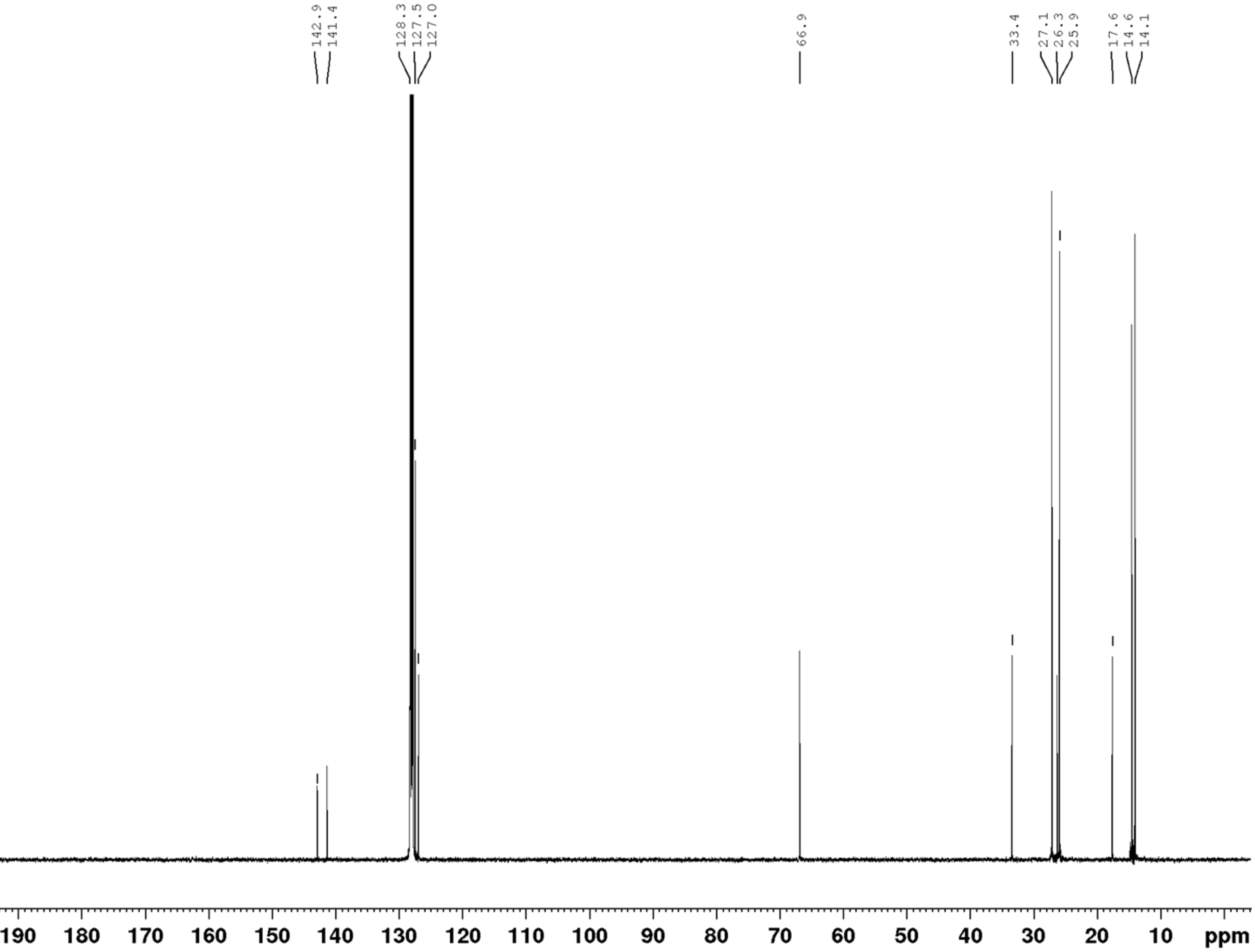

Supplementary Figure 253. ^{1}H NMR (400 MHz, $CDCl_3$) of (*R*)-1-Cyclohexylethan-1-ol [(*R*)-10]

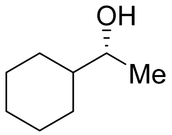

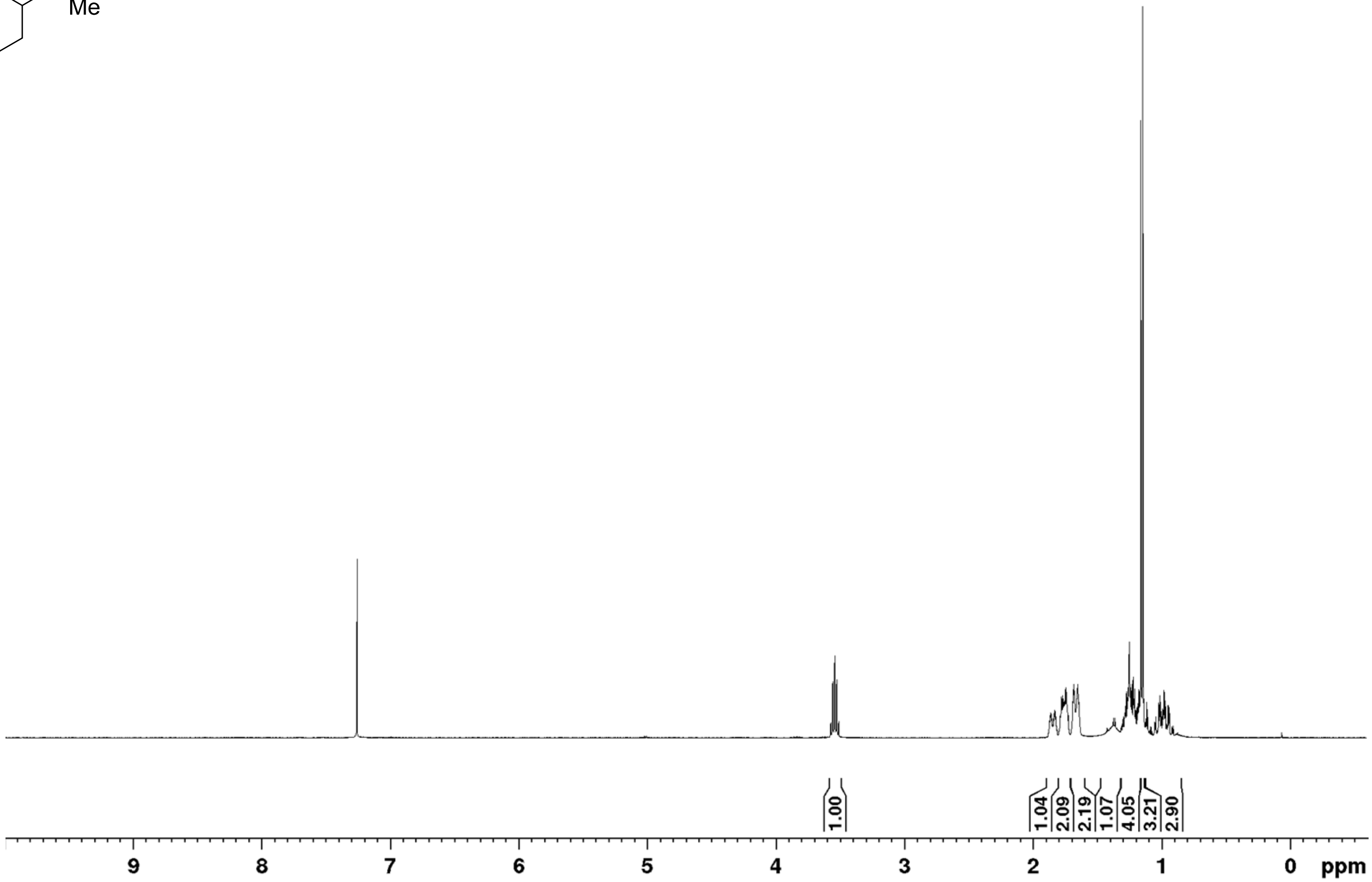

Supplementary Figure 254. **^{13}C NMR (101 MHz, $CDCl_3$) of (*R*)-1-Cyclohexylethan-1-ol [(*R*)-10]**

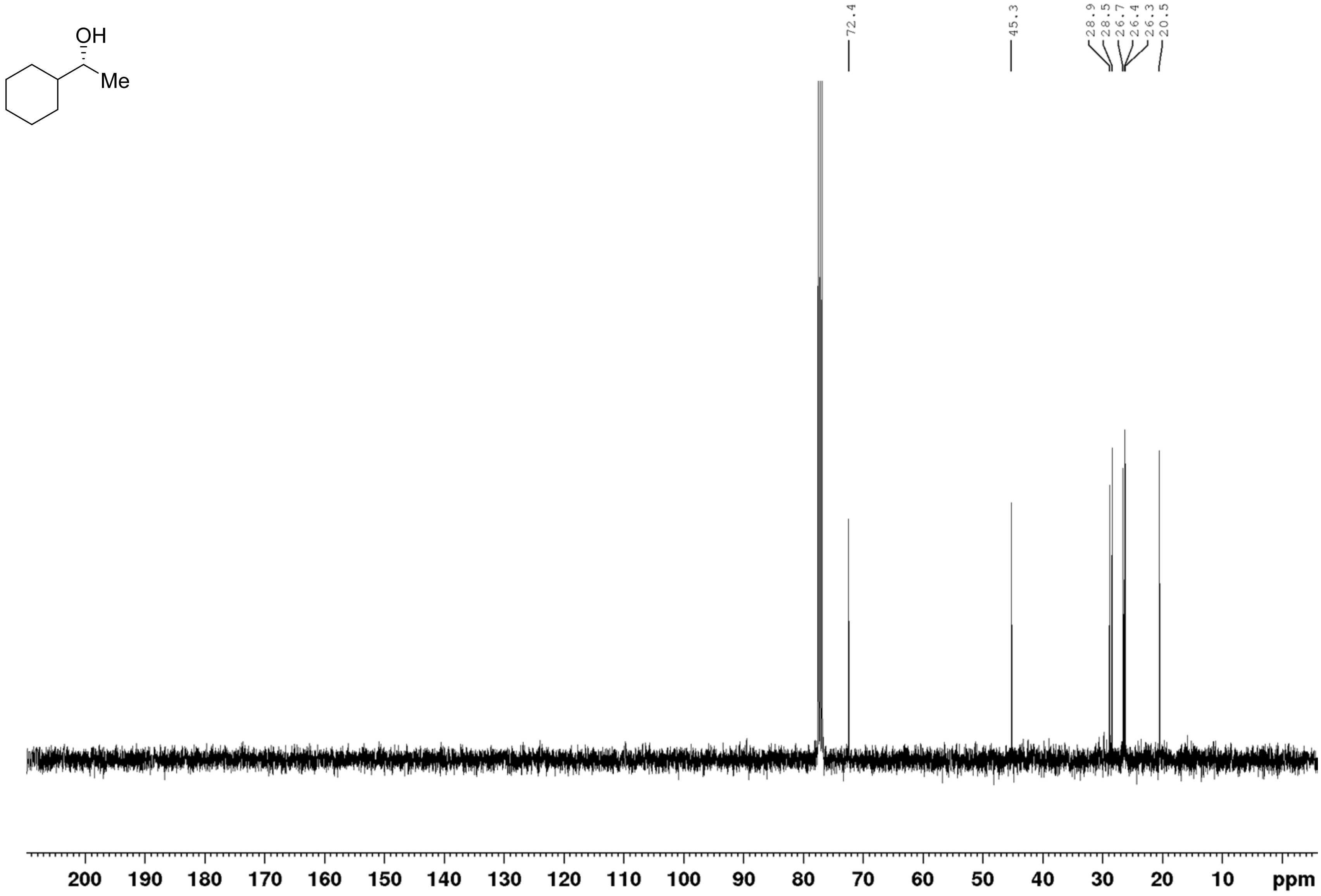

Supplementary Figure 255. ^{1}**H NMR (400 MHz, C_6D_6) of (*S*)-Tributyl(1-cyclohexylethoxy)silane [(*S*)-11h]**

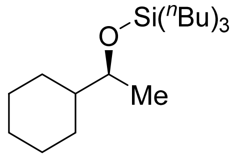

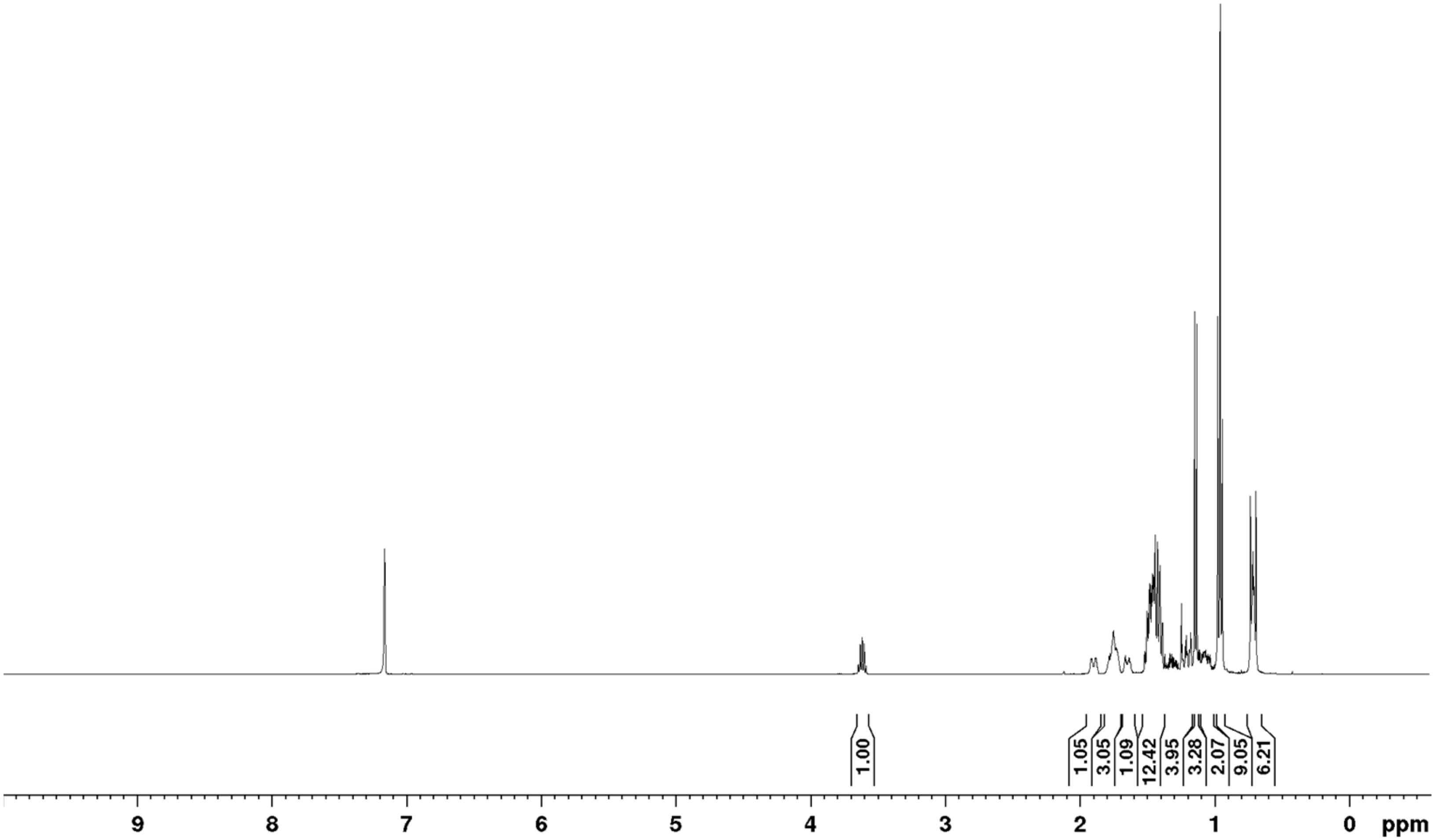

Supplementary Figure 256. ^{13}**C NMR (101 MHz, C_6D_6) of (*S*)-Tributyl(1-cyclohexylethoxy)silane [(*S*)-11h]**

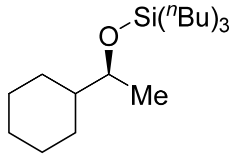

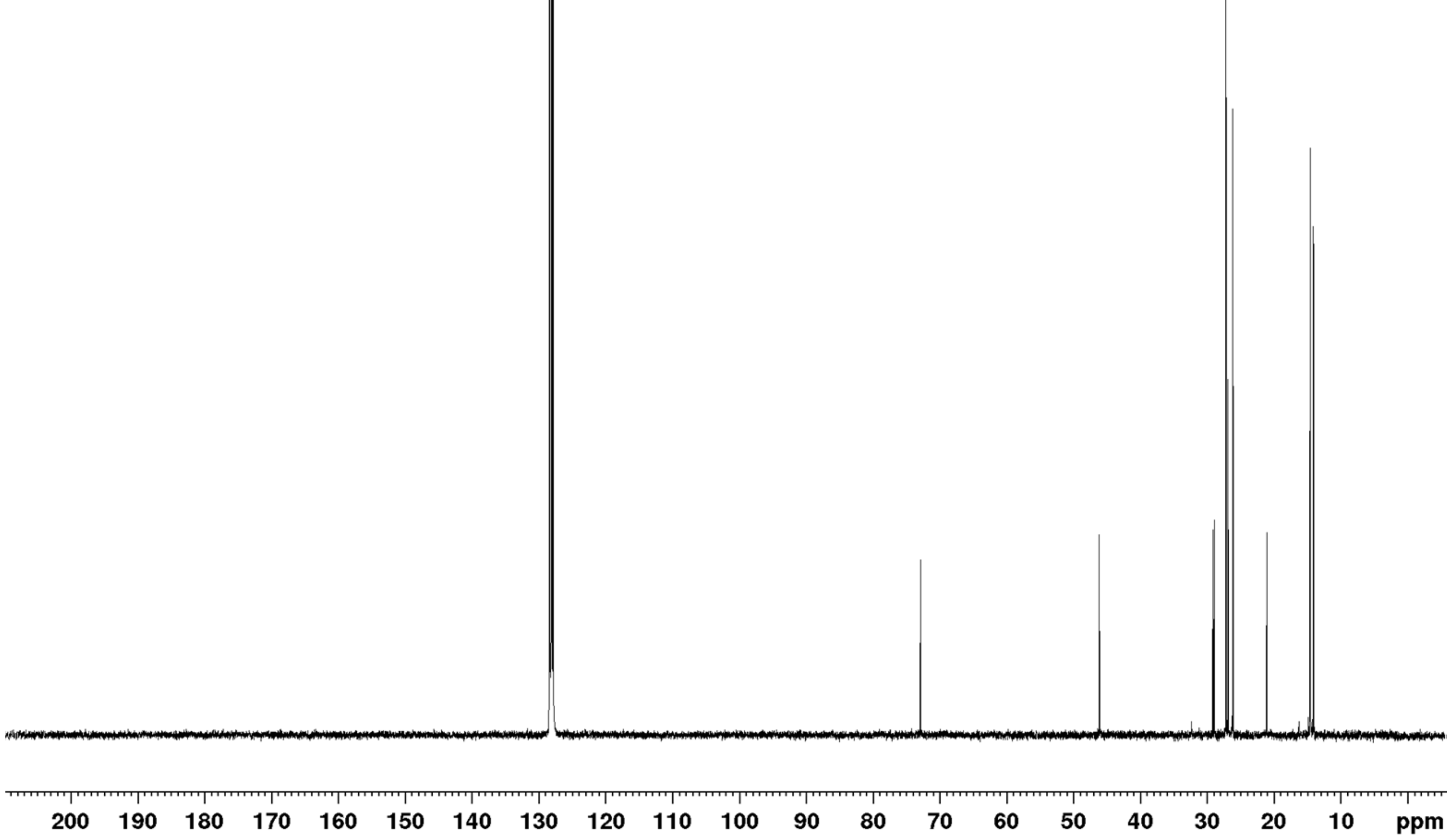

Supplementary References

[1] Sheppard, C. I., Taylor, J. L. & Wiskur, S. L. Silylation-Based Kinetic Resolution of Monofunctional Secondary Alcohols. *Org. Lett.* **13**, 3794–3797 (2011).

[2] Noji, M., Konno, Y. & Ishii, K. Metal Triflate-Catalyzed Cationic Benzylation and Allylation of 1,3-Dicarbonyl Compounds. *J. Org. Chem.* **72**, 5161–5167 (2007).

[3] Gemal, A. L. & Luche, J.-L. Lanthanoids in Organic Synthesis. 6. The Reduction of *α*-Enones by Sodium Borohydride in the Presence of Lanthanoid Chlorides: Synthetic and Mechanistic Aspects. *J. Am. Chem. Soc.* **103**, 5454–5459 (1981).

[4] Weickgenannt, A., Mewald, M., Muesmann, T. W. T. & Oestreich, M. Catalytic Asymmetric Si–O Coupling of Simple Achiral Silanes and Chiral Donor-Functionalized Alcohols. *Angew. Chem. Int. Ed.* **49**, 2223–2226 (2010).

[5] Bender, A. M., Griggs, N. W., Anand, J. P., Traynor, J. R., Jutkiewicz, E. M. & Mosberg, H. I. Asymmetric Synthesis and in Vitro and in Vivo Activity of Tetrahydroquinolines Featuring a Diverse Set of Polar Substitutions at the 6 Position as Mixed-Efficacy *μ* Opioid Receptor/*δ* Opioid Receptor Ligands. *ACS Chem. Neurosci.* **6**, 1428–1435 (2015).

[6] Lu, S.-M. & Bolm, C. Highly Enantioselective Synthesis of Optically Active Ketones by Iridium-Catalyzed Asymmetric Hydrogenation. *Angew. Chem. Int. Ed.* **47**, 8920–8923 (2008).

[7] Kowalski, C. J., Weber, A. E. & Fields, K. W. *α*-Keto Dianion Precursors via Conjugate Additions to Cyclic *α*-Bromo Enones. *J. Org. Chem.* **47**, 5088–5093 (1982).

[8] Germain, N. & Alexakis, A. Formation of Contiguous Quaternary and Tertiary Stereocenters by Sequential Asymmetric Conjugate Addition of Grignard Reagents to 2-Substituted Enones and Mg–Enolate Trapping. *Chem. Eur. J*. **21**, 8597–8606 (2015).

[9] Süsse, L., Hermeke, J. & Oestreich, M. The Asymmetric Piers Hydrosilylation. *J. Am.Chem. Soc.* **138**, 6940–6943 (2016).

[10] Chen, X. & Lu, Z. Iminophenyl Oxazolinylphenylamine for Enantioselective Cobalt-Catalyzed Hydrosilylation of Aryl Ketones. *Org. Lett.* **18**, 4658–4661 (2016).

[11] Cherng, Y.-J., Fang, J.-M. & Lu, T.-J. Pinane-Type Tridentate Reagents for Enantioselective Reactions: Reduction of Ketones and Addition of Diethylzinc to Aldehydes. *J. Org. Chem.* **64**, 3207–3212 (1999).

[12] Kobayashi, Y., Kodama, K. & Saigo, K. Supramolecular Architecture Consisting of an Enantiopure Amine and an Achiral Carboxylic Acid: Application to the Enantioseparation of Racemic Alcohols. *Org. Lett.* **6**, 2941–2944 (2004).

[13] Tian, C., Gong, L. & Meggers, E. Chiral-at-metal iridium complex for efficient enantioselective transfer hydrogenation of ketones. *Chem. Commun.* **52**, 4207–4210

(2016).
[14] Hodgkinson, R., Jurčik, V., Zanotti-Gerosa, A., Nedden, H. G., Blackaby, A., Clarkson, G. J. & Wills, M. Synthesis and Catalytic Applications of an Extended Range of Tethered Ruthenium(II)/η^6-Arene/Diamine Complexes. *Organometallics* **33**, 5517–5524 (2014).
[15] Wettergren, J., Bøgevig, A., Portier, M. & Adolfsson, H. Ruthenium-Catalyzed Enantioselective Reduction of Electron-Rich Aryl Alkyl Ketones. *Adv. Synth. Catal.* **348**, 1277–1282 (2006).
[16] Spivey, A. C., Leese, D. P., Zhu, F., Davey, S. G. & Jarvest, R. L. New atropisomeric biaryl derivatives of 4-aminopyridine—identification of an improved nucleophilic catalyst for asymmetric acylation of *sec*-alcohols. *Tetrahedron* **60**, 4513–4525 (2004).
[17] Spivey, A. C., Fekner, T. & Spey, S. E. Axially Chiral Analogues of 4-(Dimethylamino)pyridine: Novel Catalysts for Nonenzymatic Enantioselective Acylations. *J. Org. Chem.* **65**, 3154–3159 (2000).
[18] Corey, E. J., Cheng, X.-M., Cimprich, K. A. & Sarshar, S. Remarkably effective and simple syntheses of enantiomerically pure secondary carbinols from achiral ketones. *Tetrahedron Lett.* **32**, 6835–6838 (1991).
[19] Williams, D. R. & Fromhold, M. G. Enantioselective Alkylation of Aldehydes with Diethylzinc Catalyzed by C_2-Symmetric Ligands. *Synlett* 523–524 (1997).
[20] Hatano, M., Miyamoto, T. & Ishihara K. 3,3'-Diphosphoryl-1,1'-bi-2-naphthol−Zn(II) Complexes as Conjugate Acid−Base Catalysts for Enantioselective Dialkylzinc Addition to Aldehydes. *J. Org. Chem.* **71**, 6474–6484 (2006).
[21] Zuo, Z., Zhang, L., Leng, X. & Huang, Z. Iron-catalyzed asymmetric hydrosilylation of ketones. ***Chem. Commun.*** **51**, 5073–5076 (2015).
[22] Ren, X., Li, G., Wei, S. & Du, H. Facile Development of Chiral Alkenylboranes from Chiral Diynes for Asymmetric Hydrogenation of Silyl Enol Ethers, *Org. Lett.* **17**, 990–993 (2015).
[23] Kišić, A., Stephan, M. & Mohar, B. *ansa*-Ruthenium(II) Complexes of R_2NSO_2DPEN-$(CH_2)_n$(η^6-Aryl) Conjugate Ligands for Asymmetric Transfer Hydrogenation of Aryl Ketones. *Adv. Synth. Catal.* **357**, 2540–2546 (2015).
[24] Zhou, X., Zheng, D., Cui, B., Han, W. & Chen, Y. Novozyme 435 lipase mediated enantioselective kinetic resolution: a facile method for the synthesis of chiral tetrahydroquinolin-4-ol and tetrahydro-1*H*-benzo[*b*]azepin-5-ol derivatives, *Tetrahedron* **71**, 4738–4744 (2015).
[25] Murakami, K., Sasano, Y., Tomizawa, M., Shibuya, M., Kwon, E. & Iwabuchi, Y. Highly Enantioselective Organocatalytic Oxidative Kinetic Resolution of Secondary Alcohols Using Chiral Alkoxyamines as Precatalysts: Catalyst Structure, Active Species, and Substrate Scope, *J. Am. Chem. Soc.* **136**, 17591–17600 (2014).

[26] Wang, L., Akhani, R. K. & Wiskur, S. L. Diastereoselective and Enantioselective Silylation of 2-Arylcyclohexanols, *Org. Lett.* **17**, 2408−2411 (2015).

[27] Peach, P., Cross, D. J., Kenny, J. A., Mann, I., Houson, I., Campbell, L., Walsgrove, T. & Wills, M. Asymmetric transfer hydrogenation of *α*,*β*-unsaturated, *α*-tosyloxy and *α*-substituted ketones, *Tetrahedron* **62**, 1864–1876 (2006).

[28] Moser, R., Bošković, Ž. V., Crowe, C. S. & Lipshutz, B. H. CuH-Catalyzed Enantioselective 1,2-Reductions of *α*,*β*-Unsaturated Ketones, *J. Am. Chem. Soc.* **132**, 7852–7853 (2010).

[29] Zhang, Q.-Q., Xie, J.-H., Yang, X.-H., Xie, J.-B. & Zhou, Q.-L. Iridium-Catalyzed Asymmetric Hydrogenation of *α*-Substituted *α*,*β*-Unsaturated Acyclic Ketones: Enantioselective Total Synthesis of (–)-Mesembrine, *Org. Lett.* **14**, 6158–6161 (2012).

[30] Li, D. R., He, A. & Falck, J. R. Enantioselective, Organocatalytic Reduction of Ketones using Bifunctional Thiourea-Amine Catalysts, *Org. Lett*. **12**, 1756–1759 (2010).

[31] Xie, J.-B., Xie, J.-H., Liu, X.-Y., Kong, W.-L., Li, S. & Zhou, Q.-L. Highly Enantioselective Hydrogenation of *α*-Arylmethylene Cycloalkanones Catalyzed by Iridium Complexes of Chiral Spiro Aminophosphine Ligands, *J. Am. Chem. Soc.* **132**, 4538–4539 (2010).

[32] Kim, J., Bruning, J., Park, K. E., Lee, D. J. & Singaram, B. Highly Enantioselective and Regioselective Carbonyl Reduction of Cyclic *α*,*β*-Unsaturated Ketones Using TarB-NO_2 and Sodium Borohydride, *Org. Lett.* **11**, 4358–4361 (2009).
